# Patterns of prokaryotic lateral gene transfers affecting parasitic microbial eukaryotes

**DOI:** 10.1186/gb-2013-14-2-r19

**Published:** 2013-02-25

**Authors:** Cecilia Alsmark, Peter G Foster, Thomas Sicheritz-Ponten, Sirintra Nakjang, T Martin Embley, Robert P Hirt

**Affiliations:** 1 Institute for Cell and Molecular Biosciences, Newcastle University, Newcastle upon Tyne, NE2 4HH, UK; 2 Division of Pharmacognosy, Department of Medicinal Chemistry, Uppsala University, Biomedical Centre, S-751 23 Uppsala, Sweden; 3 Department of Life Sciences, Natural History Museum, Cromwell Road, London, SW7 5BD, UK; 4 Center for Biological Sequence Analysis, Department of Systems Biology, Technical University of Denmark, DK-2800 Lyngby and Novo Nordisk Foundation Center for Biosustainability, DK-2900 Hørsholm, Denmark

**Keywords:** Genome evolution, phylogenomics, lateral gene transfer, eukaryotes, parasites

## Abstract

**Background:**

The influence of lateral gene transfer on gene origins and biology in eukaryotes is poorly understood compared with those of prokaryotes. A number of independent investigations focusing on specific genes, individual genomes, or specific functional categories from various eukaryotes have indicated that lateral gene transfer does indeed affect eukaryotic genomes. However, the lack of common methodology and criteria in these studies makes it difficult to assess the general importance and influence of lateral gene transfer on eukaryotic genome evolution.

**Results:**

We used a phylogenomic approach to systematically investigate lateral gene transfer affecting the proteomes of thirteen, mainly parasitic, microbial eukaryotes, representing four of the six eukaryotic super-groups. All of the genomes investigated have been significantly affected by prokaryote-to-eukaryote lateral gene transfers, dramatically affecting the enzymes of core pathways, particularly amino acid and sugar metabolism, but also providing new genes of potential adaptive significance in the life of parasites. A broad range of prokaryotic donors is involved in such transfers, but there is clear and significant enrichment for bacterial groups that share the same habitats, including the human microbiota, as the parasites investigated.

**Conclusions:**

Our data show that ecology and lifestyle strongly influence gene origins and opportunities for gene transfer and reveal that, although the outlines of the core eukaryotic metabolism are conserved among lineages, the genes making up those pathways can have very different origins in different eukaryotes. Thus, from the perspective of the effects of lateral gene transfer on individual gene ancestries in different lineages, eukaryotic metabolism appears to be chimeric.

## Background

The protein-coding capacity of a genome is the product of a history of gene acquisitions and losses [1,2]. New genes can be created *de novo*, through gene fusions, gene duplications, and lateral gene transfer (LGT), and collectively, they may contribute to adaptive innovations [1]. LGT is the transfer and fixation of genetic material between distinct lineages independent of their reproduction cycle. LGT is now widely accepted as a major factor shaping the gene content of prokaryotic genomes in both free-living and host-dependent lineages [3,4]. Although LGT has not been studied so extensively among eukaryotes, it is already apparent that LGT has also affected eukaryotic genomes [5-7]. Thus, it has been recognized for some time that eukaryotic metabolism seems to be more similar to bacterial metabolism than to archaebacterial metabolism [8,9]. These bacterial-like genes and pathways may represent the legacy of founding bacterial partners in eukaryogenesis [10] or result from endosymbiotic gene transfers (EGTs) [11,12]. For example, it has been suggested that EGTs from the mitochondrial endosymbiont might be the source of around 600 to 800 protein-coding genes in eukaryotic nuclear genomes [12,13], and gene transfer from photosynthetic endosymbionts has additionally affected the genome content of algae and plants [12]. Gene transfers from more recent bacterial endosymbionts have also affected the genomes of some eukaryotic lineages [7,12]. Beyond endosymbiosis, it is clear that LGTs from diverse prokaryotes have also affected many protists [14-16]. Although many of these LGTs seem to represent homologous replacements of genes for existing pathways, there are also cases where LGT has conferred entirely novel functions. For example, the transfer of genes for bacterial-like nucleotide transporters to microsporidian parasites underpins their obligate intracellular lifestyle by allowing them to steal ATP from their host cells [17]. On a global economic scale, the LGT of genes for toxins between fungal plant pathogens has had a devastating impact on wheat production [18].

A range of different methods have been used to detect LGTs, with varying degrees of agreement between methods [19]. Detailed phylogenetic analyses are probably the most rigorous approach [19], but can be time-consuming for large numbers of genes, requiring a trade-off between analytical sophistication and speed. One solution has been to combine less sophisticated but rapid tree-building methods with fast non-tree-based approaches to provide a primary screen for potential LGTs that can then be subjected to more detailed analysis using better phylogenetic models [14,15]. In the present investigation, we applied this combined approach to systematically identify LGTs affecting the genomes of 13 taxonomically diverse, mostly parasitic, microbial eukaryotes (Table 1), including a number of major parasites of humans and livestock [20]. Some of these parasites occupy different niches within their hosts, providing an opportunity to investigate how patterns of LGTs and potential donor lineages might be influenced by the habitat(s) in which they live. For comparison, we also analyzed the genome of *Dictyostelium discoideum *[21], a free-living amoebozoan relative of the parasite *Entamoeba histolytica*, which lives in soil. Some of the parasites we investigated, including species of *Leishmania *and *Trypanosoma*, are closely related to each other, providing comparative insight into LGT over shorter timescales. Our systematic analyses provide a detailed insight into the dynamics, role, and potential importance of LGT in the evolution of a sample of parasitic microbial eukaryotes, but also have general implications for understanding how eukaryotic genomes and metabolic pathways have evolved.

**Table 1:** Overview of genomes analyzed and the number and type of lateral gene transfer (LGTs) detected

Taxa	Number of genes^a^	Number of trees^b^	P to E^c^, n	E to E^d^, n	LGT^e^	Percentage LGT,%^f^
*Leishmania major*	7,111	4,638	63	5	68	0.96

*Entamoeba histolytica*	9,090	6,331	51	12	63	0.68

*Trypanosoma bruceii*	9,750	6,191	45	1	46	0.47

*Dictyostelium discoideum*	13,605	9,921	61	1	62	0.46

*Plasmodium falciparum*	5,258	4,546	18	1	19	0.36

*Giardia lamblia*	6,394	1,923	15	6	21	0.36

*Plasmodium vivax*	5,393	3,766	17	0	17	0.32

*Cryptosporidium parvum*	4,074	3,515	8	3	11	0.27

*Trichomonas vaginalis*	59,681	20,729	134	15	149	0.25

*Trypanosoma cruzi*	20,184	14,598	46	3	49	0.24

*Toxoplasma gondii*	7,793	3,350	16	0	16	0.21

*Plasmodium yoelii yoelii*	7,813	5145	16	0	16	0.20

*Encephalitozoon cuniculi*	1,918	1,122	1	2	3	0.16

Total	**15,8064**	**75,818**	**492**	**49**	**542**	

^a^Number of protein-coding genes analyzed for each genome.

^b^Number of protein-coding genes producing a phylogenetic tree in the primary screen.

^c^Number of phylogenetic trees indicative of an LGT where the query organism is separated from other eukaryotes by at least one well-supported node, or the alignment has no other closely related eukaryotes than the query taxa.

^d^Potential eukaryote-to-eukaryote gene transfers involving at least one of our query taxa.

^e^Total number of LGTs. Note that the total numbers of LGTs are not additive because 'deep, ancient, transfers' to Trypanosomatides or Apicomplexa are reported for each species; the underlying LGT is inferred to have occurred once only and in the common ancestor of the group (see Figure 4).

^f^Percentage of protein-coding genes in each genome that represent LGTs; entries ranked from the highest to the lowest value.

## Results

### Quantifying LGTs across 13 eukaryotic genomes

The majority (96%) of protein trees in the primary screen (see Additional file 1) were consistent with vertical inheritance of the sampled eukaryotic genes (or this inheritance could not be robustly rejected using our stringent criteria). In the present work, we focused on the strongest cases of LGT detected by our approach (see Additional file 1). A total of 541 protein-coding genes across 13 eukaryotic genomes were identified as candidate LGTs (Table 1 see Additional file 1) and are listed in the supplementary material (see Additional file 2; see Additional file 3; see Additional file 4). The phylogenetic trees supporting these inferences are presented as Portable Document Format (PDF) files to facilitate browsing and visual inspection (see Additional file 5; see Additional file 6; see Additional file 7). The strongest cases supported by phylogenetic trees corresponded to 357 LGTs from prokaryotic donors (see Additional file 2; see Additional file 5). Topologies consistent with eukaryote-to-eukaryote LGT following initial acquisition of a gene from a prokaryotic donor were identified for 39 genes in 26 different trees (see Additional file 3; see Additional file 6). Some of the LGTs detected may represent gene transfers from a eukaryote to a bacterium (see Additional file 4; see Additional file 7, for example, tree EB001), and in some cases, it was not possible to infer the direction of transfer with confidence. Of the trees supporting LGT, only 13 contained a broad taxonomic sampling across the 3 domains of cellular life (trees ON014, 21, 23, 31, 32, 47, 51, 53, 60, and TN110, 149, 178, 225: see Additional file 5). Most genes had a more restricted or patchy taxonomic distribution, and relationships between prokaryotes often deviated from the accepted classification, consistent with a set of complex gene histories among the prokaryotes sampled.

The number of candidate LGTs per genome ranged from 3 to 149 cases (Table 1). We identified 62 LGTs in *D. discoideum*, far higher than the 18 cases of LGT identified during the annotation of its genome using a protein domain-based analysis [21]. We also identified a higher number of candidate LGTs (see Additional file 8) than previously reported for the three kinetoplastids: 68 versus 41 for *Leishmania major*, 46 versus 21 for *Trypanosoma brucei *and 49 versus 29 for *Trypanosoma cruzi *[22]. Notably, a published comparison of three *Leishmania *spp. (*Leishmania major, Leishmania infantum *and *Leishmania donovani*) with the *T. brucei *and *T. cruzi *genomes identified only a single LGT affecting the *Leishmania *lineage [23]. By contrast, our analyses identified fewer LGTs than previously reported for six species (see Additional file 8). Some of the discrepancies for *Giardia lamblia *[24], *Toxoplasma gondii *and *Plasmodium falciparum *[25], and *Cryptosporidium parvum *[26] result from our not counting LGTs that potentially originated from the mitochondrial endosymbiont, but most differences seemed to reflect our more stringent criteria for identifying LGTs (see Additional file 1; see Additional file 8). The differences between our results and those of published studies illustrate some of the difficulties in comparing numbers of LGTs inferred by different methods, and support the use of a consistent methodology in comparative analysis. The three genomes of *D. discoideum, E. histolytica*, and *L. major *had the highest proportion of LGTs in relation to the size of their annotated proteome (Table 1, see Additional file 9). *Entamoeba *and *Dictyostelium *both actively phagocytose prokaryotes, a process that is thought to provide opportunities for LGT [27], and *Dictyostelium *contains intracellular bacteria throughout its life cycle [28]. *Leishmania *encounters prokaryotes in the gut of its insect vector. The highest number of candidate LGTs was detected for *Trichomonas vaginalis*, a species that also actively phagocytoses prokaryotes [29].

Most of the LGTs detected correspond to single-copy genes, but we identified 132 LGTs that have subsequently undergone gene duplication, and a few cases of LGTs founding large paralogous gene families (mean family size 5.9 copies; see Additional file 10). The genome of *T. vaginalis *seems to be particularly prone to repeated gene duplications producing large gene families [15]; two LGTs for hypothetical proteins (see Additional file 5, trees TN146 and TN148) have proliferated into families containing over 260 and 1200 copies, respectively (see Additional file 10).

### Functional annotation of transferred genes

**Figure 1:**
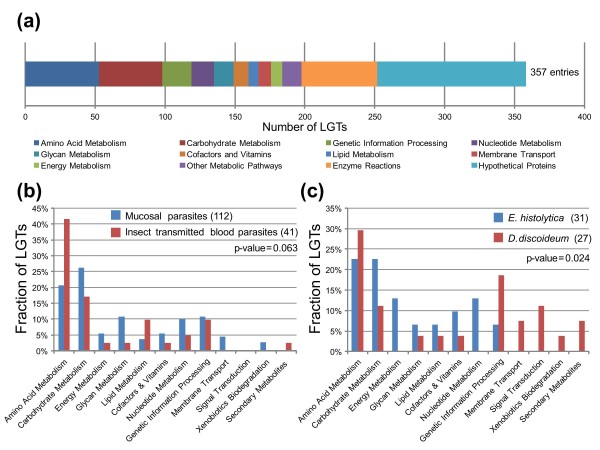
**Functional categories of identified lateral gene transfer (LGTs)**. **(a) **Distribution of functional annotation from the Kyoto Encyclopedia of Genes and Genomes (KEGG) database of LGTs supported by at least one node (357 entries; see Additional file 2; see Additional file 5). In total, 220 enzymes were identified, of which 165 (75%) could be mapped onto a KEGG pathway. The 'Other Metabolic Pathways' category includes the following KEGG pathways: 'Signal Transduction,' 'Metabolism of Secondary Metabolites,' and 'Metabolism of Terpenoids and Polyketides.' In total, 49 enzymes, labeled as 'Enzyme Reactions,' are not part of any metabolic pathway. Hypothetical proteins and poorly characterized entries are pooled in the category 'Hypothetical Proteins'. The number of entries in each functional category is based on the number of LGT events rather than genes, with an ancient LGT counted once. **(b,c) **Comparison of functional characterization of LGTs for **(b) **extracellular mucosal parasites (*Trichomonas vaginalis, Entamoeba histolytica, Giardia lamblia*) versus insect-transmitted blood parasites (*Trypanosoma brucei, Trypanosoma cruzi, Plasmodium falciparum, Plasmodium vivax, Plasmodium yoelii yoelii*) and **(c) **the parasitic amoebozoan *E. histolytica *versus the free-living amoebozoan *Dictyostelium discoideum*. Fisher's exact test was performed to test the null hypothesis that functional annotations of LGTs are distributed equally between the compared taxa. The *P*-values for the tests are indicated. The numbers of LGTs for each set of taxa are indicated between brackets.

**Figure 2:**
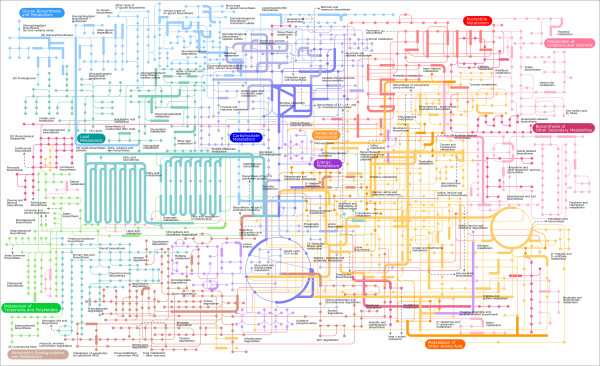
**Mapping of candidate lateral gene transfer (LGTs) onto the Kyoto Encyclopedia of Genes and Genomes (KEGG) central metabolic pathways**. Candidate LGTs (thick edges) were mapped on the KEGG central metabolic pathways using the tool iPath (version 2.0 [78]) which provides an overview of metabolic and other pathways annotated in KEGG. Nodes correspond to substrates and edges to enzymatic reactions. The 11 major metabolic pathways are color-coded (for example, light orange for amino acid metabolism). The LGTs are broadly distributed across pathways: all 11 major KEGG metabolic pathway categories are affected by LGTs. Note that the individual enzymes acyl-CoA dehydrogenase (EC:1.3.8.7) and acetyl-CoA C-acyltransferase (EC:2.3.1.16) each occur several times in the fatty-acid metabolism and elongation pathways, respectively (teal-colored pathways). The mapping of candidate LGTs onto the 'Biosynthesis of secondary metabolites map' and the 'Regulatory pathways or functional modules' is also illustrated (see Additional file 15).

Most of the LGTs we identified seem to be involved in functions that can be broadly defined as metabolism (Table 2). Enzymatic functional annotation could be inferred for 62% of the candidate LGTs (Table 2). The majority of the annotated enzymes (75%; 165 of 220 enzymes) could be mapped onto a broad range of Kyoto Encyclopedia of Genes and Genomes (KEGG) metabolic pathways (Figure 1, Figure 2). The two pathways most affected by LGTs are those involving metabolism of amino acids (15% of all detected LGTs) and sugars (13%) (see Additional file 2, Figure 1a). Comparison of the functional annotations of the pooled LGTs from the three extracellular mucosal parasites (*T. vaginalis, E. histolytica *and *G. lamblia*) with LGTs for the five insect-transmitted blood parasites (*P. falciparum, Plasmodium vivax, Plasmodium yoelli yoelli, T. brucei *and *T. cruzi*) rejected the null hypothesis (90% confidence level, *P *= 0.063) that the functional categories of LGTs were distributed similarly across the two groups (Figure 1b). The largest differences are LGTs into the mucosal parasites for enzymes mediating carbohydrate, glycan, amino acid, and lipid metabolism (Figure 1b). This is consistent with the need for mucosal parasites to be able to acquire and process these types of substrates in a highly competitive environment [30]. Similarly, comparing LGTs for the parasite *E. histolytica *and the free-living *D. discoideum *rejected the null hypothesis (95% confidence level, *P *= 0.024) for the same functional distribution of LGTs for these two amoebozoan species (Figure 1c). By contrast, there was no significant difference (*P *= 0.436) in the types of LGTs detected between the gut-dependent apicomplexans (*C. parvum *and *T. gondii) *and the three insect-transmitted *Plasmodium *spp. (see Additional file 11).

**Table 2:** Summary of the number of lateral gene transfer (LGTs) in relation to their functional annotation^a^

Description	Protein counts (LGT)				Fraction (%) from total
		
	SP^b^	TMD 1-3^c^	TMD ≥ 4^c^	Total	
Entries with annotated EC number^d^				**220^g^**	**61.6 ^g^**

Part of KEGG metabolic pathways	13	8	1	165	46.2

Involved in translation (GIP)	0	0	0	6	1.7

Reactions, enzymes, but not in a pathway	5	0	2	49	13.7

Entries without annotated EC number^e^				**137 ^g^**	**38.4 ^g^**

With some established function	1	1	8	14	3.9

Hypothetical proteins^f^					

Possess known functional conserved region	5	4	3	87	24.4

Possess domain of unknown function	3	3	2	31	8.7

No significant hit with known domains, suggesting a novel protein family	1	0	1	5	1.4

Total	28	16	17	357	

EC, Enzyme Classification; GIP, Genetic Information Processing; KEGG, Kyoto Encyclopedia of Genes and Genomes

^a^Candidate LGTs supported by at least one node (see Additional file 2; see Additional file 5).

^b^SP: Entries with an SP and without TMD are counted here.

^c^'TMDs ≥ 4' or 'TMDs 1-3' refers to the number of TMDs predicted on protein sequences. Transporters typically have at least four TMDs (TMDs ≥ 4). Proteins with one to three TMDs represent putative membrane proteins.

**^d^**EC numbers were annotated for each entry based on a significant sequence similarity to either a PRIAM enzyme profile or an enzyme annotated in KEGG (see Materials and methods).

^e^Entries without an EC annotation are classified into two major groups: entries with non-enzymatic functions and hypothetical proteins. These may be involved in cellular processes such as membrane transport or signal transduction.

^f^Hypothetical proteins were further analyzed for the presence of known conserved regions using HHsearch and InterProScan (see Additional file 2; see Additional file 3; see Additional file 4).

^g^**Total counts and fractions (%) for the two major listed categories**

A significant fraction of candidate LGTs across the 13 species (35% of total) code for hypothetical or poorly characterized proteins (Table 2; Figure 1a). Using profile-based searches, we identified protein domains in a number of these open reading frames (ORFs) (Table 2; see Additional file 3). Some cases (22 entries) are potentially membrane proteins, as they have putative transmembrane domains (TMD), and some of these (14 entries) also have additional features typical of transporters. Ten ORFs have an inferred signal peptide and are without a TMD, and hence they may be secreted (Table 2). As membrane and secreted proteins often mediate interactions with the external environment, including substrates from infected hosts, these conserved ORFs are worthy of further investigation.

**Figure 3:**
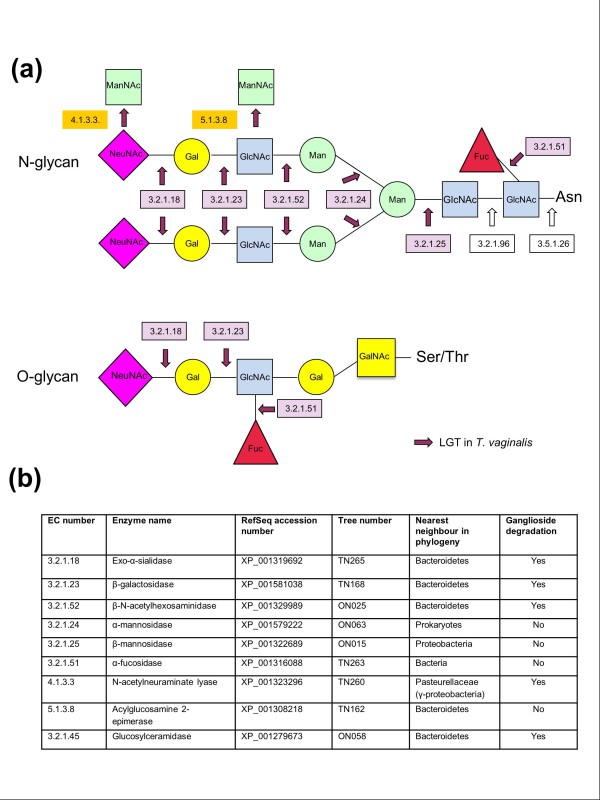
***Trichomonas vaginalis *lateral gene transfer (LGTs) that are potentially involved in glycan metabolism**. **(a) **Schematic overview of the structures of a typical N-glycan and the enzymes (EC numbers in black delineated boxes) that can degrade them, according to the KEGG pathway ec00511. A typical O-glycan (extended core 1) [79] is also illustrated, along with selected enzymes shared with N-glycan degradation. O-glycans are the major glycans found in mucins, which are degraded by *T. vaginalis*. The characteristic components of glycans are shown. NeuNAc, N-acetylneuraminic acid; Gal, galactose; GlcNAc, N-acetylglucosamine; Man, Mannose; GalNac, N-acetylgalactosamine (O-glycan specific). The activities of six glycosidases originating form LGTs, out of a total of nine required to degrade N-glycans/gangliosides, are indicated by violet arrows, with their respective EC numbers in pink boxes. Two additional enzymes (EC numbers in orange boxes), N-acetylneuraminate lyase and acylglucosamine 2-epimerase, which also correspond to LGTs, could contribute to the further metabolism of the sugars liberated during glycan degradation. **(b) **Enzyme names and activities and evidence for LGT. Enzymes shared with the pathway for gangliosides metabolism are indicated. The final step of the degradation of gangliosides by a glucosylceramidase (EC:3.2.1.45) is also an LGT into *T. vaginalis*. The structure of gangliosides and the enzymes processing them are also illustrated (see Additional file 16).

Some of the LGTs identified may have adaptive significance in the habitat occupied by the investigated species. For example, seven of the candidate LGTs affecting *T. vaginalis *provide enzymes capable of the degradation of host glycans (Figure 3). Glycans are present in the glycocalyx of epithelial cells and in the secretions of the male and female urogenital tracts, where they have important protective functions against pathogens [31,32]. *T. vaginalis *is already known to damage host tissues, and it is likely that glycan degradation contributes to that process. The carbohydrates liberated by glycan degradation could also represent a source of energy for the parasite. For example, the initial de-capping of sialic acid by sialidase (tree TN265; see Additional file 5) liberates sialic acid that can be further processed by N-acetylneuraminate lyase [33] (TN260; Figure 3; see Additional file 5) into acetylmannosamine and pyruvate. Five of these *T. vaginalis *LGTs seem to have originated from within the Bacteroidetes lineage (Figure 3). Bacteroidetes are highly abundant and nutritionally versatile members of the human mucosal microbiota; approximately 20% of their genes encode proteins that target and metabolize host and diet-derived glycans [34]. In this instance, LGT seems to have enabled *T. vaginalis *to tap into this rich metabolic capability.

Species of *Trypanosoma *have lost the urea cycle, and hence they excrete ammonia [35]. By contrast, *L. major *has most of the urea-cycle enzymes [22]; it is suggested that the excretion of neutral urea, rather than ammonia, is an adaptation by *L. major *to avoid disturbing the acid/base balance of the acidic host phagolysosomes in which it lives [36,37]. The gene for *L. major *argininosuccinate synthase, which catalyses the condensation of citrulline and aspartate to form argininosuccinate, the immediate precursor of arginine, is a candidate LGT (tree TN110, see Additional file 5). Moreover, the *L. major *arginase, shared with two other *Leishmania *species, (tree EE024, see Additional file 6) is embedded among Fungi, suggesting that a *Leishmania *spp. gained this gene from a fungus. *L. major *can grow on sucrose-containing medium [38], and its sucrose-phosphate synthase, which converts sucrose to fructose, is a candidate LGT also found in gut apicomplexans of the genus *Cryptosporidum *(tree EE017, see Additional file 6). Sucrose may represent a major nutrient source for *L. major *in the gut of the sand fly when the insect feeds on plants [39], hence, the LGT may have facilitated nutritional adaptation within the digestive tract of the sand fly vector. Homologs of ecotins, potent bacterial inhibitors of animal serine peptidases, were identified in *T. brucei, T. cruzi*, and *L. major*, and seem to have originated in their common ancestor by LGT (tree TN012, see Additional file 5). These proteins have been investigated in *L. major*, where they are thought to inhibit animal-host peptidases involved in defense mechanisms [40].

Several of the parasites have lost the pathway for oxidative phosphorylation, and therefore cannot make ATP by that route. In these species, energy is generated in other ways, including glycolysis, fermentation, and substrate-level phosphorylation [41]. Both *T. vaginalis *and *E. histolytica *can utilize amino acids as a source of energy when grown on media lacking maltose and glucose [42-44]. In *E. histolytica*, we identified several LGTs (aspartase (TN120) malic enzyme (TN183) and tryptophanase (TN224), see Additional file 5) for enzymes involved in the degradation of amino acids. Tryptophanase, which is also found as an LGT in *Trichomonas*, degrades tryptophan to ammonia, pyruvate, and indole. Pyruvate can be metabolized further to eventually contribute to ATP production by substrate-level phosphorylation in the cytosol of *Entamoeba *or the hydrogenosomes of *Trichomonas *[41].

### Dynamics of LGT among closely related parasites

**Figure 4:**
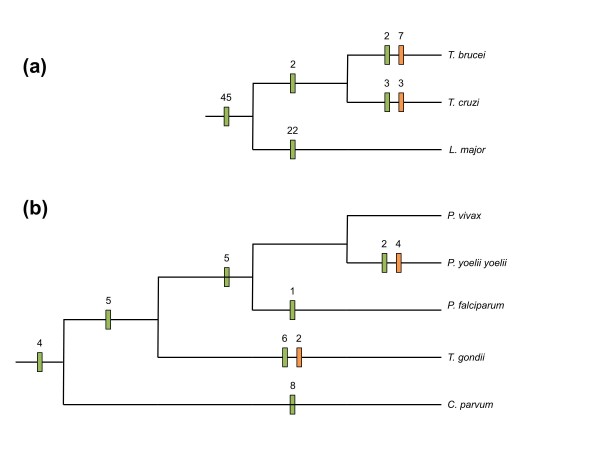
**Assessment of gains and losses of lateral gene transfer (LGTs) during parasite speciation**. Maximum parsimony was used to map candidate LGTs on the species trees for taxa among (**a) **Trypanosomatidae and **(b) **Apicomplexa. Gains and losses are indicated as green and orange bars respectively. Characters were analyzed using Dollo parsimony, so each character is allowed to have only a single gain, but may have multiple losses. It is inferred that 45 LGTs occurred (over 75 genes affected by LGT) before the divergence of the three Trypanosomatidae lineages. Fewer (7/75) LGTs are specific to the individual *Trypanosoma *spp. lineages, whereas the branch to *L. major *is inferred to have experienced 22 gains after splitting from *Trypanosoma*. The reference phylogeny for the Apicomplexa used to map the LGTs is from Wasmuth *et al*. [80]. Four LGTs were inferred to been gained by the common ancestors of all sampled apicomplexans. Losses of two and four LGTs were inferred for *Toxoplasma gondii *and *Plasmodium yoelii yoelii*, respectively. Additional LGTs were inferred across the other branches, clearly indicating the dynamic nature of LGTs during the diversification of these parasites.

We used parsimony to investigate patterns of gain and loss of LGTs among the three kinetoplastids and the five apicomplexans included in our study (Figure 4). We infer that 45 LGTs were present in the common ancestor of the three kinetoplastids (Figure 4a), a further 22 LGTs affected the *Leishmania *lineage, and two additional LGTs occurred in the common ancestor of *T. brucei *and *T. cruzi*. We also infer that *T. brucei *and *T. cruzi *each have gained additional LGTs, and both have independently lost some LGTs that were probably present in the common ancestor of the group (Figure 4a). A similar pattern of gains and losses, albeit with fewer detected LGTs, was seen for the taxonomically broader set of sampled apicomplexans (Figure 4b). In four cases, LGT seems to have occurred in the common apicomplexan ancestor, and the genes have subsequently been retained by taxonomically diverse contemporary species (Trees ON052, ON059, TN176, and TN242; see Additional file 5). We also detected examples of more ancient LGTs into the common ancestor of *E. histolytica *and *Mastigamoeba balamuthi *(tree TN145 and possibly tree EE026;l see Additional file 5, Additional file 6, respectively), and into the common ancestor of *Giardia *and *Spironucleus *(trees TN253 and EE001; see Additional file 5 and Additional file 6, respectively).

### Which groups of prokaryotes have contributed most LGTs?

**Figure 5:**
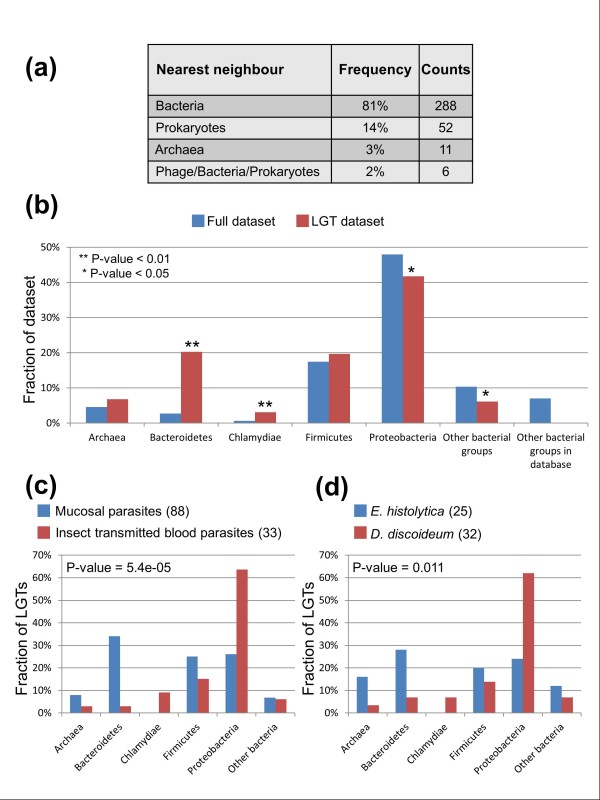
**Taxonomy of donor lineages for candidate lateral gene transfer (LGTs)**. **(a) **Donor lineages for LGTs were defined as the adjacent (as defined by Wilkinson *et al*. [81]) prokaryote to our target eukaryote(s) in trees where the relevant eukaryote(s) were separated from other eukaryotes by at least one well-supported node. Complete lists of donor lineages and the corresponding phylogenies are presented (see Additional file 13; see Additional file 5). **(b) **Taxonomic diversity of donor lineages and their contributions to LGTs. The red bars represent the proportion (%) of protein sequences identified as LGTs per donor lineage compared with the blue bars that show the proportion (%) of sequences from that lineage in the reference dataset used as the search space for the analyses. The relative significance of over-representation or under-representation established by a hypergeometric test is indicated. **(c) **Comparison of the prokaryotic lineages inferred to be donating genes to the extracellular mucosal parasites *Entamoeba histolytica, Trichomonas vaginalis*, and *Giardia lamblia *compared with the inferred donor lineages for the insect-transmitted blood parasites *Trypanosoma brucei, Trypanosoma cruzi, Plasmodium falciparum, Plasmodium vivax *and *Plasmodium yoelii yoelii*. **(d) **Comparison of the prokaryotic lineages inferred to be donating genes to the parasite *E. histolytica *and its free-living amoebozoan relative *Dictyostelium discoideum*. **(c, d) **'Other bacteria' comprise the Actinobacteria, Aquificae, Fusobacteria, Plantomycetes, Spirochaetes, or Tenericutes. Fisher's exact test was performed to test the null hypothesis that the taxonomy of the donors is distributed equally between the compared taxa. The *P*-values for the tests are indicated; they both reject the null hypothesis. The numbers of LGTs considered for each set of taxa are indicated between brackets. Complete diagrams showing all categories, including the unresolved 'Bacteria' donors and the different donors summarized as 'other bacteria,' are also presented (see Additional file 12).

The majority of LGTs are inferred to have originated from donor lineages within the bacteria, but we also identified some candidate transfers from potential archaeal donors (for example, tree TN095; Figure 5; see Additional file 5). Many of the phylogenies were not sufficiently resolved to identify specific candidate donor lineages but those that did favored (in decreasing importance) members of the Proteobacteria, Bacteroidetes, and Firmicutes, which together represent 87% of well-supported candidate donor taxa (Figure 5b;, see Additional file 2; see Additional file 12; see Additional file 13). Further analysis of these data strongly suggests that there is a bias towards transfers from prokaryotes sharing similar habitats to the recipient parasites (Figure 5; see Additional file 2; see Additional file 11). Contrasting the pooled LGTs of the three extracellular mucosal parasites (*Trichomonas, Entamoeba*, and *Giardia*) to those of the five insect-transmitted blood parasites (*Plasmodium *spp. and *Trypanosoma *spp.) strongly rejects the null hypothesis (*P *< 0.001) that the taxonomic distribution of the major prokaryotic donors are the same for the two sets of parasites (Figure 5c; see Additional files 11; see Additional file 12). For example, trees suggesting a donor lineage among the Bacteroidetes are clearly more frequent for the mucosal parasites (Figure 5c), consistent with the donor and recipient sharing similar habitats. Bacteroidetes are particularly abundant in the digestive tracts of humans and other vertebrates [45-47], but can also be present in the female urogenital tract during bacterial vaginosis [48]. A similar bias towards LGTs from Bacteroidetes emerged when comparing data between the gut parasite *E. histolytica *and the soil dwelling *D. discoideum *(Figure 5d). The range of donors of LGTs to *E. histolytica *was very similar to that identified for *G. lamblia *(see Additional file 12) suggesting that there is a significant link between habitat and LGT origins for these two extracellular mucosal parasites. Less striking similarities were also found between the taxonomic origins of LGTs to *Entamoeba *and to the gut-dependent apicomplexans *C. parvum *and *T. gondii *(see Additional file 12). Some of the candidate eukaryote-to-bacteria LGT also seem to have involved microorganisms that share the same habitat. One tree (EB002; see Additional file 9) in particular suggests a complex pattern of LGT between the ancestors of diverse mucosal bacteria and microbial eukaryotes, including *Bacteroides fragilis *(Bacteroidetes), *Treponema denticola *(Spirochaetes), *T. vaginalis *(Parabasalia) and *E. histolytica *(Amoebozoa). A number of candidate LGTs were also identified among microbial eukaryotes living on mucosal surfaces (for example, trees EE001-3, -11, -24, -26; see Additional file 6). We detected five LGTs that implicate Chlamydiae as donors to the kinetoplastids (trees TN025, TN027, TN118; see Additional file 5) or *D. discoideum *(trees TN185 and TN200; see Additional file 5). The former suggests LGT between intracellular pathogens (Chlamydiae and kinetoplastids) sharing an animal host, whereas the two LGTs to *Dictyostelium *may reflect gene sharing between *Chlamydiae *and their soil-inhabiting eukaryotic hosts [49].

## Discussion

To identify recent LGTs using a common methodology, we analyzed the published genomes of 13 microbial eukaryotes representing a broad range of eukaryotic lineages with diverse life cycles and habitats. The fraction of identified LGTs varied from 0.16% to 0.97% (average 0.38%) of protein-coding genes per genome, with an average contribution across the 13 genomes of 1 LGT per 357 protein-coding genes (Table 1; see Additional file 9). These proportions are relatively modest compared with the values reported for prokaryotes [50]. However, the number of identified LGTs may be dependent on the method of analysis and the criteria used to identify LGTs; it has already been shown that there is poor agreement between the number and identity of LGTs identified using different methods [3,19]. We also found some discrepancies between the published data for the genomes we analyzed and our own results. The 357 LGTs reported here are based upon a very conservative interpretation of phylogenetic trees: we did not count poorly supported topologies even if they depicted the type of patchy taxonomic sampling that is consistent with LGT. For example, we did not include the bacterial-like ATP transporters shared by Microsporidia and bacterial obligate intracellular pathogens in our list, despite it being likely that LGT has occurred between prokaryotes and eukaryotes for these genes [17]. Some of the lack of resolution in our trees may reflect limited sampling combined with the well-known difficulties associated with phylogenetic analysis of the divergent molecular sequences of parasites [10]. In addition, we did not investigate LGTs involving segments or domains of proteins [51], although this is already thought to affect proteins with complex domain organization such as surface proteins [52,53]. Thus, it is likely that our estimates provide only a conservative lower bound for the real number of LGTs that have affected the genomic content of the microbial eukaryotes investigated.

The patterns for LGTs affecting closely related kinetoplastids and apicomplexans demonstrates that, as for prokaryotes [8], LGT is a dynamic process involving gain and loss over relatively short genetic distances. Those LGTs that have been retained after parasite diversification are likely to be functionally important for the parasites. LGT can be a powerful source of innovation by mediating rapid phenotypic changes, in contrast to the slower changes mediated by point mutations of existing genes [1,3,6]. In addition, approximately 35% of LGTs correspond to poorly characterized proteins, suggesting that there are important gaps in our knowledge of the function of genes shared between parasites and host-associated prokaryotes.

Although some of the LGTs we detected seem to involve replacement of a previously existing host gene by a prokaryotic homolog (for example, argininosuccinate synthase (tree TN110) and thiol-peroxidase (tree TN225); see Additional file 5), other LGTs seem to have brought new capacities to the recipient eukaryote. For example, an LGT at the base of the kinetoplastids for a gene encoding the peptidase inhibitor ecotin, a known virulence-associated gene in *Yersinia *spp. [54], may provide kinetoplastids with resistance to some mammalian and insect host peptidases [40]. *T. vaginalis *provides a particularly compelling example, where LGTs seem to have greatly facilitated the ability of the organism to degrade the complex glycans that are present in the host mucosal secretions and host cell membranes [55-57]. Nine of the relevant *T. vaginalis *enzymes are the product of gene transfers, providing a striking example of an almost complete pathway that has been gained by LGTs from various prokaryotic donors. The activity of two of the *Trichomonas *enzymes has already been reported: β-galactosidase contributes to the degradation of mucus [55] and α-mannosidase is known to be secreted during *in vitro *growth [56]. The activity of a third enzyme of the pathway, N-acetyl-β-D-hexosaminidase, correlates with levels of erythrocyte lysis *in vitro *[57].

The taxonomy of some of the LGT donors was sufficiently well resolved to identify them as belonging to particular taxonomic groups and this allowed us to compare patterns of gene flow affecting specific parasites. Thus, several of the investigated parasites share a habitat with the complex and abundant prokaryotic community that lives in the gut of vertebrates [45] and on other mucosa [47], and this community is known to exchange genes frequently [58]. Our data show that important extracellular mucosal parasites, including *E. histolytica, G. lamblia *and *T. vaginalis*, which between them are responsible for over 500 million new infections annually [20], are sampling from the same pool of genes. In these species, ecology and lifestyle seem to strongly influence the opportunities for transfer and the origins of transferred genes. Thus, there is demonstrable enrichment in the genomes of *E. histolytica, G. lamblia *and *T. vaginalis *for LGTs from donors related to Bacteroidetes and Firmicutes, the dominant lineages in the gut microbiota of humans and other vertebrates [45]. Comparing LGTs detected for the gut parasite *E. histolytica *and its free-living relative *D. discoideum *also supports this distinction.

Beyond its importance for understanding how the mucosal microbial community, which is vital for human health [30], evolves and functions, the widespread sharing of genes has implications for the development of transferred genes as potential drug targets for parasites [16,59]. Thus, genes that are shared widely between parasites and indigenous prokaryotes may need to be avoided as drug targets in order to prevent the adverse affects, already seen with some antibiotics [30], on beneficial members of the human microbiota. In contrast to the mucosal parasites, the apicomplexans and the kinetoplastids were enriched in LGTs from proteobacterial donors. *Plasmodium *mosquito vectors were recently shown to have a gut microbiota that is highly enriched in proteobacteria [60]. However, comparing the taxonomy of the donor lineages for LGTs affecting the Apicomplexa and the Kintetoplastids identified no significant differences, although the tsetse fly vector for *T. brucei *harbors a bacterial flora enriched in Firmicutes [61] compared with proteobacteria [62].

Our analyses complement existing studies, which show that EGTs from the prokaryotic endosymbionts [11,12,41] that gave rise to plastids and mitochondria have had a major influence on eukaryotic metabolism, particularly but not exclusively [41] on energy metabolism. The genes that have been assigned to plastid or mitochondrial ancestry are typically those for which eukaryotes form a monophyletic group rooted in either the cyanobacteria or α-proteobacteria. In our own analyses, we did not include these contributions to eukaryotic genomes in our list of LGTs, focusing instead on transferred prokaryotic genes with much more limited taxonomic distribution among eukaryotes and hence more likely to be of recent origin. These types of LGTs were easier to detect using our approach than more ancient events, for which the limitations of data and phylogenetic models can combine to prevent robust inferences. Nevertheless, we did detect some strongly supported deeper transfers (for example, trees TN145, TN242, and TN253; see Additional file 5), and there are also reports of LGTs of algal origin into the base of the animal radiation [63]. There are, of course, no obvious reasons to suppose that barriers to LGT between prokaryotes and eukaryotes were any greater in the distant, as opposed to the more recent past.

## Conclusions

Our data strongly suggest that LGT from diverse prokaryotes has had a major effect on the origins of genes that make up metabolic pathways in contemporary eukaryotes. Thus, although the number of LGTs we detected for individual eukaryotic genomes was typically less than 1% of the genes analyzed, the significance of LGTs for eukaryotic metabolism can be better appreciated when the LGTs from all 13 genomes are shown together on a single metabolic map (Figure 2). All 11 categories of KEGG metabolic pathways have been affected by LGTs, with 44% of the 162 individual pathways containing at least one candidate LGT; gene transfer has left a strong imprint on eukaryotic metabolism (Figure 2). It has previously been suggested that genes for metabolic enzyme (operational genes) can be replaced by LGT more easily than genes for processes such as transcription and translation (informational genes) [8,27]. If we make the (albeit simplistic) assumption (see Materials and methods) that all operational genes have similar rates of LGT, and use the average number of LGTs per genome from the current study, then sampling an additional 800 taxonomically diverse eukaryotic genomes would ensure (with 95% confidence) that every operational gene was affected by LGT in at least one genome. Thus, although many metabolic pathways are conserved across the eukaryote tree of life, our results suggest that the individual genes making up those pathways in different lineages will often have very different origins.

## Materials and methods

### A primary screen for LGTs

Protein sequences from 13 completed microbial eukaryote genomes were collected from public databases (Table 1). In total, 158,064 sequences 100 amino acids or more in length were analyzed using a phylogenomic approach with SPyPhy [64]. For each sequence, a similarity search was performed using BLASTP [65] against UniProt. To avoid possible exclusion of relevant sequences, the maximum number of alignments reported in the BLASTP output was increased to 10,000. If three or more sequences related to the query sequence showing at least 25% identity over at least 50% of the length of the corresponding query sequence were found, alignments were performed using ClustalW [66]. Owing to computational limitations, the number of sequences in the alignment was limited to 100, and multiple sequences from the same organism were pruned to a single sequence when they showed 80% or greater sequence identity to each other. To ensure that wherever possible all Domains (Bacteria, Archaea, and Eukaryotes) were sampled in our alignments, we screened the BLASTP output for sequences from any domain not represented in the top 100 sequences, and added these to the alignment. GBLOCKS [67] was used to remove poorly aligned positions (allowed gap positions: half; minimum length of a block: 2; maximum number of contiguous non-conserved positions: 20). Protein p-distance neighbor-joining analyses with 100 bootstrap replicates were performed using PAUP* [68].

Based on our previous experience in identifying LGTs in the genome of *E. histolytica*, we designed an automated primary screen (that identified all the published LGTs for this parasite [14]) to allow faster processing of the large number of proteins to be analyzed. The automated screening procedure was based on e-value ratios and homology-derived secondary structure of proteins (HSSP)-value scores [69] to detect potential LGTs among the 75,818 alignments produced by SPyPhy. A sequence was considered a possible prokaryote-to eukaryote-LGT if it passed the initial criteria (described above), if the adjacent taxon in the protein p-distance neighbor-joining tree was from a prokaryote, if the ratio of the e-value of the top prokaryote versus the next best eukaryotic hit e-value was 1.00E-05 or less (prokaryote e-value/eukaryote e-value ≤ 1.00E-5), and if the highest value of the distance to the HSSP threshold curve, *n*, was 5.0 or more (this conservative minimum HSSP was chosen in order to avoid selection of false-positive sequences [70]). The HSSP distance is a measure for sequence similarity accounting for pairwise sequence identity and alignment length, where *n *describes the distance in percentage points from a standard curve derived from database entries of known homologous proteins. In some cases (for example, in candidate surface proteins) the identity with the query protein seemed to be due entirely to repeats, for example, as in the leucine-rich repeats of TvBspA [52]. These proteins were difficult to align with confidence, and were not included in our phylogenetic analyses. The primary screen yielded a total of 2,946 candidate LGTs.

### Phylogenetic analysis of candidate LGTs

Candidate LGTs passing the initial screen were subjected to phylogenetic analysis using maximum likelihood distances and Bayesian inference. We first used automated MrBayes [71] analyses to find the 'best' tree under a rates across sites model (using the function 'invgamma' with free α and fraction of invariant sites) and the Whelan And Goldman (WAG) matrix. The analyses were run for 600,000 generations, starting with a random tree, four heated chains run in parallel, and a sample frequency of 100. A 'burn in' corresponding to one-third of the total number of generations was used, and the consensus tree was calculated with branch length and posterior probabilities for the retained trees (two-thirds of the generations). Because Bayesian posterior probabilities have been criticized [72], we also used bootstrapping with maximum likelihood distances-minimum evolution distance analyses to provide an additional indication of support for relationships. Each data set was bootstrapped (100 replicates) and used to make distance matrices under the same evolutionary model as in the Bayesian analysis, using custom software in P4 [73]. Trees were estimated from the distance matrices using FastME [74] and a bootstrap consensus tree calculated using P4. The bootstrap proportions were then mapped on the MrBayes consensus trees. All cases where the tree topology showed one or more eukaryotic sequences clustered with prokaryote sequences, separated from other eukaryotes by at least one well-supported (posterior probabilities (PP) ≥ 0.95, bootstrap proportion (BP) ≥ 0.7) node, were considered as a candidate LGT. All branches with weak support values of PP less than 0.95 or BP less than 0.7 [75] were collapsed into polytomies to simplify the identification of the most strongly supported candidate LGTs.

### Mapping LGTs onto metabolic pathways

LGTs were mapped onto the KEGG [76] metabolic pathways (accessed 19 November 2010) using Enzyme Classification (EC) numbers with the tool KEGG Mapper. EC numbers were inferred by structural scores, applying a minimum threshold HSSP score of 5.0 for BLASTP hits annotated with EC numbers. This was complemented with the following analyses. BLASTP was used to perform sequence similarity searches for each candidate LGT entry against all known enzyme sequences in the KEGG database (containing 1,110,595 sequences). The BLASTP e-value was set at ≤ 1.00E-5. An LGT query sequence was assigned the EC number of the best BLASTP hit only if that hit had 31% or greater identity to the query sequence, providing a conservative annotation [77]. To investigate EC annotation for more divergent sequences, we used HMMER (version 3) to perform hidden Markov model (HMM) profile searches for PRIAM enzyme profiles (August 2010 release). A query sequence was assigned an EC number resulting from the HMMER search only if the best 1-domain e-value was 1.00E-5 or less.

### Statistical analyses

Fisher's exact test was used to test the hypothesis that functional annotation of LGTs or the taxonomy of the candidate donor lineage in well-resolved phylogenies was distributed equally between sets of contrasted taxa.

Over-representation or under-representation of LGTs from a given taxonomic group (Figure 5b) was determined using a hypergeometric test. The test is based on the probability of observing *x *number of protein sequences from a given taxonomic group as LGTs, given a process of sampling without replacement from the whole dataset used to search for homologs. The probability of observing *x *number of a particular donor lineage is described as:

$P\left(X=k\right)=\frac{\left(\begin{array}{c} m\\ k \end{array}\right)\left(\begin{array}{c} N-m\\ n-k \end{array}\right)}{\left(\begin{array}{c} N\\ n \end{array}\right)},$

where N (1,646,205) represents the total number of prokaryotic protein sequences in the whole dataset used as the search space for this study, m (163) is the total number of sequences from identified prokaryotic donor lineages defined as the adjacent (as defined by Wilkinson *et al*. [81]) prokaryote to our target eukaryote(s) in trees where the relevant eukaryote(s) were separated from other eukaryotes by at least one well-supported node, n is the number of protein sequences from a particular taxonomic group (for example, Bacteroidetes) within the whole dataset, and k is the subset from m for a given taxonomic prokaryotic donor lineage (for example, Bacteroidetes).

### How many genomes need to be sampled for LGT to have affected every enzyme in the core KEGG pathways for eukaryotes?

Based on the genome-coding capacities, KEGG annotations and number of identified LGTs for our target taxa (see Additional file 14) we can estimate the number of similar genomes that would need to be analyzed in order to ensure that 1) every KEGG enzyme can be found in the pooled set of genes from the genomes, and 2) every KEGG enzyme can be found in the subset of genes that have been laterally transferred. The calculation of these estimates is based on the following set of naive assumptions. We assume that for a given KEGG enzyme there is a fixed probability, p_obs_, that it can be found in a randomly selected genome, and that the presence or absence of the enzyme is independent between genomes. Under this assumption, the number of genomes that must be sampled in order for the enzyme to be observed in the collection of pooled genes has a geometric distribution with parameter p_obs_, and the probability that the enzyme is observed in k genomes is 1-(1-p_obs_)^k^. We additionally assumed that the probability p_obs _is the same for all KEGG enzymes and that presence or absence of an enzyme in a genome is independent of all other KEGG enzymes. Using the empirical value p_obs _= 328/1,806 (see Additional file 14) gives an estimate of k = 52 genomes that will be required in order to obtain 95% probability of observing all 1,806 eukaryotic KEGG enzymes in the pooled collection of genes.

To calculate the number of genomes required to similarly find every KEGG enzyme in the subset of laterally transferred genes, p_obs _is replaced with the corresponding empirical value from the table; there are on average 38 genes per genome identified as having been laterally transferred, of which 62% are KEGG enzymes. The empirical probability that a given KEGG enzyme will be found in the set of laterally transferred genes within a particular randomly selected genome is therefore 62% × 38/1,806 = 0.013. Repeating the calculation above gives an estimate of k = 800 genomes to obtain 95% probability of observing all eukaryotic KEGG enzymes within the subset of laterally transferred genes. Given the naivety of our assumptions and level of approximation, these estimates are crude, and are really only a rough indication of the number of genomes required.

## Abbreviations

BP: bootstrap proportion; EC: Enzyme Classification; EGT: Endosymbiotic gene transfer; HMM: Hidden Markov model; HSSP: Homology-derived secondary structure of proteins; KEGG: Kyoto Encyclopedia of Genes and Genomes; LGT: Lateral gene transfer; ORF: Open reading frame; PDF: Portable Document Format; PP: posterior probabilities; TMD: Transmembrane domain; WAG: Whelan And Goldman.

## Competing interests

The authors declare that they have no competing interests.

## Authors' contributions

CA, TME, and RPH conceived of the project and wrote the manuscript. CA performed the bulk of the bioinformatic analyses, complemented by analyses from PF, TSP, and SN. All authors edited the manuscript. All authors read and approved the final manuscript.

## Supplementary Material

Additional file 1 **Flowchart of methodology**. Figure depicting the flowchart of the methodology used to identify lateral gene transfers (LGTs) including the number of genes retained at each step of the analysis for the 13 analyzed genomes. Click here for file

Additional file 2 **Prokaryote-to-eukaryote lateral gene transfers (LGTs)**. Table with the accession numbers and annotations of proteins for prokaryote-to-eukaryote LGTs supported in the Bayesian consensus trees by at least one node. For legends to the table, see Additional file 17; for illustrations of the trees, see Additional file 5. Click here for file

Additional file 3 **Eukaryote-to-eukaryote lateral gene transfers (LGTs)**. Table with the accession numbers and annotations of proteins for eukaryote-to-eukaryote LGTs supported in the Bayesian consensus trees by at least one node. For legends to the table, see Additional file 17; for illustrations of the trees, see Additional file 6. Click here for file

Additional file 4 **Eukaryote-to-prokaryote lateral gene transfers (LGTs)**. Table with the accession numbers and annotations of proteins for eukaryote-to-prokaryote LGTs supported in the Bayesian consensus trees by at least one node. For legends to the table, see Additional file 17; for illustrations of the trees, see Additional file 7. Click here for file

Additional file 5 **Phylogenetic trees supporting prokaryote-to-eukaryote lateral gene transfers (LGTs)**. Figure illustrating the phylogenies for the candidate LGTs from prokaryotes to eukaryotes supported by at least one well-supported node in the phylogenetic tree. Click here for file

Additional file 6 **Phylogenetic trees supporting eukaryote-to-eukaryote lateral gene transfers (LGTs)**. Figure illustrating the phylogenetic trees for the candidate LGTs from eukaryotes to prokaryotes supported by at least one well-supported node in the phylogenetic tree. Click here for file

Additional file 7 **Phylogenetic trees supporting eukaryote-to-prokaryote lateral gene transfers (LGTs)**. Figure illustrating the phylogenetic trees for the candidate LGTs from eukaryotes to prokaryotes supported by at least one well-supported node in the phylogenetic tree. Click here for file

Additional file 8 **Comparison of lateral gene transfers (LGTs) detected in this and previous studies for individual taxa**. Number of LGTs from this study contrasted with previously published LGTs cases for the target taxa. For all legends to the table, see Additional file 17. Click here for file

Additional file 9 **Relative numbers of lateral gene transfers (LGTs) to proteome size**. Figure and table of the relationship between the number of identified LGTs and the number of annotated genes in each respective genome. Click here for file

Additional file 10 **Paralog counts for lateral gene transfers (LGTs)**. Estimated number of paralogs for each LGT listed in Supplementary Tables 1 to 3. For all legends to the table, see Additional file 17. Click here for file

Additional file 11 **Taxonomy of donors of lateral gene transfers (LGTs)**. Table with the counts for specific comparisons between selected target taxa for the functional categories of LGTs or the taxonomy of LGT donor lineages supported by a least one node. For all legends to the table, see Additional file 17. Click here for file

Additional file 12 **Taxonomy of donor lineages for candidate lateral gene transfer (LGT) between specific subsets of protists, with extended versions and additional comparison**. Diagrams presenting comparisons of donor lineages for candidate LGTs between different groups of protists. Click here for file

Additional file 13 **Taxonomic counts of donors of lateral gene transfer (LGTs)**. Table with the counts of the taxonomy of the potential prokaryotic donor lineages for LGTs supported by at least one node (defined as the adjacent lineage to a given target taxa in trees (for list, see Additional file 2; for illustrations, see Additional file 5). For all legends to the table, see Additional file 17. Click here for file

Additional file 14 **Kyoto Encyclopedia of Genes and Genomes (KEGG) annotations**. Table with the number of proteins annotated in KEGG for all the 13 target genomes analyzed in this study and the corresponding diversity of KEGG entries for annotated enzymes. For all legends to the table, see Additional file 17. Click here for file

Additional file 15 **Lateral gene transfer (LGTs) affecting KEGG secondary metabolites and regulatory pathways**. Figure illustrating the LGTs mapped onto the KEGG secondary metabolite and regulatory pathways. Click here for file

Additional file 16 **Kyoto Encyclopedia of Genes and Genomes (KEGG) pathway for the degradation of gangliosides**. Figure illustrating a schematic overview of the KEGG pathway for the degradation of gangliosides. Click here for file

Additional file 17 **Table legends**. Legends for tables in additional files 2, 3, 4, 8, 10, 11, 13, and 14. Click here for file
