# Supplementary material for: Patterns of prokaryotic lateral gene transfers affecting parasitic microbial eukaryotes
Source: Genome Biol. 2013 Feb 25;14(2):R19. doi: 10.1186/gb-2013-14-2-r19 (PMC4053834; doi:10.1186/gb-2013-14-2-r19)
Supplement: Additional file 1 — Flowchart of methodology. Figure depicting the flowchart of the methodology used to identify lateral gene transfers (LGTs) including the number of genes retained at each step of the analysis for the 13 analyzed genomes. [file gb-2013-14-2-r19-S1.PDF]

Additional file 1.

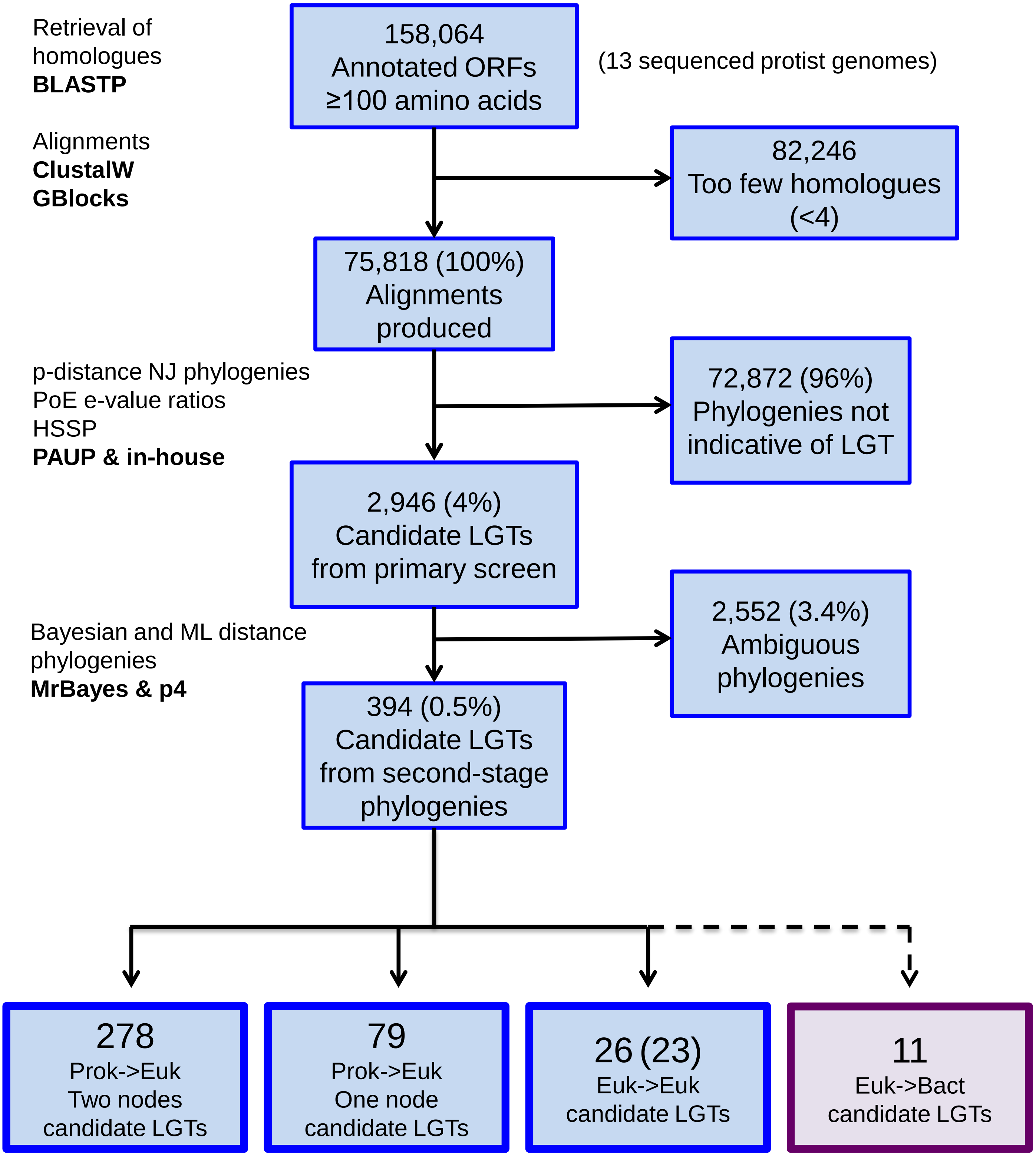

Additional File 1. Flowchart of the methodology used to identify LGT including the resulting number of genes at each step. Protein sequences from 13 microbial eukaryote genomes were collected from public databases (Table 1). In total 158064 sequences longer or equal to 100 amino acids were analysed. Homology searches were performed using BLASTP against a database consisting of TREMBL and Swissprot databases and orthologues were extracted. Alignments were performed using ClustalW for all genes attracting three or more homologues resulting in 75818 alignments. The GBLOCKS program was used to remove poorly aligned and diverged positions. Neighbor-joining analysis with 100 bootstrap replicates was performed using PAUP* and p-distances. An automated screening procedure based upon the analysis of eukaryote-prokaryote relationships in the bootstrapped p-distance trees, e-value ratios between the top (for the query sequence) prokaryote and eukaryote blast hits and homology-derived secondary structure of proteins (HSSP-value) yielded 2946 candidate LGTs. After manual inspection and further refinement of alignments, candidate LGTs were investigated by more detailed second-stage phylogenetic analyses using a more realistic model in MrBayes and P4. Each data set was bootstrapped (100 replicates) and used to make distance matrices under the same evolutionary model as in the Bayesian analysis, using custom (P4) software. All trees where a candidate LGT was clustered with prokaryote sequences and separated from other non-self (by self we mean the same species or member of the same monophyletic group – e.g all kinetoplastids) eukaryotes by at least one well supported node (bootstrap value ≥ 70%, posterior probability ≥ 0.95) were scored as LGTs. During these analyses 26 eukaryote-to-eukaryote (Euk->Euk) LGTs were also inferred, as were 11 eukaryote-to-prokaryote (Euk->Pro) LGTs. In 23 of the Euk->Euk LGTs the two eukaryotes involved nest among prokaryotes consistent with an initial LGT from a prokaryote to an ancestor of one of the eukaryotes followed by transfer to another eukaryote.
