# Supplementary material for: Patterns of prokaryotic lateral gene transfers affecting parasitic microbial eukaryotes
Source: Genome Biol. 2013 Feb 25;14(2):R19. doi: 10.1186/gb-2013-14-2-r19 (PMC4053834; doi:10.1186/gb-2013-14-2-r19)

Additional File 5. Candidate LGT from prokaryotes to eukaryotes

Each tree is a Bayesian consensus tree, based on an alignment of homologues from one or more of the target taxa studied in this survey, where LGT from prokaryotes to eukaryotes is the most parsimonious explanation for the resulting tree topology. The key for the symbols used to differentiate the taxonomy of the analyses sequences and provide taxonomic contextualization for the candidate LGTs is indicated just after the list of trees. A parallel phylogenetic analyses was performed with pairwise maximum likelihood distances calculated with the same model used for the Bayesian analysis and bootstrap values estimated and mapped onto the Bayesian consensus tree. Only well-supported branches are indicated (posterior probabilities $\geq$ 0.95, bootstrap $\geq$ 0.7) on the trees with all other branches being collapsed into polytomies. The LGTs supported by at least one node (trees numbered ONXXX) or at least two nodes (trees numbered TNXXX) separating the target taxa from other eukaryotes are illustrated. Datasets where the target taxa are the only eukaryote (or with closely related taxa, the latter suggesting deep LGT prior speciation events) among prokaryotic sequences are also labeled as TNXXX. The accession number and annotations of the sequences from the target taxa (boxed in the trees) are indicated. A PHOBIUS analysis investigated the potential presence of a signal peptide (SP) and transmembrane domains (TMD).

Entries in the table of contents below are clickable hyperlinks to the tree figures.

21 September, 2012

Contents

Taxa group color key . . . 2
ON001 . . . 3
ON002 . . . 4
ON003 . . . 5
ON004 . . . 6
ON005 . . . 7
ON006 . . . 8
ON007 . . . 9
ON008 . . . 10
ON009 . . . 11
ON010 . . . 12
ON011 . . . 13
ON012 . . . 14
ON013 . . . 15
ON014 . . . 16
ON015 . . . 17
ON016 . . . 18
ON017 . . . 19
ON018 . . . 20
ON019 . . . 21
ON020 . . . 22
ON021 . . . 23
ON022 . . . 24
ON023 . . . 25
ON024 . . . 26
ON025 . . . 27
ON026 . . . 28
ON027 . . . 29
ON028 . . . 30
ON029 . . . 31
ON030 . . . 32
ON031 . . . 33
ON032 . . . 34
ON033 . . . 35
ON034 . . . 36
ON035 . . . 37
ON036 . . . 38
ON037 . . . 39
ON038 . . . 40
ON039 . . . 41
ON040 . . . 42
ON041 . . . 43
ON042 . . . 44
ON043 . . . 45
ON044 . . . 46
ON045 . . . 47
ON046 . . . 48
ON047 . . . 49
ON048 . . . 50
ON049 . . . 51
ON050 . . . 52
ON051 . . . 53
ON052 . . . 54
ON053 . . . 55
ON054 . . . 56
ON055 . . . 57
ON056 . . . 58
ON057 . . . 59
ON058 . . . 60
ON059 . . . 61
ON060 . . . 62
ON061 . . . 63
ON062 . . . 64
ON063 . . . 65
TN001 . . . 66
TN002 . . . 67
TN003 . . . 68
TN004 . . . 69
TN005 . . . 70
TN006 . . . 71
TN007 . . . 72
TN008 . . . 73
TN009 . . . 74
TN010 . . . 75
TN011 . . . 76
TN012 . . . 77
TN013 . . . 78
TN014 . . . 79
TN015 . . . 80
TN016 . . . 81
TN017 . . . 82
TN018 . . . 83
TN019 . . . 84
TN020 . . . 85
TN021 . . . 86
TN022 . . . 87
TN023 . . . 88
TN024 . . . 89
TN025 . . . 90
TN026 . . . 91
TN027 . . . 92
TN028 . . . 93
TN029 . . . 94
TN030 . . . 95
TN031 . . . 96
TN032 . . . 97
TN033 . . . 98
TN034 . . . 99
TN035 . . . 100
TN036 . . . 101
TN037 . . . 102
TN038 . . . 103
TN039 . . . 104
TN040 . . . 105
TN041 . . . 106
TN042 . . . 107
TN043 . . . 108
TN044 . . . 109
TN045 . . . 110
TN046 . . . 111
TN047 . . . 112
TN048 . . . 113
TN049 . . . 114
TN050 . . . 115
TN051 . . . 116
TN052 . . . 117
TN053 . . . 118
TN054 . . . 119
TN055 . . . 120
TN056 . . . 121
TN057 . . . 122
TN058 . . . 123
TN059 . . . 124
TN060 . . . 125
TN061 . . . 126
TN062 . . . 127
TN063 . . . 128
TN064 . . . 129
TN065 . . . 130
TN066 . . . 131
TN067 . . . 132
TN068 . . . 133
TN069 . . . 134
TN070 . . . 135
TN071 . . . 136
TN072 . . . 137
TN073 . . . 138
TN074 . . . 139
TN075 . . . 140
TN076 . . . 141
TN077 . . . 142
TN078 . . . 143
TN079 . . . 144
TN080 . . . 145
TN081 . . . 146
TN082 . . . 147
TN083 . . . 148
TN084 . . . 149
TN085 . . . 150
TN086 . . . 151
TN087 . . . 152
TN088 . . . 153
TN089 . . . 154
TN090 . . . 155
TN091 . . . 156
TN092 . . . 157
TN093 . . . 158
TN094 . . . 159
TN095 . . . 160
TN096 . . . 161
TN097 . . . 162
TN098 . . . 163
TN099 . . . 164
TN100 . . . 165
TN101 . . . 166
TN102 . . . 167
TN103 . . . 168
TN104 . . . 169
TN105 . . . 170
TN106 . . . 171
TN107 . . . 172
TN108 . . . 173
TN109 . . . 174
TN110 . . . 175
TN111 . . . 176
TN112 . . . 177
TN113 . . . 178
TN115 . . . 179
TN116 . . . 180
TN117 . . . 181
TN118 . . . 182
TN119 . . . 183
TN120 . . . 184
TN121 . . . 185
TN122 . . . 186
TN123 . . . 187
TN124 . . . 188
TN125 . . . 189
TN126 . . . 190
TN127 . . . 191
TN128 . . . 192
TN129 . . . 193
TN131 . . . 194
TN132 . . . 195
TN133 . . . 196
TN134 . . . 197
TN135 . . . 198
TN136 . . . 199
TN137 . . . 200
TN138 . . . 201
TN139 . . . 202
TN140 . . . 203
TN141 . . . 204
TN142 . . . 205
TN143 . . . 206
TN144 . . . 207
TN145 . . . 208
TN146 . . . 209
TN147 . . . 210
TN148 . . . 211
TN149 . . . 212
TN150 . . . 213
TN151 . . . 214
TN152 . . . 215
TN153 . . . 216
TN154 . . . 217
TN155 . . . 218
TN156 . . . 219
TN157 . . . 220
TN158 . . . 221
TN159 . . . 222
TN160 . . . 223
TN161 . . . 224
TN162 . . . 225
TN163 . . . 226
TN164 . . . 227
TN165 . . . 228
TN166 . . . 229
TN167 . . . 230
TN168 . . . 231
TN169 . . . 232
TN170 . . . 233
TN171 . . . 234
TN172 . . . 235
TN173 . . . 236
TN174 . . . 237
TN175 . . . 238
TN176 . . . 239
TN177 . . . 240
TN178 . . . 241
TN179 . . . 242
TN180 . . . 243
TN181 . . . 244
TN182 . . . 245
TN183 . . . 246
TN184 . . . 247
TN185 . . . 248
TN186 . . . 249
TN187 . . . 250
TN188 . . . 251
TN189 . . . 252
TN190 . . . 253
TN191 . . . 254
TN192 . . . 255
TN193 . . . 256
TN194 . . . 257
TN195 . . . 258
TN196 . . . 259
TN197 . . . 260
TN198 . . . 261
TN199 . . . 262
TN200 . . . 263
TN201 . . . 264
TN202 . . . 265
TN203 . . . 266
TN204 . . . 267
TN205 . . . 268
TN206 . . . 269
TN207 . . . 270
TN208 . . . 271
TN209 . . . 272
TN210 . . . 273
TN211 . . . 274
TN212 . . . 275
TN213 . . . 276
TN214 . . . 277
TN215 . . . 278
TN216 . . . 279
TN217 . . . 280
TN218 . . . 281
TN219 . . . 282
TN220 . . . 283
TN221 . . . 284
TN222 . . . 285
TN224 . . . 286
TN225 . . . 287
TN226 . . . 288
TN227 . . . 289
TN228 . . . 290
TN229 . . . 291
TN230 . . . 292
TN231 . . . 293
TN232 . . . 294
TN233 . . . 295
TN234 . . . 296
TN235 . . . 297
TN236 . . . 298
TN237 . . . 299
TN238 . . . 300
TN239 . . . 301
TN240 . . . 302
TN241 . . . 303
TN242 . . . 304
TN243 . . . 305
TN244 . . . 306
TN245 . . . 307
TN246 . . . 308
TN247 . . . 309
TN248 . . . 310
TN249 . . . 311
TN250 . . . 312
TN251 . . . 313
TN252 . . . 314
TN253 . . . 315
TN254 . . . 316
TN255 . . . 317
TN256 . . . 318
TN257 . . . 319
TN258 . . . 320
TN259 . . . 321
TN260 . . . 322
TN261 . . . 323
TN262 . . . 324
TN263 . . . 325
TN264 . . . 326
TN265 . . . 327

Taxa group color key

Bacteria		
	ACT	Actinobacteria
	AQU	Aquificae
	BC	Bacteroidetes/Chlorobi group
	CAL	Caldiserica
	C-V	Chlamydiae/Verrucomicrobia group
	CHL	Chloroflexi
	CHR	Chrysiogenetes
	CYA	Cyanobacteria
	DEF	Deferribacteres
	D-T	Deinococcus-Thermus
	DIC	Dictyoglomi
	ELU	Elusimicrobia
	F-A	Fibrobacteres/Acidobacteria group
	FIR	Firmicutes
	FUS	Fusobacteria
	GEM	Gemmatimonadetes
	NIT	Nitrospirae
	PLA	Planctomycetes
	PRO	Proteobacteria
	SPI	Spirochaetes
	SYN	Synergistetes
	TEN	Tenericutes
	THED	Thermodesulfobacteria
	THET	Thermotogae
	UB	unclassified Bacteria
	EB	environmental samples
Archaea		
	CRE	Crenarchaeota
	EUR	Euryarchaeota
	KOR	Korarchaeota
	NAN	Nanoarchaeota
	THA	Thaumarchaeota
	UA	unclassified Archaea
	EA	environmental samples
Eukaryotes		
	AMO	Amoebozoa
	OPI	Opisthokonta
	NUC	Nucleariidae
	CEN	Centroheliozoa
	APU	Apusozoa
	KAT	Katablepharidophyta
	RHI	Rhizaria
	HET	Heterolobosea
	FOR	Fornicata
	MAL	Malawimonadidae
	EUG	Euglenozoa
	JOK	Jakobida
	OXY	Oxymonadida
	PAR	Parabasalia
	HAP	Haptophyceae
	CRY	Cryptophyta
	ALV	Alveolata
	STR	stramenopiles
	RHO	Rhodophyta
	GLA	Glaucocystophyceae
	VIR	Viridiplantae
	UE	unclassified eukaryotes
	EE	environmental samples
Superkingdoms		
	●	Bacteria
	▲	Archaea
	■	Eukaryotes
	❊	Viruses

ON001

```
                Candy accession: Q4Q871_LEIMA
               RefSeq accession: XP_001684477.1
              Uniprot accession: Q4Q871_LEIMA
                       Comments: LGT - KINETOPLASTIDS ONE NODE
               Species affected: LM,TC, FUNGI
          Adjacent taxa in tree: Proteobacteria - Campylobacter
EC annotation - (Blast/Profile): na
                     PHOBIUS SP: 0
                    PHOBIUS TMD: 0
              RefSeq annotation: X-pro, dipeptidyl-peptidase,serine
                                 peptidase, Clan SC, family S15
        Name of enzyme/protein: NonD putative hydrolase
        KEGG PATHWAY - level 1: Reaction
        KEGG PATHWAY - level 2: Reaction
```

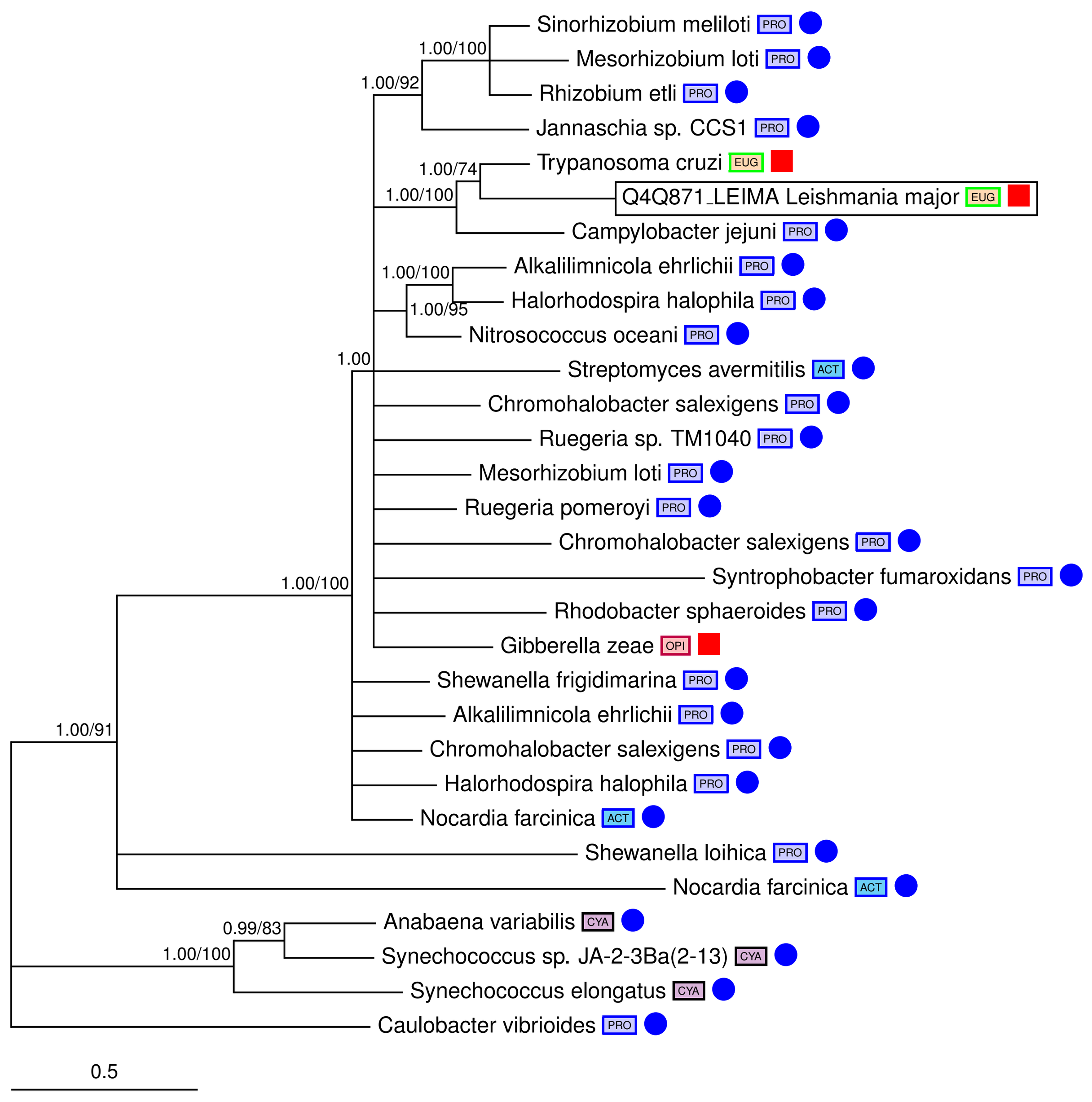

ON002

Candy accession: Q38BC8_9TRYP
RefSeq accession: XP_822720.1
Uniprot accession: Q38BC8_9TRYP
Comments: LGT - KINETOPLASTIDS ONE NODE + HUMAN SEQUENCE
Species affected: LM,TB,TC
Adjacent taxa in tree: Bacteria
EC annotation - (Blast/Profile): EC:3.1.26.4
PHOBIUS SP: 0
PHOBIUS TMD: 0
RefSeq annotation: ribonuclease HII
Name of enzyme/protein: Ribonuclease H
KEGG PATHWAY - level 1: Reaction
KEGG PATHWAY - level 2: Reaction

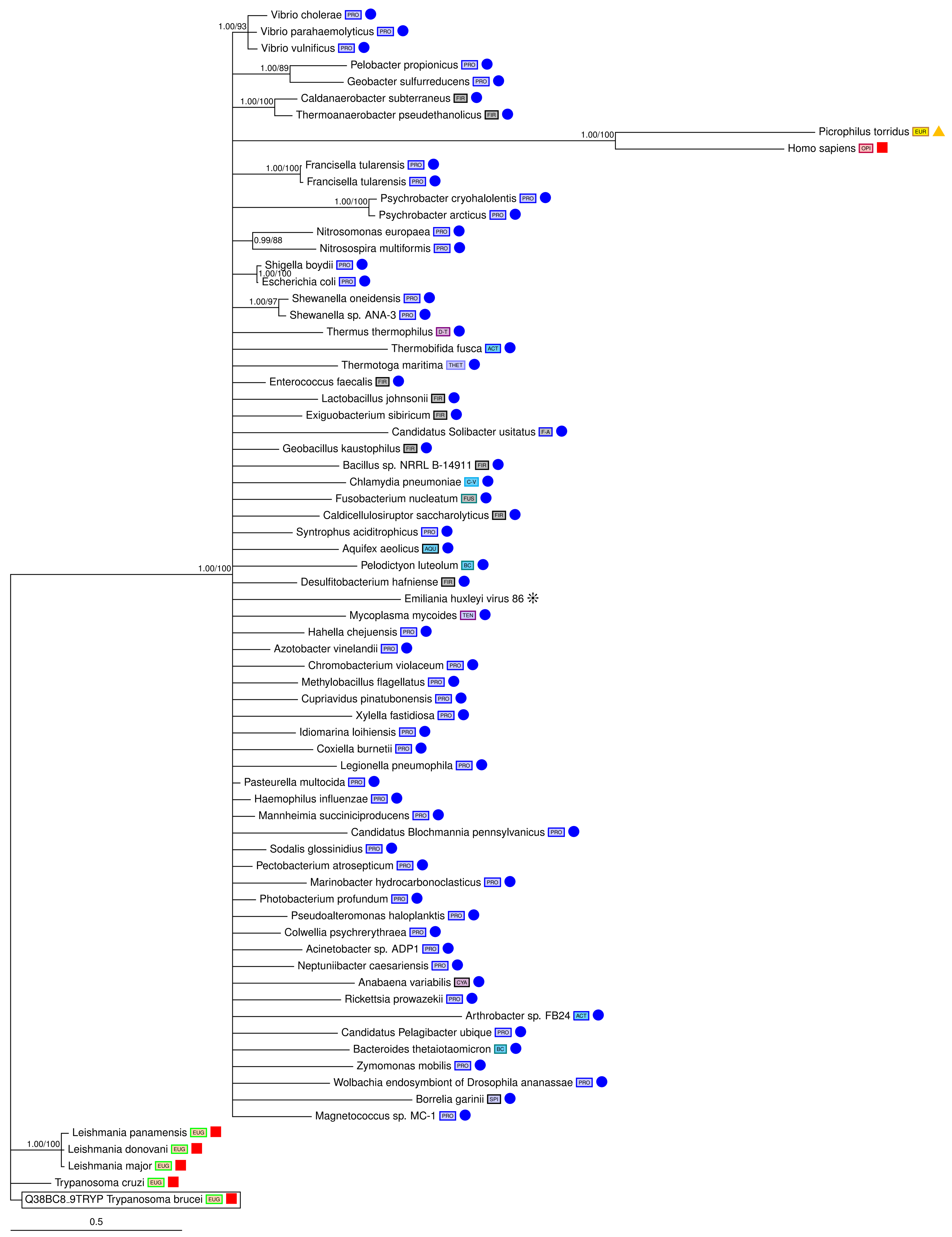

ON003

```
            Candy accession: Q50U03_ENTHI
           RefSeq accession: XP_650453.1
          Uniprot accession: C4M3S0_ENTHI
                   Comments: LGT - EH ONE NODE + ONE FUNGI
           Species affected: EH
     Adjacent taxa in tree: Bacteroidetes/Chlorobi
EC annotation - (Blast/Profile): EC:2.7.7.22
                PHOBIUS SP: 0
               PHOBIUS TMD: 0
          RefSeq annotation: mannose-1-phosphate guanylyltransferase
     Name of enzyme/protein: mannose-1-phosphate guanylyltransferase
                             (GDP)
     KEGG PATHWAY - level 1: Carbohydrate Metabolism
     KEGG PATHWAY - level 2: Fructose and mannose metabolism, Amino
                             sugar and nucleotide sugar metabolism
```

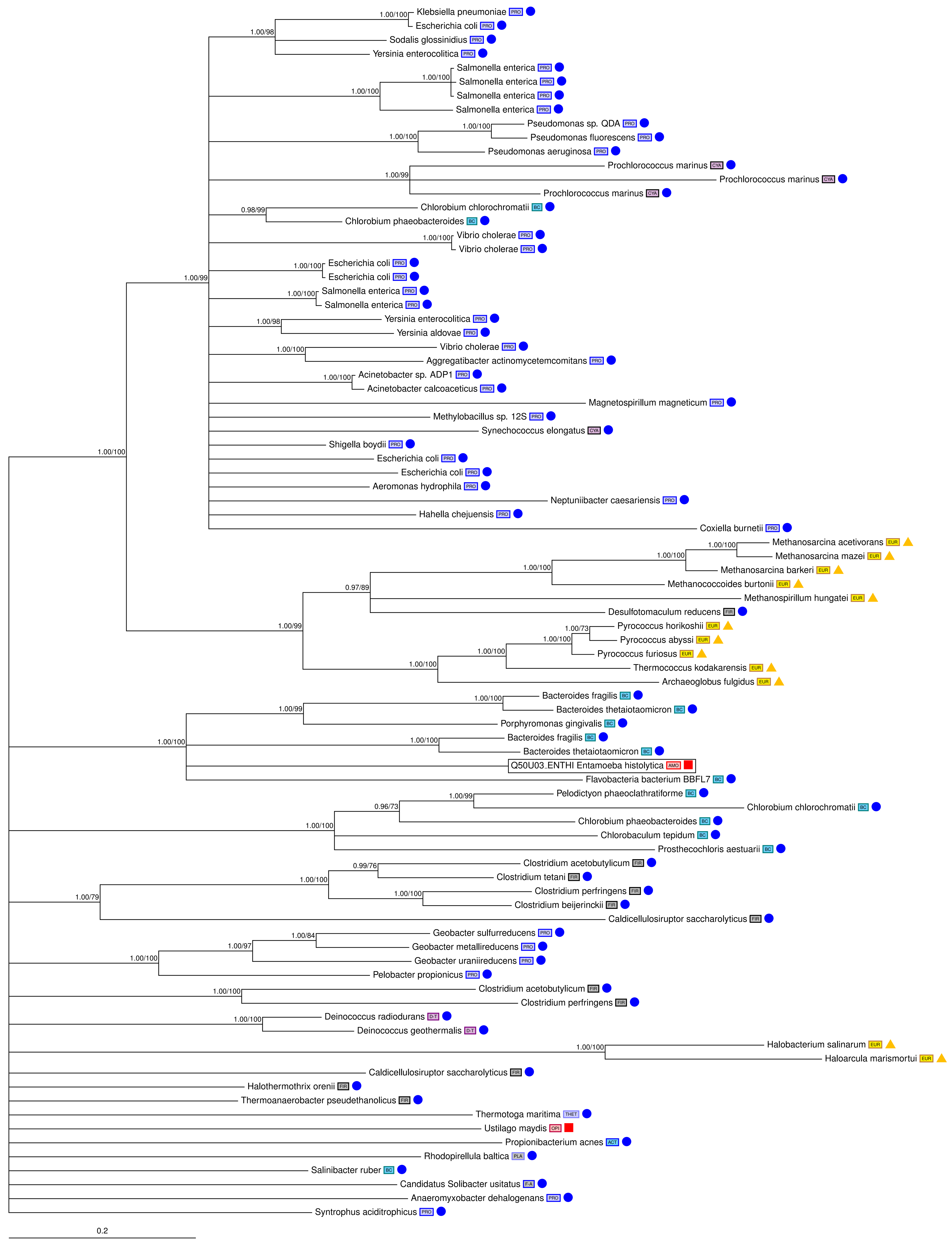

ON004

```
                Candy accession: Q50YM7_ENTHI
               RefSeq accession: XP_652087.1
              Uniprot accession: C4M2I2_ENTHI
                       Comments: LGT - EH ONE NODE
               Species affected: EH
          Adjacent taxa in tree: Prokaryote
EC annotation - (Blast/Profile): EC:2.7.1.4
                     PHOBIUS SP: 0
                    PHOBIUS TMD: 0
              RefSeq annotation: fructokinase
         Name of enzyme/protein: Fructokinase
         KEGG PATHWAY - level 1: Carbohydrate Metabolism
         KEGG PATHWAY - level 2: Fructose and mannose metabolism, Starch
                                 and sucrose metabolism, Amino sugar and
                                 nucleotide sugar metabolism
```

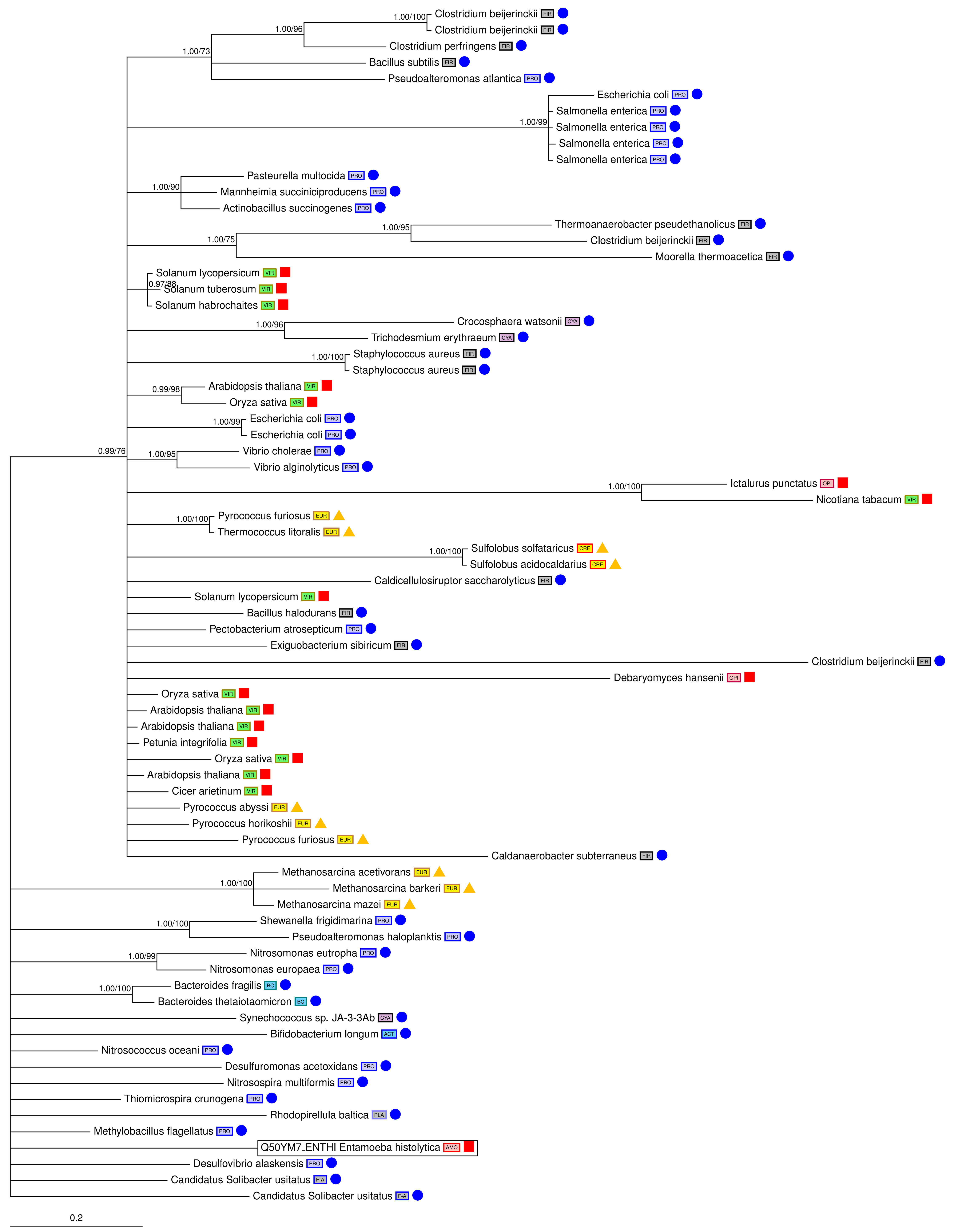

ON005

```
              Candy accession: Q51F93_ENTHI
              RefSeq accession: XP_656980.1
             Uniprot accession: C4LSR8_ENTHI
                      Comments: LGT - EH ONE NODE - LGT INTO PLASMODIUM?
              Species affected: EH,PF,PY
           Adjacent taxa in tree: Firmicutes
EC annotation - (Blast/Profile): EC:2.6.1.42
                    PHOBIUS SP: 0
                   PHOBIUS TMD: 0
             RefSeq annotation: branched-chain amino acid
                                aminotransferase
          Name of enzyme/protein: Branched-chain amino acid
                                aminotransferase
         KEGG PATHWAY - level 1: Amino Acid Metabolism, Metabolism of
                                Cofactors and Vitamins
         KEGG PATHWAY - level 2: Valine, leucine and isoleucine
                                degradation, Valine, leucine and
                                isoleucine biosynthesis, Pantothenate
                                and CoA biosynthesis
```

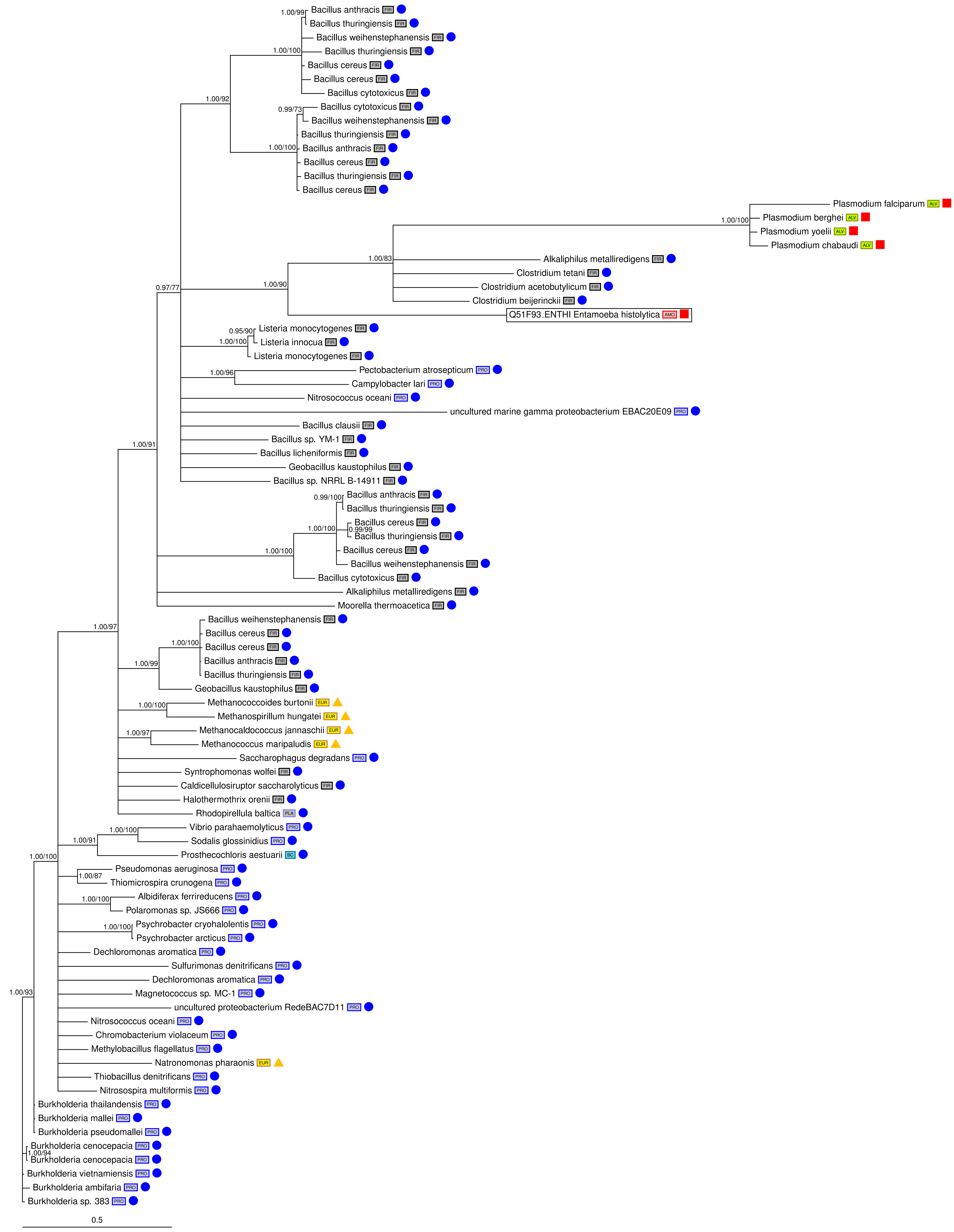

ON006

```
                Candy accession: Q51GY0_ENTHI
               RefSeq accession: XP_657511.1
              Uniprot accession: C4LSZ9_ENTHI
                       Comments: LGT - EH ONE NODE
               Species affected: EH
          Adjacent taxa in tree: Bacteria
EC annotation - (Blast/Profile): EC:2.7.6.2
                     PHOBIUS SP: 0
                    PHOBIUS TMD: 0
              RefSeq annotation: thiamine pyrophosphokinase
         Name of enzyme/protein: Thiamine pyrophosphokinase
         KEGG PATHWAY - level 1: Metabolism of Cofactors and Vitamins
         KEGG PATHWAY - level 2: Thiamine metabolism
```

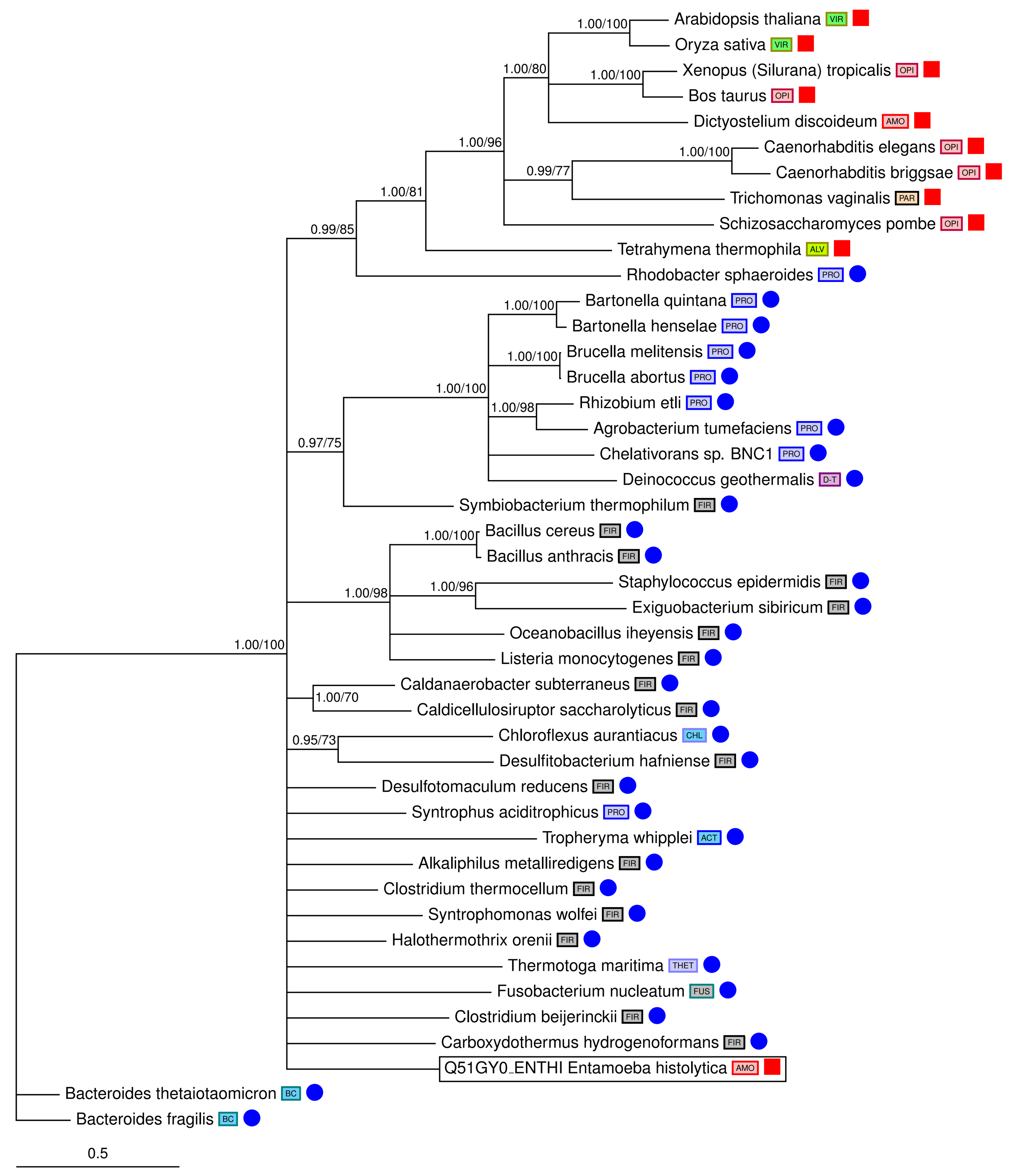

ON007

```
              Candy accession: Q54I80_DICDI
             RefSeq accession: XP_636499.1
            Uniprot accession: PANC_DICDI
                     Comments: LGT - DD ONE NODE
             Species affected: DD
       Adjacent taxa in tree: Proteobacteria - Francisella
EC annotation - (Blast/Profile): EC:6.3.2.1
                   PHOBIUS SP: 0
                  PHOBIUS TMD: 0
            RefSeq annotation: pantoate-beta-alanine ligase
      Name of enzyme/protein: Pantoate-beta-alanine ligase
      KEGG PATHWAY - level 1: Metabolism of Other Amino Acids,
                              Metabolism of Cofactors and Vitamins
      KEGG PATHWAY - level 2: beta-Alanine metabolism, Pantothenate
                              and CoA biosynthesis
```

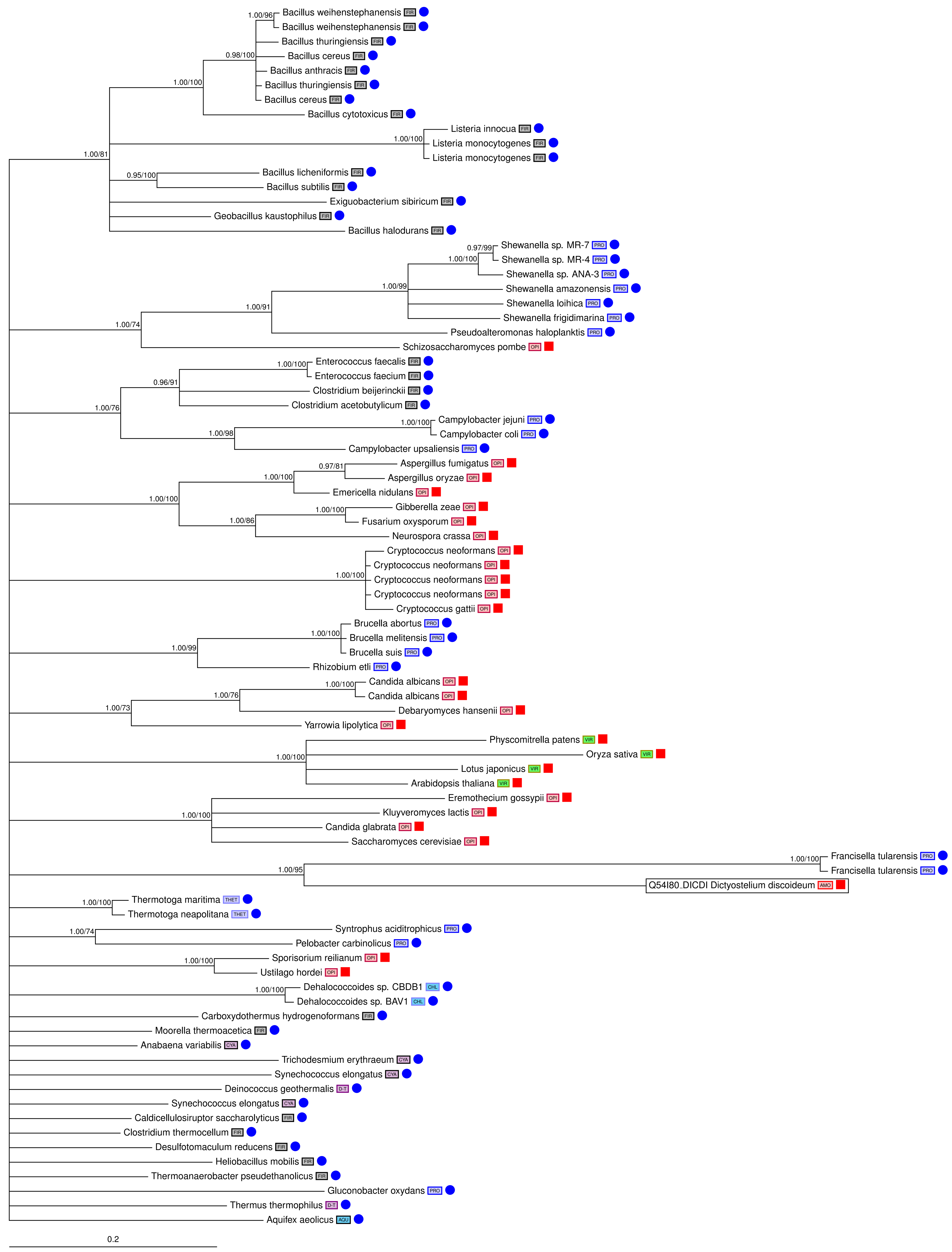

ON008

Candy accession: Q54R90_DICDI
RefSeq accession: XP_639157.1
Uniprot accession: Q54R90_DICDI
Comments: LGT - DD ONE NODE
Species affected: DD
Adjacent taxa in tree: Protobacteria
EC annotation - (Blast/Profile): EC:5.3.1.23
PHOBIUS SP: 0
PHOBIUS TMD: 0
RefSeq annotation: translation initiation factor eIF-2B alpha subunit
Name of enzyme/protein: S-methyl-5-thioribose-1-phosphate isomerase
KEGG PATHWAY - level 1: Amino Acid Metabolism
KEGG PATHWAY - level 2: Cysteine and methionine metabolism

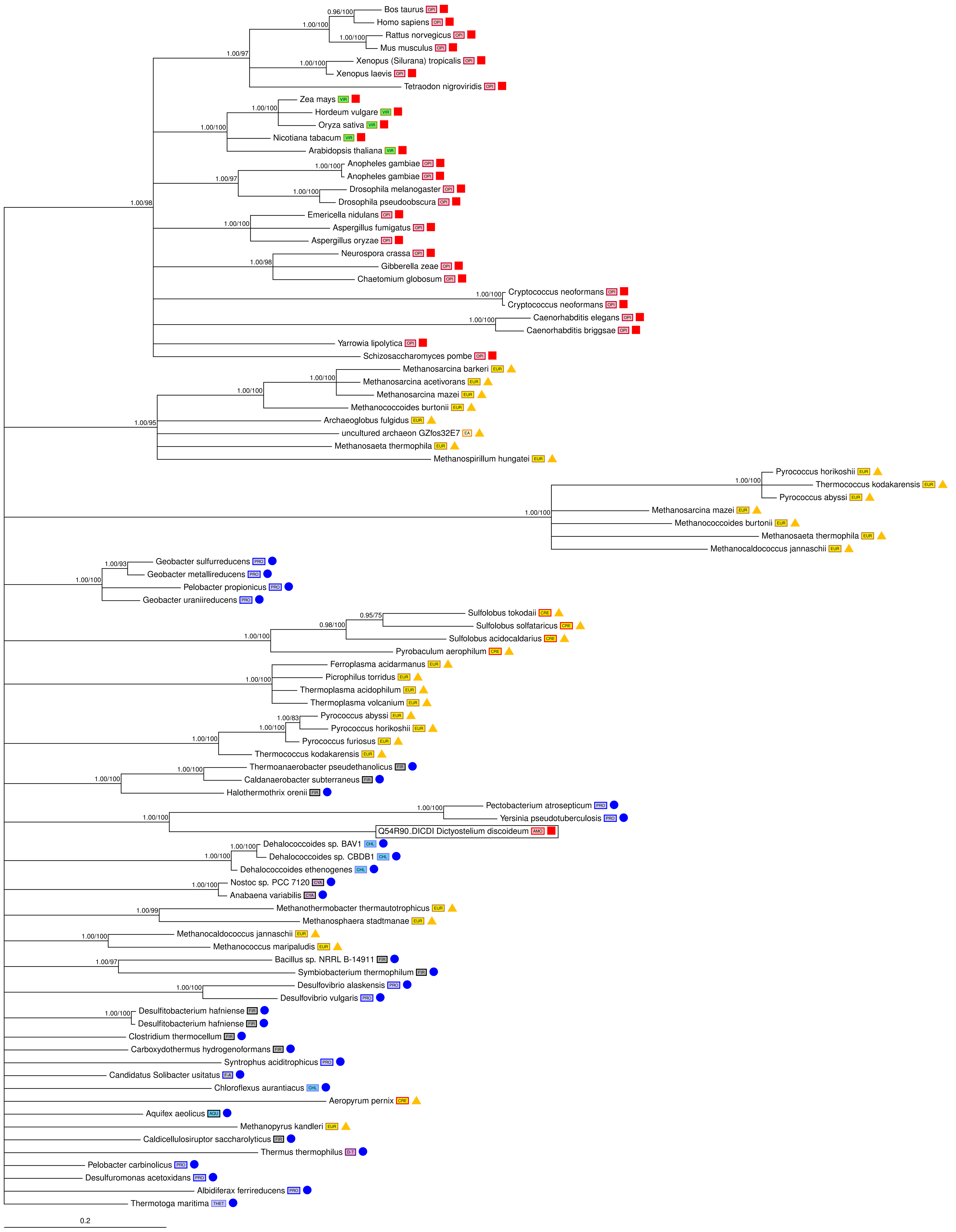

ON009

```
               Candy accession: Q4X9V4_PLACH
              RefSeq accession: XP_735375.1
             Uniprot accession: Q4X9V4_PLACH
                      Comments: LGT - EH ONE NODE
              Species affected: PC,PB
         Adjacent taxa in tree: Proteobacteria
EC annotation - (Blast/Profile): EC:3.2.2.1
                    PHOBIUS SP: 0
                   PHOBIUS TMD: 0
             RefSeq annotation: A/G-specific adenine glycosylase
         Name of enzyme/protein: purine nucleosidase
        KEGG PATHWAY - level 1: Nucleotide Metabolism
        KEGG PATHWAY - level 2: Purine metabolism, Nicotinate and
                                nicotinamide metabolism
```

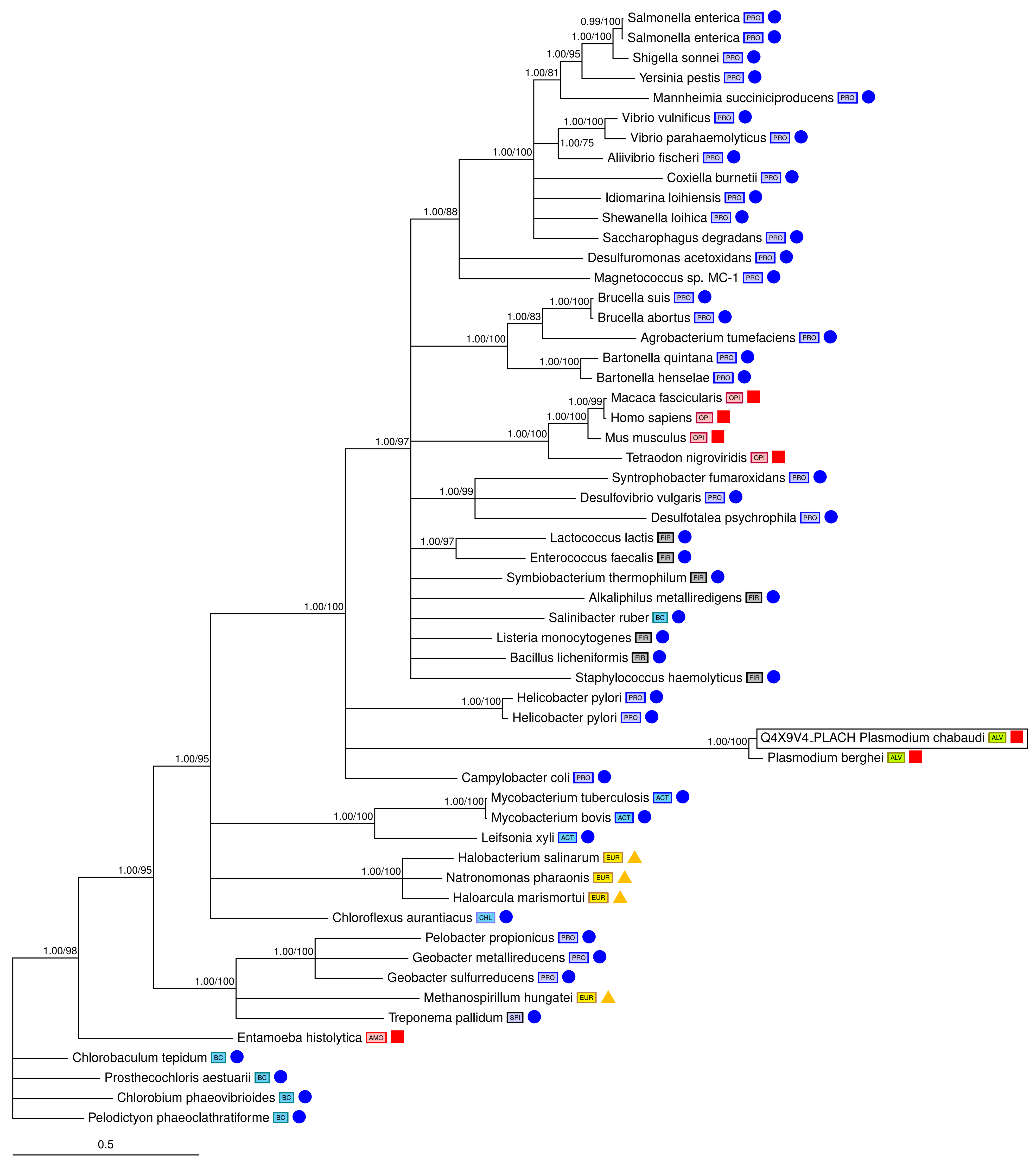

ON010

```
                  Candy accession: TV81529550
                 RefSeq accession: XP_001583442.1
                Uniprot accession: A2DAF3_TRIVA
                         Comments: LGT - TV ONE NODE + FUNGI PATHOGEN
                                   Cryptococcus neoformans
                 Species affected: TV
            Adjacent taxa in tree: Prokaryotes
EC annotation - (Blast/Profile): EC:3.5.1.81
                      PHOBIUS SP: 0
                     PHOBIUS TMD: 0
               RefSeq annotation: hypothetical protein
           Name of enzyme/protein: N-acyl-D-amino-acid deacylase
          KEGG PATHWAY - level 1: Reaction
          KEGG PATHWAY - level 2: Reaction
```

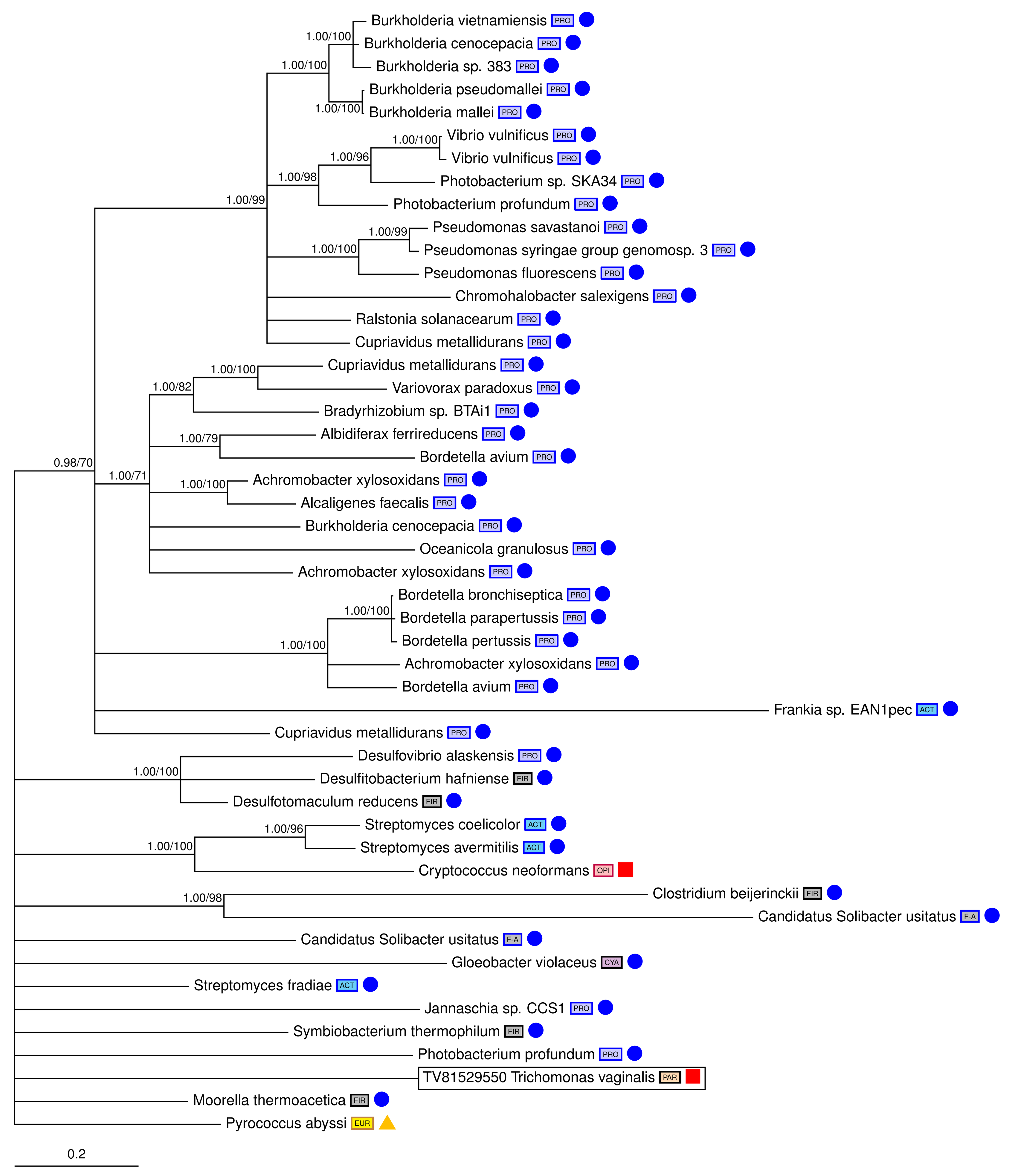

ON011

```
            Candy accession: TV85889496
           RefSeq accession: XP_001582328.1
          Uniprot accession: A2DEC4_TRIVA
                   Comments: LGT - TV ONE NODE
           Species affected: TV
      Adjacent taxa in tree: Bacteroides
EC annotation - (Blast/Profile): EC:3.1.1.31
                 PHOBIUS SP: 0
                PHOBIUS TMD: 0
          RefSeq annotation: hypothetical protein
   Name of enzyme/protein: 6-phosphogluconolactonase
   KEGG PATHWAY - level 1: Carbohydrate Metabolism
   KEGG PATHWAY - level 2: Pentose phosphate pathway
```

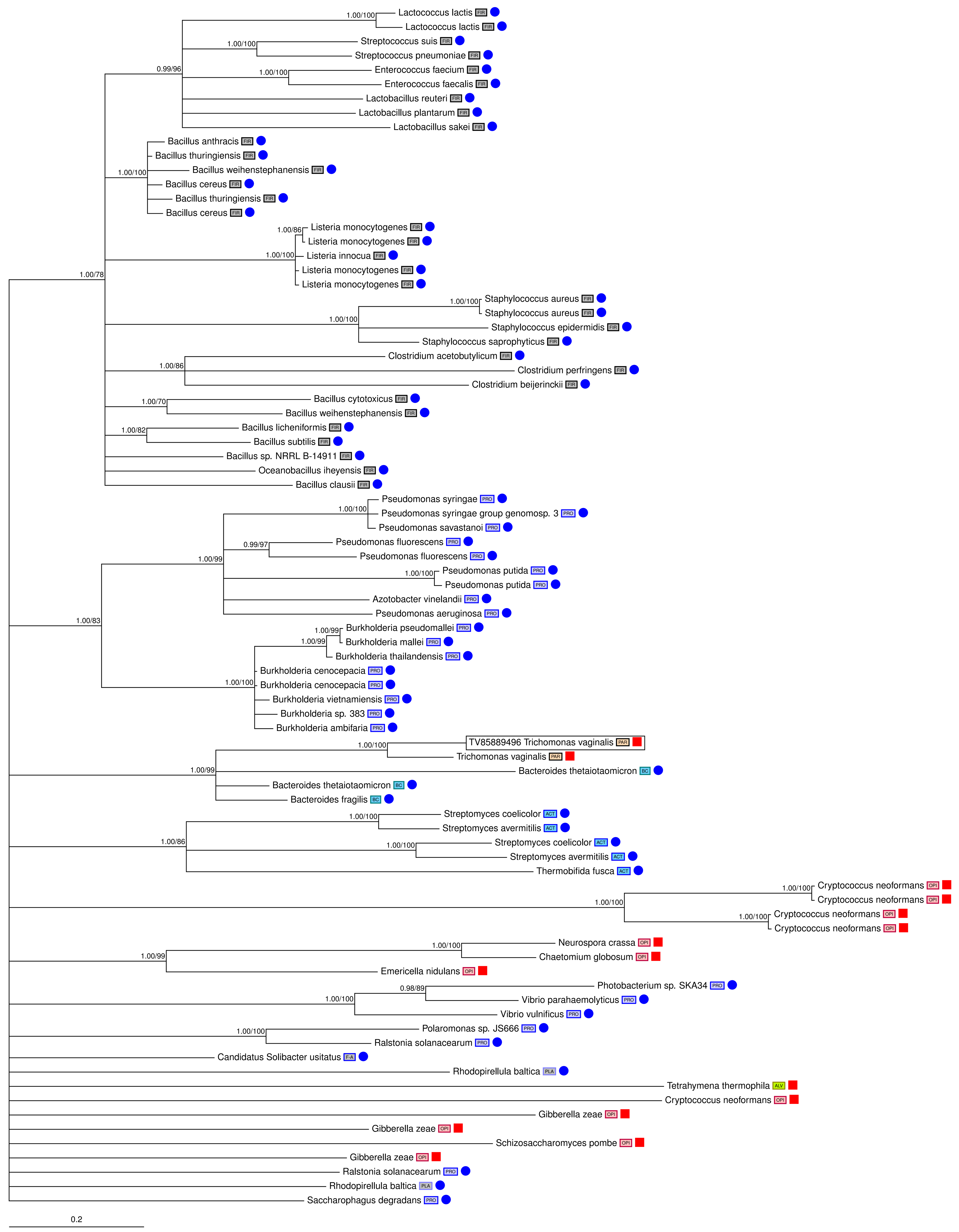

ON012

```
                Candy accession: TV88719020
               RefSeq accession: XP_001300045.1
              Uniprot accession: A2G6V8_TRIVA
                       Comments: LGT - TV ONE NODE
               Species affected: TV
           Adjacent taxa in tree: Bacteria
EC annotation - (Blast/Profile): EC:3.4.13.21
                      PHOBIUS SP: 0
                     PHOBIUS TMD: 0
               RefSeq annotation: Clan PC(S), family S51, peptidase E-like
                                  serine peptidase
          Name of enzyme/protein: dipeptidase E
          KEGG PATHWAY - level 1: Reaction
          KEGG PATHWAY - level 2: Reaction
```

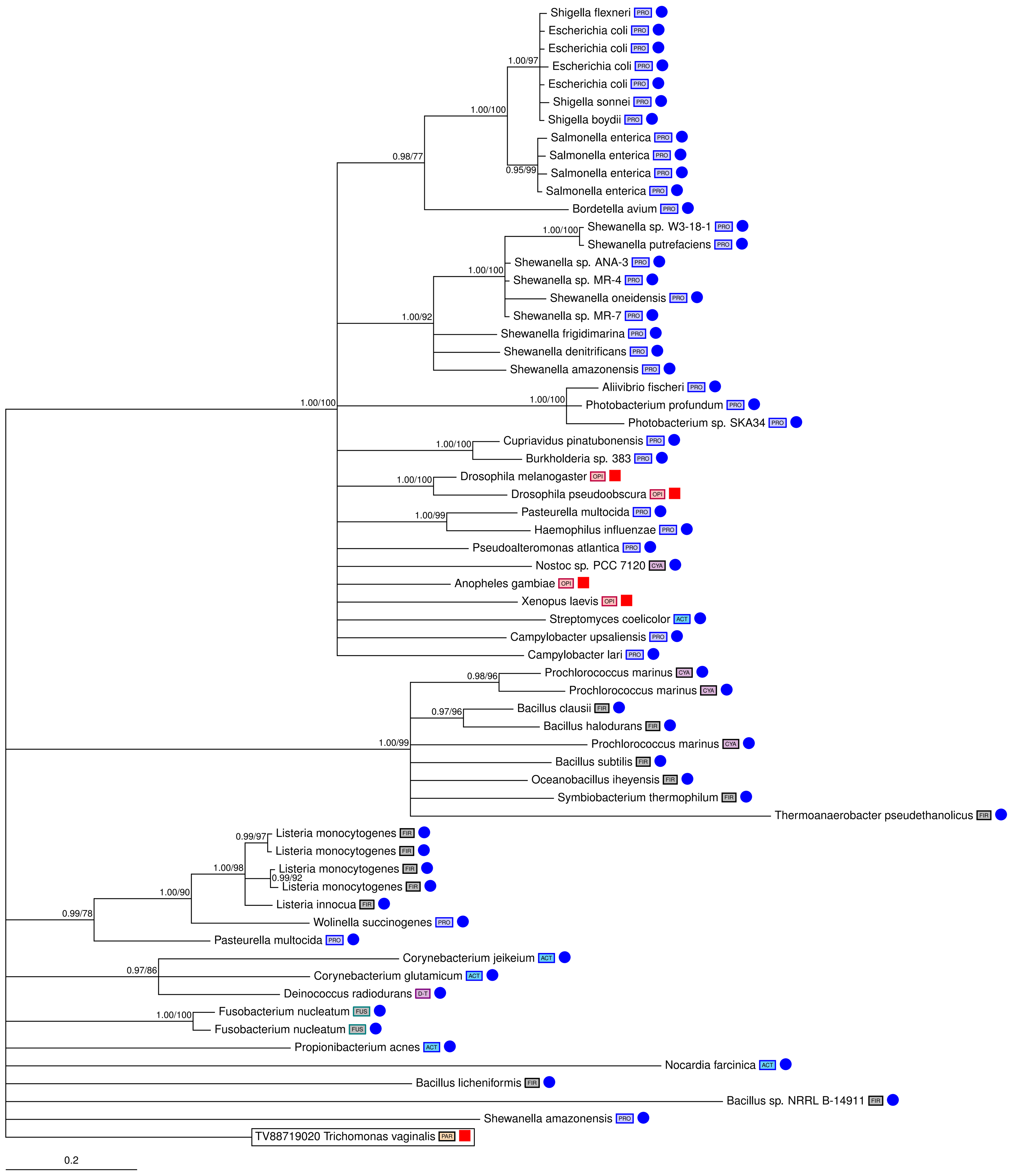

ON013

```
            Candy accession: TV88848003
           RefSeq accession: XP_001293713.1
          Uniprot accession: A2GPY4_TRIVA
                   Comments: LGT - TV ONE NODE - LGT INTO FUNGI
           Species affected: TV,FUNGI
       Adjacent taxa in tree: Proteobacteria
EC annotation - (Blast/Profile): EC:2.6.1.62
                PHOBIUS SP: 0
               PHOBIUS TMD: 0
          RefSeq annotation: adenosylmethionine-8-amino-7-oxononanoat
                             e aminotransferase family protein
   Name of enzyme/protein: adenosylmethionine-8-amino-7-oxononanoat
                             e transaminase
      KEGG PATHWAY - level 1: Metabolism of Cofactors and Vitamins
      KEGG PATHWAY - level 2: Biotin metabolism
```

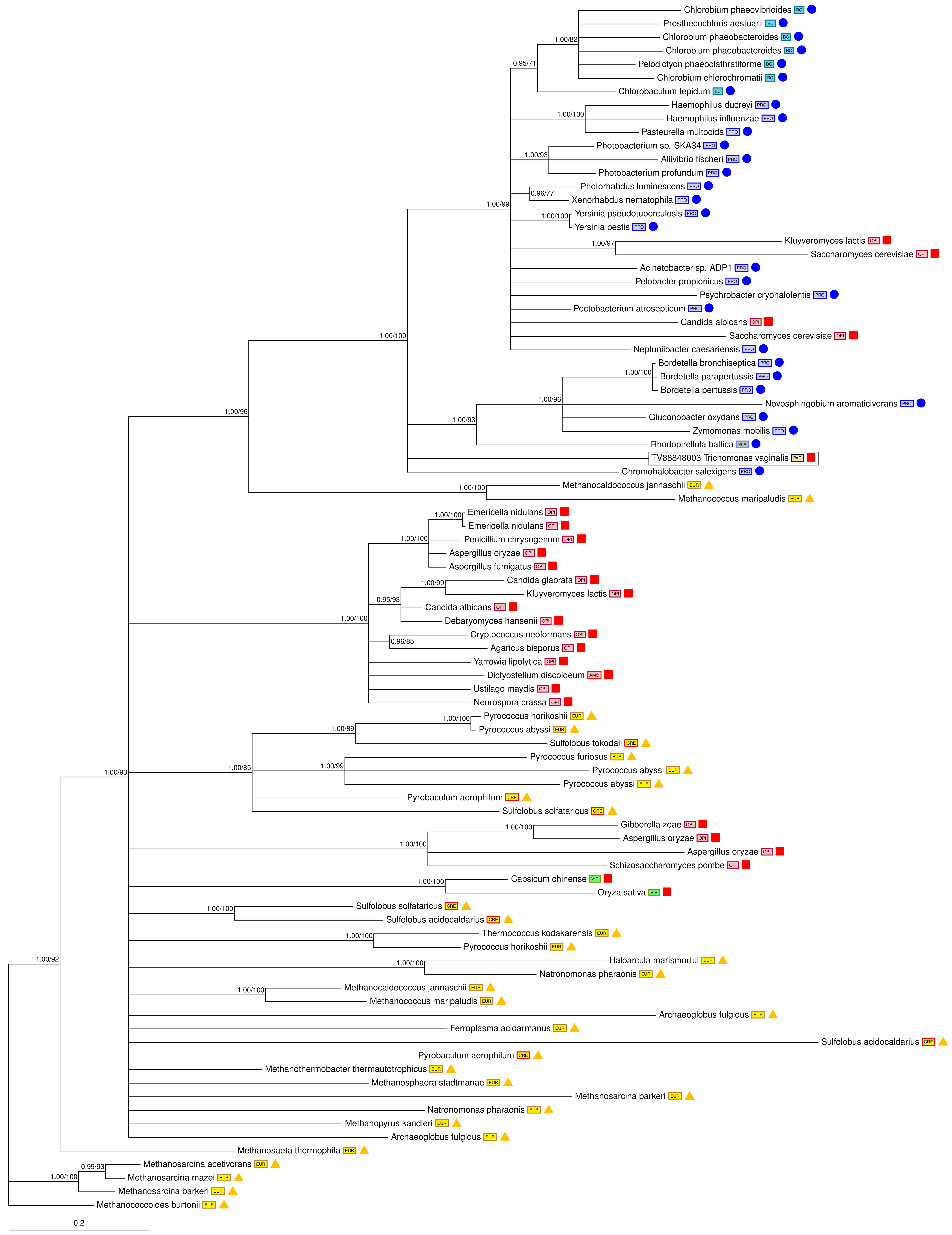

ON014

```
          Candy accession: TV92553049
         RefSeq accession: XP_001308730.1
        Uniprot accession: A2FH32_TRIVA
                 Comments: LGT - TV ONE NODE - 3 DOMAIN TREE
         Species affected: TV
   Adjacent taxa in tree: Bacteroidetes/Chlorobi  - Bacteroides
EC annotation - (Blast/Profile): EC:1.1.1.-
               PHOBIUS SP: 0
              PHOBIUS TMD: 0
        RefSeq annotation: oxidoreductase, short chain
                           dehydrogenase/reductase family protein
    Name of enzyme/protein: Oxidoreductase
    KEGG PATHWAY - level 1: Reaction
    KEGG PATHWAY - level 2: Reaction
```

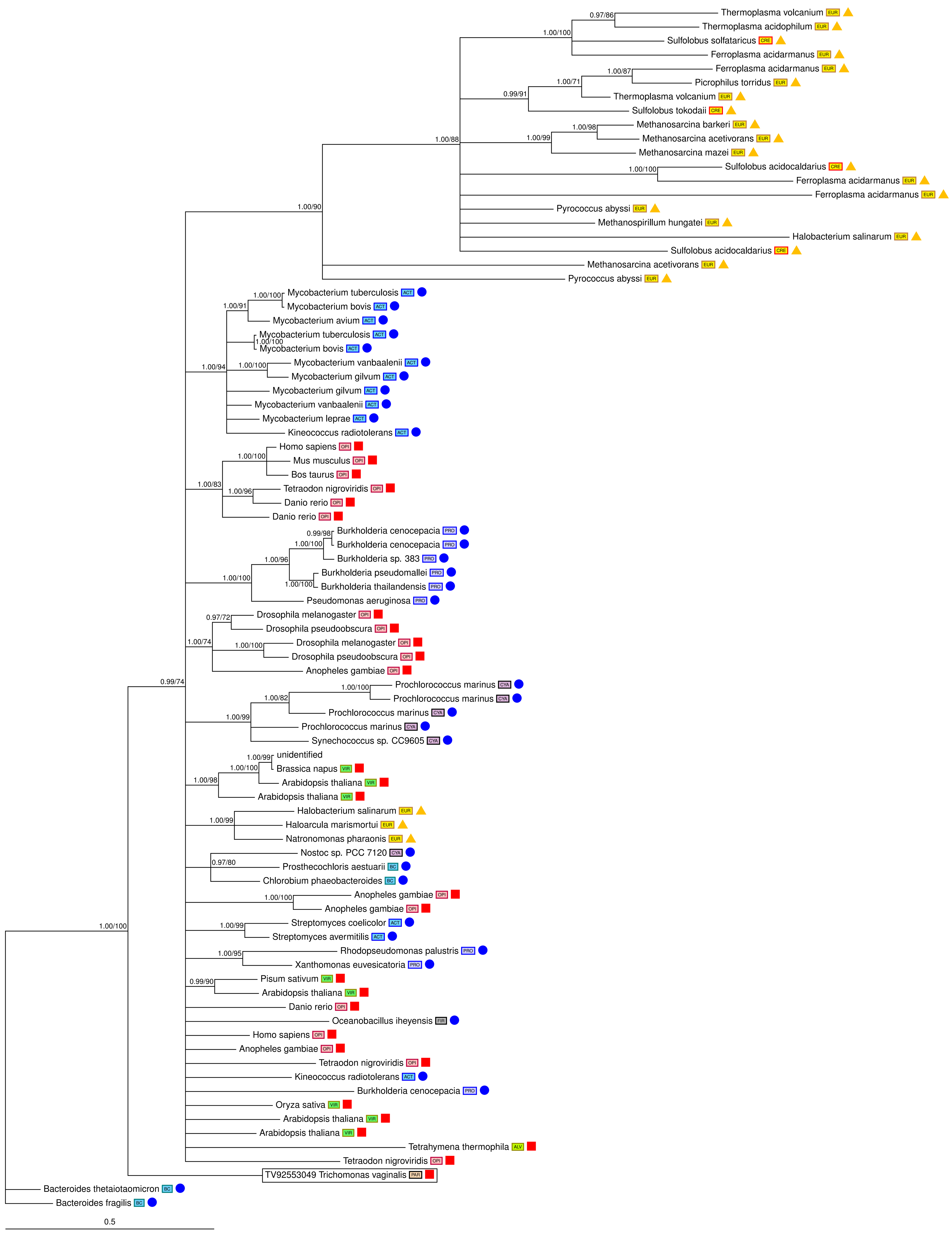

ON015

```
                Candy accession: TV97007147
               RefSeq accession: XP_001322689.1
              Uniprot accession: A2EA20_TRIVA
                       Comments: LGT - TV ONE NODE
               Species affected: TV
        Adjacent taxa in tree: Proteobacteria
EC annotation - (Blast/Profile): EC:3.2.1.25
                     PHOBIUS SP: 0
                    PHOBIUS TMD: 0
              RefSeq annotation: glycosyl hydrolase
      Name of enzyme/protein: beta-mannosidase
      KEGG PATHWAY - level 1: Glycan Biosynthesis and Metabolism
      KEGG PATHWAY - level 2: Other glycan degradation
```

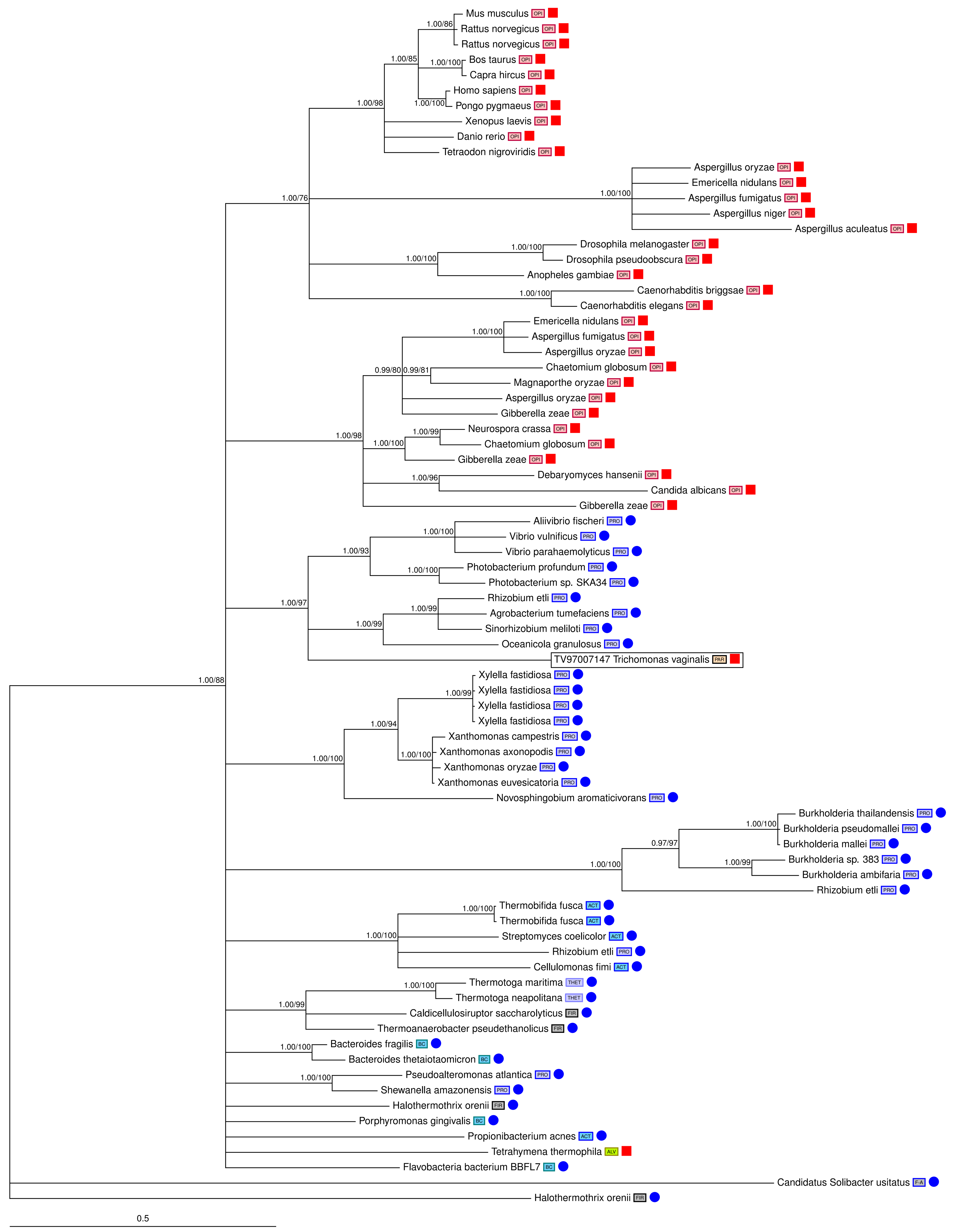

ON016

```
                Candy accession: TV97304085
               RefSeq accession: XP_001309329.1
              Uniprot accession: A2FFA7_TRIVA
                       Comments: LGT - TV ONE NODE
               Species affected: TV
          Adjacent taxa in tree: Photobacterium
EC annotation - (Blast/Profile): na
                     PHOBIUS SP: 0
                    PHOBIUS TMD: 4
              RefSeq annotation: PRS2 protein-related protein
       Name of enzyme/protein: Protein containing DUF962
         KEGG PATHWAY - level 1: Function unknown
         KEGG PATHWAY - level 2: na
```

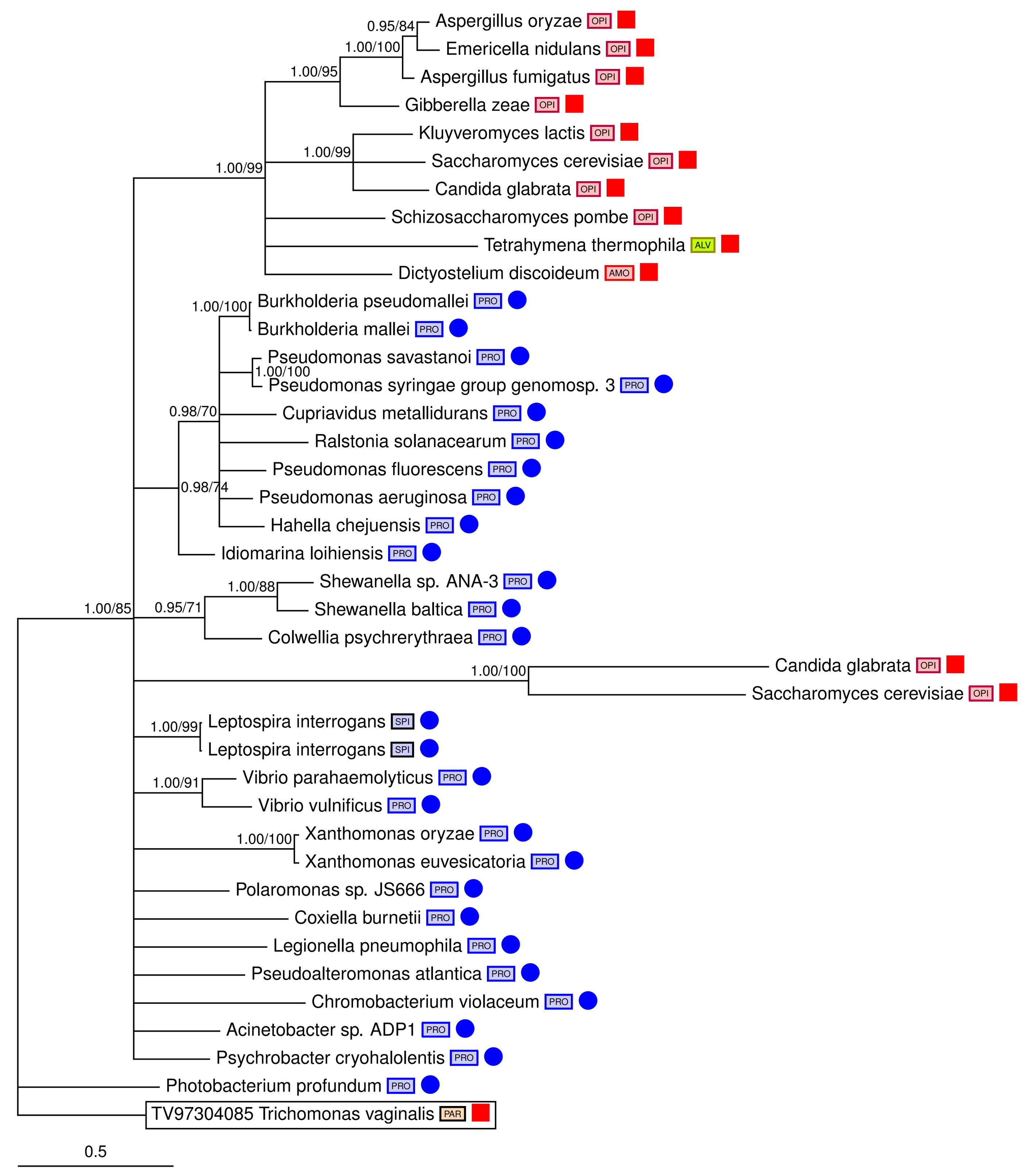

ON017

```
                Candy accession: TV101660002
               RefSeq accession: XP_001292898.1
              Uniprot accession: A2GS99_TRIVA
                       Comments: LGT - TV ONE NODE
               Species affected: TV
          Adjacent taxa in tree: Bacteria
EC annotation - (Blast/Profile): na
                     PHOBIUS SP: 0
                    PHOBIUS TMD: 1
              RefSeq annotation: antigen, 67 kD-related protein
         Name of enzyme/protein: Predicted myosin-cross-reactive antigen
                                 like family
         KEGG PATHWAY - level 1: Other function
         KEGG PATHWAY - level 2: na
```

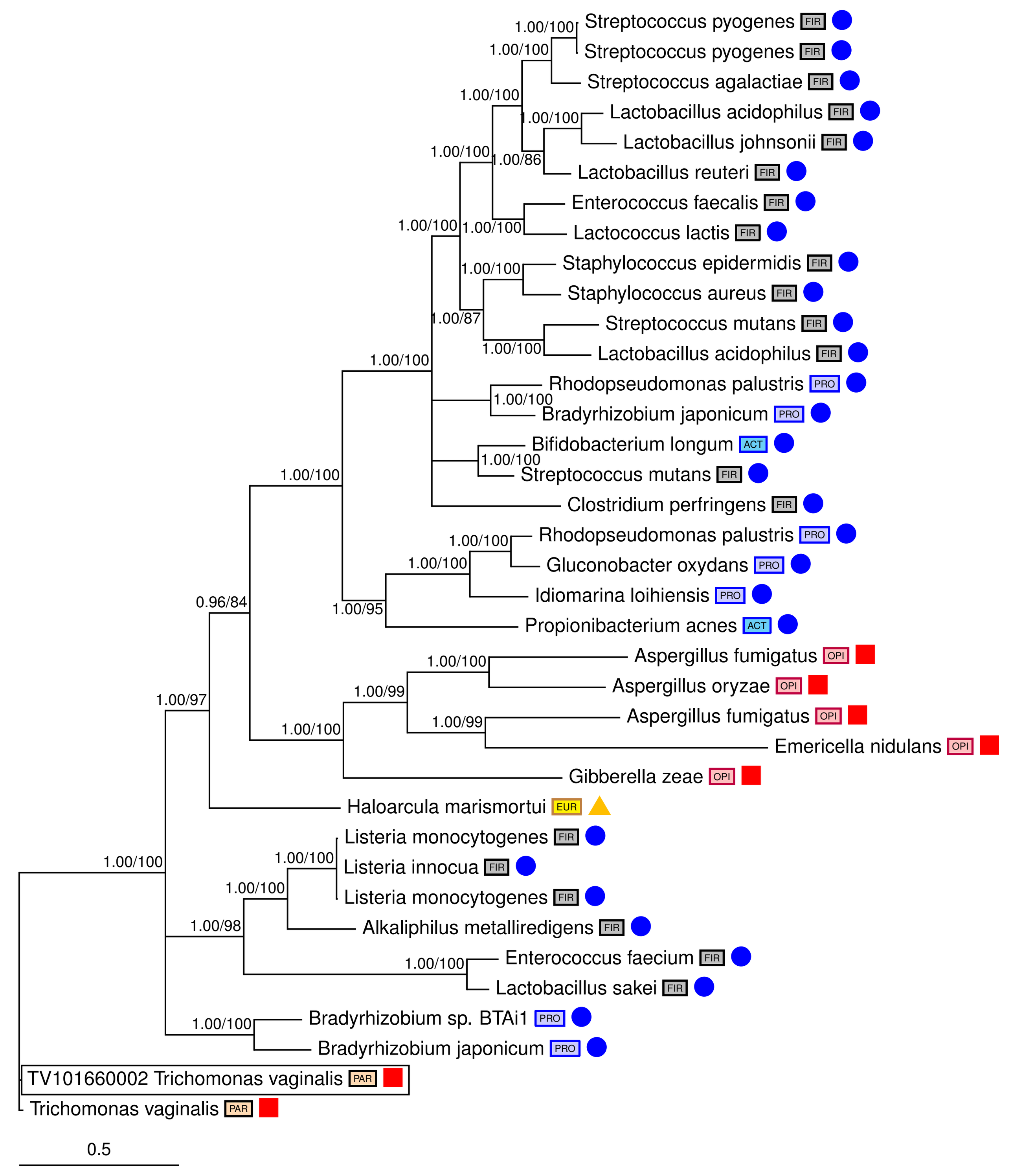

ON018

```
              Candy accession: TV89808120
             RefSeq accession: XP_001313482.1
            Uniprot accession: A2F3H3_TRIVA
                     Comments: LGT - TV ONE NODE
             Species affected: TV
        Adjacent taxa in tree: Protobacteria
EC annotation - (Blast/Profile): na
                   PHOBIUS SP: 0
                  PHOBIUS TMD: 0
            RefSeq annotation: CutC family protein
     Name of enzyme/protein: Predicted CutC Uncharacterized protein
                               involved in copper resistance
      KEGG PATHWAY - level 1: Other function
      KEGG PATHWAY - level 2: na
```

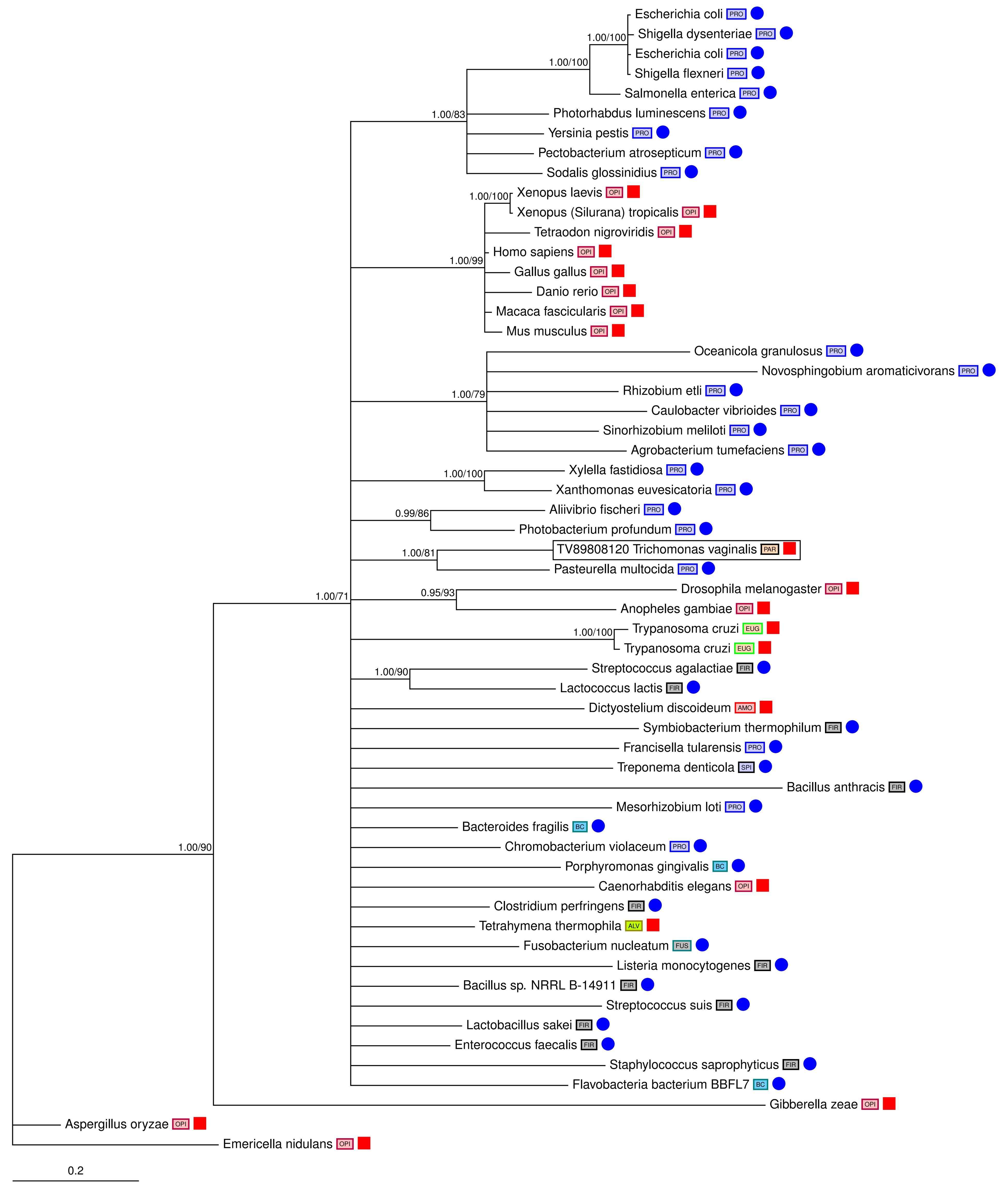

ON019

```
           Candy accession: Q4Q7G6_LEIMA
          RefSeq accession: XP_001684732.1
         Uniprot accession: Q4Q7G6_LEIMA
                  Comments: LGT - KINETOPLASTID ONE NODE +
                            DUPLICATIONS IN LM
          Species affected: LM,TB,TC
     Adjacent taxa in tree: Prokaryote
EC annotation - (Blast/Profile): EC:3.5.1.14
               PHOBIUS SP: 0
              PHOBIUS TMD: 0
         RefSeq annotation: n-acyl-l-amino acid amidohydrolase
    Name of enzyme/protein: aminoacylase
    KEGG PATHWAY - level 1: Amino Acid Metabolism
    KEGG PATHWAY - level 2: Arginine and proline metabolism
```

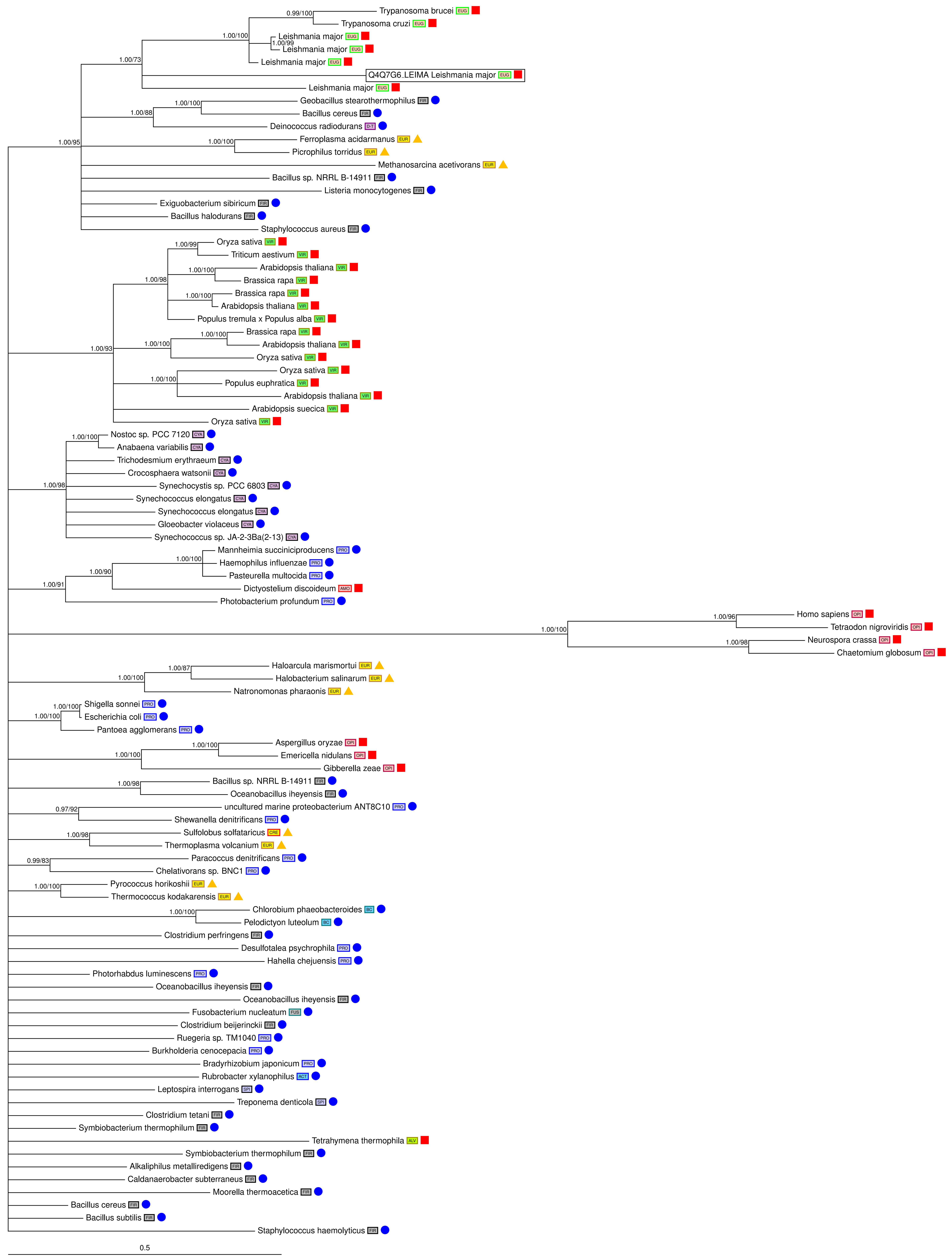

ON020

```
                Candy accession: Q4Q9B1_LEIMA
               RefSeq accession: XP_001684087.1
              Uniprot accession: Q4Q9B1_LEIMA
                       Comments: LGT - KINETOPLASTIDS ONE NODE
               Species affected: LM,TB,TC
          Adjacent taxa in tree: Proteobacteria
EC annotation - (Blast/Profile): EC:6.3.1.1
                     PHOBIUS SP: 0
                    PHOBIUS TMD: 0
              RefSeq annotation: asparagine synthetase a; aspartate--
                                 ammonia ligase
         Name of enzyme/protein: aspartate-ammonia ligase
         KEGG PATHWAY - level 1: Amino Acid Metabolism, Metabolism of
                                 Other Amino Acids, Energy Metabolism
         KEGG PATHWAY - level 2: Alanine, aspartate and glutamate
                                 metabolism, Cyanoamino acid metabolism,
                                 Nitrogen metabolism

                Candy accession: Q51GZ9_ENTHI
               RefSeq accession: XP_657492.1
              Uniprot accession: C4LT17_ENTHI
                       Comments: LGT - EH ONE NODE -3 DOMAIN TREE
               Species affected: EH
          Adjacent taxa in tree: Bacteroidetes/Chlorobi  - Bacteroides
EC annotation - (Blast/Profile): EC:6.3.1.1
                     PHOBIUS SP: 0
                    PHOBIUS TMD: 0
              RefSeq annotation: aspartate--ammonia ligase
         Name of enzyme/protein: aspartate-ammonia ligase
         KEGG PATHWAY - level 1: Amino Acid Metabolism, Metabolism of
                                 Other Amino Acids, Energy Metabolism
         KEGG PATHWAY - level 2: Alanine, aspartate and glutamate
                                 metabolism, Cyanoamino acid metabolism,
                                 Nitrogen metabolism
```

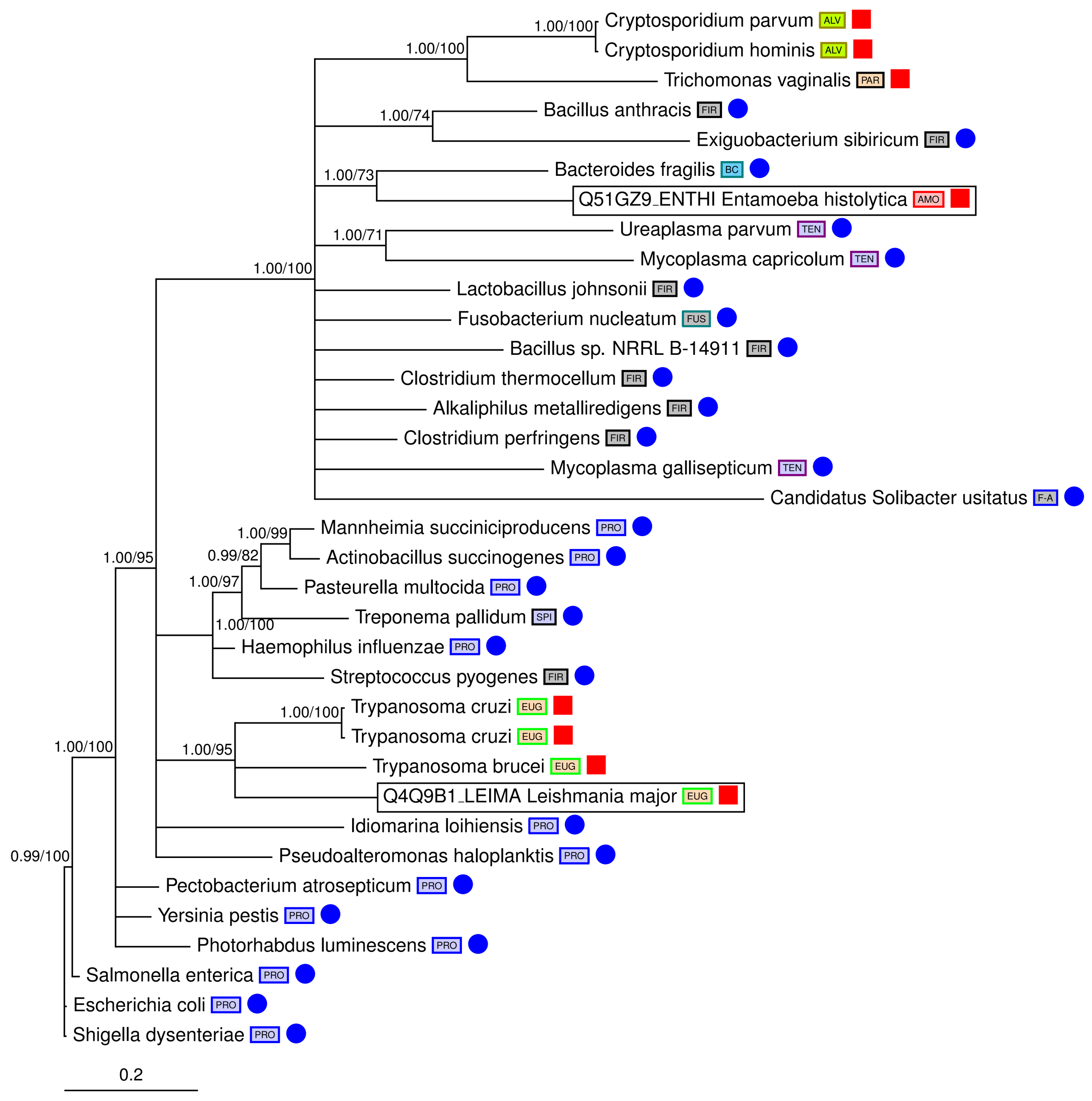

ON021

```
                Candy accession: TV93601047
               RefSeq accession: XP_001304645.1
              Uniprot accession: A2FTP3_TRIVA
                       Comments: LGT -  TV ONE NODE
               Species affected: TV
           Adjacent taxa in tree: Ambiguous
EC annotation - (Blast/Profile): EC:3.5.2.2
                     PHOBIUS SP: 0
                    PHOBIUS TMD: 0
              RefSeq annotation: D-hydantoinase family protein
          Name of enzyme/protein: dihydropyrimidinase
           KEGG PATHWAY - level 1: Nucleotide Metabolism, Metabolism of
                                  Other Amino Acids, Metabolism of
                                  Cofactors and Vitamins
           KEGG PATHWAY - level 2: Pyrimidine metabolism, beta-Alanine
                                  metabolism, Pantothenate and CoA
                                  biosynthesis
```

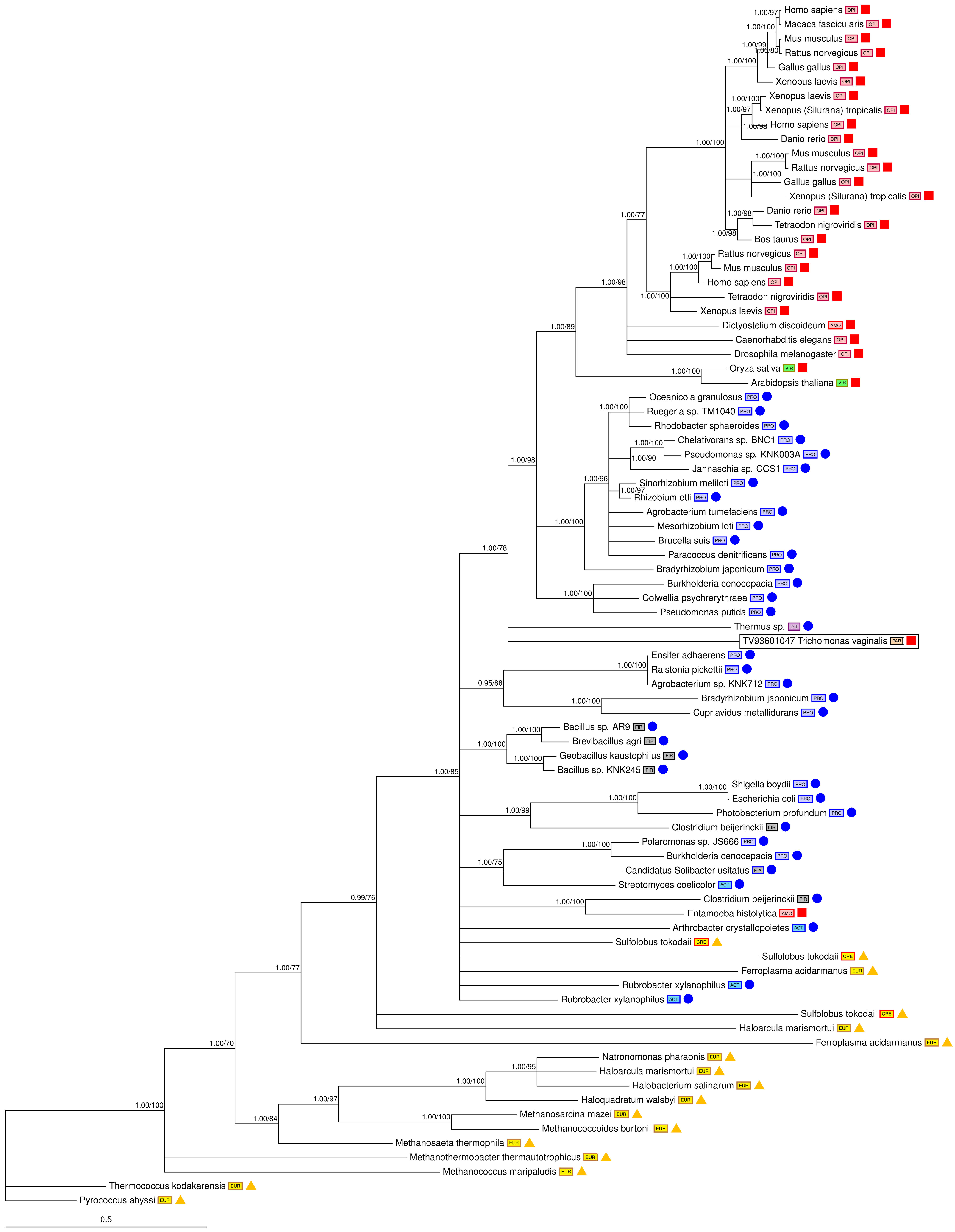

ON022

```
                Candy accession: HEM6_LEIMA
               RefSeq accession: XP_001680900.1
              Uniprot accession: HEM6_LEIMA
                       Comments: LGT - ML ONE NODE
               Species affected: DD
          Adjacent taxa in tree: Proteobacteria
EC annotation - (Blast/Profile): EC:1.3.3.3
                     PHOBIUS SP: 0
                    PHOBIUS TMD: 0
              RefSeq annotation: coproporphyrinogen iii oxidase
         Name of enzyme/protein: coproporphyrinogen oxidase
        KEGG PATHWAY - level 1: Metabolism of Cofactors and Vitamins
        KEGG PATHWAY - level 2: Porphyrin and chlorophyll metabolism
```

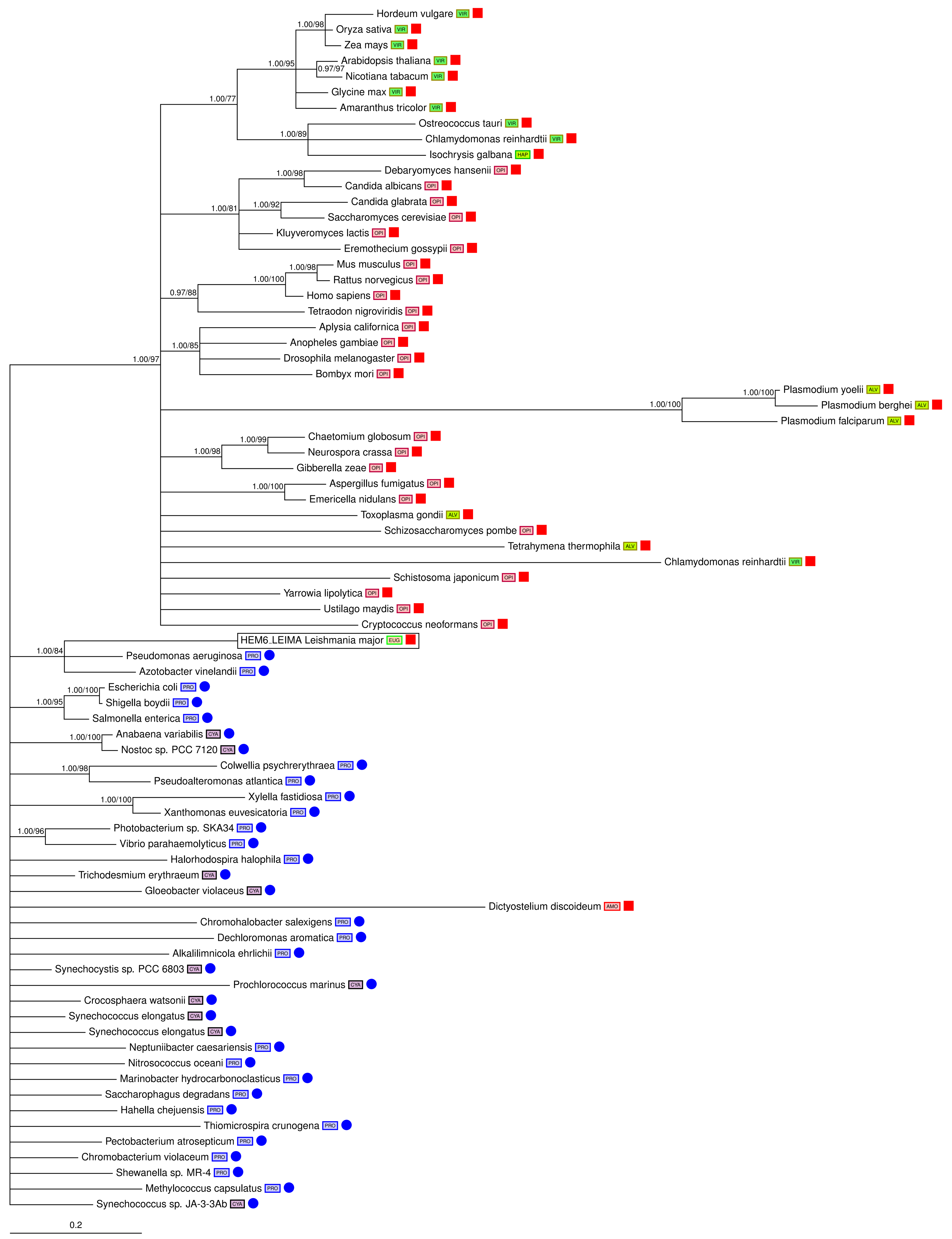

ON023

```
            Candy accession: TV94596075
          RefSeq accession: XP_001315334.1
         Uniprot accession: A2EW75_TRIVA
                  Comments: LGT - ONE NODE 3 DOMAIN TREE
          Species affected: TV
      Adjacent taxa in tree: Firmicutes - Desulfitobacterium
EC annotation - (Blast/Profile): na
               PHOBIUS SP: 0
              PHOBIUS TMD: 0
          RefSeq annotation: hypothetical protein
   Name of enzyme/protein: Predicted pirin-like protein
   KEGG PATHWAY - level 1: Function unknown
   KEGG PATHWAY - level 2: na
```

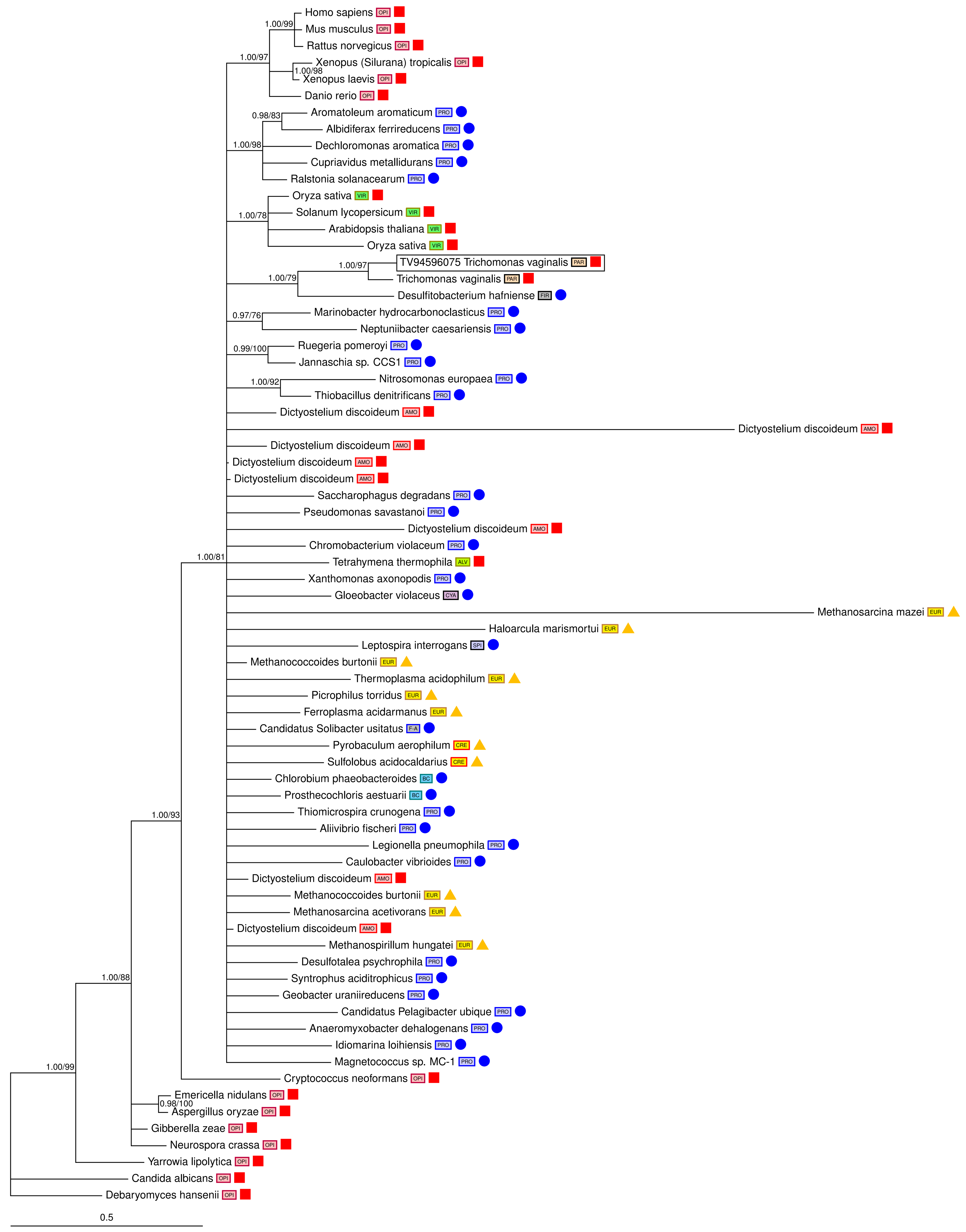

ON024

```
                Candy accession: TV100018002
               RefSeq accession: XP_001307119.1
              Uniprot accession: A2FLL6_TRIVA
                       Comments: LGT - TV ONE NODE
               Species affected: TV
         Adjacent taxa in tree: Bacteria
EC annotation - (Blast/Profile): EC:4.1.1.32
                    PHOBIUS SP: 0
                   PHOBIUS TMD: 0
             RefSeq annotation: phosphoenol pyruvate carboxykinase
     Name of enzyme/protein: phosphoenolpyruvate carboxykinase (GTP)
        KEGG PATHWAY - level 1: Carbohydrate Metabolism
        KEGG PATHWAY - level 2: Glycolysis / Gluconeogenesis, Citrate
                                cycle (TCA cycle), Pyruvate metabolism
```

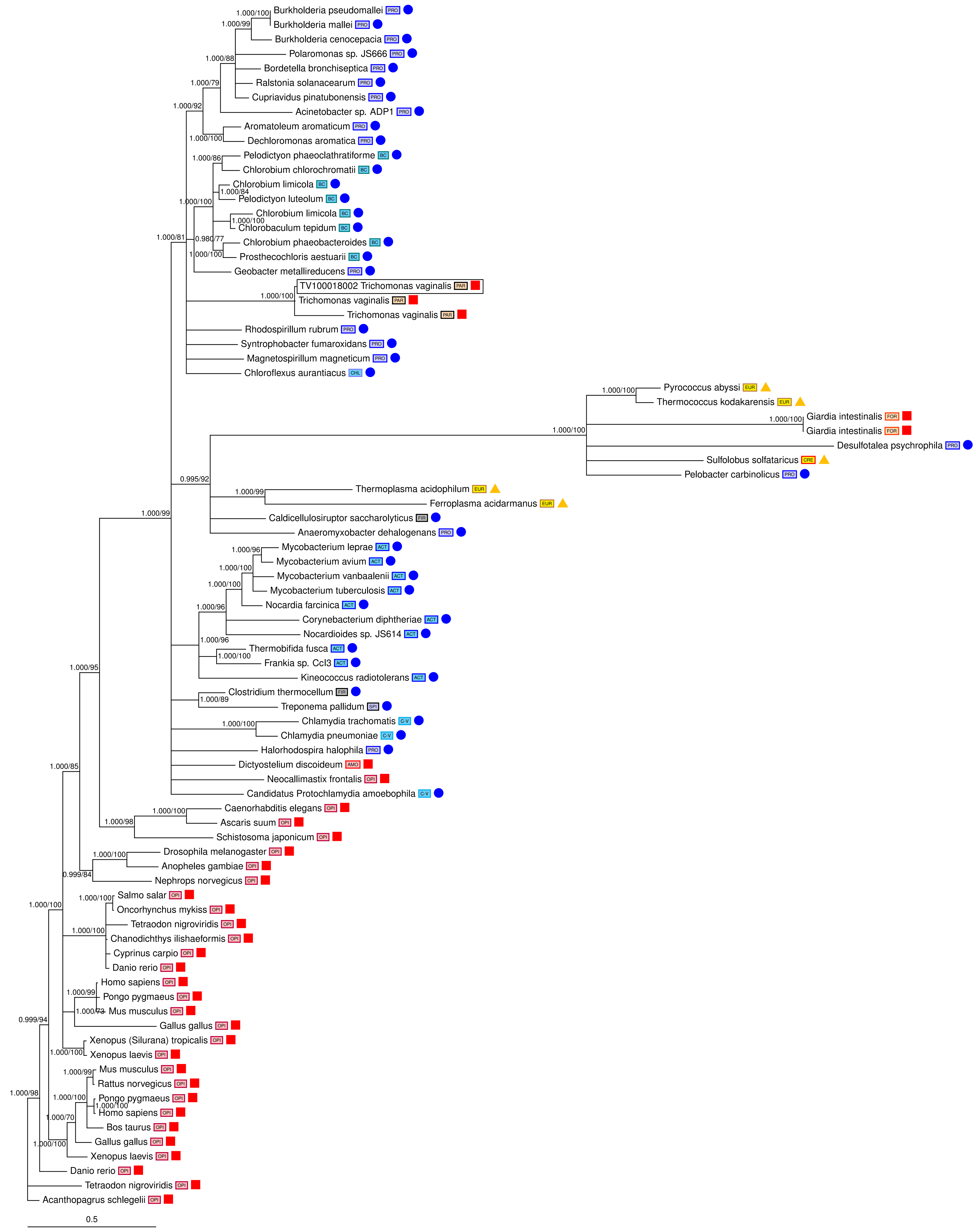

ON025

```
                 Candy accession: TV80596181
                RefSeq accession: XP_001329989.1
               Uniprot accession: A2DPO5_TRIVA
                        Comments: LGT - TV ONE NODE WITH BACTEROIDES
                Species affected: TV
          Adjacent taxa in tree: Bacteroidetes/Chlorobi  - Bacteroides
EC annotation - (Blast/Profile): EC:3.2.1.52
                      PHOBIUS SP: 0
                     PHOBIUS TMD: 0
               RefSeq annotation: glycosyl hydrolase
         Name of enzyme/protein: beta-N-acetylhexosaminidase
         KEGG PATHWAY - level 1: Glycan Biosynthesis and Metabolism,
                                  Carbohydrate Metabolism
         KEGG PATHWAY - level 2: Other glycan degradation, Various types
                                  of N-glycan biosynthesis, Amino sugar
                                  and nucleotide sugar metabolism
```

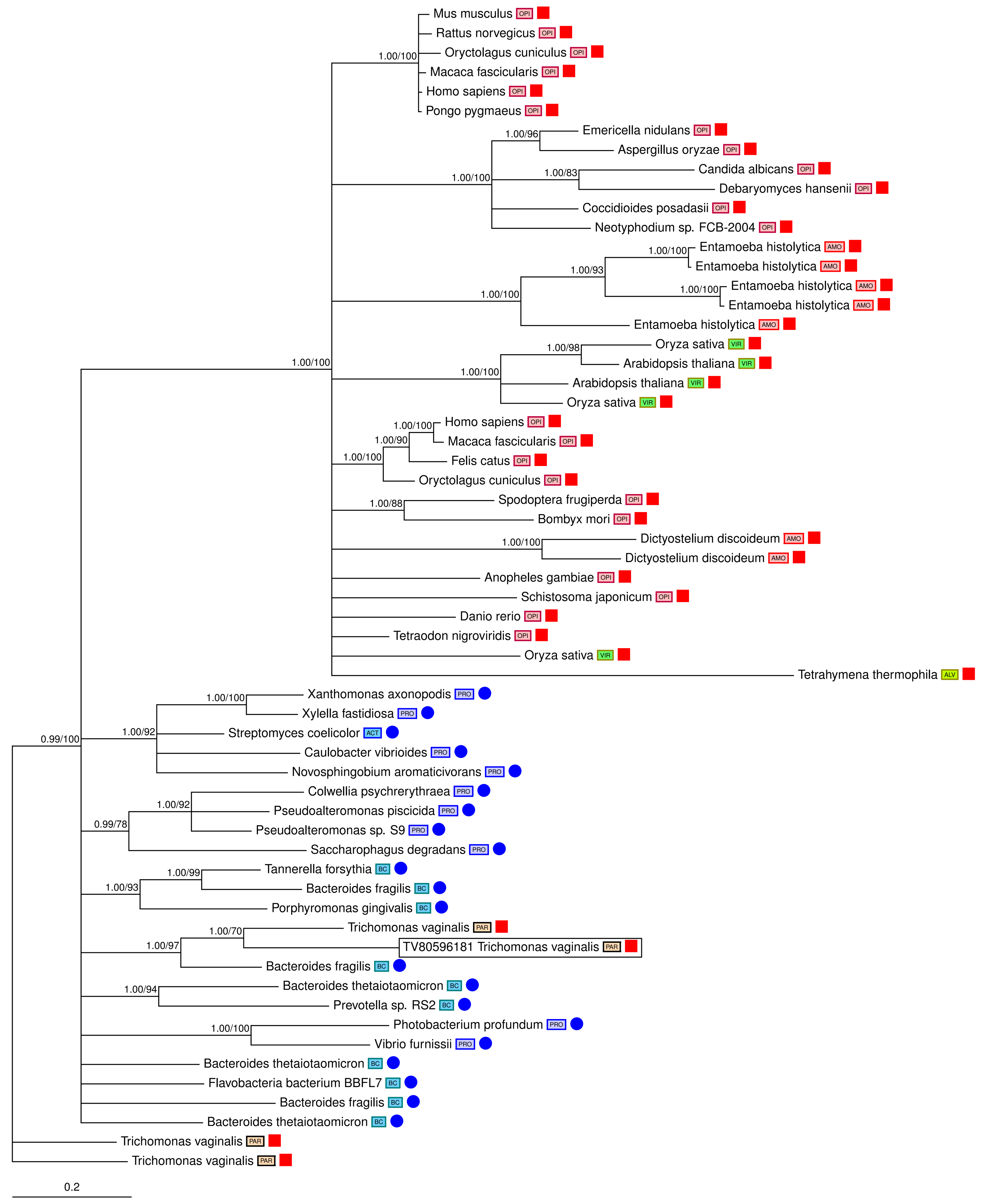

ON026

Candy accession: Q54CI9_DICDI
RefSeq accession: XP_629427.1
Uniprot accession: GLUA_DICDI
Comments: LGT - DD ONE NODE
Species affected: DD
Adjacent taxa in tree: Bacteria
EC annotation - (Blast/Profile): EC:3.2.1.21
PHOBIUS SP: Y
PHOBIUS TMD: 0
RefSeq annotation: beta glucosidase
Name of enzyme/protein: beta-glucosidase
KEGG PATHWAY - level 1: Metabolism of Other Amino Acids, Carbohydrate Metabolism, Biosynthesis of Other Secondary Metabolites
KEGG PATHWAY - level 2: Cyanoamino acid metabolism, Starch and sucrose metabolism, Phenylpropanoid biosynthesis

Candy accession: TV80671424
RefSeq accession: XP_001582566.1
Uniprot accession: A2DDA1_TRIVA
Comments: LGT - TV ONE NODE
Species affected: TV
Adjacent taxa in tree: Actinobacteria - Bifidobacterium
EC annotation - (Blast/Profile): EC:3.2.1.21
PHOBIUS SP: 0
PHOBIUS TMD: 0
RefSeq annotation: glycosyl hydrolase
Name of enzyme/protein: beta-glucosidase
KEGG PATHWAY - level 1: Metabolism of Other Amino Acids, Carbohydrate Metabolism, Biosynthesis of Other Secondary Metabolites
KEGG PATHWAY - level 2: Cyanoamino acid metabolism, Starch and sucrose metabolism, Phenylpropanoid biosynthesis

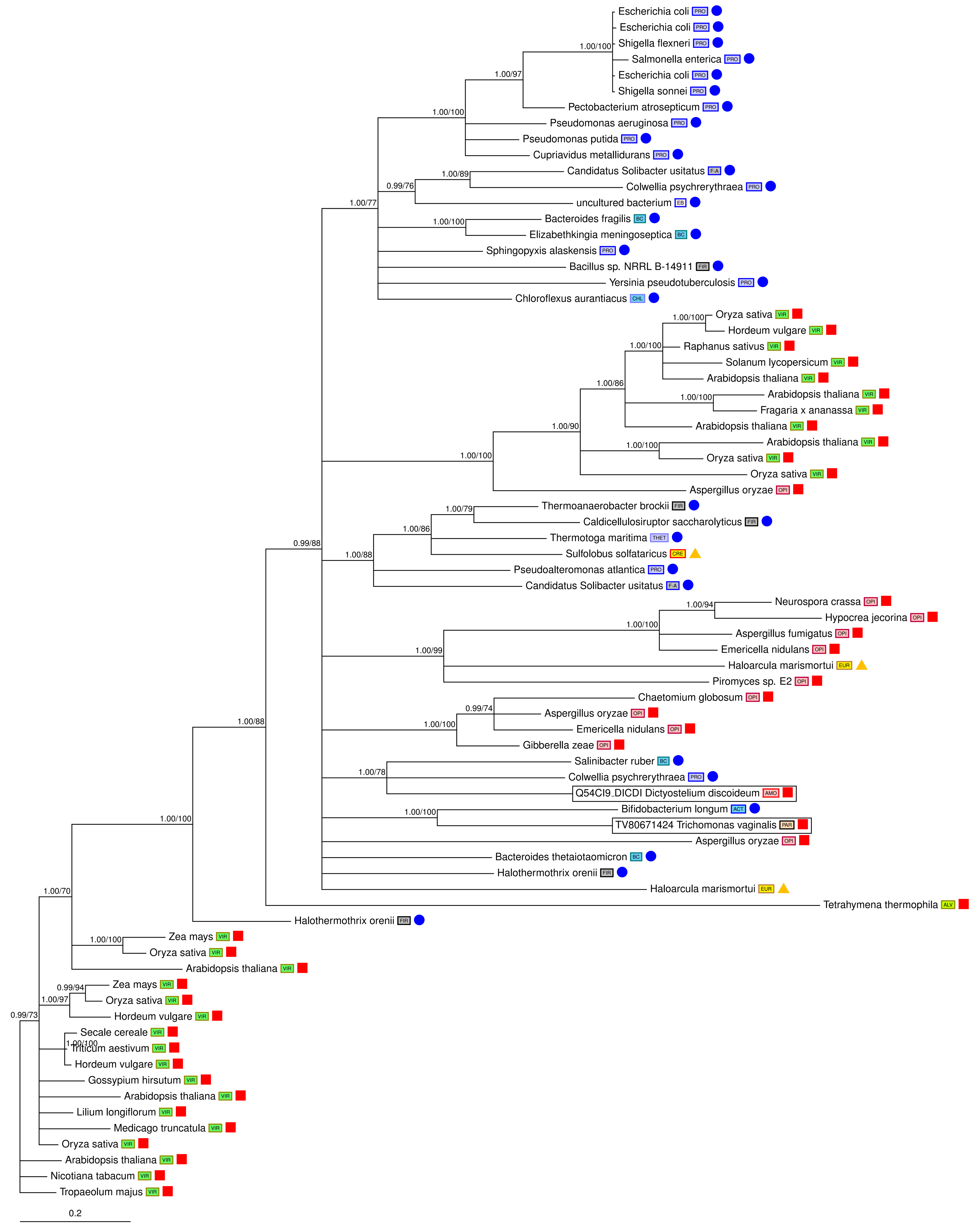

ON027

```
          Candy accession: Q55E34_DICDI
         RefSeq accession: XP_645947.1
        Uniprot accession: P5CR2_DICDI
                 Comments: LGT - DD ONE NODE
         Species affected: DD
   Adjacent taxa in tree: Bacteroidetes/Chlorobi  - Bacteroides
EC annotation - (Blast/Profile): EC:1.5.1.2
               PHOBIUS SP: 0
              PHOBIUS TMD: 0
        RefSeq annotation: pyrroline-5-carboxylate reductase
  Name of enzyme/protein: pyrroline-5-carboxylate reductase
   KEGG PATHWAY - level 1: Amino Acid Metabolism
   KEGG PATHWAY - level 2: Arginine and proline metabolism,
```

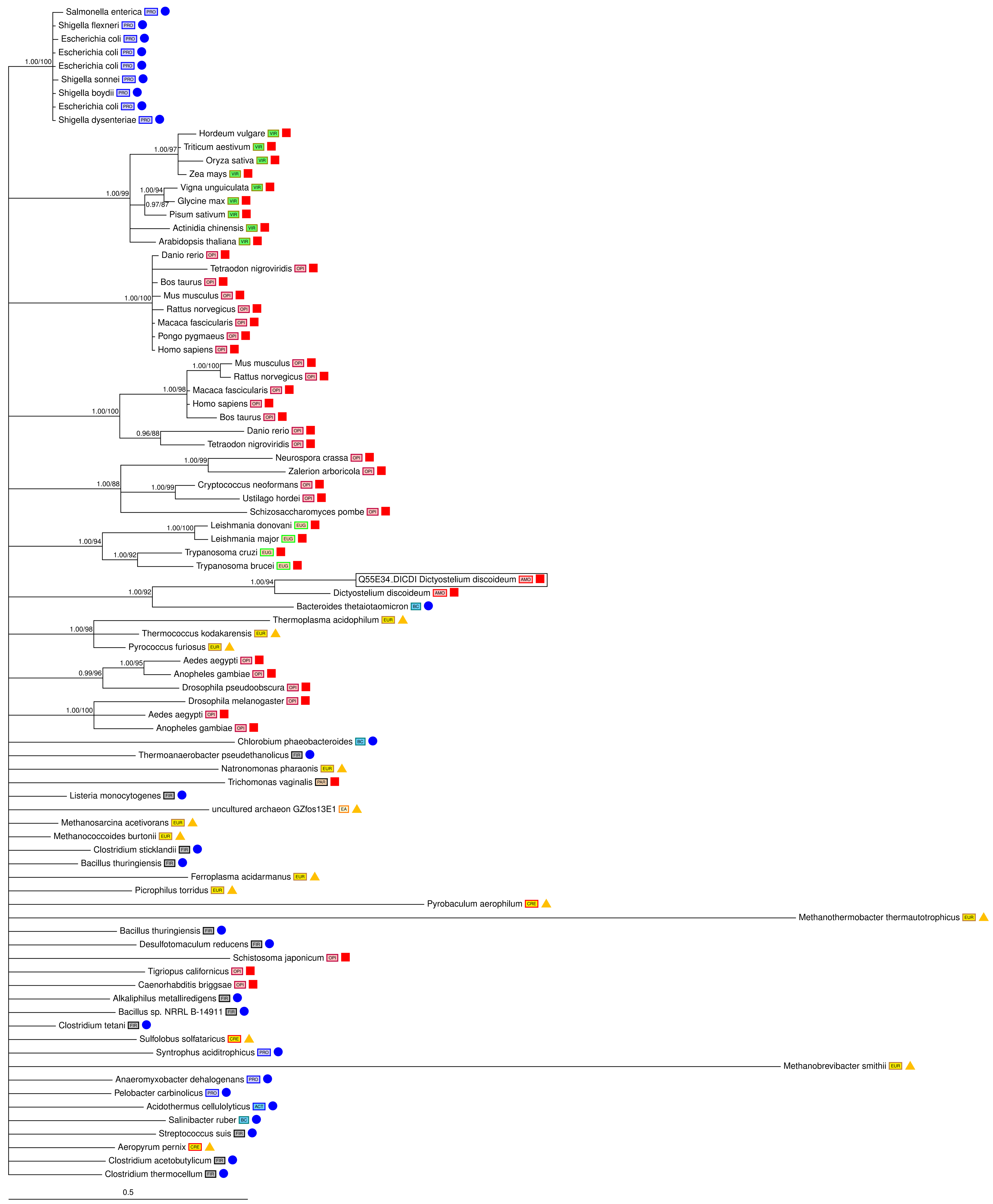

ON028

```
                Candy accession: TV86763156
               RefSeq accession: XP_001316223.1
              Uniprot accession: A2ETN2_TRIVA
                       Comments: LGT - TV ONE NODE WITH BACTEROIDES
               Species affected: TV
          Adjacent taxa in tree: Bacteroidetes/Chlorobi  - Bacteroides
EC annotation - (Blast/Profile): EC:3.1.3.5
                     PHOBIUS SP: 0
                    PHOBIUS TMD: 0
              RefSeq annotation: Acid phosphatase surE
        Name of enzyme/protein: 5'-nucleotidase
        KEGG PATHWAY - level 1: Nucleotide Metabolism, Metabolism of
                                Cofactors and Vitamins
        KEGG PATHWAY - level 2: Purine metabolism, Pyrimidine
                                metabolism, Nicotinate and nicotinamide
                                metabolism
```

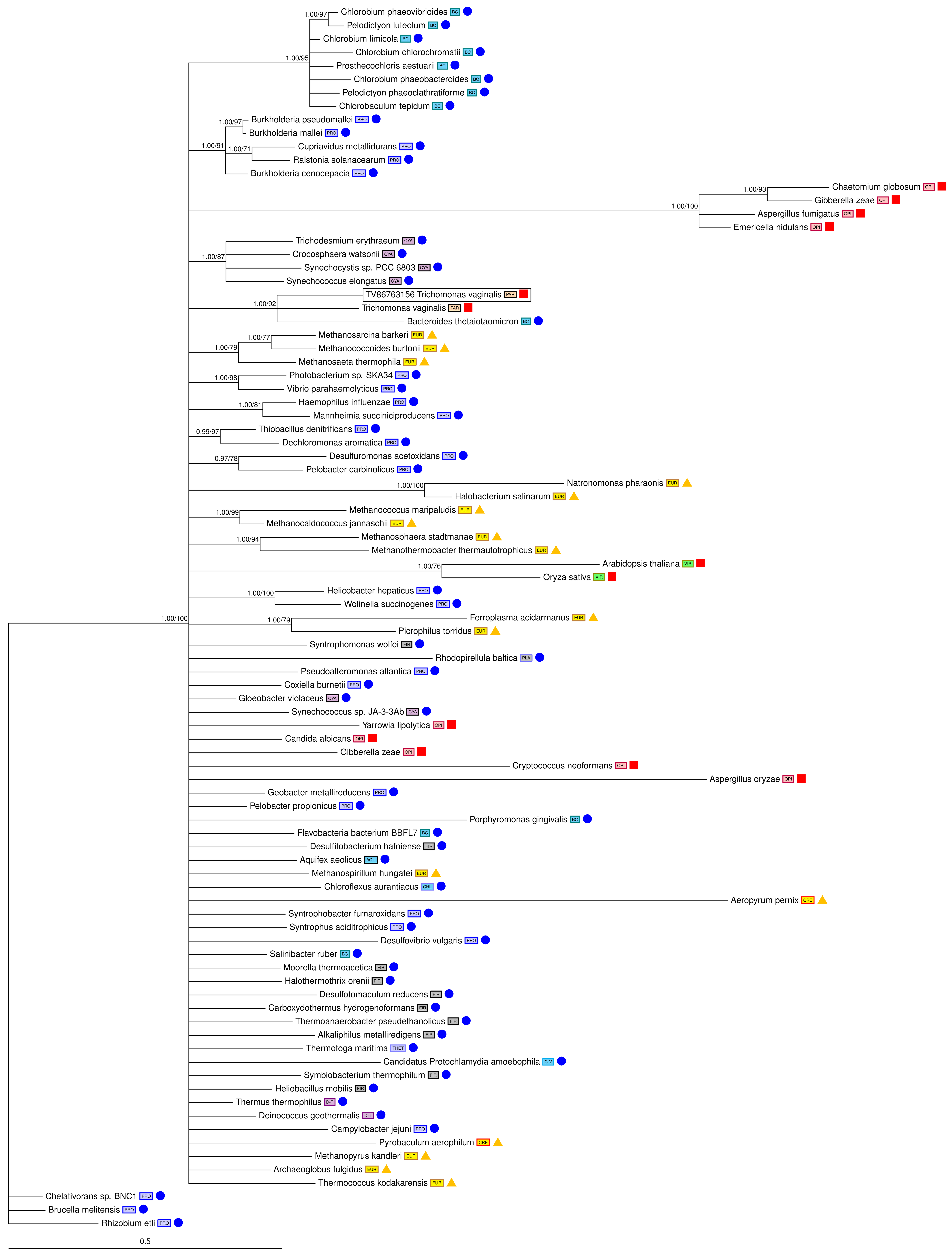

ON029

```
                Candy accession: T583_05667
               RefSeq accession: XP_002364705.1
              Uniprot accession: B6K9I1_TOXGO
                       Comments: LGT - TV ONE NODE WITH BACTEROIDES
               Species affected: TV
          Adjacent taxa in tree: Bacteroidetes/Chlorobi  - Bacteroides
EC annotation - (Blast/Profile): EC:1.4.1.1
                     PHOBIUS SP: 0
                    PHOBIUS TMD: 0
              RefSeq annotation: alanine dehydrogenase, putative
         Name of enzyme/protein: alanine dehydrogenase
         KEGG PATHWAY - level 1: Amino Acid Metabolism, Metabolism of
                                 Other Amino Acids
         KEGG PATHWAY - level 2: Alanine, aspartate and glutamate
                                 metabolism, Taurine and hypotaurine
                                 metabolism
```

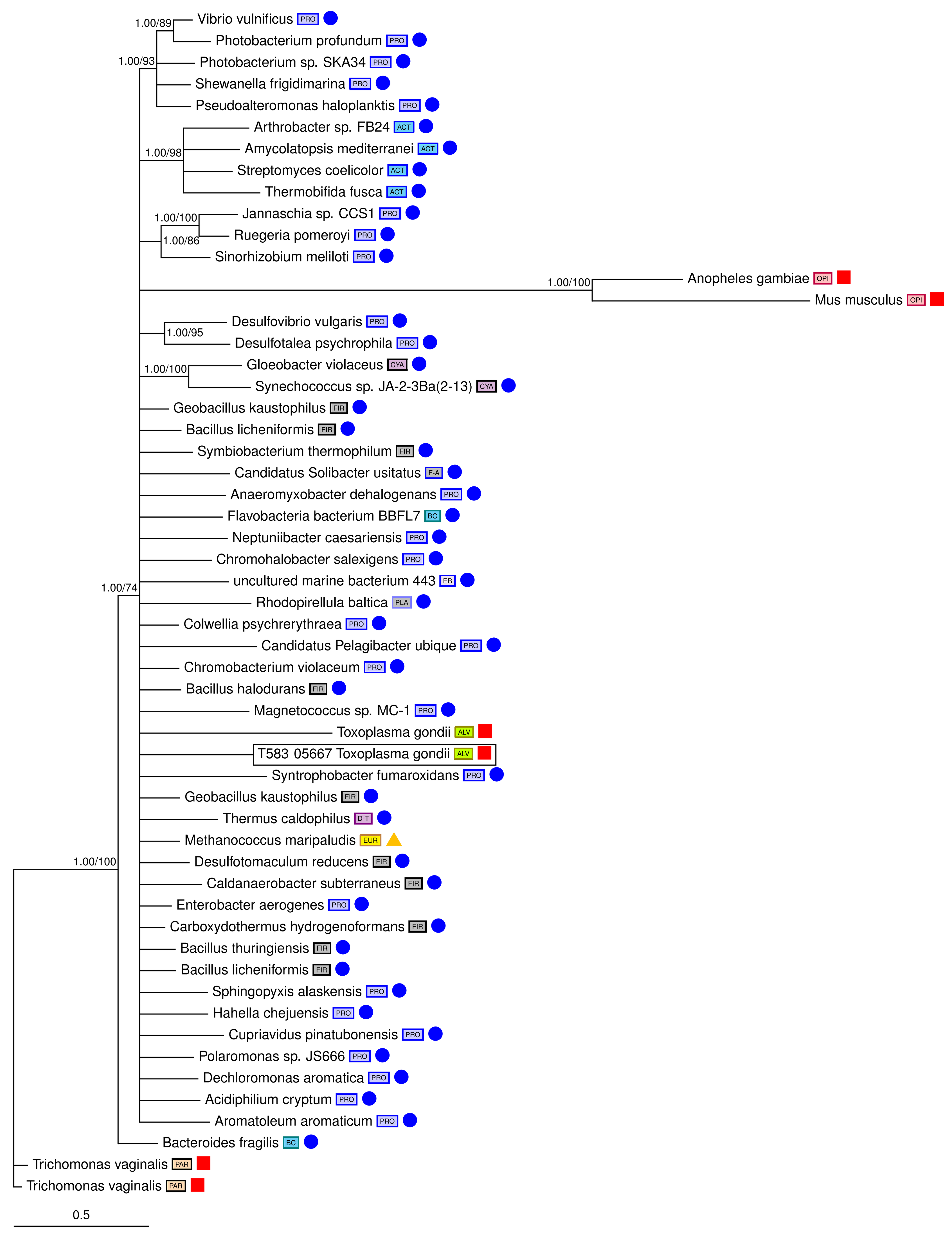

ON030

```
              Candy accession: T641_00183
             RefSeq accession: XP_002369919.1
            Uniprot accession: B6KPP7_TOXGO
                     Comments: LGT - APICOMPLEXA + HETEROCAPSA ONE NODE
             Species affected: PF,PV,PY,PC,TG,HT
         Adjacent taxa in tree: Proteobacteria
EC annotation - (Blast/Profile): EC:2.4.2.29
                   PHOBIUS SP: 0
                  PHOBIUS TMD: 0
            RefSeq annotation: queuine tRNA-ribosyltransferase,
                               putative
        Name of enzyme/protein: tRNA-guanine transglycosylase
       KEGG PATHWAY - level 1: Reaction
       KEGG PATHWAY - level 2: Reaction
```

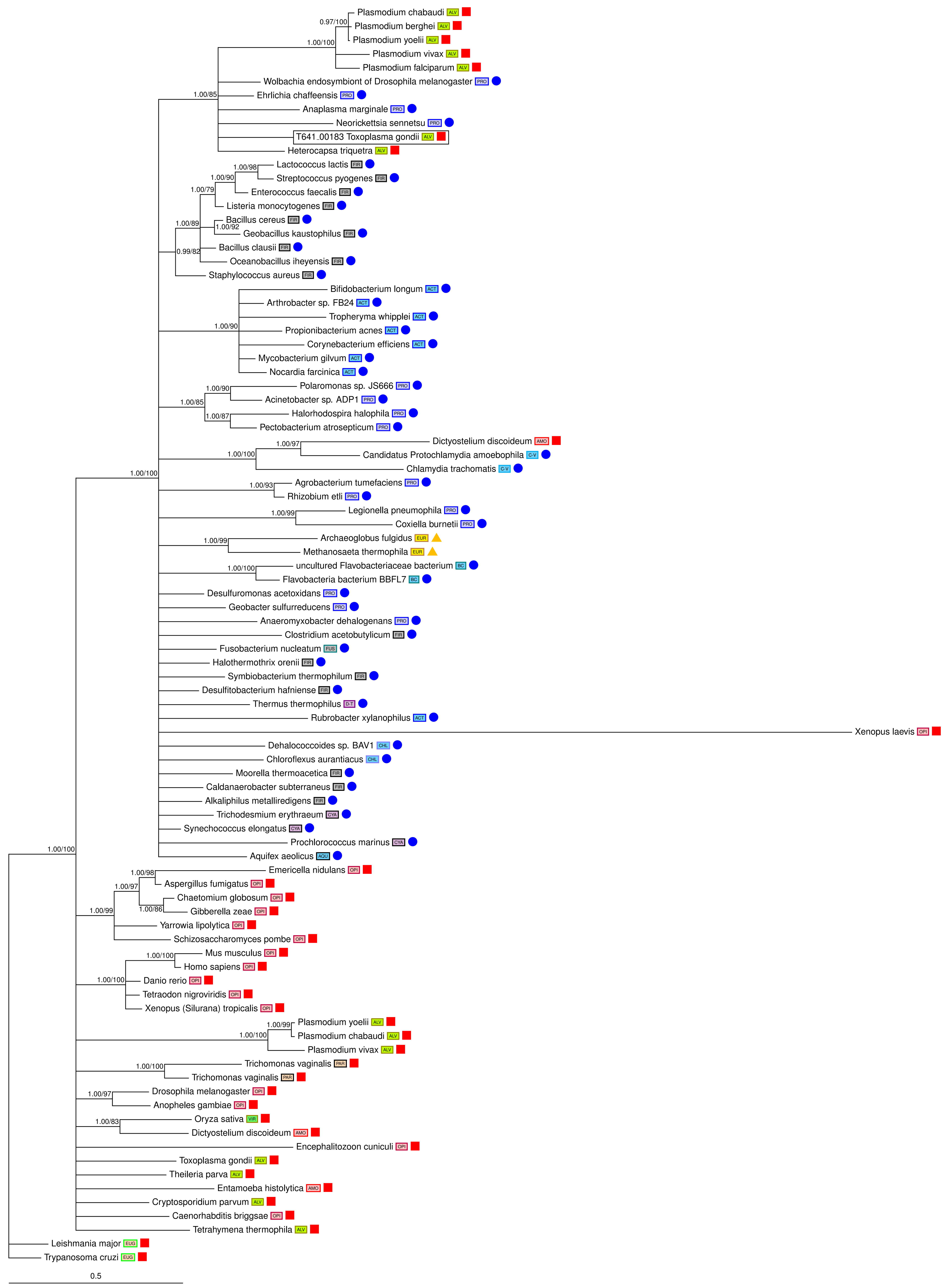

ON031

```
               Candy accession: TV94534123
              RefSeq accession: XP_001321965.1
             Uniprot accession: A2EC65_TRIVA
                      Comments: LGT - TV ONE NODE - WITH ANAEROBIC FUNGI
                                - 3 DOMAIN ToL
              Species affected: TV,FUNGI
         Adjacent taxa in tree: Bacteria
EC annotation - (Blast/Profile): EC:2.7.1.17
                   PHOBIUS SP: 0
                  PHOBIUS TMD: 0
             RefSeq annotation: xylulokinase family protein
        Name of enzyme/protein: Xylulokinase
        KEGG PATHWAY - level 1: Carbohydrate Metabolism
        KEGG PATHWAY - level 2: Pentose and glucuronate interconversions

               Candy accession: TV91130026
              RefSeq accession: XP_001302338.1
             Uniprot accession: A2G0B1_TRIVA
                      Comments: LGT - TV ONE NODE - 3 DOMAIN ToL
              Species affected: TV
         Adjacent taxa in tree: Firmicutes
EC annotation - (Blast/Profile): EC:2.7.1.17
                   PHOBIUS SP: 0
                  PHOBIUS TMD: 0
             RefSeq annotation: FGGY family of carbohydrate kinases,
                                N-terminal domain containing protein
        Name of enzyme/protein: Xylulokinase
        KEGG PATHWAY - level 1: Carbohydrate Metabolism
        KEGG PATHWAY - level 2: Pentose and glucuronate interconversions

               Candy accession: Q4Q252_LEIMA
              RefSeq accession: XP_001686596.1
             Uniprot accession: Q4Q252_LEIMA
                      Comments: LGT - LM ONE NODE -3 DOMAIN ToL
              Species affected: LM
         Adjacent taxa in tree: Proteobacteria
EC annotation - (Blast/Profile): EC:2.7.1.17
                   PHOBIUS SP: 0
                  PHOBIUS TMD: 0
             RefSeq annotation: xylulokinase
        Name of enzyme/protein: Xylulokinase
        KEGG PATHWAY - level 1: Carbohydrate Metabolism
        KEGG PATHWAY - level 2: Pentose and glucuronate interconversions

               Candy accession: C4LTL6_ENTHI
              RefSeq accession: XP_656185.1
             Uniprot accession: C4LTL6_ENTHI
                      Comments: LGT - EH ONE NODE - 3 DOMAIN ToL
              Species affected: EH
         Adjacent taxa in tree: Bacteria
EC annotation - (Blast/Profile): EC:2.7.1.17
                   PHOBIUS SP: 0
                  PHOBIUS TMD: 0
             RefSeq annotation: xylulose kinase
        Name of enzyme/protein: Xylulokinase
        KEGG PATHWAY - level 1: Carbohydrate Metabolism
        KEGG PATHWAY - level 2: Pentose and glucuronate interconversions
```

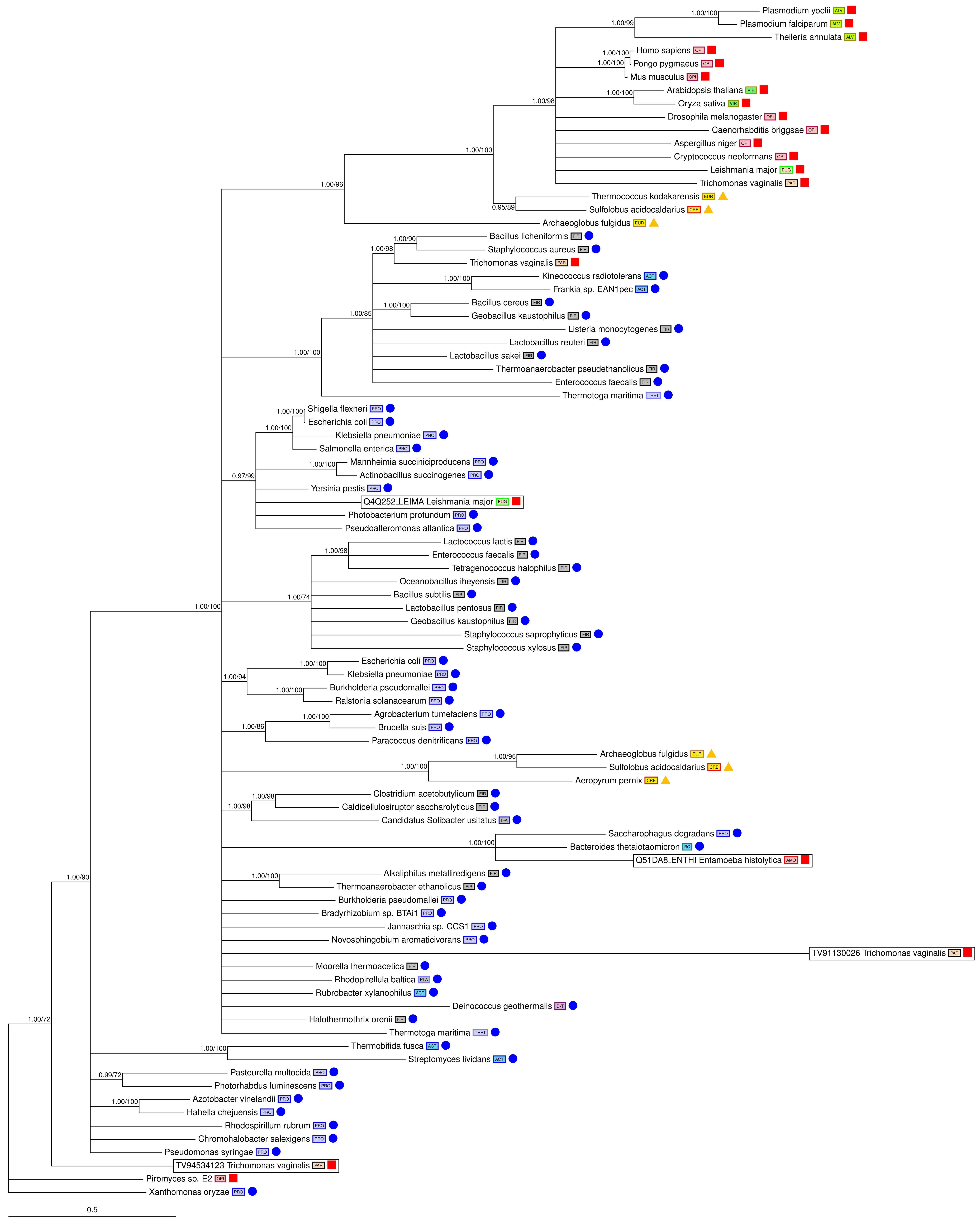

ON032

```
             Candy accession: Q8I5J3_PLAF7
            RefSeq accession: XP_001350618.1
           Uniprot accession: Q8I5J3_PLAF7
                    Comments: LGT - PF, PV ONE NODE - 3 DOMAIN ToL
            Species affected: PF,PV
       Adjacent taxa in tree: Ambiguous
EC annotation - (Blast/Profile): na
                 PHOBIUS SP: Y
                PHOBIUS TMD: 0
          RefSeq annotation: conserved protein
   Name of enzyme/protein: Uncharacterised conserved protein family
     KEGG PATHWAY - level 1: Function unknown
     KEGG PATHWAY - level 2: na

             Candy accession: Q7QTD7_GIALA
            RefSeq accession: XP_001709735.1
           Uniprot accession: A8B4Z5_GIALA
                    Comments: LGT - GI ONE NODE - ODD TAXONOMIC SAMPLE
            Species affected: GI
       Adjacent taxa in tree: Prokaryote/Phage
EC annotation - (Blast/Profile): na
                 PHOBIUS SP: 0
                PHOBIUS TMD: 0
          RefSeq annotation: RTCB PROTEIN
   Name of enzyme/protein: Uncharacterised conserved protein family
     KEGG PATHWAY - level 1: Function unknown
     KEGG PATHWAY - level 2: na
```

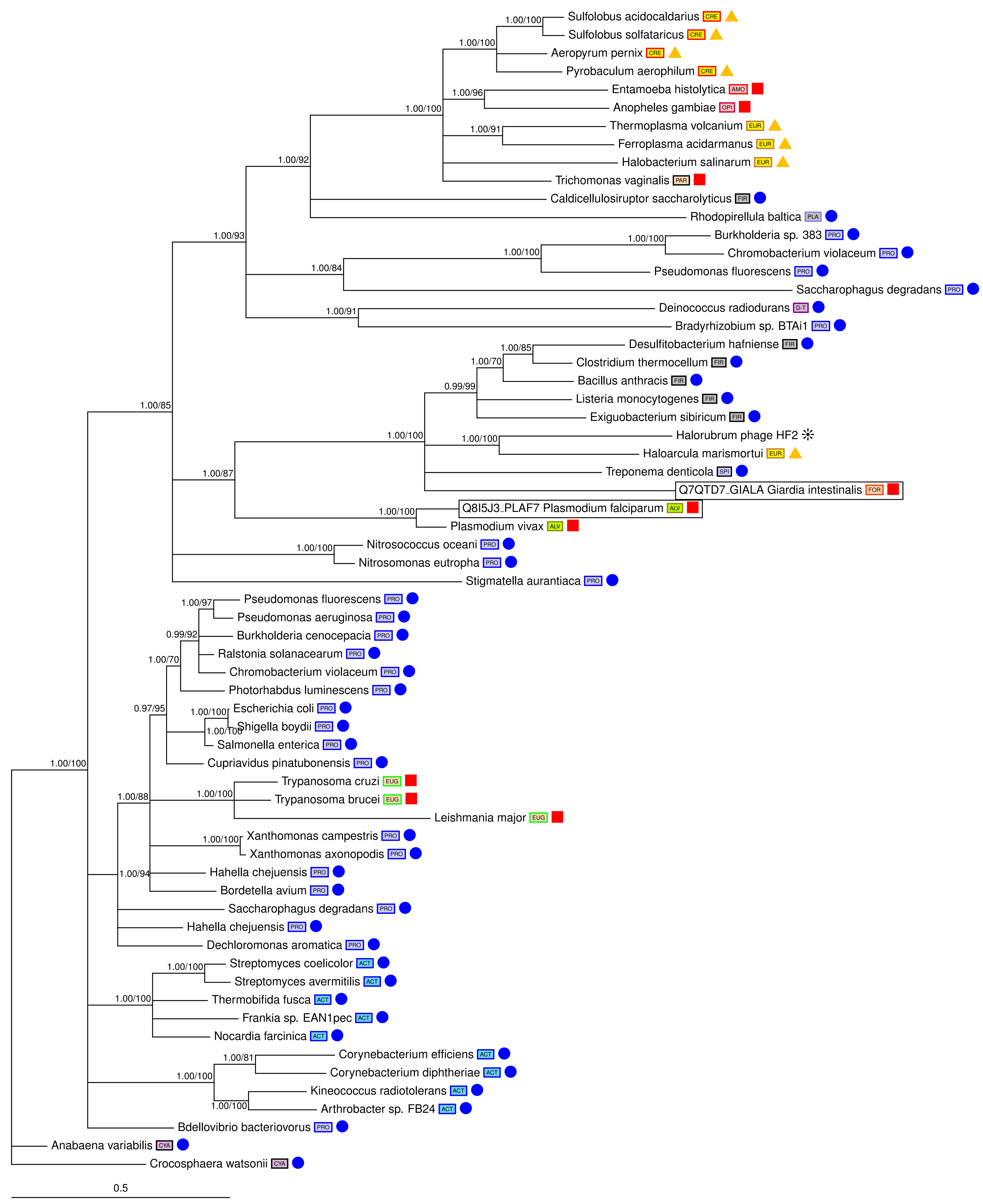

ON033

```
               Candy accession: Q25348_LEIMA
              RefSeq accession: XP_001687423.1
             Uniprot accession: E9AC20_LEIMA
                      Comments: LGT - LM ONE NODE
              Species affected: LM
         Adjacent taxa in tree: Prokaryotes
EC annotation - (Blast/Profile): EC:3.6.1.11
                    PHOBIUS SP: 0
                   PHOBIUS TMD: 0
             RefSeq annotation: acidocalcisomal exopolyphosphatase
        Name of enzyme/protein: exopolyphosphatase
        KEGG PATHWAY - level 1: Nucleotide Metabolism
        KEGG PATHWAY - level 2: Purine metabolism

               Candy accession: Q7QWT6_GIALA
              RefSeq accession: XP_001709297.1
             Uniprot accession: A8B5T0_GIALA
                      Comments: LGT - GI ONE NODE
              Species affected: GI
         Adjacent taxa in tree: Bacteria
EC annotation - (Blast/Profile): EC:3.6.1.11
                    PHOBIUS SP: 0
                   PHOBIUS TMD: 0
             RefSeq annotation: Manganese-dependent inorganic
                                pyrophosphatase, putative
        Name of enzyme/protein: exopolyphosphatase
        KEGG PATHWAY - level 1: Nucleotide Metabolism
        KEGG PATHWAY - level 2: Purine metabolism
```

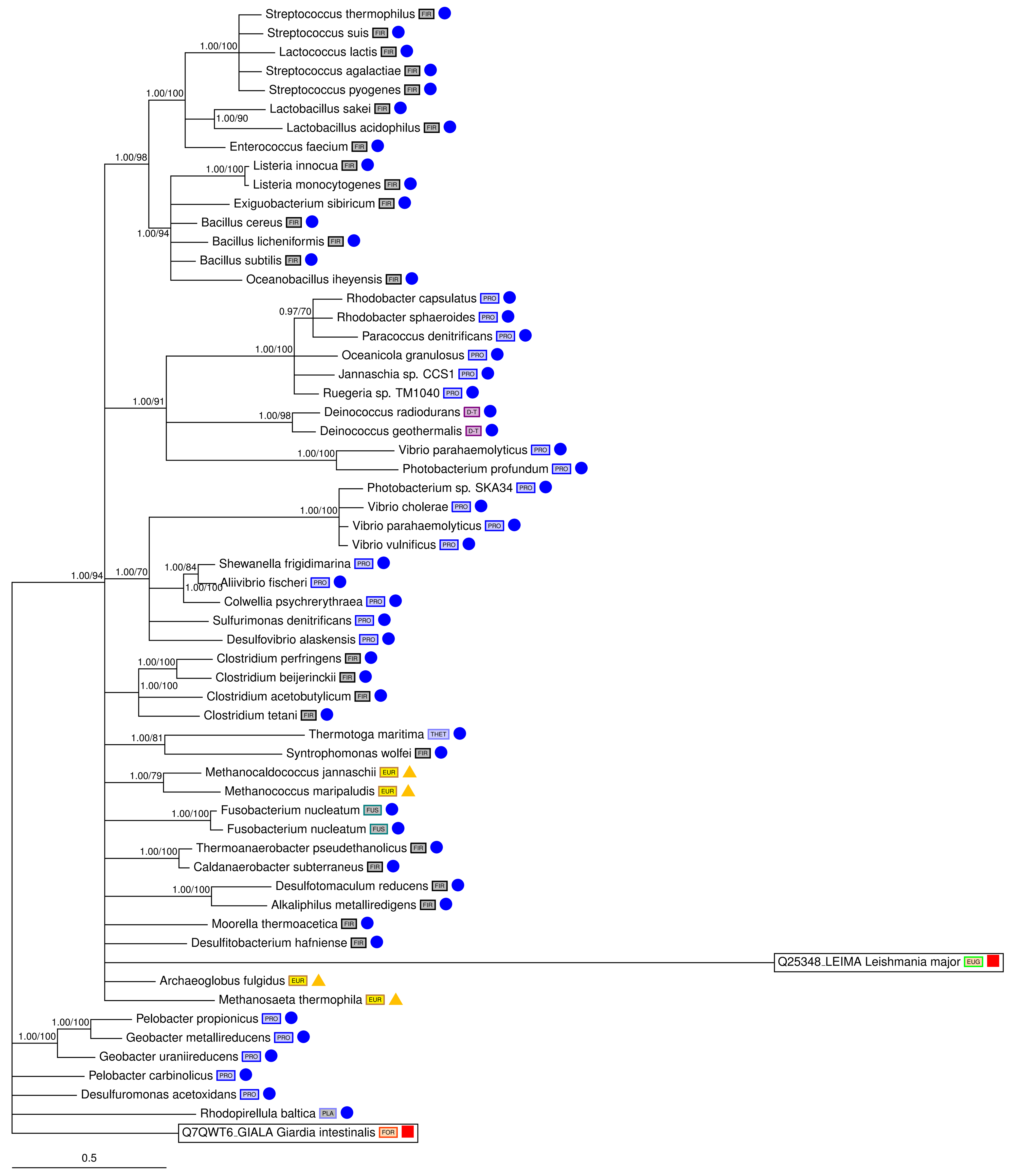

ON034

Candy accession: Q7R192_GIALA
RefSeq accession: XP_001704875.1
Uniprot accession: A8BTG2_GIALA
Comments: LGT - GI ONE NODE
Species affected: GI,TG
Adjacent taxa in tree: Bacteria
EC annotation - (Blast/Profile): na
PHOBIUS SP: 0
PHOBIUS TMD: 0
RefSeq annotation: Metalloprotease, insulinase family
Name of enzyme/protein: Predicted Zn-dependent peptidases, insulinase-like
KEGG PATHWAY - level 1: Other function
KEGG PATHWAY - level 2: na

Candy accession: Q7QYM9_GIALA
RefSeq accession: XP_001705918.1
Uniprot accession: A8BNI1_GIALA
Comments: LGT - GI ONE NODE
Species affected: GI,TG
Adjacent taxa in tree: Bacteria
EC annotation - (Blast/Profile): na
PHOBIUS SP: 0
PHOBIUS TMD: 0
RefSeq annotation: Metalloprotease, insulinase family
Name of enzyme/protein: Predicted Zn-dependent peptidases, insulinase-like
KEGG PATHWAY - level 1: Other function
KEGG PATHWAY - level 2: na

Candy accession: T42_03594
RefSeq accession: XP_002366418.1
Uniprot accession: B6KEZ5_TOXGO
Comments: LGT - TG ONE NODE
Species affected: TG
Adjacent taxa in tree: Proteobacteria
EC annotation - (Blast/Profile): na
PHOBIUS SP: Y
PHOBIUS TMD: 0
RefSeq annotation: zinc metalloprotease 2, putative
Name of enzyme/protein: Predicted Zn-dependent peptidases, insulinase-like
KEGG PATHWAY - level 1: Other function
KEGG PATHWAY - level 2: na

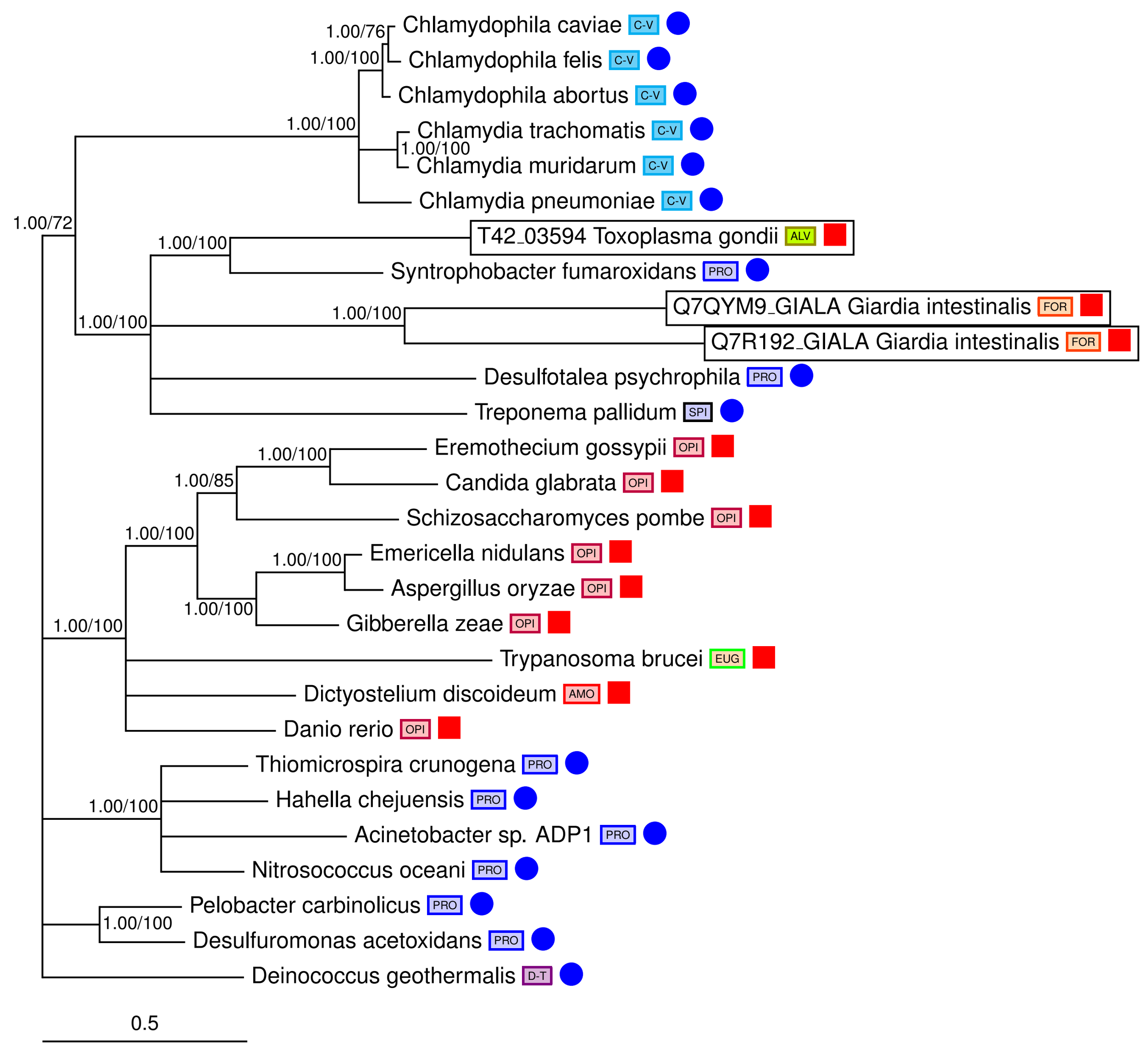

ON035

```
               Candy accession: Q7R6R6_GIALA
              RefSeq accession: XP_001707847.1
             Uniprot accession: A8BC83_GIAIC
                      Comments: LGT - GI ONE NODE
              Species affected: GI
          Adjacent taxa in tree: Bacteria
EC annotation - (Blast/Profile): na
                    PHOBIUS SP: 0
                   PHOBIUS TMD: 0
             RefSeq annotation: ATPases of the PP-loop superfamily
         Name of enzyme/protein: Predicted Adenine nucleotide alpha
                                hydrolase/ATPases of the PP-loop
                                superfamily
         KEGG PATHWAY - level 1: Other function
         KEGG PATHWAY - level 2: na
```

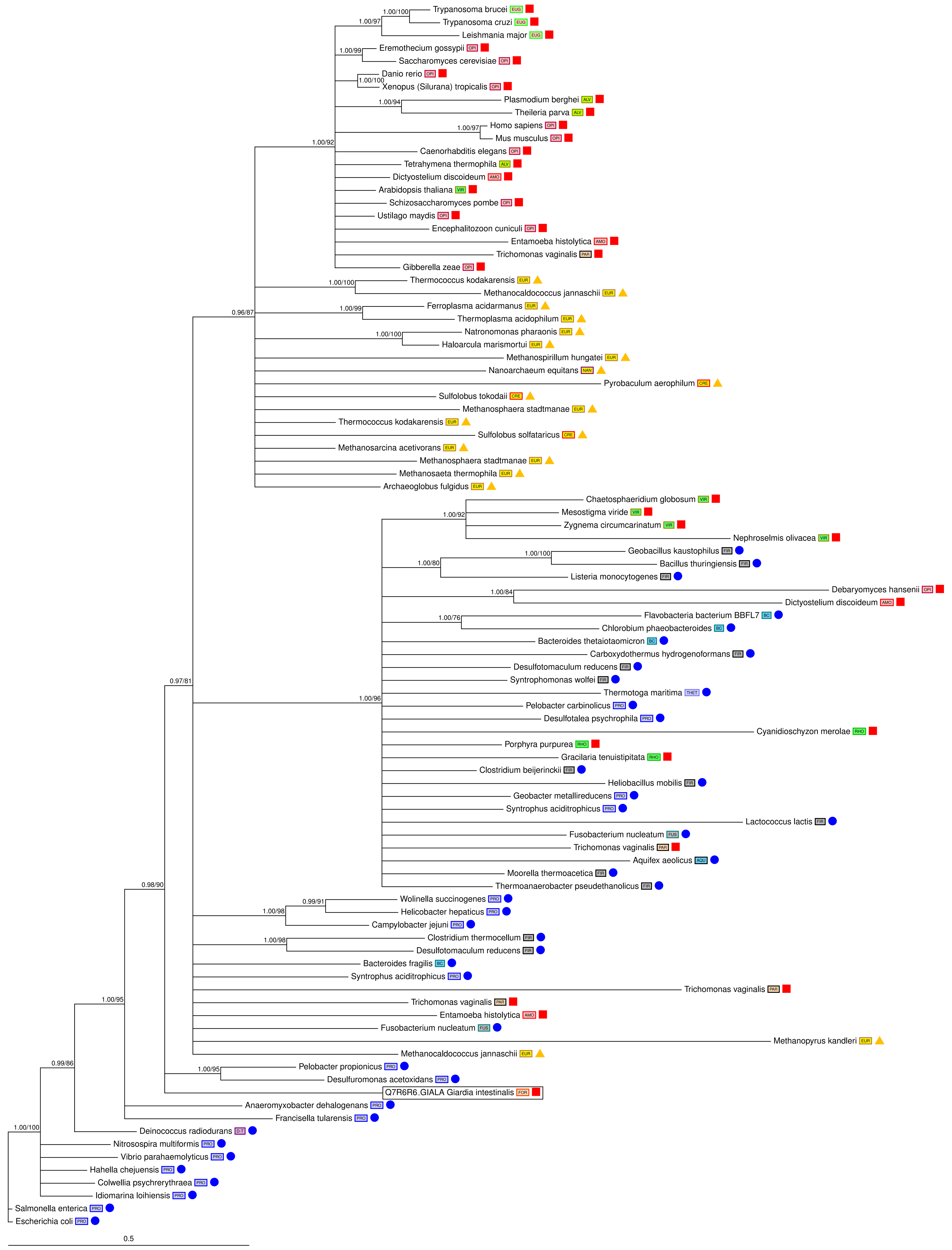

ON036

```
                 Candy accession: Q7R381_GIALA
                RefSeq accession: XP_001709207.1
               Uniprot accession: A8B5E9_GIAIC
                        Comments: LGT - GI ONE NODE
                Species affected: GI
            Adjacent taxa in tree: Prokaryotes
EC annotation - (Blast/Profile): na
                      PHOBIUS SP: 0
                     PHOBIUS TMD: 0
               RefSeq annotation: Hypothetical protein GL50803_3910
         Name of enzyme/protein: Protein containing thioredoxin domain
         KEGG PATHWAY - level 1: Other function
         KEGG PATHWAY - level 2: na
```

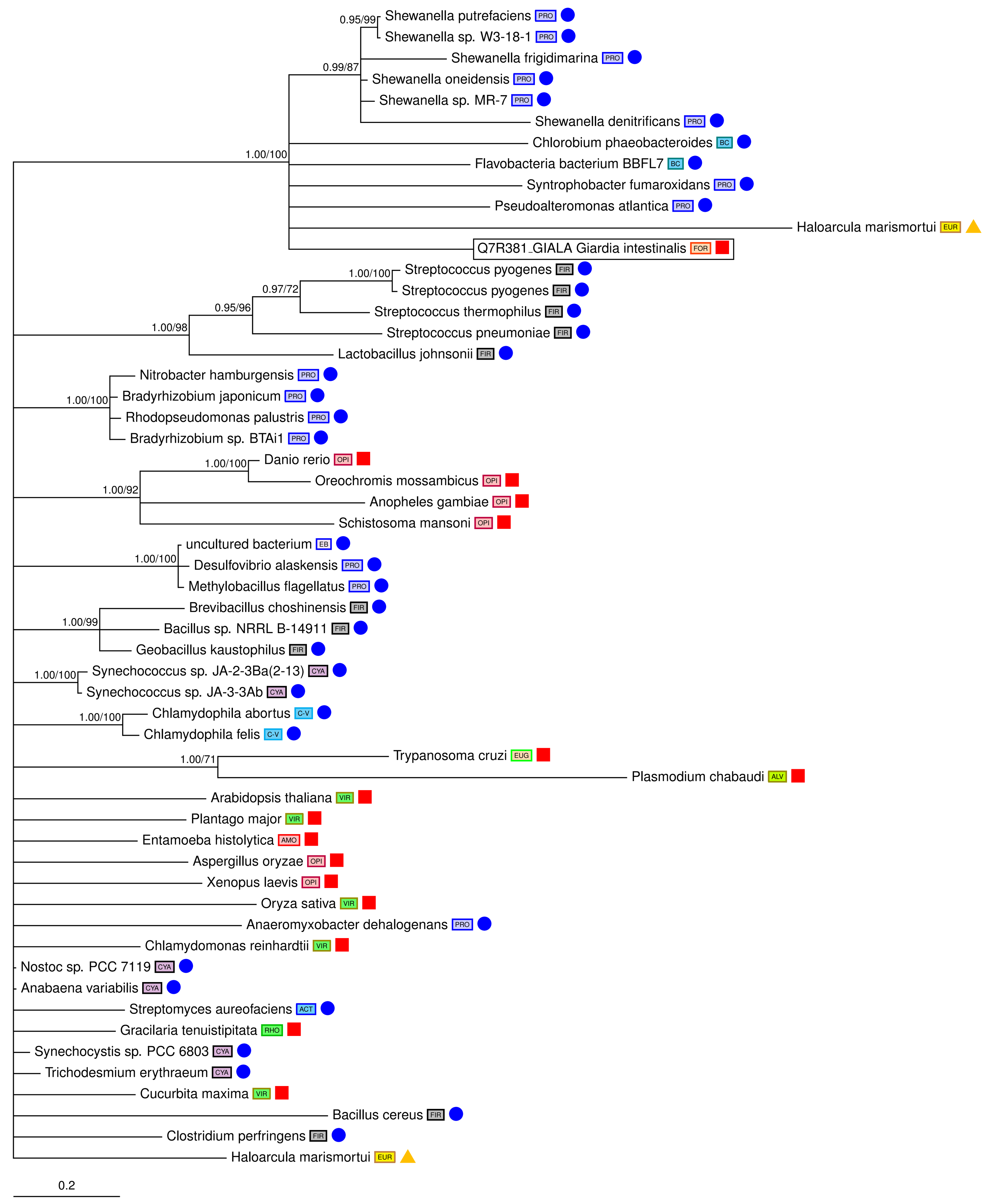

ON037

```
                Candy accession: Q7R548_GIALA
               RefSeq accession: XP_001707737.1
              Uniprot accession: A8BD82_GIALA
                       Comments: LGT - GI ONE NODE
               Species affected: GI
         Adjacent taxa in tree: Pelobacter
EC annotation - (Blast/Profile): EC:2.7.8.8
                     PHOBIUS SP: 0
                    PHOBIUS TMD: 15
              RefSeq annotation: Hypothetical protein GL50803_17427
          Name of enzyme/protein: CDP-diacylglycerol-serine
                                 O-phosphatidyltransferase
         KEGG PATHWAY - level 1: Amino Acid Metabolism, Lipid Metabolism
         KEGG PATHWAY - level 2: Glycine, serine and threonine
                                 metabolism, Glycerophospholipid
                                 metabolism
```

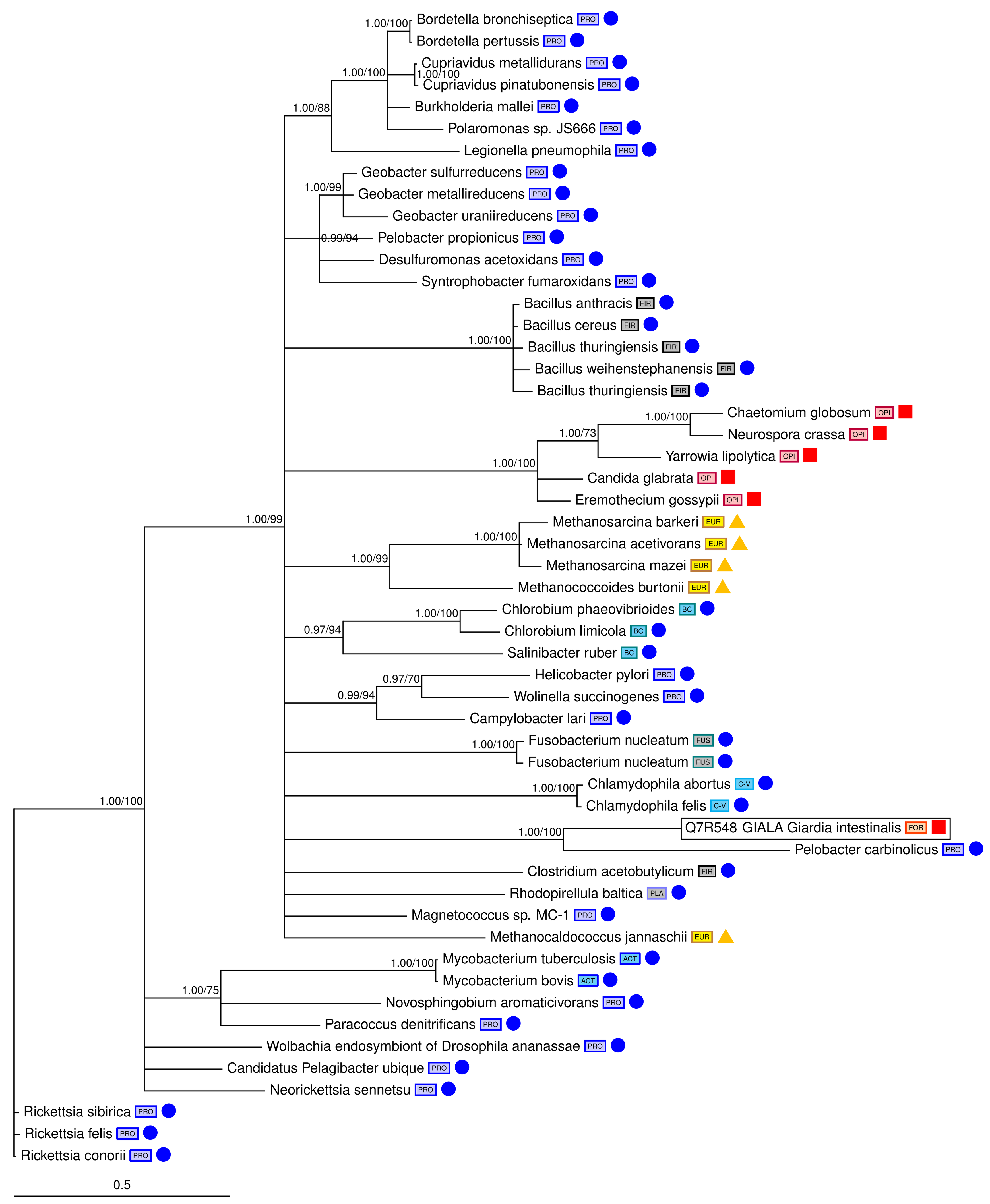

ON038

```
                 Candy accession: Q7RGW5_PLAYO
                RefSeq accession: XP_724496.1
               Uniprot accession: Q7RGW5_PLAYO
                        Comments: LGT - APICOMPLEXA ONE NODE
                Species affected: PF,PV,PY
           Adjacent taxa in tree: Bacteria
EC annotation - (Blast/Profile): na
                      PHOBIUS SP: 0
                     PHOBIUS TMD: 0
               RefSeq annotation: hypothetical protein
         Name of enzyme/protein: Predicted flavin mononucleotide
                                 phosphatase, haloacid dehalogenase
         KEGG PATHWAY - level 1: Other function
         KEGG PATHWAY - level 2: na

                 Candy accession: PV091555
                RefSeq accession: XP_001615302.1
               Uniprot accession: A5K4J7_PLAVS
                        Comments: LGT - APICOMPLEXA ONE NODE
                Species affected: PF,PV,PY
           Adjacent taxa in tree: Bacteria
EC annotation - (Blast/Profile): EC:3.8.1.2
                      PHOBIUS SP: 0
                     PHOBIUS TMD: 0
               RefSeq annotation: haloacid dehalogenase-like hydrolase
         Name of enzyme/protein: (S)-2-haloacid dehalogenase
         KEGG PATHWAY - level 1: Xenobiotics Biodegradation and
                                 Metabolism
         KEGG PATHWAY - level 2: Chlorocyclohexane and chlorobenzene
                                 degradation, Chloroalkane and
                                 chloroalkene degradation
```

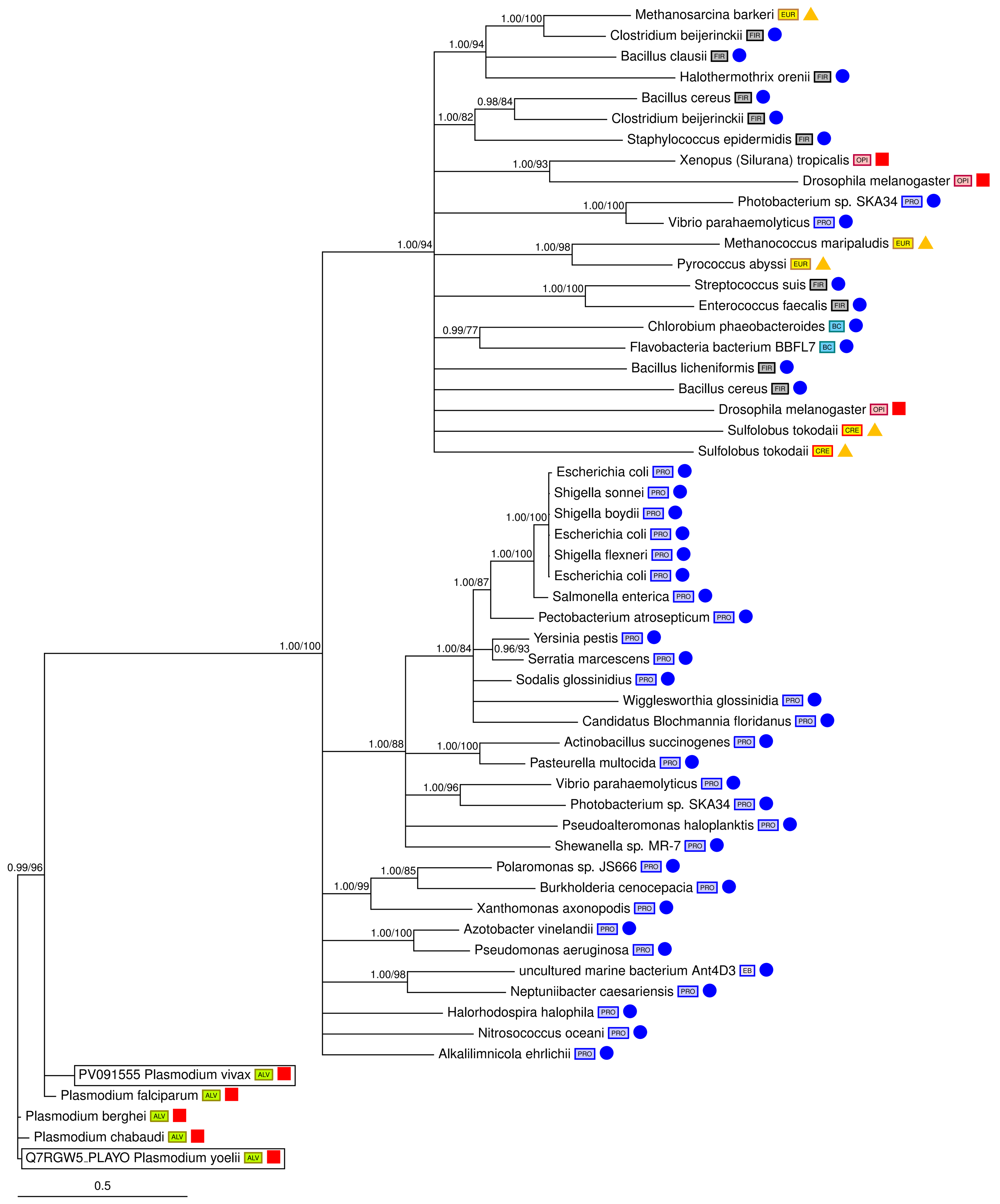

ON039

Candy accession: Q50S10_ENTHI
RefSeq accession: XP_649756.2
Uniprot accession: C4M213_ENTHI
Comments: LGT - EH ONE NODE
Species affected: EH
Adjacent taxa in tree: Prokaryotes
EC annotation - (Blast/Profile): EC:1.1.1.37
PHOBIUS SP: 0
PHOBIUS TMD: 0
RefSeq annotation: malate dehydrogenase
Name of enzyme/protein: Malate dehydrogenase
KEGG PATHWAY - level 1: Carbohydrate Metabolism
KEGG PATHWAY - level 2: Citrate cycle (TCA cycle), Pyruvate metabolism, Glyoxylate and dicarboxylate metabolism

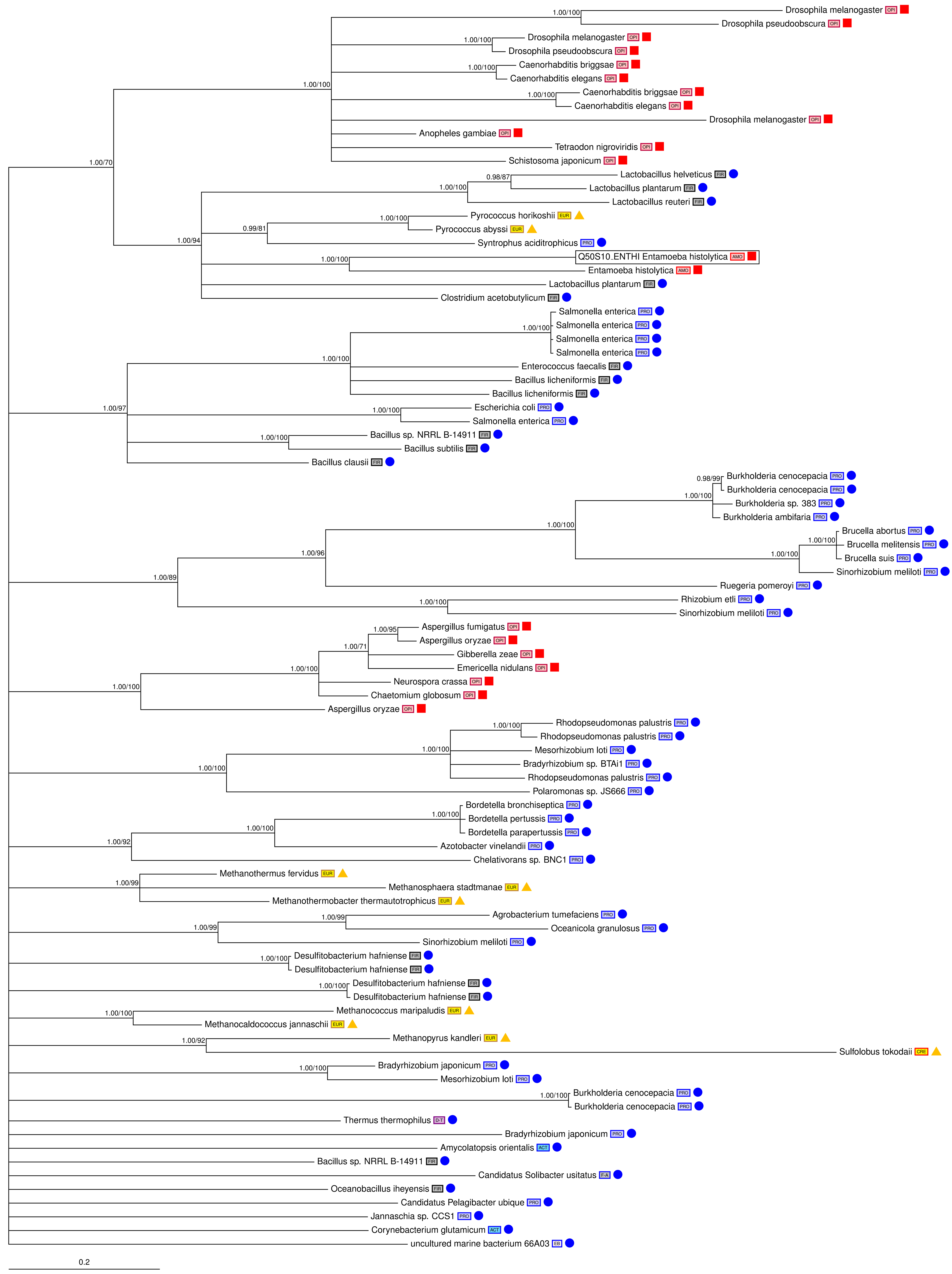

ON040

```
              Candy accession: TV81660102
             RefSeq accession: XP_001317174.1
            Uniprot accession: A2EQX7_TRIVA
                     Comments: LGT - TV ONE NODE
             Species affected: TV
         Adjacent taxa in tree: Prokaryotes
EC annotation - (Blast/Profile): na
                   PHOBIUS SP: 0
                  PHOBIUS TMD: 0
            RefSeq annotation: hypothetical protein
       Name of enzyme/protein: Predicted multimeric flavodoxin WrbA,
                               NADPH-dependent FMN reductase
       KEGG PATHWAY - level 1: Other function
       KEGG PATHWAY - level 2: na

              Candy accession: Q51A69_ENTHI
             RefSeq accession: XP_655130.1
            Uniprot accession: C4M3J0_ENTHI
                     Comments: LGT - EH ONE NODE
             Species affected: EH
         Adjacent taxa in tree: Prokaryotes
EC annotation - (Blast/Profile): na
                   PHOBIUS SP: 0
                  PHOBIUS TMD: 0
            RefSeq annotation: iron-sulfur flavoprotein
       Name of enzyme/protein: Predicted multimeric flavodoxin WrbA,
                               NADPH-dependent FMN reductase
       KEGG PATHWAY - level 1: Other function
       KEGG PATHWAY - level 2: na
```

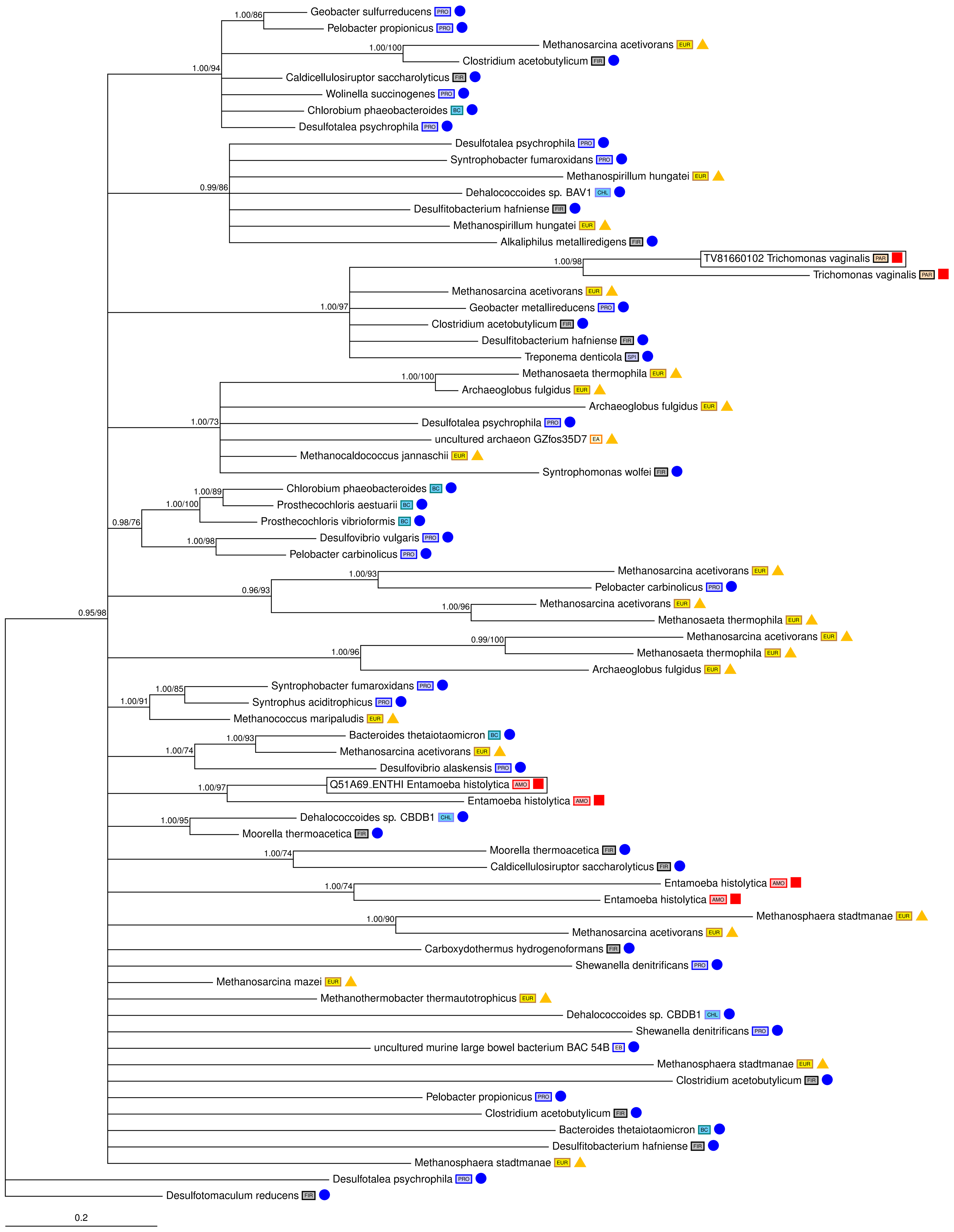

ON041

```
              Candy accession: TV85337242
             RefSeq accession: XP_001329114.1
            Uniprot accession: A2DRU4_TRIVA
                     Comments: LGT - TV ONE NODE + FUNGI ONE NODE
             Species affected: TV
        Adjacent taxa in tree: Planctomycetes - Rhodopirellula
EC annotation - (Blast/Profile): EC:3.1.-.-
                  PHOBIUS SP: 0
                 PHOBIUS TMD: 0
            RefSeq annotation: Sulphohydrolase/Glycosulfatase, Zn-
                               dependent hydrolase
       Name of enzyme/protein: Protein containing beta-lactamase
                               superfamily domain
       KEGG PATHWAY - level 1: Reaction
       KEGG PATHWAY - level 2: Reaction

              Candy accession: Q51CL3_ENTHI
             RefSeq accession: XP_656005.1
            Uniprot accession: C4M1A9_ENTHI
                     Comments: LGT - EH ONE NODE + FUNGI ONE NODE
             Species affected: EH
        Adjacent taxa in tree: Prokaryotes
EC annotation - (Blast/Profile): EC:3.1.-.-
                  PHOBIUS SP: 0
                 PHOBIUS TMD: 0
            RefSeq annotation: metallo-beta-lactamase family protein
       Name of enzyme/protein: Protein containing beta-lactamase
                               superfamily domain
       KEGG PATHWAY - level 1: Reaction
       KEGG PATHWAY - level 2: Reaction
```

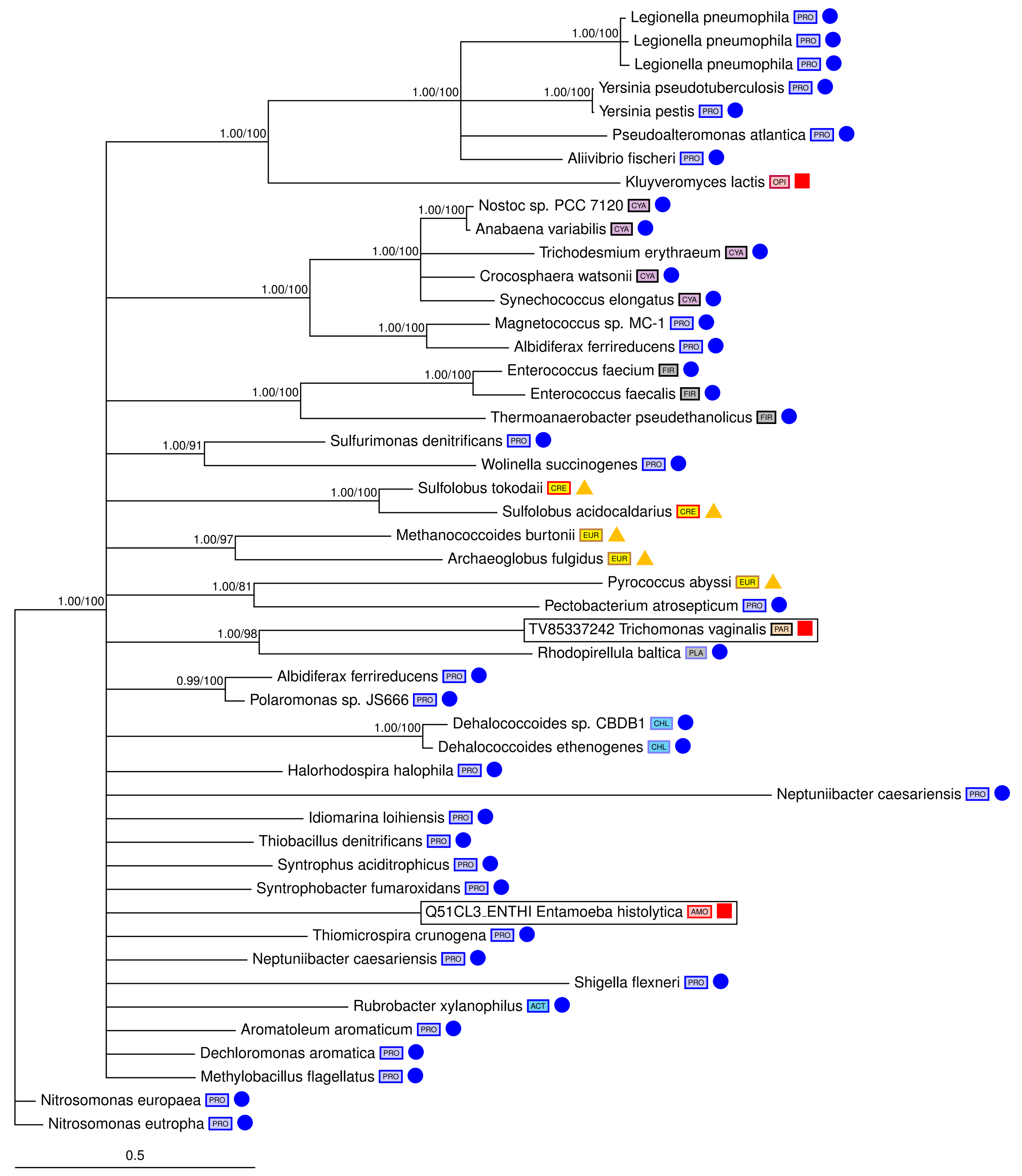

ON042

```
               Candy accession: Q54FP6_DICDI
              RefSeq accession: XP_635590.1
             Uniprot accession: PKS31_DICDI
                      Comments: LGT - DD ONE NODE
              Species affected: DD
          Adjacent taxa in tree: Proteobacteria - Legionella
EC annotation - (Blast/Profile): EC:2.3.1.-
                    PHOBIUS SP: 0
                   PHOBIUS TMD: 0
             RefSeq annotation: hypothetical protein DDB_G0290703
        Name of enzyme/protein: Transferases
       KEGG PATHWAY - level 1: Reaction
       KEGG PATHWAY - level 2: Reaction
```

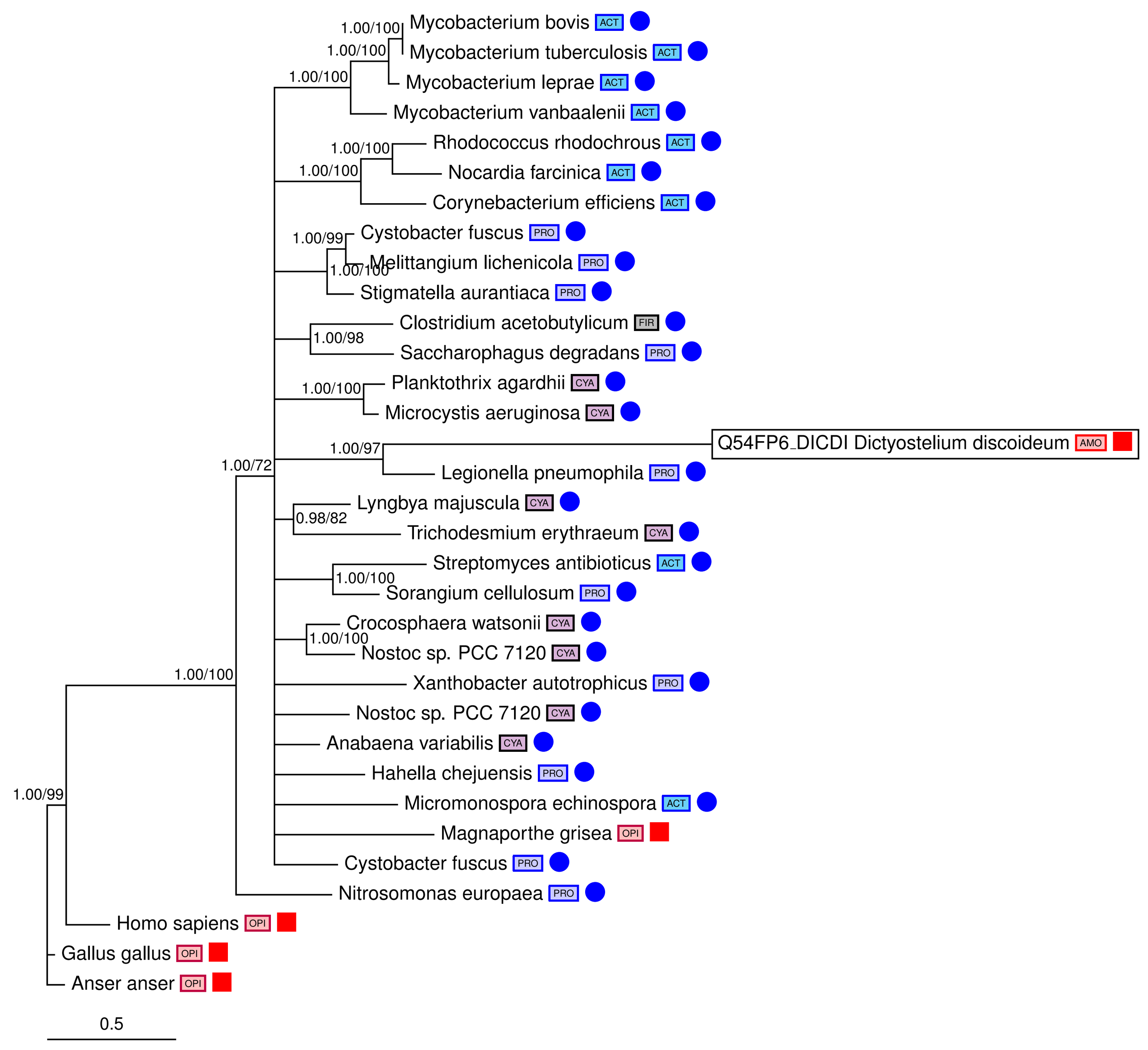

ON043

```
                Candy accession: Q54KK2_DICDI
               RefSeq accession: XP_637221.1
              Uniprot accession: Q54KK2_DICDI
                       Comments: LGT - DD ONE NODES + LGT  YL FUNGI TWO
                                 NODES
               Species affected: DD,FUNGI
          Adjacent taxa in tree: Prokaryotes
EC annotation - (Blast/Profile): na
                     PHOBIUS SP: 0
                    PHOBIUS TMD: 12
              RefSeq annotation: hypothetical protein DDB_G0287303
        Name of enzyme/protein: Predicted cationic amino acid transport
                                 permease
         KEGG PATHWAY - level 1: Other function - Membrane transport
         KEGG PATHWAY - level 2: na
```

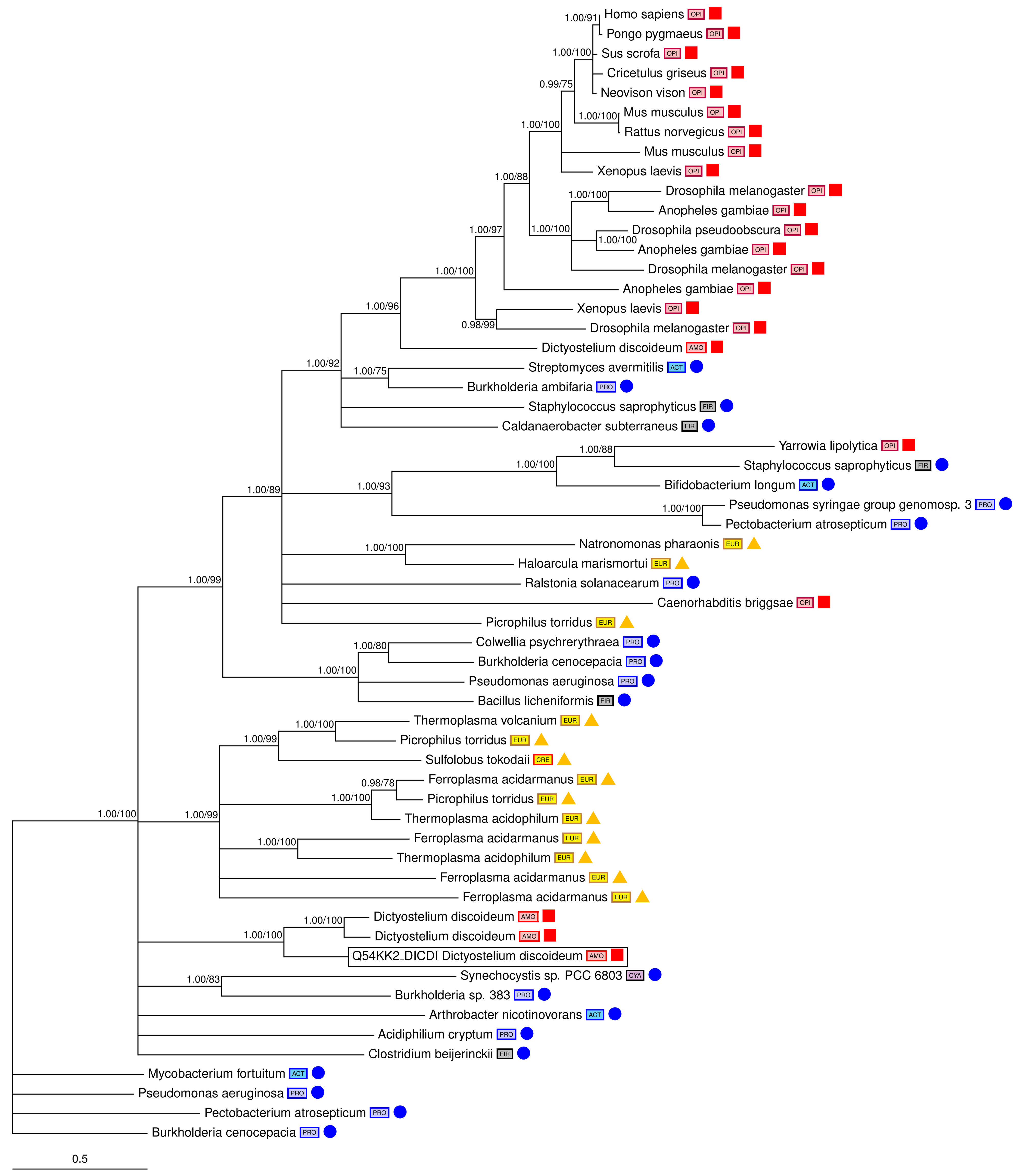

ON044

```
             Candy accession: Q54LR7_DICDI
            RefSeq accession: XP_637763.1
           Uniprot accession: Q54LR7_DICDI
                    Comments: LGT - DD ONE NODE
            Species affected: DD
       Adjacent taxa in tree: Tenericute
EC annotation - (Blast/Profile): na
                  PHOBIUS SP: 0
                 PHOBIUS TMD: 0
           RefSeq annotation: hypothetical protein DDB_G0286443
      Name of enzyme/protein: Protein containing  Type 1 glutamine
                              amidotransferase (GATase1)-like domain
      KEGG PATHWAY - level 1: Other function
      KEGG PATHWAY - level 2: na
```

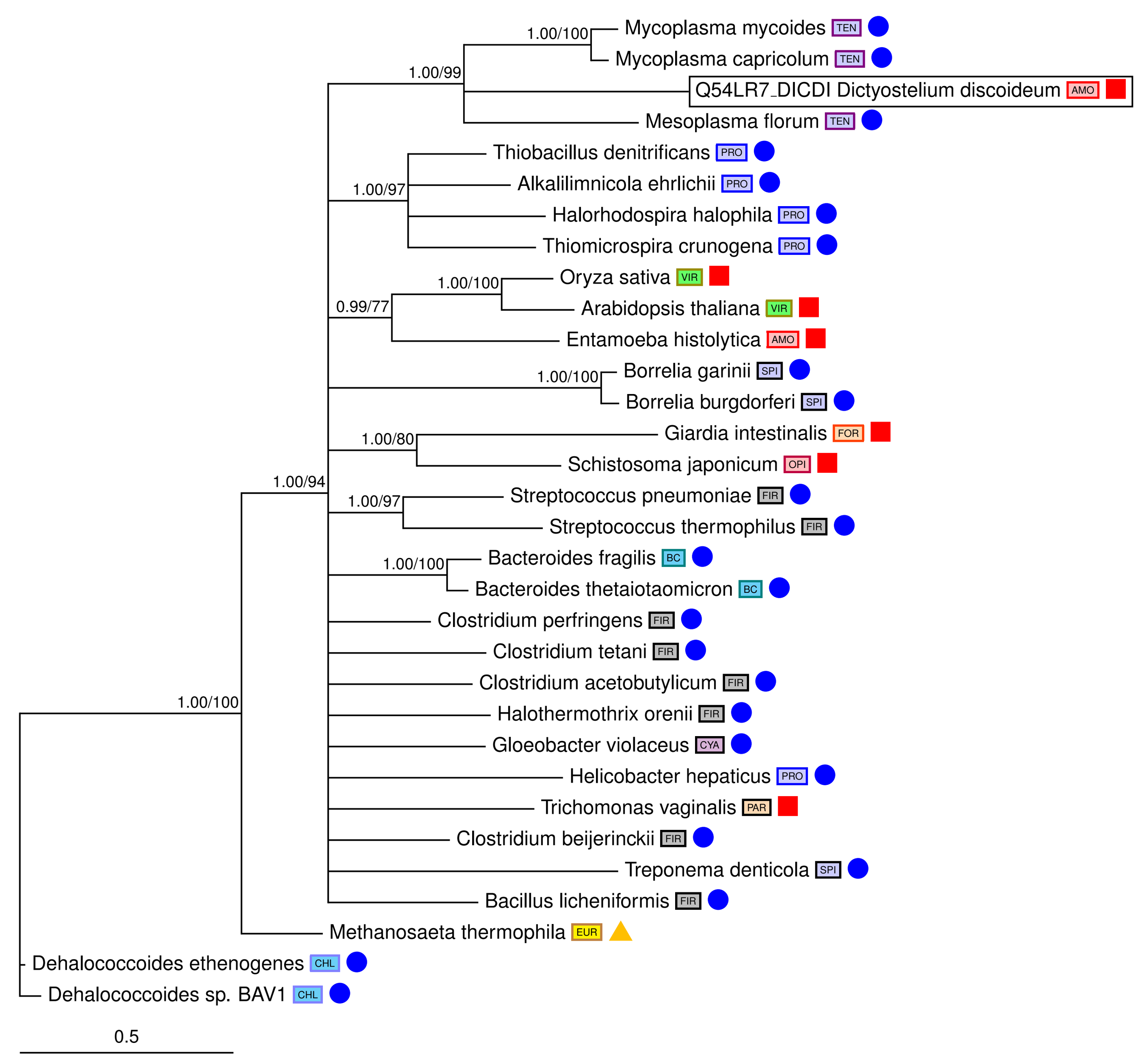

ON045

```
           Candy accession: TV95721149
          RefSeq accession: XP_001323199.1
         Uniprot accession: A2E8N7_TRIVA
                  Comments: LGT - TV ONE NODE
          Species affected: TV
      Adjacent taxa in tree: Bacteroides
EC annotation - (Blast/Profile): EC:1.1.1.-
                 PHOBIUS SP: Y
                PHOBIUS TMD: 0
          RefSeq annotation: oxidoreductase, short chain
                             dehydrogenase/reductase family
      Name of enzyme/protein: Oxidoreductases
     KEGG PATHWAY - level 1: Reaction
     KEGG PATHWAY - level 2: Reaction

           Candy accession: Q54PT0_DICDI
          RefSeq accession: XP_638574.1
         Uniprot accession: Q54PT0_DICDI
                  Comments: LGT - DD ONE NODE
          Species affected: DD
      Adjacent taxa in tree: Bacteria
EC annotation - (Blast/Profile): EC:1.1.1.-
                 PHOBIUS SP: Y
                PHOBIUS TMD: 0
          RefSeq annotation: hypothetical protein DDB_G0284351
      Name of enzyme/protein: Oxidoreductases
     KEGG PATHWAY - level 1: Reaction
     KEGG PATHWAY - level 2: Reaction

           Candy accession: Q54D03_DICDI
          RefSeq accession: XP_629517.1
         Uniprot accession: 54D03_DICDI
                  Comments: LGT - DD ONE NODE
          Species affected: DD
      Adjacent taxa in tree: Bacteria
EC annotation - (Blast/Profile): EC:1.1.1.-
                 PHOBIUS SP: 0
                PHOBIUS TMD: 0
          RefSeq annotation: hypothetical protein DDB_G0292602
      Name of enzyme/protein: Oxidoreductases
     KEGG PATHWAY - level 1: Reaction
     KEGG PATHWAY - level 2: Reaction
```

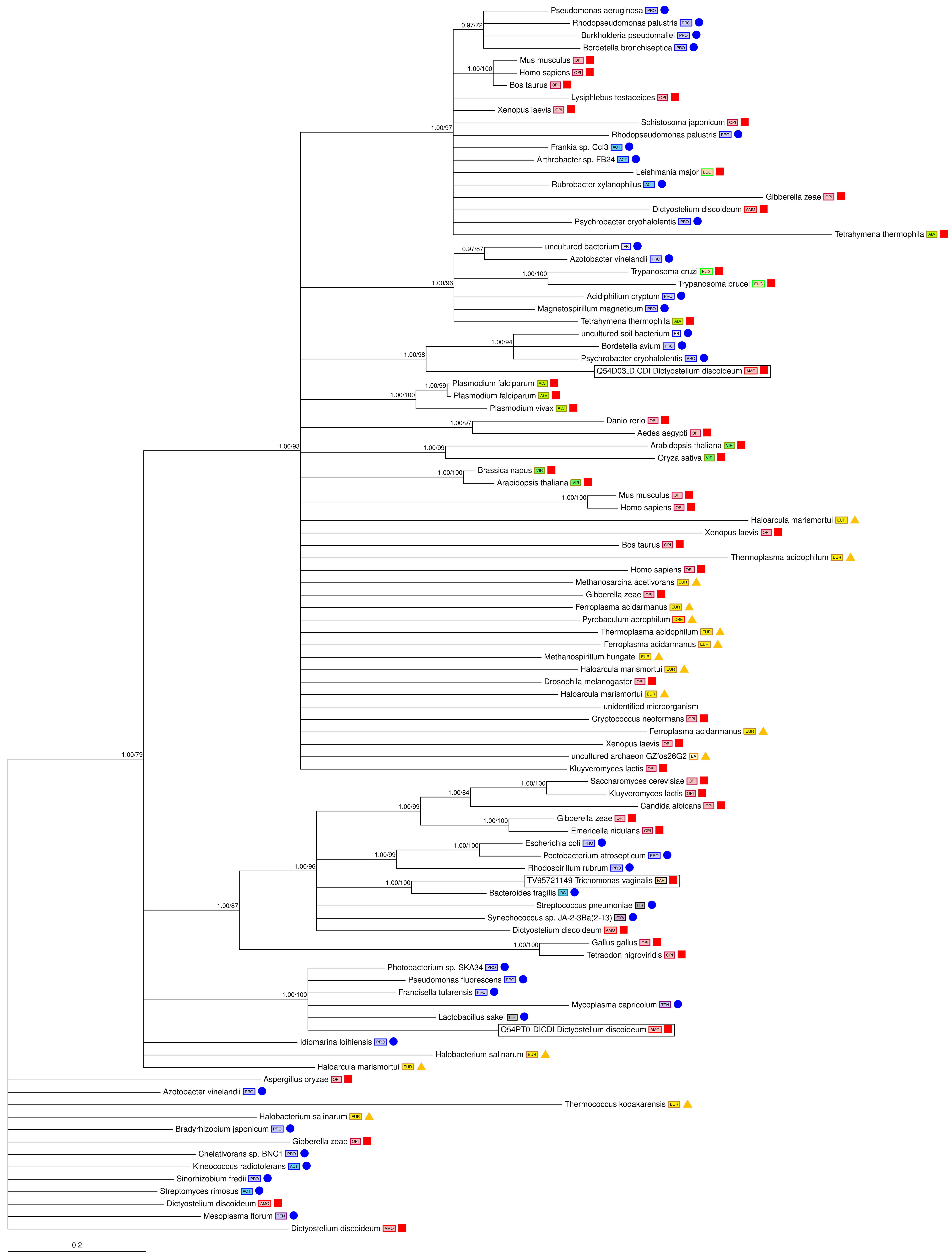

ON046

```
               Candy accession: Q54RA4_DICDI
              RefSeq accession: XP_639143.1
             Uniprot accession: Y3291_DICDI
                      Comments: LGT - DD ONE NODE
              Species affected: DD
        Adjacent taxa in tree: Bacteria
EC annotation - (Blast/Profile): EC:1.21.3.1
                    PHOBIUS SP: 0
                   PHOBIUS TMD: 0
             RefSeq annotation: hypothetical protein DDB_G0283291
       Name of enzyme/protein: isopenicillin-N synthase
        KEGG PATHWAY - level 1: Biosynthesis of Other Secondary
                                Metabolites
        KEGG PATHWAY - level 2: Penicillin and cephalosporin
                                biosynthesis

               Candy accession: Q4CQK0_TRYCR
              RefSeq accession: XP_804402.1
             Uniprot accession: Q4CQK0_TRYCR
                      Comments: LGT - KINETOPLASTIDS ONE NODE
              Species affected: TC,TB,LM
        Adjacent taxa in tree: Pseudomonas
EC annotation - (Blast/Profile): EC:1.2.3.4
                    PHOBIUS SP: 0
                   PHOBIUS TMD: 0
             RefSeq annotation: oxidoreductase
       Name of enzyme/protein: oxalate oxidase
        KEGG PATHWAY - level 1: Carbohydrate Metabolism
        KEGG PATHWAY - level 2: Glyoxylate and dicarboxylate metabolism
```

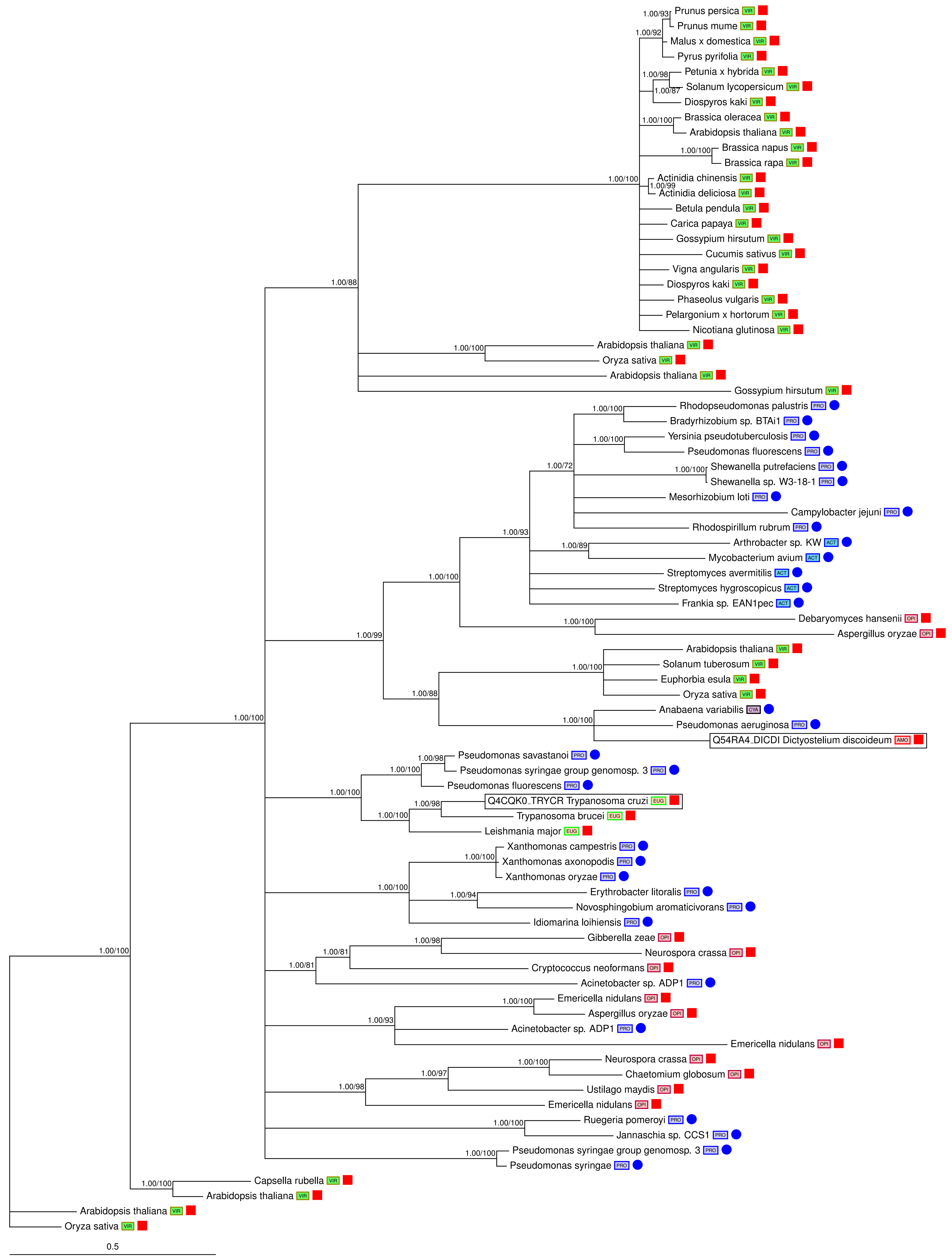

ON047

```
            Candy accession: Q55CR1_DICDI
           RefSeq accession: XP_646420.1
          Uniprot accession: Q55CR1_DICDI
                   Comments: LGT - DD ONE NODE - 3 DOMAIN
           Species affected: DD
       Adjacent taxa in tree: Prokaryote
EC annotation - (Blast/Profile): na
                 PHOBIUS SP: 0
                PHOBIUS TMD: 1
          RefSeq annotation: hypothetical protein DDB_G0270694
 Name of enzyme/protein: Predicted subgroup of the band 7 domain
                         of flotillin (reggie) like proteins
   KEGG PATHWAY - level 1: Other function
   KEGG PATHWAY - level 2: na
```

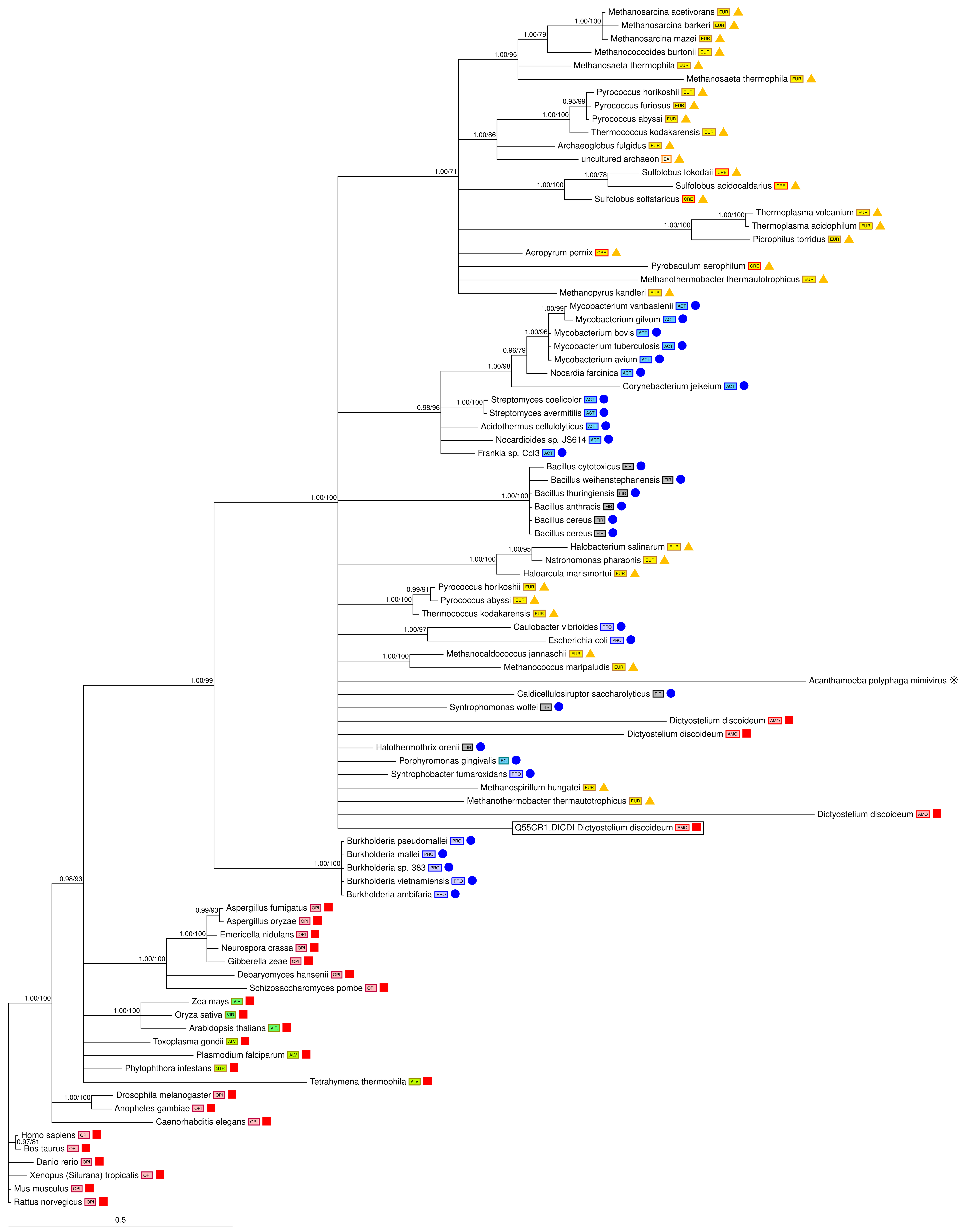

ON048

```
                Candy accession: Q50SU3_ENTHI
               RefSeq accession: XP_650055.1
              Uniprot accession: Q401L6_ENTHI
                       Comments: LGT - EH ONE NODE + FUNGI ONE NODE
               Species affected: EH
          Adjacent taxa in tree: Firmicutes - Clostridium
EC annotation - (Blast/Profile): EC:2.7.1.31
                     PHOBIUS SP: 0
                    PHOBIUS TMD: 0
              RefSeq annotation: glycerate kinase
         Name of enzyme/protein: Glycerate kinase
         KEGG PATHWAY - level 1: Amino Acid Metabolism, Lipid Metabolism,
                                 Carbohydrate Metabolism
         KEGG PATHWAY - level 2: Glycine, serine and threonine
                                 metabolism, Glycerophospholipid
                                 metabolism, Glyoxylate and dicarboxylate
                                 metabolism
```

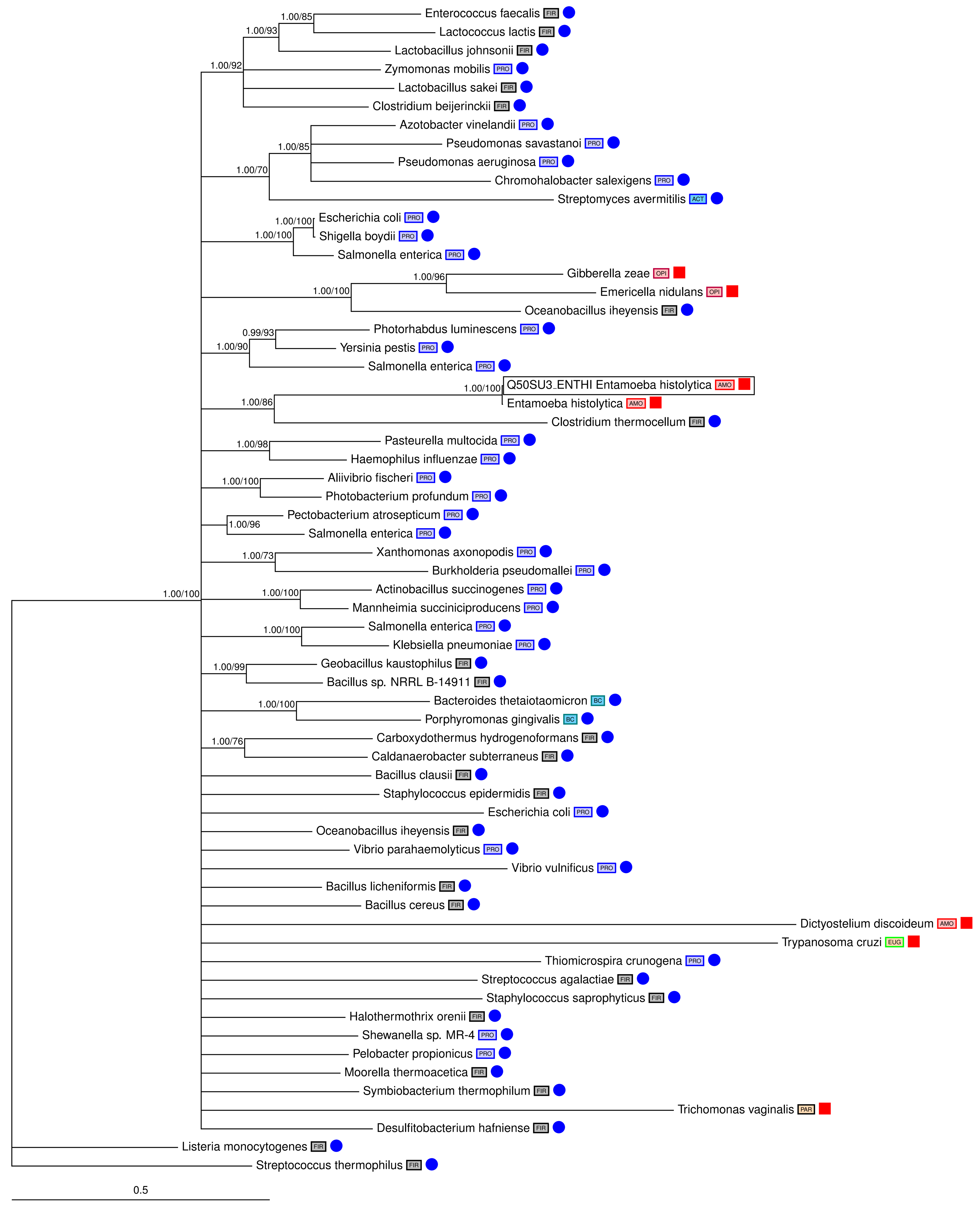

ON049

```
               Candy accession: Q86HZ8_DICDI
              RefSeq accession: XP_644821.1
             Uniprot accession: Q557D9_DICDI
                      Comments: LGT - DD ONE NODE
              Species affected: DD
         Adjacent taxa in tree: Bacteria
EC annotation - (Blast/Profile): EC:2.1.1.34
                    PHOBIUS SP: 0
                   PHOBIUS TMD: 0
             RefSeq annotation: hypothetical protein DDB_G0273135
        Name of enzyme/protein: tRNA
                                (guanosine18-2’-O)-methyltransferase
        KEGG PATHWAY - level 1: Translation - Genetic Information
                                Processing
        KEGG PATHWAY - level 2: Transfer RNA biogenesis
```

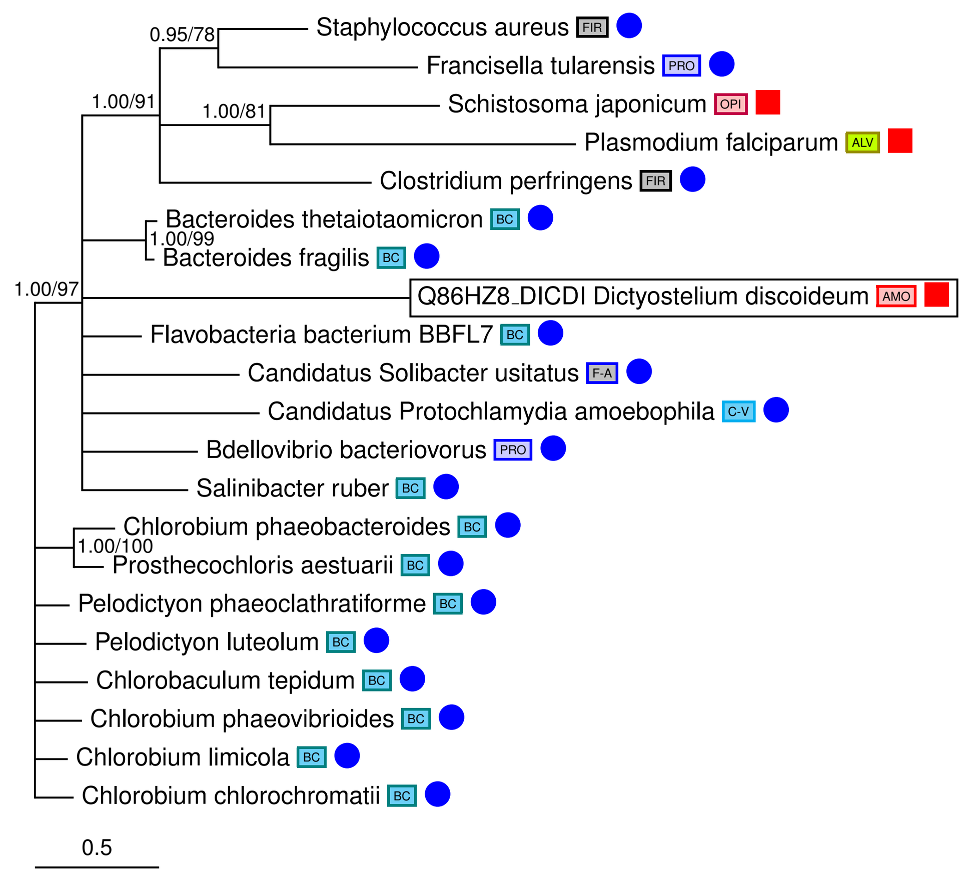

ON050

```
               Candy accession: Q584R7_9TRYP
              RefSeq accession: XP_845360.1
             Uniprot accession: Q584R7_9TRYP
                      Comments: LGT - ONE NODE TB,TC,LM
              Species affected: LM,TB,TC
         Adjacent taxa in tree: Actinobacteria
EC annotation - (Blast/Profile): na
                   PHOBIUS SP: 0
                  PHOBIUS TMD: 0
             RefSeq annotation: hypothetical protein
        Name of enzyme/protein: Hypothetical proteins
        KEGG PATHWAY - level 1: Function unknown
        KEGG PATHWAY - level 2: na
```

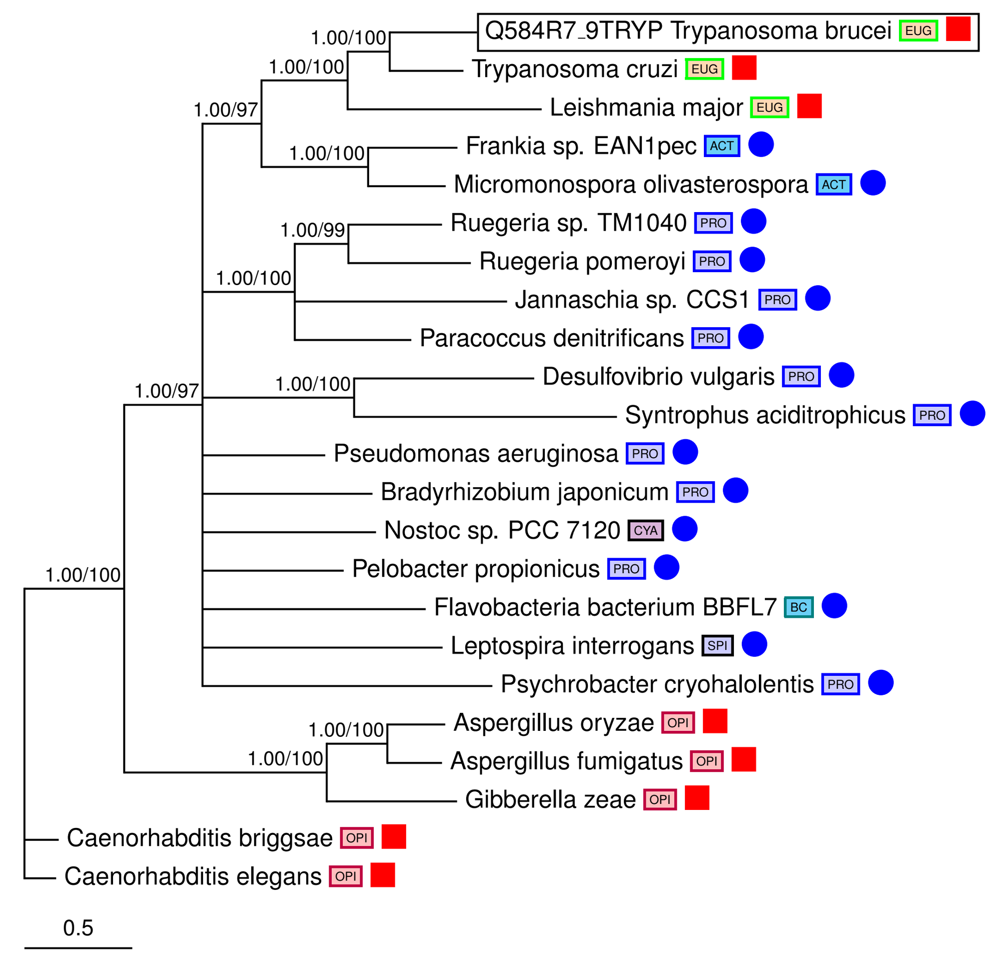

ON051

```
                Candy accession: T50_00005
               RefSeq accession: XP_002367118.1
              Uniprot accession: B6KGZ5_TOXGO
                       Comments: LGT - TG ONE NODE -3 DOMAIN ToL
               Species affected: TG
           Adjacent taxa in tree: Bacteria
EC annotation - (Blast/Profile): EC:3.1.3.11
                     PHOBIUS SP: 0
                    PHOBIUS TMD: 0
              RefSeq annotation: fructose-1,6-bisphosphatase, putative
          Name of enzyme/protein: fructose-bisphosphatase
          KEGG PATHWAY - level 1: Carbohydrate Metabolism
          KEGG PATHWAY - level 2: Glycolysis / Gluconeogenesis, Pentose
                                  phosphate pathway,  Fructose and mannose
                                  metabolism
```

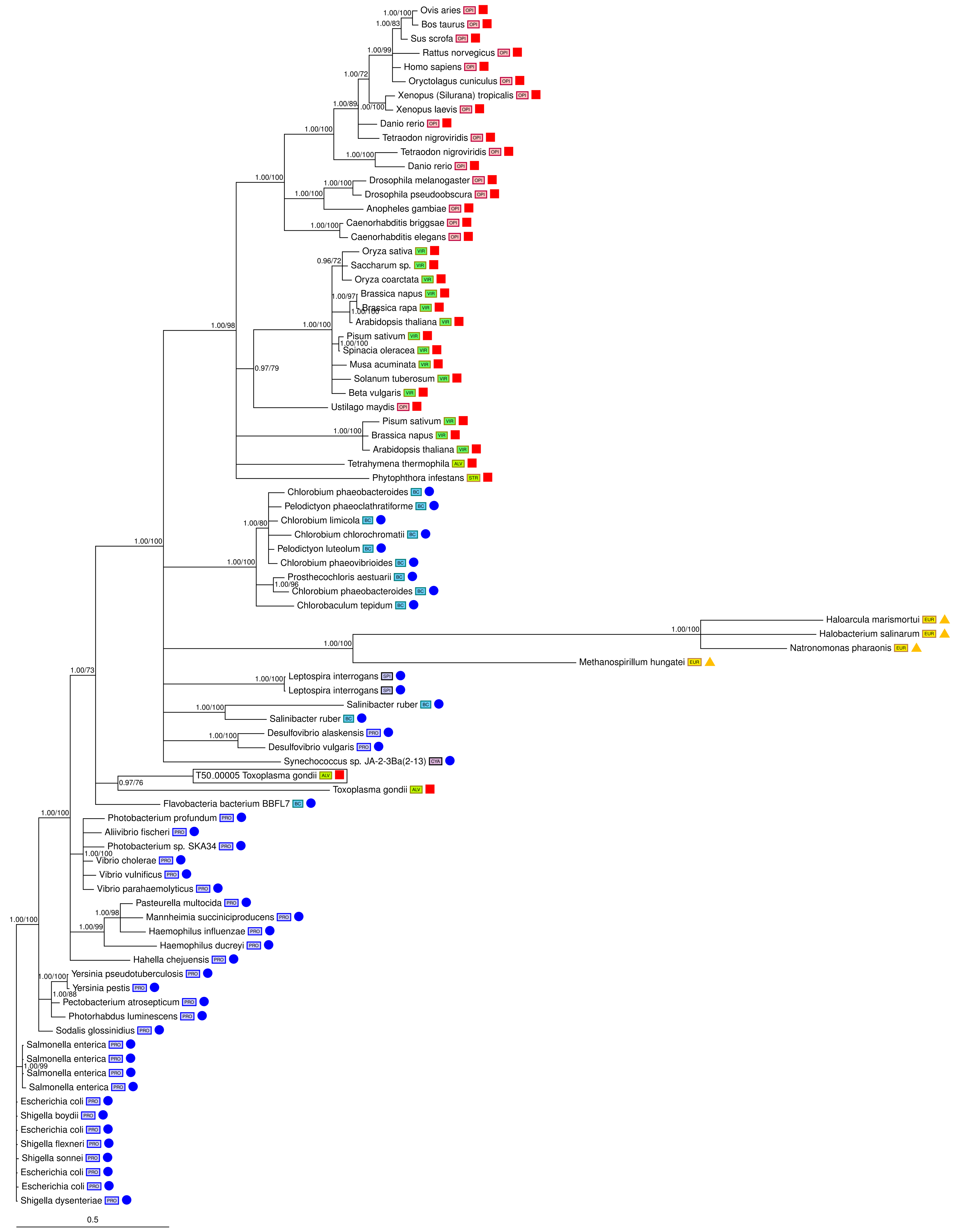

ON052

```
               Candy accession: T59_03682
              RefSeq accession: XP_002365950.1
             Uniprot accession: B6KDM7_TOXGO
                      Comments: LGT - APICOMPLEXA ONE NODE + FUNGI
              Species affected: PF,PV,PY,TG,CP
          Adjacent taxa in tree: Bacteria
EC annotation - (Blast/Profile): EC:6.3.1.2
                    PHOBIUS SP: Y
                   PHOBIUS TMD: 0
             RefSeq annotation: glutamine synthetase, putative
      Name of enzyme/protein: glutamate-ammonia ligase
      KEGG PATHWAY - level 1: Amino Acid Metabolism, Energy Metabolism
      KEGG PATHWAY - level 2: Alanine, aspartate and glutamate
                              metabolism, Arginine and proline
                              metabolism,  Nitrogen metabolism
```

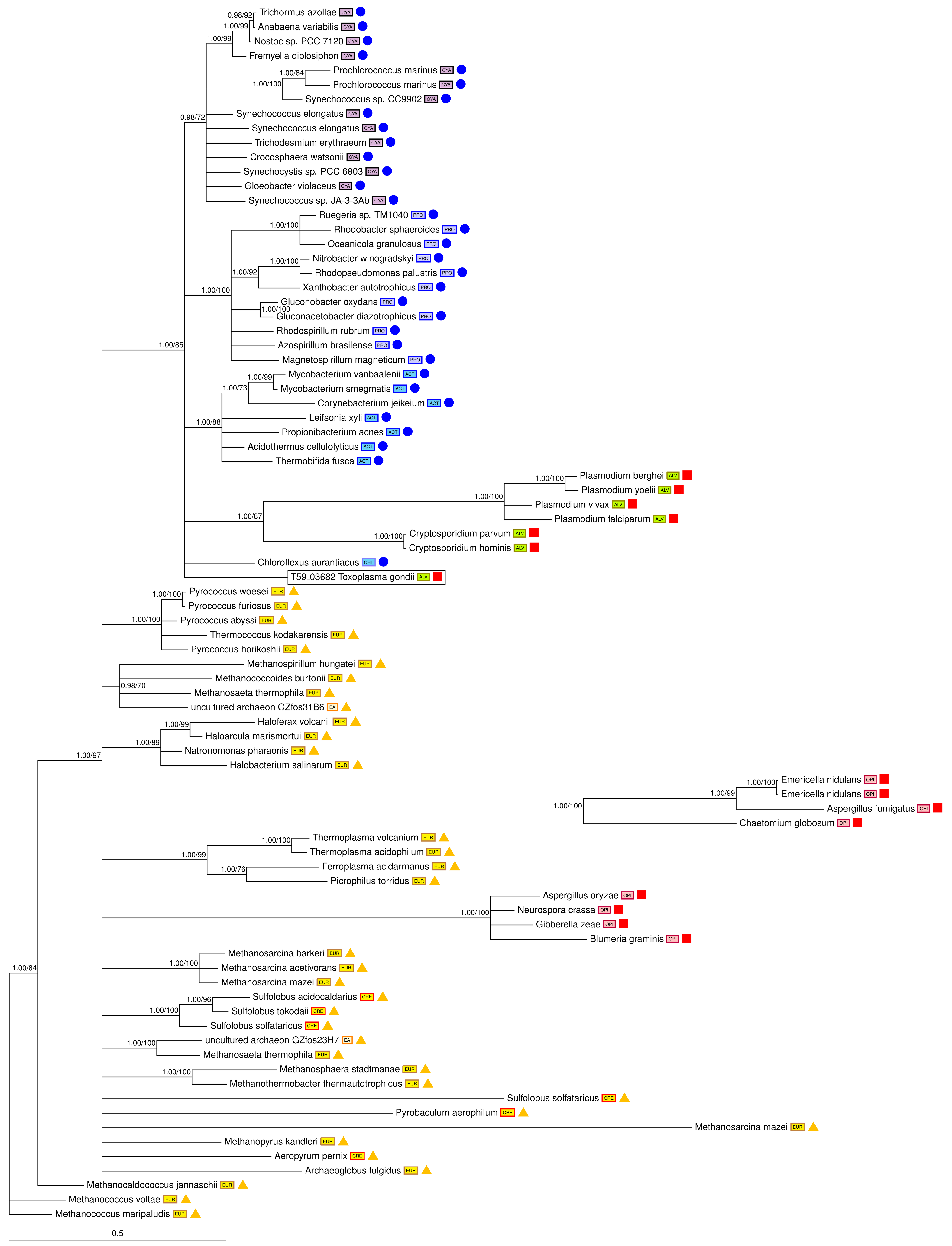

ON053

```
              Candy accession: TV94179274
             RefSeq accession: XP_001580988.1
            Uniprot accession: A2DI13_TRIVA
                     Comments: LGT - TV ONE NODE + PHAGES + VIRUSES -
                               3 DOMAIN ToL
             Species affected: TV
         Adjacent taxa in tree: Prokaryotes/Phages
EC annotation - (Blast/Profile): EC:1.17.4.1
                   PHOBIUS SP: 0
                  PHOBIUS TMD: 0
            RefSeq annotation: Ribonucleotide reductase, all-alpha
                               domain containing protein
     Name of enzyme/protein: Ribonucleoside-diphosphate reductase
                               alpha chain
       KEGG PATHWAY - level 1: Nucleotide Metabolism, Metabolism of
                               Other Amino Acids
       KEGG PATHWAY - level 2: Purine metabolism, Pyrimidine
                               metabolism, Glutathione metabolism
```

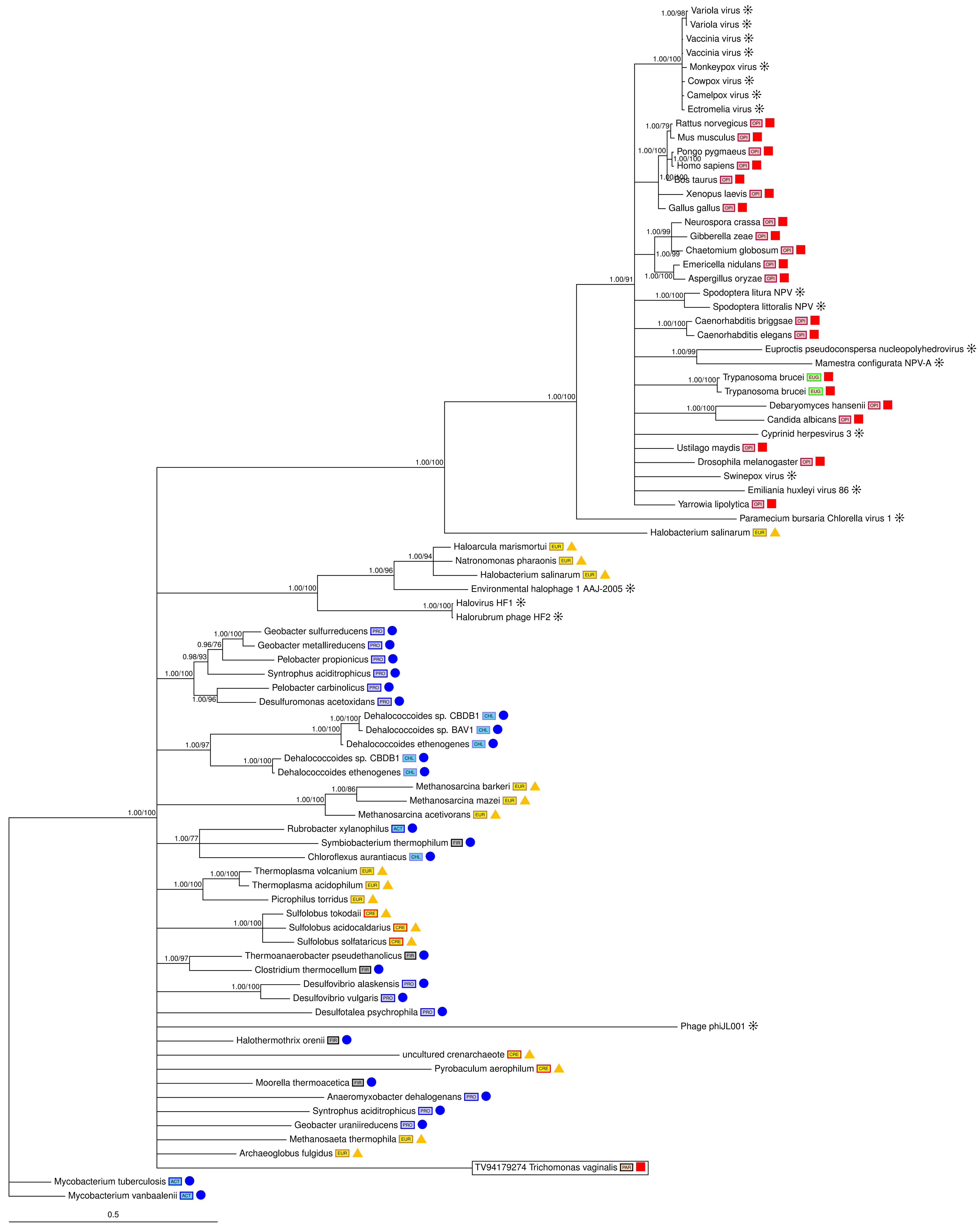

ON054

```
                  Candy accession: Q54N71_DICDI
                 RefSeq accession: XP_638072.1
                Uniprot accession: Q54N71_DICDI
                         Comments: LGT - DD ONE NODES
                 Species affected: DD
           Adjacent taxa in tree: Proteobacteria
EC annotation - (Blast/Profile): EC:3.5.99.3
                       PHOBIUS SP: 0
                      PHOBIUS TMD: 0
               RefSeq annotation: hypothetical protein DDB_G0285467
          Name of enzyme/protein: hydroxydechloroatrazine
                                   ethylaminohydrolase
          KEGG PATHWAY - level 1: Xenobiotics Biodegradation and
                                   Metabolism
          KEGG PATHWAY - level 2: Atrazine degradation
```

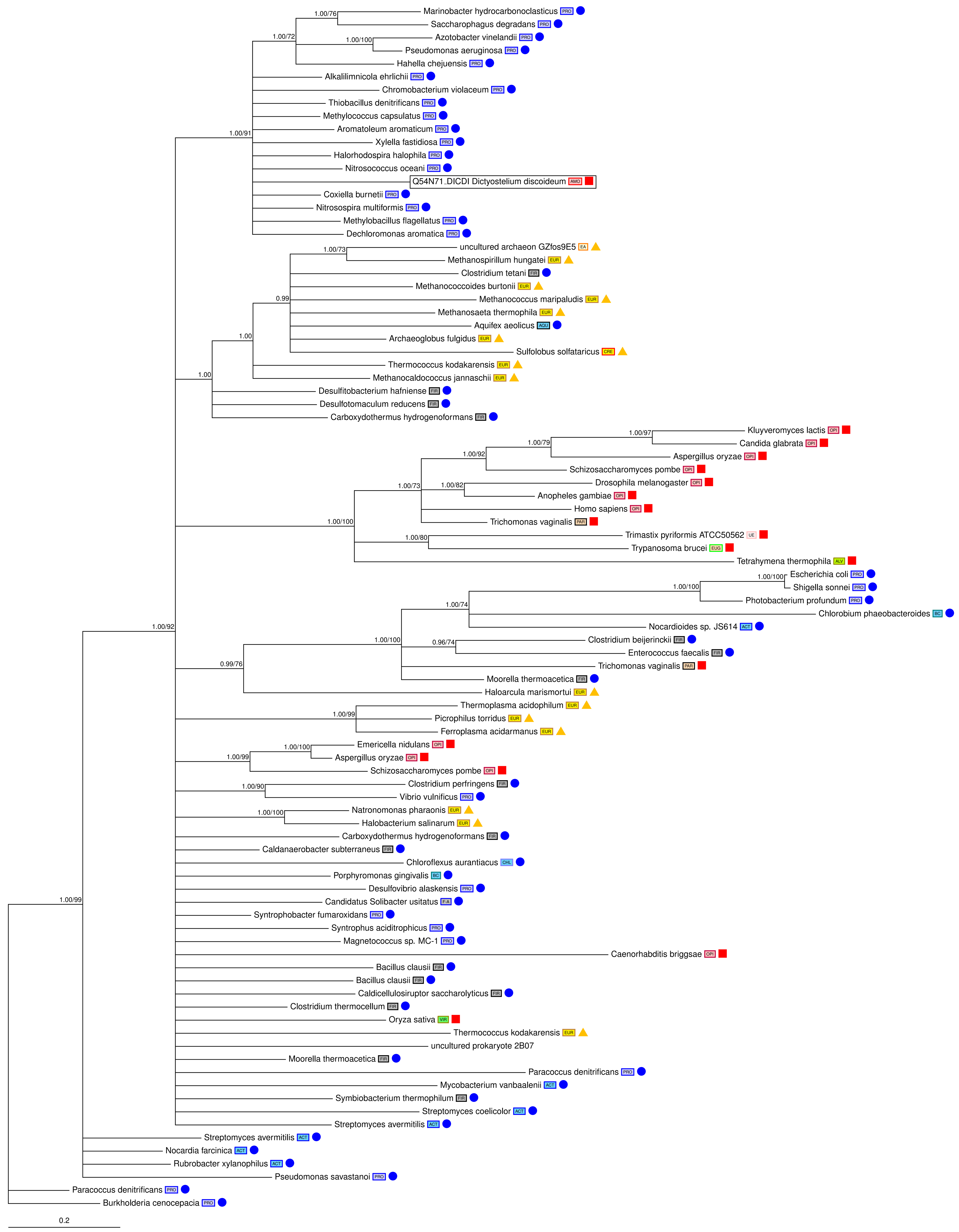

ON055

```
         Candy accession: TV92069237
        RefSeq accession: XP_001328466.1
       Uniprot accession: A2DTU6_TRIVA
                Comments: LGT - TV ONE NODE FROM FUNGI
        Species affected: TV,DD
  Adjacent taxa in tree: Firmicutes - Streptococcus
EC annotation - (Blast/Profile): na
              PHOBIUS SP: 0
             PHOBIUS TMD: 0
       RefSeq annotation: hypothetical protein
  Name of enzyme/protein: Predicted Esterase/lipase; alpha/beta
                          hydrolase fold
  KEGG PATHWAY - level 1: Other function
  KEGG PATHWAY - level 2: na
```

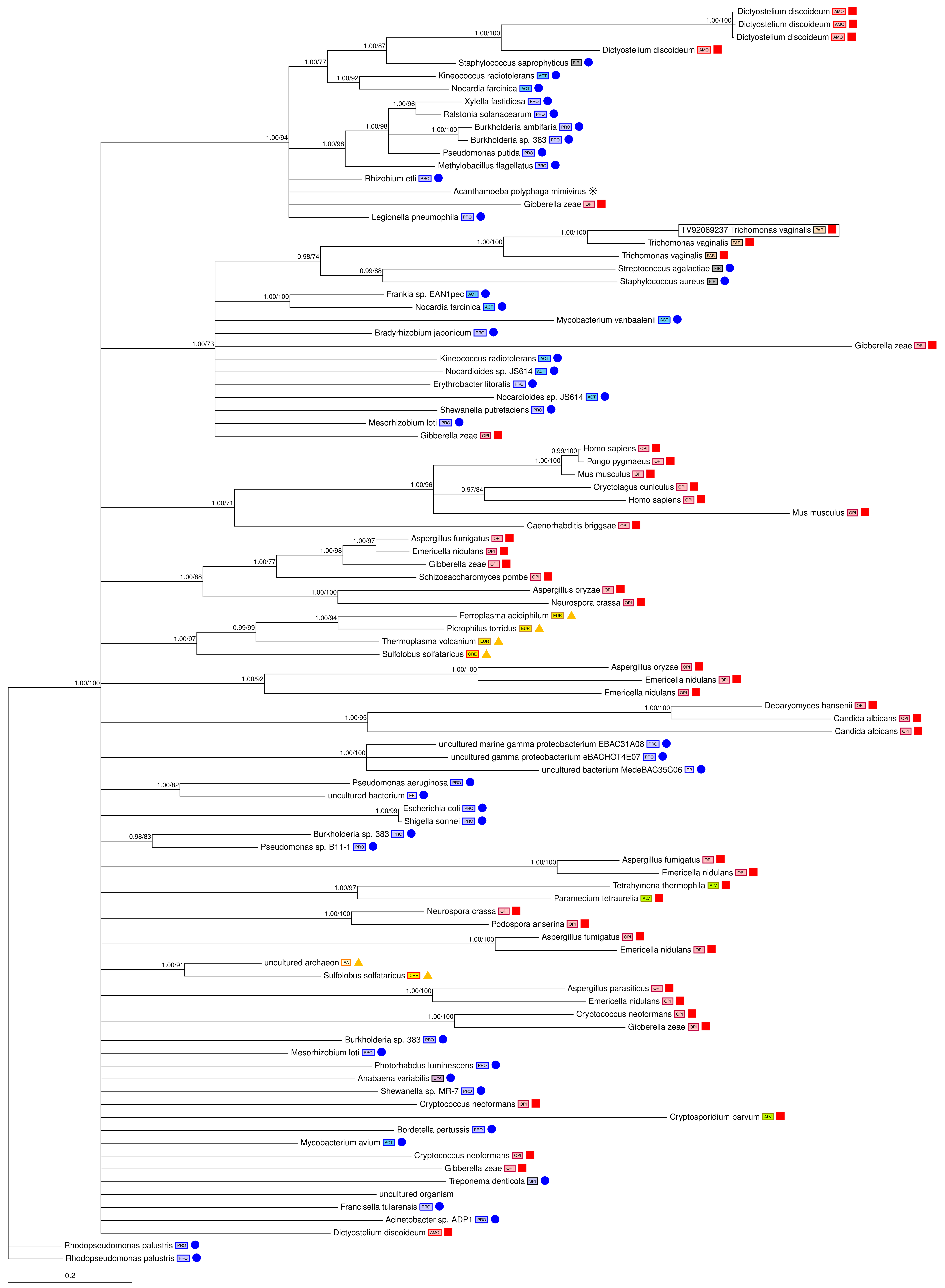

ON056

```
            Candy accession: TV93091063
           RefSeq accession: XP_001308954.1
          Uniprot accession: A2FGG0_TRIVA
                   Comments: LGT - TV ONE NODE
           Species affected: TV
       Adjacent taxa in tree: Bacteria
EC annotation - (Blast/Profile): EC:6.1.1.7
                 PHOBIUS SP: 0
                PHOBIUS TMD: 0
          RefSeq annotation: hypothetical protein
    Name of enzyme/protein: Probable R-RNA methyltransferase
     KEGG PATHWAY - level 1: Translation - Genetic Information
                             Processing
     KEGG PATHWAY - level 2: Aminoacyl-tRNA biosynthesis
```

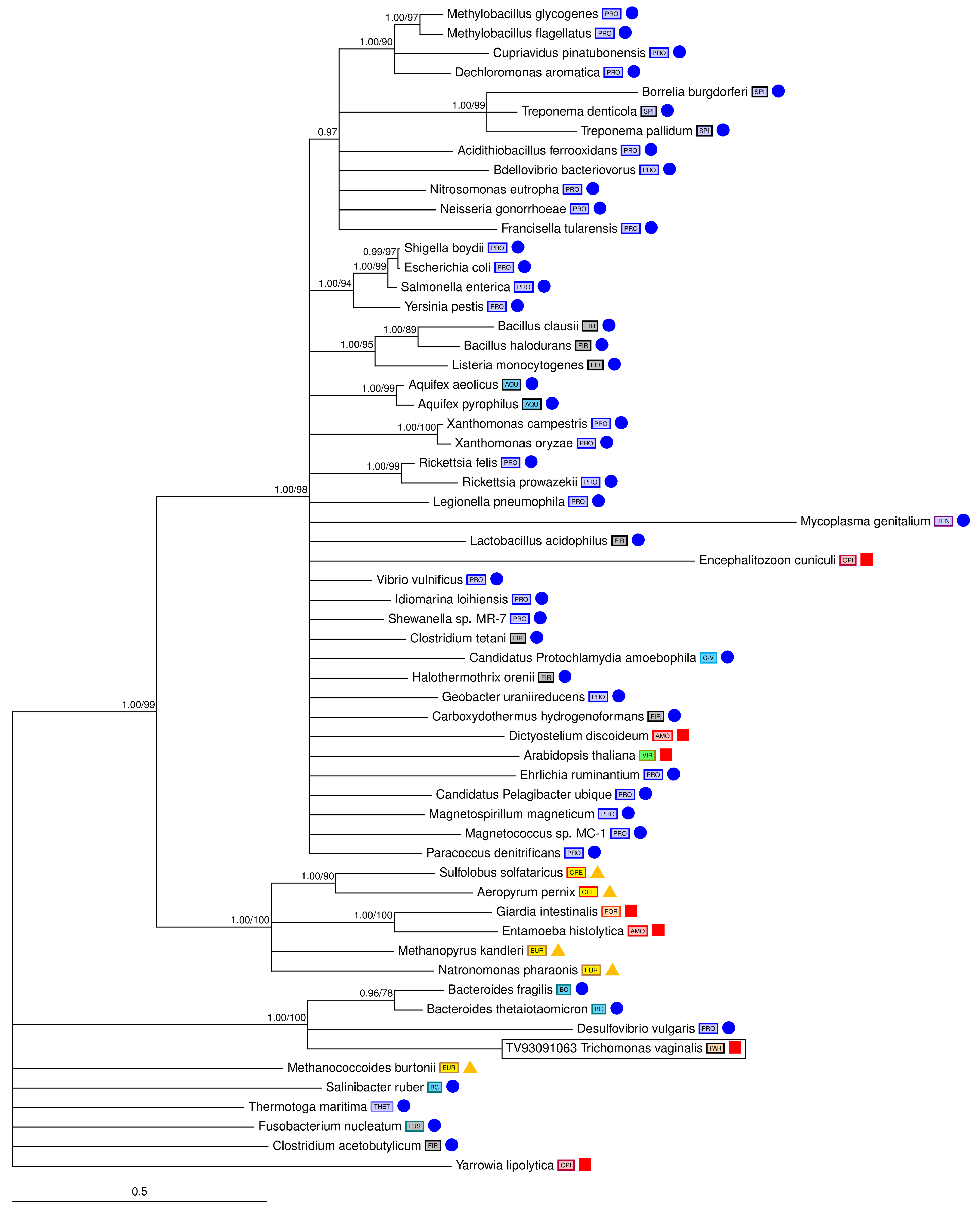

ON057

Candy accession: TV94534157
RefSeq accession: XP_001321975.1
Uniprot accession: A2EC75_TRIVA
Comments: LGT - TV ONE NODE
Species affected: TV
Adjacent taxa in tree: Bacteria
EC annotation - (Blast/Profile): EC:1.3.1.20
PHOBIUS SP: 0
PHOBIUS TMD: 0
RefSeq annotation: Oxidoreductase family, NAD-binding Rossmann fold containing protein
Name of enzyme/protein: trans-1,2-dihydrobenzene-1,2-diol dehydrogenase
KEGG PATHWAY - level 1: Xenobiotics Biodegradation and Metabolism
KEGG PATHWAY - level 2: Metabolism of xenobiotics by cytochrome P450

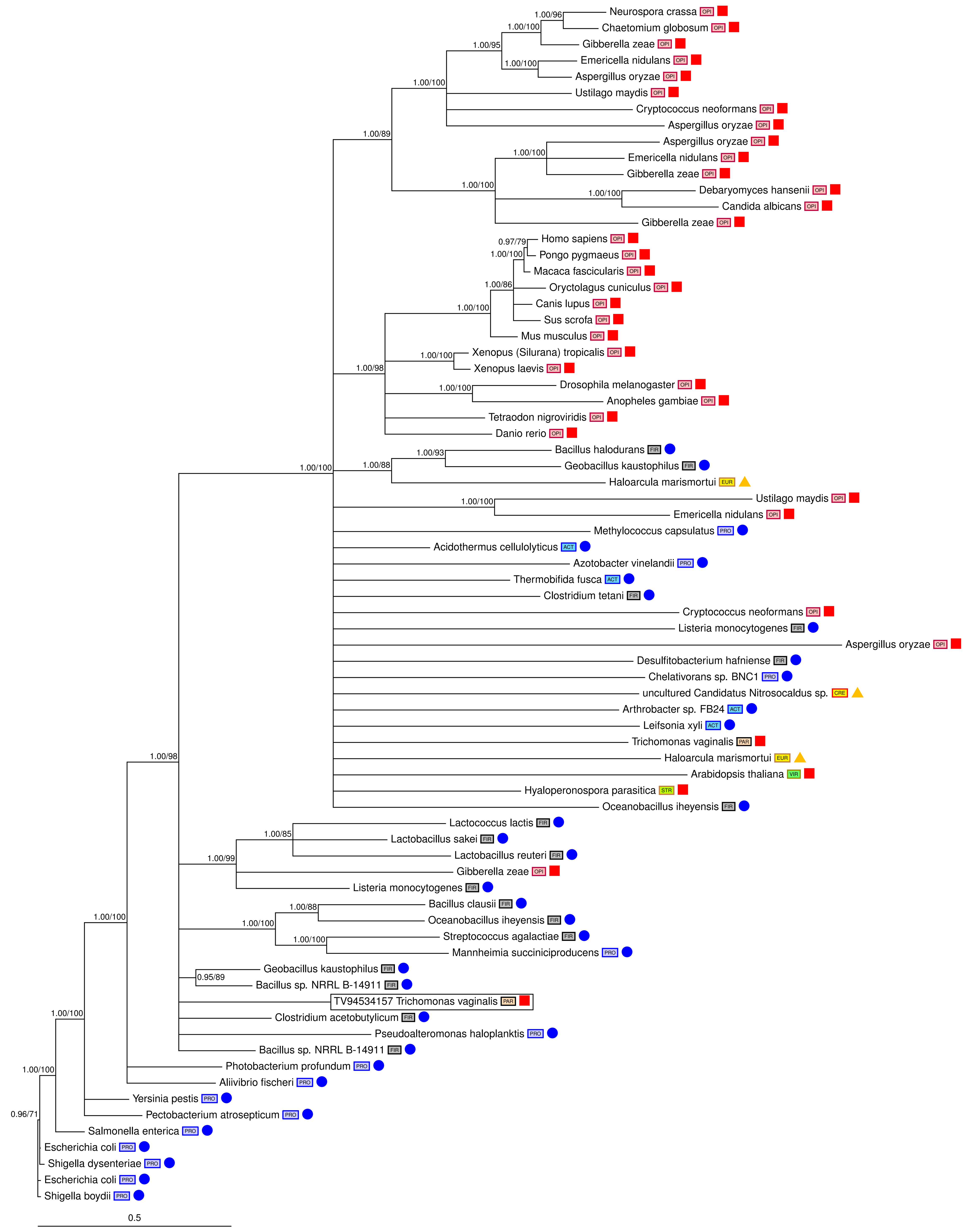

ON058

```
                Candy accession: TV138932002
               RefSeq accession: XP_001279673.1
              Uniprot accession: A2HV24_TRIVA
                       Comments: LGT - TV ONE NODE
               Species affected: TV
           Adjacent taxa in tree: Bacteroidetes/Chlorobi  - Bacteroides
EC annotation - (Blast/Profile): EC:3.2.1.45
                     PHOBIUS SP: 0
                    PHOBIUS TMD: 0
               RefSeq annotation: glucosylceramidase precursor
          Name of enzyme/protein: glucosylceramidase
          KEGG PATHWAY - level 1: Glycan Biosynthesis and Metabolism,
                                  Lipid Metabolism
          KEGG PATHWAY - level 2: Other glycan degradation,  Sphingolipid
                                  metabolism
```

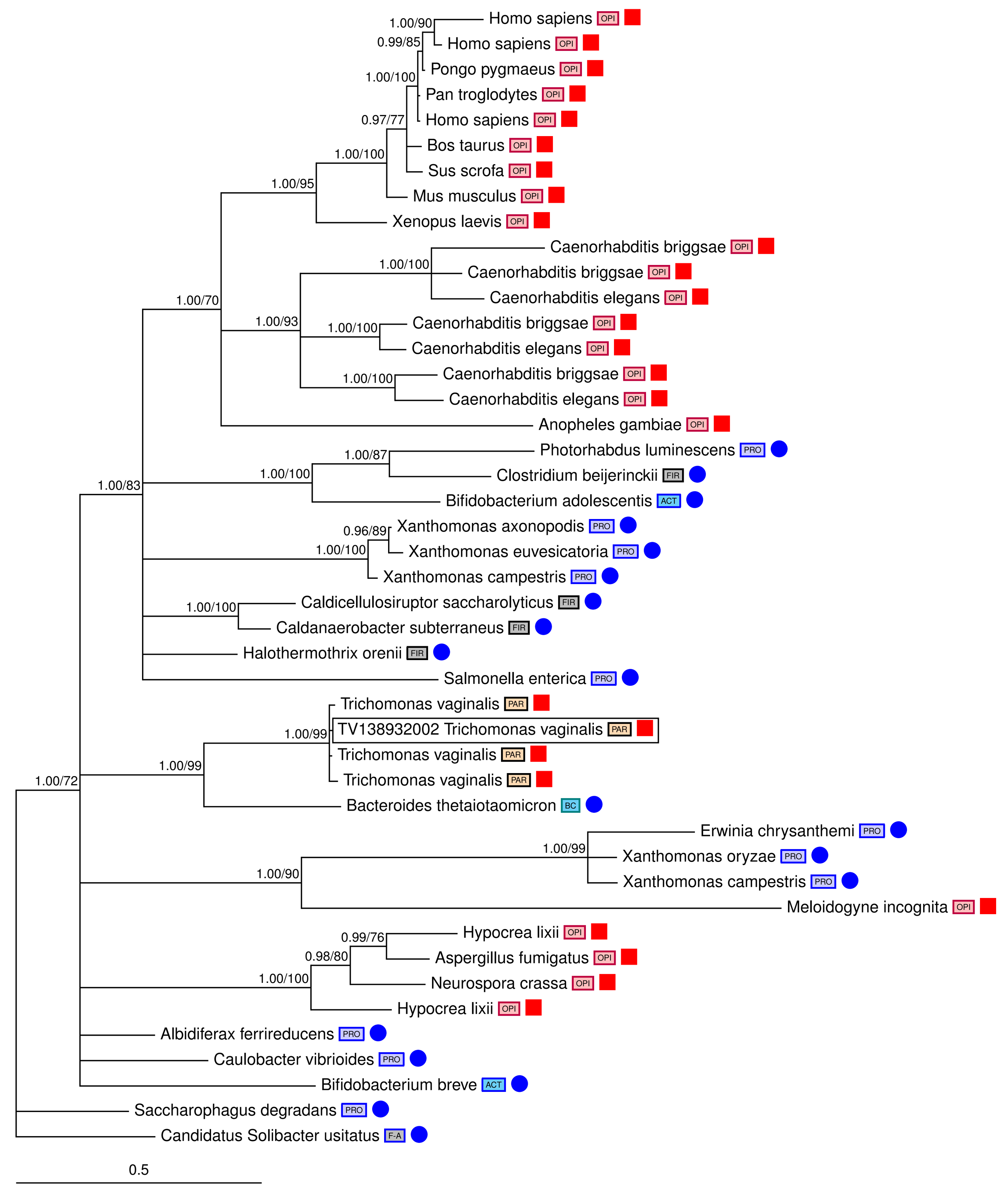

ON059

```
                Candy accession: Q7YYI0_CRYPV
               RefSeq accession: XP_627573.1
              Uniprot accession: Q7YYI0_CRYPV
                       Comments: LGT - APICOMPLEXA ONE NODE
               Species affected: CP,CH,TP,TA,TL,PC,PY,PF,PV
          Adjacent taxa in tree: Bacteria
EC annotation - (Blast/Profile): na
                     PHOBIUS SP: 0
                    PHOBIUS TMD: 0
              RefSeq annotation: hypothetical protein
       Name of enzyme/protein: Predicted R-RNA methyltransferase
       KEGG PATHWAY - level 1: Other function
       KEGG PATHWAY - level 2: na

                Candy accession: Q54SC0_DICDI
               RefSeq accession: XP_640119.1
              Uniprot accession: Q54SC0_DICDI
                       Comments: LGT - DD ONE NODE
               Species affected: DD
          Adjacent taxa in tree: Bacteria
EC annotation - (Blast/Profile): na
                     PHOBIUS SP: 0
                    PHOBIUS TMD: 0
              RefSeq annotation: hypothetical protein DDB_G0282549
       Name of enzyme/protein: Predicted R-RNA methyltransferase
       KEGG PATHWAY - level 1: Other function
       KEGG PATHWAY - level 2: na
```

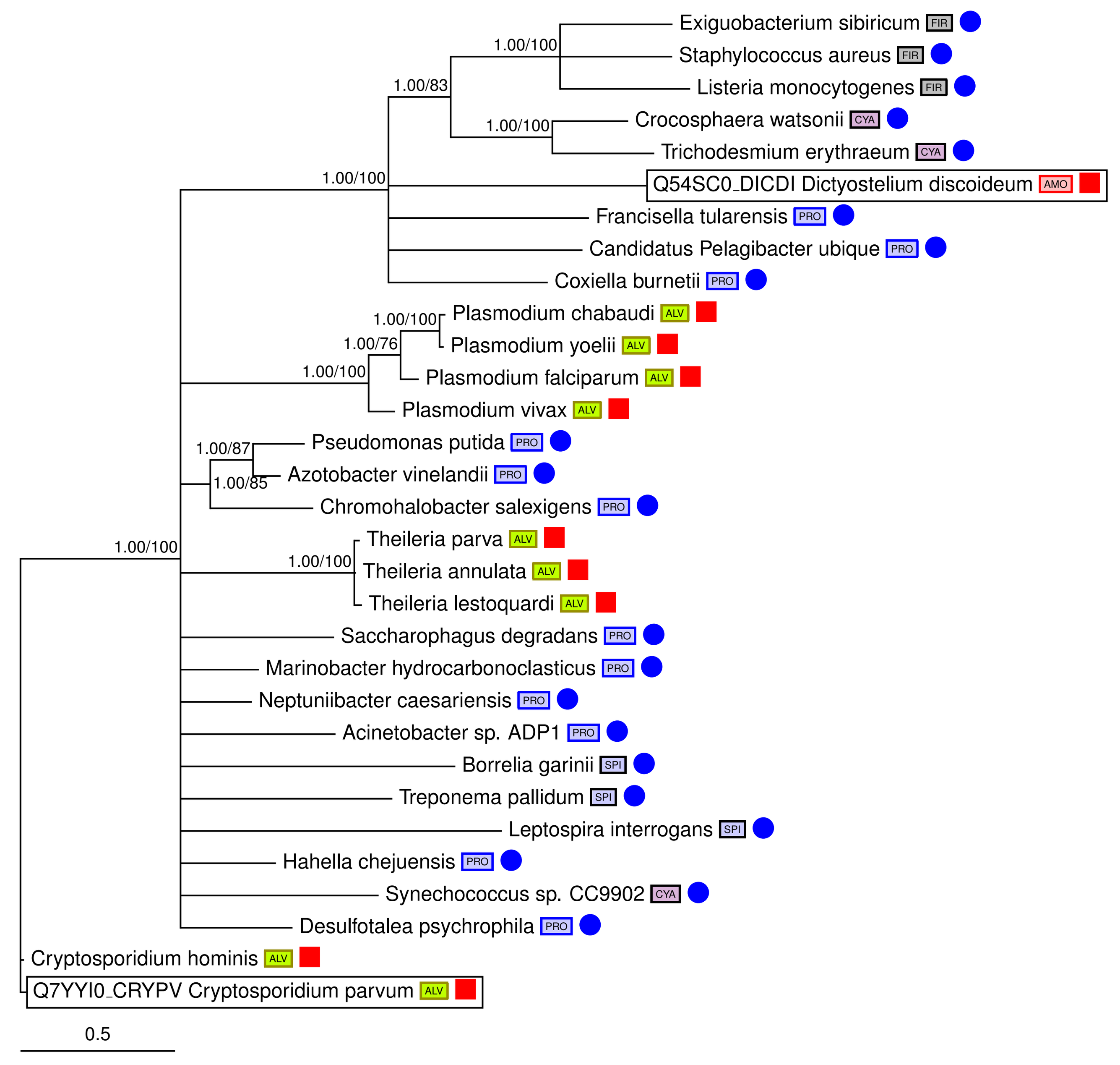

ON060

```
          Candy accession: Q7QPA1_GIALA
         RefSeq accession: XP_001709668.1
        Uniprot accession: Q7QPA1_GIALA
                 Comments: LGT - GI, SB ONE NODE - 3 DOMAIN ToL
         Species affected: GI,SB
      Adjacent taxa in tree: Prokaryotes
EC annotation - (Blast/Profile): EC:6.3.4.2
              PHOBIUS SP: 0
             PHOBIUS TMD: 0
        RefSeq annotation: CTP synthase
  Name of enzyme/protein: CTP synthase
   KEGG PATHWAY - level 1: Nucleotide Metabolism
   KEGG PATHWAY - level 2: Pyrimidine metabolism
```

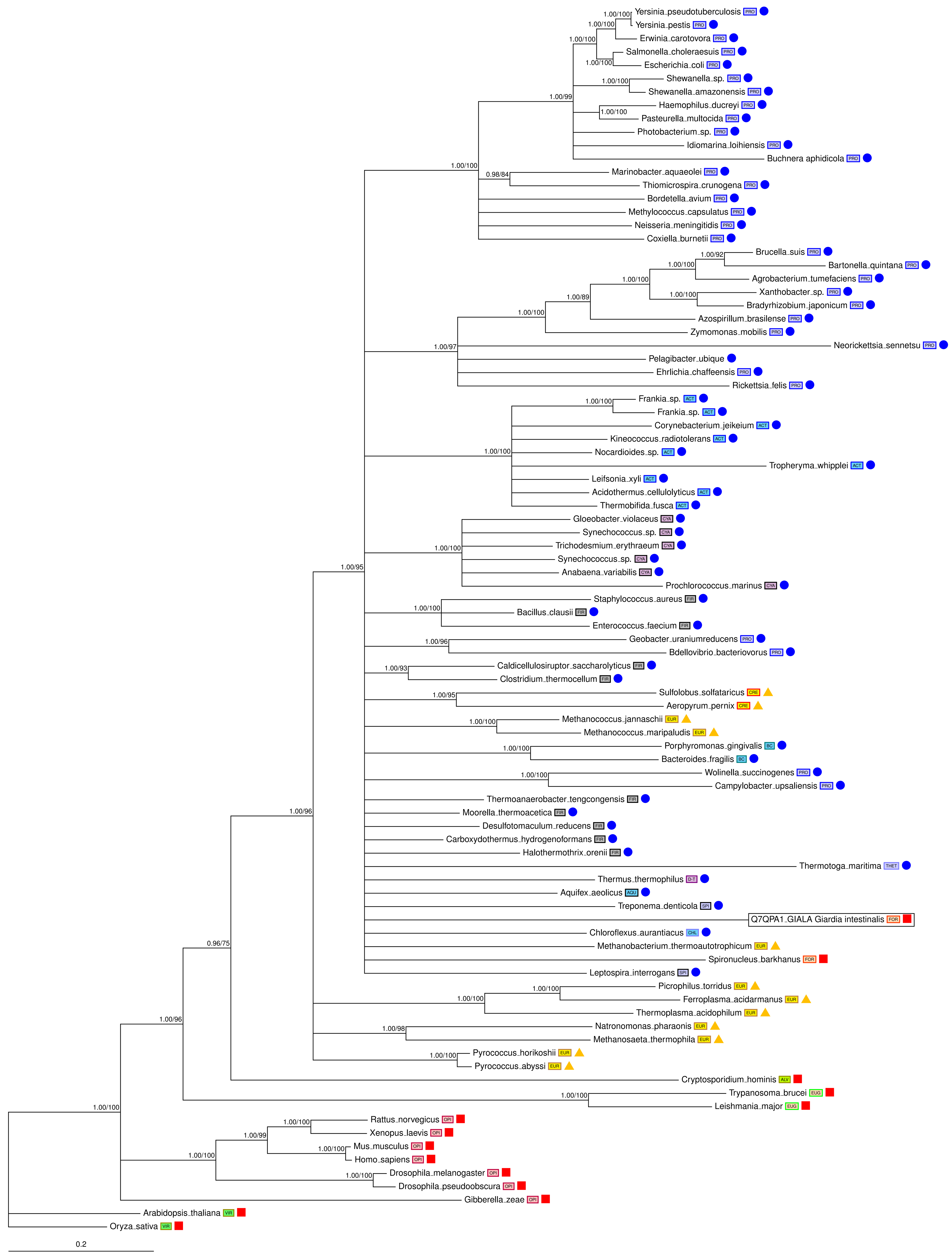

ON061

```
            Candy accession: XP_625304
           RefSeq accession: XP_625304
          Uniprot accession: Q5CPD7_CRYPV
                   Comments: LGT - CP ONE NODE
           Species affected: CP,FUNGI,PLANT, STRAMENOPILE
       Adjacent taxa in tree: Proteobacteria - Campylobacter
EC annotation - (Blast/Profile): EC:4.2.1.20
                 PHOBIUS SP: 0
                PHOBIUS TMD: 1
          RefSeq annotation: tryptophan synthase trpB of possible
                             bacterial origin
      Name of enzyme/protein: Tryptophan synthase
     KEGG PATHWAY - level 1: Amino Acid Metabolism
     KEGG PATHWAY - level 2: Glycine, serine and threonine
                             metabolism, Phenylalanine, tyrosine and
                             tryptophan biosynthesis
```

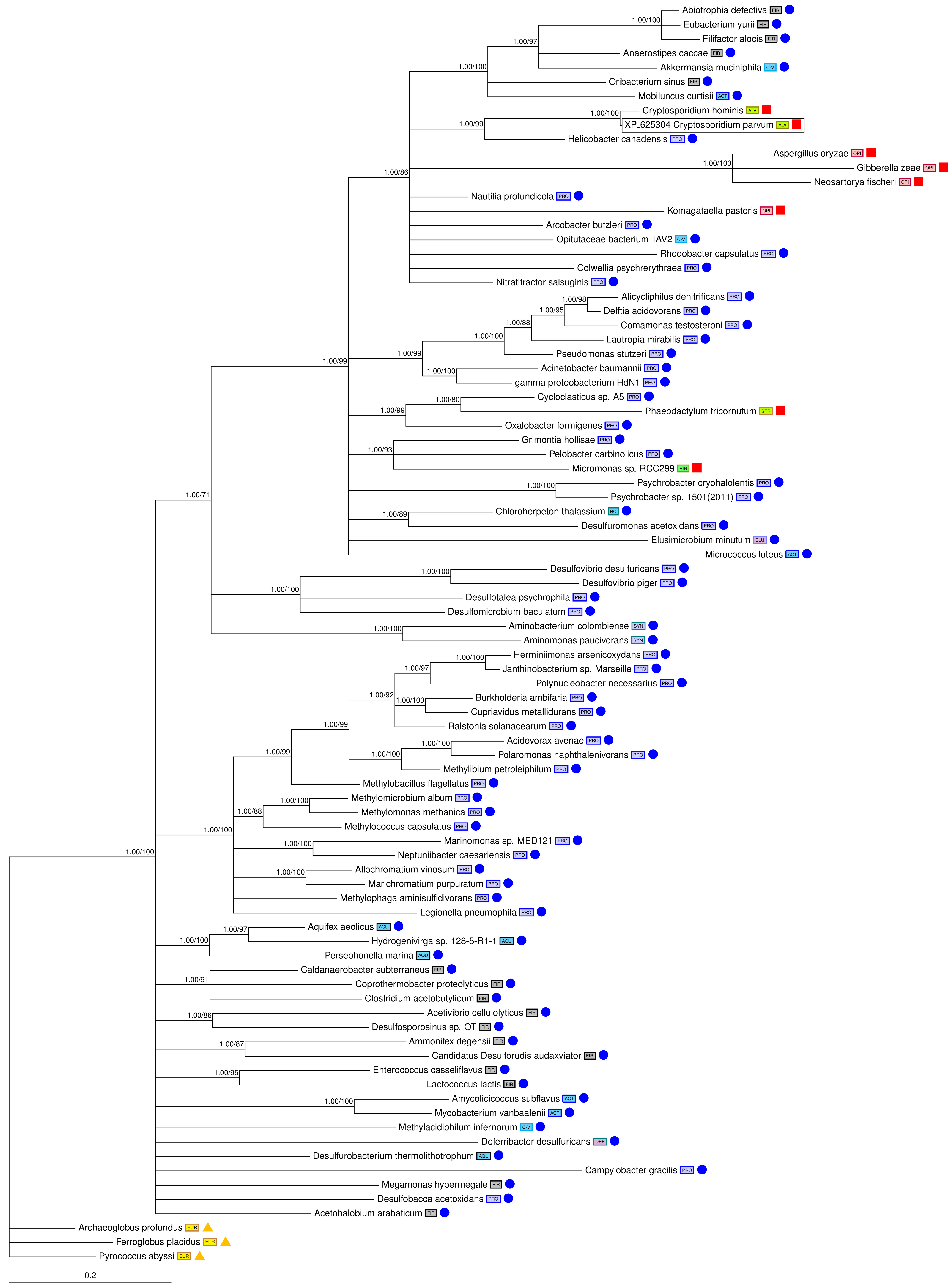

ON062

Candy accession: XP_001686812
RefSeq accession: XP_001686812
Uniprot accession: Q4Q1I6_LEIMA
Comments: LGT - KINETOPLASTIDS (plus LGT into bumblebee Bombus impatiens?)
Species affected: LM, TB, TC
Adjacent taxa in tree: g-proteobacteria
EC annotation - (Blast/Profile): EC:1.2.1.12
PHOBIUS SP: 0
PHOBIUS TMD: 0
RefSeq annotation: glyceraldehyde 3-phosphate dehydrogenase, cytosolic
Name of enzyme/protein: Glyceraldehyde 3-phosphate dehydrogenase
KEGG PATHWAY - level 1: Carbohydrate Metabolism
KEGG PATHWAY - level 2: Glycolysis / Gluconeogenesis

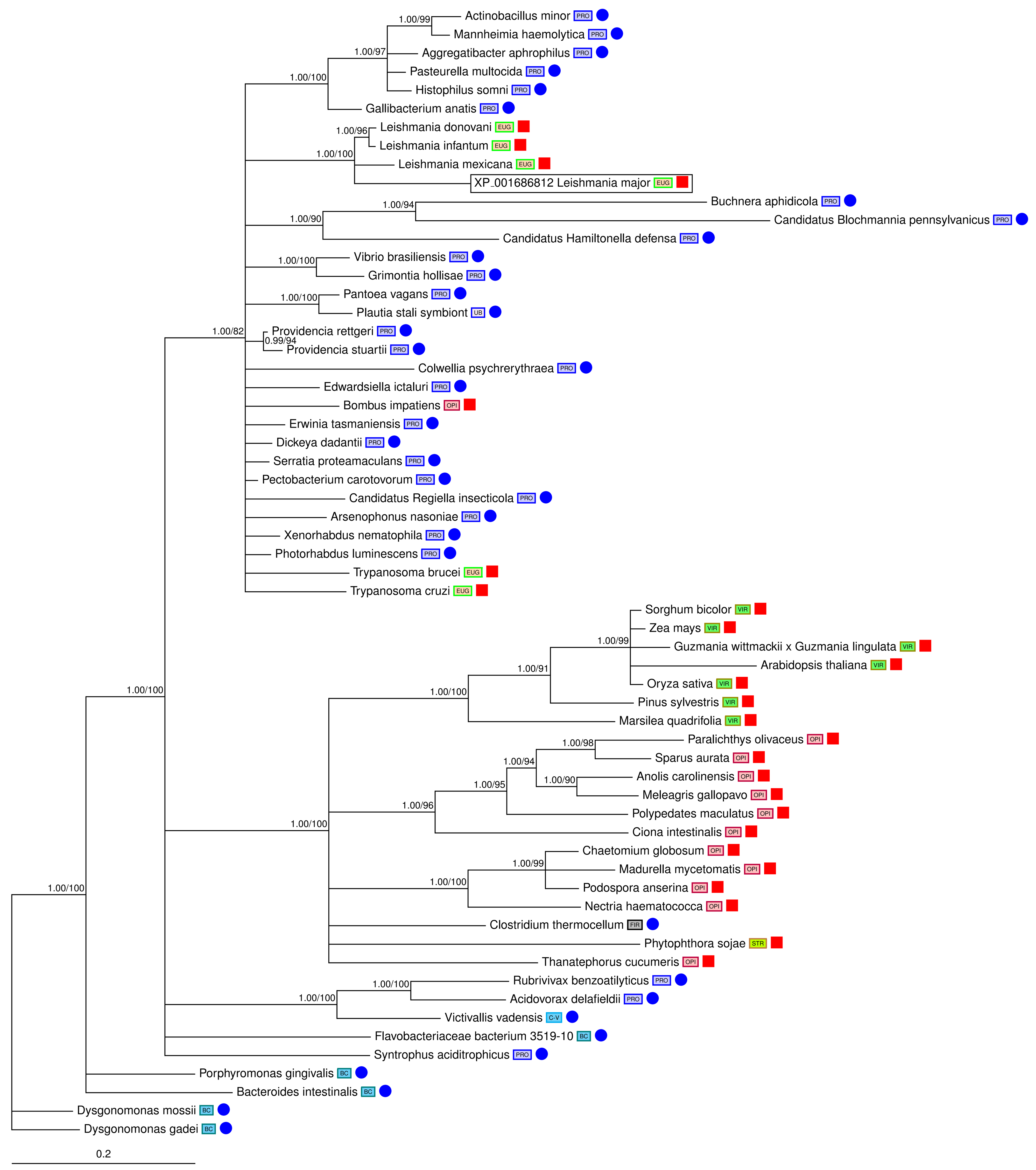

ON063

```
                Candy accession: TV84302326
               RefSeq accession: XP_001579222.1
              Uniprot accession: A2DN59_TRIVA
                       Comments: LGT - TV ONE NODE
               Species affected: TV
         Adjacent taxa in tree: Prokaryotes
EC annotation - (Blast/Profile): EC:3.2.1.24
                    PHOBIUS SP: 0
                   PHOBIUS TMD: 0
             RefSeq annotation: glycosyl hydrolase
        Name of enzyme/protein: a-mannosidase
        KEGG PATHWAY - level 1: Glycan Biosynthesis and Metabolism
        KEGG PATHWAY - level 2: Other glycan degradation
```

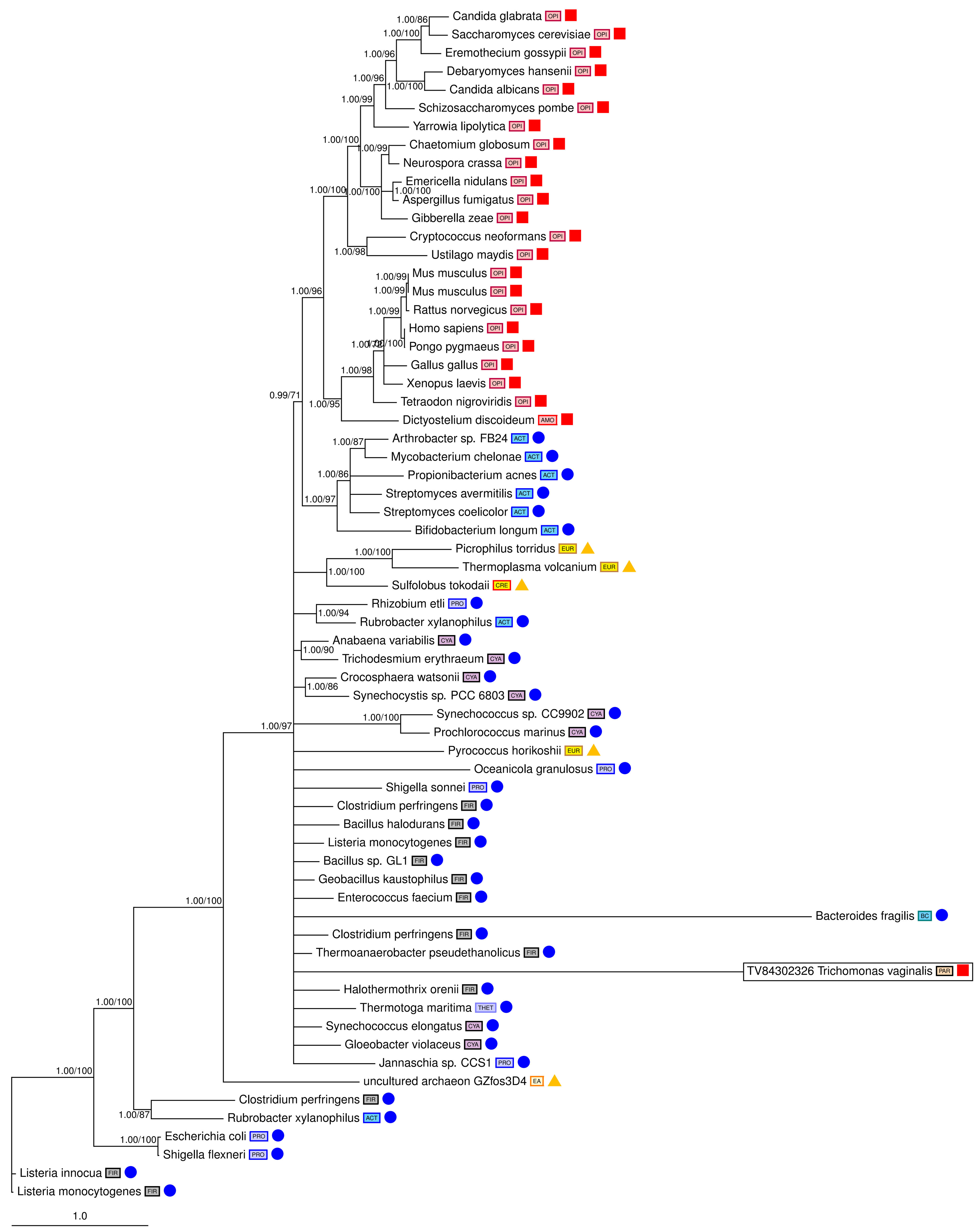

TN001

Candy accession:	TV122580003
RefSeq accession:	XP_001285086.1
Uniprot accession:	A2HEL1_TRIVA
Comments:	LGT - TV ONLY
Species affected:	TV
Adjacent taxa in tree:	Bacteria
EC annotation - (Blast/Profile):	na
PHOBIUS SP:	0
PHOBIUS TMD:	0
RefSeq annotation:	hypothetical protein
Name of enzyme/protein:	Predictded D12 class N6 adenine-specific DNA methyltransferase
KEGG PATHWAY - level 1:	Other function - Genetic Information Processing
KEGG PATHWAY - level 2:	na

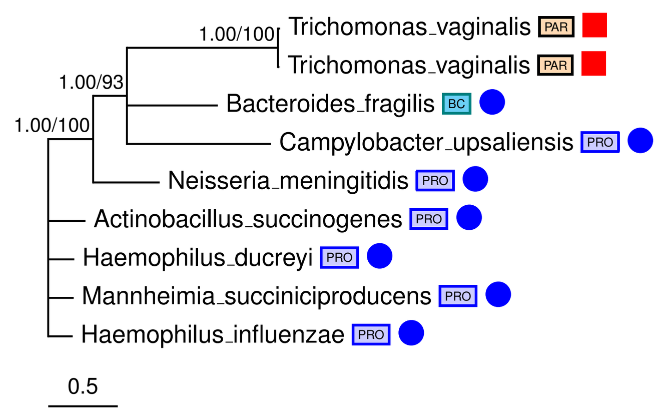

TN002

```
                 Candy accession: Q4D587_TRYCR
                RefSeq accession: XP_809539.1
               Uniprot accession: Q4D587_TRYCR
                        Comments: LGT - TC ONLY
                Species affected: TC
           Adjacent taxa in tree: Prokaryotes
EC annotation - (Blast/Profile): na
                      PHOBIUS SP: 0
                     PHOBIUS TMD: 0
               RefSeq annotation: metallo-beta-lactamase superfamily
         Name of enzyme/protein: Predicted metallo-beta-lactamase protein
         KEGG PATHWAY - level 1: Other function
         KEGG PATHWAY - level 2: na
```

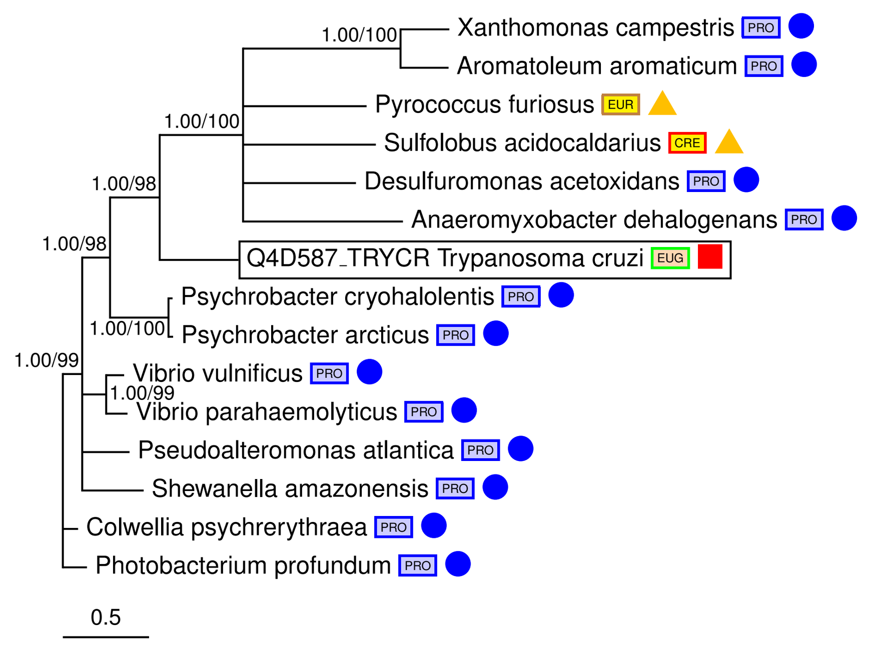

TN003

Candy accession:	Q4GY96_9TRYP
RefSeq accession:	XP_001219175.1
Uniprot accession:	Q4GY96_9TRYP
Comments:	LGT - TB ONLY
Species affected:	TB
Adjacent taxa in tree:	Bacteria
EC annotation - (Blast/Profile):	EC:3.1.1.32
PHOBIUS SP:	0
PHOBIUS TMD:	0
RefSeq annotation:	phospholipase A1
Name of enzyme/protein:	phospholipase A1
KEGG PATHWAY - level 1:	Lipid Metabolism
KEGG PATHWAY - level 2:	Glycerophospholipid metabolism, alpha-Linolenic acid metabolism

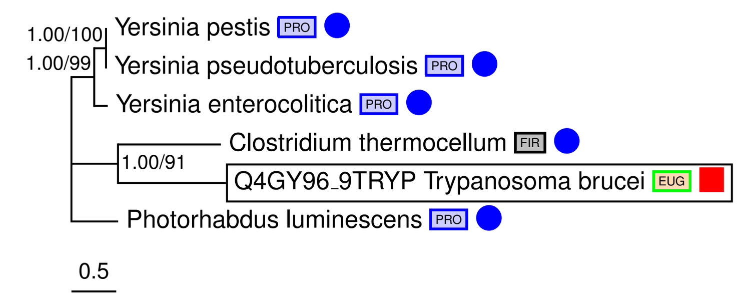

TN004

```
                Candy accession: Q4Q0L8_LEIMA
               RefSeq accession: XP_001687130.1
              Uniprot accession: Q4Q0L8_LEIMA
                       Comments: LGT - KINETOPLASTIDS ONLY
               Species affected: LM,TB,TC
          Adjacent taxa in tree: Proteobacteria
EC annotation - (Blast/Profile): na
                     PHOBIUS SP: 0
                    PHOBIUS TMD: 0
              RefSeq annotation: hypothetical protein
         Name of enzyme/protein: Macrocin-O-methyltransferase
         KEGG PATHWAY - level 1: Other function
         KEGG PATHWAY - level 2: na
```

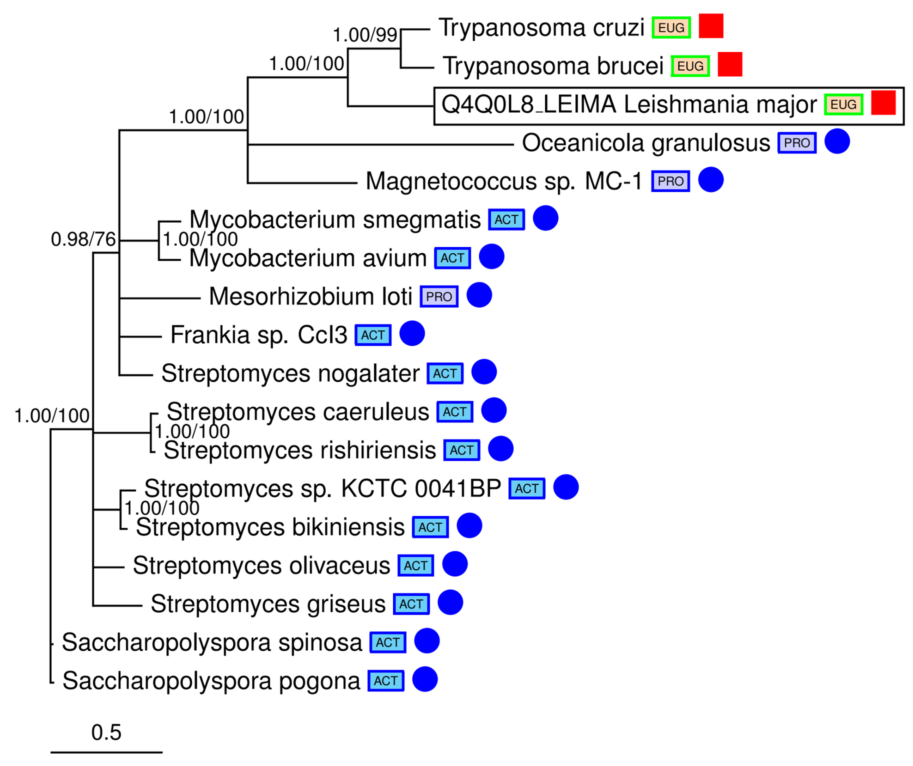

TN005

```
                Candy accession: Q4Q1L6_LEIMA
               RefSeq accession: XP_001686782.1
              Uniprot accession: Q4Q1L6_LEIMA
                       Comments: LGT - KINETOPLASTIDS ONLY
               Species affected: LM,TB,TC
          Adjacent taxa in tree: Bacteria
EC annotation - (Blast/Profile): na
                     PHOBIUS SP: Y
                    PHOBIUS TMD: 0
              RefSeq annotation: hypothetical protein
       Name of enzyme/protein: Hypothetical protein
       KEGG PATHWAY - level 1: Function unknown
       KEGG PATHWAY - level 2: na
```

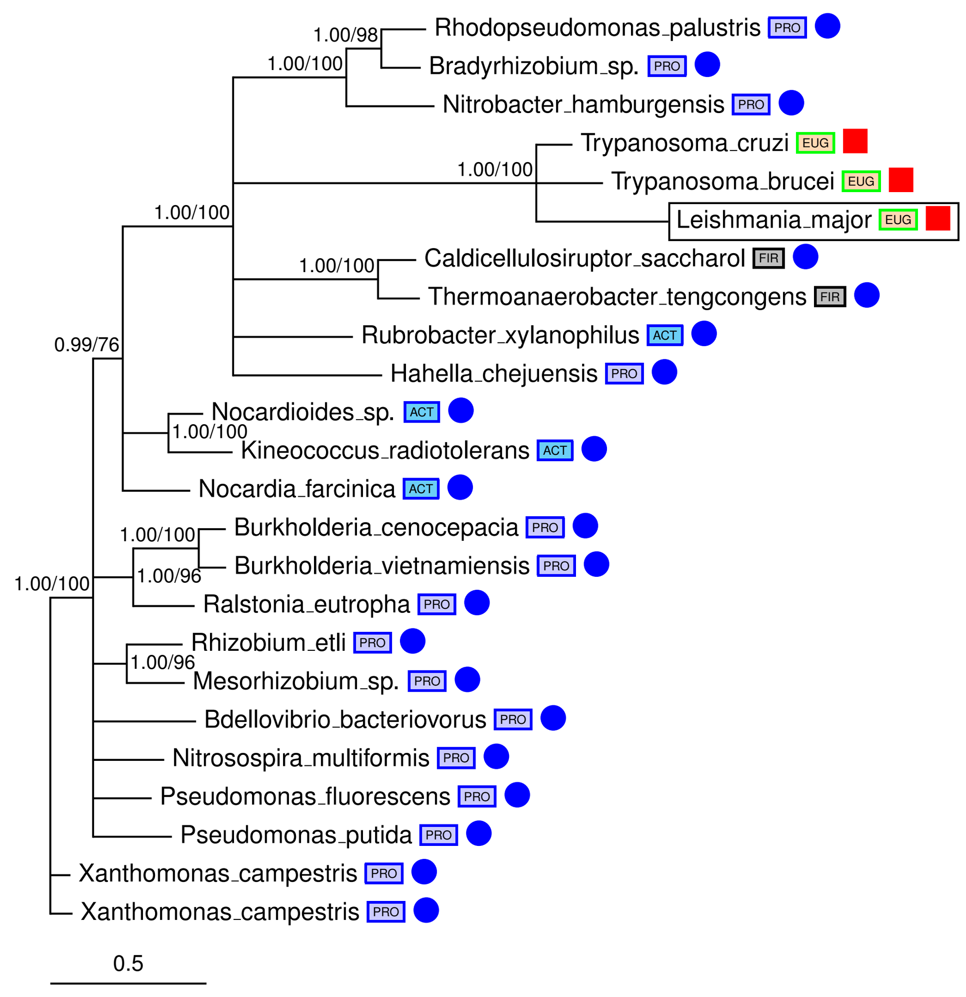

TN006

```
              Candy accession: Q4Q5Y1_LEIMA
             RefSeq accession: XP_001685267.1
            Uniprot accession: Q4Q5Y1_LEIMA
                     Comments: LGT - KINETOPLASTIDS ONLY
             Species affected: LM,TC
       Adjacent taxa in tree: Bacteria
EC annotation - (Blast/Profile): EC:3.5.1.16
                   PHOBIUS SP: 0
                  PHOBIUS TMD: 0
            RefSeq annotation: hypothetical protein
      Name of enzyme/protein: acetylornithine deacetylase
      KEGG PATHWAY - level 1: Amino Acid Metabolism
      KEGG PATHWAY - level 2: Arginine and proline metabolism
```

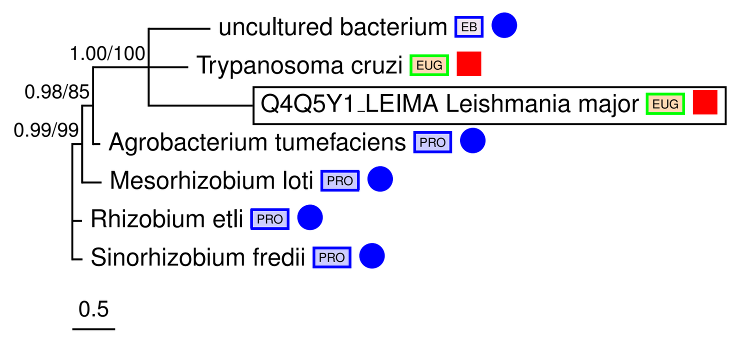

TN007

```
               Candy accession: Q4Q6Z3_LEIMA
              RefSeq accession: XP_001684905.1
             Uniprot accession: Q4Q6Z3_LEIMA
                      Comments: LGT - KINETOPLASTIDS ONLY
              Species affected: LM,TB,TC
        Adjacent taxa in tree: Cyanobacteria - Trichodesmium
EC annotation - (Blast/Profile): na
                    PHOBIUS SP: Y
                   PHOBIUS TMD: 1
             RefSeq annotation: hypothetical protein
       Name of enzyme/protein: Protein containing peptidase M14-like
                                domain
       KEGG PATHWAY - level 1: Other function
       KEGG PATHWAY - level 2: na
```

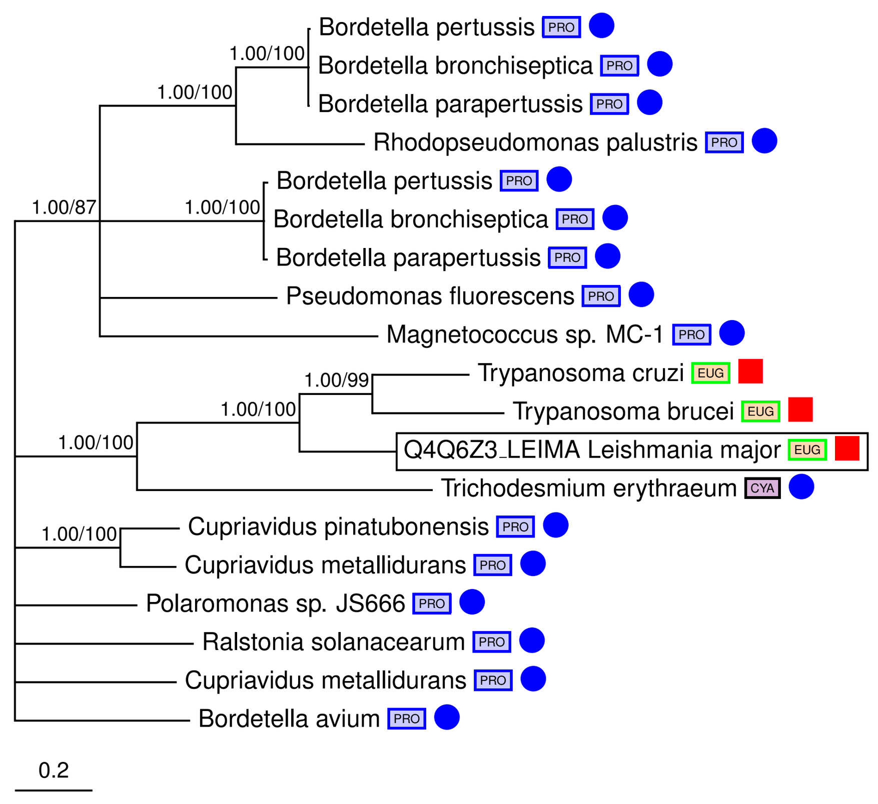

TN008

```
                 Candy accession: Q4Q8D2_LEIMA
                RefSeq accession: XP_001684416.1
               Uniprot accession: Q4Q8D2_LEIMA
                        Comments: LGT - KINETOPLASTIDS ONLY
                Species affected: LM,TB,TC
          Adjacent taxa in tree: Bacteria
EC annotation - (Blast/Profile): na
                     PHOBIUS SP: 0
                    PHOBIUS TMD: 0
              RefSeq annotation: hypothetical protein
       Name of enzyme/protein: Putative hydrolase
         KEGG PATHWAY - level 1: Other function
         KEGG PATHWAY - level 2: na
```

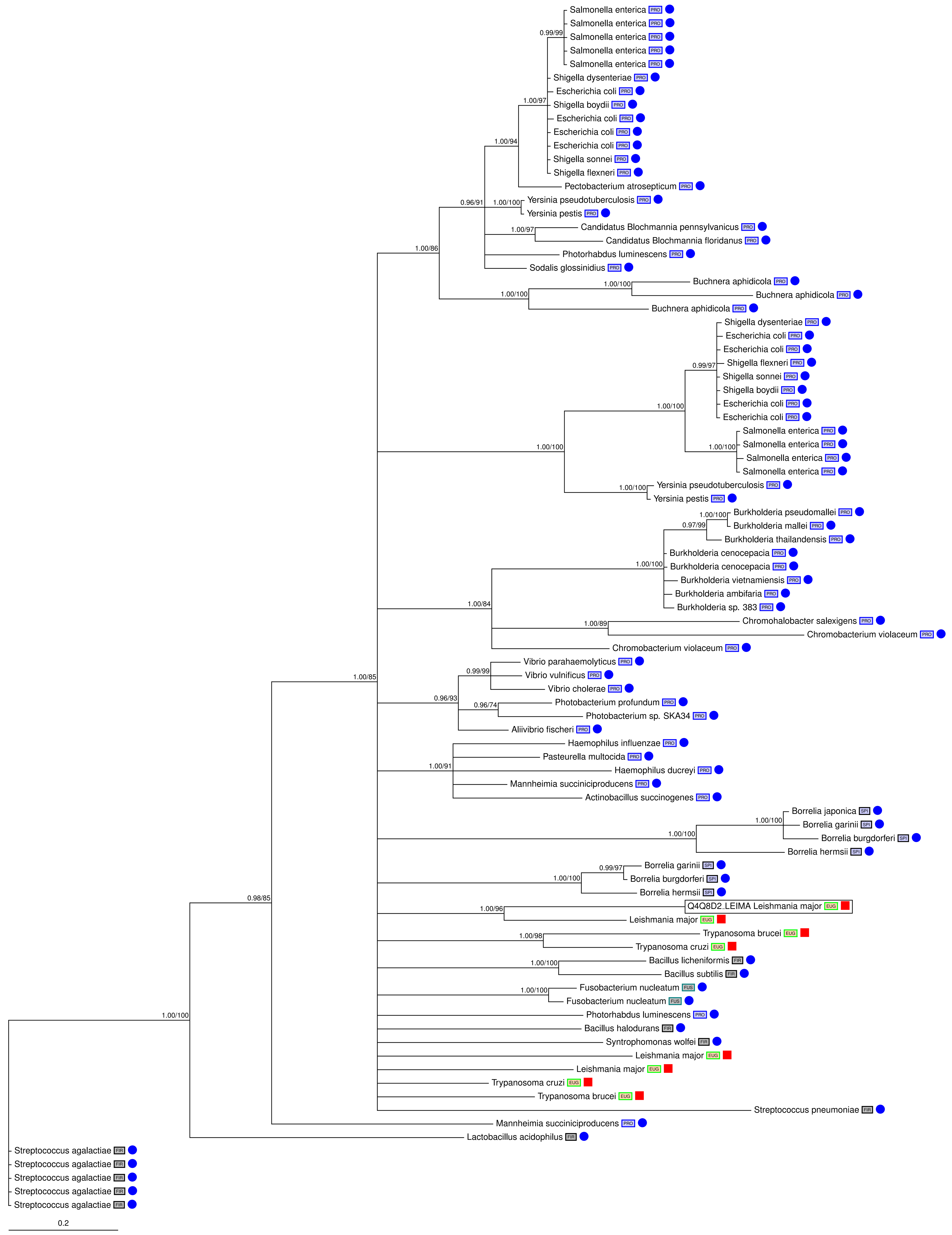

TN009

```
              Candy accession: Q4Q9X3_LEIMA
             RefSeq accession: XP_001683875.1
            Uniprot accession: Q4Q9X3_LEIMA
                     Comments: LGT - KINETOPLASTIDS ONLY
             Species affected: TB, LM
        Adjacent taxa in tree: Bacteria
EC annotation - (Blast/Profile): EC:2.1.1.37
                   PHOBIUS SP: 0
                  PHOBIUS TMD: 0
            RefSeq annotation: modification methylase-like protein
      Name of enzyme/protein: DNA (cytosine-5-)-methyltransferase
      KEGG PATHWAY - level 1: Amino Acid Metabolism
      KEGG PATHWAY - level 2: Cysteine and methionine metabolism
```

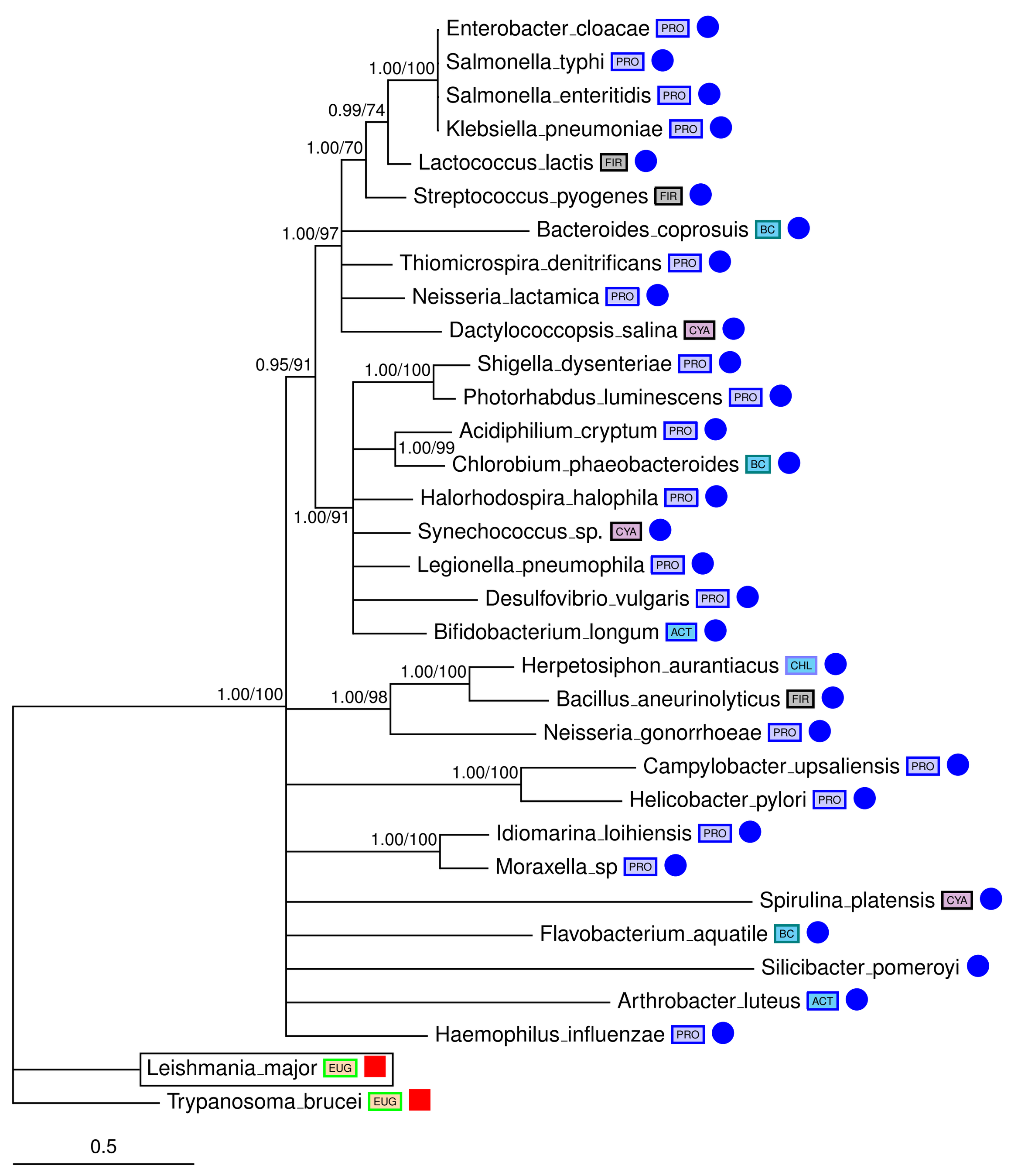

TN010

Candy accession:	Q4QBB1_LEIMA
RefSeq accession:	XP_001683387.1
Uniprot accession:	Q4QBB1_LEIMA
Comments:	LGT - LM ONLY
Species affected:	LM
Adjacent taxa in tree:	Bacteria
EC annotation - (Blast/Profile):	EC:6.1.1.20
PHOBIUS SP:	0
PHOBIUS TMD:	0
RefSeq annotation:	hypothetical protein
Name of enzyme/protein:	phenylalanyl-tRNA synthetase beta chain
KEGG PATHWAY - level 1:	Translation - Genetic Information Processing
KEGG PATHWAY - level 2:	Aminoacyl-tRNA biosynthesis

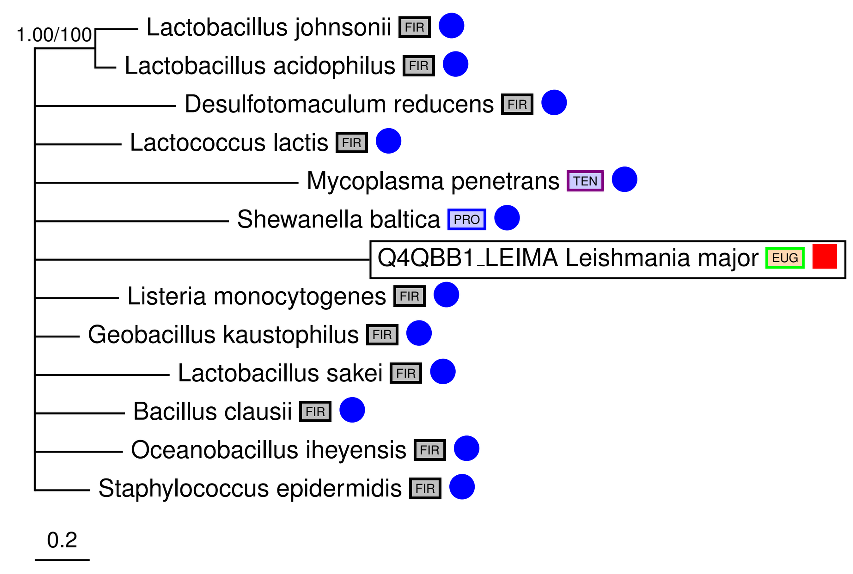

TN011

```
                Candy accession: Q4QFB1_LEIMA
               RefSeq accession: XP_001681987.1
              Uniprot accession: Q4QFB1_LEIMA
                       Comments: LGT - LM ONLY
               Species affected: LM
           Adjacent taxa in tree: Bacteria
EC annotation - (Blast/Profile): na
                     PHOBIUS SP: 0
                    PHOBIUS TMD: 4
              RefSeq annotation: hypothetical protein
        Name of enzyme/protein: Protein containing DUF2871
         KEGG PATHWAY - level 1: Function unknown
         KEGG PATHWAY - level 2: na
```

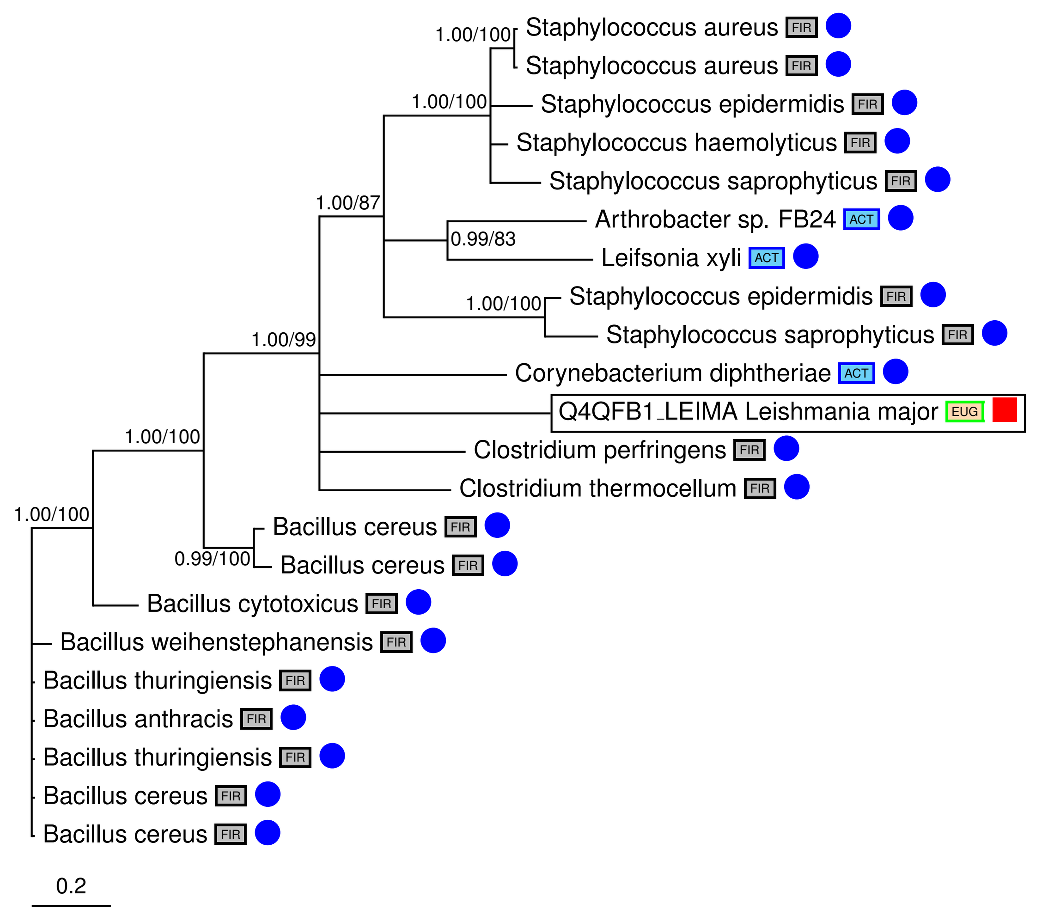

TN012

```
              Candy accession: Q4QFD4_LEIMA
             RefSeq accession: XP_001681964.1
            Uniprot accession: ECOT2_LEIMA
                     Comments: LGT - KINETOPLASTIDS ONLY
             Species affected: LM,TB,TC
        Adjacent taxa in tree: Bacteria
EC annotation - (Blast/Profile): na
                  PHOBIUS SP: 0
                 PHOBIUS TMD: 0
             RefSeq annotation: ecotin
      Name of enzyme/protein: ecotin
       KEGG PATHWAY - level 1: Other function
       KEGG PATHWAY - level 2: na
```

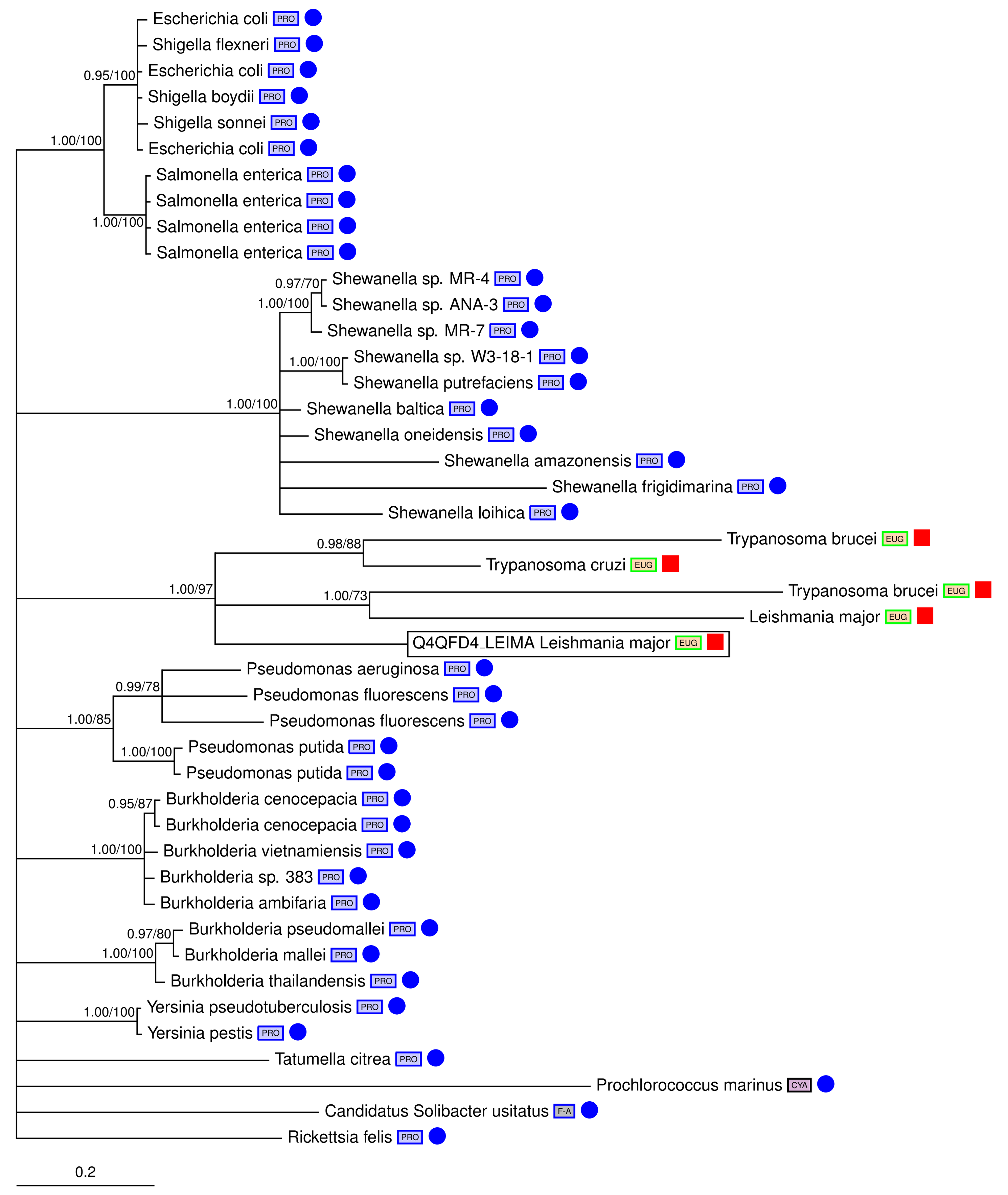

TN013

```
             Candy accession: Q4QGE5_LEIMA
            RefSeq accession: XP_001681753.1
           Uniprot accession: Q4QGE5_LEIMA
                    Comments: LGT - KINETOPLASTIDS ONLY
            Species affected: LM,TB,TC
         Adjacent taxa in tree: Prokaryotes
EC annotation - (Blast/Profile): EC:3.4.17.19
                  PHOBIUS SP: 0
                 PHOBIUS TMD: 0
           RefSeq annotation: carboxypeptidase; metallo-peptidase,
                              Clan MA(E), family 32
     Name of enzyme/protein: Metallo-peptidase, Clan MA(E), family 32
       KEGG PATHWAY - level 1: Reaction
       KEGG PATHWAY - level 2: Reaction
```

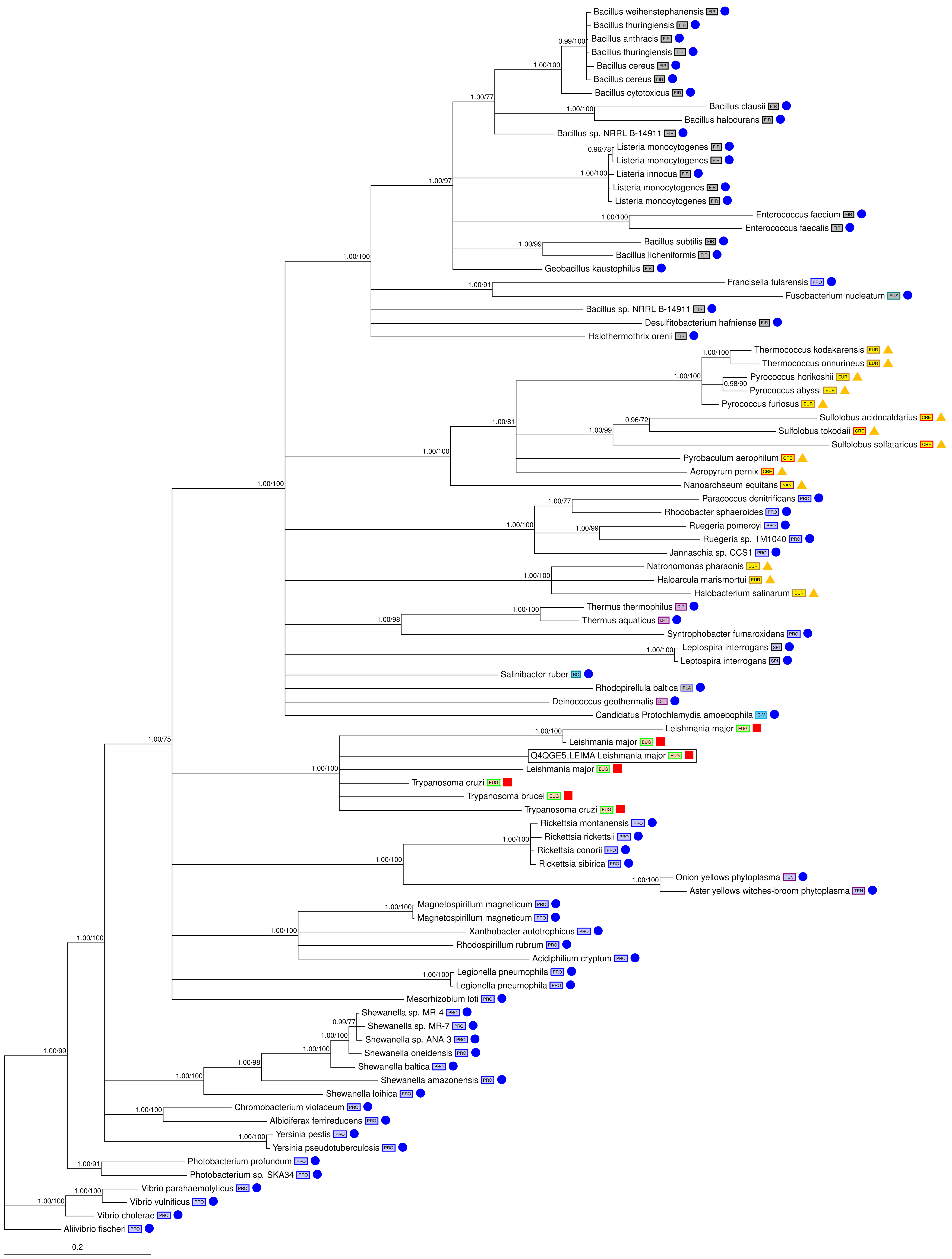

TN014

```
             Candy accession: Q4QGE9_LEIMA
            RefSeq accession: XP_001681749.1
           Uniprot accession: Q4QGE9_LEIMA
                    Comments: LGT - LM ONLY
            Species affected: LM
      Adjacent taxa in tree: Prokaryotes
EC annotation - (Blast/Profile): EC:3.1.26.4
                  PHOBIUS SP: 0
                 PHOBIUS TMD: 0
           RefSeq annotation: ribonuclease hii
    Name of enzyme/protein: ribonuclease HII
     KEGG PATHWAY - level 1: Reaction
     KEGG PATHWAY - level 2: Reaction
```

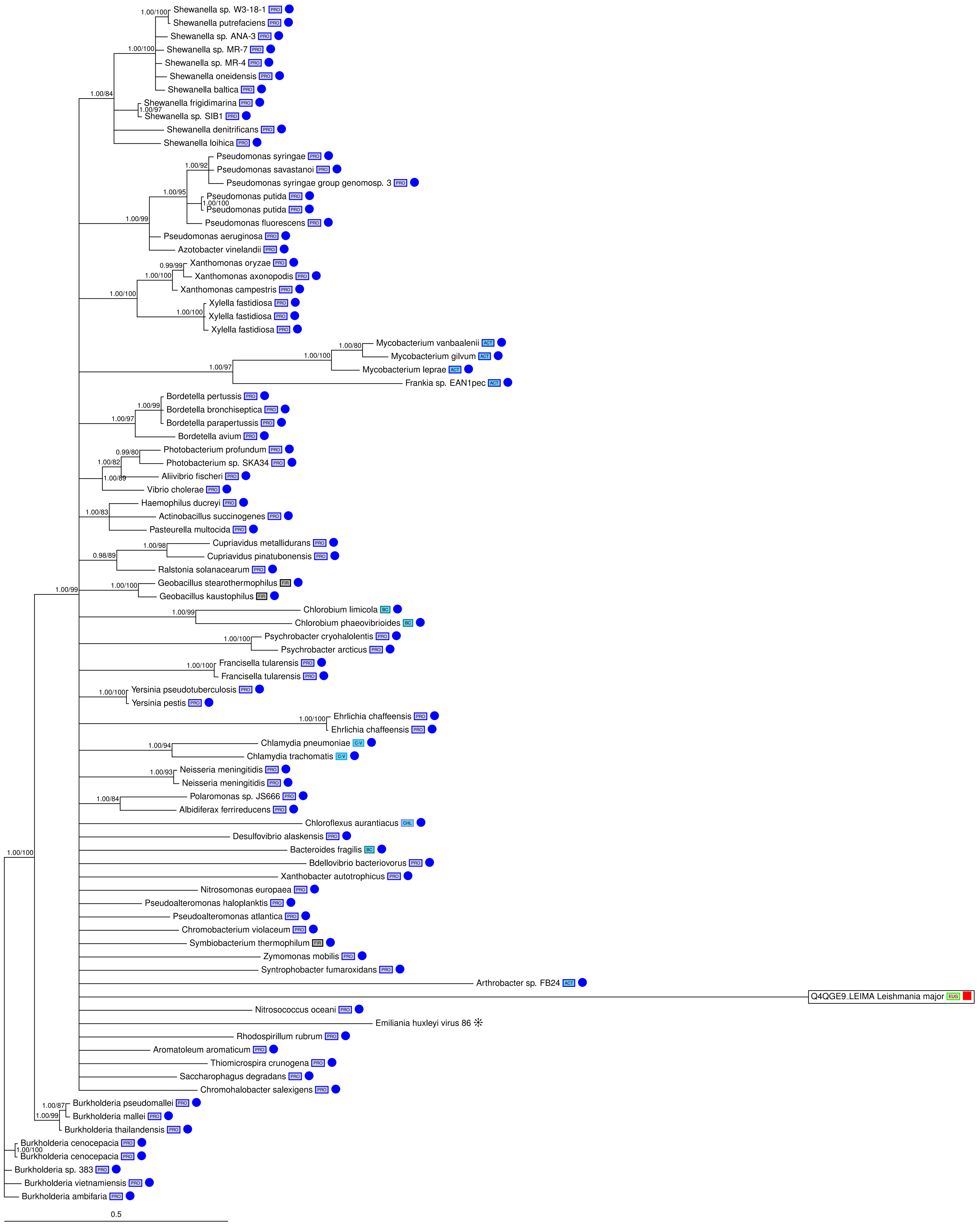

TN015

```
              Candy accession: Q4QH87_LEIMA
             RefSeq accession: XP_001681461.1
            Uniprot accession: Q4QH87_LEIMA
                     Comments: LGT - LM ONLY
             Species affected: LM
        Adjacent taxa in tree: Bacteria
EC annotation - (Blast/Profile): na
                   PHOBIUS SP: 0
                  PHOBIUS TMD: 0
            RefSeq annotation: hypothetical protein
      Name of enzyme/protein: Protein containing DUF1861
       KEGG PATHWAY - level 1: Function unknown
       KEGG PATHWAY - level 2: na
```

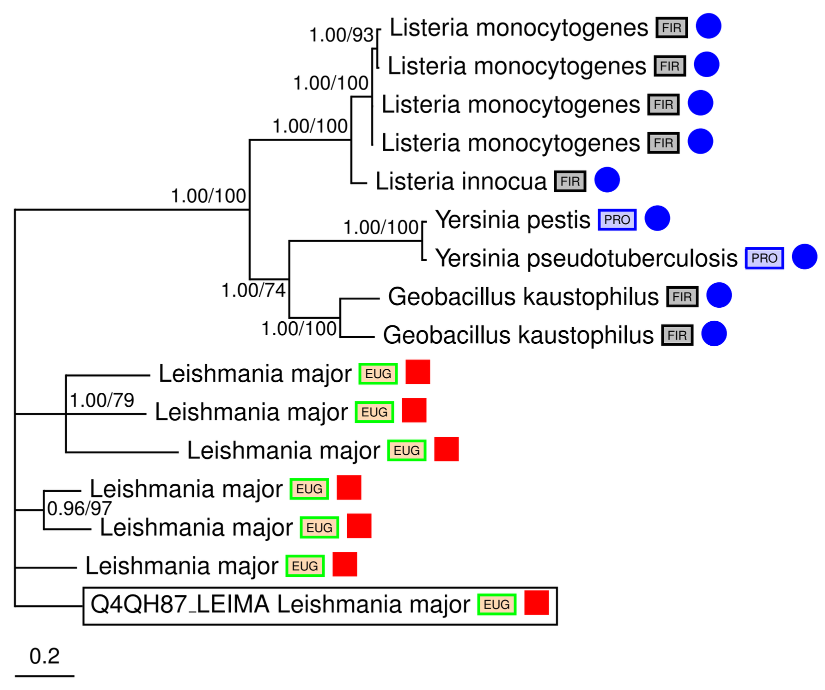

TN016

```
                Candy accession: Q4QIU1_LEIMA
               RefSeq accession: XP_001680907.1
              Uniprot accession: Q4QIU1_LEIMA
                       Comments: LGT - KINETOPLASTIDS ONLY
               Species affected: LM,TB,TC
          Adjacent taxa in tree: Proteobacteria
EC annotation - (Blast/Profile): EC:3.5.1.16
                     PHOBIUS SP: 0
                    PHOBIUS TMD: 0
               RefSeq annotation: hypothetical protein
        Name of enzyme/protein: acetylornithine deacetylase
       KEGG PATHWAY - level 1: Amino Acid Metabolism
       KEGG PATHWAY - level 2: Arginine and proline metabolism
```

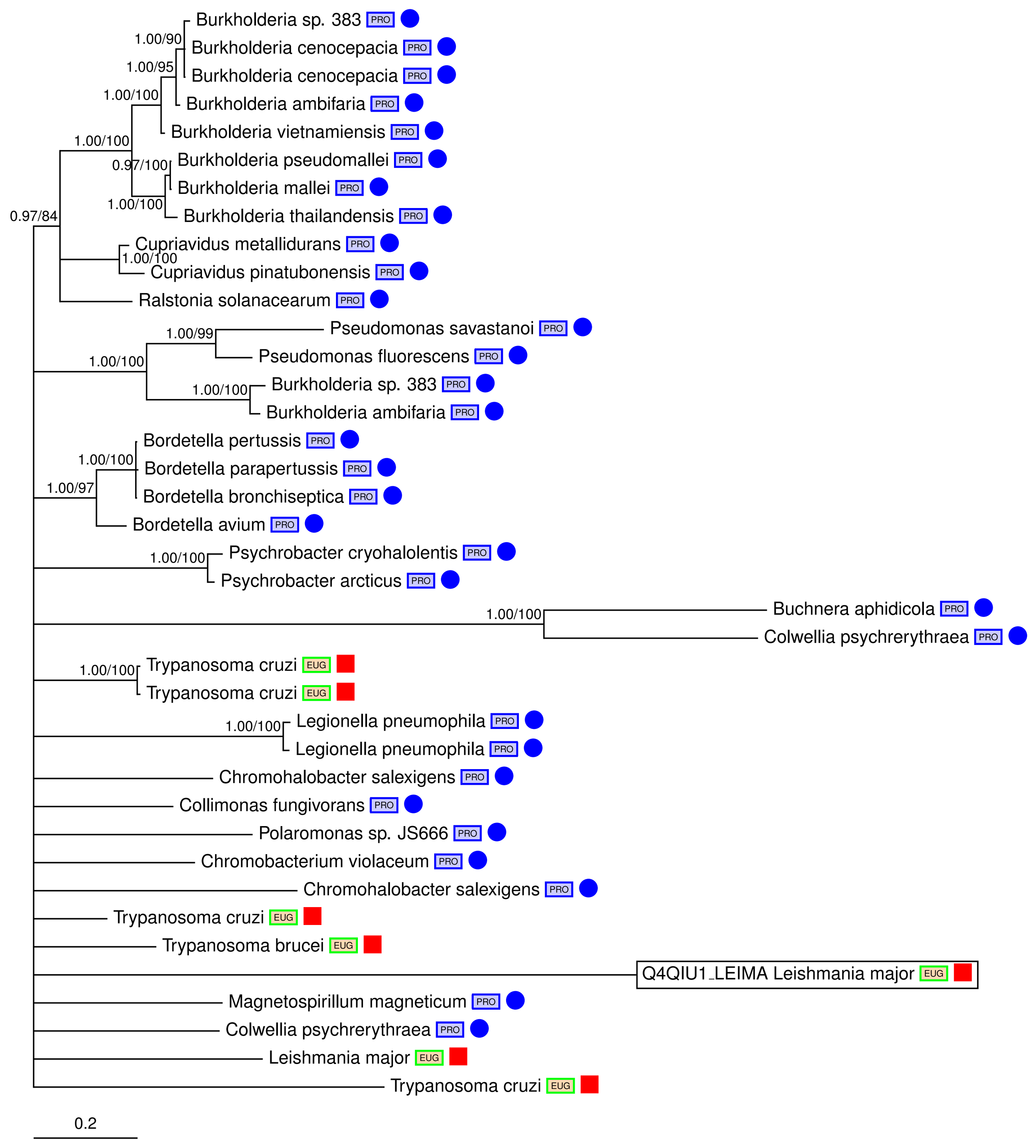

TN017

```
            Candy accession: Q4QIU7_LEIMA
           RefSeq accession: XP_001680901.1
          Uniprot accession: Q4QIU7_LEIMA
                   Comments: LGT - LM ONLY
           Species affected: LM
     Adjacent taxa in tree: Proteobacteria
EC annotation - (Blast/Profile): EC:1.3.3.4
                 PHOBIUS SP: 0
                PHOBIUS TMD: 1
          RefSeq annotation: protoporphyrinogen oxidase-like protein
     Name of enzyme/protein: protoporphyrinogen oxidase
     KEGG PATHWAY - level 1: Metabolism of Cofactors and Vitamins
     KEGG PATHWAY - level 2: Porphyrin and chlorophyll metabolism
```

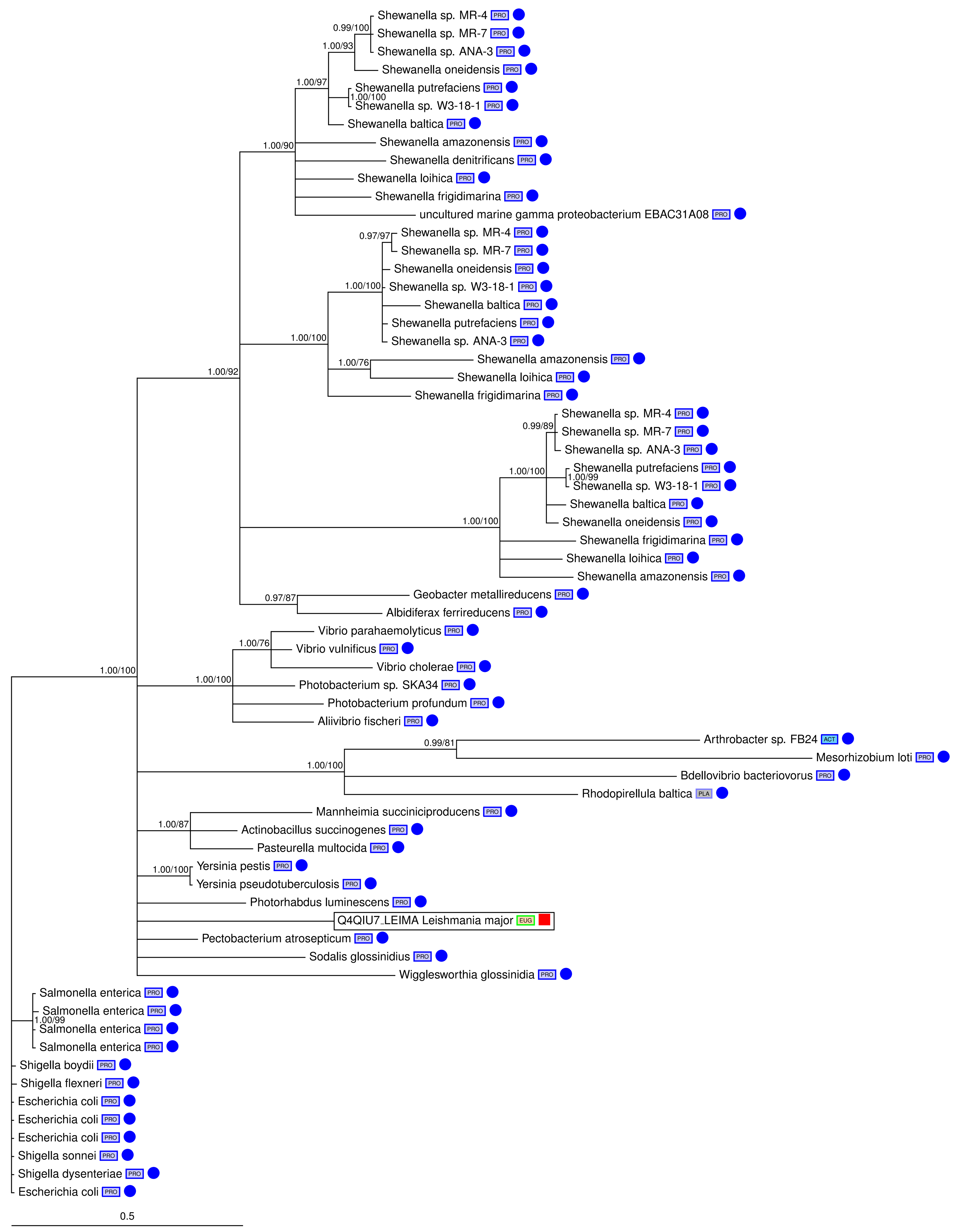

TN018

```
                 Candy accession: Q5XM24_DICDI
                RefSeq accession: XP_640566.1
               Uniprot accession: APRA_DICDI
                        Comments: LGT - DD ONLY
                Species affected: DD
           Adjacent taxa in tree: Bacteria
EC annotation - (Blast/Profile): na
                      PHOBIUS SP: Y
                     PHOBIUS TMD: 0
               RefSeq annotation: PhoPQ-activated pathogenicity-related
                                  protein
          Name of enzyme/protein: PhoPQ-activated pathogenicity-related
                                  protein
         KEGG PATHWAY - level 1: Other function - Signal Transduction
         KEGG PATHWAY - level 2: na
```

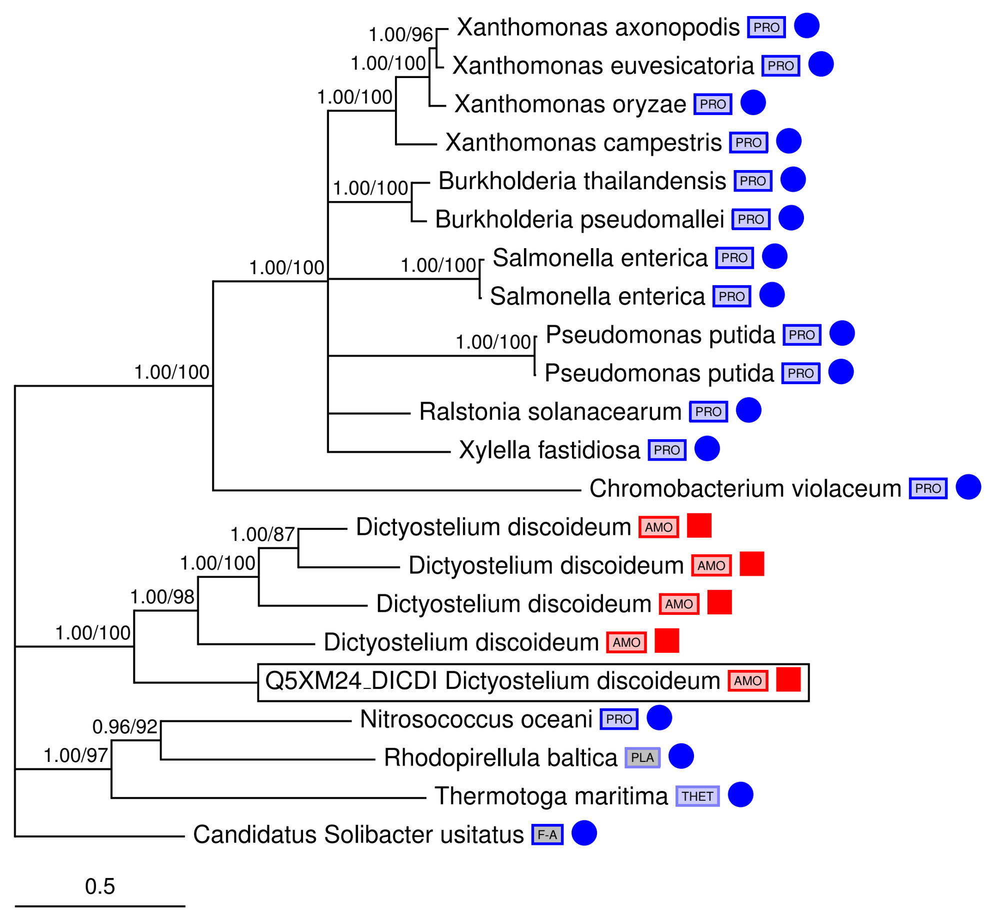

TN019

```
                 Candy accession: Q7R5L6_GIALA
                RefSeq accession: XP_001707536.1
               Uniprot accession: A8BDW4_GIALA
                        Comments: LGT - GI ONLY
                Species affected: GI
           Adjacent taxa in tree: Firmicutes
EC annotation - (Blast/Profile): na
                      PHOBIUS SP: 0
                     PHOBIUS TMD: 0
               RefSeq annotation: Transglutaminase/protease, putative
          Name of enzyme/protein: Transglutaminase/protease, putative
          KEGG PATHWAY - level 1: Other function
          KEGG PATHWAY - level 2: na
```

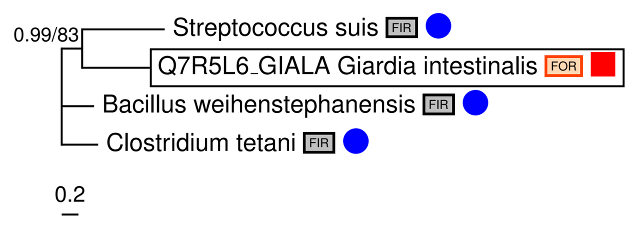

TN020

```
                 Candy accession: Q7R6X5_PLAYO
                RefSeq accession: XP_728783.1
               Uniprot accession: Q7R6X5_PLAYO
                        Comments: LGT - PY ONLY
                Species affected: PY
           Adjacent taxa in tree: Bacteria
EC annotation - (Blast/Profile): na
                      PHOBIUS SP: 0
                     PHOBIUS TMD: 0
               RefSeq annotation: hypothetical protein
         Name of enzyme/protein: Putative ribosomal subunit interface
                                 protein; sigma-54 modulation protein
                                 family
          KEGG PATHWAY - level 1: Other function - Genetic Information
                                 Processing
          KEGG PATHWAY - level 2: na
```

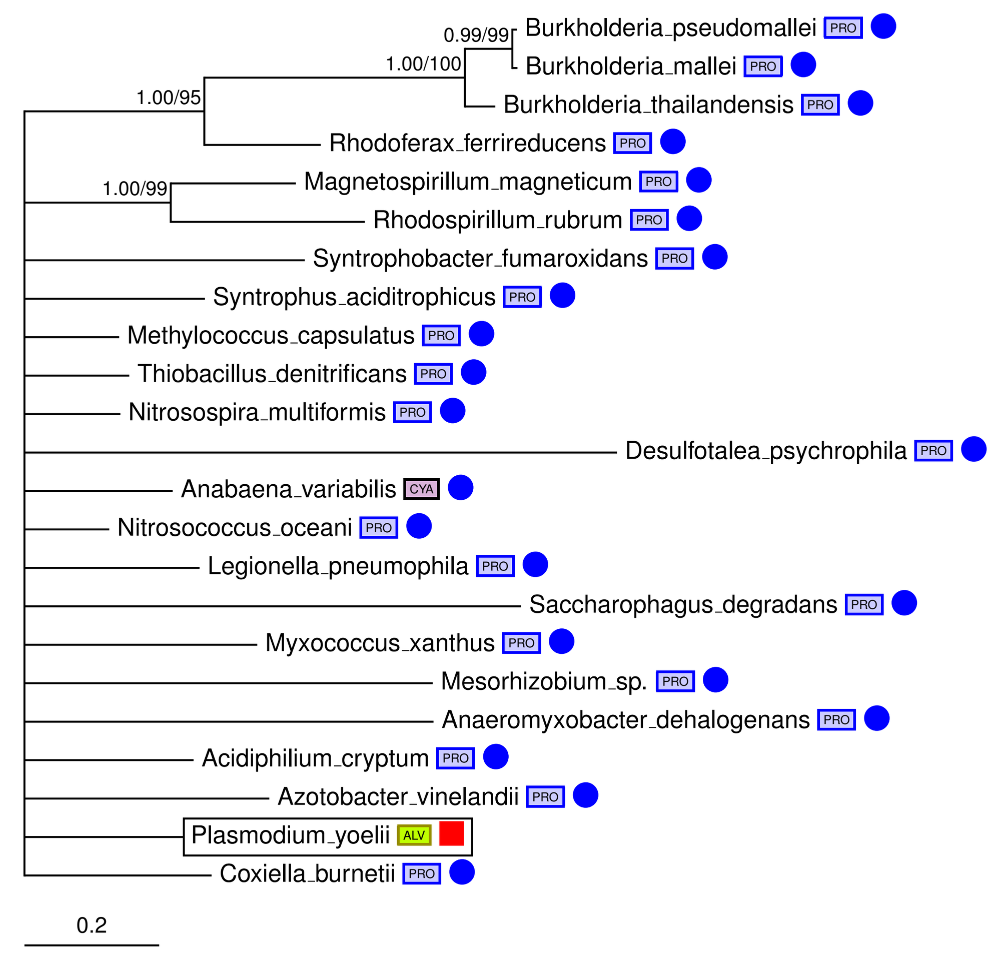

TN021

```
              Candy accession: Q7R6Y3_PLAYO
             RefSeq accession: XP_728775.1
            Uniprot accession: Q7R6Y3_PLAYO
                     Comments: LGT - PY ONLY
             Species affected: PY
        Adjacent taxa in tree: Proteobacteria
EC annotation - (Blast/Profile): na
                   PHOBIUS SP: 0
                  PHOBIUS TMD: 0
            RefSeq annotation: hypothetical protein
       Name of enzyme/protein: Hypothetical protein
       KEGG PATHWAY - level 1: Function unknown
       KEGG PATHWAY - level 2: na
```

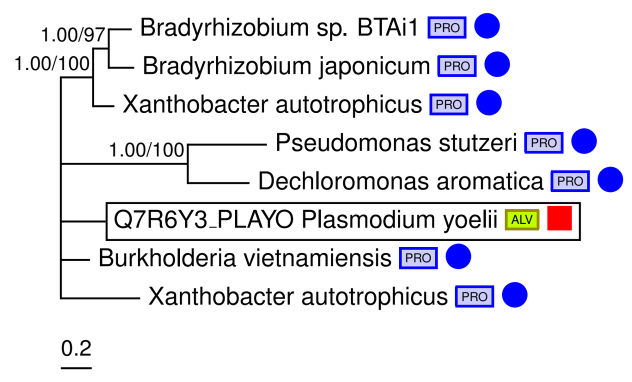

TN022

```
            Candy accession: Q7R799_PLAYO
           RefSeq accession: XP_728635.1
          Uniprot accession: Q7R799_PLAYO
                   Comments: LGT - PY ONLY
           Species affected: PY
       Adjacent taxa in tree: Bacteria
EC annotation - (Blast/Profile): na
                 PHOBIUS SP: 0
                PHOBIUS TMD: 0
          RefSeq annotation: hypothetical protein
     Name of enzyme/protein: Protein containing DUF2699
     KEGG PATHWAY - level 1: Function unknown
     KEGG PATHWAY - level 2: na
```

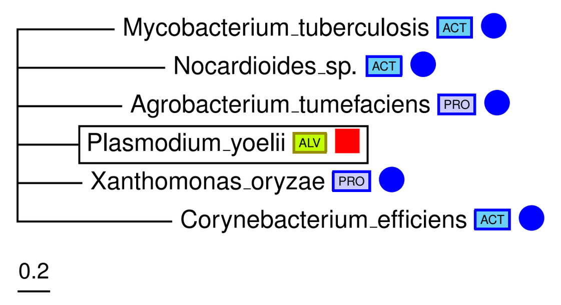

TN023

```
              Candy accession: Q8SS83_ENCCU
             RefSeq accession: NP_597635.1
            Uniprot accession: KCY_ENCCU
                     Comments: LGT - EC ONLY
             Species affected: EC
        Adjacent taxa in tree: Bacteria
EC annotation - (Blast/Profile): EC:2.7.4.14
                    PHOBIUS SP: 0
                   PHOBIUS TMD: 0
            RefSeq annotation: CYTIDYLATE KINASE
       Name of enzyme/protein: cytidylate kinase
       KEGG PATHWAY - level 1: Metabolism of Cofactors and Vitamins,
                               Metabolism of Other Amino Acids,
                               Nucleotide Metabolism
       KEGG PATHWAY - level 2: Pyrimidine metabolism, beta-Alanine
                               metabolism
```

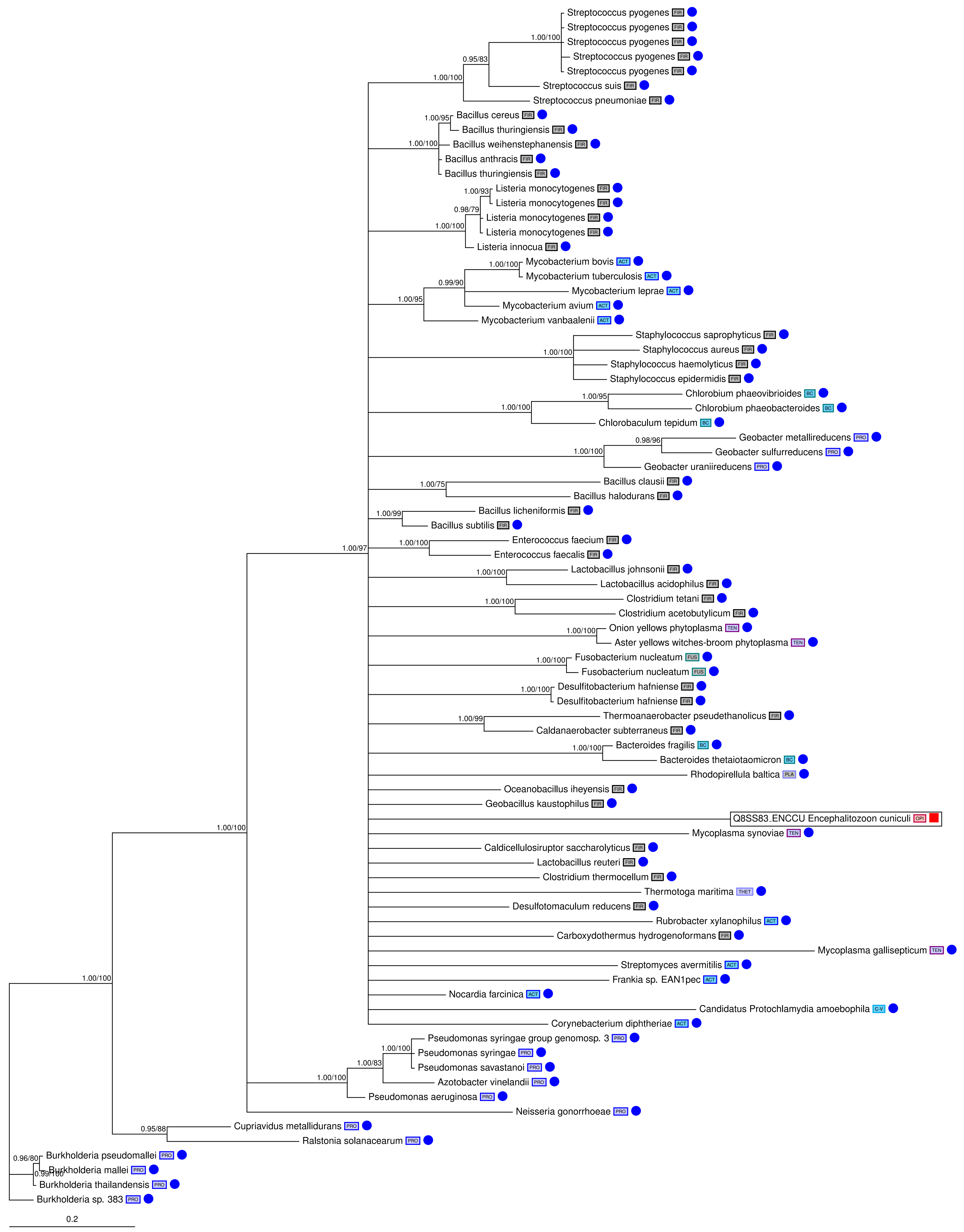

TN024

```
             Candy accession: Q8T2H6_DICDI
            RefSeq accession: XP_642591.1
           Uniprot accession: Q8T2H6_DICDI
                    Comments: LGT - DD ONLY
            Species affected: DD
       Adjacent taxa in tree: Achraea - Haloarcula
EC annotation - (Blast/Profile): na
                  PHOBIUS SP: 0
                 PHOBIUS TMD: 0
           RefSeq annotation: hypothetical protein DDB_G0277409
     Name of enzyme/protein: Protein containing DUF162
      KEGG PATHWAY - level 1: Function unknown
      KEGG PATHWAY - level 2: na
```

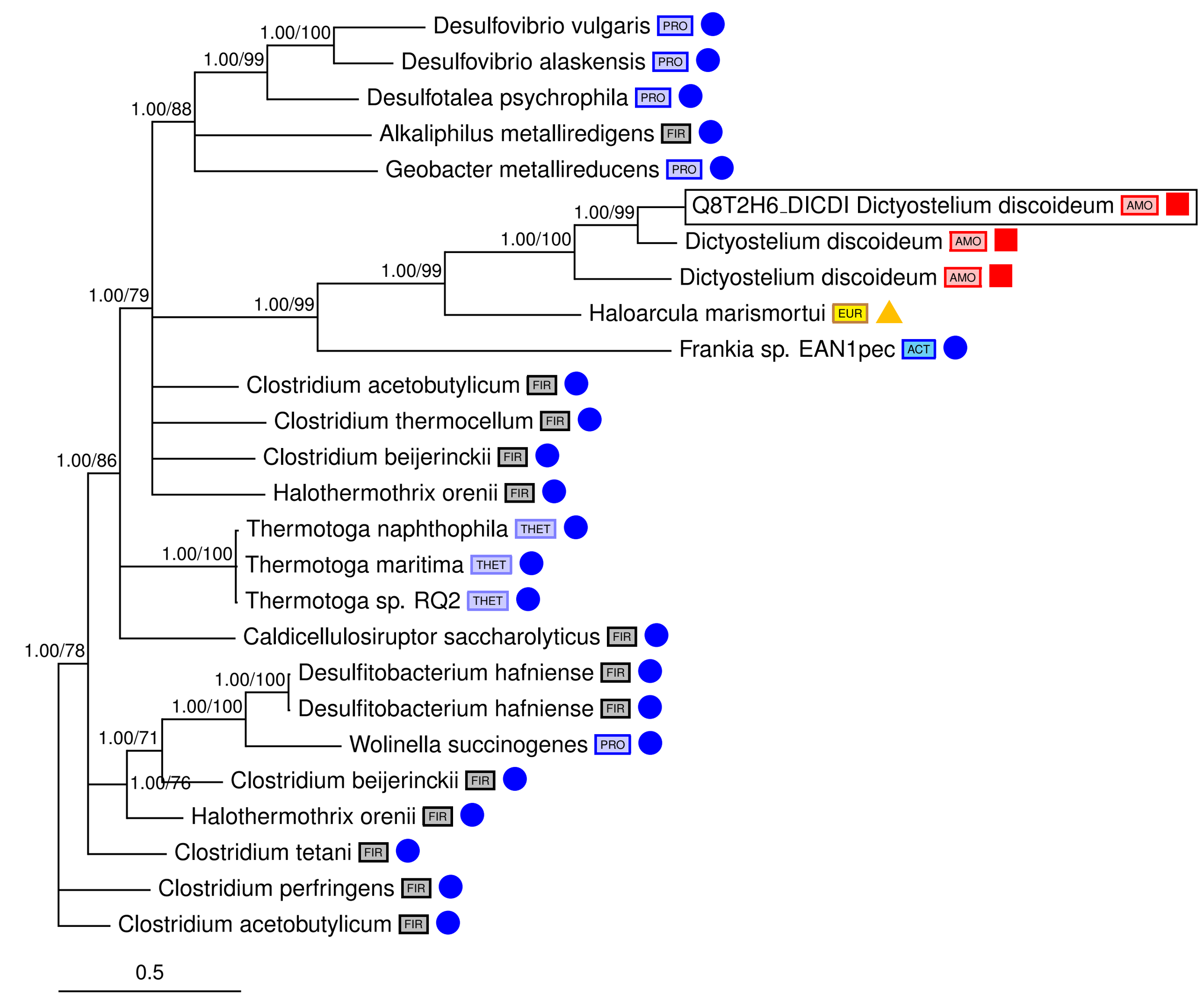

TN025

```
                Candy accession: Q9BHG3_LEIMA
               RefSeq accession: XP_001685059.1
              Uniprot accession: Q9BHG3_LEIMA
                       Comments: LGT - KINETOPLASTIDS ONLY - THE PROTEINS
                                 POSSESS THE BACTERIAL CEST DOMAIN HENCE
                                 IT IS CONSIDERED AS A CANDIDATE B->E LGT
               Species affected: LM,TB,TC
           Adjacent taxa in tree: Chlamydiae/Verrucomicrobia
EC annotation - (Blast/Profile): na
                    PHOBIUS SP: 0
                   PHOBIUS TMD: 0
              RefSeq annotation: hypothetical protein
         Name of enzyme/protein: Secretion chaperone CesT
         KEGG PATHWAY - level 1: Other function - Genetic Information
                                 Processing
         KEGG PATHWAY - level 2: na
```

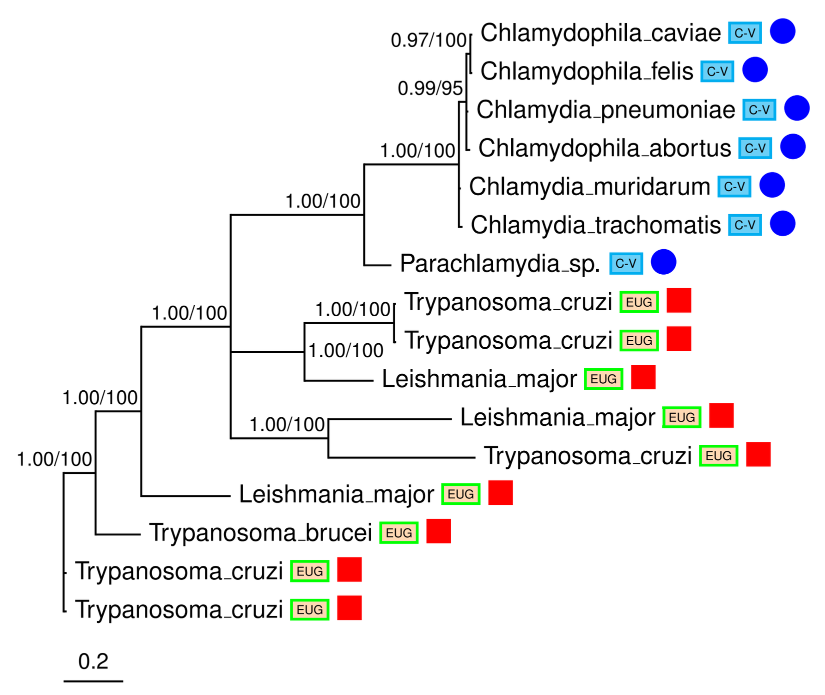

TN026

```
               Candy accession: Q9NKQ8_LEIMA
              RefSeq accession: XP_001687362.1
             Uniprot accession: Q9NKQ8_LEIMA
                      Comments: LGT - LM ONLY
              Species affected: LM
         Adjacent taxa in tree: Bacteria
EC annotation - (Blast/Profile): EC:4.2.1.75
                    PHOBIUS SP: 0
                   PHOBIUS TMD: 0
            RefSeq annotation: hypothetical protein
       Name of enzyme/protein: uroporphyrinogen-III synthase
       KEGG PATHWAY - level 1: Metabolism of Cofactors and Vitamins
       KEGG PATHWAY - level 2: Porphyrin and chlorophyll metabolism
```

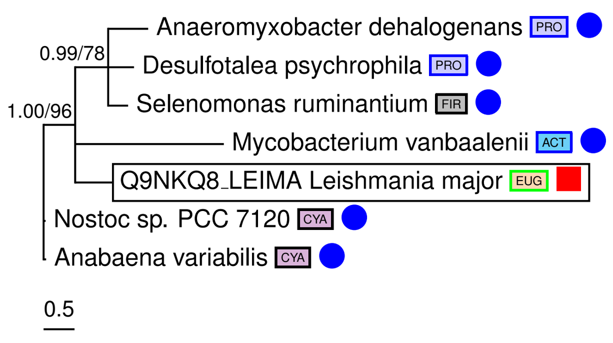

TN027

```
                Candy accession: Q38CZ3_9TRYP
               RefSeq accession: XP_827657.1
              Uniprot accession: Q38CZ3_9TRYP
                       Comments: LGT - KINETOPLASTIDS ONLY - ADDITIONAL
                                 BACTERIA TAXA ARE RECOVERED BUT
                                 COREPSOND TO MUCH WEAKER HITS HENCE IT
                                 IS CONSIDERED AS A CANDIDATE B->E LGT
               Species affected: LM,TB,TC
          Adjacent taxa in tree: Chlamydiae/Verrucomicrobia
EC annotation - (Blast/Profile): na
                     PHOBIUS SP: 0
                    PHOBIUS TMD: 0
              RefSeq annotation: hypothetical protein
         Name of enzyme/protein: Predicted Rossmann fold nucleotide-
                                 binding protein
         KEGG PATHWAY - level 1: Other function - Genetic Information
                                 Processing
         KEGG PATHWAY - level 2: na
```

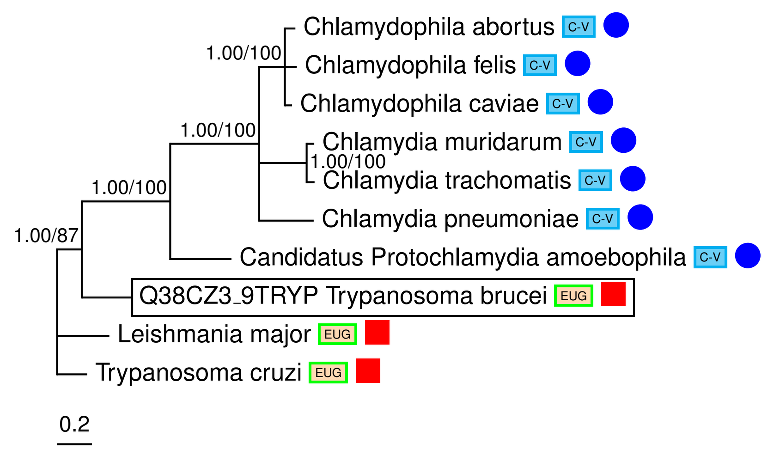

TN028

```
                Candy accession: Q50LR6_ENTHI
               RefSeq accession: XP_650165.1
              Uniprot accession: C4MAL3_ENTHI
                       Comments: LGT - EH ONLY
               Species affected: EH
          Adjacent taxa in tree: Bacteria
EC annotation - (Blast/Profile): na
                     PHOBIUS SP: 0
                    PHOBIUS TMD: 0
              RefSeq annotation: hypothetical protein
         Name of enzyme/protein: Predicted tRNA 2'-O-methylase; Predicted
                                 HD superfamily hydrolase
         KEGG PATHWAY - level 1: Other function - Genetic Information
                                 Processing
         KEGG PATHWAY - level 2: na
```

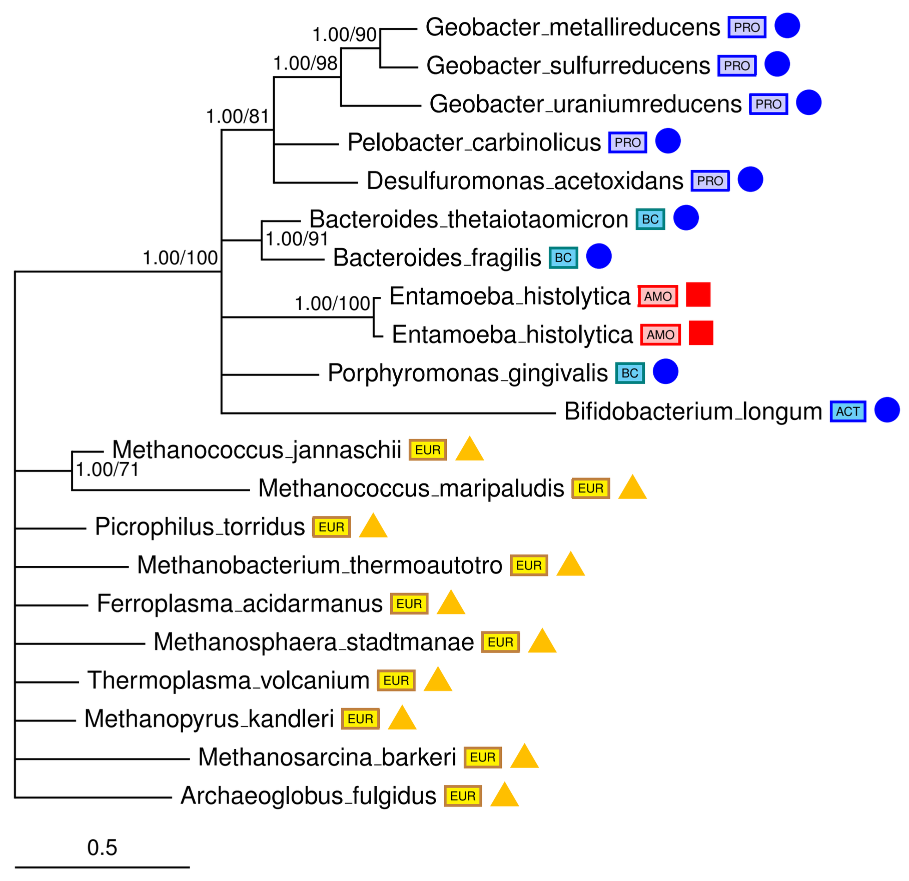

TN029

```
                 Candy accession: Q50SB1_ENTHI
                RefSeq accession: XP_649873.2
               Uniprot accession: C4M1J6_ENTHI
                        Comments: LGT - EH ONLY
                Species affected: EH
          Adjacent taxa in tree: Firmicutes
EC annotation - (Blast/Profile): na
                      PHOBIUS SP: 0
                     PHOBIUS TMD: 0
              RefSeq annotation: hypothetical protein
         Name of enzyme/protein: Protein containing DUF1963
         KEGG PATHWAY - level 1: Function unknown
         KEGG PATHWAY - level 2: na
```

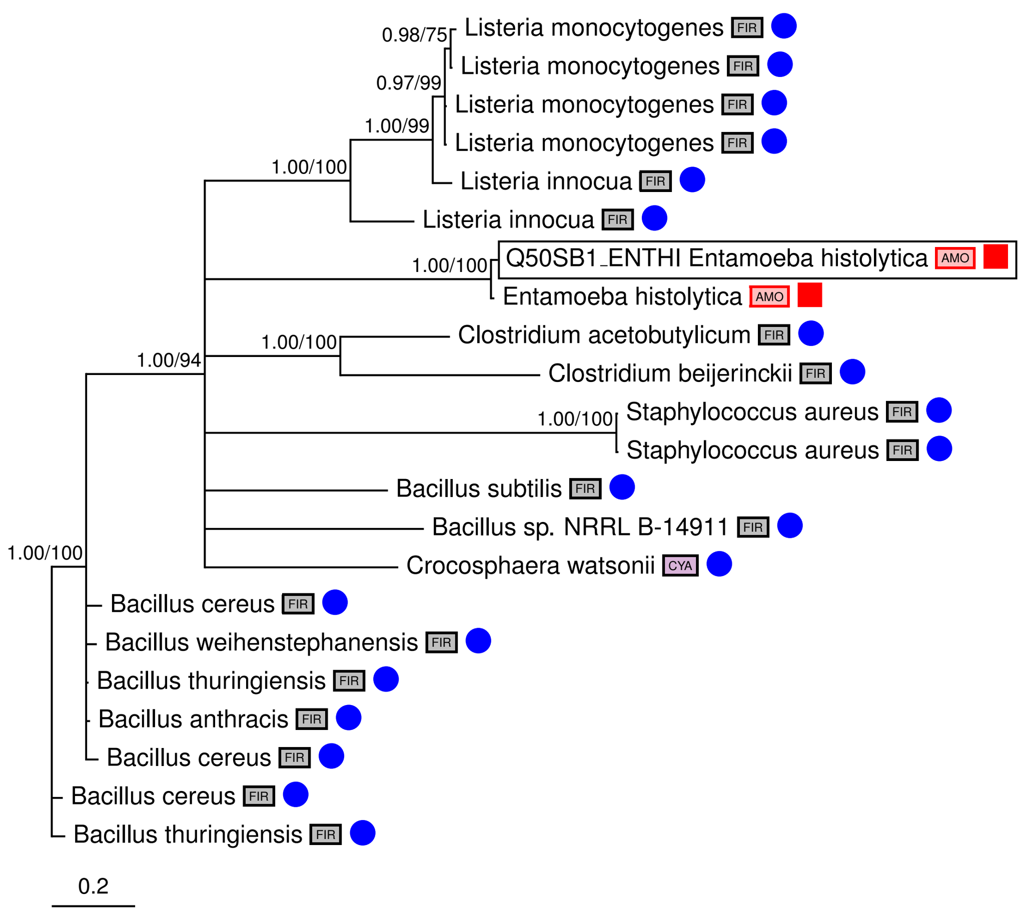

TN030

```
               Candy accession: Q50VJ7_ENTHI
              RefSeq accession: XP_651004.1
             Uniprot accession: C4MBP5_ENTHI
                      Comments: LGT - EH ONLY
              Species affected: EH
          Adjacent taxa in tree: Bacteroides
EC annotation - (Blast/Profile): na
                    PHOBIUS SP: 0
                   PHOBIUS TMD: 0
             RefSeq annotation: hypothetical protein
        Name of enzyme/protein: Protein containing DUF1810
        KEGG PATHWAY - level 1: Function unknown
        KEGG PATHWAY - level 2: na
```

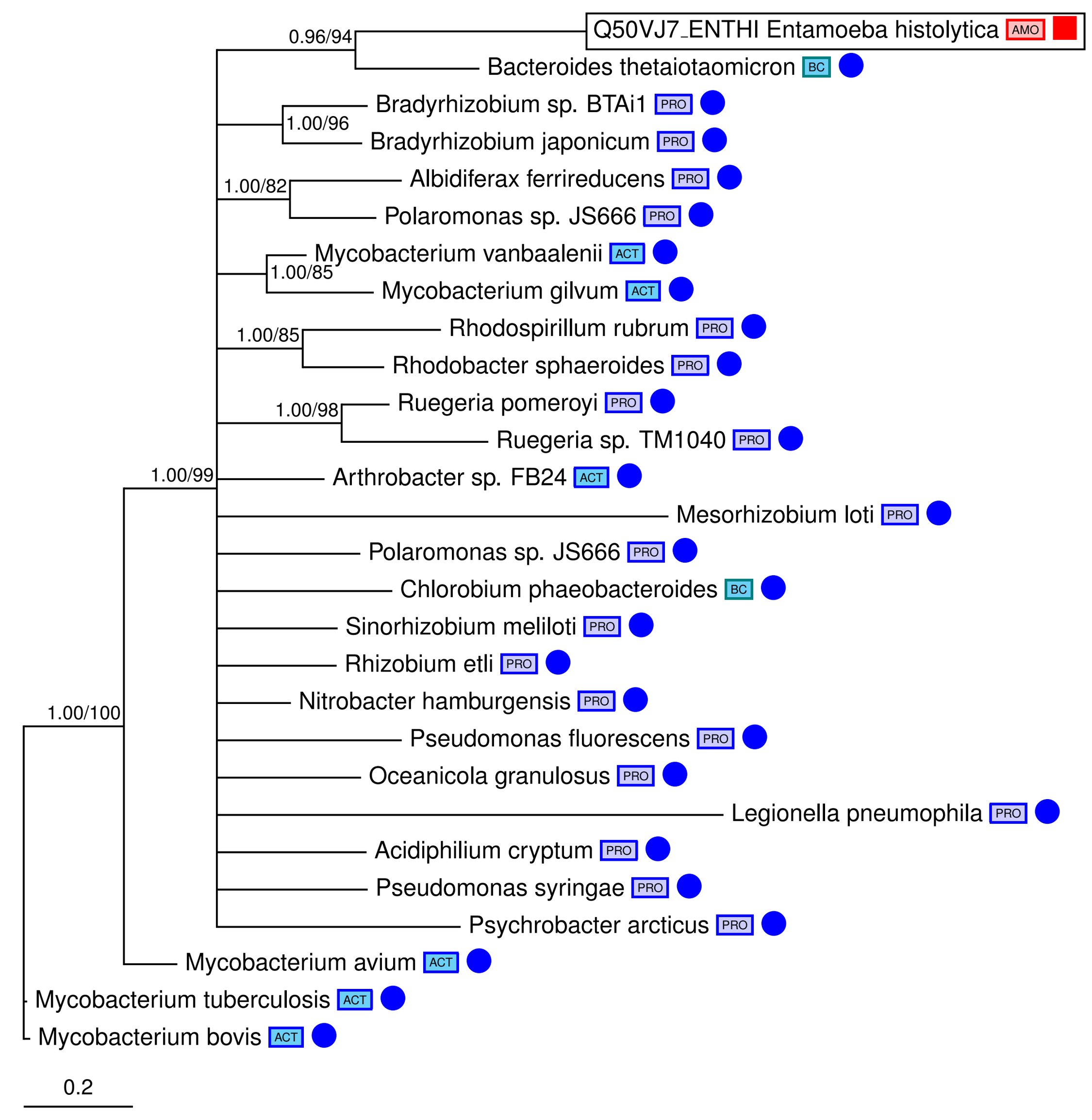

TN031

```
            Candy accession: Q50WY8_ENTHI
           RefSeq accession: XP_651498.1
          Uniprot accession: C4M560_ENTHI
                   Comments: LGT - EH ONLY
           Species affected: EH
       Adjacent taxa in tree: Bacteria
EC annotation - (Blast/Profile): EC:4.6.1.1
                 PHOBIUS SP: 0
                PHOBIUS TMD: 0
          RefSeq annotation: hypothetical protein
     Name of enzyme/protein: adenylate cyclase
     KEGG PATHWAY - level 1: Nucleotide Metabolism, Signal
                             Transduction
     KEGG PATHWAY - level 2: Purine metabolism - several other
                             entries
```

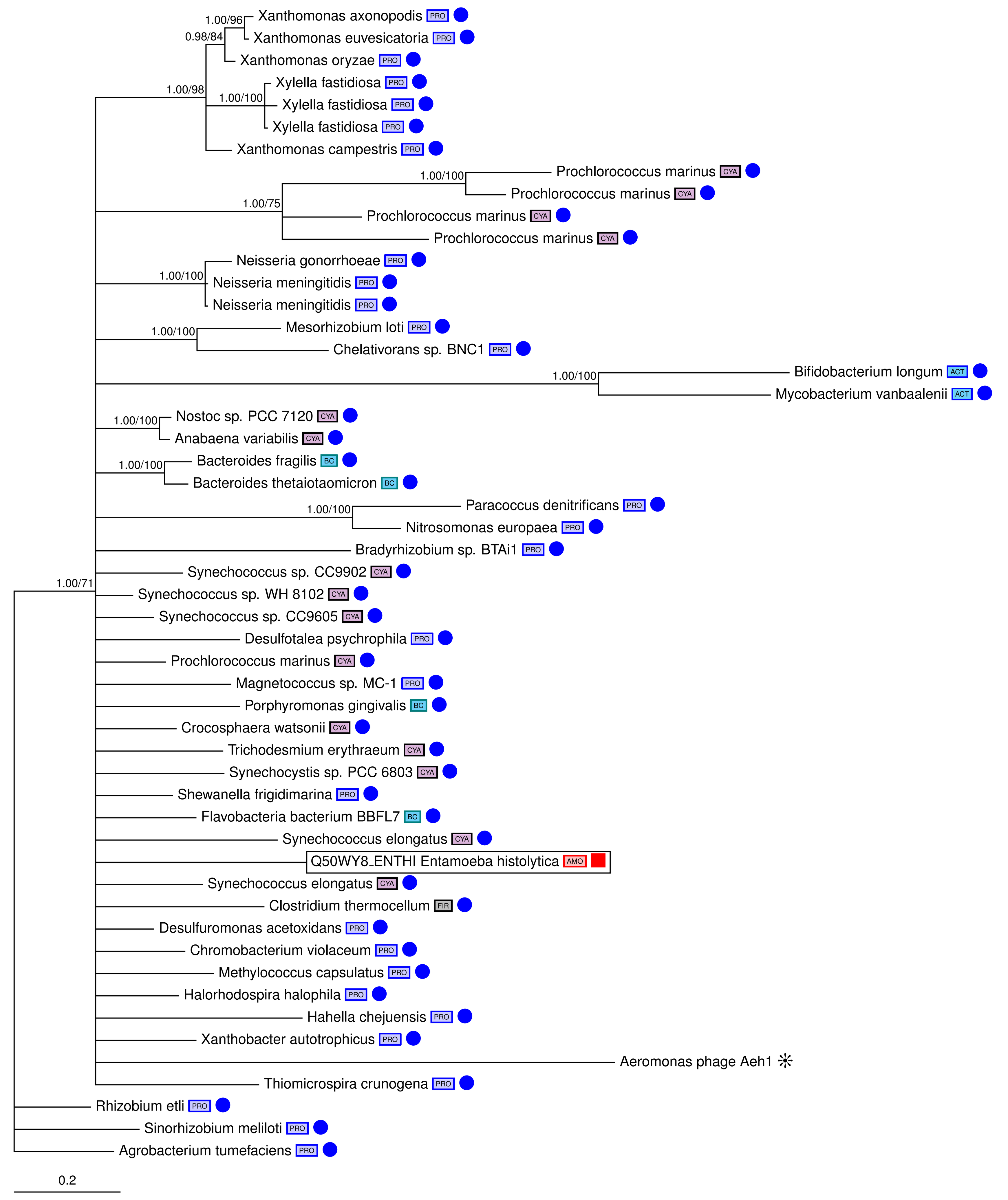

TN032

```
                  Candy accession: Q50XL0_ENTHI
                 RefSeq accession: XP_651699.1
                Uniprot accession: C4M0V4_ENTHI
                         Comments: LGT - EH ONLY
                 Species affected: EH
            Adjacent taxa in tree: Bacteria
EC annotation - (Blast/Profile): na
                       PHOBIUS SP: 0
                      PHOBIUS TMD: 0
                RefSeq annotation: hypothetical protein
          Name of enzyme/protein: Protein containing cupin-like fold and
                                   unknown function
          KEGG PATHWAY - level 1: Function unknown
          KEGG PATHWAY - level 2: na
```

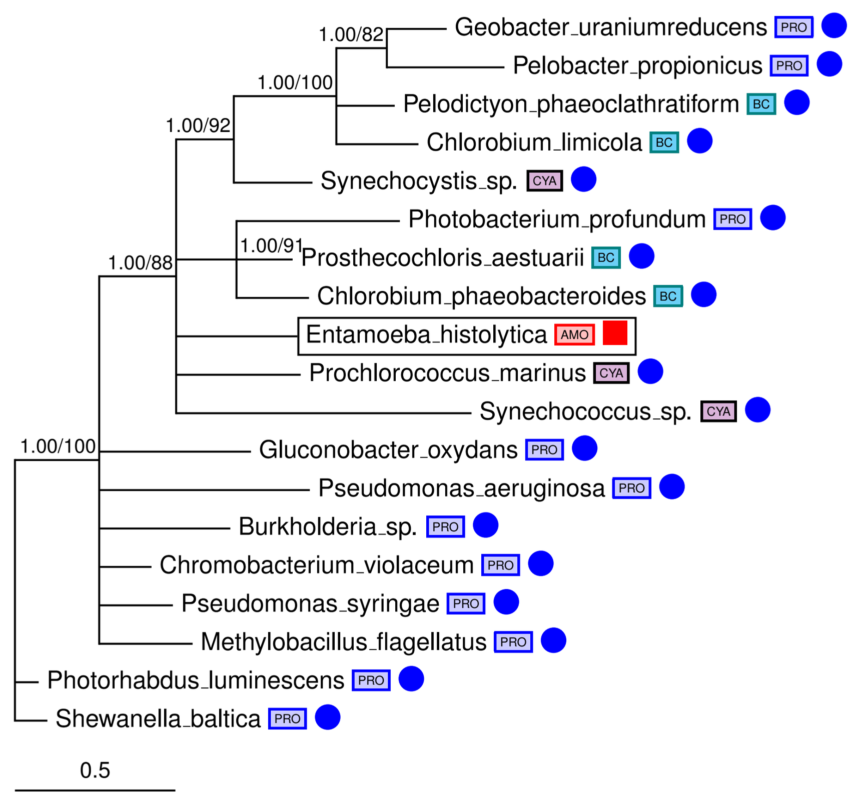

TN033

```
              Candy accession: Q50XL2_ENTHI
             RefSeq accession: XP_651697.1
            Uniprot accession: C4M0V6_ENTHI
                     Comments: LGT - EH ONLY
             Species affected: EH
        Adjacent taxa in tree: Prokaryotes
EC annotation - (Blast/Profile): na
                  PHOBIUS SP: 0
                 PHOBIUS TMD: 0
            RefSeq annotation: hypothetical protein
     Name of enzyme/protein: Predicted S-adenosyl-l-methionine
                              hydroxide adenosyltransferase
       KEGG PATHWAY - level 1: Other function
       KEGG PATHWAY - level 2: na
```

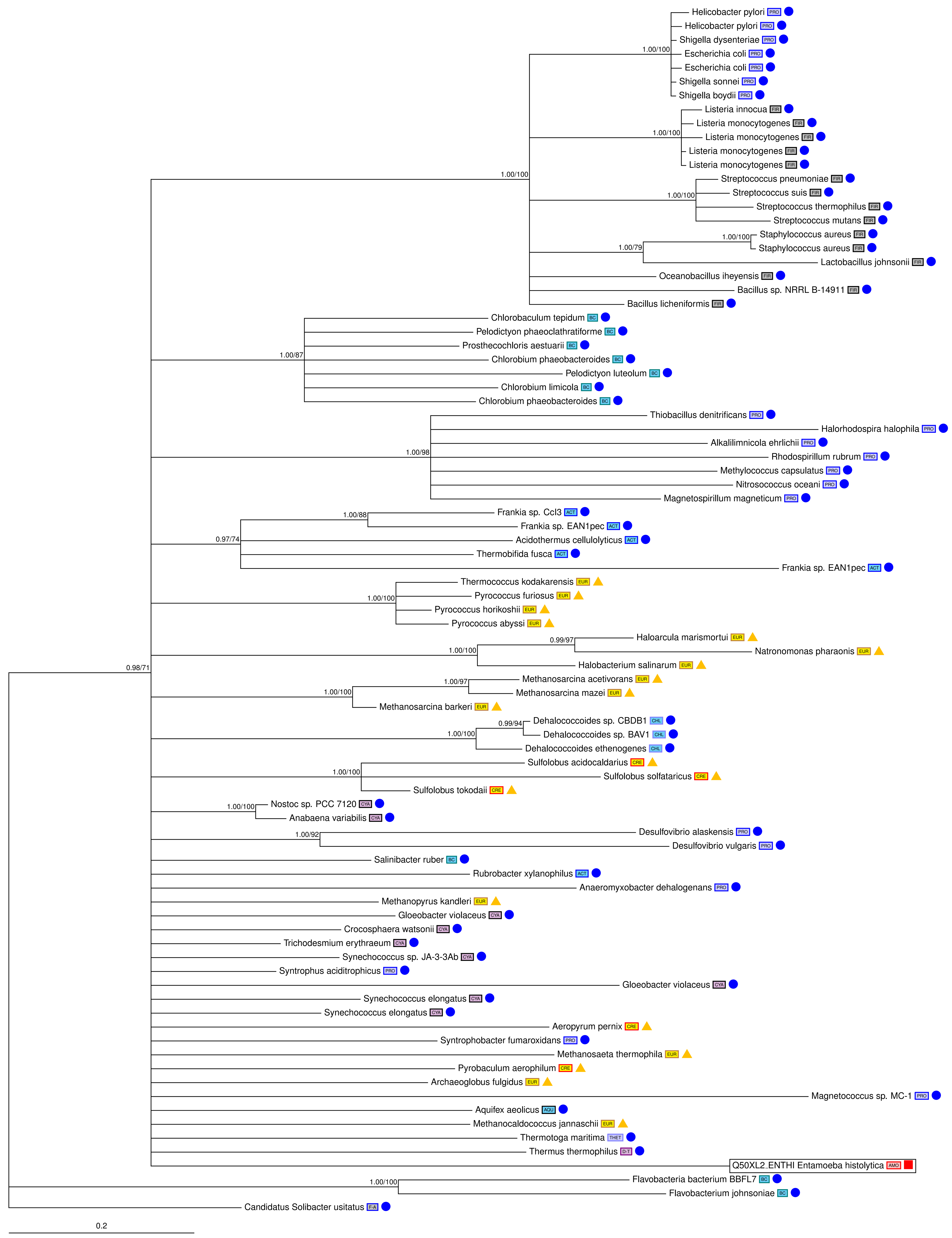

TN034

```
                Candy accession: Q50YL9_ENTHI
               RefSeq accession: XP_652065.1
              Uniprot accession: C4M2I8_ENTHI
                       Comments: LGT - EH ONLY
               Species affected: EH
           Adjacent taxa in tree: Prokaryotes
EC annotation - (Blast/Profile): na
                     PHOBIUS SP: 0
                    PHOBIUS TMD: 0
              RefSeq annotation: metal dependent hydrolase
        Name of enzyme/protein: Predicted metal dependent hydrolase
        KEGG PATHWAY - level 1: Other function
        KEGG PATHWAY - level 2: na
```

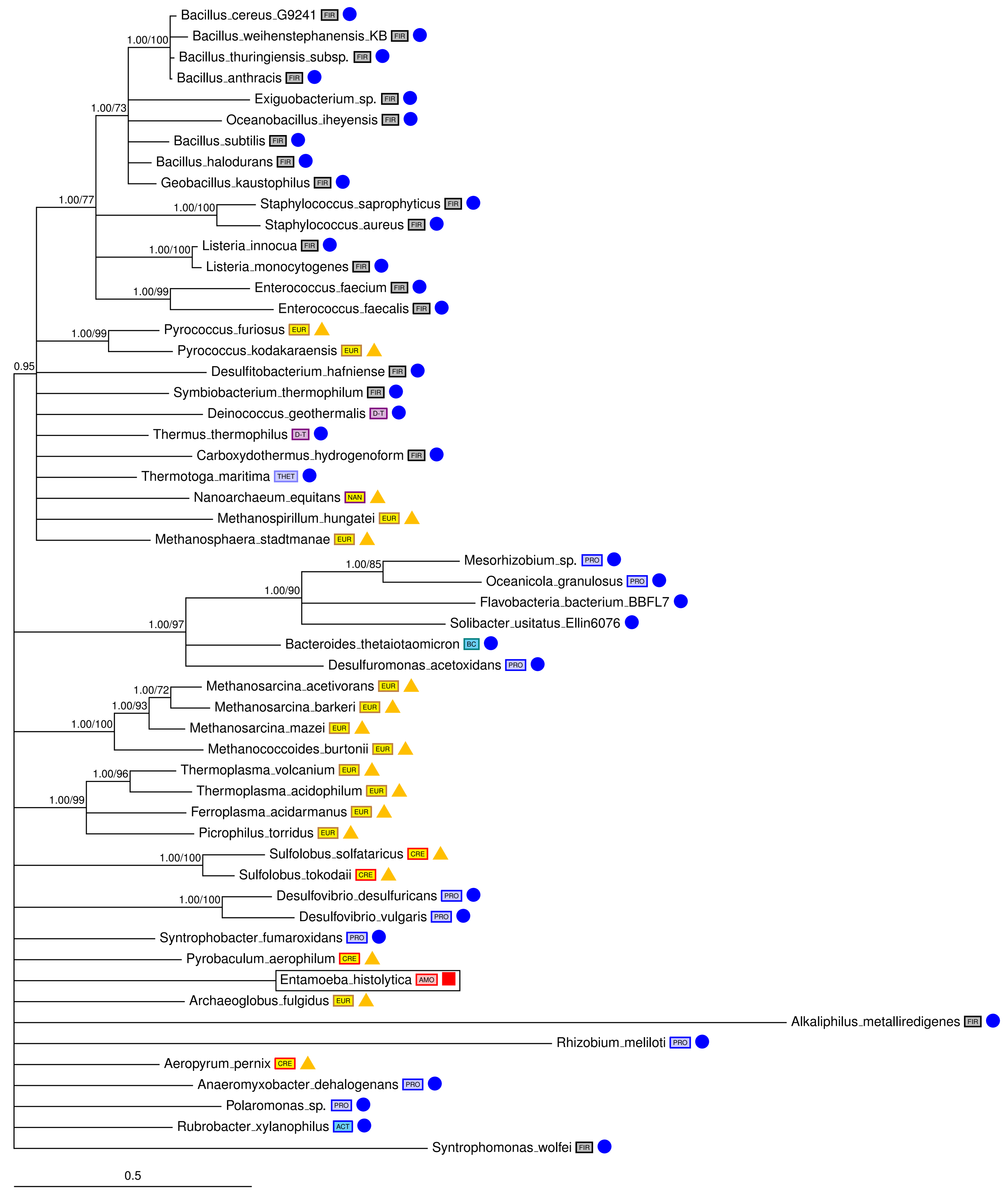

TN035

```
              Candy accession: Q50YZ1_ENTHI
             RefSeq accession: XP_652209.1
            Uniprot accession: C4M3K4_ENTHI
                     Comments: LGT - EH ONLY
             Species affected: EH
        Adjacent taxa in tree: Bacteria
EC annotation - (Blast/Profile): na
                   PHOBIUS SP: 0
                  PHOBIUS TMD: 0
            RefSeq annotation: hypothetical protein
      Name of enzyme/protein: Protein containing uncharacterized
                               domain HDIG
      KEGG PATHWAY - level 1: Function unknown
      KEGG PATHWAY - level 2: na
```

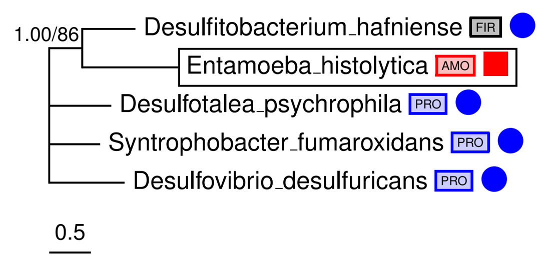

TN036

```
                Candy accession: Q51AD1_ENTHI
               RefSeq accession: XP_655177.1
              Uniprot accession: C4LWR0_ENTHI
                       Comments: LGT - EH ONLY
               Species affected: EH
          Adjacent taxa in tree: Prokaryotes
EC annotation - (Blast/Profile): na
                     PHOBIUS SP: 0
                    PHOBIUS TMD: 0
              RefSeq annotation: hypothetical protein
        Name of enzyme/protein: Predicted Mug G:T/U mismatch-specific
                                DNA glycosylase
         KEGG PATHWAY - level 1: Other function - Genetic Information
                                Processing
         KEGG PATHWAY - level 2: na
```

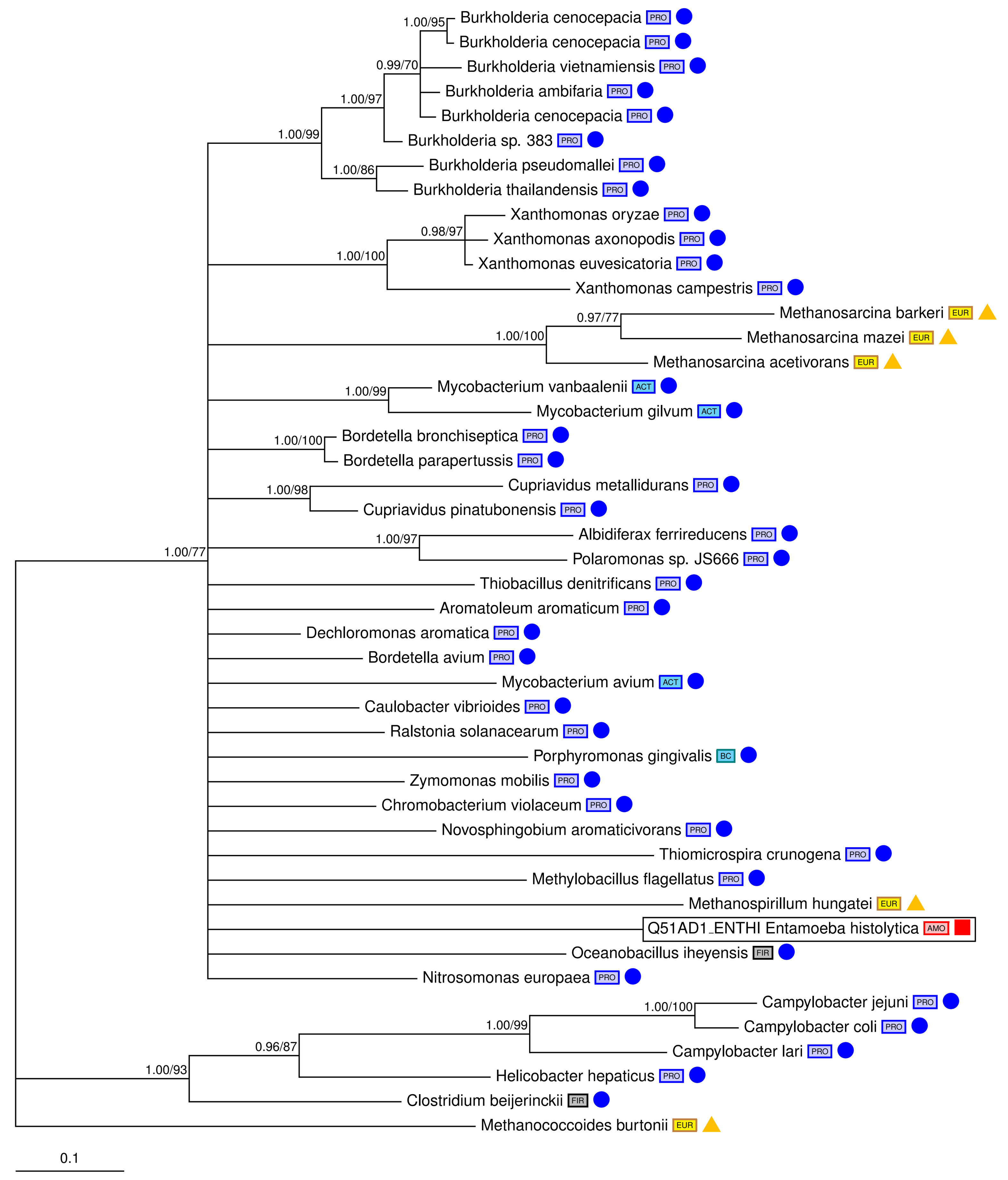

TN037

```
                Candy accession: Q51AD5_ENTHI
               RefSeq accession: XP_655200.1
              Uniprot accession: C4LWQ7_ENTHI
                       Comments: LGT - EH ONLY
               Species affected: EH
          Adjacent taxa in tree: Proteobacteria
EC annotation - (Blast/Profile): na
                     PHOBIUS SP: 0
                    PHOBIUS TMD: 0
              RefSeq annotation: hypothetical protein
         Name of enzyme/protein: Predicted flavoprotein, FMN binding,
                                 electron transport
         KEGG PATHWAY - level 1: Other function
         KEGG PATHWAY - level 2: na
```

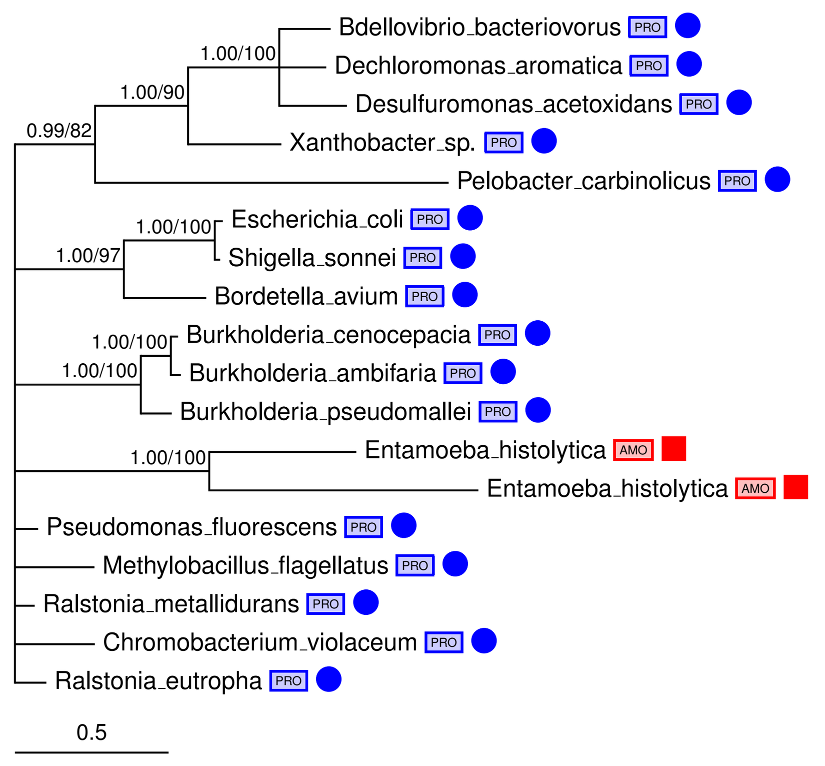

TN038

```
             Candy accession: Q51DS8_ENTHI
            RefSeq accession: XP_656375.1
           Uniprot accession: C4LSD2_ENTHI
                    Comments: LGT - EH ONLY
            Species affected: EH
      Adjacent taxa in tree: Prokaryotes
EC annotation - (Blast/Profile): EC:1.-.-.-
                  PHOBIUS SP: 0
                 PHOBIUS TMD: 0
           RefSeq annotation: nitroreductase family protein
     Name of enzyme/protein: nitroreductase family protein
      KEGG PATHWAY - level 1: Reaction
      KEGG PATHWAY - level 2: Reaction
```

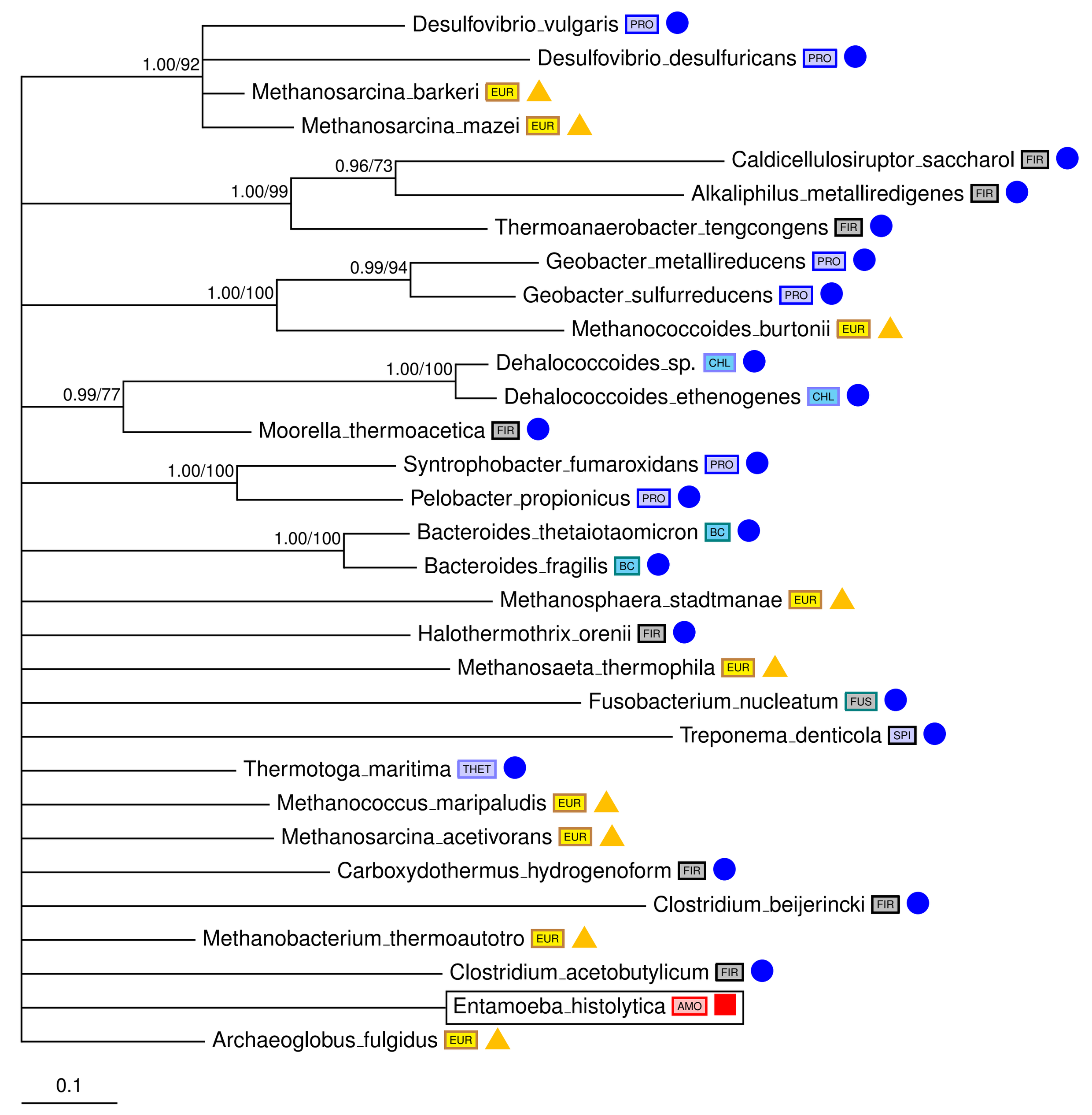

TN039

```
            Candy accession: Q54DG5_DICDI
            RefSeq accession: XP_629676.1
           Uniprot accession: Q54DG5_DICDI
                    Comments: LGT - DD ONLY
            Species affected: DD
       Adjacent taxa in tree: Protobacteria
EC annotation - (Blast/Profile): na
                 PHOBIUS SP: Y
                PHOBIUS TMD: 1
           RefSeq annotation: hypothetical protein DDB_G0292288
      Name of enzyme/protein: Uncharacterized protein with SCP/PR1
                              domains
      KEGG PATHWAY - level 1: Function unknown
      KEGG PATHWAY - level 2: na
```

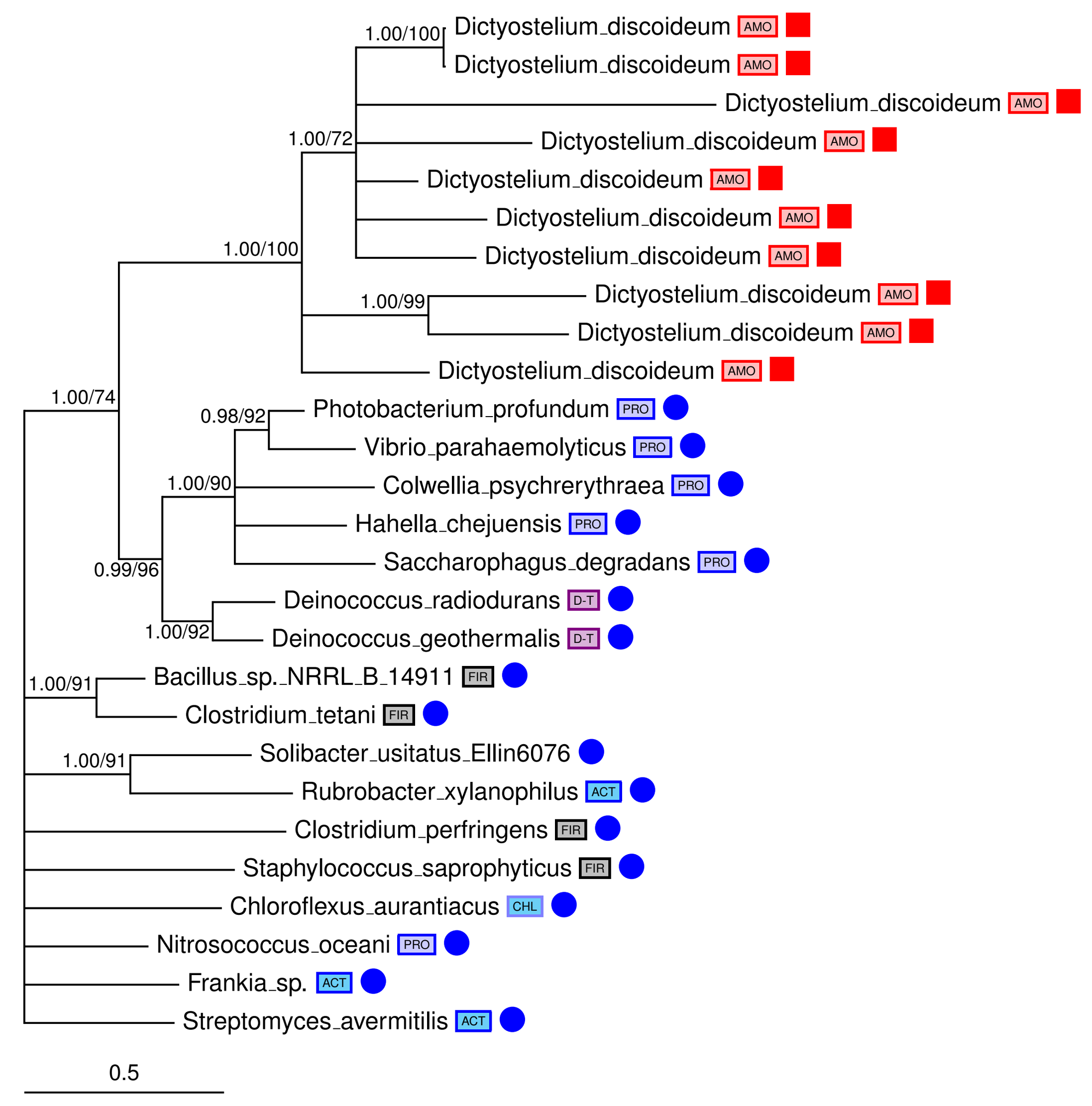

TN040

```
               Candy accession: Q54I75_DICDI
              RefSeq accession: XP_636504.1
             Uniprot accession: Q54I75_DICDI
                      Comments: LGT - DD ONLY
              Species affected: DD
         Adjacent taxa in tree: Protobacteria
EC annotation - (Blast/Profile): na
                    PHOBIUS SP: 0
                   PHOBIUS TMD: 0
             RefSeq annotation: hypothetical protein DDB_G0288943
        Name of enzyme/protein: Protein containing DUF796
        KEGG PATHWAY - level 1: Function unknown
        KEGG PATHWAY - level 2: na
```

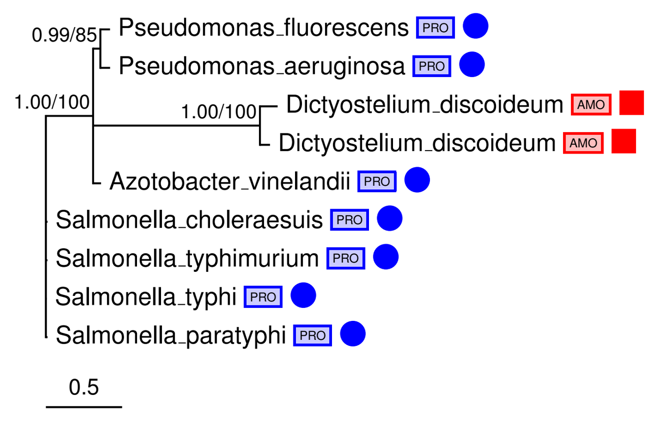

TN041

```
                Candy accession: Q54J86_DICDI
               RefSeq accession: XP_636850.1
              Uniprot accession: DTML1_DICDI
                       Comments: LGT - DD ONLY
               Species affected: DD
          Adjacent taxa in tree: Proteobacteria
EC annotation - (Blast/Profile): na
                     PHOBIUS SP: Y
                    PHOBIUS TMD: 0
              RefSeq annotation: hypothetical protein DDB_G0288219
         Name of enzyme/protein: Protein containing peptidase M66 domain
         KEGG PATHWAY - level 1: Function unknown
         KEGG PATHWAY - level 2: na
```

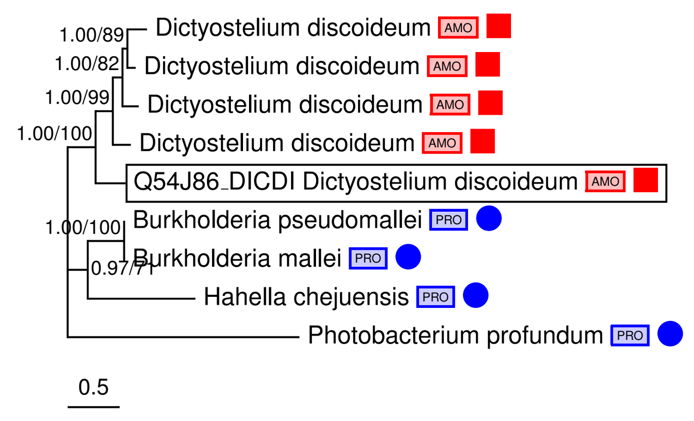

TN042

```
               Candy accession: Q54L61_DICDI
              RefSeq accession: XP_637502.1
             Uniprot accession: Q54L61_DICDI
                      Comments: LGT - DD ONLY
              Species affected: DD
        Adjacent taxa in tree: Bacteria
EC annotation - (Blast/Profile): na
                    PHOBIUS SP: 0
                   PHOBIUS TMD: 0
             RefSeq annotation: hypothetical protein DDB_G0286879
        Name of enzyme/protein: Hypothetical protein
         KEGG PATHWAY - level 1: Function unknown
         KEGG PATHWAY - level 2: na
```

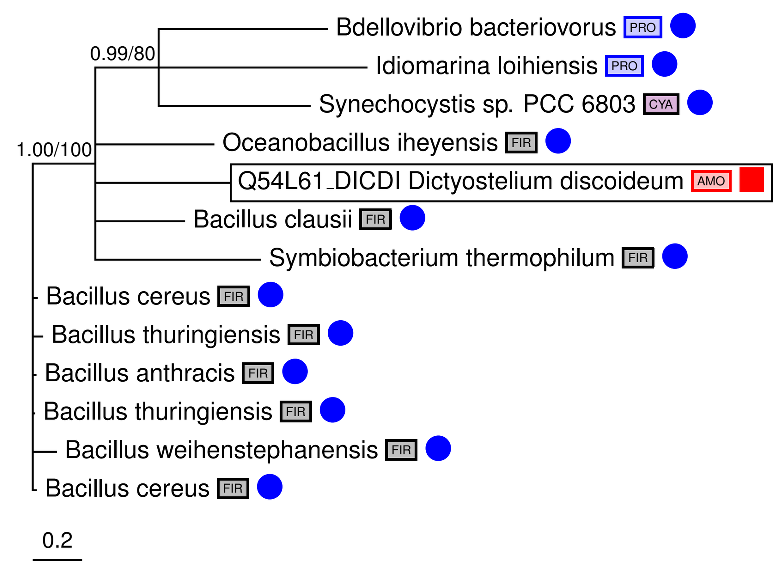

TN043

```
                Candy accession: Q54MN5_DICDI
               RefSeq accession: XP_638042.1
              Uniprot accession: Q54MN5_DICDI
                       Comments: LGT - DD ONLY
               Species affected: DD
          Adjacent taxa in tree: Bacteria
EC annotation - (Blast/Profile): na
                     PHOBIUS SP: 0
                    PHOBIUS TMD: 3
              RefSeq annotation: hypothetical protein DDB_G0285825
         Name of enzyme/protein: Protein containing DUF1294
         KEGG PATHWAY - level 1: Function unknown
         KEGG PATHWAY - level 2: na
```

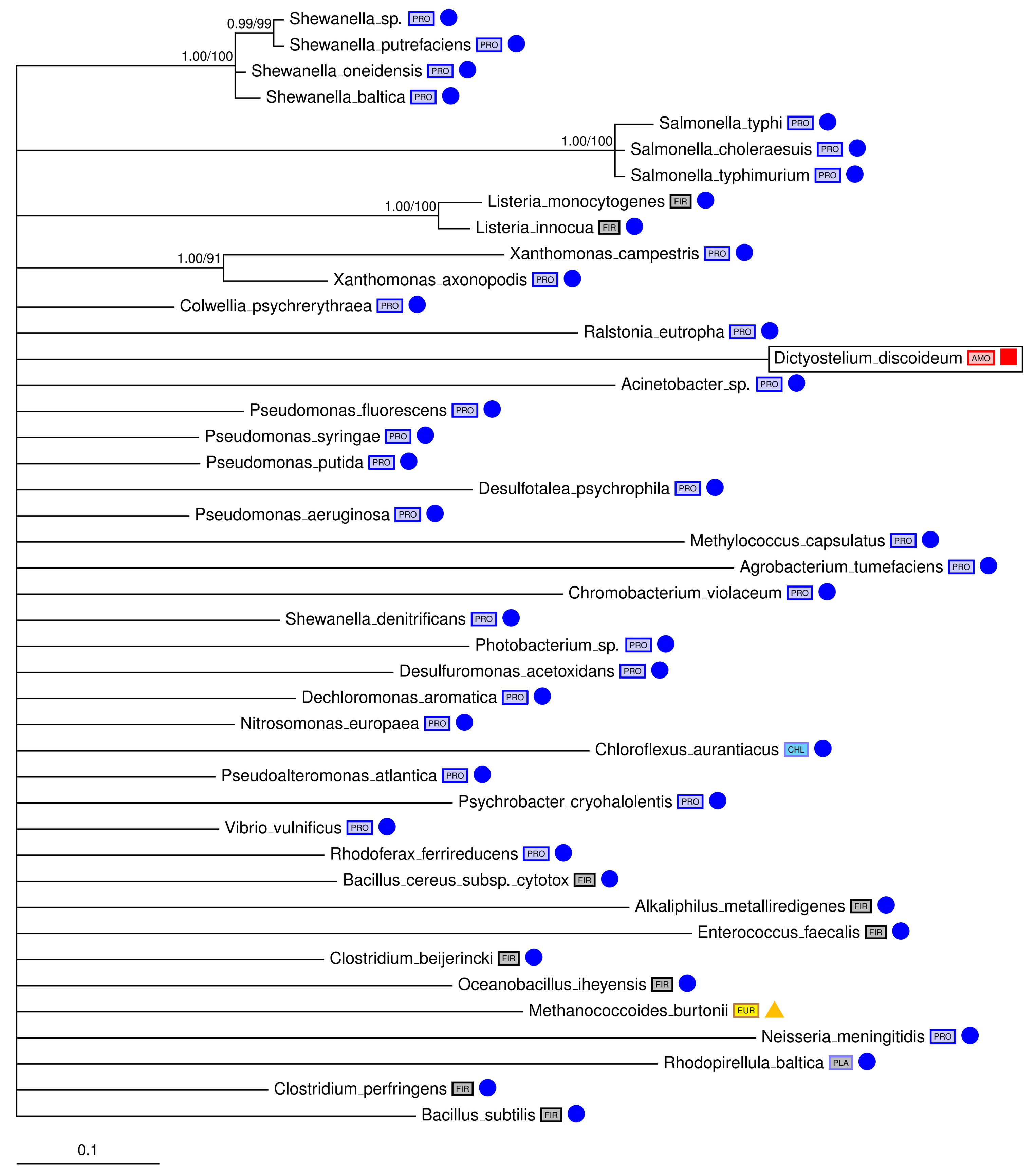

TN044

Candy accession: Q54MZ6_DICDI
RefSeq accession: XP_638147.1
Uniprot accession: Q54MZ6_DICDI
Comments: LGT - DD ONLY
Species affected: DD
Adjacent taxa in tree: Proteobacteria - Bdellovibrio
EC annotation - (Blast/Profile): na
PHOBIUS SP: 0
PHOBIUS TMD: 0
RefSeq annotation: dihydroxybiphenyl dioxygenase domain-containing protein
Name of enzyme/protein: dihydroxybiphenyl dioxygenase domain-containing protein
KEGG PATHWAY - level 1: Other function
KEGG PATHWAY - level 2: na

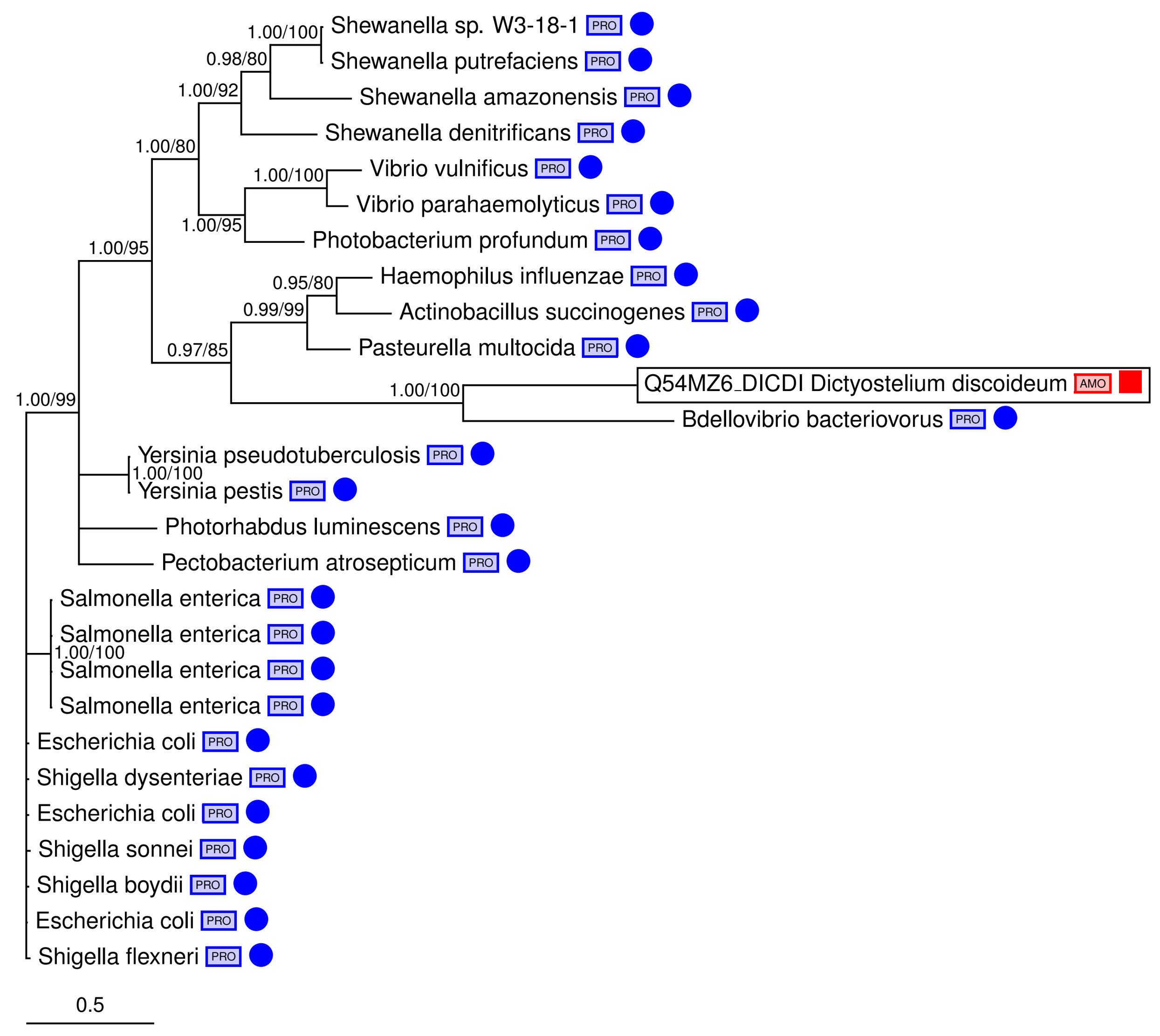

TN045

```
               Candy accession: Q54PR8_DICDI
              RefSeq accession: XP_638586.1
             Uniprot accession: Q54PR8_DICDI
                      Comments: LGT - DD ONLY
              Species affected: DD
        Adjacent taxa in tree: Prokaryotes
EC annotation - (Blast/Profile): EC:2.4.2.-
                    PHOBIUS SP: 0
                   PHOBIUS TMD: 0
            RefSeq annotation: hypothetical protein DDB_G0284365
      Name of enzyme/protein: phosphoribosyltransferase
       KEGG PATHWAY - level 1: Reaction
       KEGG PATHWAY - level 2: Reaction
```

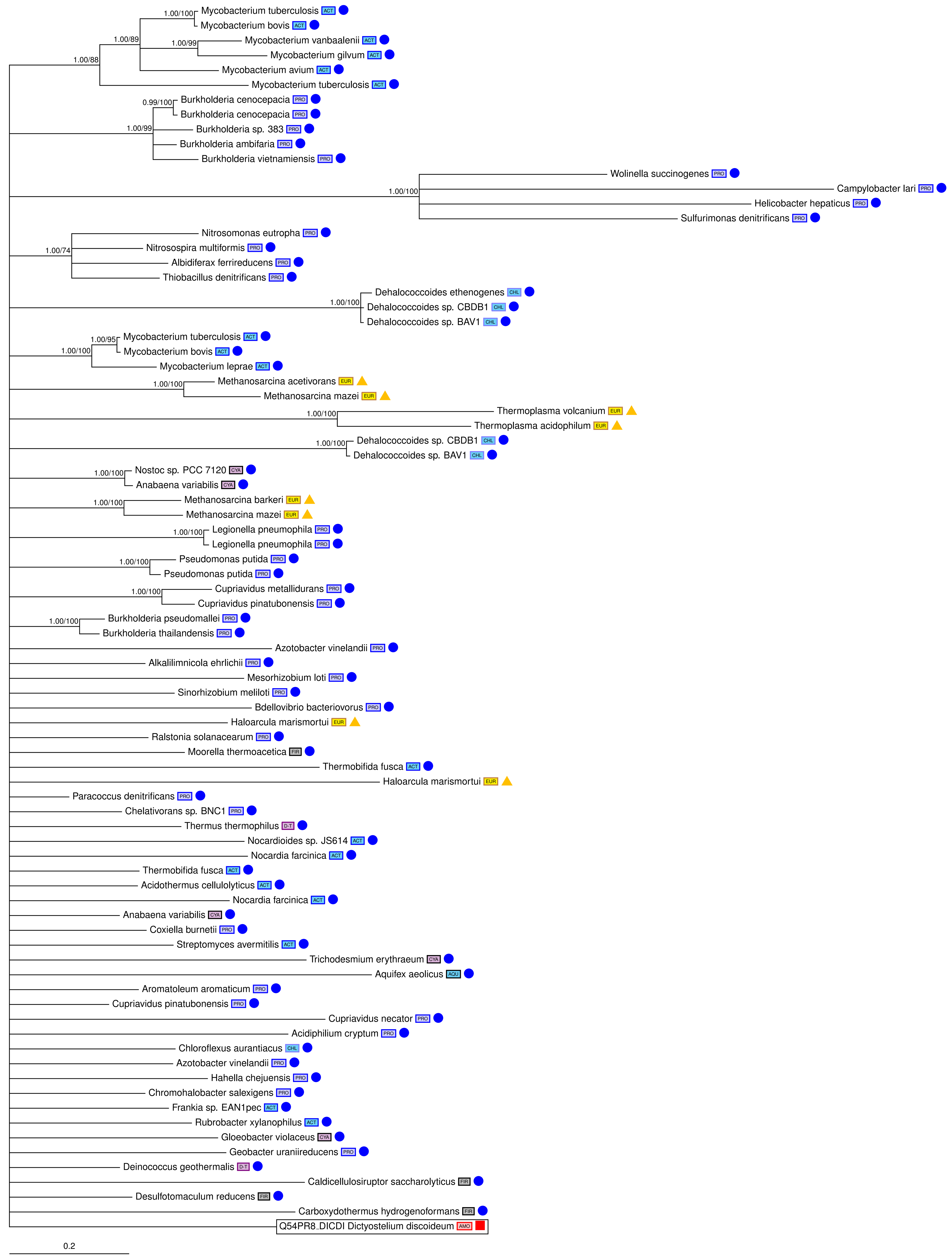

TN046

```
                  Candy accession: Q54QA3_DICDI
                 RefSeq accession: XP_638841.1
                Uniprot accession: Q54QA3_DICDI
                         Comments: LGT - DD ONLY
                 Species affected: DD
           Adjacent taxa in tree: Actinobacteria - Streptomyces
EC annotation - (Blast/Profile): na
                       PHOBIUS SP: 0
                      PHOBIUS TMD: 0
                RefSeq annotation: hypothetical protein DDB_G0283975
          Name of enzyme/protein: Protein containing methyltransferase
                                   domain
          KEGG PATHWAY - level 1: Other function
          KEGG PATHWAY - level 2: na
```

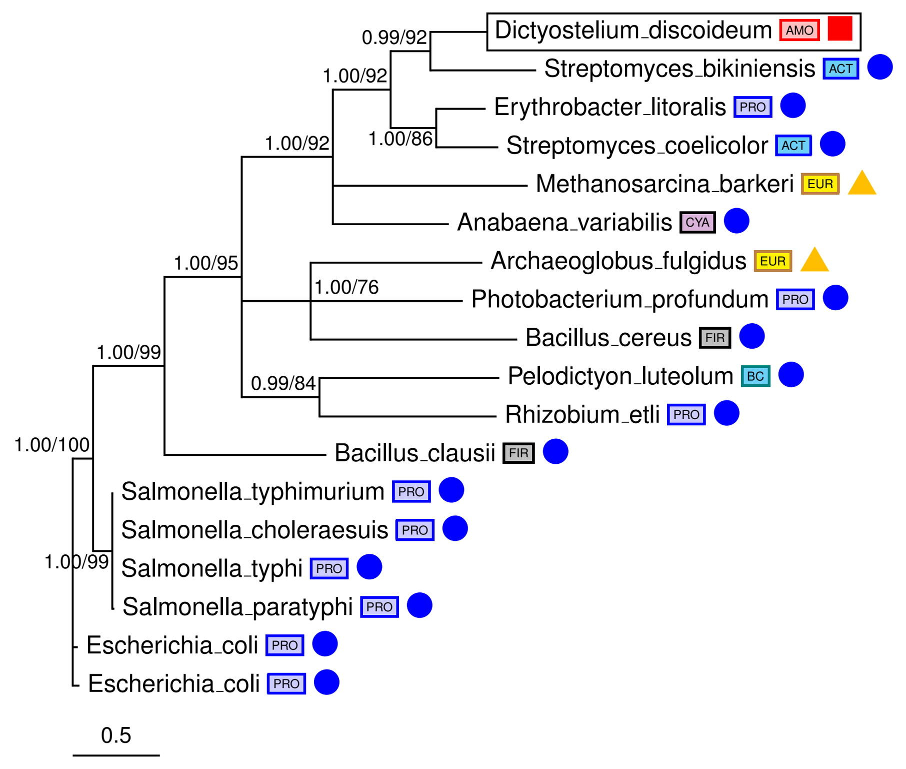

TN047

```
              Candy accession: Q54RW2_DICDI
             RefSeq accession: XP_639375.1
            Uniprot accession: Q54RW2_DICDI
                     Comments: LGT - DD ONLY
             Species affected: DD
        Adjacent taxa in tree: Proteobacteria
EC annotation - (Blast/Profile): na
                   PHOBIUS SP: 0
                  PHOBIUS TMD: 0
            RefSeq annotation: hypothetical protein DDB_G0282879
      Name of enzyme/protein: Predicted protein of unknown function
                               HutD
       KEGG PATHWAY - level 1: Function unknown
       KEGG PATHWAY - level 2: na
```

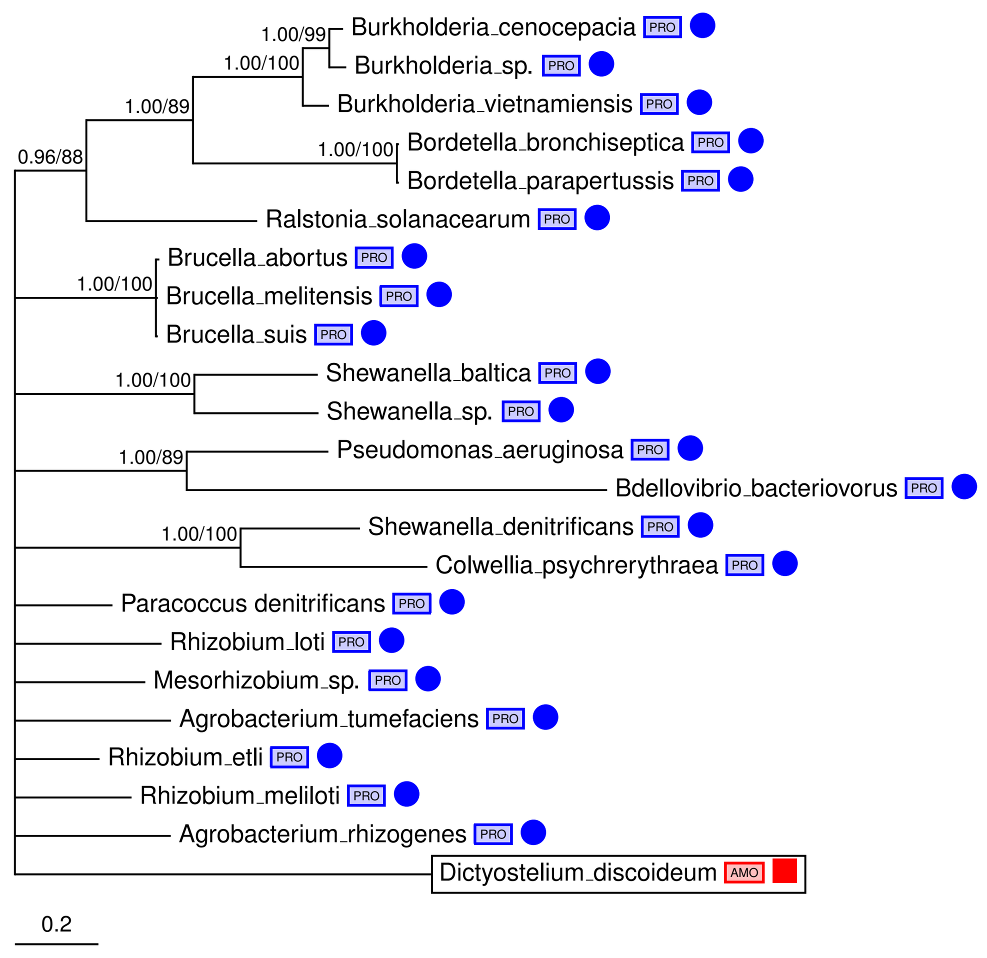

TN048

Candy accession: Q54SD6_DICDI
RefSeq accession: XP_640103.1
Uniprot accession: Q54SD6_DICDI
Comments: LGT - DD ONLY
Species affected: DD
Adjacent taxa in tree: Bacteria
EC annotation - (Blast/Profile): EC:2.7.7.12
PHOBIUS SP: 0
PHOBIUS TMD: 0
RefSeq annotation: hypothetical protein DDB_G0282525
Name of enzyme/protein: UDPglucose--hexose-1-phosphate uridylyltransferase
KEGG PATHWAY - level 1: Carbohydrate Metabolism
KEGG PATHWAY - level 2: Galactose metabolism, Amino sugar and nucleotide sugar metabolism

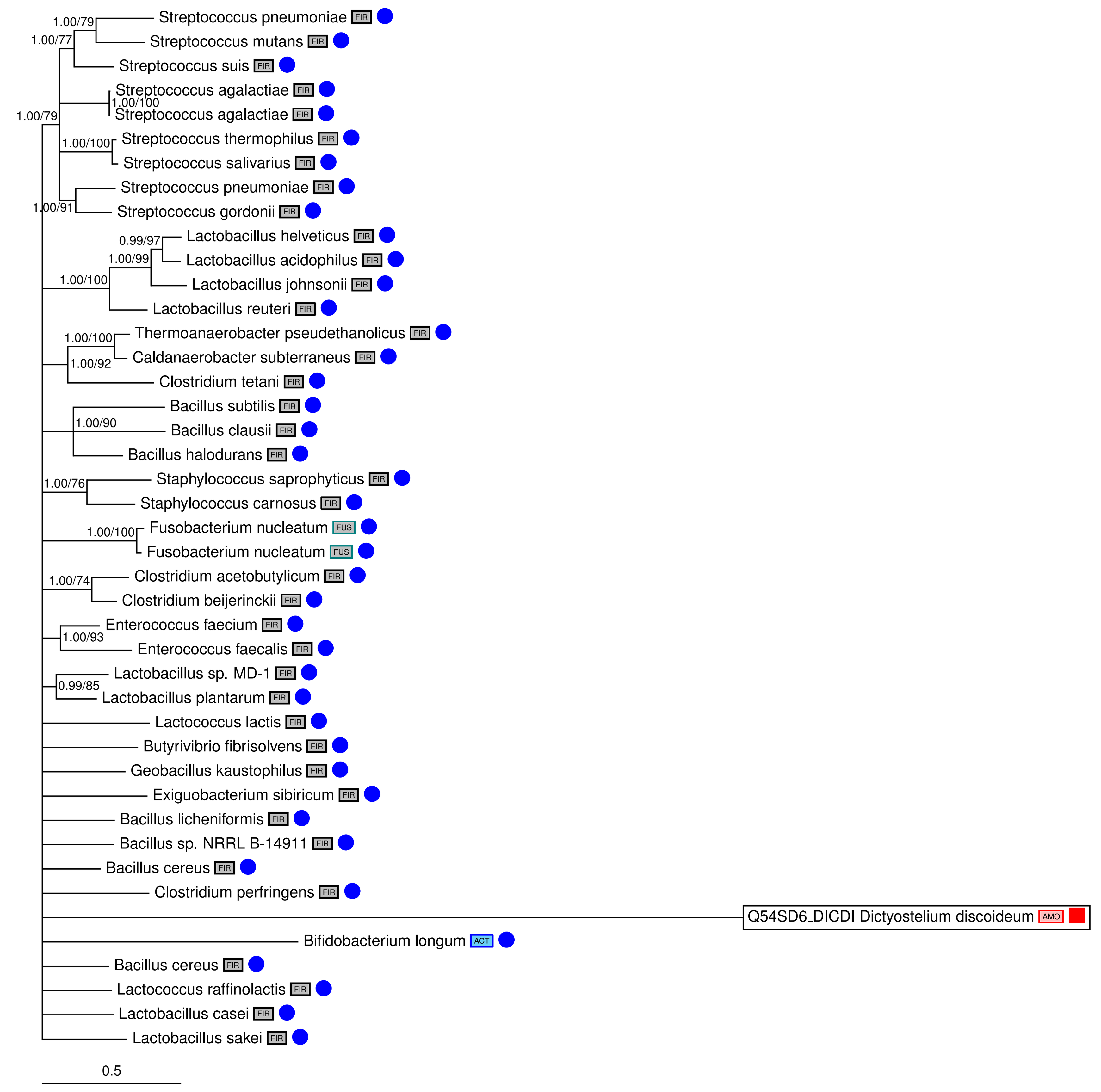

TN049

```
                Candy accession: Q54YR0_DICDI
               RefSeq accession: XP_002649169.1
              Uniprot accession: C7G007_DICDI
                       Comments: LGT - DD ONLY
               Species affected: DD
           Adjacent taxa in tree: Bacteria
EC annotation - (Blast/Profile): EC:2.3.1.128
                     PHOBIUS SP: 0
                    PHOBIUS TMD: 0
              RefSeq annotation: hypothetical protein DDB_G0295725
         Name of enzyme/protein: Ribosomal-protein-alanine
                                 N-acetyltransferase
         KEGG PATHWAY - level 1: Reaction
         KEGG PATHWAY - level 2: Reaction
```

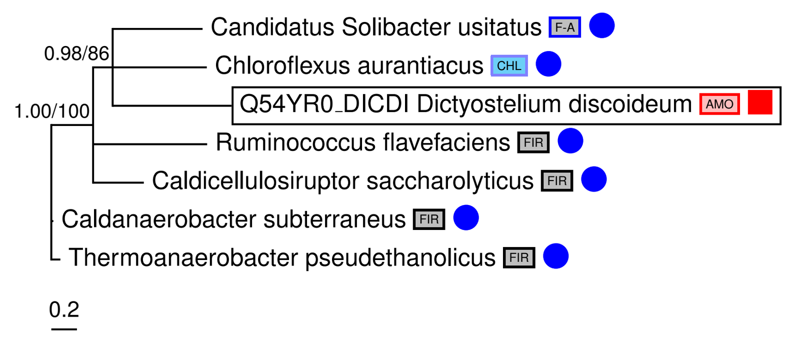

TN050

```
              Candy accession: Q55C83_DICDI
             RefSeq accession: XP_646598.1
            Uniprot accession: Q55C83_DICDI
                     Comments: LGT - DD ONLY
             Species affected: DD
       Adjacent taxa in tree: Bacteria
EC annotation - (Blast/Profile): na
                  PHOBIUS SP: 0
                 PHOBIUS TMD: 0
           RefSeq annotation: hypothetical protein DDB_G0270172
       Name of enzyme/protein: Protein containing bifunctional DNA-
                               binding transcriptional repressor/
                               nicotinamide-nucleotide
                               adenylyltransferase
        KEGG PATHWAY - level 1: Other function - Genetic Information
                               Processing
        KEGG PATHWAY - level 2: na
```

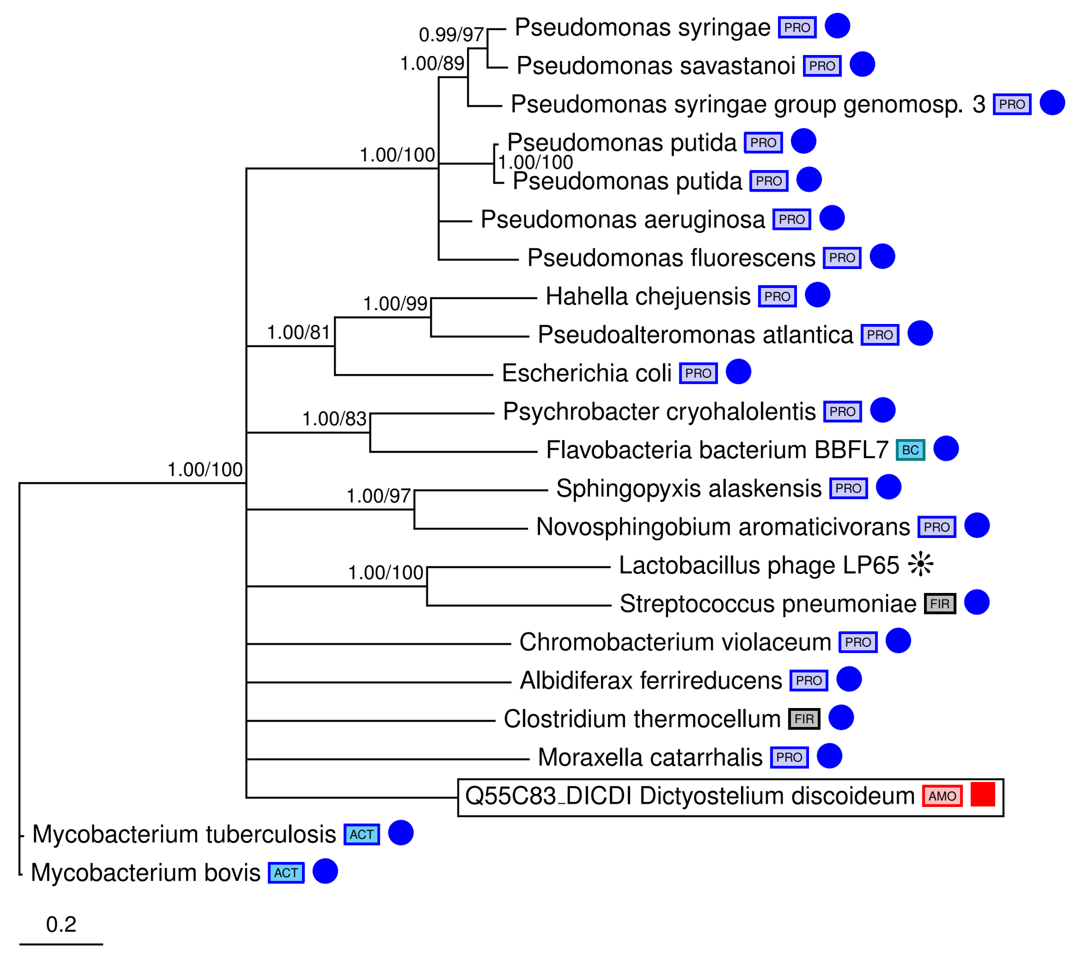

TN051

```
                Candy accession: Q55EI4_DICDI
               RefSeq accession: XP_647036.1
              Uniprot accession: Q55EI4_DICDI
                       Comments: LGT - DD ONLY
               Species affected: DD
         Adjacent taxa in tree: Bacteria
EC annotation - (Blast/Profile): EC:1.11.1.15
                     PHOBIUS SP: 0
                    PHOBIUS TMD: 0
              RefSeq annotation: osmotically inducible family protein
         Name of enzyme/protein: peroxiredoxin, thioredoxin peroxidase
         KEGG PATHWAY - level 1: Metabolism of Other Amino Acids
         KEGG PATHWAY - level 2: Glutathione metabolism
```

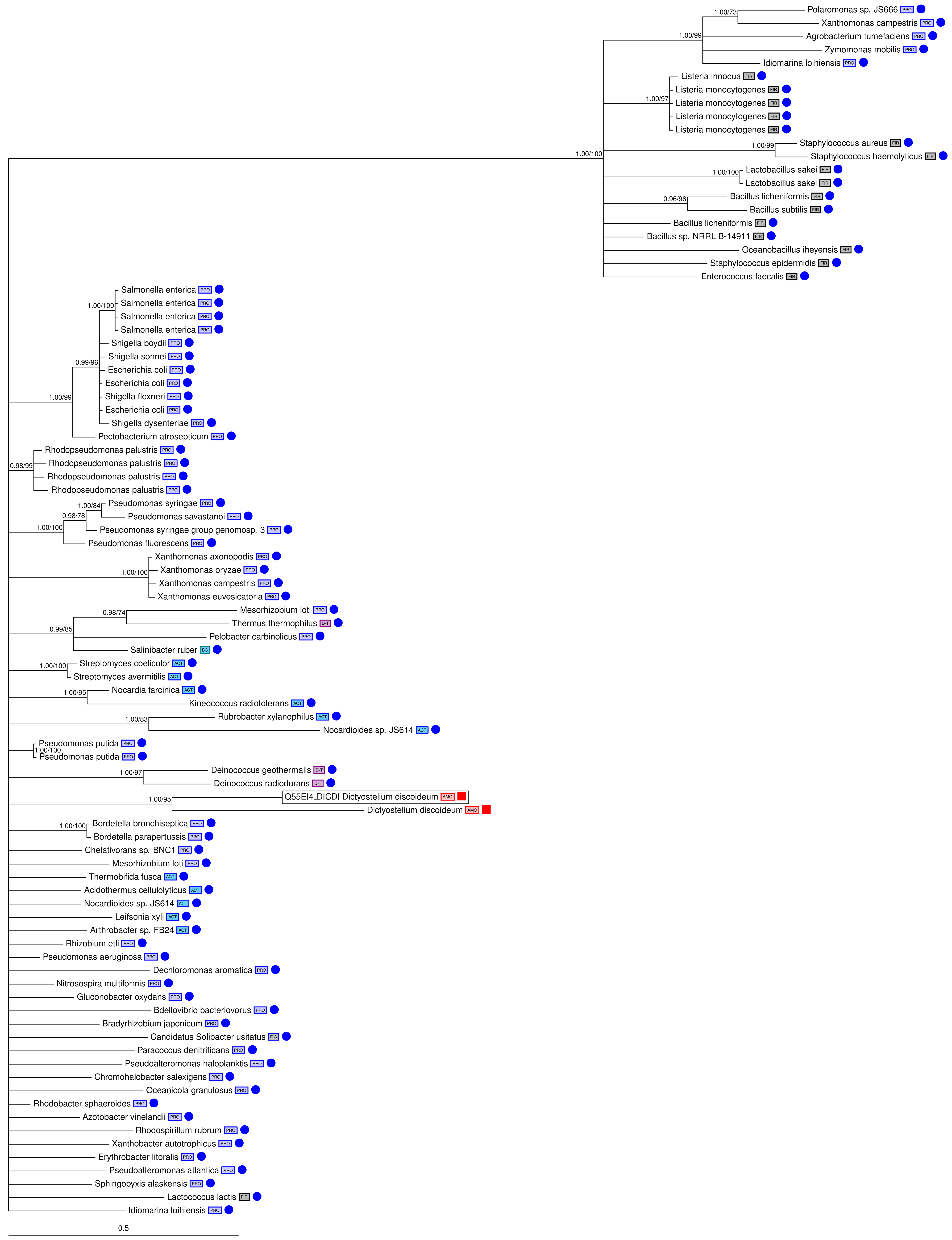

TN052

```
            Candy accession: Q55EP2_DICDI
           RefSeq accession: XP_646978.1
          Uniprot accession: Q55EP2_DICDI
                   Comments: LGT - DD ONLY
           Species affected: DD
     Adjacent taxa in tree: Bacteria
EC annotation - (Blast/Profile): na
                 PHOBIUS SP: 0
                PHOBIUS TMD: 0
          RefSeq annotation: hypothetical protein DDB_G0269088
   Name of enzyme/protein: Protein containing Phage Tail Collar
                            Domain/MdpB Microcystin-dependent
                            domains
     KEGG PATHWAY - level 1: Other function
     KEGG PATHWAY - level 2: na
```

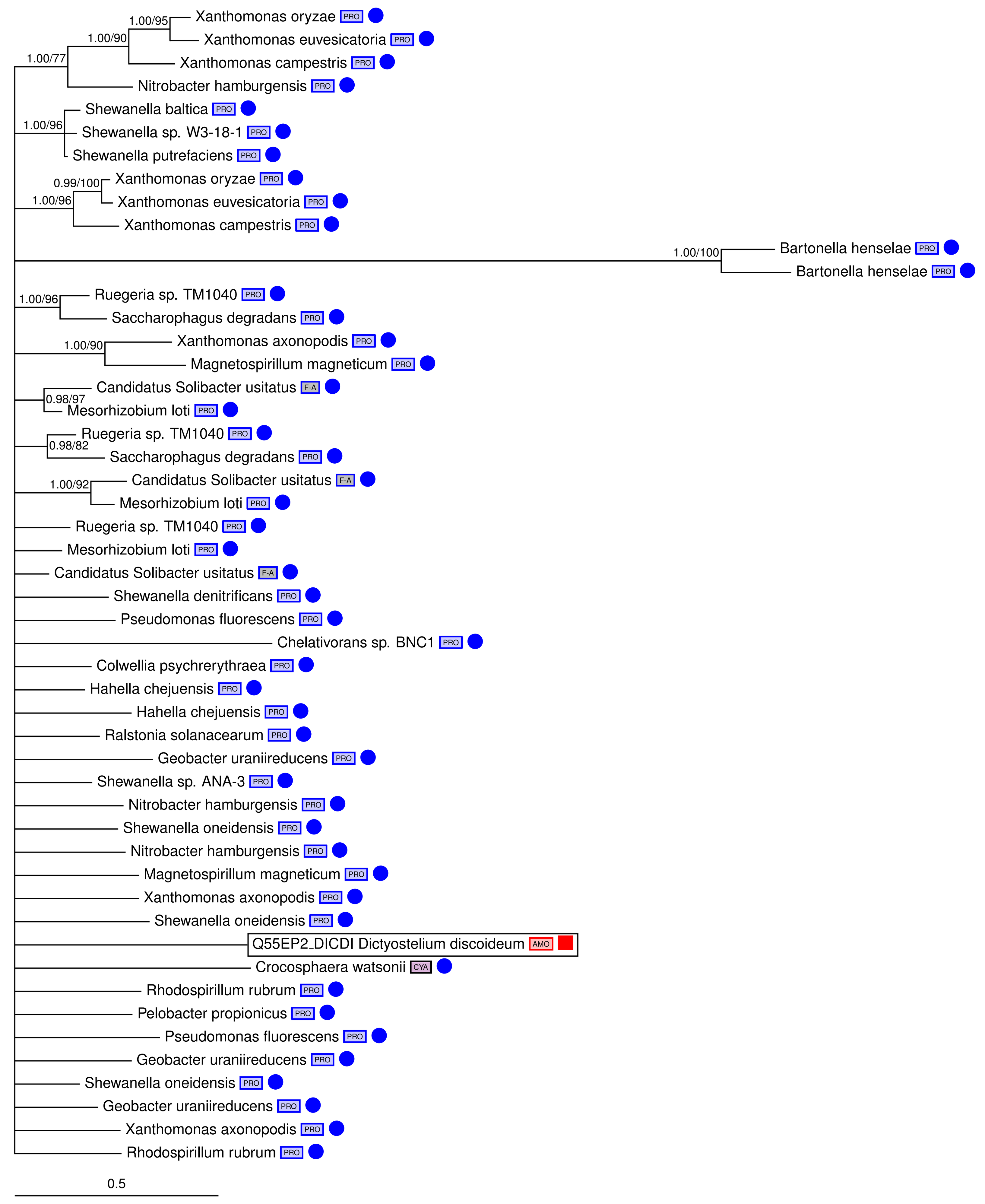

TN053

```
              Candy accession: Q55GC9_DICDI
             RefSeq accession: XP_647255.1
            Uniprot accession: Q55GC9_DICDI
                     Comments: LGT - DD ONLY
             Species affected: DD
       Adjacent taxa in tree: Bacteria
EC annotation - (Blast/Profile): na
                   PHOBIUS SP: 0
                  PHOBIUS TMD: 0
            RefSeq annotation: hypothetical protein DDB_G0267722
      Name of enzyme/protein: Putative ParB-like nuclease
       KEGG PATHWAY - level 1: Other function - Genetic Information
                               Processing
       KEGG PATHWAY - level 2: na
```

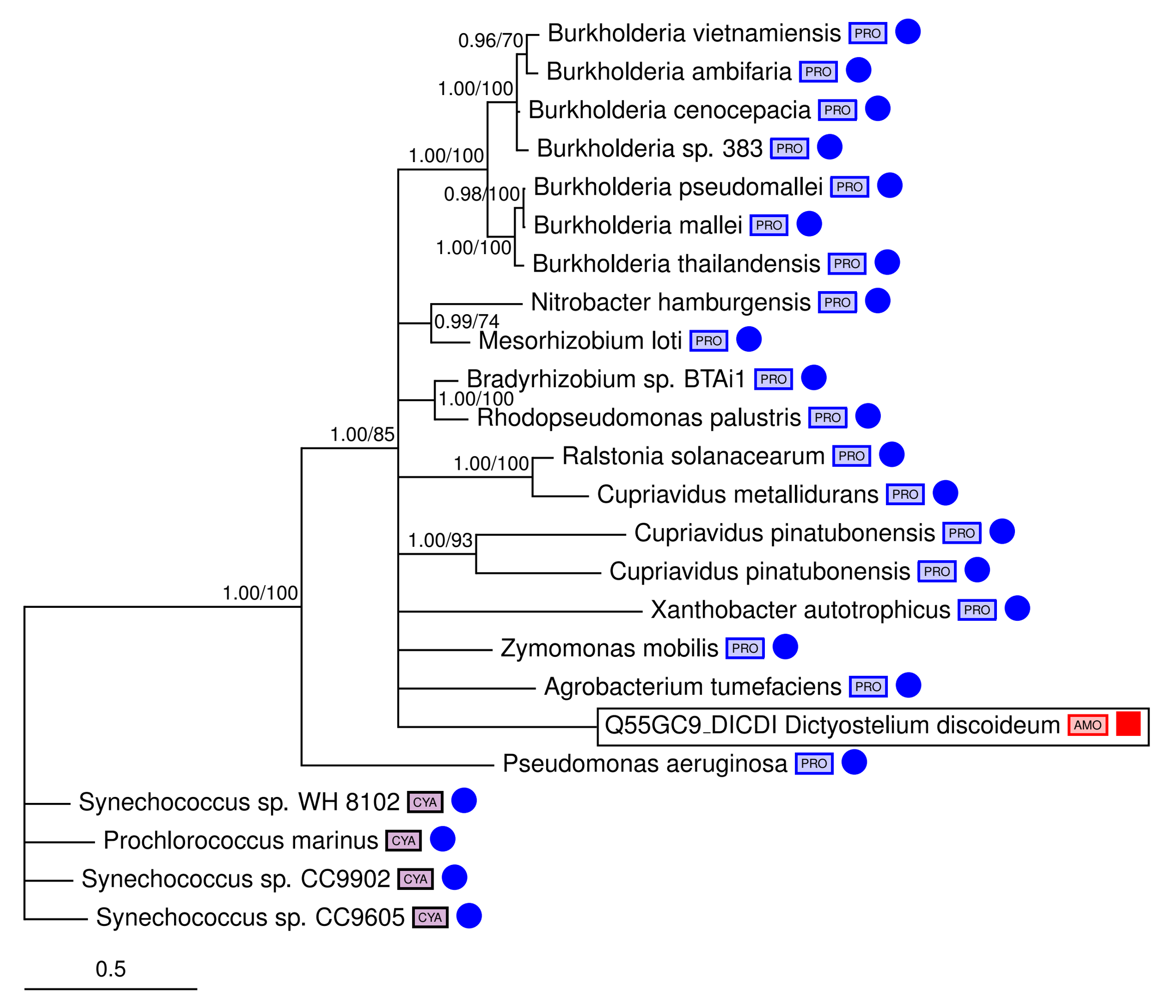

TN054

```
                Candy accession: Q57WT0_9TRYP
              RefSeq accession: XP_845744.1
             Uniprot accession: Q57WT0_9TRYP
                      Comments: LGT - KINETOPLASTIDS ONLY
              Species affected: LM,TB,TC
          Adjacent taxa in tree: Bacteria
EC annotation - (Blast/Profile): na
                    PHOBIUS SP: 0
                   PHOBIUS TMD: 0
             RefSeq annotation: hypothetical protein
        Name of enzyme/protein: Protein containing DUF525
        KEGG PATHWAY - level 1: Function unknown
        KEGG PATHWAY - level 2: na
```

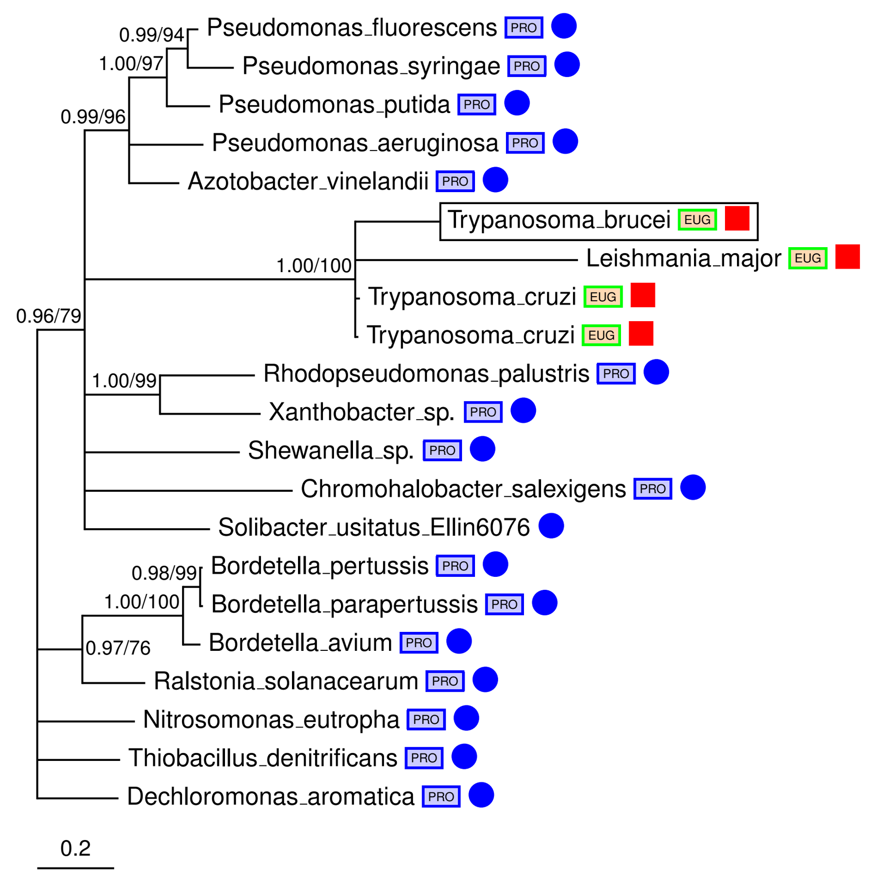

TN055

```
               Candy accession: Q57Y46_9TRYP
              RefSeq accession: XP_846303.1
             Uniprot accession: Q57Y46_9TRYP
                      Comments: LGT - KINETOPLASTIDS ONLY
              Species affected: TB,TC,TCO,TR,TCAR
         Adjacent taxa in tree: Bacteria
EC annotation - (Blast/Profile): EC:3.2.1.18
                    PHOBIUS SP: Y
                   PHOBIUS TMD: 0
             RefSeq annotation: trans-sialidase
        Name of enzyme/protein: exo-alpha-sialidase; neuraminidase;
                                sialidase
        KEGG PATHWAY - level 1: Glycan Biosynthesis and Metabolism,
                                Lipid Metabolism
        KEGG PATHWAY - level 2: Other glycan degradation, Sphingolipid
                                metabolism
```

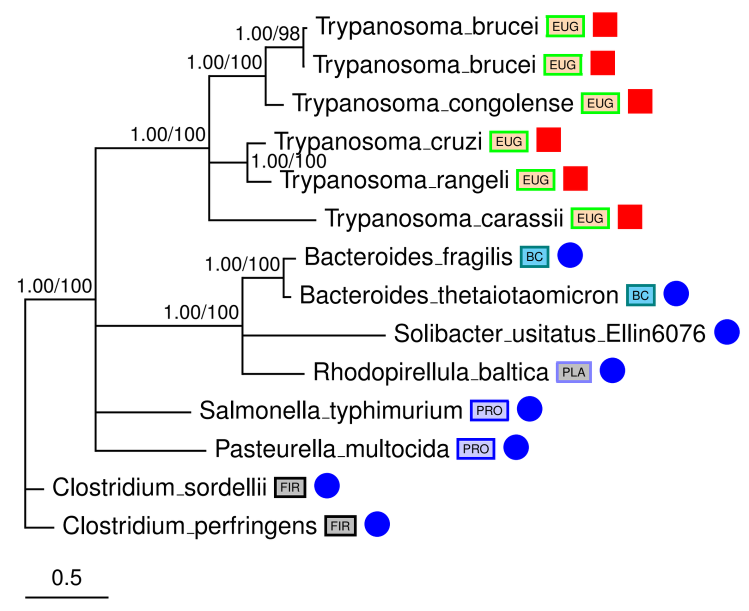

TN056

```
                Candy accession: Q57ZH3_9TRYP
               RefSeq accession: XP_846139.1
              Uniprot accession: Q57ZH3_9TRYP
                       Comments: LGT - KINETOPLASTIDS ONLY
               Species affected: LM,TB,TC
         Adjacent taxa in tree: Proteobacteria
EC annotation - (Blast/Profile): EC:3.6.1.23
                     PHOBIUS SP: Y
                    PHOBIUS TMD: 0
              RefSeq annotation: deoxyuridine triphosphatase
       Name of enzyme/protein: dUTP pyrophosphatase
       KEGG PATHWAY - level 1: Nucleotide Metabolism
       KEGG PATHWAY - level 2: Pyrimidine metabolism
```

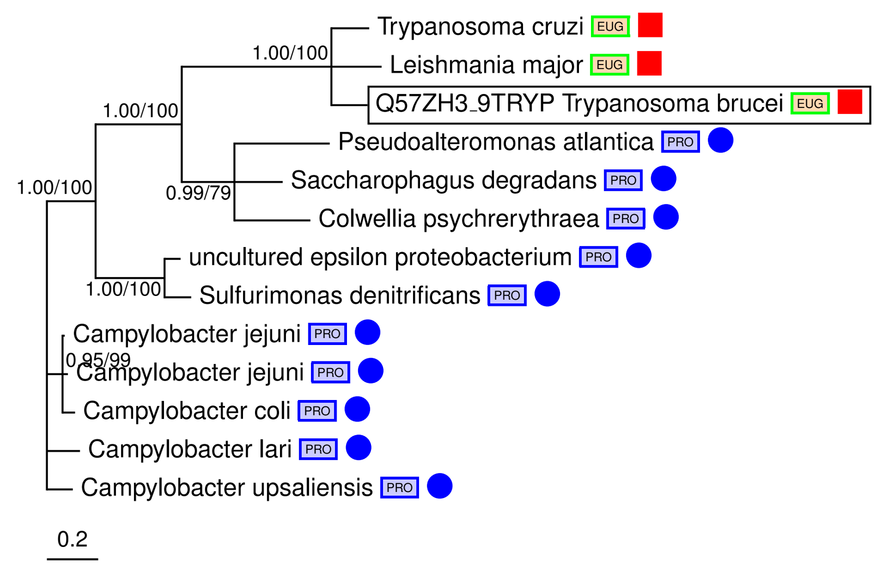

TN057

Candy accession:	Q86I79_DICDI
RefSeq accession:	XP_645397.1
Uniprot accession:	PSCA_DICDI
Comments:	LGT - DD - POTENTIAL CYANOBACTERIA DONOR
Species affected:	DD
Adjacent taxa in tree:	Cyanobacteria
EC annotation - (Blast/Profile):	EC:3.4.16.4
PHOBIUS SP:	Y
PHOBIUS TMD:	0
RefSeq annotation:	hypothetical protein DDB_G0271902
Name of enzyme/protein:	serine-type D-Ala-D-Ala carboxypeptidase
KEGG PATHWAY - level 1:	Glycan Biosynthesis and Metabolism
KEGG PATHWAY - level 2:	Peptidoglycan biosynthesis

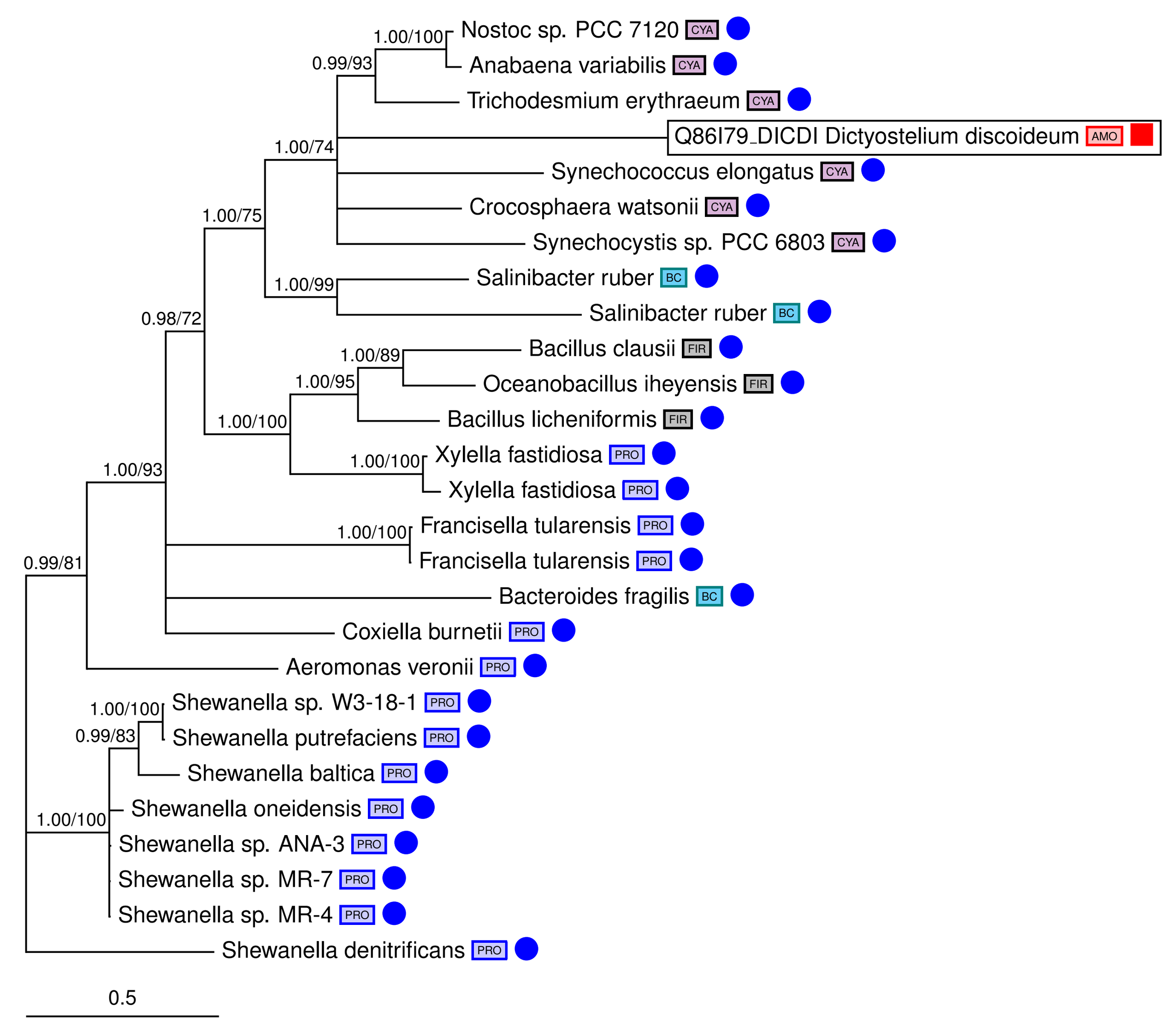

TN058

Candy accession: Q86JX4_DICDI
RefSeq accession: XP_644699.1
Uniprot accession: Y3871_DICDI
Comments: LGT - DD ONLY
Species affected: DD
Adjacent taxa in tree: Proteobacteria
EC annotation - (Blast/Profile): na
PHOBIUS SP: 0
PHOBIUS TMD: 0
RefSeq annotation: hypothetical protein DDB_G0273177
Name of enzyme/protein: Predicted rhamnose mutarotase
KEGG PATHWAY - level 1: Function unknown
KEGG PATHWAY - level 2: na

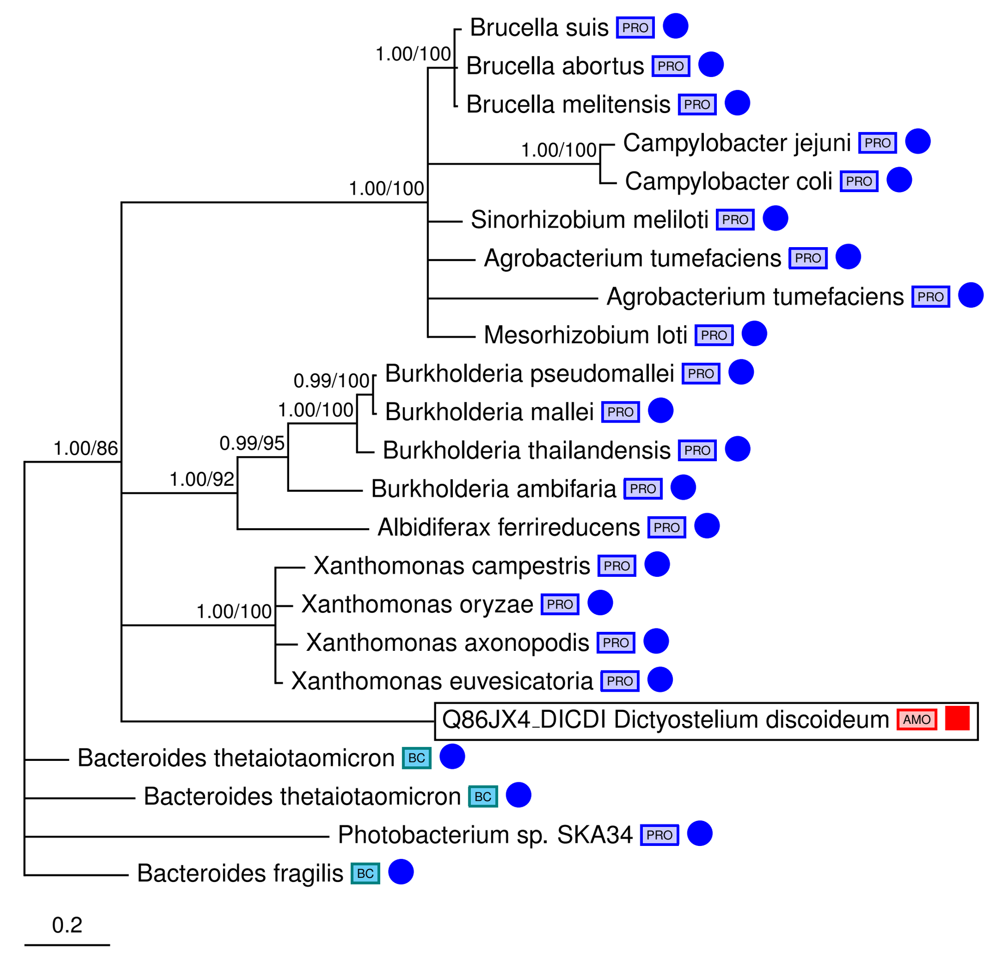

TN059

Candy accession: Q86K62_DICDI
RefSeq accession: XP_642725.1
Uniprot accession: Q86K62_DICDI
Comments: LGT - DD ONLY - MANY VIRUSES
Species affected: DD
Adjacent taxa in tree: Proteobacteria & phage
EC annotation - (Blast/Profile): na
PHOBIUS SP: Y
PHOBIUS TMD: 0
RefSeq annotation: hypothetical protein DDB_G0277201
Name of enzyme/protein: Predicted N-acetylglucosamine-binding protein; Chitin-binding protein
KEGG PATHWAY - level 1: Other function
KEGG PATHWAY - level 2: na

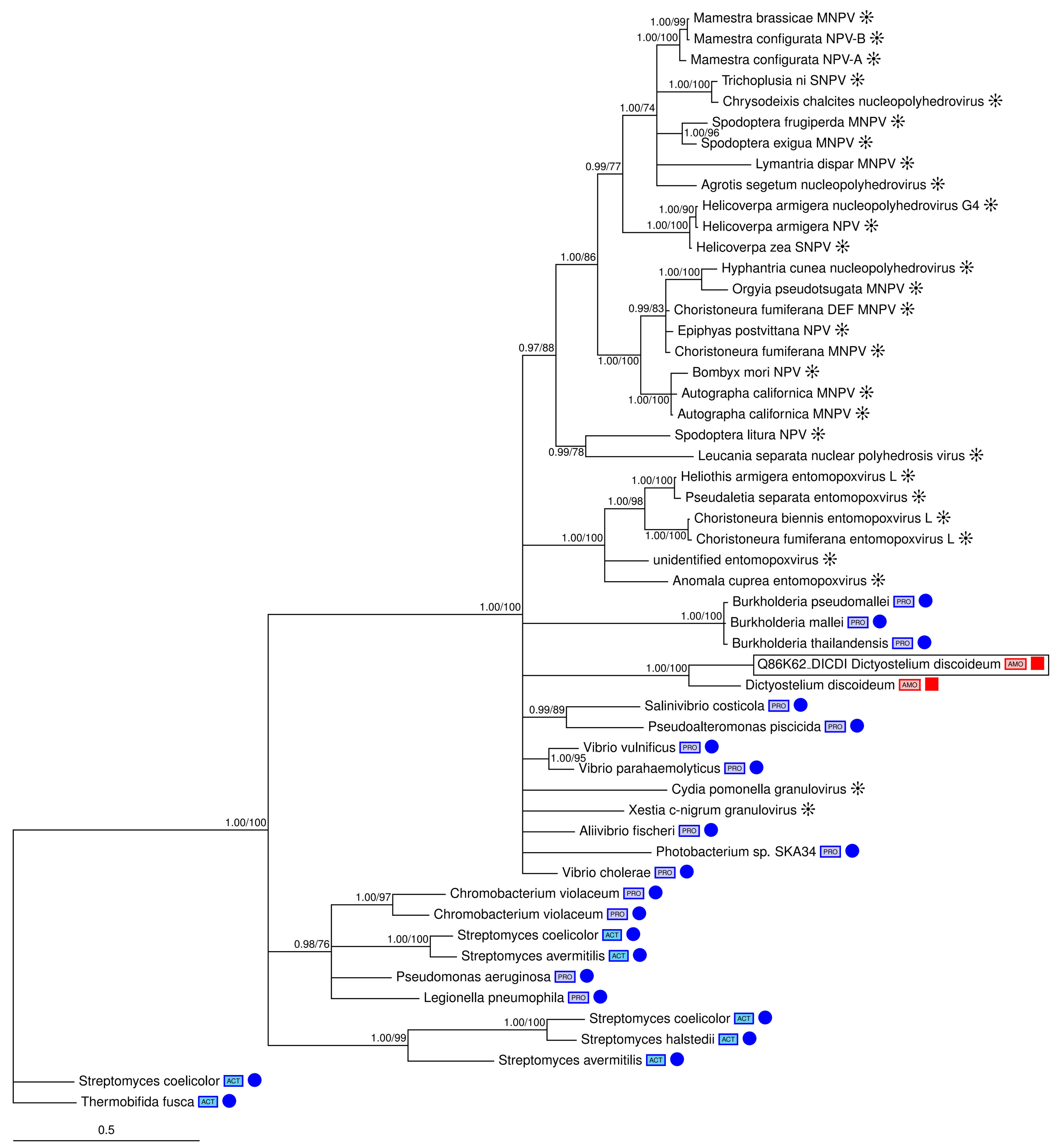

TN060

```
              Candy accession: Q512Y4_ENTHI
             RefSeq accession: XP_653291.1
            Uniprot accession: C4LWV3_ENTHI
                     Comments: LGT - EH ONLY
             Species affected: EH
         Adjacent taxa in tree: Prokaryotes
EC annotation - (Blast/Profile): EC:3.1.3.71
                   PHOBIUS SP: 0
                  PHOBIUS TMD: 0
            RefSeq annotation: 2-phosphosulfolactate phosphatase
      Name of enzyme/protein: 2-phosphosulfolactate phosphatase
       KEGG PATHWAY - level 1: Energy Metabolism
       KEGG PATHWAY - level 2: Methane metabolism
```

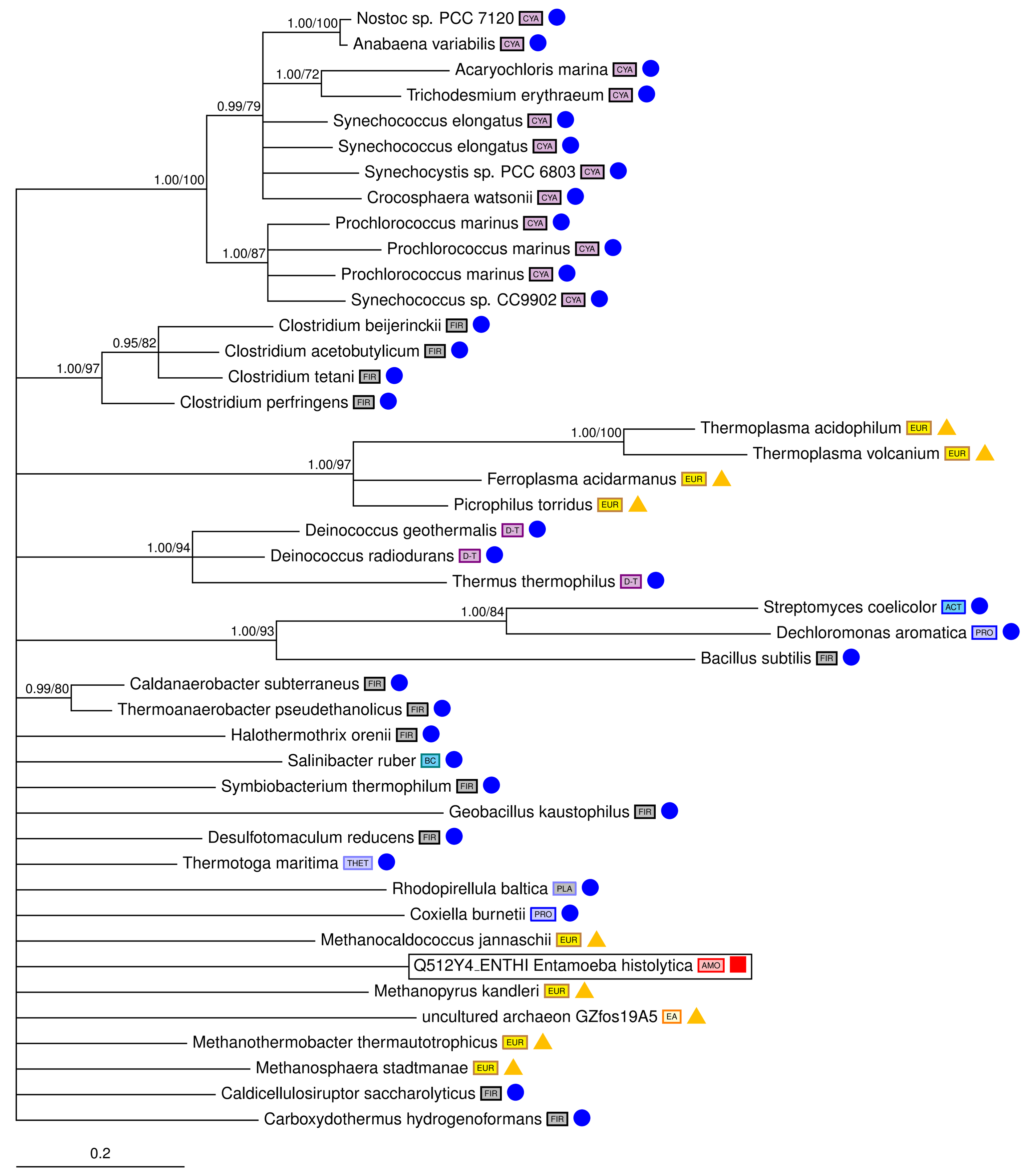

TN061

```
               Candy accession: Q513M6_ENTHI
              RefSeq accession: XP_653421.1
             Uniprot accession: C4M2N2_ENTHI
                      Comments: LGT - EH ONLY
              Species affected: EH
         Adjacent taxa in tree: Proteobacteria - Pelobacter
EC annotation - (Blast/Profile): na
                    PHOBIUS SP: 0
                   PHOBIUS TMD: 0
             RefSeq annotation: hypothetical protein
        Name of enzyme/protein: Putative redox-active protein
                                (C_GCAxxG_C_C)
        KEGG PATHWAY - level 1: Other function
        KEGG PATHWAY - level 2: na
```

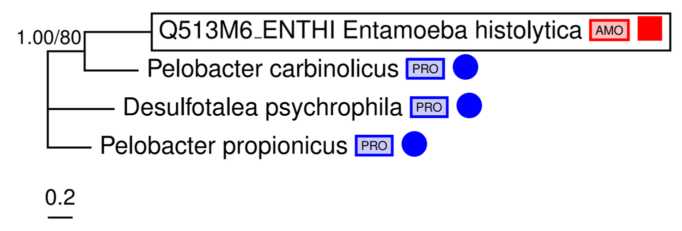

TN062

```
                Candy accession: Q516Y7_ENTHI
               RefSeq accession: XP_654288.1
              Uniprot accession: C4M6B4_ENTHI
                       Comments: LGT - EH ONLY with 4 Archaea
               Species affected: EH
          Adjacent taxa in tree: Archaea
EC annotation - (Blast/Profile): na
                     PHOBIUS SP: 0
                    PHOBIUS TMD: 0
               RefSeq annotation: hypothetical protein
         Name of enzyme/protein: Predicted phosphohydrolase
         KEGG PATHWAY - level 1: Other function
         KEGG PATHWAY - level 2: na
```

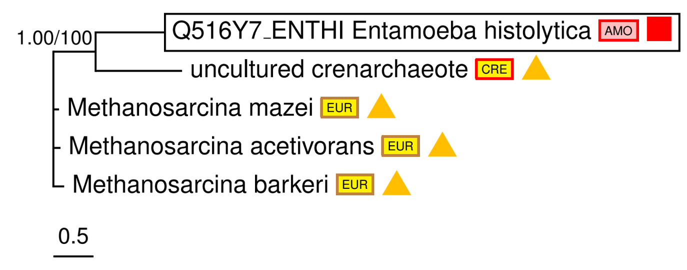

TN063

```
                Candy accession: Q555M2_DICDI
               RefSeq accession: XP_644044.1
              Uniprot accession: Q555M2_DICDI
                       Comments: LGT - DD ONLY
               Species affected: DD
          Adjacent taxa in tree: Bacteria
EC annotation - (Blast/Profile): na
                     PHOBIUS SP: 0
                    PHOBIUS TMD: 0
              RefSeq annotation: hypothetical protein DDB_G0274701
         Name of enzyme/protein: Predicted Lincosamide
                                 nucleotidyltransferase
         KEGG PATHWAY - level 1: Other function
         KEGG PATHWAY - level 2: na
```

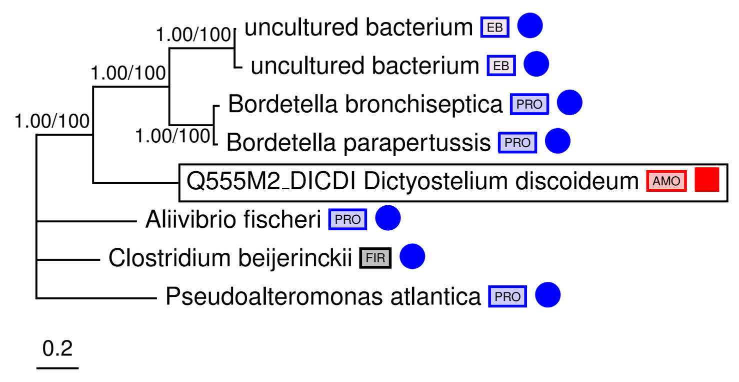

TN064

```
            Candy accession: T42_00077
           RefSeq accession: XP_002366295.1
          Uniprot accession: B6KEM2_TOXGO
                   Comments: LGT - TG ONLY
           Species affected: TG
     Adjacent taxa in tree: Proteobacteria - Magnetococcus
EC annotation - (Blast/Profile): EC:3.4.24.15
                 PHOBIUS SP: 0
                PHOBIUS TMD: 0
          RefSeq annotation: oligoendopeptidase F, putative
  Name of enzyme/protein: thimet oligopeptidase
     KEGG PATHWAY - level 1: Reaction
     KEGG PATHWAY - level 2: Reaction
```

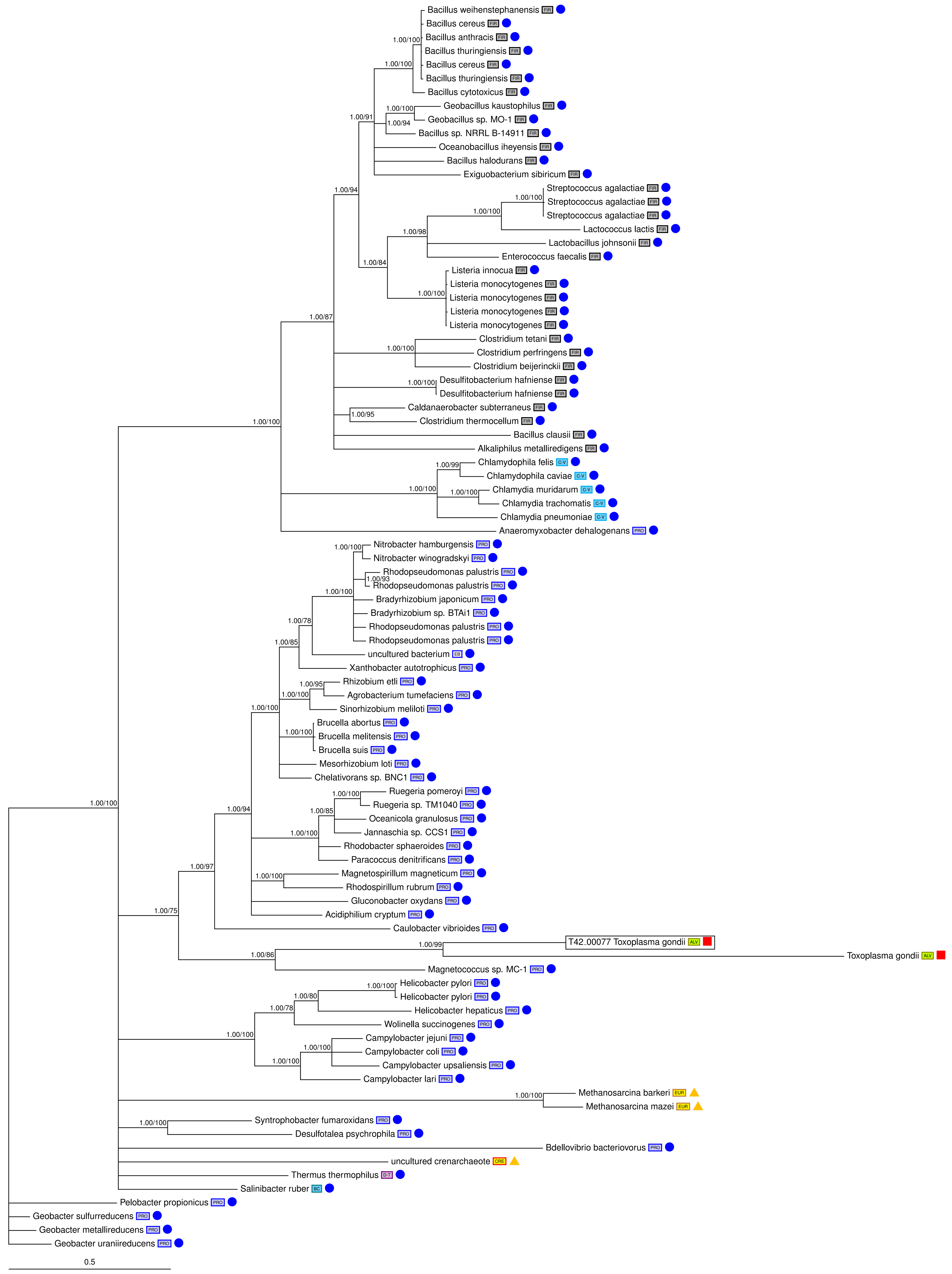

TN065

Candy accession: T49_03363
RefSeq accession: XP_002366867.1
Uniprot accession: B6KFY6_TOXGO
Comments: LGT - TG ONLY
Species affected: TG
Adjacent taxa in tree: Archaea
EC annotation - (Blast/Profile): EC:1.3.1.26
PHOBIUS SP: 0
PHOBIUS TMD: 0
RefSeq annotation: dihydrodipicolinate reductase, putative
Name of enzyme/protein: dihydrodipicolinate reductase
KEGG PATHWAY - level 1: Amino Acid Metabolism
KEGG PATHWAY - level 2: Lysine biosynthesis

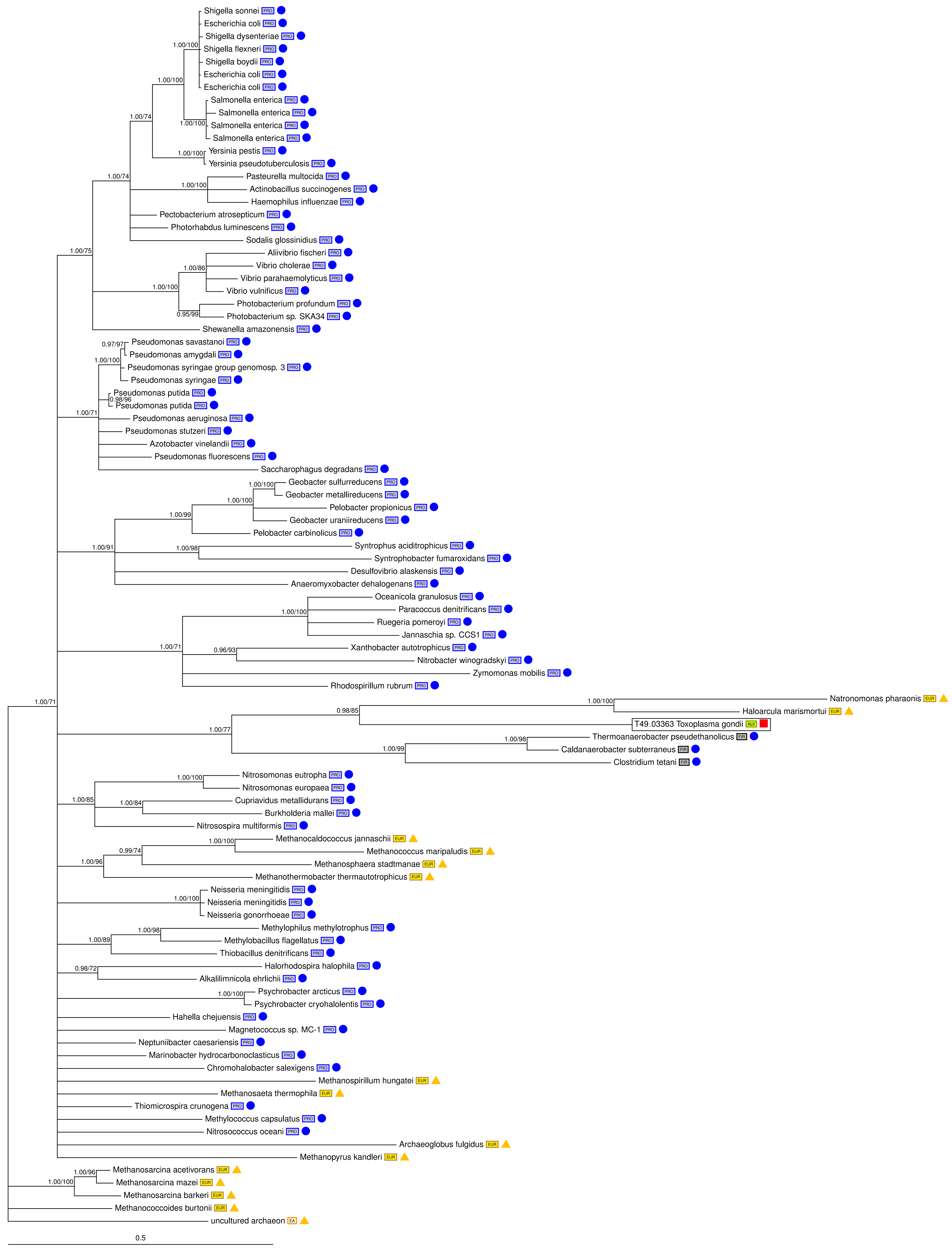

TN066

Candy accession: T52_01632
RefSeq accession: XP_002369463.1
Uniprot accession: B6KNP0_TOXGO
Comments: LGT - TG ONLY
Species affected: TG
Adjacent taxa in tree: Bacteria
EC annotation - (Blast/Profile): na
PHOBIUS SP: 0
PHOBIUS TMD: 7
RefSeq annotation: zinc transporter ZIP domain-containing protein
Name of enzyme/protein: zinc transporter ZIP domain-containing protein
KEGG PATHWAY - level 1: Other function - Membrane transport
KEGG PATHWAY - level 2: na

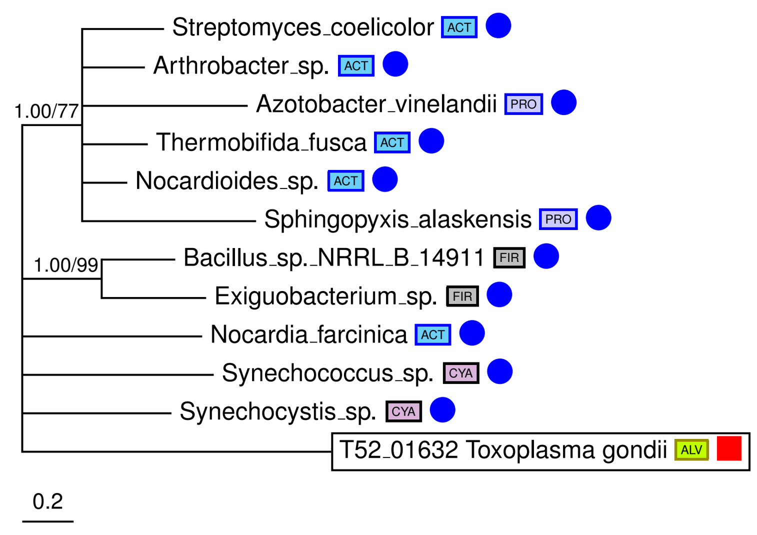

TN067

```
            Candy accession: TV80361101
           RefSeq accession: XP_001314405.1
          Uniprot accession: A2EZT6_TRIVA
                   Comments: LGT - TV ONLY
           Species affected: TV
      Adjacent taxa in tree: Bacteria
EC annotation - (Blast/Profile): na
                PHOBIUS SP: 0
               PHOBIUS TMD: 0
          RefSeq annotation: hypothetical protein
   Name of enzyme/protein: Protein containing DUF488
   KEGG PATHWAY - level 1: Function unknown
   KEGG PATHWAY - level 2: na
```

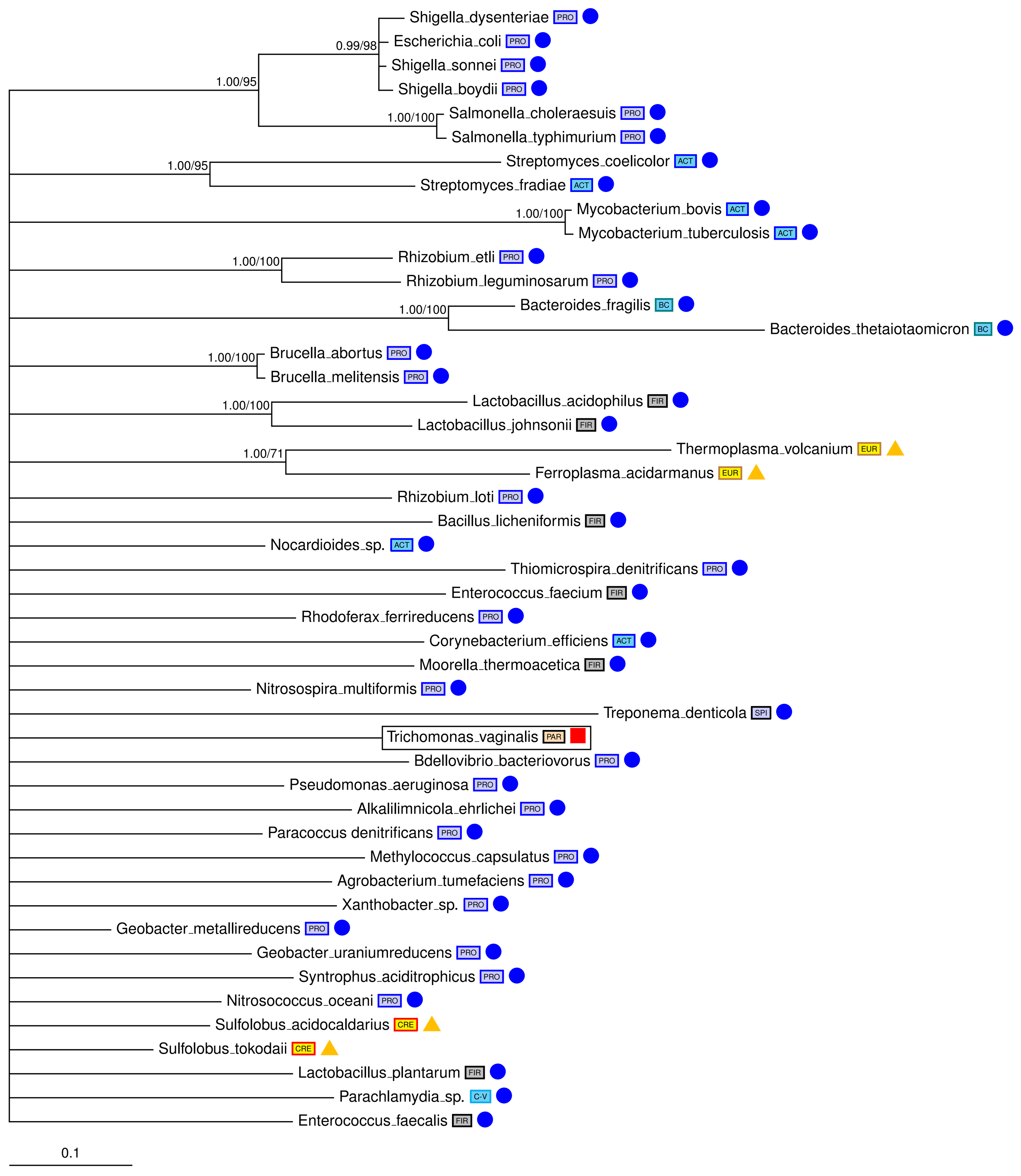

TN068

```
              Candy accession: TV81350063
             RefSeq accession: XP_001307357.1
            Uniprot accession: A2FKX3_TRIVA
                     Comments: LGT - TV ONLY
             Species affected: TV
        Adjacent taxa in tree: Bacteria
EC annotation - (Blast/Profile): na
                   PHOBIUS SP: 0
                  PHOBIUS TMD: 12
            RefSeq annotation: hypothetical protein
      Name of enzyme/protein: Hypothetical protein
      KEGG PATHWAY - level 1: Function unknown
      KEGG PATHWAY - level 2: na
```

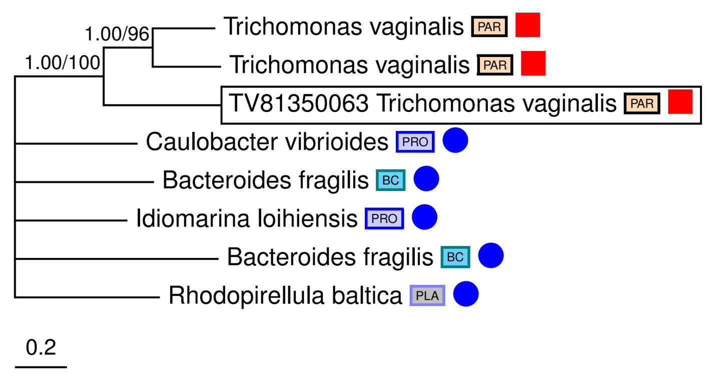

TN069

```
                  Candy accession: TV82114022
                 RefSeq accession: XP_001301892.1
                Uniprot accession: A2G1K4_TRIVA
                         Comments: LGT - TV ONLY
                 Species affected: TV
            Adjacent taxa in tree: Bacteroidetes/Chlorobi - Bacteroides
EC annotation - (Blast/Profile): EC:2.7.1.162
                      PHOBIUS SP: 0
                     PHOBIUS TMD: 0
                RefSeq annotation: hypothetical protein
         Name of enzyme/protein: N-acetylhexosamine 1-kinase
          KEGG PATHWAY - level 1: Reaction
          KEGG PATHWAY - level 2: Reaction
```

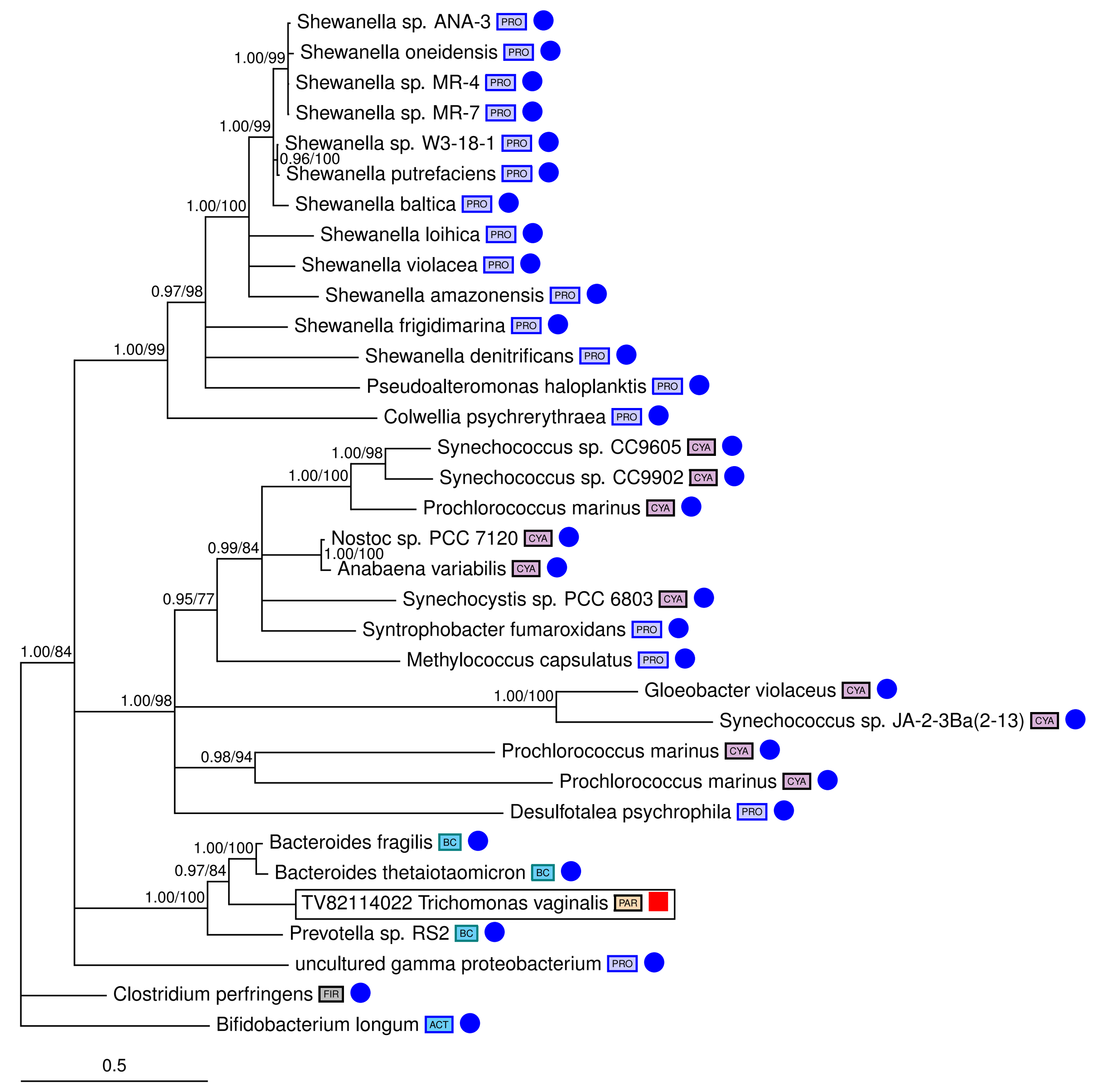

TN070

```
              Candy accession: TV83237063
             RefSeq accession: XP_001309866.1
            Uniprot accession: A2FDT7_TRIVA
                     Comments: LGT - TV ONLY
             Species affected: TV
       Adjacent taxa in tree: Bacteria
EC annotation - (Blast/Profile): na
                  PHOBIUS SP: 0
                 PHOBIUS TMD: 0
           RefSeq annotation: hypothetical protein
      Name of enzyme/protein: Protein containg DUF3111
      KEGG PATHWAY - level 1: Function unknown
      KEGG PATHWAY - level 2: na
```

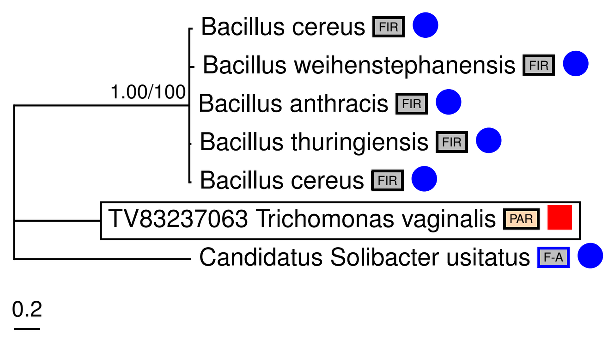

TN071

```
               Candy accession: TV83928086
              RefSeq accession: XP_001309751.1
             Uniprot accession: A2FE49_TRIVA
                      Comments: LGT - TV ONLY
              Species affected: TV
          Adjacent taxa in tree: Actinobacteria - Bifidobacterium
EC annotation - (Blast/Profile): EC:4.1.1.44
                     PHOBIUS SP: 0
                    PHOBIUS TMD: 0
              RefSeq annotation: 4-carboxymuconolactone decarboxylase
         Name of enzyme/protein: 4-carboxymuconolactone decarboxylase
         KEGG PATHWAY - level 1: Xenobiotics Biodegradation and
                                 Metabolism
         KEGG PATHWAY - level 2: Benzoate degradation
```

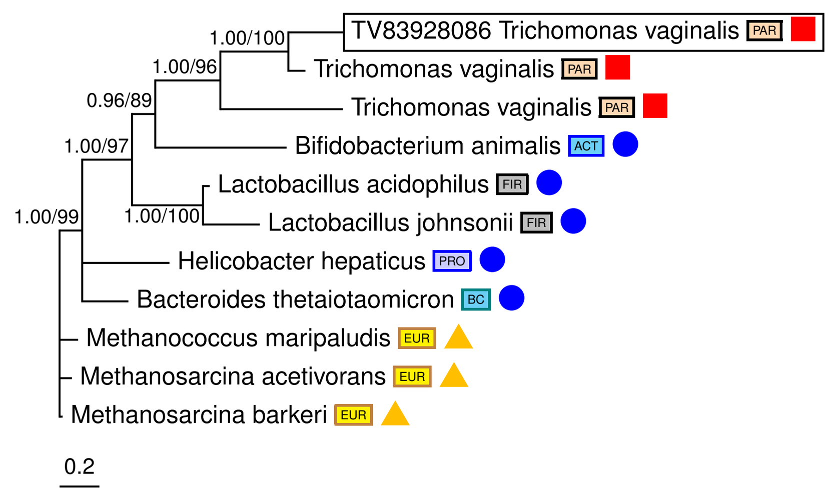

TN072

```
              Candy accession: TV84285194
             RefSeq accession: XP_001323014.1
            Uniprot accession: A2E9A6_TRIVA
                     Comments: LGT - TV ONLY
             Species affected: TV
          Adjacent taxa in tree: Bacteria
EC annotation - (Blast/Profile): na
                   PHOBIUS SP: 0
                  PHOBIUS TMD: 0
            RefSeq annotation: NDP-hexose 3,4-dehydratase
      Name of enzyme/protein: Predicted 3-amino-5-hydroxybenzoic acid
                               synthase family
      KEGG PATHWAY - level 1: Other function
      KEGG PATHWAY - level 2: na
```

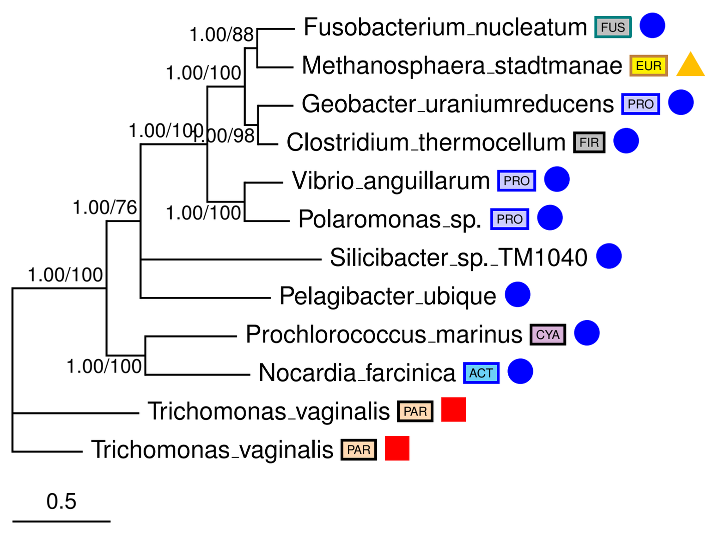

TN073

```
            Candy accession: TV85232129
           RefSeq accession: XP_001320703.1
          Uniprot accession: A2EFS9_TRIVA
                   Comments: LGT - TV ONLY
           Species affected: TV
      Adjacent taxa in tree: Bacteria
EC annotation - (Blast/Profile): EC:4.4.1.5
                 PHOBIUS SP: 0
                PHOBIUS TMD: 0
          RefSeq annotation: glyoxalase
   Name of enzyme/protein: lactoylglutathione lyase
    KEGG PATHWAY - level 1: Carbohydrate Metabolism
    KEGG PATHWAY - level 2: Pyruvate metabolism
```

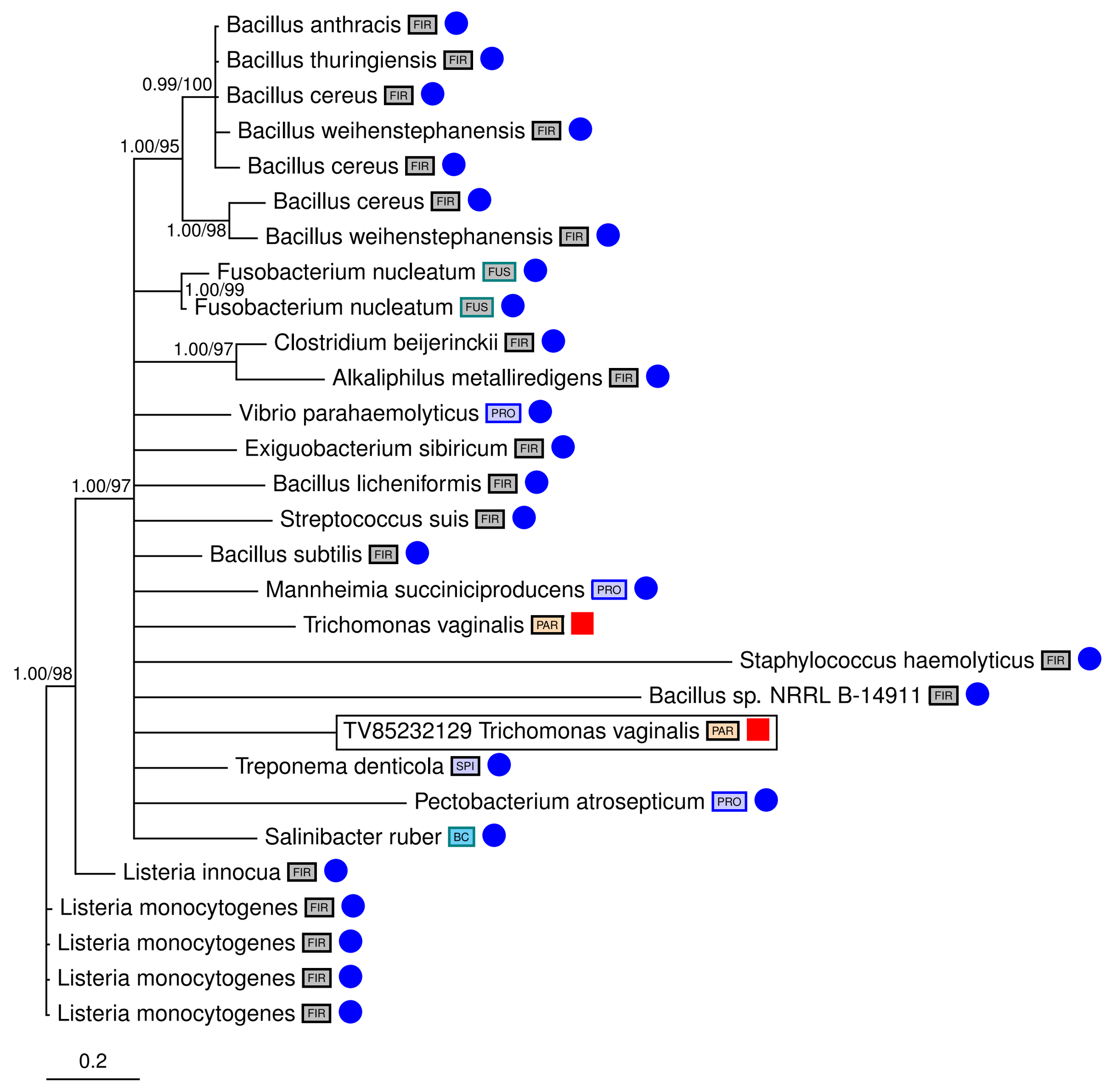

TN074

```
                  Candy accession: TV85337301
                 RefSeq accession: XP_001329100.1
                Uniprot accession: A2DRT0_TRIVA
                         Comments: LGT - TV ONLY
                 Species affected: TV
            Adjacent taxa in tree: Bacteria
EC annotation - (Blast/Profile): EC:5.3.2.-
                       PHOBIUS SP: 0
                      PHOBIUS TMD: 0
                RefSeq annotation: Tautomerase enzyme family protein
          Name of enzyme/protein: Tautomerase enzyme family protein
          KEGG PATHWAY - level 1: Reaction
          KEGG PATHWAY - level 2: Reaction
```

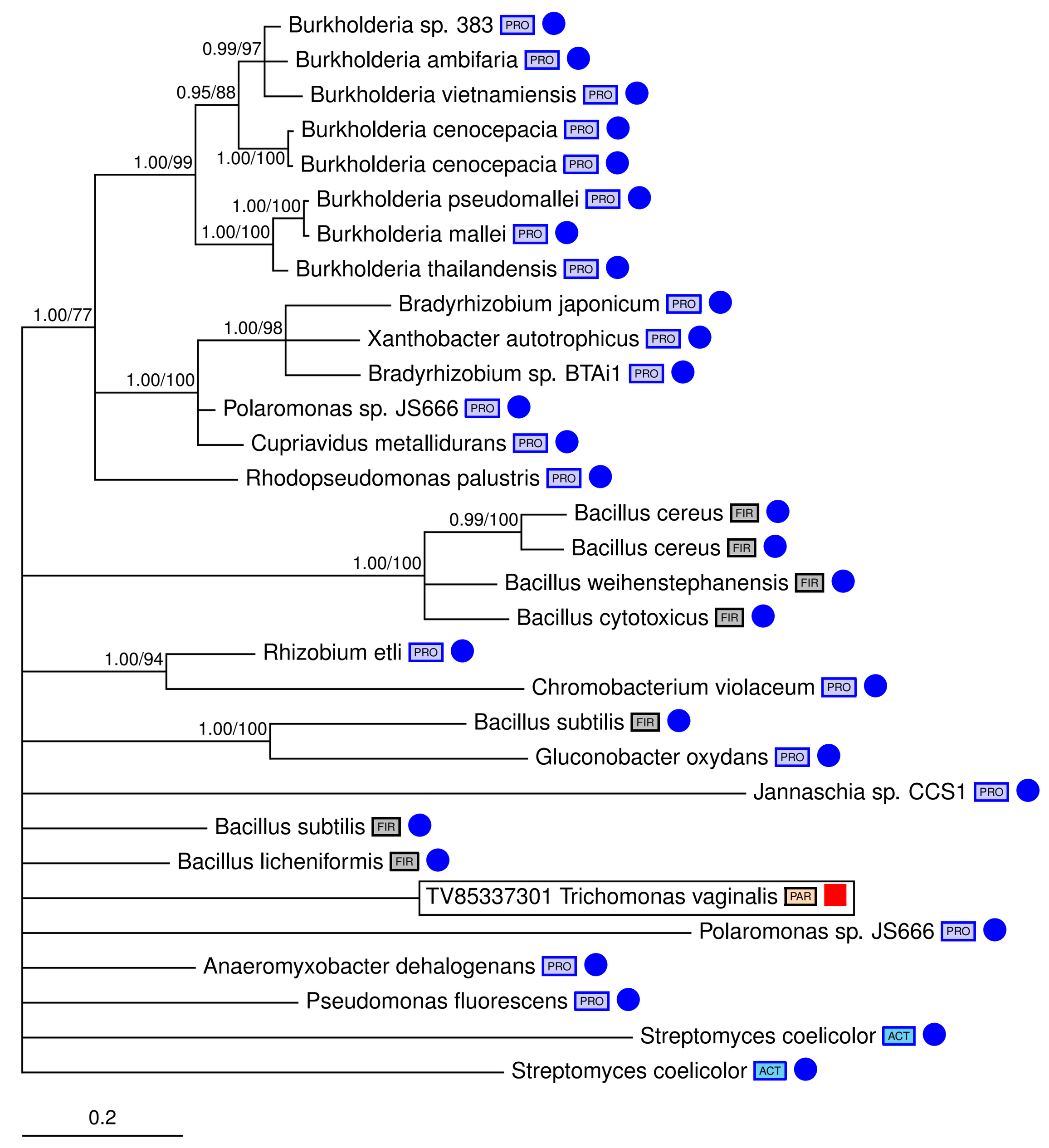

TN075

```
              Candy accession: TV85862250
             RefSeq accession: XP_001325565.1
            Uniprot accession: A2E1Z3_TRIVA
                     Comments: LGT - TV ONLY
             Species affected: TV
       Adjacent taxa in tree: Prokaryotes
EC annotation - (Blast/Profile): na
                   PHOBIUS SP: 0
                  PHOBIUS TMD: 0
            RefSeq annotation: hypothetical protein
     Name of enzyme/protein: Predicted selenium-dependent hydroxylase
                             accessory protein YqeC
     KEGG PATHWAY - level 1: Other function
     KEGG PATHWAY - level 2: na
```

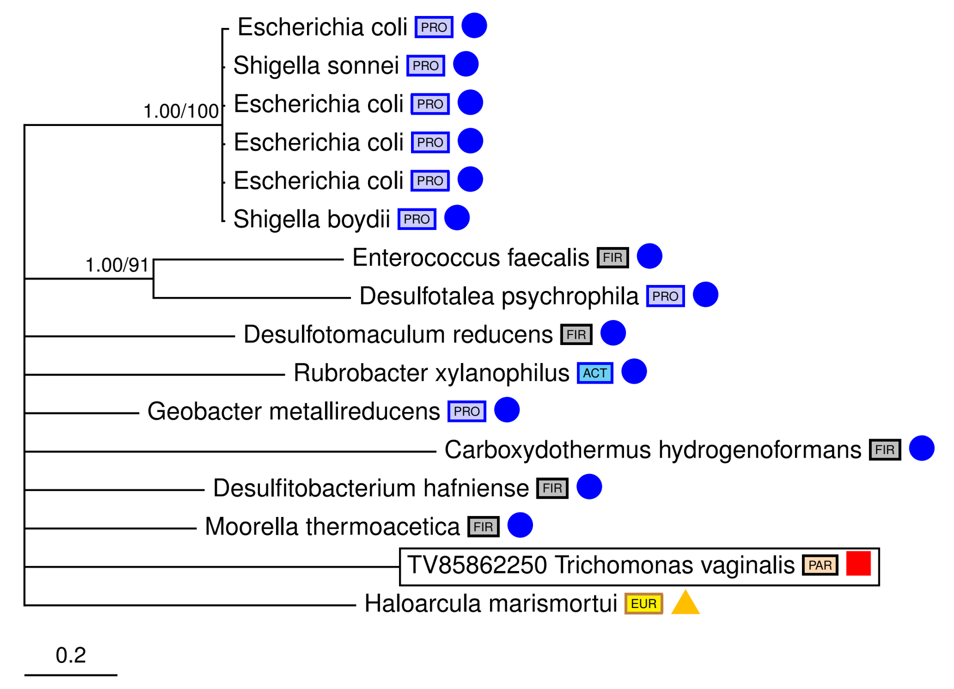

TN076

```
              Candy accession: TV85914041
             RefSeq accession: XP_001307232.1
            Uniprot accession: A2FLC1_TRIVA
                     Comments: LGT - TV ONLY
             Species affected: TV
       Adjacent taxa in tree: Bacteroides
EC annotation - (Blast/Profile): na
                   PHOBIUS SP: 0
                  PHOBIUS TMD: 0
            RefSeq annotation: hypothetical protein
        Name of enzyme/protein: Predicted iron-sulfur cluster repair di-
                               iron protein
       KEGG PATHWAY - level 1: Other function
       KEGG PATHWAY - level 2: na
```

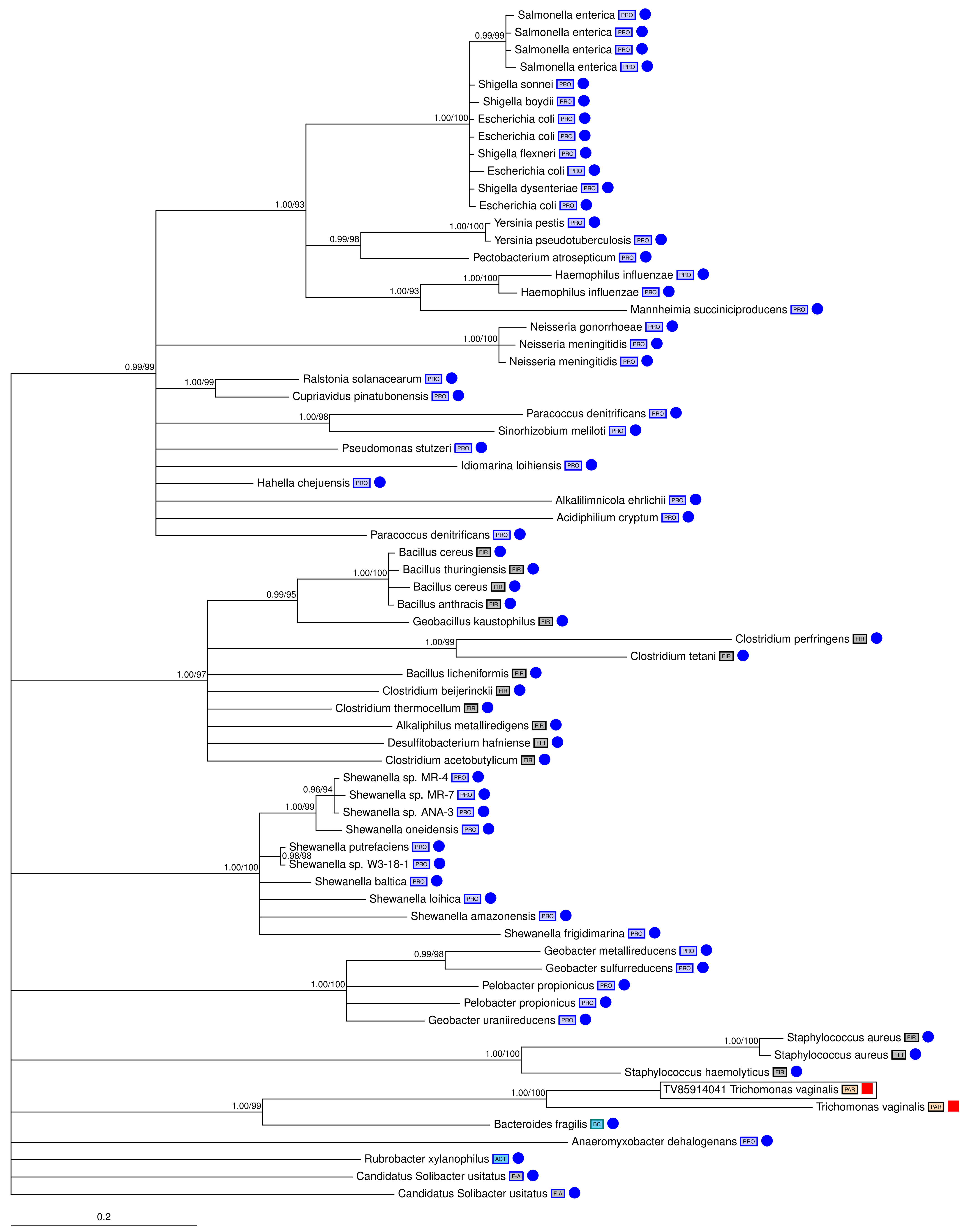

TN077

```
               Candy accession: TV86120111
              RefSeq accession: XP_001315078.1
             Uniprot accession: A2EWU0_TRIVA
                      Comments: LGT - TV ONLY
              Species affected: TV
         Adjacent taxa in tree: Prokaryotes
EC annotation - (Blast/Profile): na
                    PHOBIUS SP: 0
                   PHOBIUS TMD: 0
             RefSeq annotation: acetyltransferase, GNAT family-related
                                protein
      Name of enzyme/protein: Predicted acetyltransferase
      KEGG PATHWAY - level 1: Other function
      KEGG PATHWAY - level 2: na
```

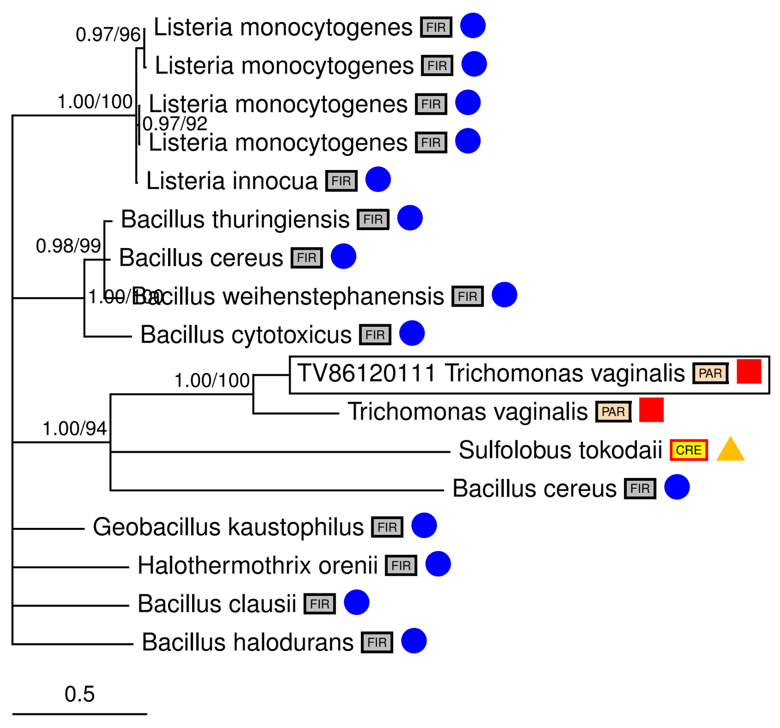

TN078

```
             Candy accession: TV86507075
            RefSeq accession: XP_001305824.1
           Uniprot accession: A2FQA4_TRIVA
                    Comments: LGT - TV ONLY
            Species affected: TV
       Adjacent taxa in tree: Bacteria
EC annotation - (Blast/Profile): na
                  PHOBIUS SP: 0
                 PHOBIUS TMD: 2
           RefSeq annotation: TrkA-C domain containing protein
     Name of enzyme/protein: TrkA-C domain containing protein
      KEGG PATHWAY - level 1: Function unknown
      KEGG PATHWAY - level 2: na
```

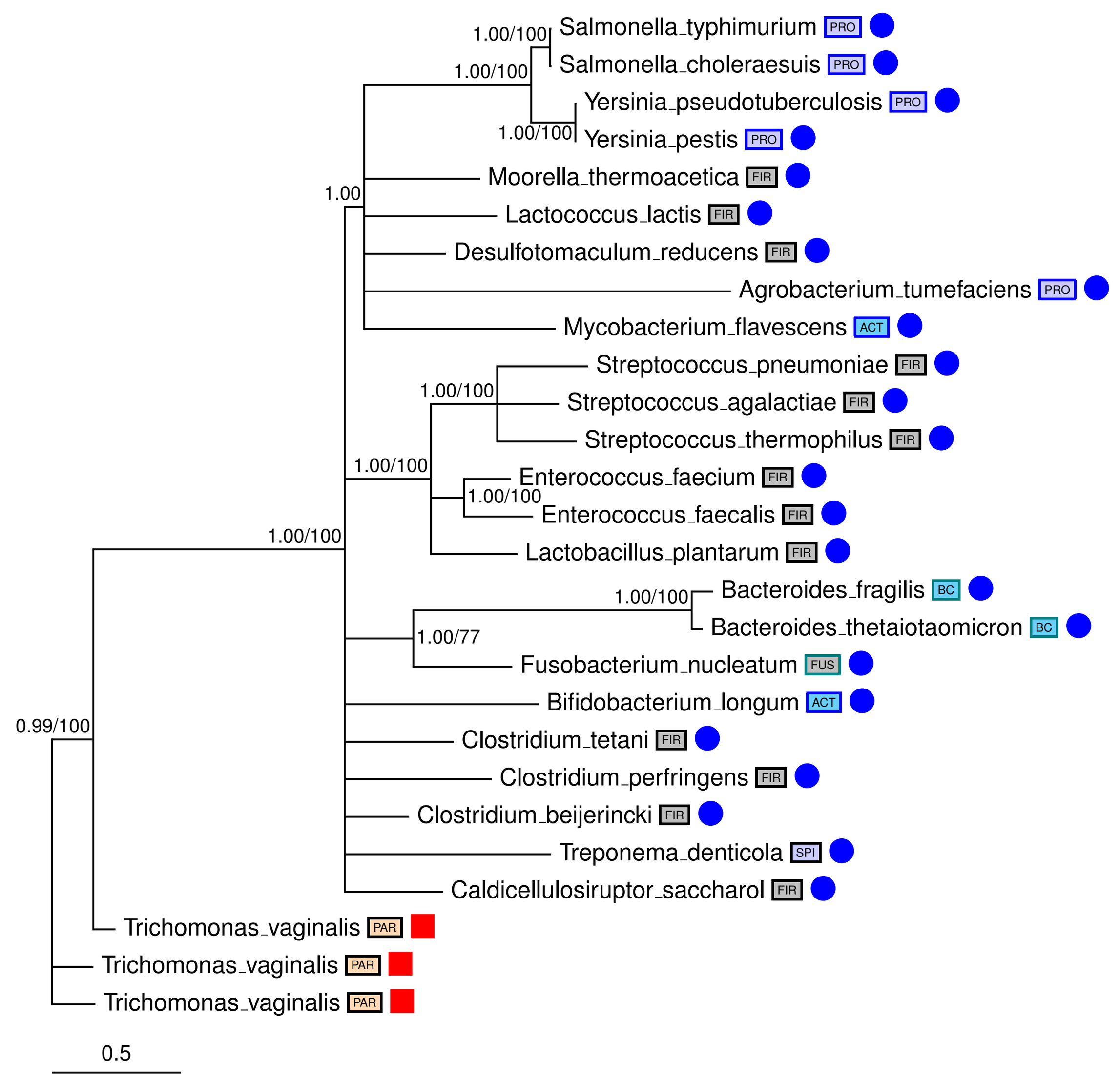

TN079

```
                Candy accession: TV86576190
               RefSeq accession: XP_001327976.1
              Uniprot accession: A2DV26_TRIVA
                       Comments: LGT - TV ONLY
               Species affected: TV
          Adjacent taxa in tree: Prokaryotes
EC annotation - (Blast/Profile): na
                     PHOBIUS SP: 0
                    PHOBIUS TMD: 0
              RefSeq annotation: hypothetical protein
         Name of enzyme/protein: Predicted flavodoxin
         KEGG PATHWAY - level 1: Other function
         KEGG PATHWAY - level 2: na
```

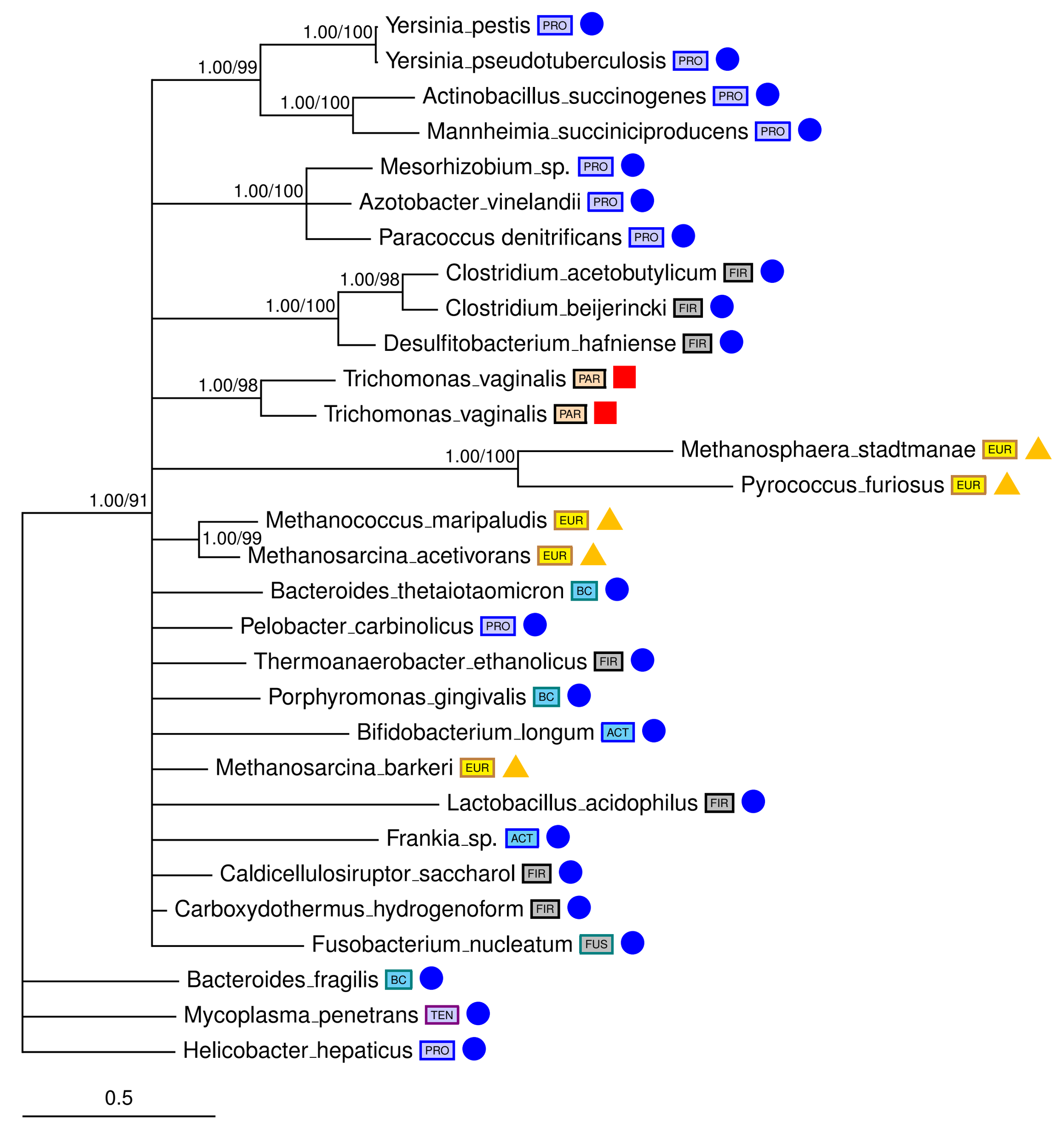

TN080

Candy accession:	TV86997188
RefSeq accession:	XP_001327447.1
Uniprot accession:	A2DWN9_TRIVA
Comments:	LGT - TV ONLY
Species affected:	TV
Adjacent taxa in tree:	Firmicutes
EC annotation - (Blast/Profile):	EC:3.2.1.4
PHOBIUS SP:	0
PHOBIUS TMD:	0
RefSeq annotation:	xylanase
Name of enzyme/protein:	endo-1,4-beta-D-glucanase
KEGG PATHWAY - level 1:	Carbohydrate Metabolism
KEGG PATHWAY - level 2:	Starch and sucrose metabolism

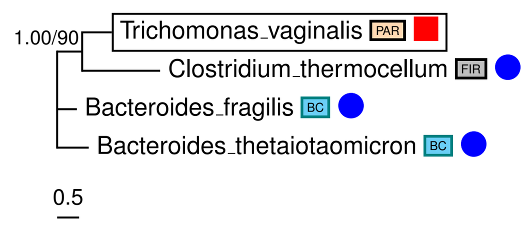

TN081

```
             Candy accession: TV86997261
            RefSeq accession: XP_001327422.1
           Uniprot accession: A2DWL4_TRIVA
                    Comments: LGT - TV ONLY
            Species affected: TV
        Adjacent taxa in tree: Bacteria
EC annotation - (Blast/Profile): na
                  PHOBIUS SP: 0
                 PHOBIUS TMD: 10
           RefSeq annotation: YeeE/YedE family protein
      Name of enzyme/protein: YeeE/YedE family protein
      KEGG PATHWAY - level 1: Other function - Membrane transport
      KEGG PATHWAY - level 2: na
```

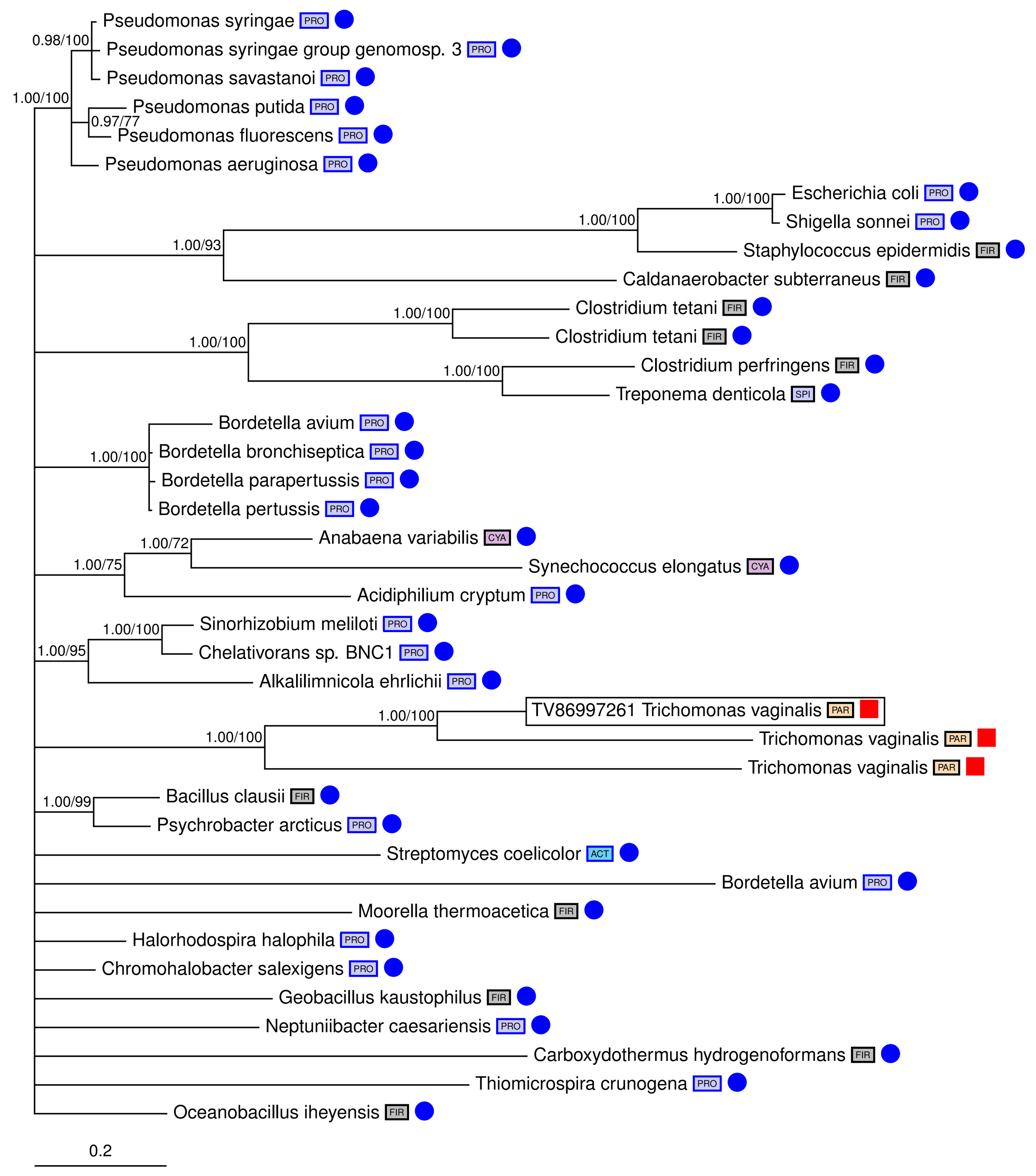

TN082

Candy accession: TV87363164
RefSeq accession: XP_001325291.1
Uniprot accession: A2E2P0_TRIVA
Comments: LGT - TV ONLY
Species affected: TV
Adjacent taxa in tree: Bacteria
EC annotation - (Blast/Profile): na
PHOBIUS SP: 0
PHOBIUS TMD: 0
RefSeq annotation: ATP-dependent Zn protease
Name of enzyme/protein: Protein containing DUF1846
KEGG PATHWAY - level 1: Function unknown
KEGG PATHWAY - level 2: na

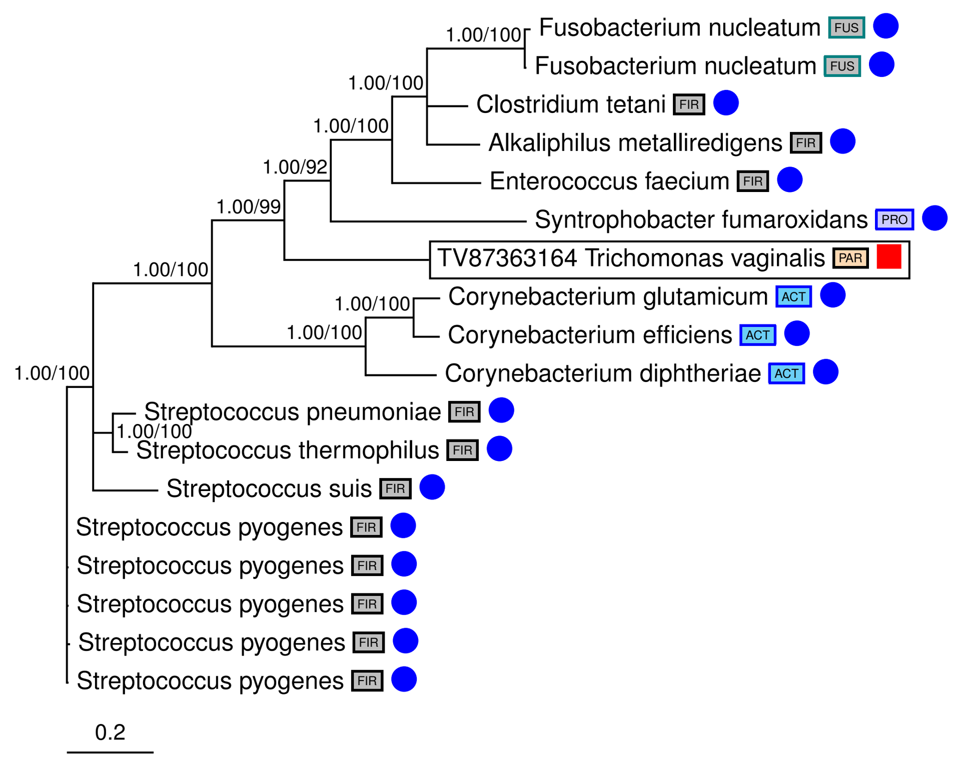

TN083

```
              Candy accession: TV87719223
             RefSeq accession: XP_001325053.1
            Uniprot accession: A2E3D9_TRIVA
                     Comments: LGT - TV ONLY
             Species affected: TV
       Adjacent taxa in tree: Bacteria
EC annotation - (Blast/Profile): EC:4.4.1.5
                   PHOBIUS SP: 0
                  PHOBIUS TMD: 0
            RefSeq annotation: Lactoylglutathione lyase
     Name of enzyme/protein: lactoylglutathione lyase
     KEGG PATHWAY - level 1: Carbohydrate Metabolism
     KEGG PATHWAY - level 2: Pyruvate metabolism
```

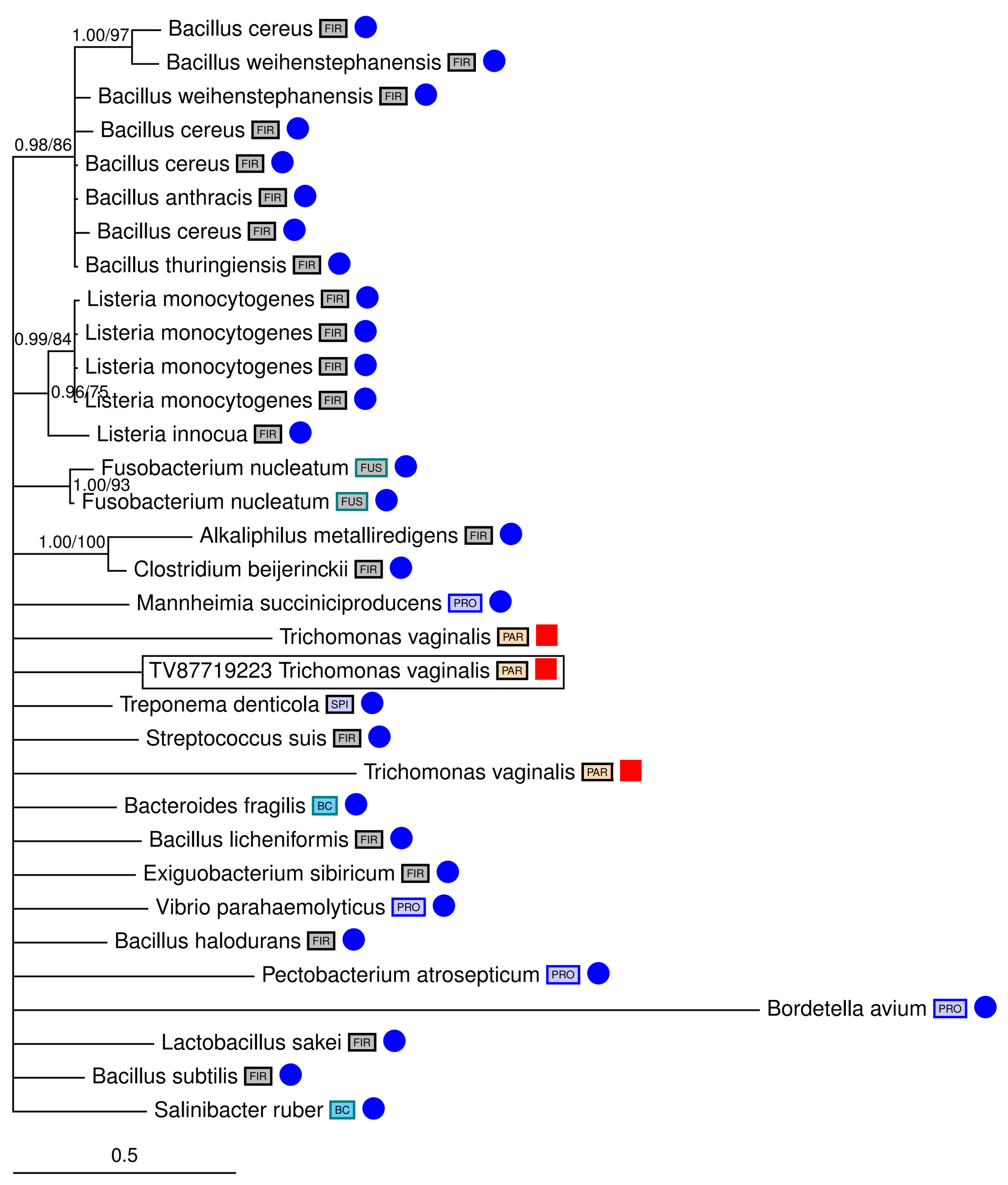

TN084

```
              Candy accession: TV87835279
             RefSeq accession: XP_001327568.1
            Uniprot accession: A2DW44_TRIVA
                     Comments: LGT - TV ONLY
             Species affected: TV
        Adjacent taxa in tree: Bacteria
EC annotation - (Blast/Profile): na
                   PHOBIUS SP: 0
                  PHOBIUS TMD: 0
            RefSeq annotation: hypothetical protein
        Name of enzyme/protein: Hypothetical protein
       KEGG PATHWAY - level 1: Function unknown
       KEGG PATHWAY - level 2: na
```

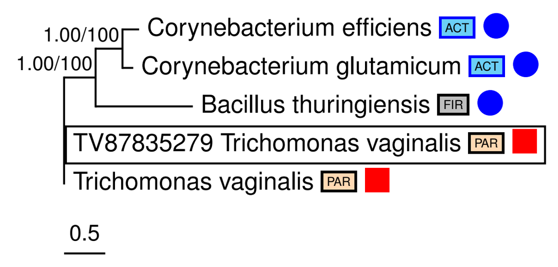

TN085

```
                Candy accession: TV87854275
               RefSeq accession: XP_001578953.1
              Uniprot accession: A2DNX2_TRIVA
                       Comments: LGT - TV ONLY
               Species affected: TV
           Adjacent taxa in tree: Archaea - Pyrobaculum
EC annotation - (Blast/Profile): na
                     PHOBIUS SP: 0
                    PHOBIUS TMD: 0
              RefSeq annotation: hypothetical protein
         Name of enzyme/protein: Predicted L-ara-isomerases L-fucose
                                 isomerase (FucIase) and L-arabinose
                                 isomerase (AI) family
         KEGG PATHWAY - level 1: Other function
         KEGG PATHWAY - level 2: na
```

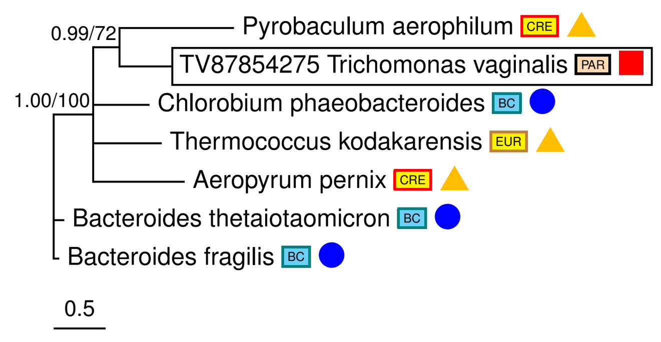

TN086

```
                Candy accession: TV88428070
               RefSeq accession: XP_001307942.1
              Uniprot accession: A2FJ98_TRIVA
                       Comments: LGT - TV ONLY - ONE PHAGE, ONE PLASMID
               Species affected: TV
           Adjacent taxa in tree: Firmicutes
EC annotation - (Blast/Profile): na
                    PHOBIUS SP: 0
                   PHOBIUS TMD: 0
              RefSeq annotation: Transposase family protein
          Name of enzyme/protein: Transposase family protein
          KEGG PATHWAY - level 1: Other function - Genetic Information
                                  Processing
          KEGG PATHWAY - level 2: na
```

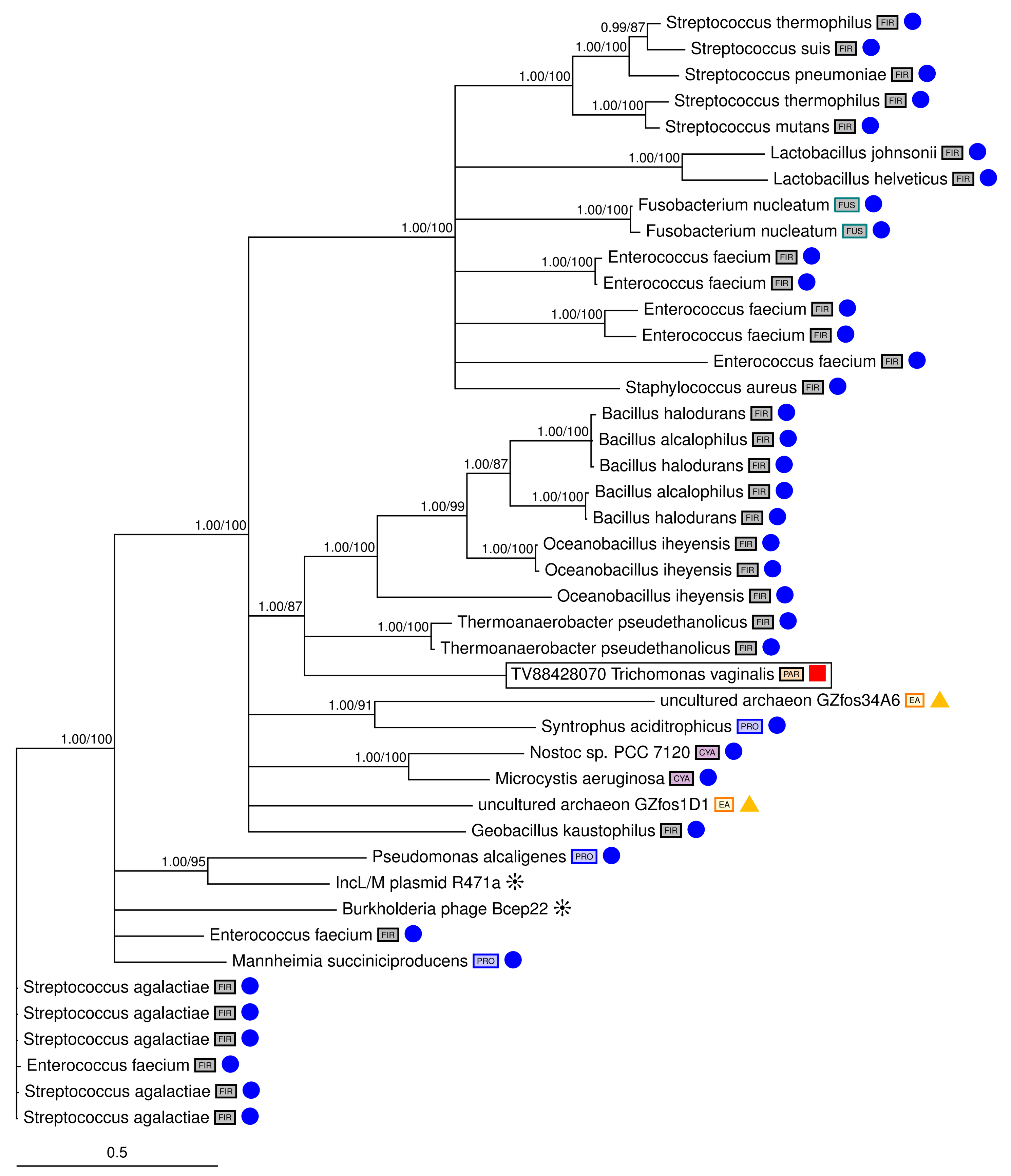

TN087

```
                Candy accession: TV88428077
               RefSeq accession: XP_001307936.1
              Uniprot accession: A2FJ92_TRIVA
                       Comments: LGT - TV ONLY
               Species affected: TV
           Adjacent taxa in tree: Bacteria
EC annotation - (Blast/Profile): EC:2.6.1.5
                     PHOBIUS SP: 0
                    PHOBIUS TMD: 0
              RefSeq annotation: transcriptional regulator, gntR family
                                 protein
         Name of enzyme/protein: tyrosine transaminase
         KEGG PATHWAY - level 1: Amino Acid Metabolism
         KEGG PATHWAY - level 2: Tyrosine metabolism
```

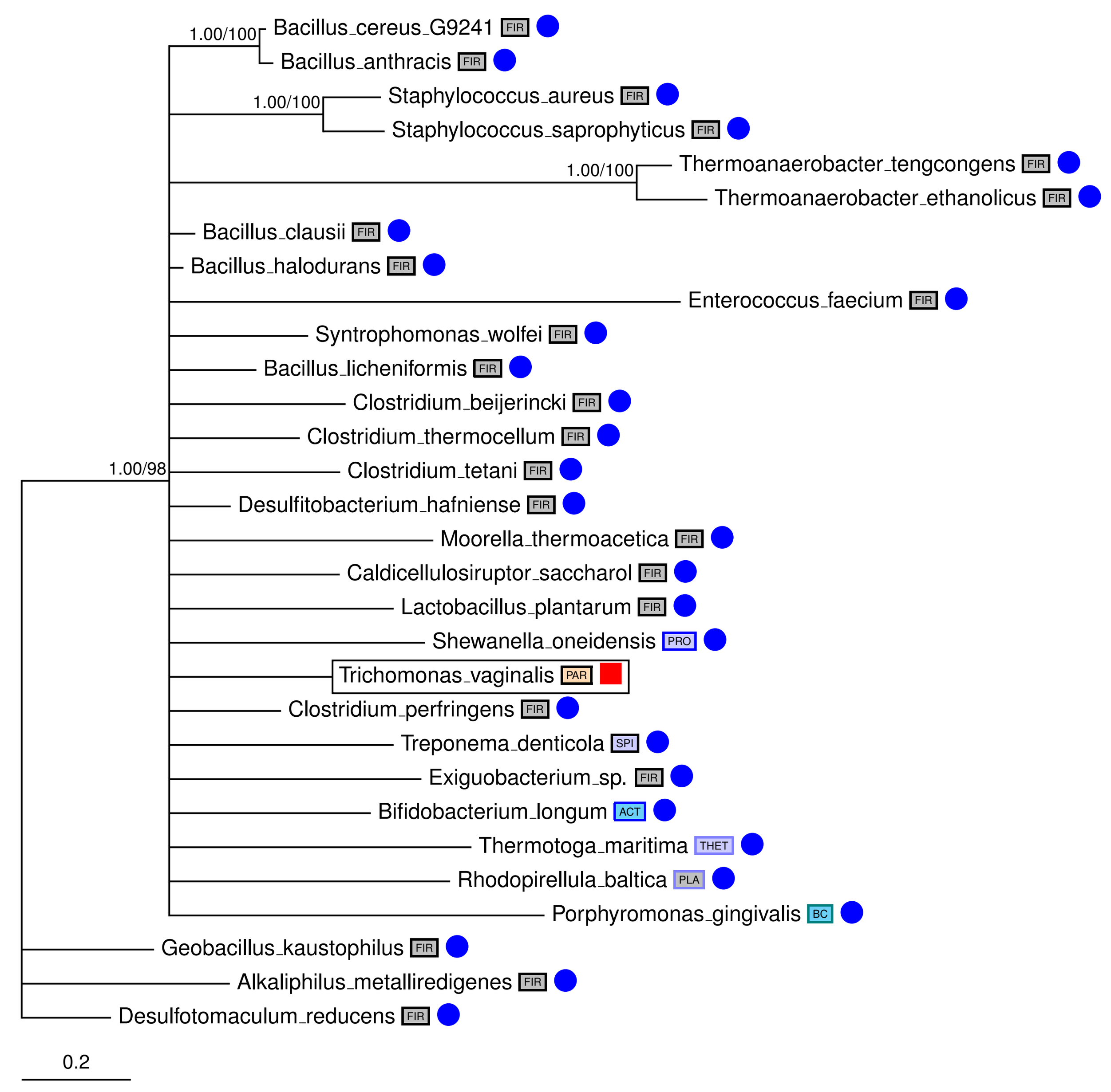

TN088

```
               Candy accession: TV88428086
              RefSeq accession: XP_001307935.1
             Uniprot accession: A2FJ91_TRIVA
                      Comments: LGT - TV ONLY
              Species affected: TV
         Adjacent taxa in tree: Firmicutes
EC annotation - (Blast/Profile): na
                    PHOBIUS SP: 0
                   PHOBIUS TMD: 0
             RefSeq annotation: Transposase IS116/IS110/IS902 family
                                protein
        Name of enzyme/protein: Transposase IS116/IS110/IS902 family
                                protein
        KEGG PATHWAY - level 1: Other function - Genetic Information
                                Processing
        KEGG PATHWAY - level 2: na
```

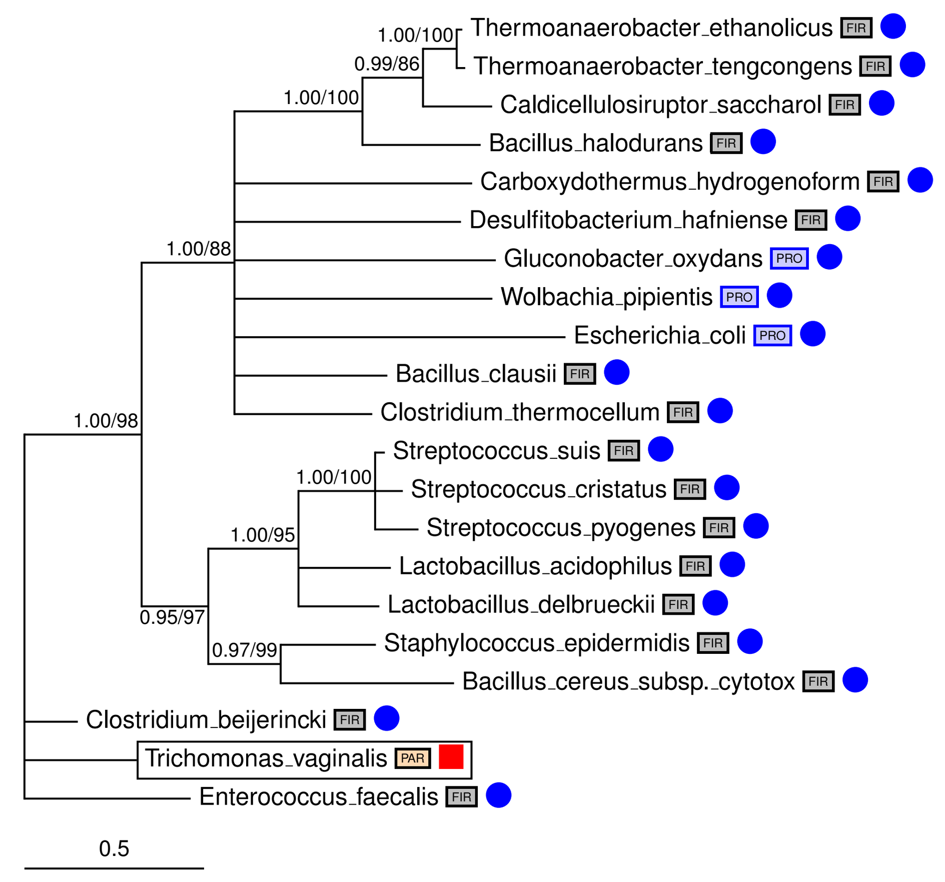

TN089

```
                Candy accession: TV88428091
               RefSeq accession: XP_001307960.1
              Uniprot accession: A2FJB6_TRIVA
                       Comments: LGT - TV ONLY
               Species affected: TV
          Adjacent taxa in tree: Bacteria
EC annotation - (Blast/Profile): na
                     PHOBIUS SP: 0
                    PHOBIUS TMD: 0
             RefSeq annotation: transcription elongation factor
          Name of enzyme/protein: Predicted transcription elongation
                                  factor
          KEGG PATHWAY - level 1: Other function - Genetic Information
                                  Processing
          KEGG PATHWAY - level 2: na
```

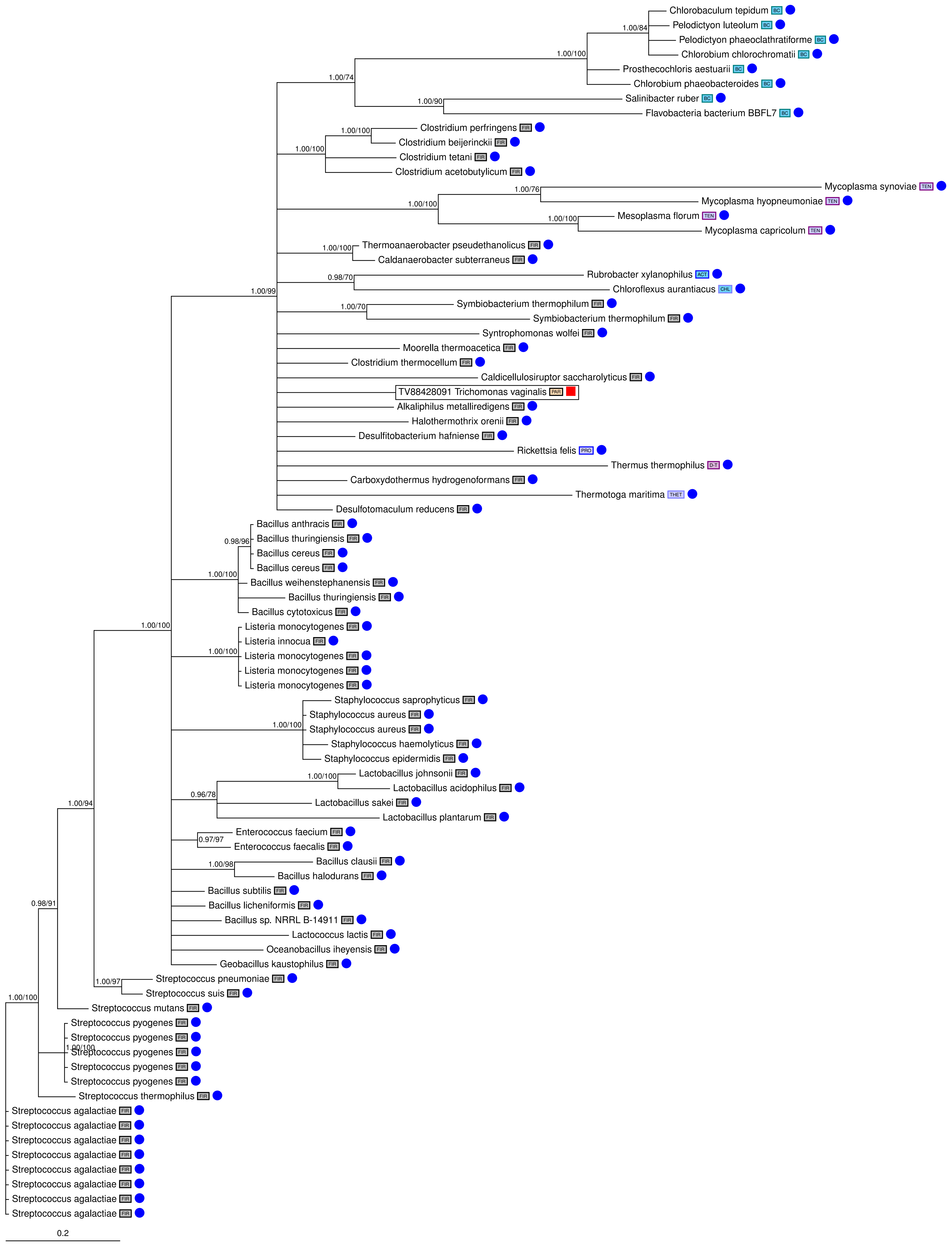

TN090

```
                Candy accession: TV88613086
               RefSeq accession: XP_001316524.1
              Uniprot accession: A2ESR0_TRIVA
                       Comments: LGT - TV ONLY
               Species affected: TV
          Adjacent taxa in tree: Bacteria
EC annotation - (Blast/Profile): na
                     PHOBIUS SP: 0
                    PHOBIUS TMD: 0
              RefSeq annotation: hypothetical protein
         Name of enzyme/protein: Predicted Zn-binding protein
         KEGG PATHWAY - level 1: Other function
         KEGG PATHWAY - level 2: na
```

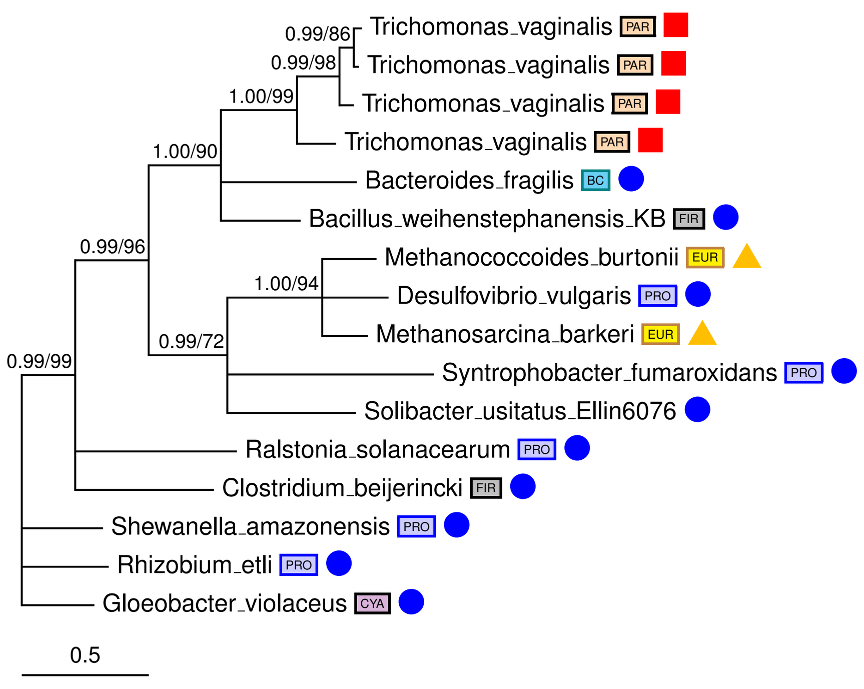

TN091

```
                 Candy accession: TV88807024
                RefSeq accession: XP_001302201.1
               Uniprot accession: A2G0P6_TRIVA
                        Comments: LGT - TV ONLY
                Species affected: TV
          Adjacent taxa in tree: Firmicutes
EC annotation - (Blast/Profile): EC:3.1.4.14
                      PHOBIUS SP: 0
                     PHOBIUS TMD: 0
              RefSeq annotation: Flavodoxin-like fold family protein
          Name of enzyme/protein: Phosphodiesterase
          KEGG PATHWAY - level 1: Metabolism of Cofactors and Vitamins
          KEGG PATHWAY - level 2: Pantothenate and CoA biosynthesis
```

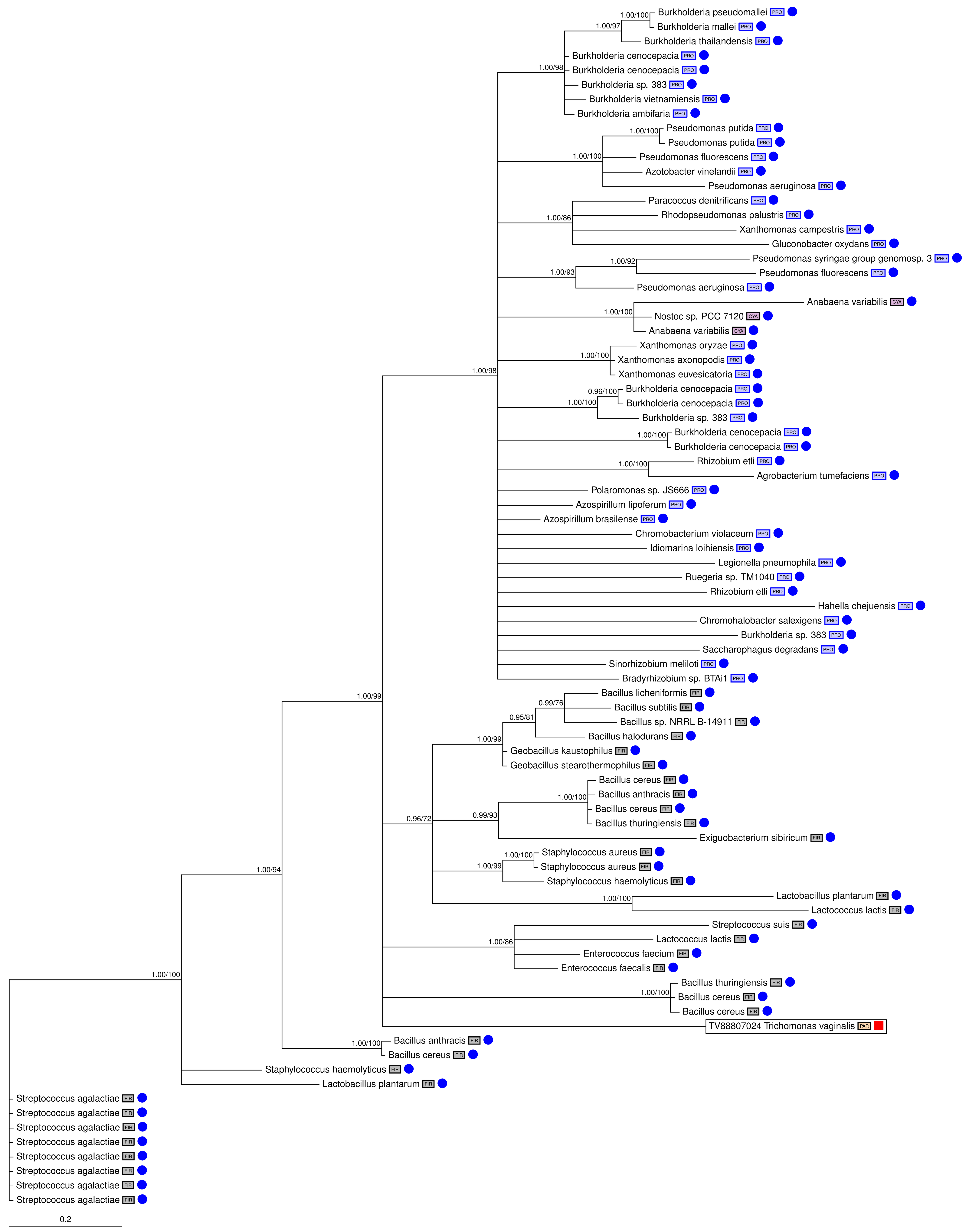

TN092

Candy accession: TV89386134
RefSeq accession: XP_001322324.1
Uniprot accession: A2EB55_TRIVA
Comments: LGT - TV ONLY
Species affected: TV
Adjacent taxa in tree: Bacteria
EC annotation - (Blast/Profile): na
PHOBIUS SP: 0
PHOBIUS TMD: 0
RefSeq annotation: hypothetical protein
Name of enzyme/protein: Protein containing DUF1015
KEGG PATHWAY - level 1: Function unknown
KEGG PATHWAY - level 2: na

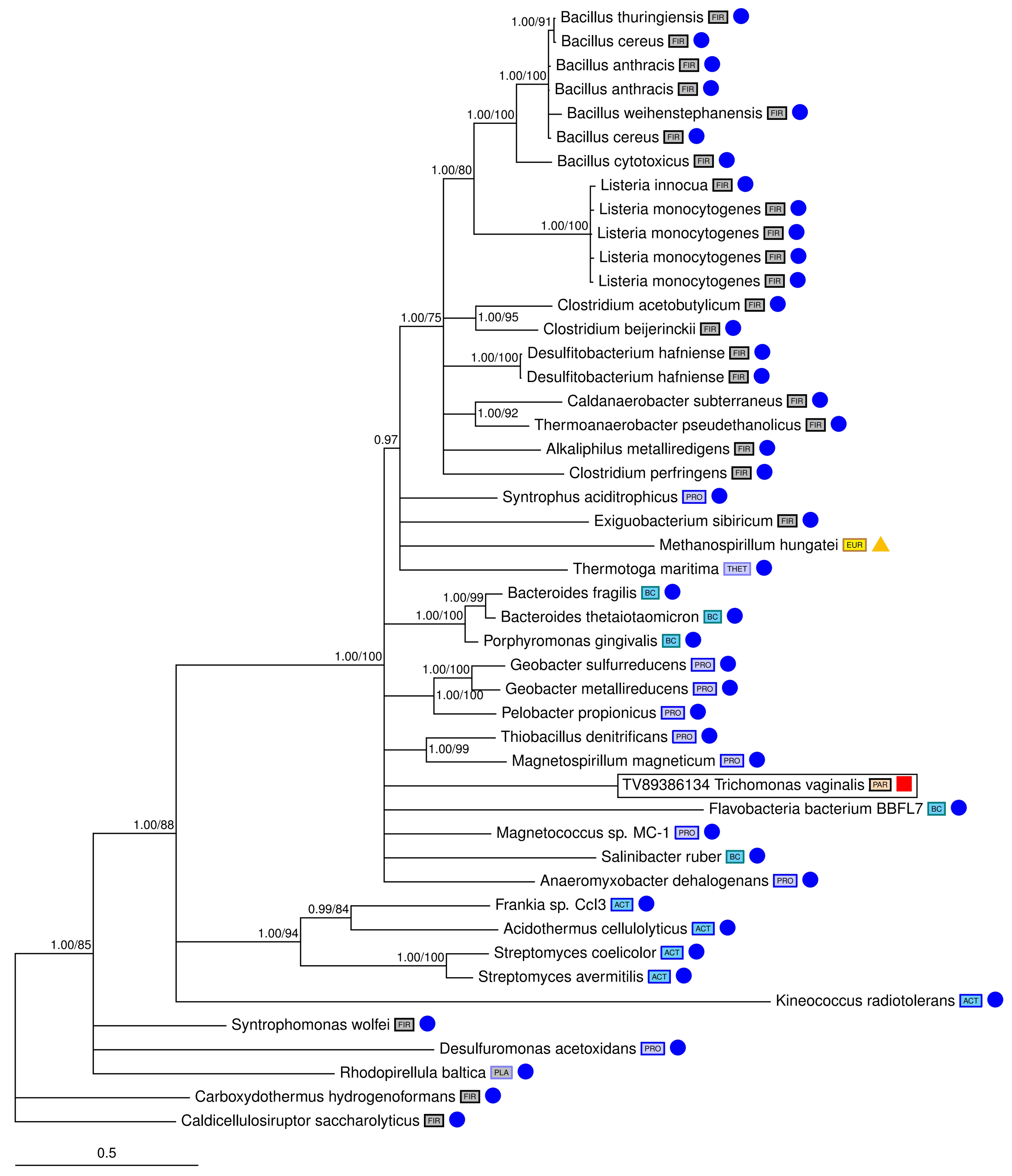

TN093

```
                Candy accession: TV89789075
              RefSeq accession: XP_001306702.1
             Uniprot accession: A2FMV5_TRIVA
                      Comments: LGT - TV ONLY
              Species affected: TV
           Adjacent taxa in tree: Bacteria
EC annotation - (Blast/Profile): EC:1.1.1.193
                    PHOBIUS SP: 0
                   PHOBIUS TMD: 0
             RefSeq annotation: RibD C-terminal domain containing
                                protein
          Name of enzyme/protein: 5-amino-6-(5-phosphoribosylamino)uracil
                                reductase
          KEGG PATHWAY - level 1: Metabolism of Cofactors and Vitamins
          KEGG PATHWAY - level 2: Riboflavin metabolism
```

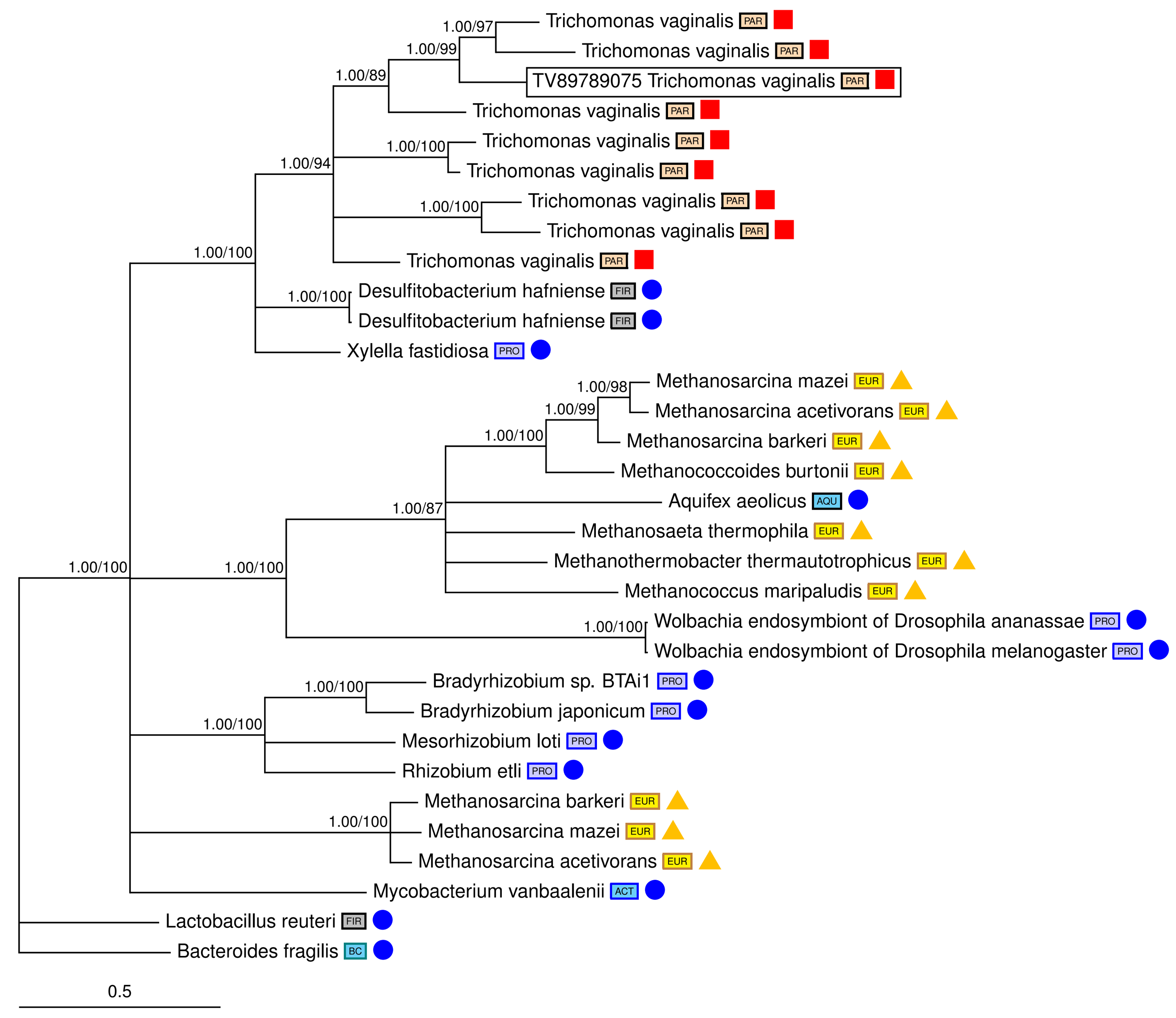

TN094

Candy accession:	TV90417119
RefSeq accession:	XP_001320377.1
Uniprot accession:	A2EGR6_TRIVA
Comments:	LGT - TV ONLY
Species affected:	TV
Adjacent taxa in tree:	Prokaryotes
EC annotation - (Blast/Profile):	na
PHOBIUS SP:	0
PHOBIUS TMD:	0
RefSeq annotation:	hypothetical protein
Name of enzyme/protein:	Protein containing carbohydrate kinase domain
KEGG PATHWAY - level 1:	Other function
KEGG PATHWAY - level 2:	na

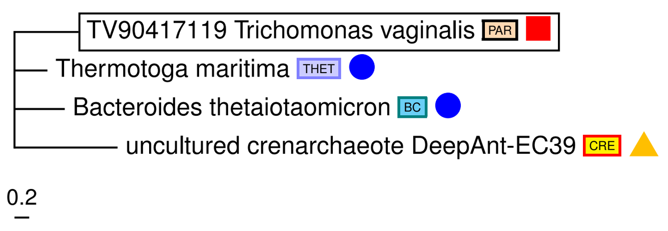

TN095

Candy accession:	TV90492230
RefSeq accession:	XP_001579117.1
Uniprot accession:	A2DNE3_TRIVA
Comments:	LGT - TV ONLY
Species affected:	TV
Adjacent taxa in tree:	Archaea
EC annotation - (Blast/Profile):	na
PHOBIUS SP:	0
PHOBIUS TMD:	0
RefSeq annotation:	hypothetical protein
Name of enzyme/protein:	Protein containing DUF3737
KEGG PATHWAY - level 1:	Function unknown
KEGG PATHWAY - level 2:	na

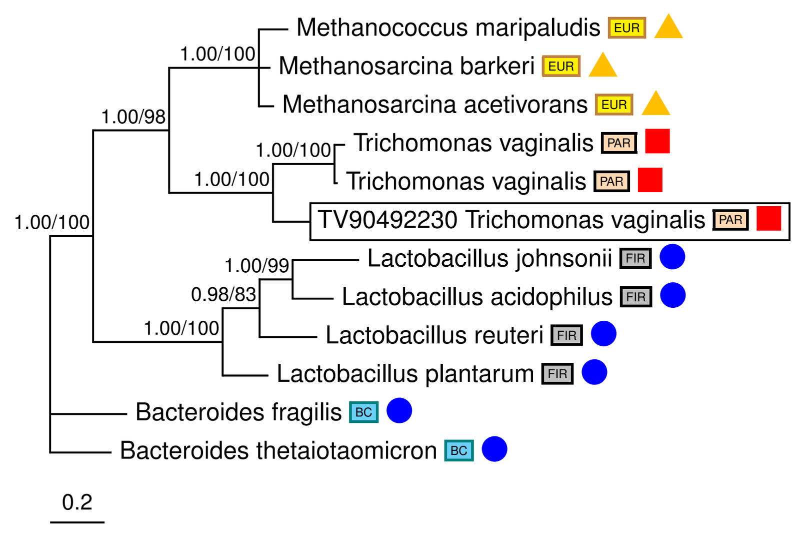

TN096

```
                Candy accession: TV90569185
               RefSeq accession: XP_001320437.1
              Uniprot accession: A2EGL9_TRIVA
                       Comments: LGT - TV ONLY
               Species affected: TV
          Adjacent taxa in tree: Firmicutes - Clostridia
EC annotation - (Blast/Profile): na
                     PHOBIUS SP: 0
                    PHOBIUS TMD: 0
              RefSeq annotation: hydrolase, NUDIX family protein
        Name of enzyme/protein: Putative hydrolase, NUDIX family protein
        KEGG PATHWAY - level 1: Other function
        KEGG PATHWAY - level 2: na
```

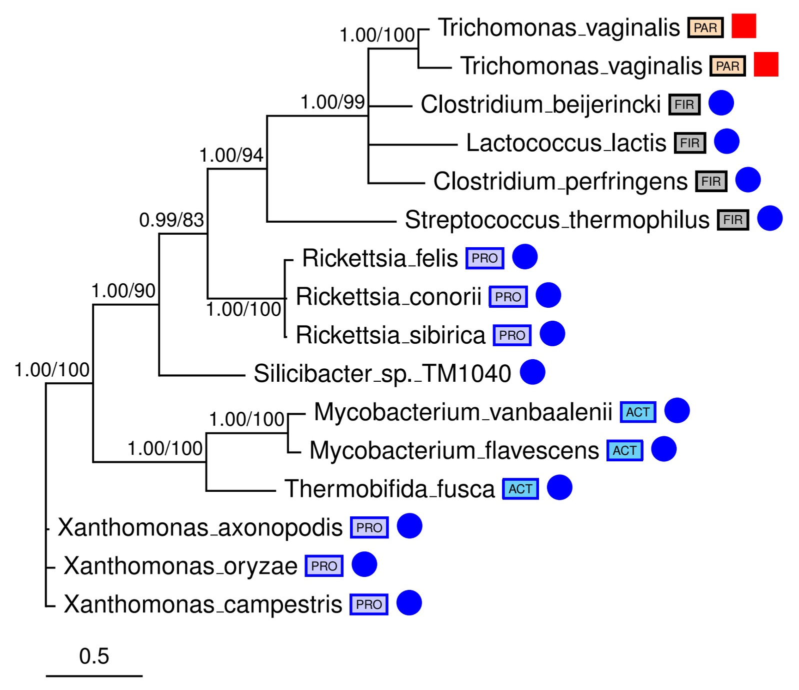

TN097

```
               Candy accession: TV92145135
              RefSeq accession: XP_001319335.1
             Uniprot accession: A2EJN8_TRIVA
                      Comments: LGT - TV ONLY
              Species affected: TV
         Adjacent taxa in tree: Bacteria
EC annotation - (Blast/Profile): na
                    PHOBIUS SP: 0
                   PHOBIUS TMD: 0
             RefSeq annotation: beta-N-acetylhexosaminidase
       Name of enzyme/protein: Protein containig F5/8 type C domain
       KEGG PATHWAY - level 1: Other function
       KEGG PATHWAY - level 2: na
```

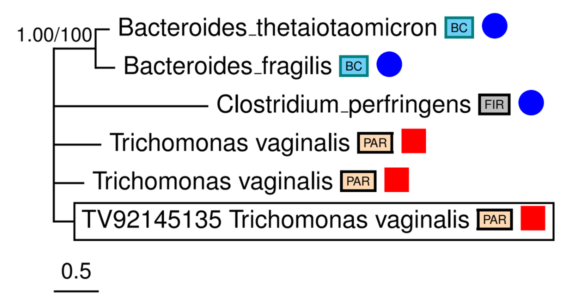

TN098

```
              Candy accession: TV94782134
             RefSeq accession: XP_001321629.1
            Uniprot accession: A2ED51_TRIVA
                     Comments: LGT - TV ONLY
             Species affected: TV
        Adjacent taxa in tree: Proteobacteria
EC annotation - (Blast/Profile): na
                   PHOBIUS SP: 0
                  PHOBIUS TMD: 1
            RefSeq annotation: hypothetical protein
      Name of enzyme/protein: Predicted disintegrin and
                              metalloprotease-like protein
       KEGG PATHWAY - level 1: Other function
       KEGG PATHWAY - level 2: na
```

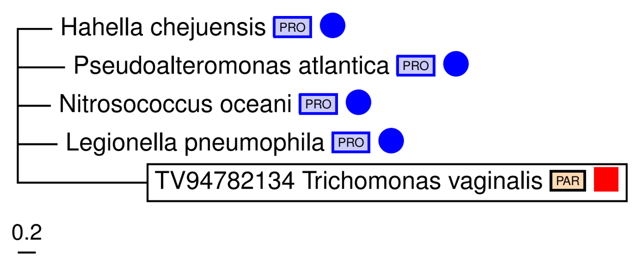

TN099

Candy accession: TV95240171
RefSeq accession: XP_001325131.1
Uniprot accession: A2E398_TRIVA
Comments: LGT - TV ONLY
Species affected: TV
Adjacent taxa in tree: Bacteria
EC annotation - (Blast/Profile): na
PHOBIUS SP: 0
PHOBIUS TMD: 0
RefSeq annotation: hypothetical protein
Name of enzyme/protein: Protein containing DUF1801
KEGG PATHWAY - level 1: Function unknown
KEGG PATHWAY - level 2: na

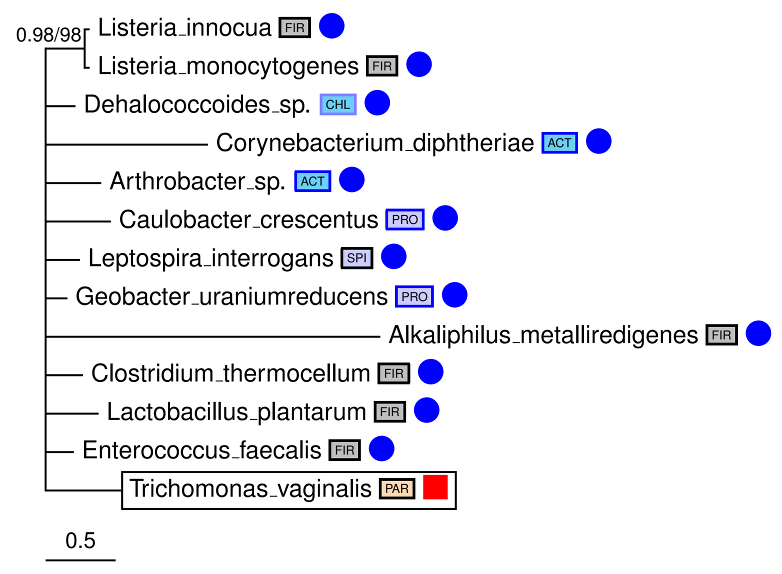

TN100

```
Candy accession: TV95569066
RefSeq accession: XP_001307274.1
Uniprot accession: A2FL88_TRIVA
Comments: LGT - TV ONLY
Species affected: TV
Adjacent taxa in tree: Bacteria
EC annotation - (Blast/Profile): na
PHOBIUS SP: 0
PHOBIUS TMD: 10
RefSeq annotation: hypothetical protein
Name of enzyme/protein: Predicted 2-keto-3-deoxygluconate
permease
KEGG PATHWAY - level 1: Other function
KEGG PATHWAY - level 2: na
```

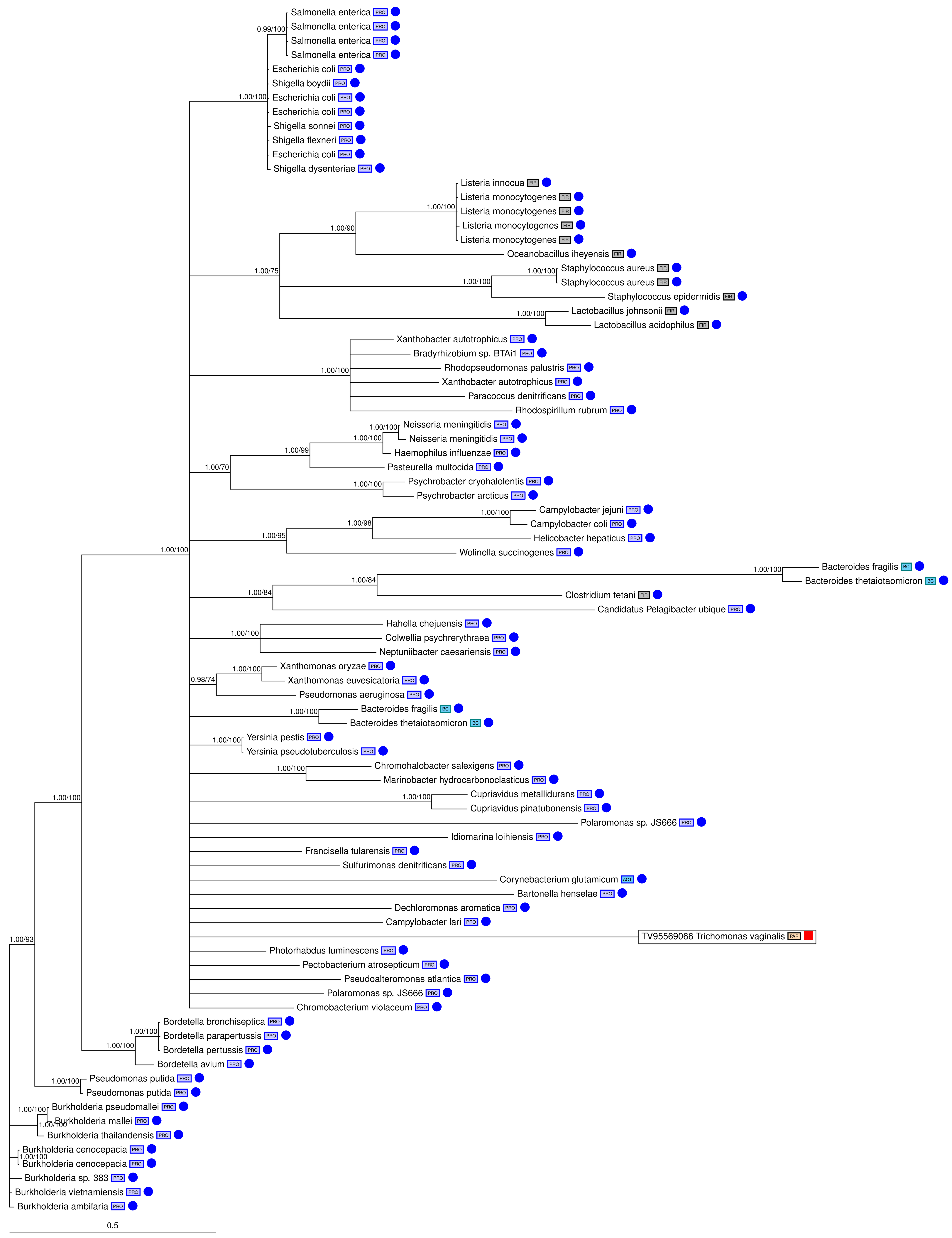

TN101

```
               Candy accession: TV96281253
              RefSeq accession: XP_001328362.1
             Uniprot accession: A2DTZ2_TRIVA
                      Comments: LGT - TV ONLY
              Species affected: TV
         Adjacent taxa in tree: Bacteria
EC annotation - (Blast/Profile): na
                    PHOBIUS SP: Y
                   PHOBIUS TMD: 0
             RefSeq annotation: hypothetical protein
        Name of enzyme/protein: Protein containing DUF369
        KEGG PATHWAY - level 1: Function unknown
        KEGG PATHWAY - level 2: na
```

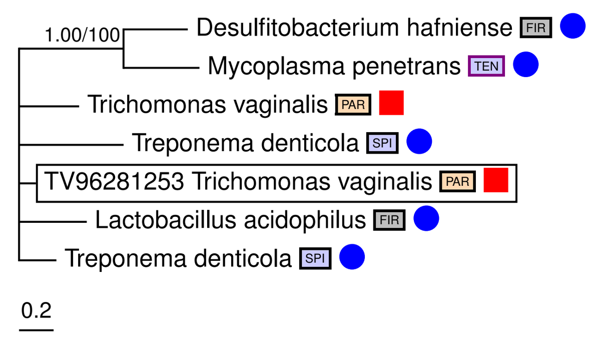

TN102

```
                Candy accession: TV97114181
               RefSeq accession: XP_001326490.1
              Uniprot accession: A2DZD8_TRIVA
                       Comments: LGT - TV ONLY
               Species affected: TV
          Adjacent taxa in tree: Prokaryotes
EC annotation - (Blast/Profile): na
                     PHOBIUS SP: 0
                    PHOBIUS TMD: 0
              RefSeq annotation: hypothetical protein
         Name of enzyme/protein: Predicted adenosine specific kinase
         KEGG PATHWAY - level 1: Other function
         KEGG PATHWAY - level 2: na
```

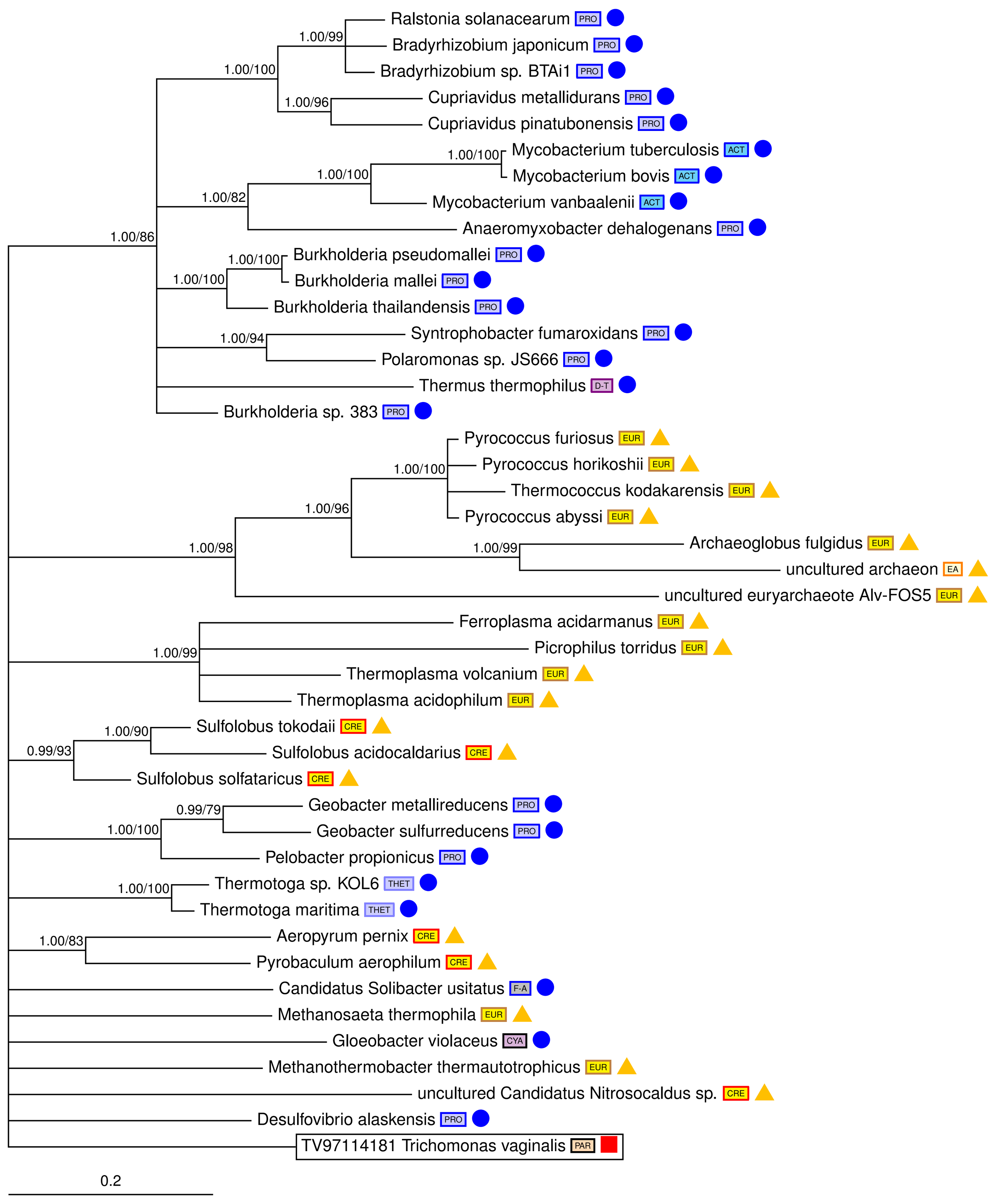

TN103

```
                Candy accession: TV97417099
               RefSeq accession: XP_001320324.1
              Uniprot accession: A2EGW7_TRIVA
                       Comments: LGT - TV ONLY
               Species affected: TV
          Adjacent taxa in tree: Bacteria
EC annotation - (Blast/Profile): EC:3.2.1.8
                     PHOBIUS SP: 0
                    PHOBIUS TMD: 0
              RefSeq annotation: hypothetical protein
         Name of enzyme/protein: endo-1,4-beta-xylanase
         KEGG PATHWAY - level 1: Reaction
         KEGG PATHWAY - level 2: Reaction
```

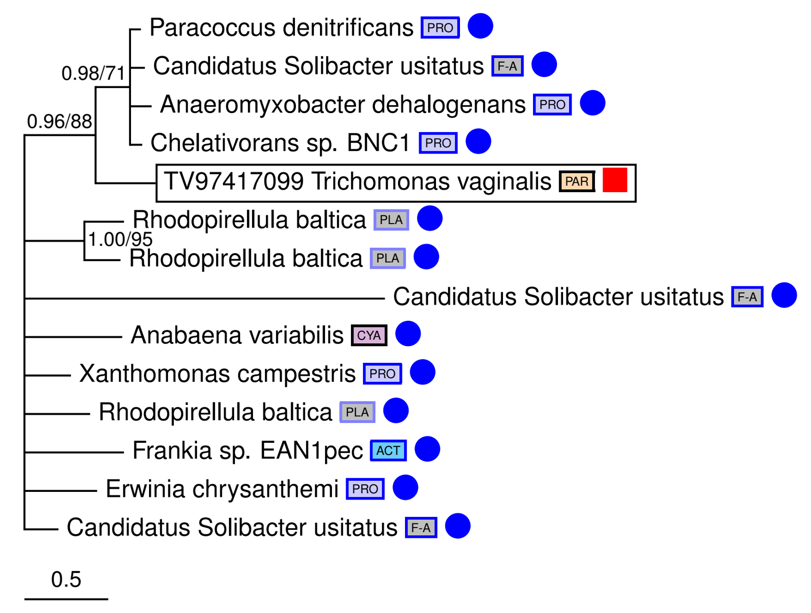

TN104

```
                Candy accession: Q4GYC9_9TRYP
               RefSeq accession: XP_001219142.1
              Uniprot accession: Q4GYC9_9TRYP
                       Comments: LGT - TB
               Species affected: TB
            Adjacent taxa in tree: Bacteria
EC annotation - (Blast/Profile): EC:2.3.1.57
                     PHOBIUS SP: 0
                    PHOBIUS TMD: 0
              RefSeq annotation: acetyltransferase
       Name of enzyme/protein: diamine N-acetyltransferase
         KEGG PATHWAY - level 1: Amino Acid Metabolism
         KEGG PATHWAY - level 2: Arginine and proline metabolism
```

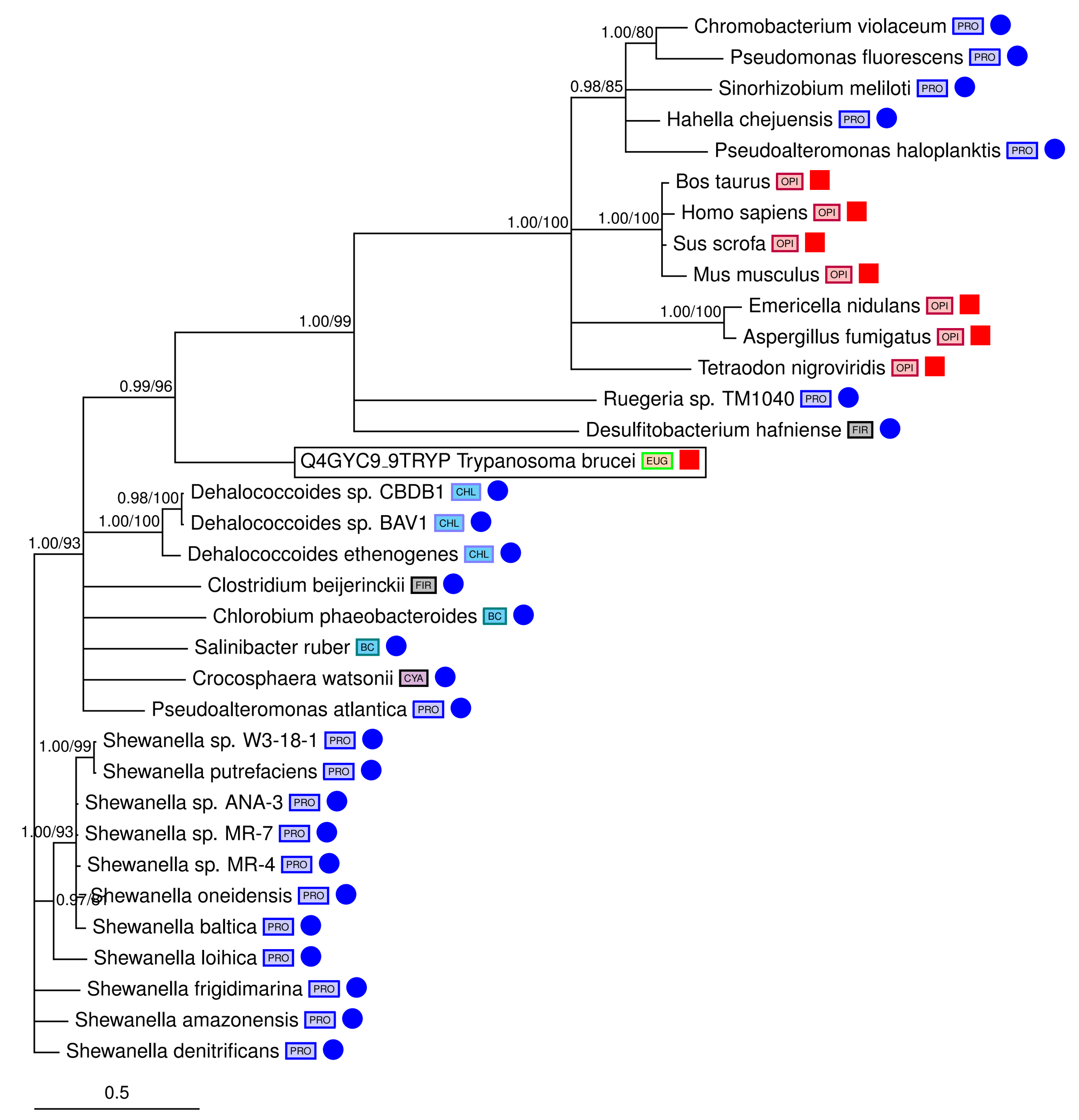

TN105

```
            Candy accession: Q4Q0I4_LEIMA
           RefSeq accession: XP_001687164.1
          Uniprot accession: Q4Q0I4_LEIMA
                   Comments: LGT - KINETOPLASTIDS ONLY
           Species affected: TB,TC,LM
     Adjacent taxa in tree: Prokaryotes
EC annotation - (Blast/Profile): na
                PHOBIUS SP: 0
               PHOBIUS TMD: 0
          RefSeq annotation: hypothetical protein
     Name of enzyme/protein: Predicted mechanosensitive channel
                             protein
      KEGG PATHWAY - level 1: Other function
      KEGG PATHWAY - level 2: na
```

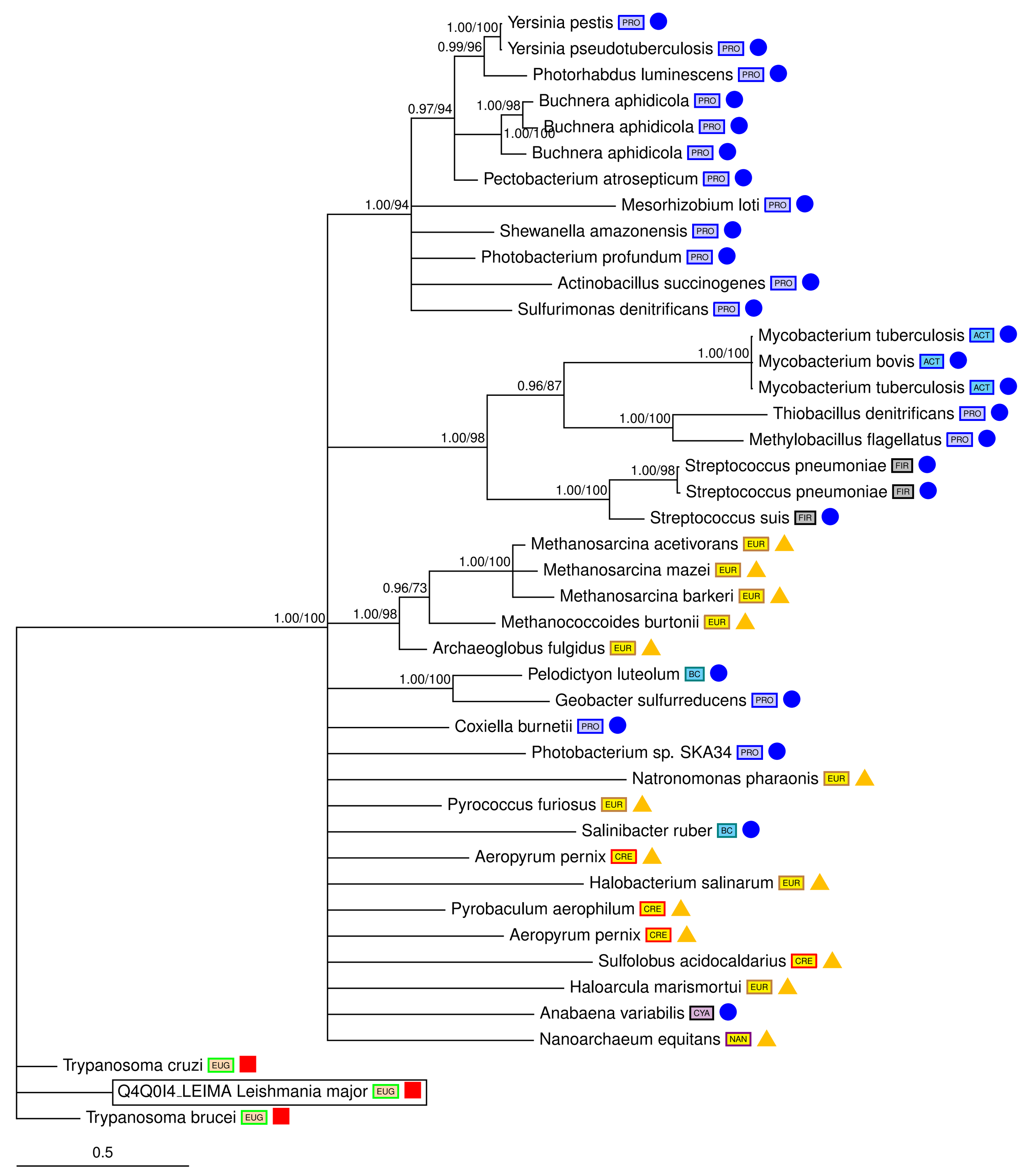

TN106

```
              Candy accession: Q4Q2V9_LEIMA
             RefSeq accession: XP_001686339.1
            Uniprot accession: Q4Q2V9_LEIMA
                     Comments: LGT - KINETOPLASTIDS ONLY
             Species affected: LM,TB,TC
        Adjacent taxa in tree: Firmicutes
EC annotation - (Blast/Profile): EC:2.6.1.42
                   PHOBIUS SP: 0
                  PHOBIUS TMD: 0
            RefSeq annotation: hypothetical protein
      Name of enzyme/protein: branched-chain amino acid
                               aminotransferase
       KEGG PATHWAY - level 1: Amino Acid Metabolism, Metabolism of
                               Cofactors and Vitamins
       KEGG PATHWAY - level 2: Valine, leucine and isoleucine
                               degradation and biosynthesis,
                               Pantothenate and CoA biosynthesis
```

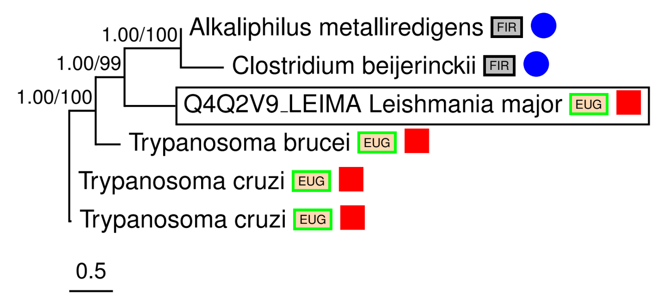

TN107

```
                Candy accession: Q4Q8S0_LEIMA
               RefSeq accession: XP_001684278.1
              Uniprot accession: Q4Q8S0_LEIMA
                       Comments: LGT - LM TWO NODES
               Species affected: LM
           Adjacent taxa in tree: Proteobacteria
EC annotation - (Blast/Profile): EC:2.7.2.11
                     PHOBIUS SP: 0
                    PHOBIUS TMD: 0
              RefSeq annotation: glutamate 5-kinase
        Name of enzyme/protein: Glutamate 5-kinase
        KEGG PATHWAY - level 1: Amino Acid Metabolism
        KEGG PATHWAY - level 2: Arginine and proline metabolism
```

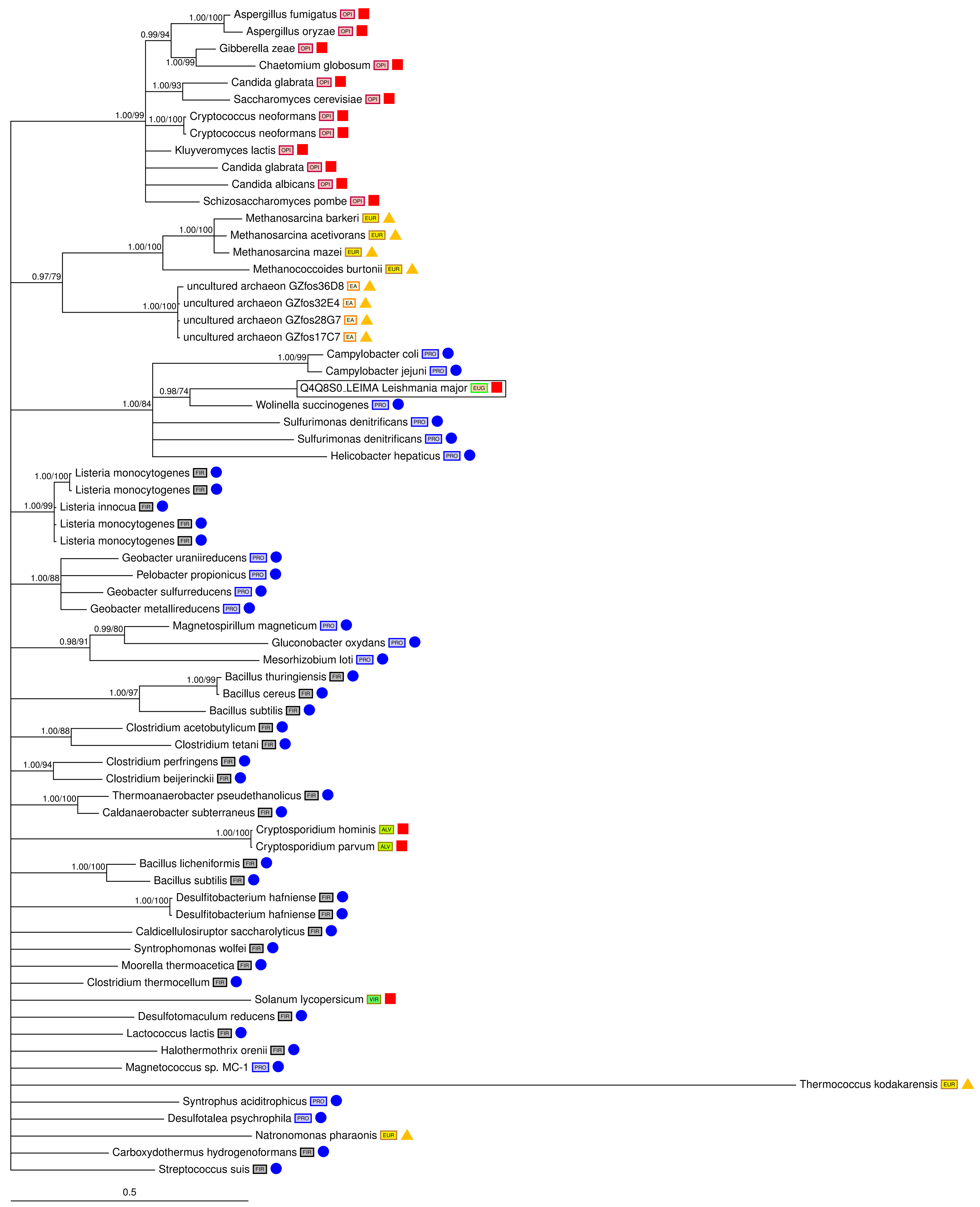

TN108

Candy accession: Q4Q273_LEIMA
RefSeq accession: XP_001686575.1
Uniprot accession: Q4Q273_LEIMA
Comments: LGT - KINETOPLASTIDS TWO NODES
Species affected: LM, TC
Adjacent taxa in tree: Proteobacteria
EC annotation - (Blast/Profile): EC:2.7.1.16
PHOBIUS SP: 0
PHOBIUS TMD: 0
RefSeq annotation: L-ribulokinase
Name of enzyme/protein: L-ribulokinase
KEGG PATHWAY - level 1: Carbohydrate Metabolism
KEGG PATHWAY - level 2: Pentose and glucuronate interconversions

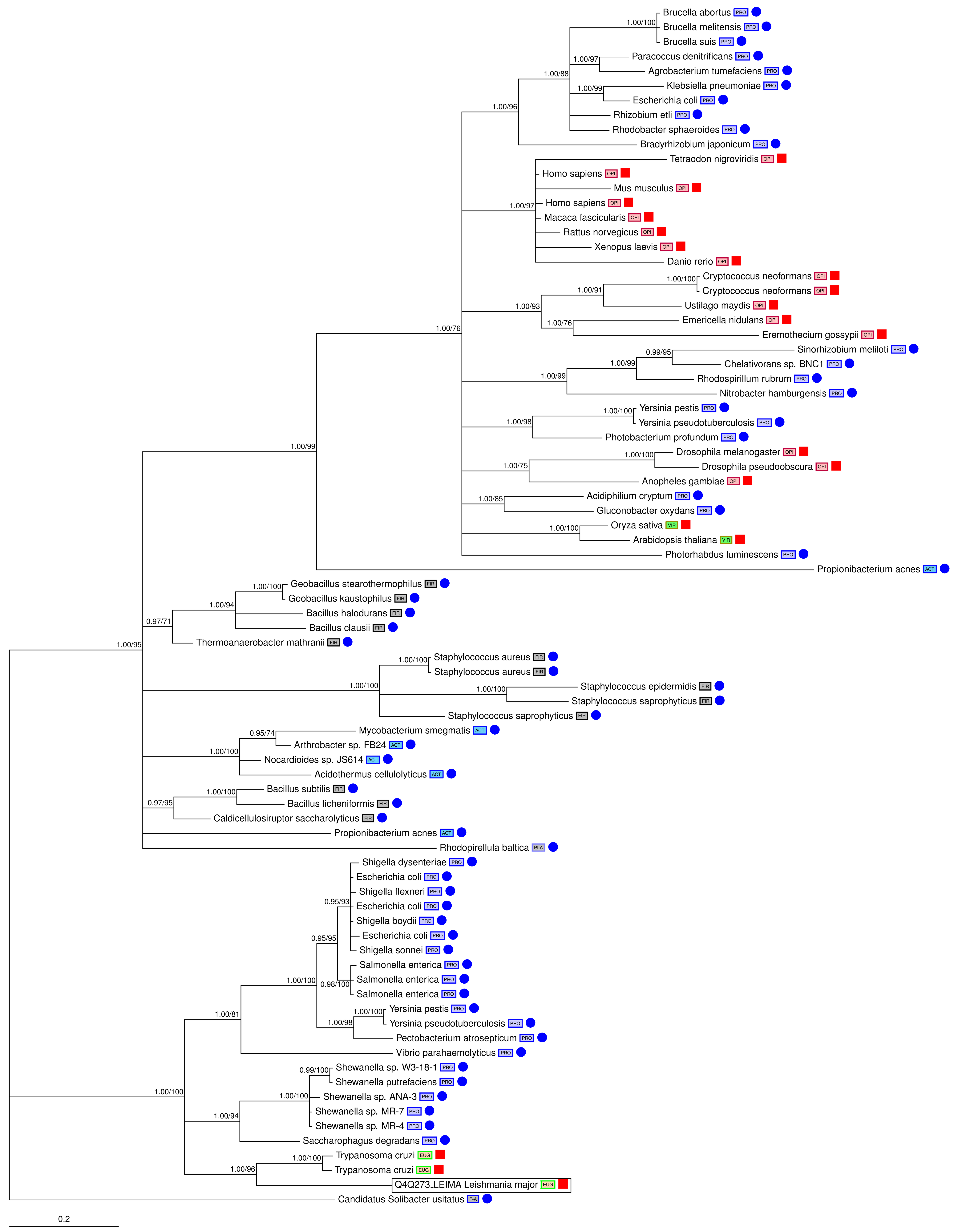

TN109

```
                Candy accession: Q4Q762_LEIMA
               RefSeq accession: XP_001684836.1
              Uniprot accession: Q4Q762_LEIMA
                       Comments: LGT - KINETOPLASTIDS ONLY
               Species affected: TB,TC,LM
         Adjacent taxa in tree: Prokaryotes
EC annotation - (Blast/Profile): EC:2.1.1.173
                     PHOBIUS SP: 0
                    PHOBIUS TMD: 0
              RefSeq annotation: hypothetical protein
          Name of enzyme/protein: 23S rRNA
                                 (guanine2445-N2)-methyltransferase
        KEGG PATHWAY - level 1: Reaction
        KEGG PATHWAY - level 2: Reaction
```

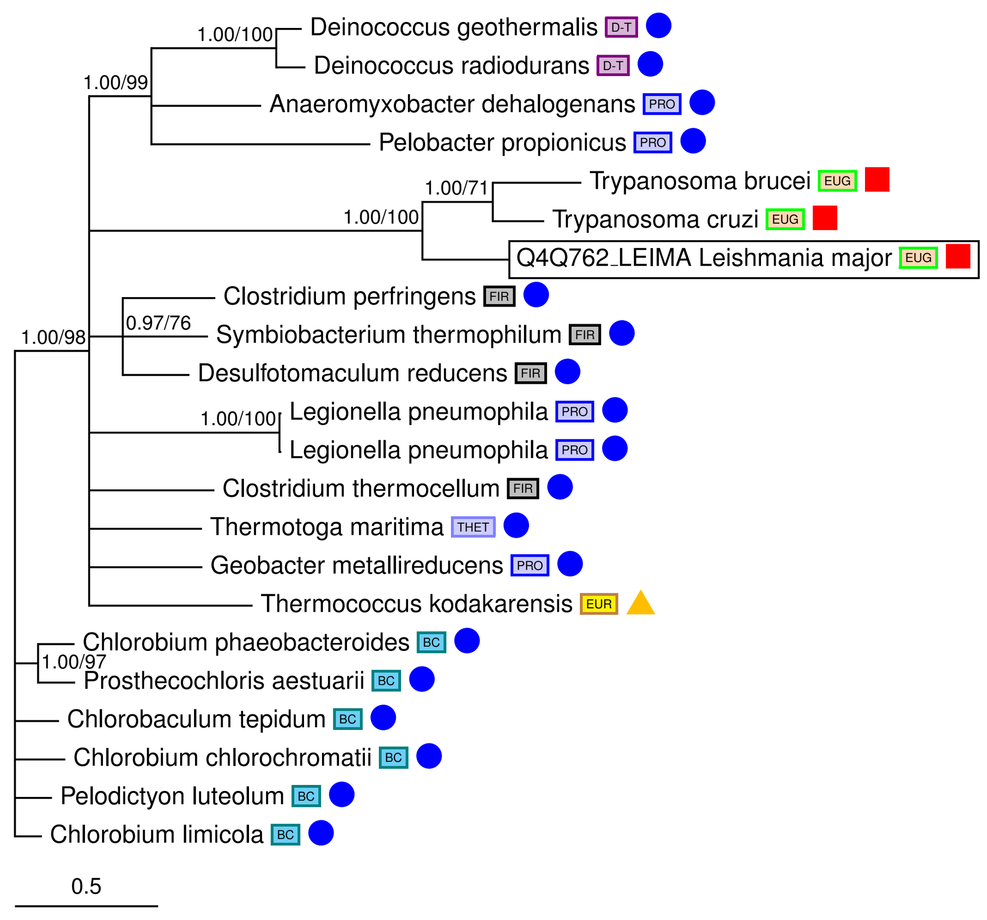

TN110

Candy accession: Q4QBE8_LEIMA
RefSeq accession: XP_001683350.1
Uniprot accession: Q4QBE8_LEIMA
Comments: LGT - LM TWO NODES - 3 DOMAIN TREE
Species affected: LM
Adjacent taxa in tree: Firmicutes - Clostridia
EC annotation - (Blast/Profile): EC:6.3.4.5
PHOBIUS SP: Y
PHOBIUS TMD: 0
RefSeq annotation: argininosuccinate synthase
Name of enzyme/protein: Argininosuccinate synthase
KEGG PATHWAY - level 1: Amino Acid Metabolism
KEGG PATHWAY - level 2: Alanine and aspartate metabolism,
Arginine and proline metabolism

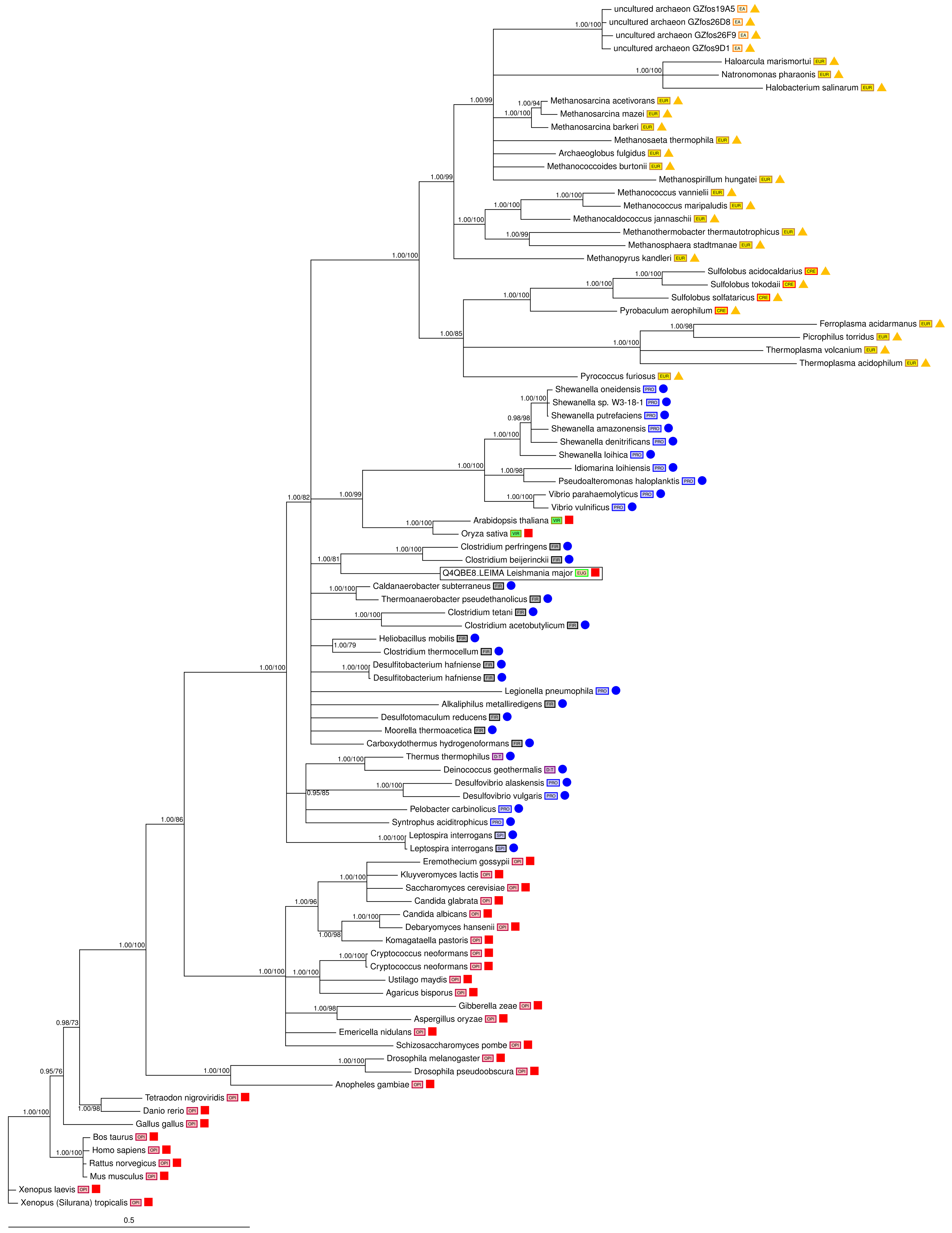

TN111

```
                 Candy accession: Q4QIW8_LEIMA
                RefSeq accession: XP_001680880.1
               Uniprot accession: Q4QIW8_LEIMA
                        Comments: LGT - LM ONLY
                Species affected: LM
           Adjacent taxa in tree: Prokaryotes
EC annotation - (Blast/Profile): EC:3.1.31.1
                      PHOBIUS SP: 0
                     PHOBIUS TMD: 0
               RefSeq annotation: hypothetical protein
         Name of enzyme/protein: micrococcal nuclease
         KEGG PATHWAY - level 1: Reaction
         KEGG PATHWAY - level 2: Reaction
```

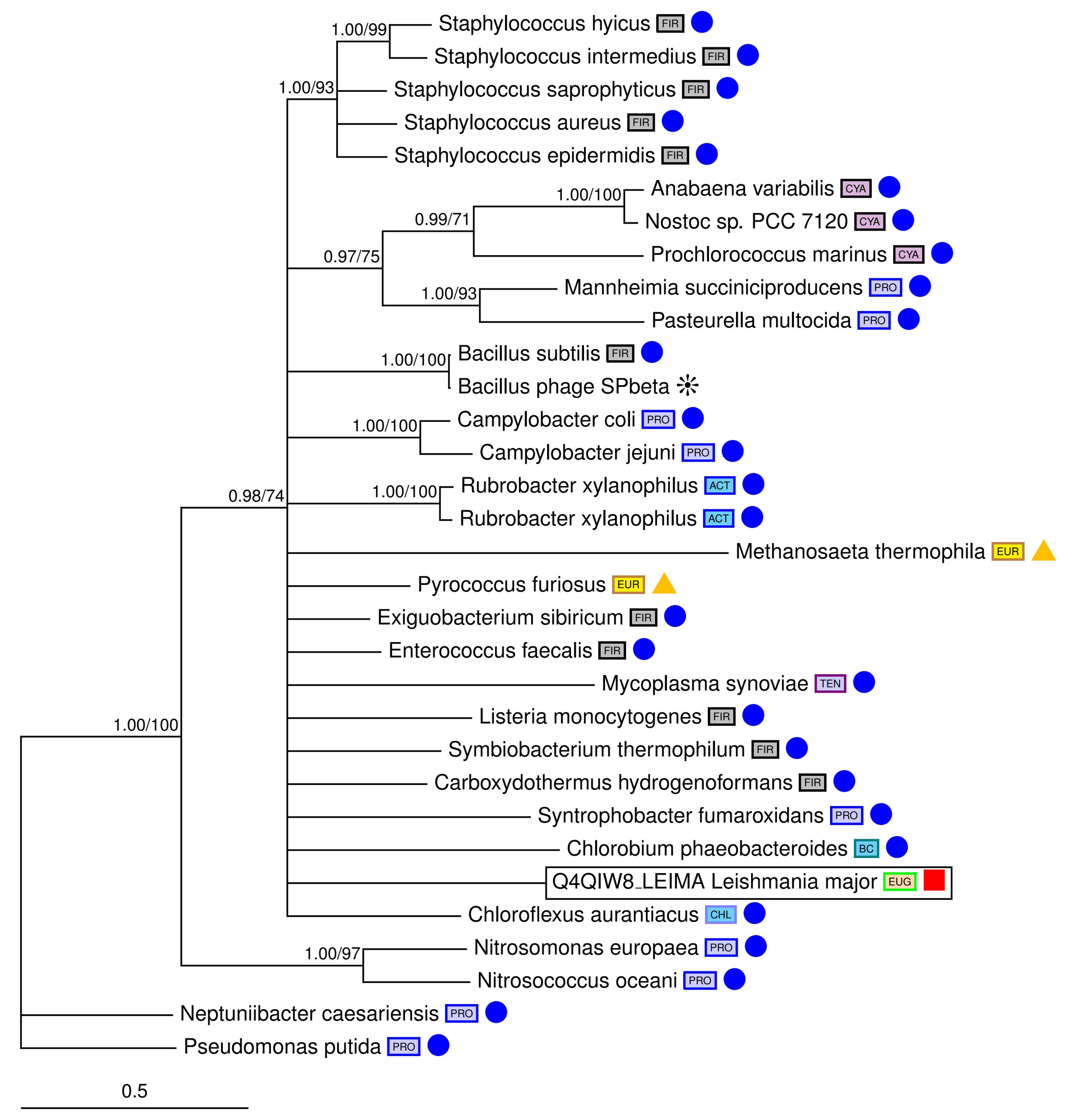

TN112

```
               Candy accession: Q5CRD4_CRYPV
              RefSeq accession: XP_626252.1
             Uniprot accession: Q5CRD4_CRYPV
                      Comments: LGT - CP ONLY
              Species affected: CP
         Adjacent taxa in tree: Bacteria
EC annotation - (Blast/Profile): EC:2.4.1.-
                    PHOBIUS SP: 0
                   PHOBIUS TMD: 0
             RefSeq annotation: secreted lipopolysaccharide sugar
                                transferase like family 8
                                glycosyltransferase
        Name of enzyme/protein: lipopolysaccharide
                                1,2-glycosyltransferase
        KEGG PATHWAY - level 1: Glycan Biosynthesis and Metabolism
        KEGG PATHWAY - level 2: Lipopolysaccharide biosynthesis
```

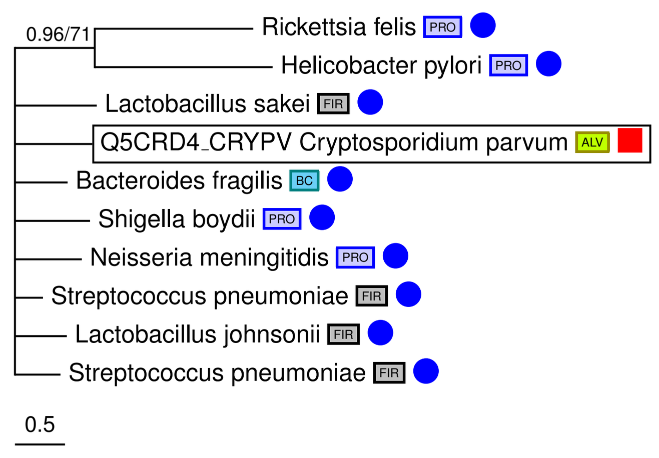

TN113

```
                Candy accession: Q5CV50_CRYPV
               RefSeq accession: XP_627419.1
              Uniprot accession: Q5CV50_CRYPV
                       Comments: LGT - CP AND CH TWO NODES
               Species affected: CP
          Adjacent taxa in tree: Tenericutes - Mycoplasma
EC annotation - (Blast/Profile): EC:3.2.2.21
                    PHOBIUS SP: 0
                   PHOBIUS TMD: 0
             RefSeq annotation: DNA-3-methyladenine glycosidase
       Name of enzyme/protein: DNA-3-methyladenine glycosidase II
         KEGG PATHWAY - level 1: Reaction
         KEGG PATHWAY - level 2: Reaction
```

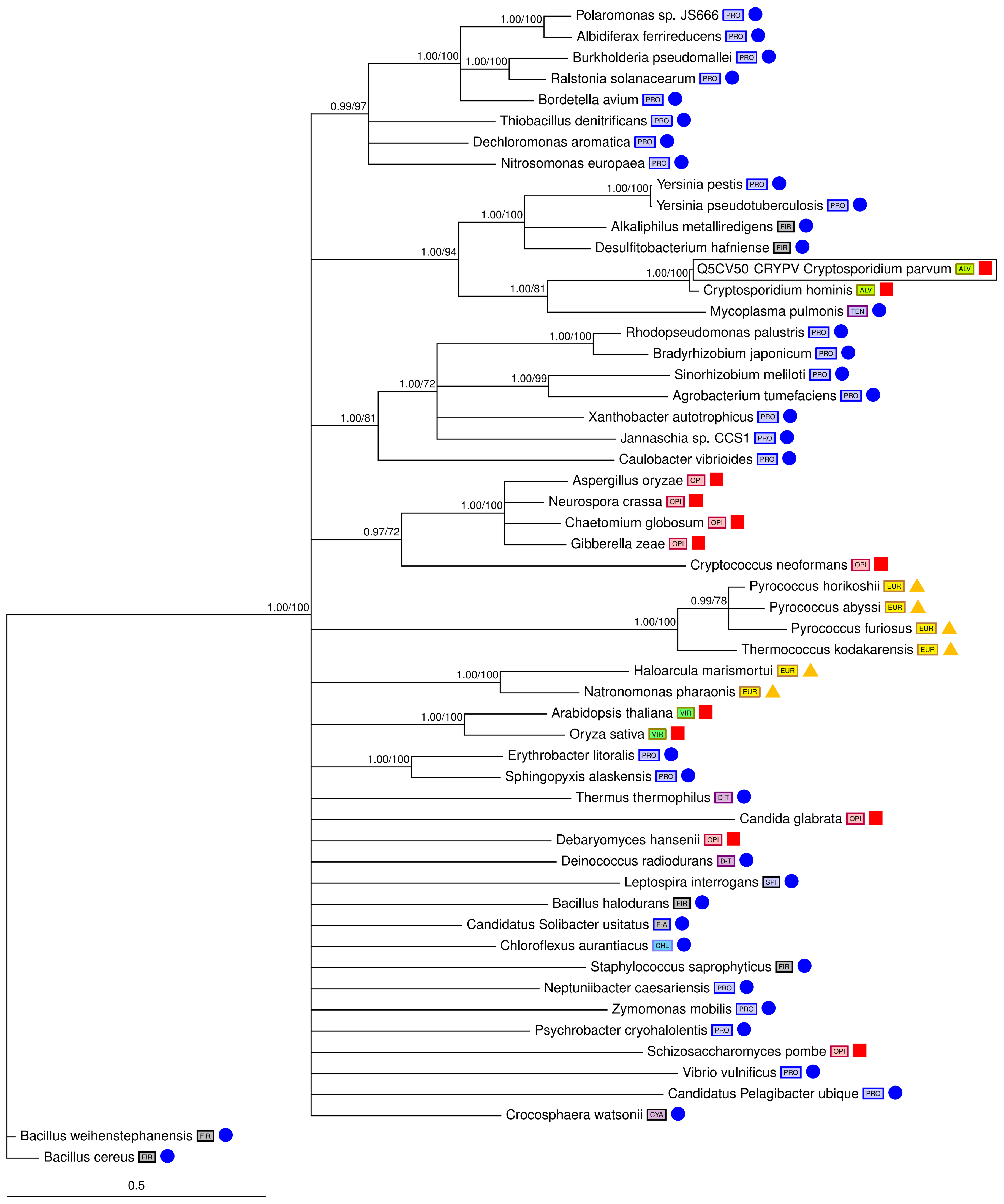

TN115

```
                Candy accession: Q7R5Z7_GIALA
               RefSeq accession: XP_001706848.1
              Uniprot accession: A8BII9_GIALA
                       Comments: LGT - GI ONLY
               Species affected: GI
          Adjacent taxa in tree: Bacteria
EC annotation - (Blast/Profile): EC:3.1.3.15
                     PHOBIUS SP: 0
                    PHOBIUS TMD: 0
              RefSeq annotation: Histidinol-phosphatase, putative
         Name of enzyme/protein: Histidinol-phosphatase
         KEGG PATHWAY - level 1: Amino Acid Metabolism
         KEGG PATHWAY - level 2: Histidine metabolism
```

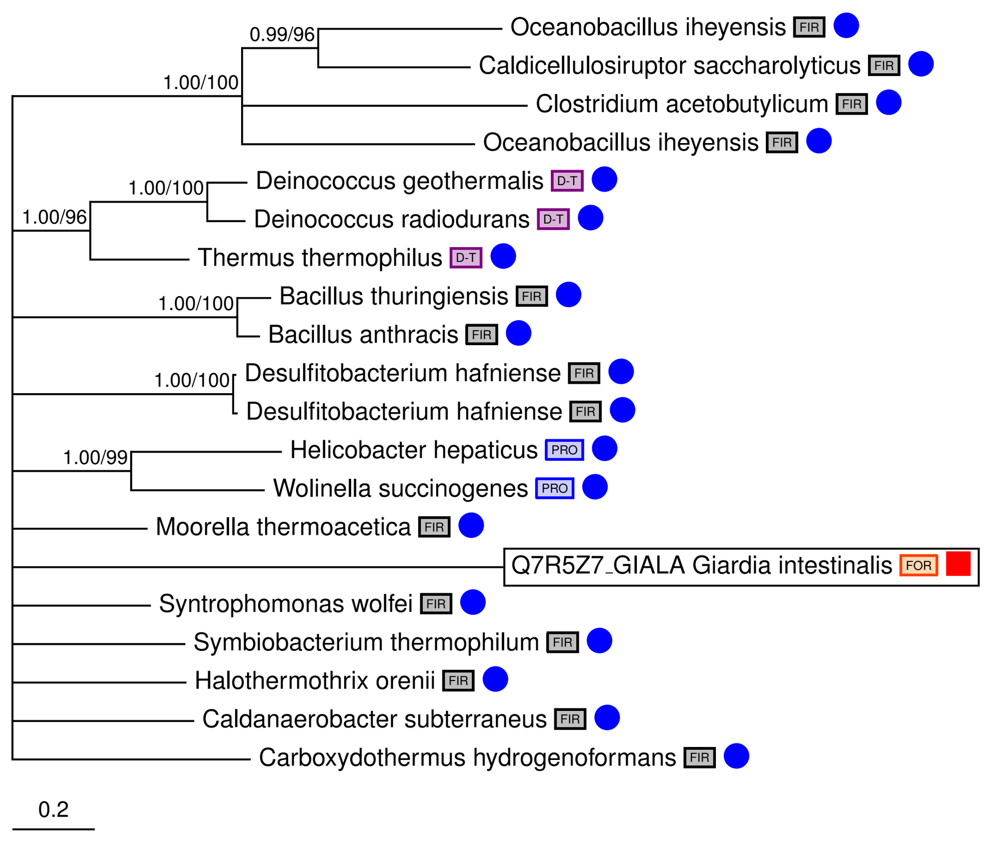

TN116

```
                Candy accession: Q7R406_GIALA
               RefSeq accession: XP_001709106.1
              Uniprot accession: A8B707_GIALA
                       Comments: LGT - GI + ONE CANDIDA AND PHAGE
               Species affected: GI
          Adjacent taxa in tree: Bacteroidetes/Chlorobi - Chlorobium
EC annotation - (Blast/Profile): EC:3.6.1.-
                     PHOBIUS SP: 0
                    PHOBIUS TMD: 0
              RefSeq annotation: Helicase-related protein
         Name of enzyme/protein:  DNA/RNA helicase
         KEGG PATHWAY - level 1: Reaction
         KEGG PATHWAY - level 2: Reaction
```

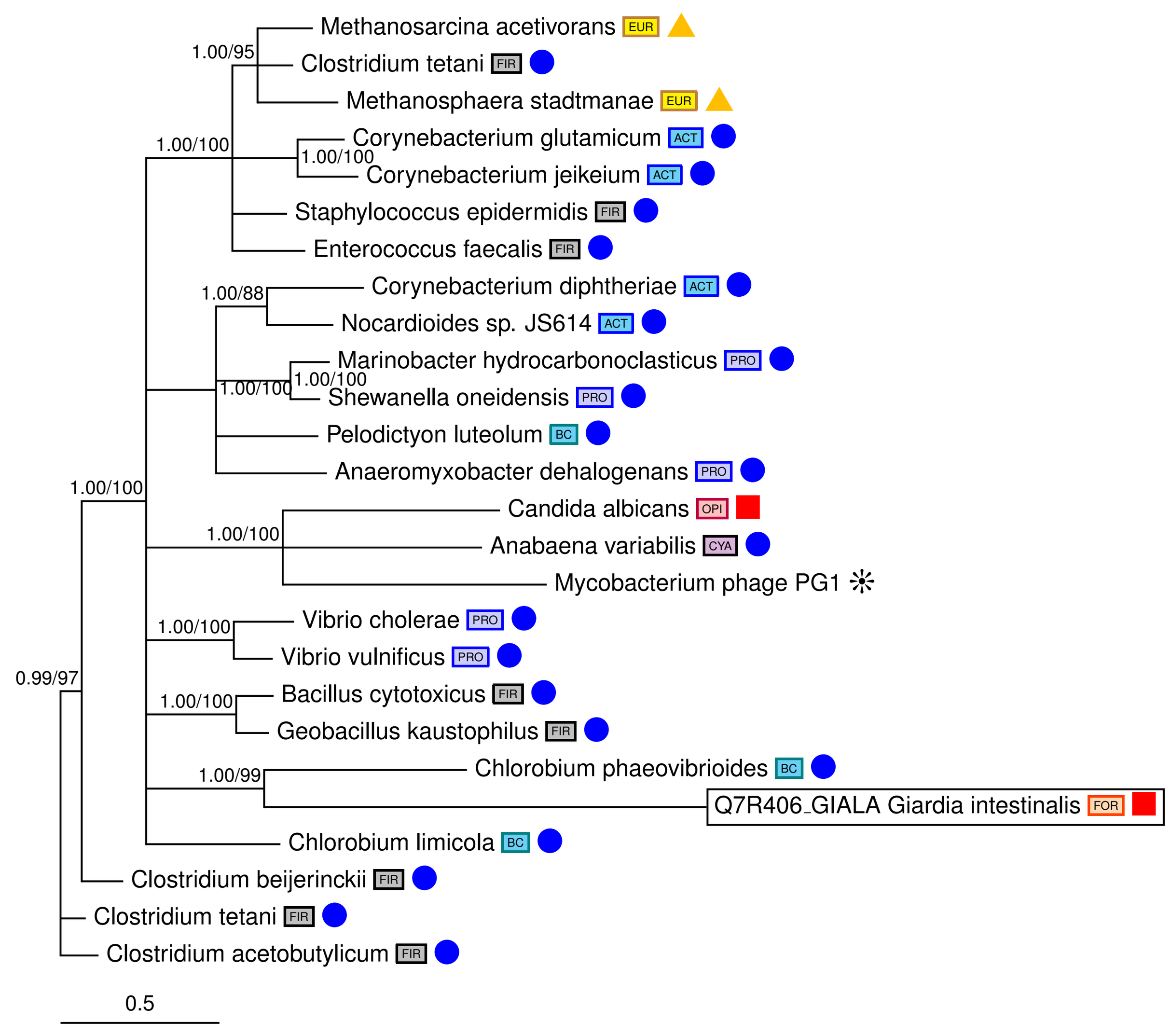

TN117

```
             Candy accession: Q9BHF7_LEIMA
            RefSeq accession: XP_001685041.1
           Uniprot accession: Q9BHF7_LEIMA
                    Comments: LGT - KINETOPLASTIDS ONLY
            Species affected: LM,TB,TC
       Adjacent taxa in tree: Prokaryotes
EC annotation - (Blast/Profile): EC:2.7.1.36
                  PHOBIUS SP: 0
                 PHOBIUS TMD: 0
           RefSeq annotation: mevalonate kinase
     Name of enzyme/protein: mevalonate kinase
     KEGG PATHWAY - level 1: Metabolism of Terpenoids and Polyketides
     KEGG PATHWAY - level 2: Terpenoid backbone biosynthesis
```

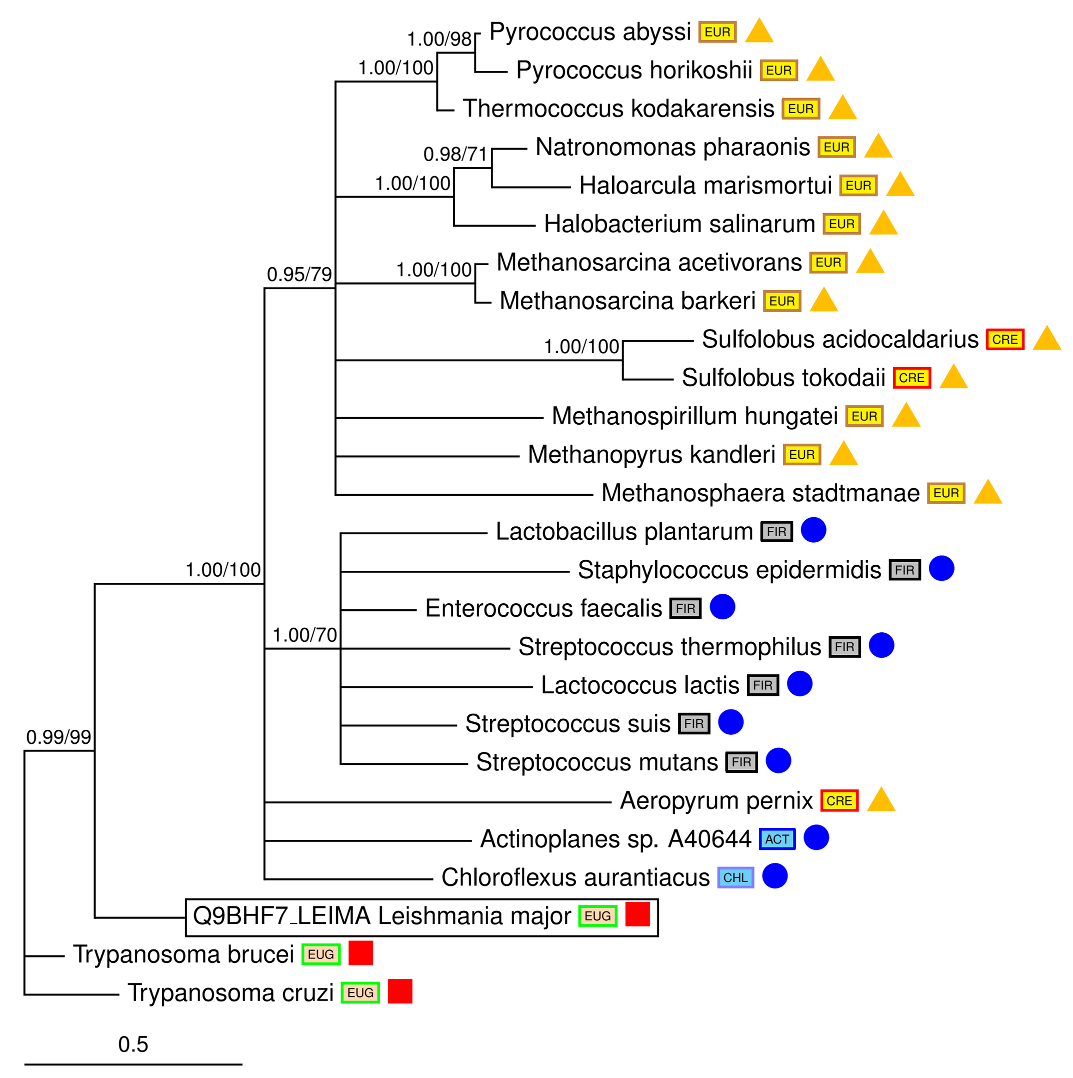

TN118

```
           Candy accession: Q38A50_9TRYP
          RefSeq accession: XP_823148.1
         Uniprot accession: Q38A50_9TRYP
                  Comments: LGT - KINETOPLASTIDS ONLY
          Species affected: LM,TB,TC
     Adjacent taxa in tree: Chlamydiae/Verrucomicrobia -
                            Protochlamydia
EC annotation - (Blast/Profile): na
                PHOBIUS SP: 0
               PHOBIUS TMD: 0
         RefSeq annotation: hypothetical protein
  Name of enzyme/protein: Predicted CesT Tir chaperone protein
   KEGG PATHWAY - level 1: Other function
   KEGG PATHWAY - level 2: na
```

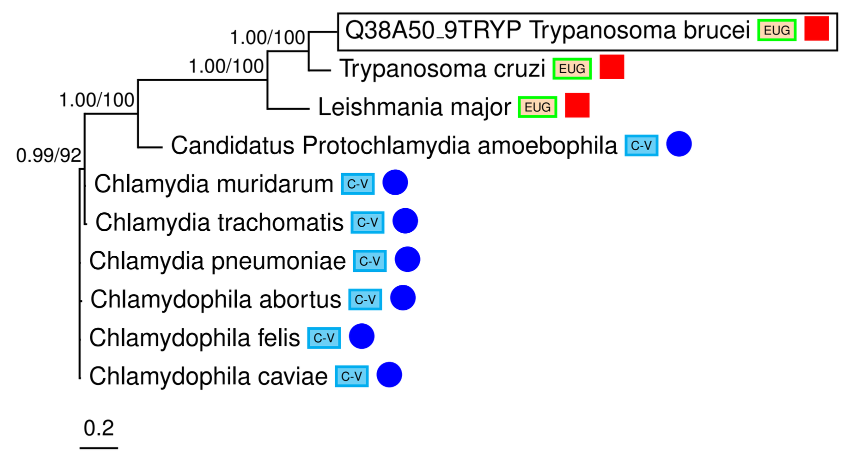

TN119

```
                Candy accession: Q51A23_ENTHI
               RefSeq accession: XP_655080.2
              Uniprot accession: C4LXS0_ENTHI
                       Comments: LGT - EH ONLY
               Species affected: EH
          Adjacent taxa in tree: Archaea
EC annotation - (Blast/Profile): na
                     PHOBIUS SP: 0
                    PHOBIUS TMD: 0
              RefSeq annotation: hypothetical protein
         Name of enzyme/protein: Protein containing DUF82
         KEGG PATHWAY - level 1: Function unknown
         KEGG PATHWAY - level 2: na
```

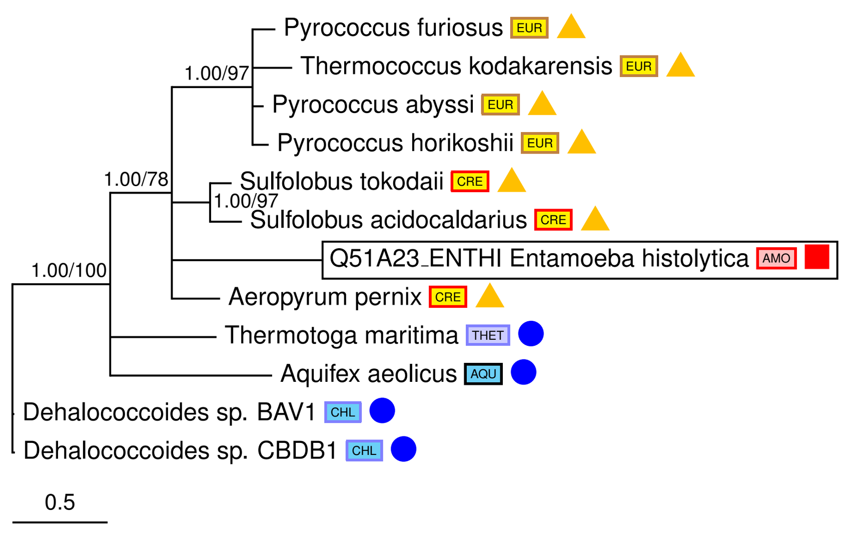

TN120

```
                Candy accession: Q51C60_ENTHI
               RefSeq accession: XP_655826.2
              Uniprot accession: C4M220_ENTHI
                       Comments: LGT - EH - PLUS ANIMAL FUNGI CLADE
               Species affected: EH
          Adjacent taxa in tree: Prokaryotes
EC annotation - (Blast/Profile): EC:4.3.1.1
                     PHOBIUS SP: 0
                    PHOBIUS TMD: 0
              RefSeq annotation: aspartate ammonia-lyase
         Name of enzyme/protein: aspartate ammonia-lyase
         KEGG PATHWAY - level 1: Amino Acid Metabolism, Energy Metabolism
         KEGG PATHWAY - level 2: Alanine, aspartate and glutamate
                                 metabolism, Nitrogen metabolism
```

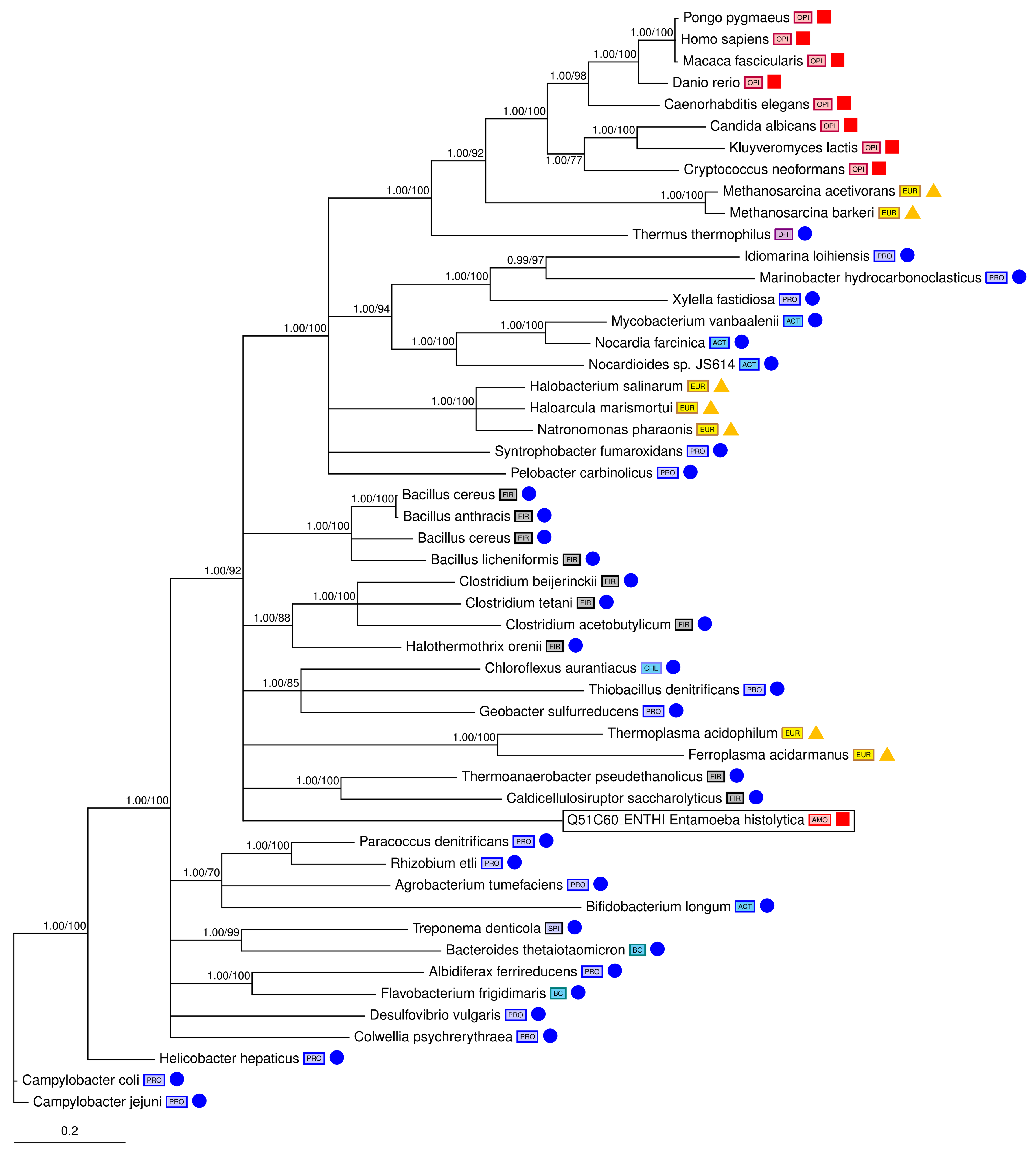

TN121

```
                Candy accession: Q51C98_ENTHI
               RefSeq accession: XP_655888.1
              Uniprot accession: Q8MU40_ENTHI
                       Comments: LGT - EH ONLY
               Species affected: EH
          Adjacent taxa in tree: Proteobacteria
EC annotation - (Blast/Profile): EC:1.7.1.4
                     PHOBIUS SP: 0
                    PHOBIUS TMD: 0
              RefSeq annotation: Fe-S cluster assembly protein NifU
         Name of enzyme/protein: nitrite reductase [NAD(P)H]
         KEGG PATHWAY - level 1: Energy Metabolism
         KEGG PATHWAY - level 2: Nitrogen metabolism
```

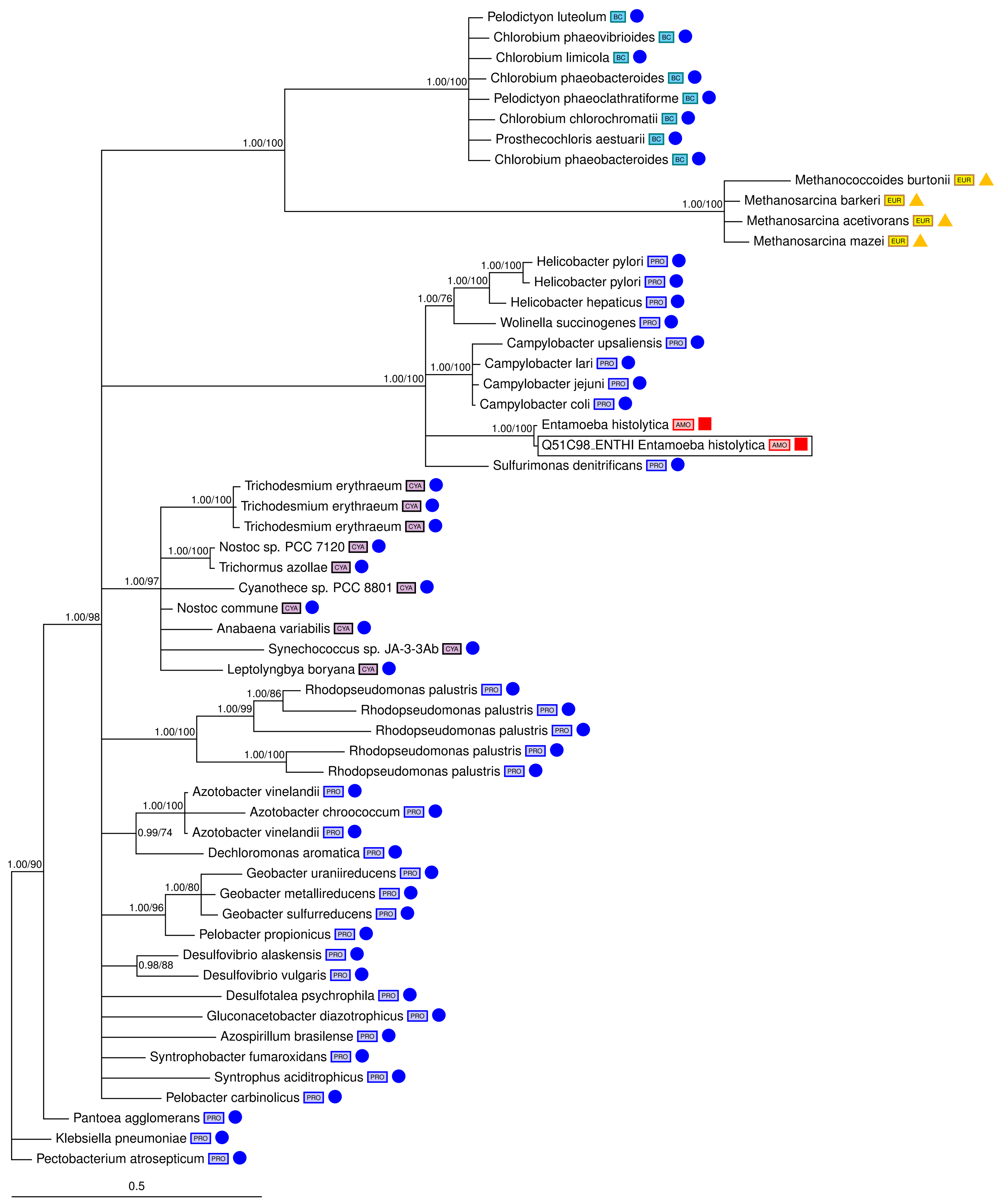

TN122

```
                Candy accession: Q51CJ1_ENTHI
               RefSeq accession: XP_655905.1
              Uniprot accession: C4LZG3_ENTHI
                       Comments: LGT - EH ONLY
               Species affected: EH
         Adjacent taxa in tree: Bacteria
EC annotation - (Blast/Profile): EC:2.7.1.144
                     PHOBIUS SP: 0
                    PHOBIUS TMD: 0
             RefSeq annotation: tagatose-6-phosphate kinase
         Name of enzyme/protein: tagatose-6-phosphate kinase
         KEGG PATHWAY - level 1: Carbohydrate Metabolism
         KEGG PATHWAY - level 2: Galactose metabolism
```

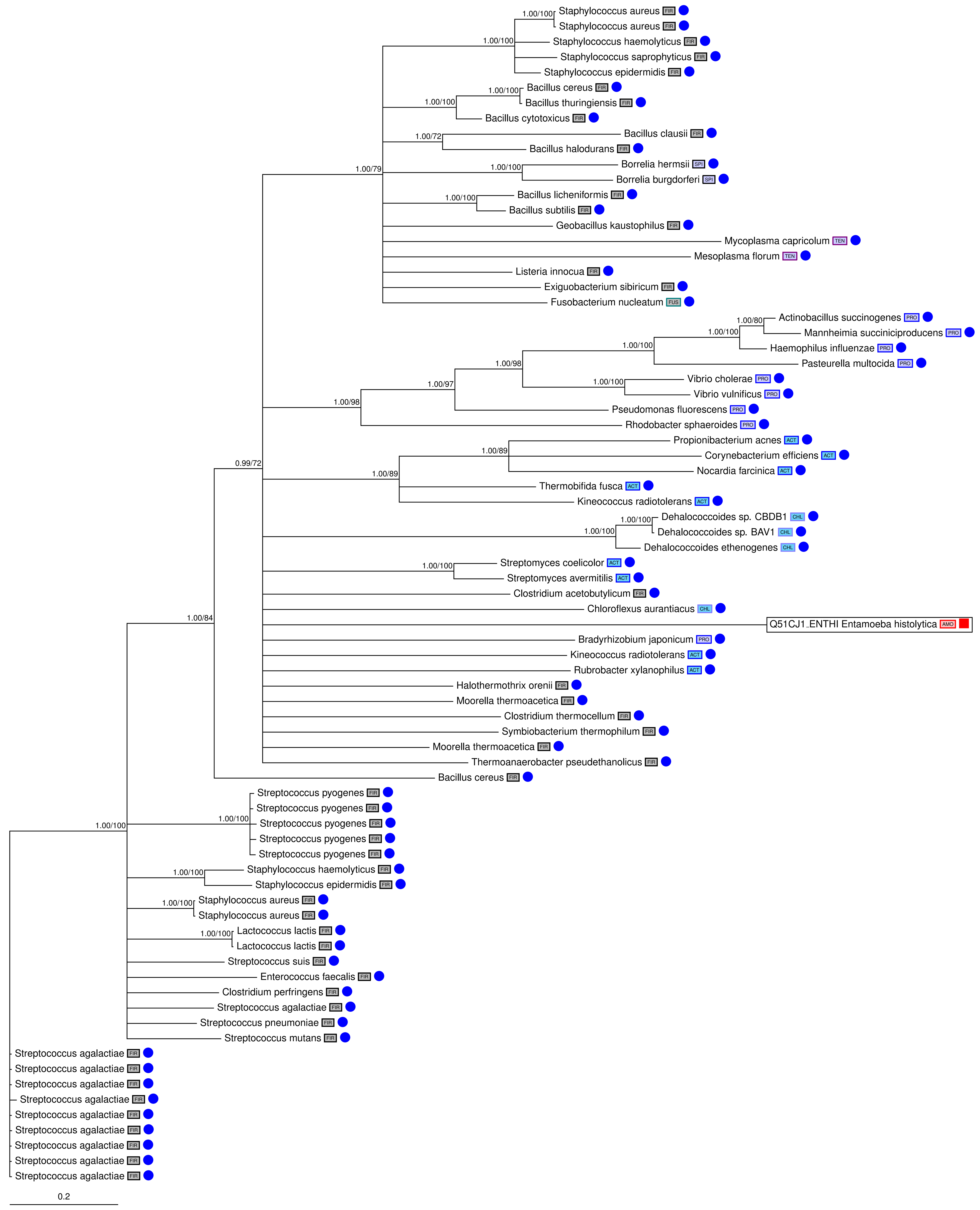

TN123

```
                Candy accession: Q51FH0_ENTHI
               RefSeq accession: XP_656903.1
              Uniprot accession: C4LWE1_ENTHI
                       Comments: LGT - EH ONLY
               Species affected: EH
          Adjacent taxa in tree: Bacteria
EC annotation - (Blast/Profile): EC:3.2.1.8
                     PHOBIUS SP: Y
                    PHOBIUS TMD: 0
              RefSeq annotation: endo-1,4-beta-xylanase
       Name of enzyme/protein: endo-1,4-beta-xylanase
         KEGG PATHWAY - level 1: Reaction
         KEGG PATHWAY - level 2: Reaction
```

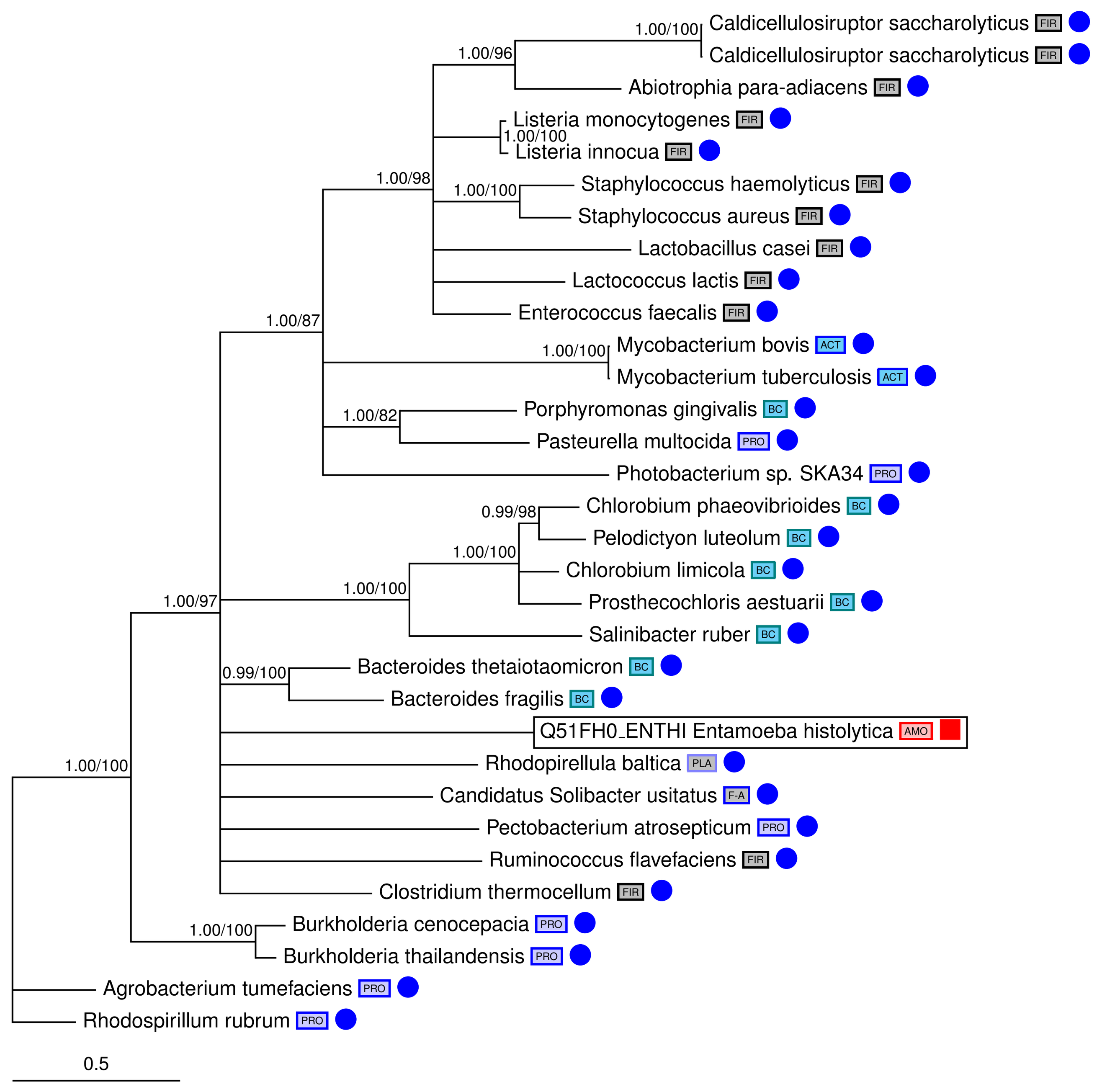

TN124

```
                Candy accession: Q54B13_DICDI
               RefSeq accession: XP_628903.1
              Uniprot accession: Q54B13_DICDI
                       Comments: LGT - DD ONLY
               Species affected: DD
         Adjacent taxa in tree: Bacteroidetes/Chlorobi - Flavobacterium
EC annotation - (Blast/Profile): na
                    PHOBIUS SP: 0
                   PHOBIUS TMD: 0
              RefSeq annotation: hypothetical protein DDB_G0293956
        Name of enzyme/protein: Predicted tRNA-binding protein
        KEGG PATHWAY - level 1: Other function - Genetic Information
                                Processing
        KEGG PATHWAY - level 2: na
```

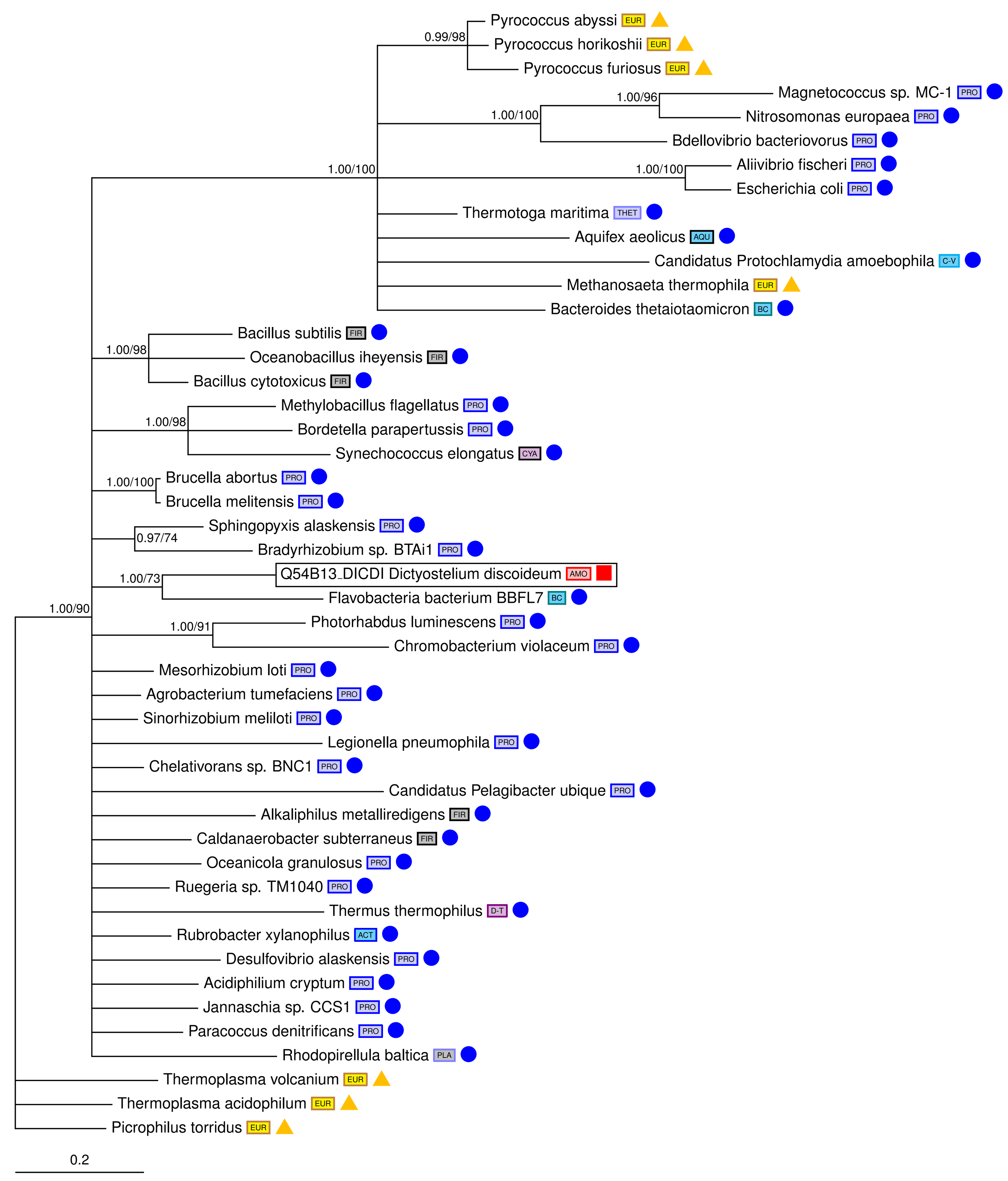

TN125

```
               Candy accession: Q54BY6_DICDI
              RefSeq accession: XP_629205.1
             Uniprot accession: Q54BY6_DICDI
                      Comments: LGT - DD ONLY
              Species affected: DD
          Adjacent taxa in tree: Proteobacteria
EC annotation - (Blast/Profile): EC:3.4.21.26
                     PHOBIUS SP: 0
                    PHOBIUS TMD: 0
             RefSeq annotation: hypothetical protein DDB_G0293330
         Name of enzyme/protein: Hypothetical protein
         KEGG PATHWAY - level 1: Reaction
         KEGG PATHWAY - level 2: Reaction
```

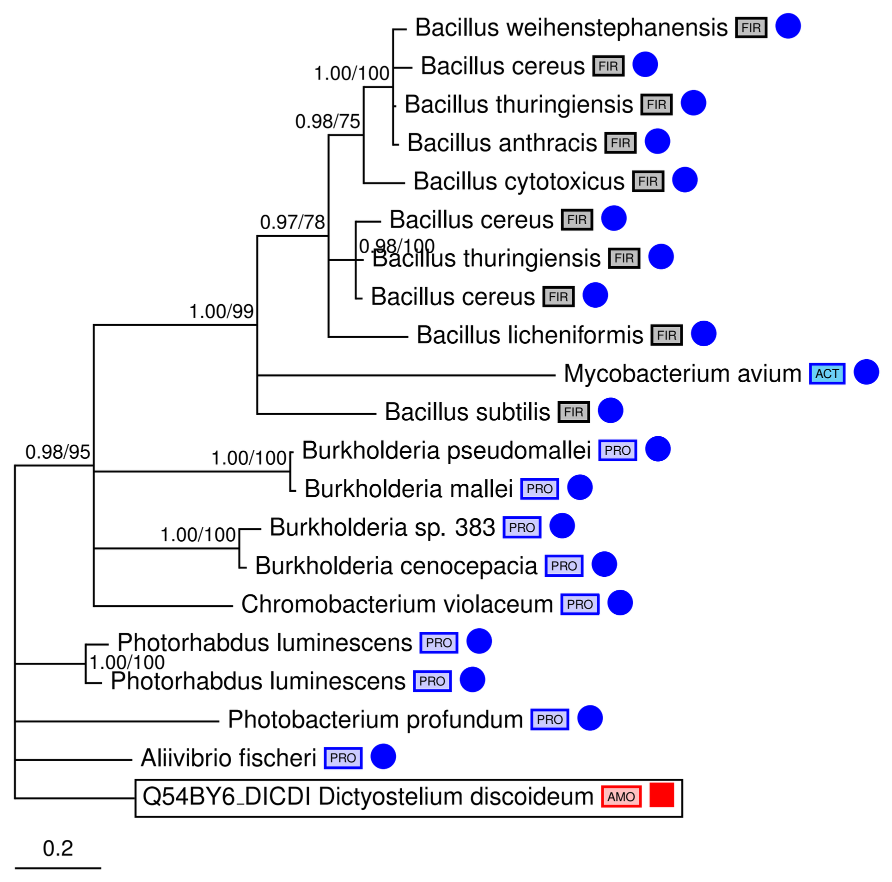

TN126

```
              Candy accession: Q54FI4_DICDI
             RefSeq accession: XP_635556.1
            Uniprot accession: Q54FI4_DICDI
                     Comments: LGT - DD ONLY
             Species affected: DD
       Adjacent taxa in tree: Bacteria
EC annotation - (Blast/Profile): EC:3.8.1.1
                   PHOBIUS SP: 0
                  PHOBIUS TMD: 0
            RefSeq annotation: hypothetical protein DDB_G0290825
       Name of enzyme/protein: alkylhalidase
      KEGG PATHWAY - level 1: Reaction
      KEGG PATHWAY - level 2: Reaction
```

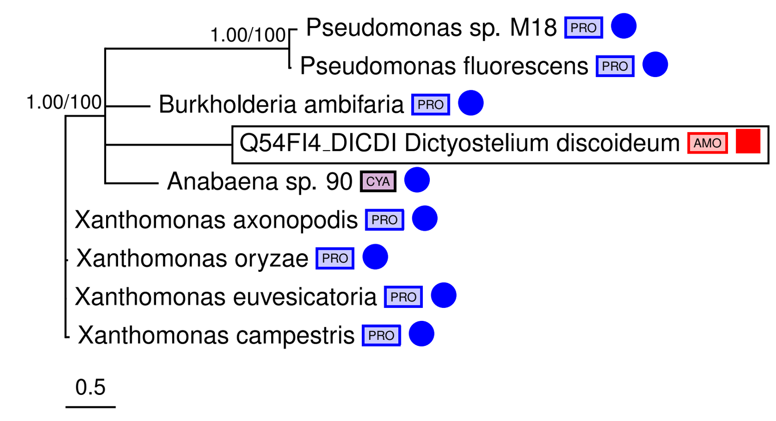

TN127

```
               Candy accession: Q54LW1_DICDI
              RefSeq accession: XP_637719.1
             Uniprot accession: Q54LW1_DICDI
                      Comments: LGT - DD ONLY + 2 BACT + ONE ARCHAEA
              Species affected: DD
          Adjacent taxa in tree: Prokaryotes
EC annotation - (Blast/Profile): na
                    PHOBIUS SP: 0
                   PHOBIUS TMD: 0
             RefSeq annotation: hypothetical protein DDB_G0286383
        Name of enzyme/protein: Protein containing GAF domain
        KEGG PATHWAY - level 1: Other function - Signal Transduction
        KEGG PATHWAY - level 2: na
```

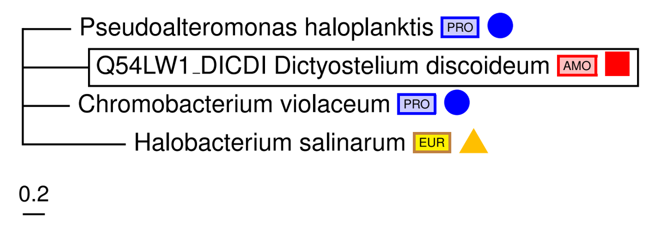

TN128

```
                  Candy accession: Q54TU4_DICDI
                 RefSeq accession: XP_640717.1
                Uniprot accession: Q54TU4_DICDI
                         Comments: LGT - DD ONLY
                 Species affected: DD
            Adjacent taxa in tree: Prokaryotes
EC annotation - (Blast/Profile): na
                       PHOBIUS SP: Y
                      PHOBIUS TMD: 0
               RefSeq annotation: hypothetical protein DDB_G0281495
          Name of enzyme/protein: Protein containing DUF377
          KEGG PATHWAY - level 1: Function unknown
          KEGG PATHWAY - level 2: na
```

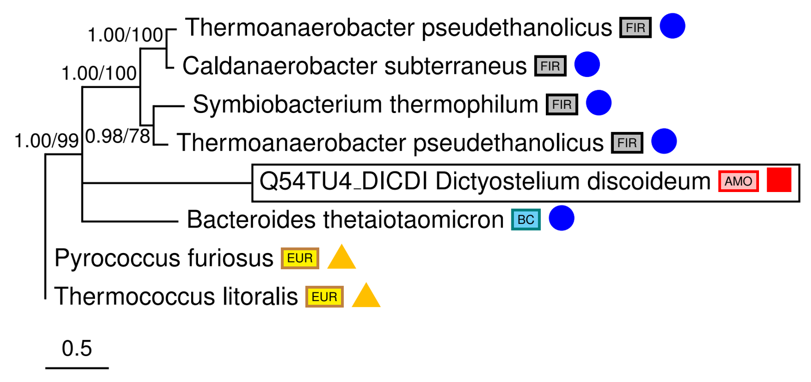

TN129

Candy accession: Q55G42_DICDI
RefSeq accession: XP_647341.1
Uniprot accession: Q55G42_DICDI
Comments: LGT - DD ONLY
Species affected: DD
Adjacent taxa in tree: Prokaryotes
EC annotation - (Blast/Profile): EC:2.1.1.14
PHOBIUS SP: 0
PHOBIUS TMD: 0
RefSeq annotation: hypothetical protein DDB_G0267834
Name of enzyme/protein: 5-methyltetrahydropteroyltriglutamate---homocysteine S-methyltransferase
KEGG PATHWAY - level 1: Amino Acid Metabolism
KEGG PATHWAY - level 2: Cysteine and methionine metabolism

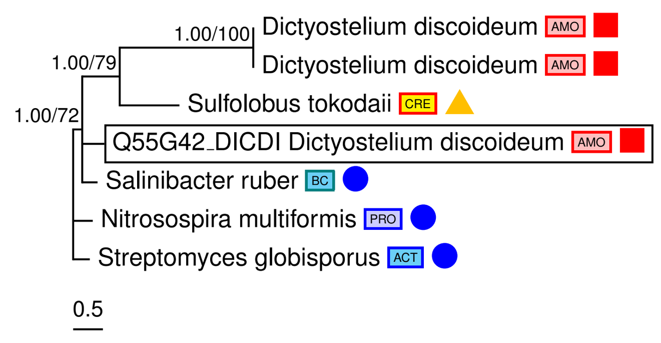

TN131

```
               Candy accession: Q57XJ4_9TRYP
              RefSeq accession: XP_843936.1
             Uniprot accession: Q57XJ4_9TRYP
                      Comments: LGT - KINETOPLASTIDS ONLY
              Species affected: LM,LD,TB,TC
          Adjacent taxa in tree: Bacteria
EC annotation - (Blast/Profile): na
                    PHOBIUS SP: 0
                   PHOBIUS TMD: 0
             RefSeq annotation: hypothetical protein
         Name of enzyme/protein: Protein containing phytanoyl-COA
                                 dioxygenase domain
         KEGG PATHWAY - level 1: Other function
         KEGG PATHWAY - level 2: na
```

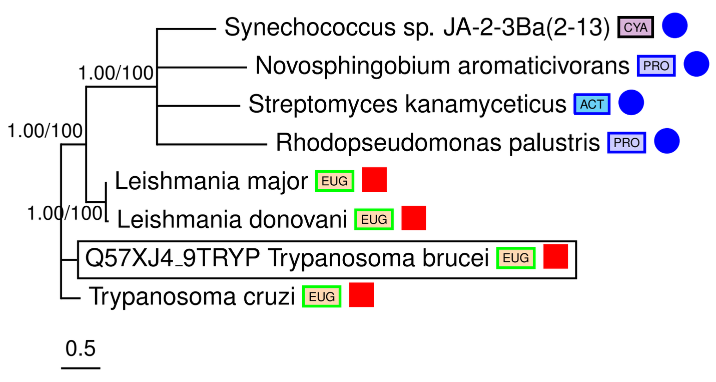

TN132

```
                Candy accession: Q57XW9_9TRYP
               RefSeq accession: XP_843730.1
              Uniprot accession: Q57XW9_9TRYP
                       Comments: LGT - KINETOPLASTIDS ONLY
               Species affected: LM,TB
          Adjacent taxa in tree: Prokaryotes
EC annotation - (Blast/Profile): EC:2.1.1.37
                     PHOBIUS SP: 0
                    PHOBIUS TMD: 0
              RefSeq annotation: cytosine-specific DNA methylase
         Name of enzyme/protein: DNA (cytosine-5-)-methyltransferase
         KEGG PATHWAY - level 1: Amino Acid Metabolism
         KEGG PATHWAY - level 2: Methionine metabolism
```

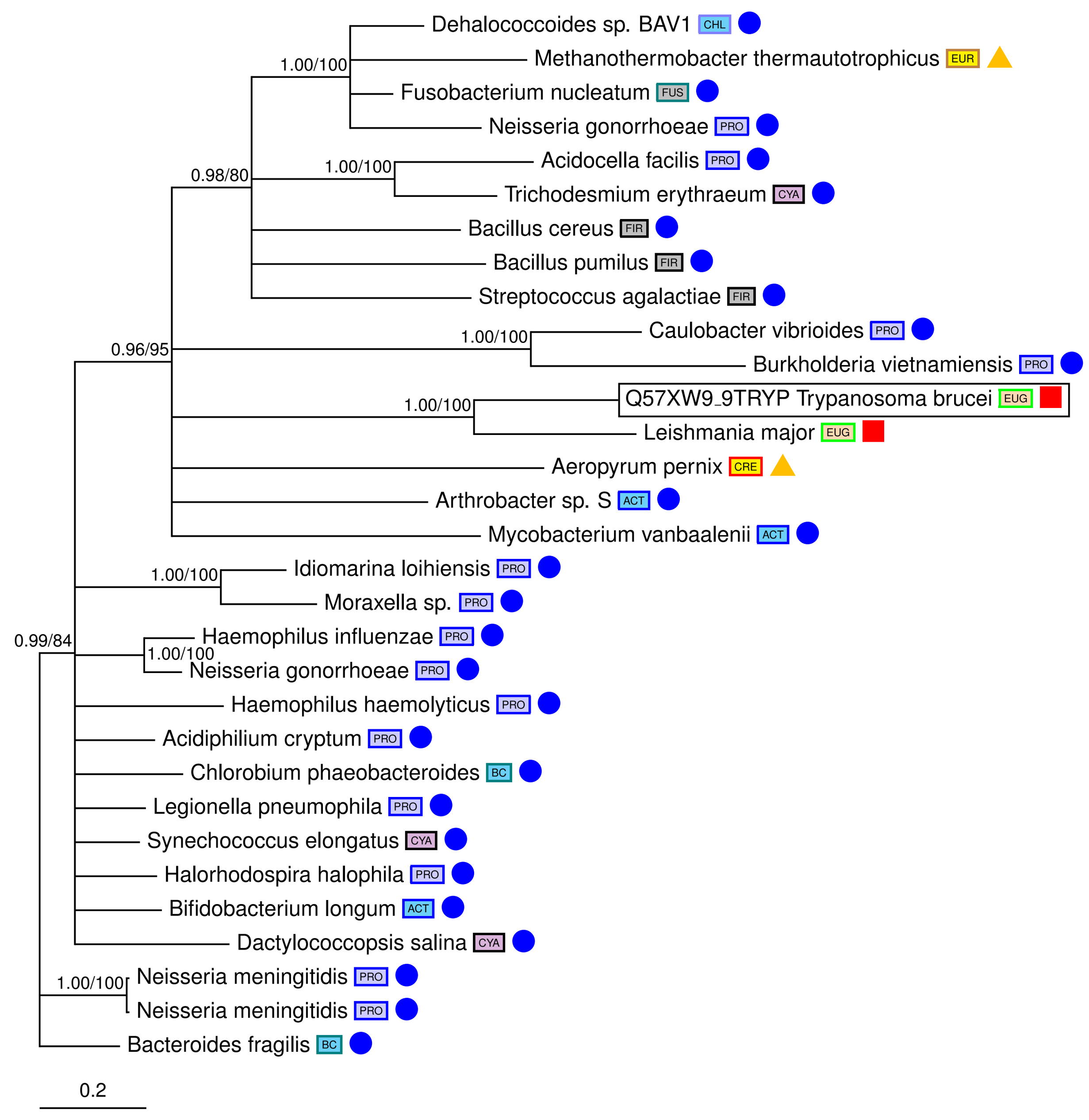

TN133

```
           Candy accession: Q86IX8_DICDI
          RefSeq accession: XP_643917.1
         Uniprot accession: Q86IX8_DICDI
                  Comments: LGT - DD AND ONE FUNGI
          Species affected: DD,SP
      Adjacent taxa in tree: Fibrobacteres/Acidobacteria - Solibacter
EC annotation - (Blast/Profile): EC:5.2.1.8
               PHOBIUS SP: Y
              PHOBIUS TMD: 0
         RefSeq annotation: cyclophilin-type peptidylprolyl cis-
                            trans isomerase
    Name of enzyme/protein: peptidylprolyl isomerase
    KEGG PATHWAY - level 1: Reaction
    KEGG PATHWAY - level 2: Reaction
```

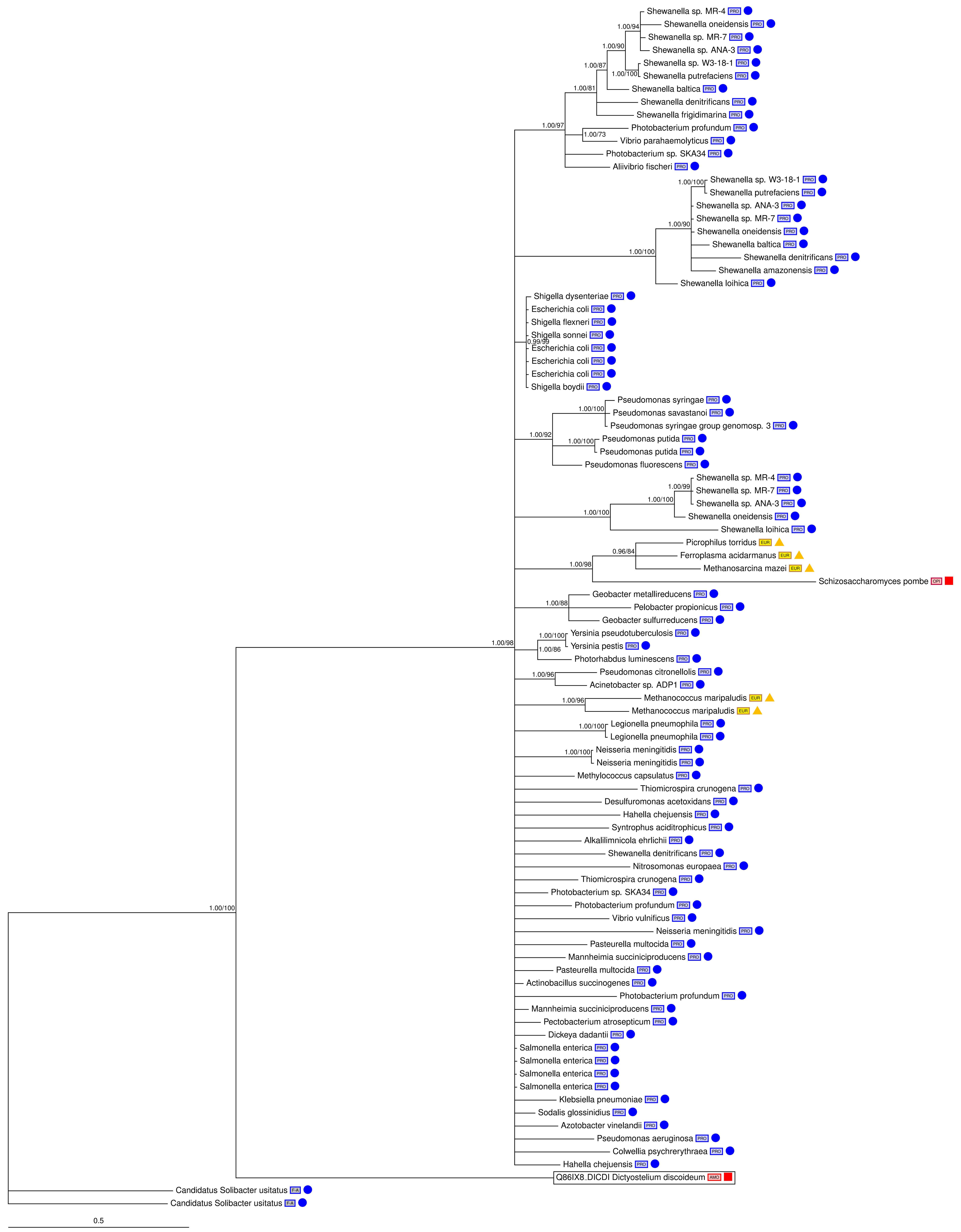

TN134

```
              Candy accession: Q86JV3_DICDI
             RefSeq accession: XP_644739.1
            Uniprot accession: Q556V8_DICDI
                     Comments: LGT - DD TWO NODES - LGT INTO FUNGI AND
                               SCHISTOSOMA
             Species affected: DD,FUNGI,SJ
        Adjacent taxa in tree: Proteobacteria
EC annotation - (Blast/Profile): na
                  PHOBIUS SP: 0
                 PHOBIUS TMD: 0
            RefSeq annotation: Dyp-type peroxidase family protein
       Name of enzyme/protein: Predicted Dyp-type peroxidase family
                               protein
       KEGG PATHWAY - level 1: Other function
       KEGG PATHWAY - level 2: na
```

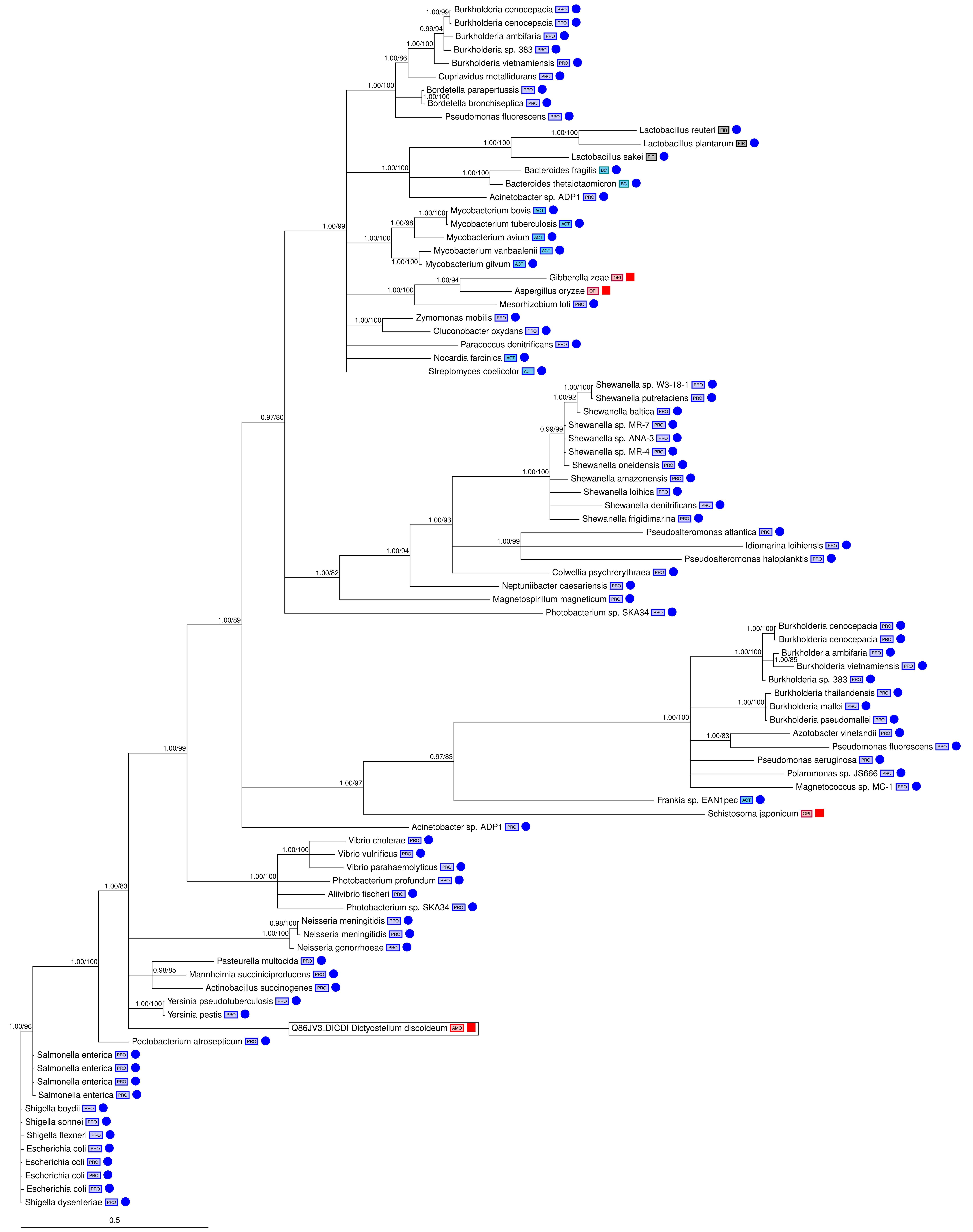

TN135

```
               Candy accession: Q86K13_DICDI
              RefSeq accession: XP_645041.1
             Uniprot accession: Q556I6_DICDI
                      Comments: LGT - DD ONLY
              Species affected: DD
         Adjacent taxa in tree: Bacteria
EC annotation - (Blast/Profile): na
                    PHOBIUS SP: 0
                   PHOBIUS TMD: 0
            RefSeq annotation: hypothetical protein DDB_G0272612
        Name of enzyme/protein: Predicted transglutaminase-like
                                superfamily
        KEGG PATHWAY - level 1: Function unknown
        KEGG PATHWAY - level 2: na
```

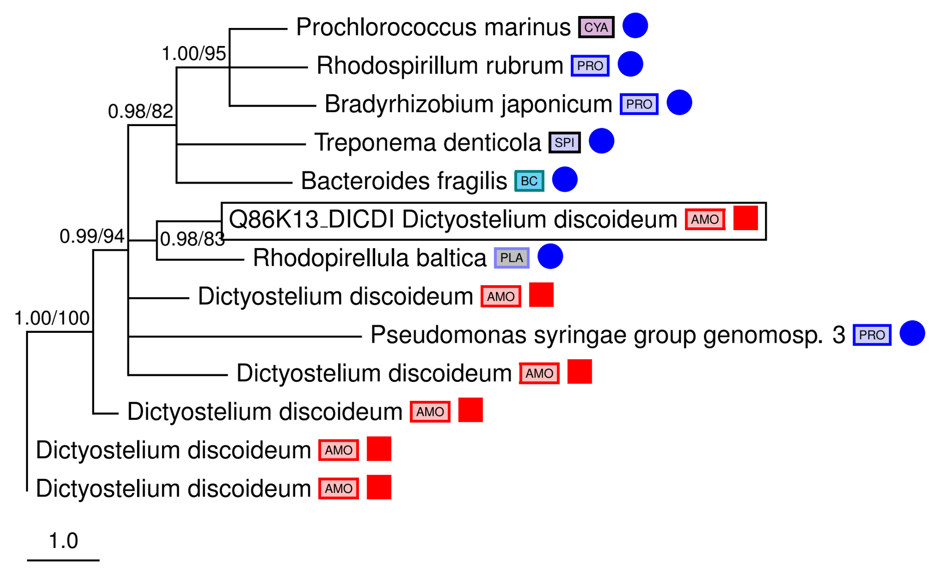

TN136

```
          Candy accession: Q387A0_9TRYP
         RefSeq accession: XP_828243.1
        Uniprot accession: Q387A0_9TRYP
                 Comments: LGT - KINETOPLASTIDS
         Species affected: LM,TB,TC
     Adjacent taxa in tree: Spirochaetes - Leptospira
EC annotation - (Blast/Profile): EC:1.3.99.10
               PHOBIUS SP: Y
              PHOBIUS TMD: 0
        RefSeq annotation: isovaleryl-coA dehydrogenase
   Name of enzyme/protein: Isovaleryl-coA dehydrogenase
   KEGG PATHWAY - level 1: Amino Acid Metabolism
   KEGG PATHWAY - level 2: Valine, leucine and isoleucine
                           degradation
```

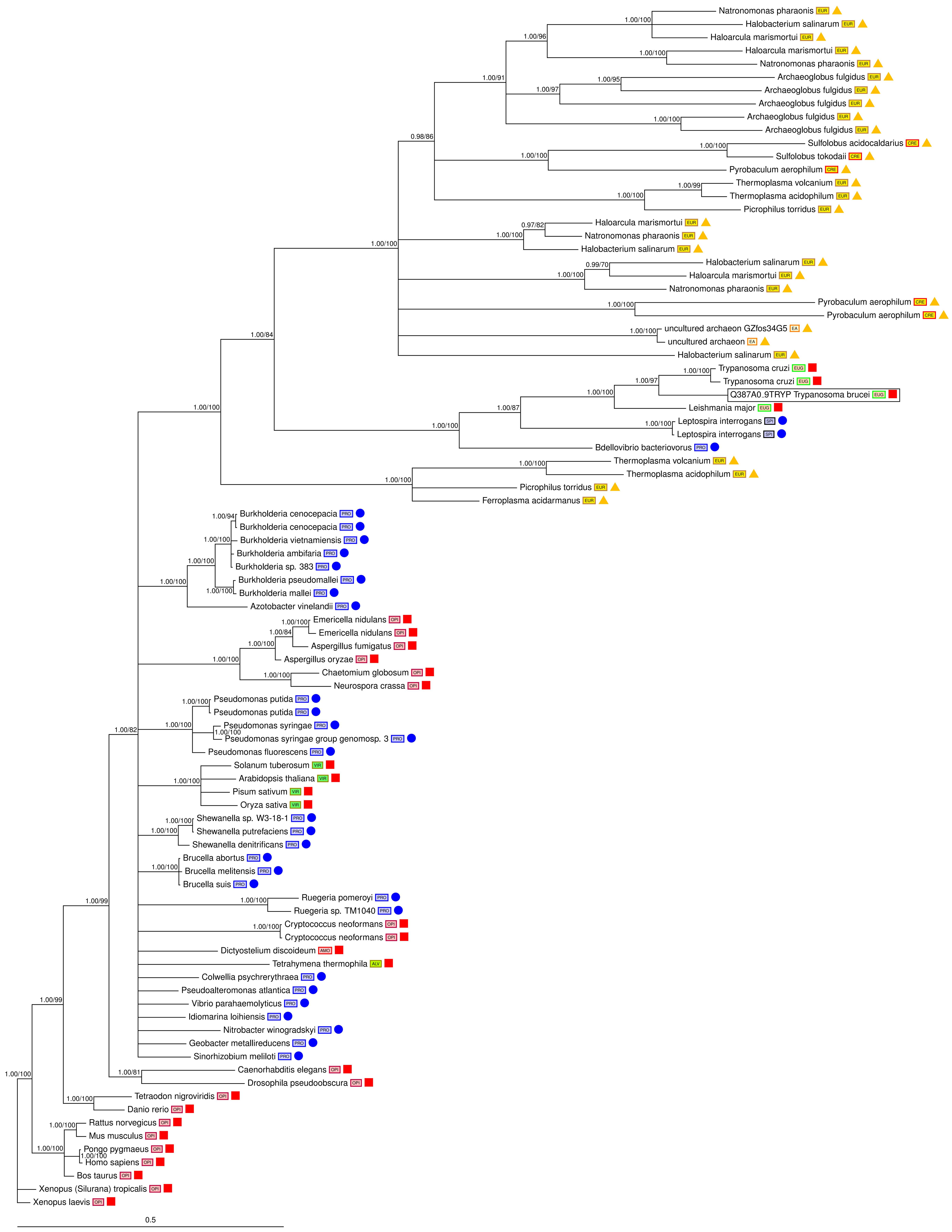

TN137

```
              Candy accession: Q512A6_ENTHI
             RefSeq accession: XP_653060.1
            Uniprot accession: C4LYV7_ENTHI
                     Comments: LGT - EH TWO NODES
             Species affected: EH
        Adjacent taxa in tree: Bacteroidetes/Chlorobi group
EC annotation - (Blast/Profile): EC:3.2.2.1
                  PHOBIUS SP: 0
                 PHOBIUS TMD: 0
            RefSeq annotation: A/G-specific adenine glycosylase
       Name of enzyme/protein:  A/G-specific adenine glycosylase
        KEGG PATHWAY - level 1: Nucleotide Metabolism
        KEGG PATHWAY - level 2: Purine metabolism
```

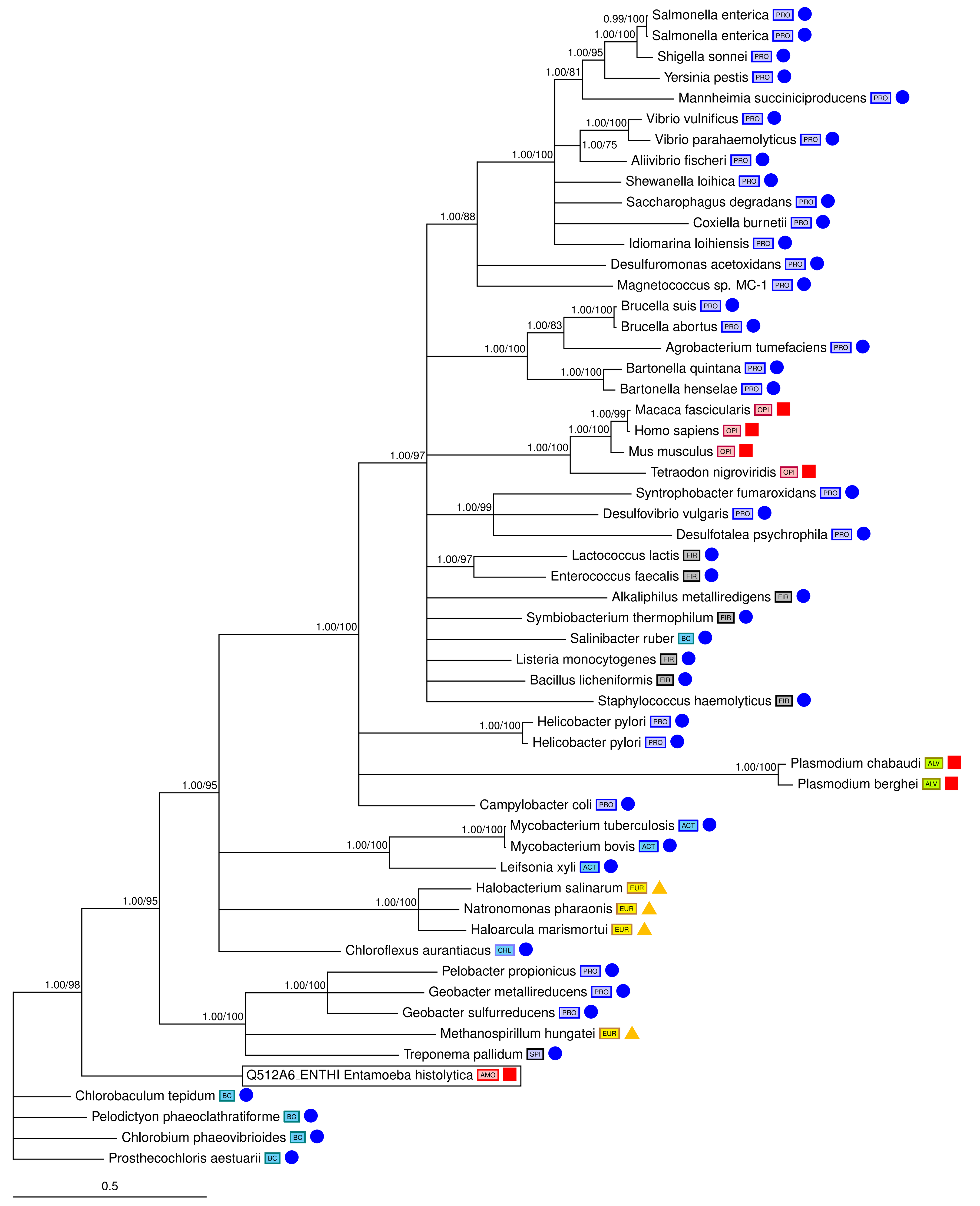

TN138

```
                 Candy accession: Q518L2_ENTHI
                RefSeq accession: XP_654665.1
               Uniprot accession: C4LZ28_ENTHI
                        Comments: LGT - EH TWO NODES + FUNGI
                Species affected: EH
          Adjacent taxa in tree: Firmicutes
EC annotation - (Blast/Profile): EC:3.2.1.113
                      PHOBIUS SP: Y
                     PHOBIUS TMD: 0
               RefSeq annotation: alpha-1,2-mannosidase
         Name of enzyme/protein: Protein containing alpha-1,2-mannosidase
                                 domain (Glycosyl hydrolase family 92)
        KEGG PATHWAY - level 1: Glycan Biosynthesis and Metabolism
        KEGG PATHWAY - level 2: Glycan biosynthesis and metabolism
```

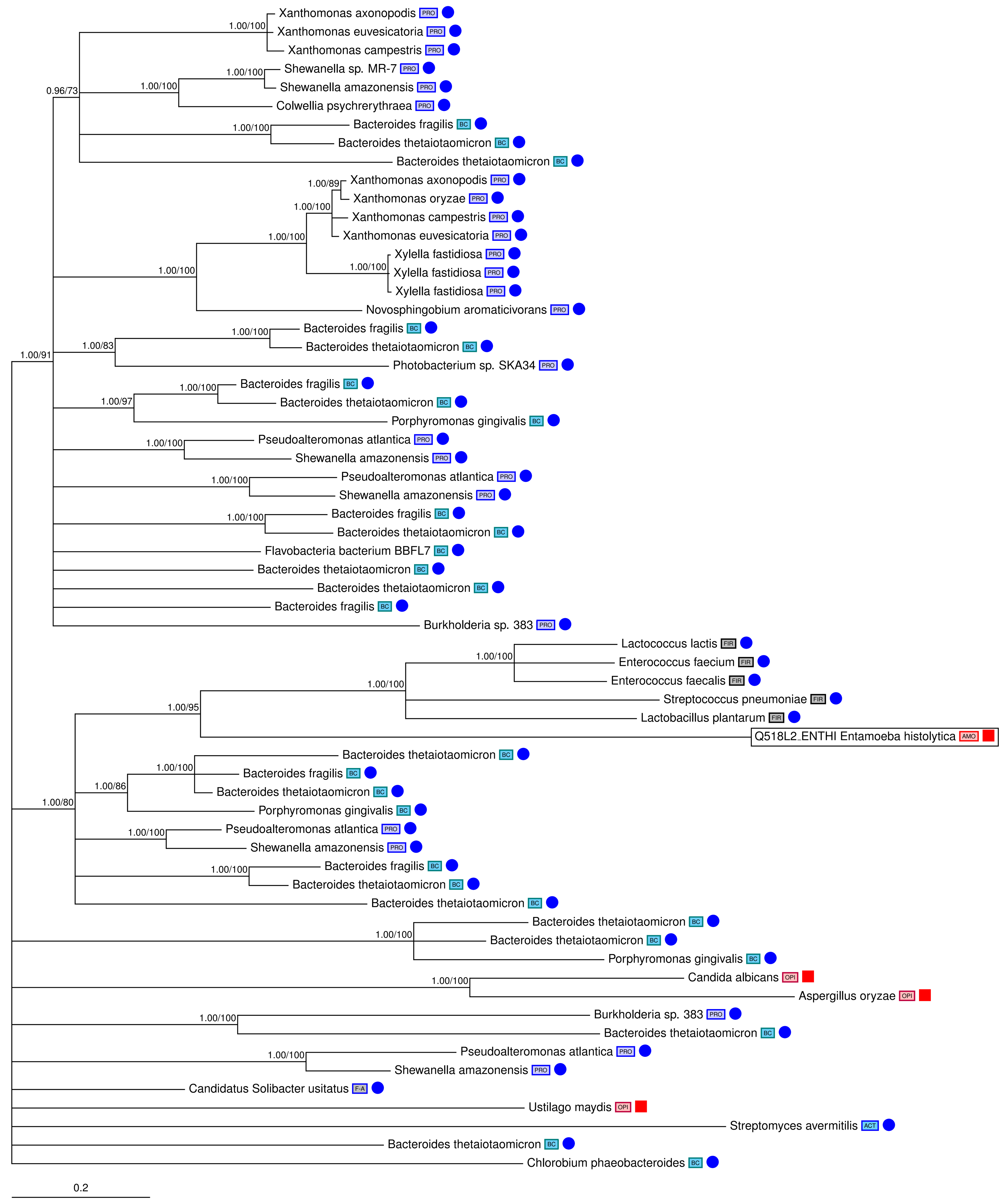

TN139

```
         Candy accession: Q519U0_ENTHI
        RefSeq accession: XP_654999.1
       Uniprot accession: C4LWS6_ENTHI
                Comments: LGT - EH ONLY
        Species affected: EH
    Adjacent taxa in tree: Archaea
EC annotation - (Blast/Profile): EC:2.7.7.39
              PHOBIUS SP: 0
             PHOBIUS TMD: 0
       RefSeq annotation: cytidylyltransferase
   Name of enzyme/protein: glycerol-3-phosphate
                           cytidylyltransferase
   KEGG PATHWAY - level 1: Lipid Metabolism
   KEGG PATHWAY - level 2: Glycerophospholipid metabolism
```

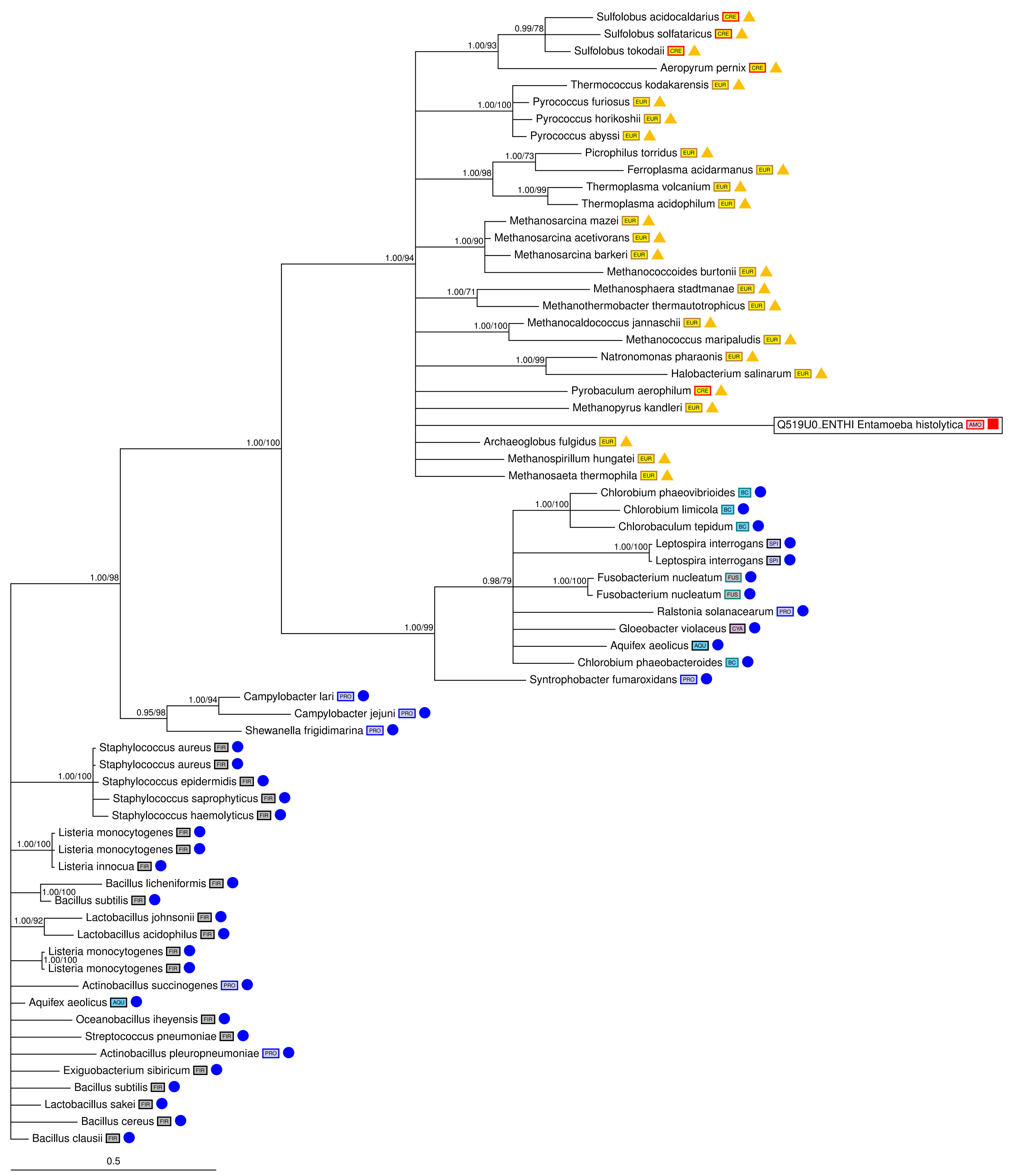

TN140

```
                 Candy accession: Q583A8_9TRYP
                RefSeq accession: XP_844420.1
               Uniprot accession: Q583A8_9TRYP
                        Comments: LGT - KINETOPLASTIDS TWO NODES
                Species affected: TB,TC
           Adjacent taxa in tree: Bacteroidetes/Chlorobi
EC annotation - (Blast/Profile): EC:4.1.3.4
                      PHOBIUS SP: 0
                     PHOBIUS TMD: 0
               RefSeq annotation: hydroxymethylglutaryl-CoA lyase
        Name of enzyme/protein: 3-hydroxy-3-methylglutaryl-CoA lyase
        KEGG PATHWAY - level 1: Lipid Metabolism, Amino Acid Metabolism
        KEGG PATHWAY - level 2: Synthesis and degradation of ketone
                                bodies, Valine, leucine and isoleucine
                                degradation
```

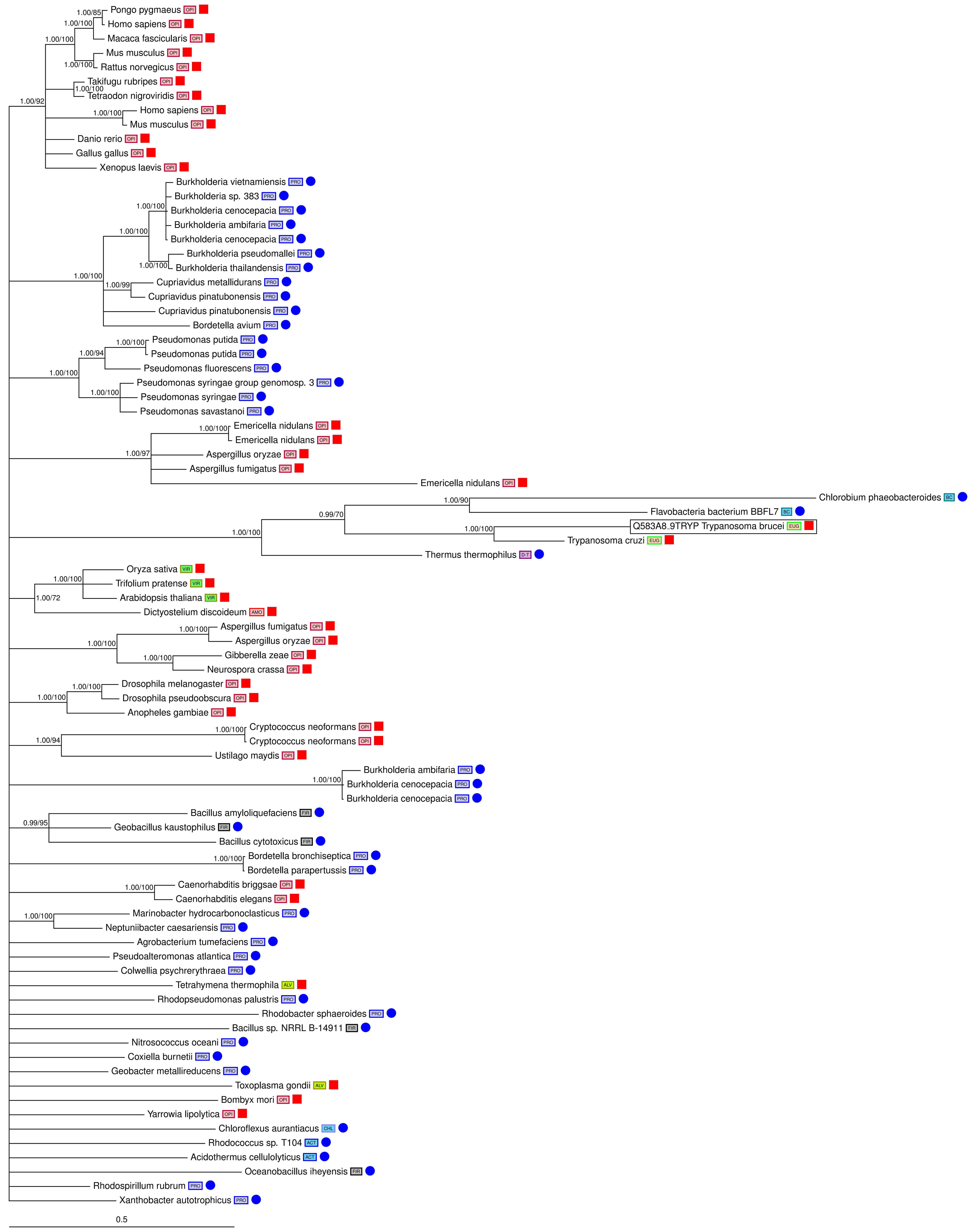

TN141

```
                Candy accession: T57_01777
               RefSeq accession: XP_002368713.1
              Uniprot accession: B6KLW3_TOXGO
                       Comments: LGT - APICOMPLEXA ONLY
               Species affected: TG,PF,PV,PY
         Adjacent taxa in tree: Bacteria
EC annotation - (Blast/Profile): EC:3.4.24.-
                     PHOBIUS SP: 0
                    PHOBIUS TMD: 6
              RefSeq annotation: sterol-regulatory element binding
                                 protein site 2 protease, putative
         Name of enzyme/protein: Predicted sterol regulatory element-
                                 binding protein/ peptidase M50 family
                                 protein
         KEGG PATHWAY - level 1: Reaction
         KEGG PATHWAY - level 2: Reaction
```

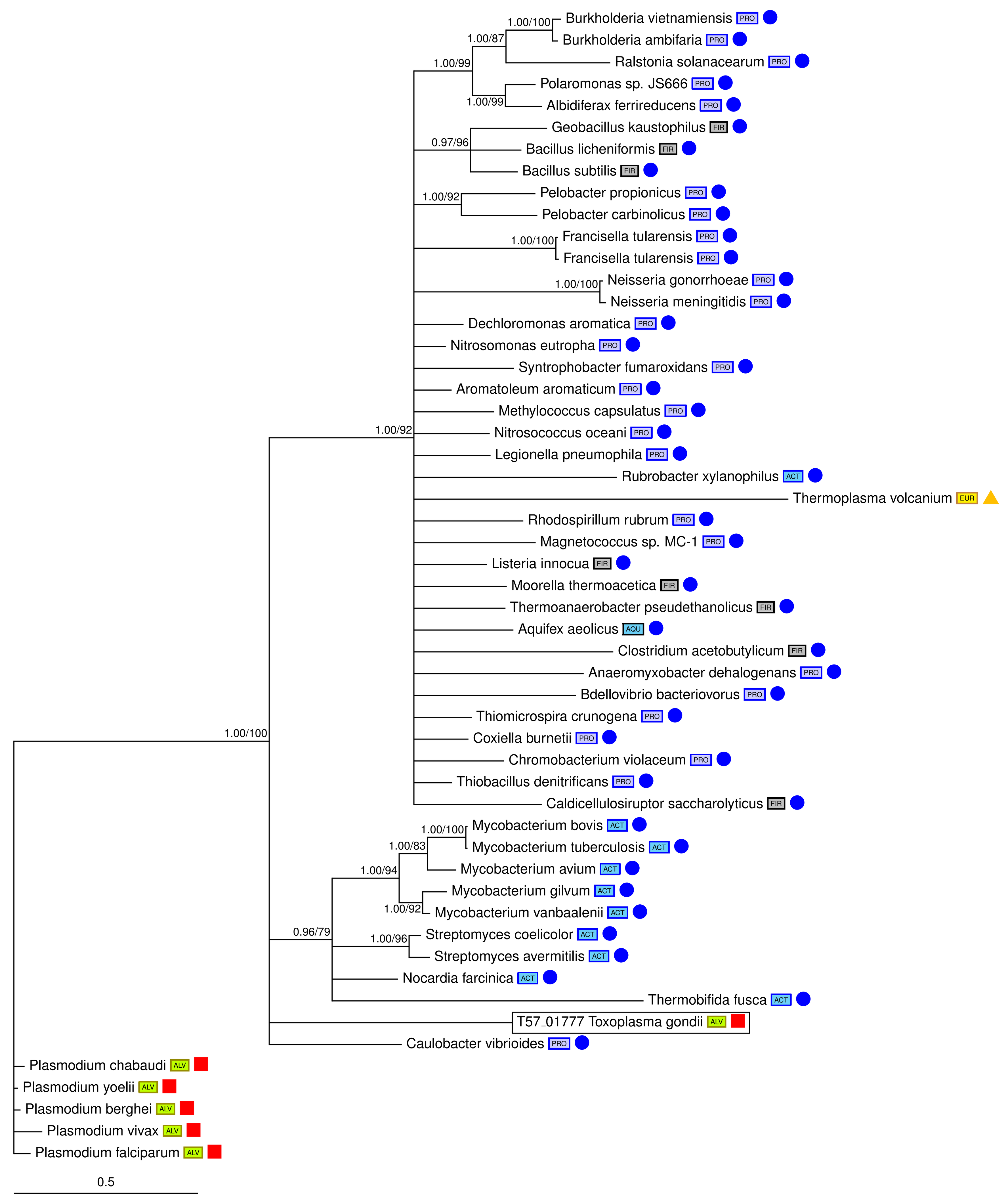

TN142

```
                 Candy accession: T76_01670
                RefSeq accession: XP_002369289.1
               Uniprot accession: B6KN66_TOXGO
                        Comments: LGT - APICOMPLEXA + TWO LIKELY CASES OF
                                  E->B LGT CASES
                Species affected: PF,PC,PV,TG
           Adjacent taxa in tree: Proteobacteria
EC annotation - (Blast/Profile): EC:1.11.1.15
                      PHOBIUS SP: Y
                     PHOBIUS TMD: 0
               RefSeq annotation: peroxiredoxin family
                                  protein/glutaredoxin, putative
        Name of enzyme/protein: peroxiredoxin
        KEGG PATHWAY - level 1: Metabolism of Other Amino Acids
        KEGG PATHWAY - level 2: Glutathione metabolism
```

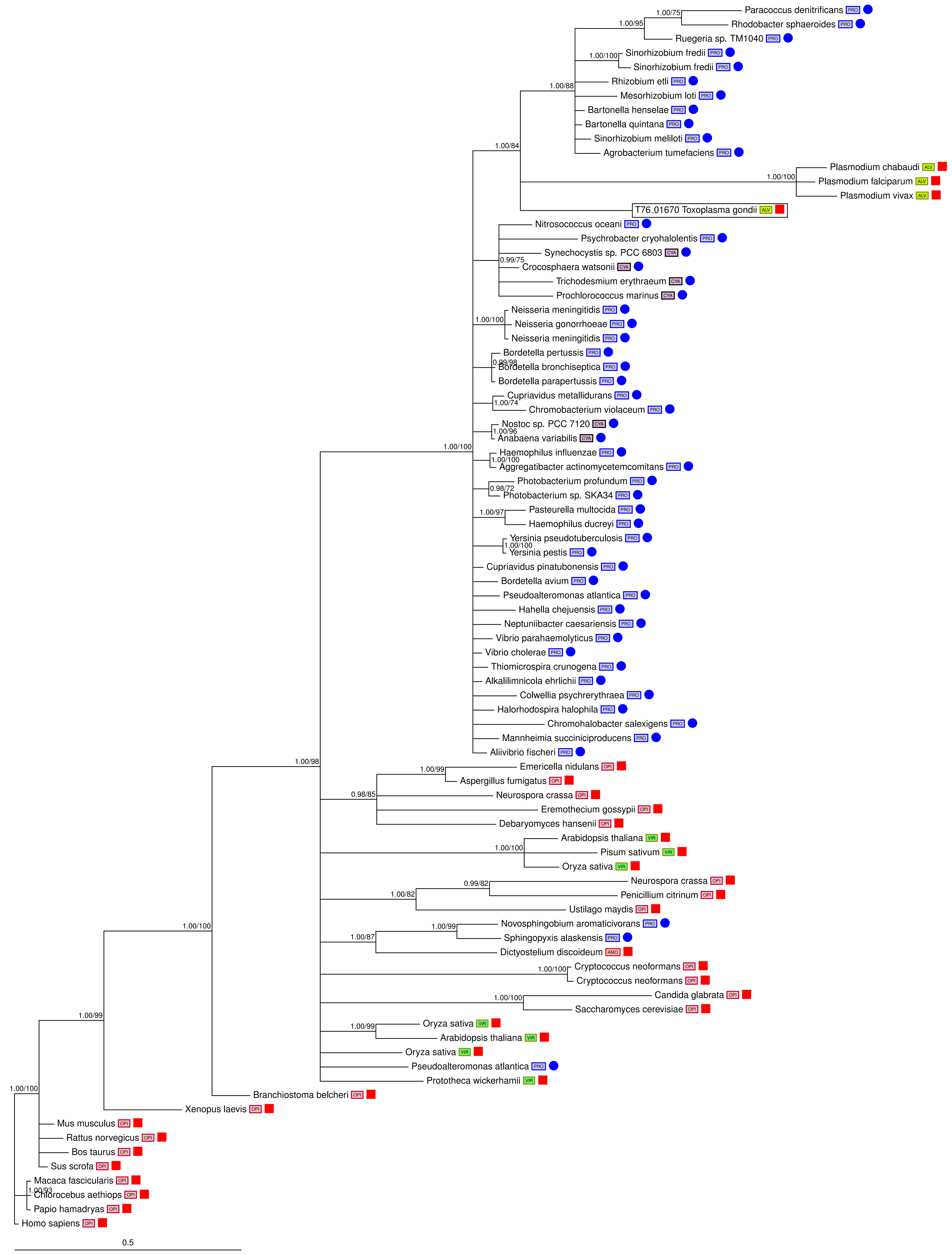

TN143

Candy accession: TV80277040
RefSeq accession: XP_001304924.1
Uniprot accession: A2FSX2_TRIVA
Comments: LGT - TV TWO NODES
Species affected: TV
Adjacent taxa in tree: Bacteroidetes/Chlorobi - Bacteroides
EC annotation - (Blast/Profile): EC:4.1.1.20
PHOBIUS SP: 0
PHOBIUS TMD: 0
RefSeq annotation: Pyridoxal-dependent decarboxylase, C-terminal sheet domain containing protein
Name of enzyme/protein: diaminopimelate decarboxylase
KEGG PATHWAY - level 1: Amino Acid Metabolism
KEGG PATHWAY - level 2: Lysine biosynthesis

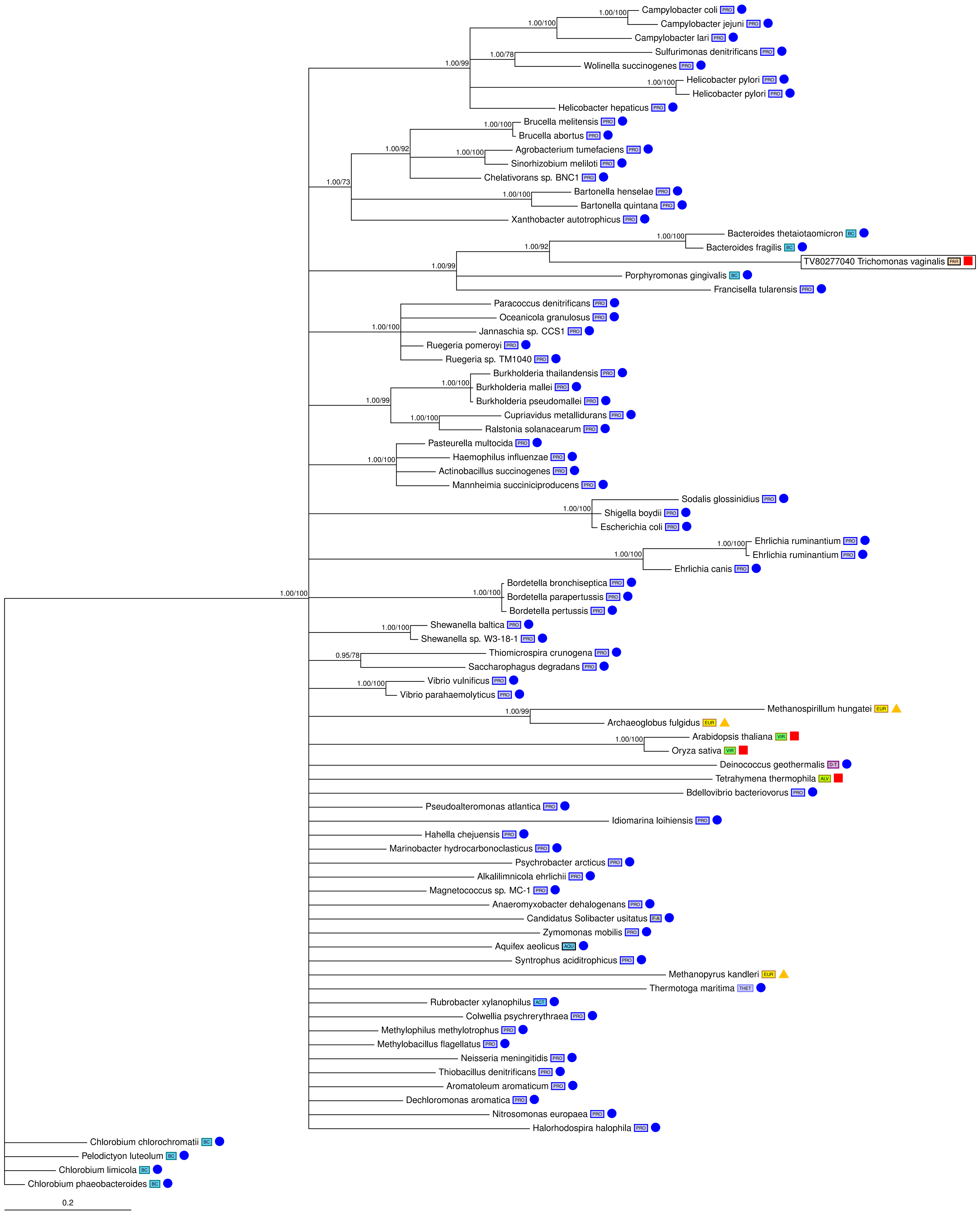

TN144

```
            Candy accession: TV80572029
           RefSeq accession: XP_001302043.1
          Uniprot accession: A2G151_TRIVA
                   Comments: LGT - TV ONLY
           Species affected: TV
      Adjacent taxa in tree: Bacteria
EC annotation - (Blast/Profile): na
                 PHOBIUS SP: 0
                PHOBIUS TMD: 0
          RefSeq annotation: methyltransferase, TIGR00027 family
                             protein
   Name of enzyme/protein: Predicted methyltransferase
   KEGG PATHWAY - level 1: Other function
   KEGG PATHWAY - level 2: na
```

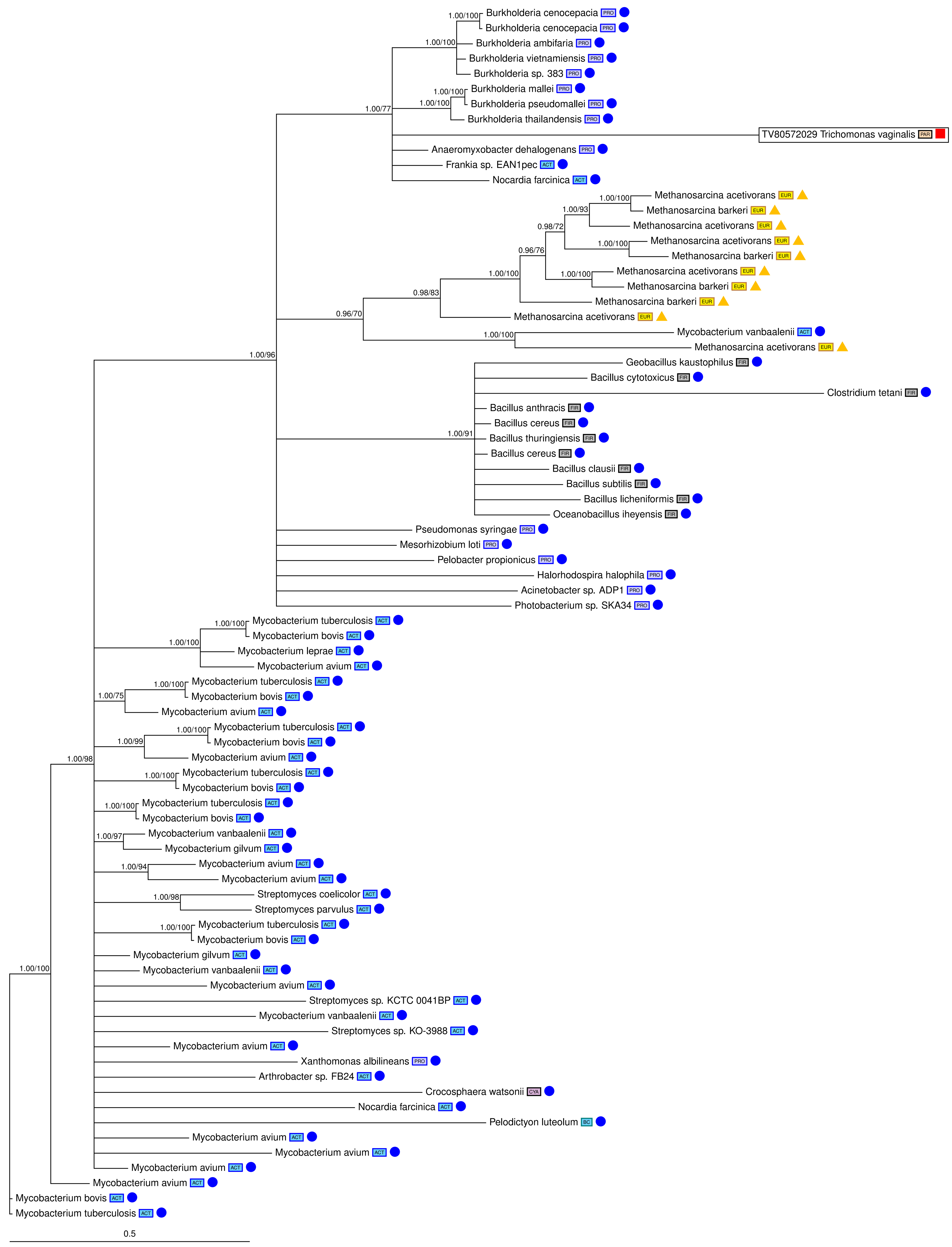

TN145

```
                Candy accession: Q50XU8_ENTHI
               RefSeq accession: XP_651815.1
              Uniprot accession: Q50XU8_ENTHI
                       Comments: LGT - AMOEBOZOA TWO NODES - DEAP LGT
                                 INTO AMOEBOZOA
               Species affected: EH,EI,EM,MB
            Adjacent taxa in tree: Bacteria
EC annotation - (Blast/Profile): na
                    PHOBIUS SP: 0
                   PHOBIUS TMD: 0
              RefSeq annotation: type A flavoprotein
         Name of enzyme/protein: Uncharacterised flavoproteins
         KEGG PATHWAY - level 1: Other function
         KEGG PATHWAY - level 2: na
```

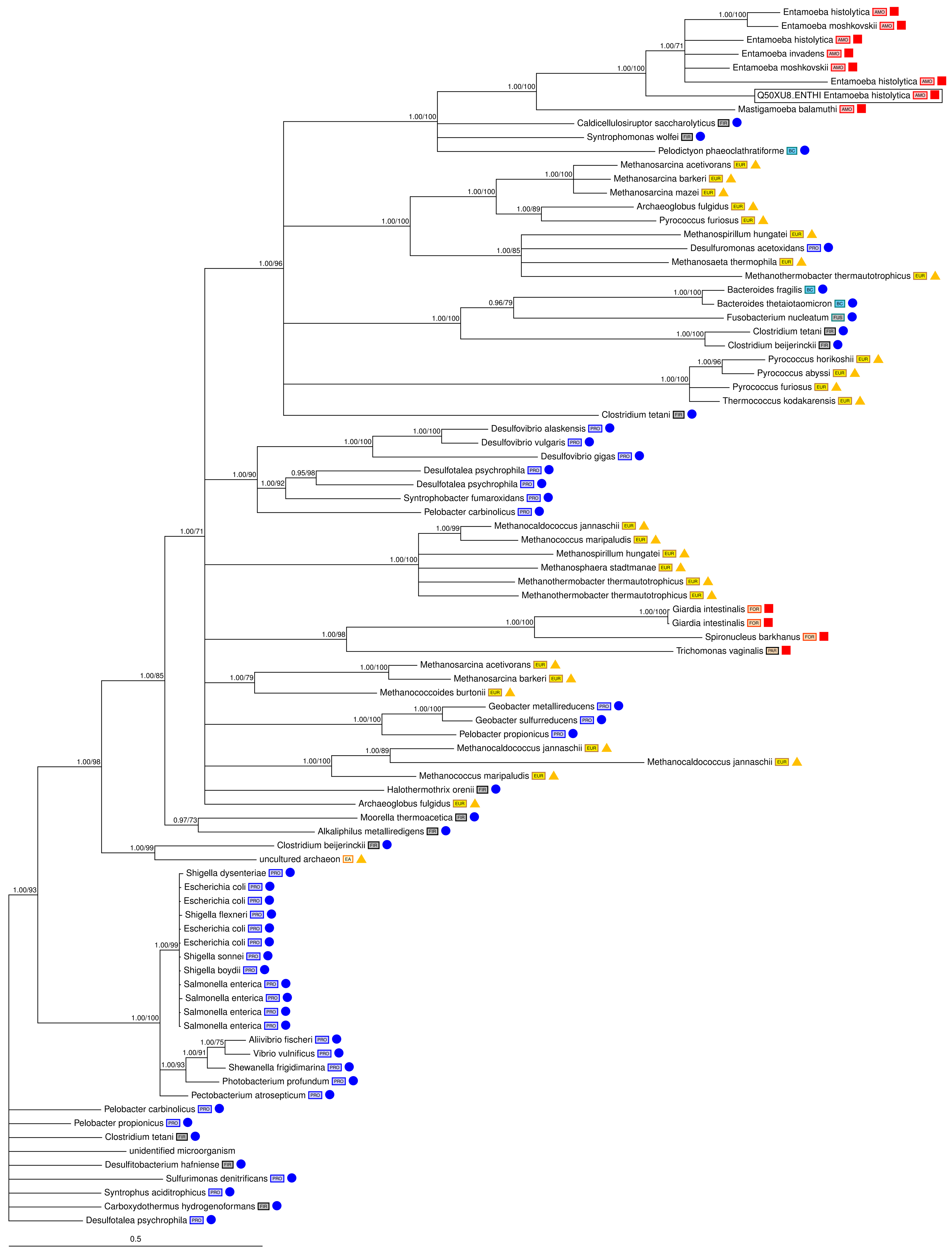

TN146

Candy accession:	TV81617225
RefSeq accession:	XP_001324287.1
Uniprot accession:	A2E5M3_TRIVA
Comments:	LGT - TV ONLY
Species affected:	TV
Adjacent taxa in tree:	Bacteria
EC annotation - (Blast/Profile):	na
PHOBIUS SP:	0
PHOBIUS TMD:	0
RefSeq annotation:	hypothetical protein
Name of enzyme/protein:	Predicted D12 class N6 adenine-specific DNA methyltransferase
KEGG PATHWAY - level 1:	Other function - Genetic Information Processing
KEGG PATHWAY - level 2:	na

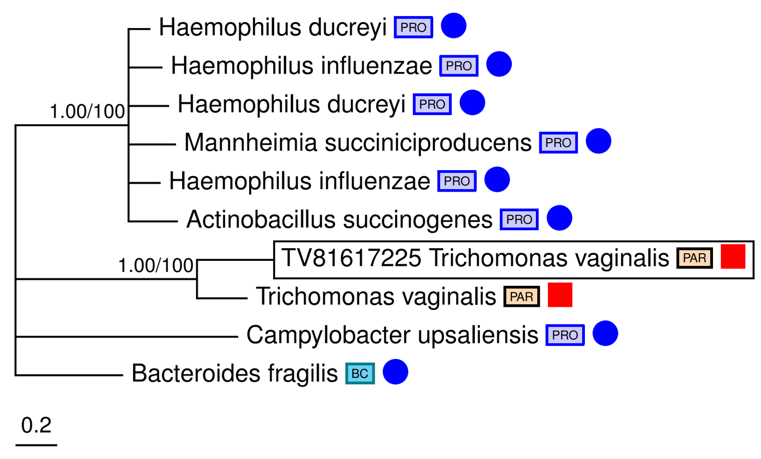

TN147

```
                Candy accession: TV82578492
               RefSeq accession: XP_001584430.1
              Uniprot accession: A2D811_TRIVA
                       Comments: LGT - TV TWO NODES
               Species affected: TV
         Adjacent taxa in tree: Bacteroidetes/Chlorobi - Chlorobium
EC annotation - (Blast/Profile): EC:2.5.1.47
                    PHOBIUS SP: 0
                   PHOBIUS TMD: 0
              RefSeq annotation: Pyridoxal-phosphate dependent enzyme
                                 family protein
          Name of enzyme/protein: cysteine synthase
          KEGG PATHWAY - level 1: Amino Acid Metabolism, Energy Metabolism
          KEGG PATHWAY - level 2: Cysteine and methionine metabolism,
                                 Sulfur metabolism
```

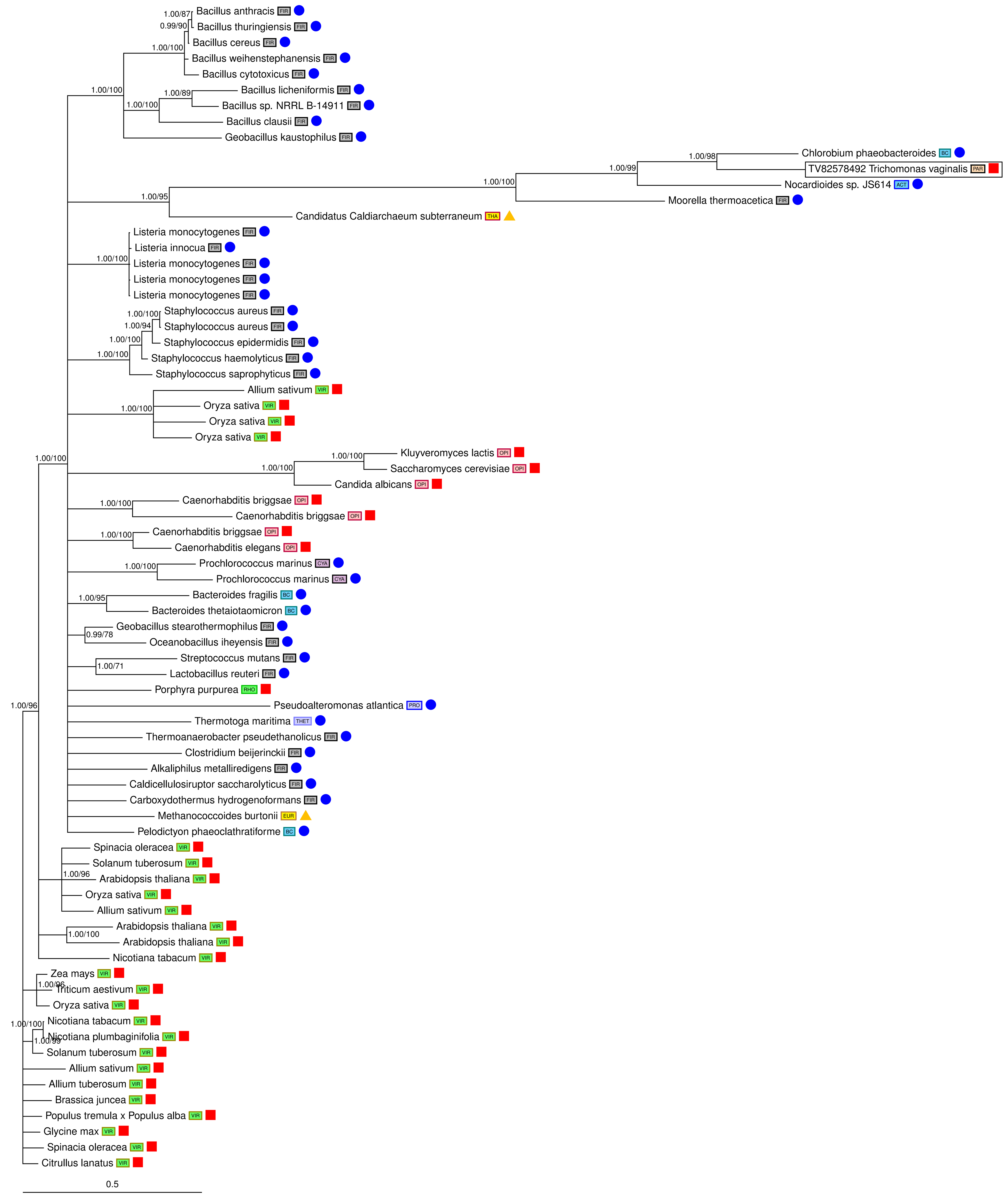

TN148

```
                Candy accession: TV84293003
               RefSeq accession: XP_001321311.1
              Uniprot accession: A2EDX6_TRIVA
                       Comments: LGT - TV ONLY - PHAGES AND BACTERIA
               Species affected: TV
          Adjacent taxa in tree: Bacteria/phage
EC annotation - (Blast/Profile): na
                     PHOBIUS SP: 0
                    PHOBIUS TMD: 0
              RefSeq annotation: hypothetical protein
        Name of enzyme/protein: Protein containing phage tail repeat
                                 like domain
        KEGG PATHWAY - level 1: Function unknown
        KEGG PATHWAY - level 2: na
```

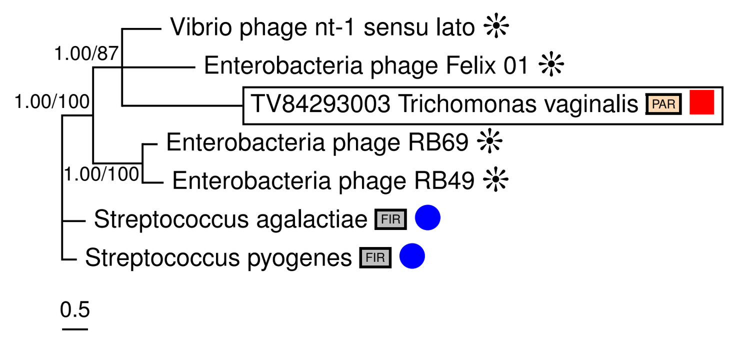

TN149

```
        Candy accession: TV84767006
       RefSeq accession: XP_001295357.1
      Uniprot accession: A2GK91_TRIVA
               Comments: LGT - TWO NODES + PHAGES AND VIRUSES - 3
                         DOMAIN ToL
       Species affected: TV
  Adjacent taxa in tree: Bacteria/phage
EC annotation - (Blast/Profile): EC:2.7.4.9
             PHOBIUS SP: 0
            PHOBIUS TMD: 0
      RefSeq annotation: thymidylate kinase family protein
Name of enzyme/protein: Thymidylate kinase family protein
 KEGG PATHWAY - level 1: Nucleotide Metabolism
 KEGG PATHWAY - level 2: Pyrimidine metabolism
```

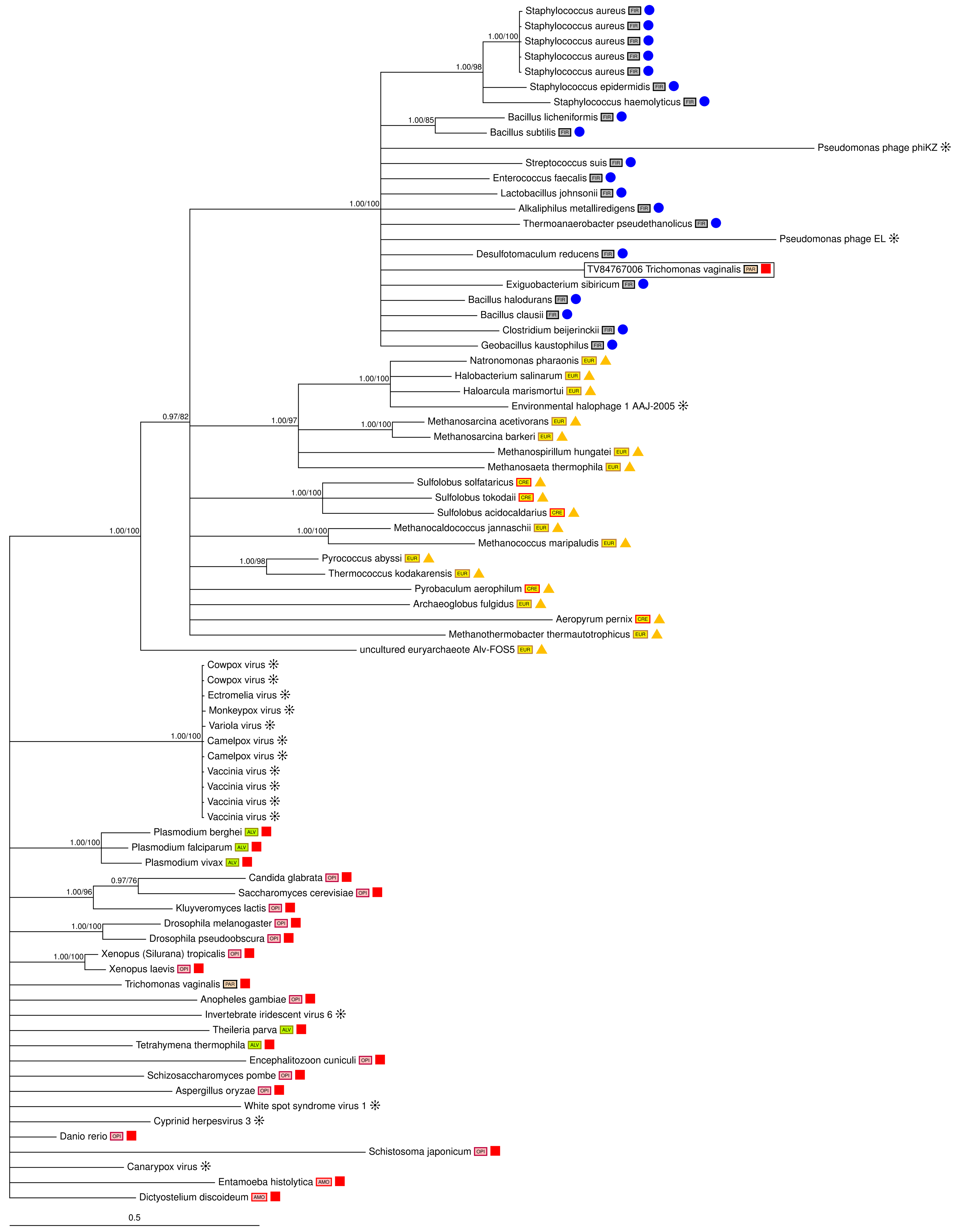

TN150

```
                Candy accession: TV85228078
               RefSeq accession: XP_001315888.1
              Uniprot accession: A2EUL6_TRIVA
                       Comments: LGT - TV TWO NODES
               Species affected: TV
         Adjacent taxa in tree: Bacteroidetes/Chlorobi - Bacteroides
EC annotation - (Blast/Profile): EC:3.2.1.22
                     PHOBIUS SP: 0
                    PHOBIUS TMD: 0
              RefSeq annotation: Melibiase family protein
       Name of enzyme/protein: alpha-galactosidase
       KEGG PATHWAY - level 1: Carbohydrate Metabolism
       KEGG PATHWAY - level 2: Galactose metabolism, Glycerolipid
                               metabolism, Sphingolipid metabolism,
                               Glycosphingolipid biosynthesis
```

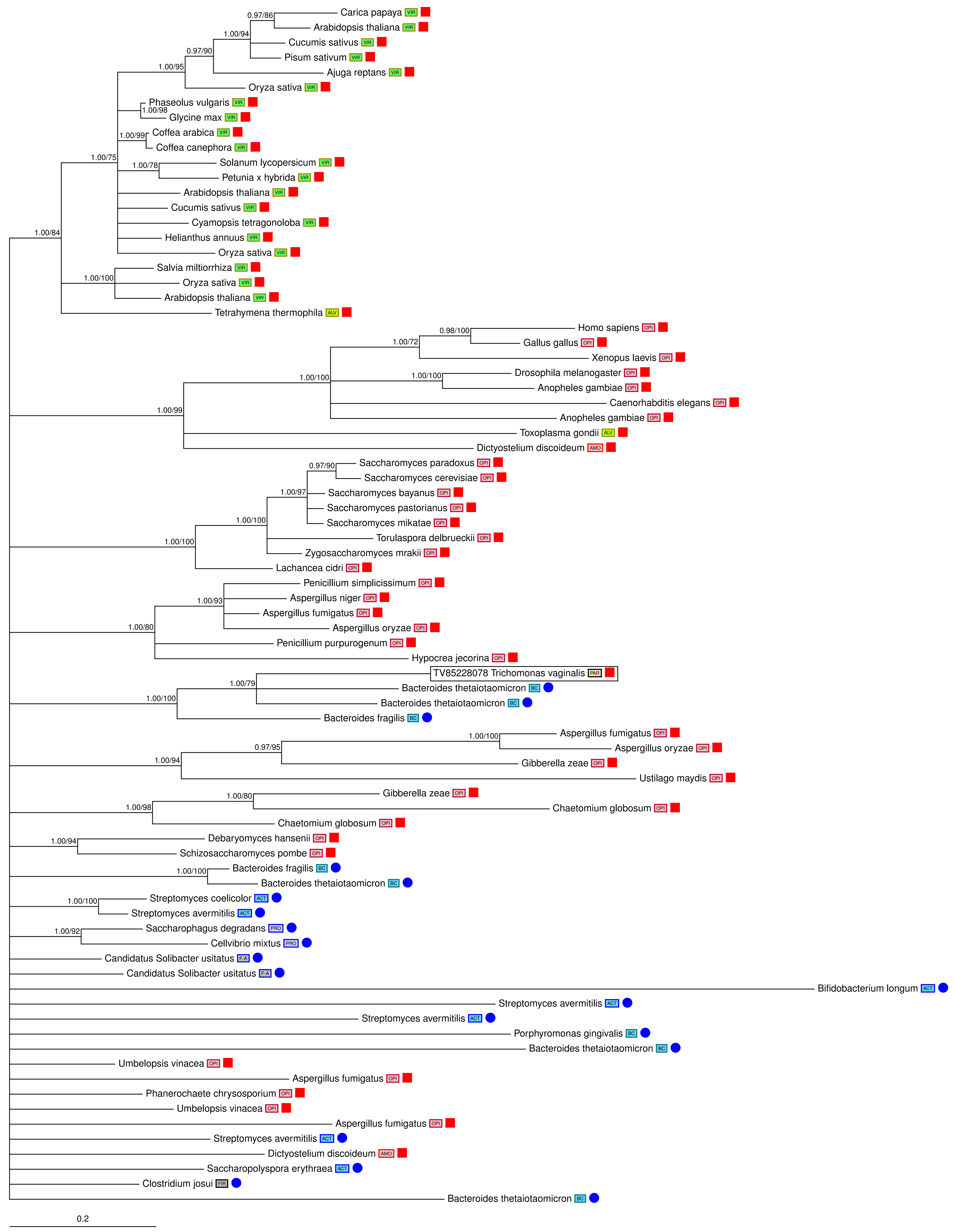

TN151

```
                 Candy accession: TV86763131
                RefSeq accession: XP_001316224.1
               Uniprot accession: A2ETN3_TRIVA
                        Comments: LGT - TV TWO NODES + C.ELEGANS
                Species affected: TV,CE
          Adjacent taxa in tree: Bacteroidetes/Chlorobi - Bacteroides
EC annotation - (Blast/Profile): na
                      PHOBIUS SP: 0
                     PHOBIUS TMD: 11
               RefSeq annotation: Acyltransferase family protein
          Name of enzyme/protein: Acyltransferase family protein
          KEGG PATHWAY - level 1: Other function - Membrane transport
          KEGG PATHWAY - level 2: na
```

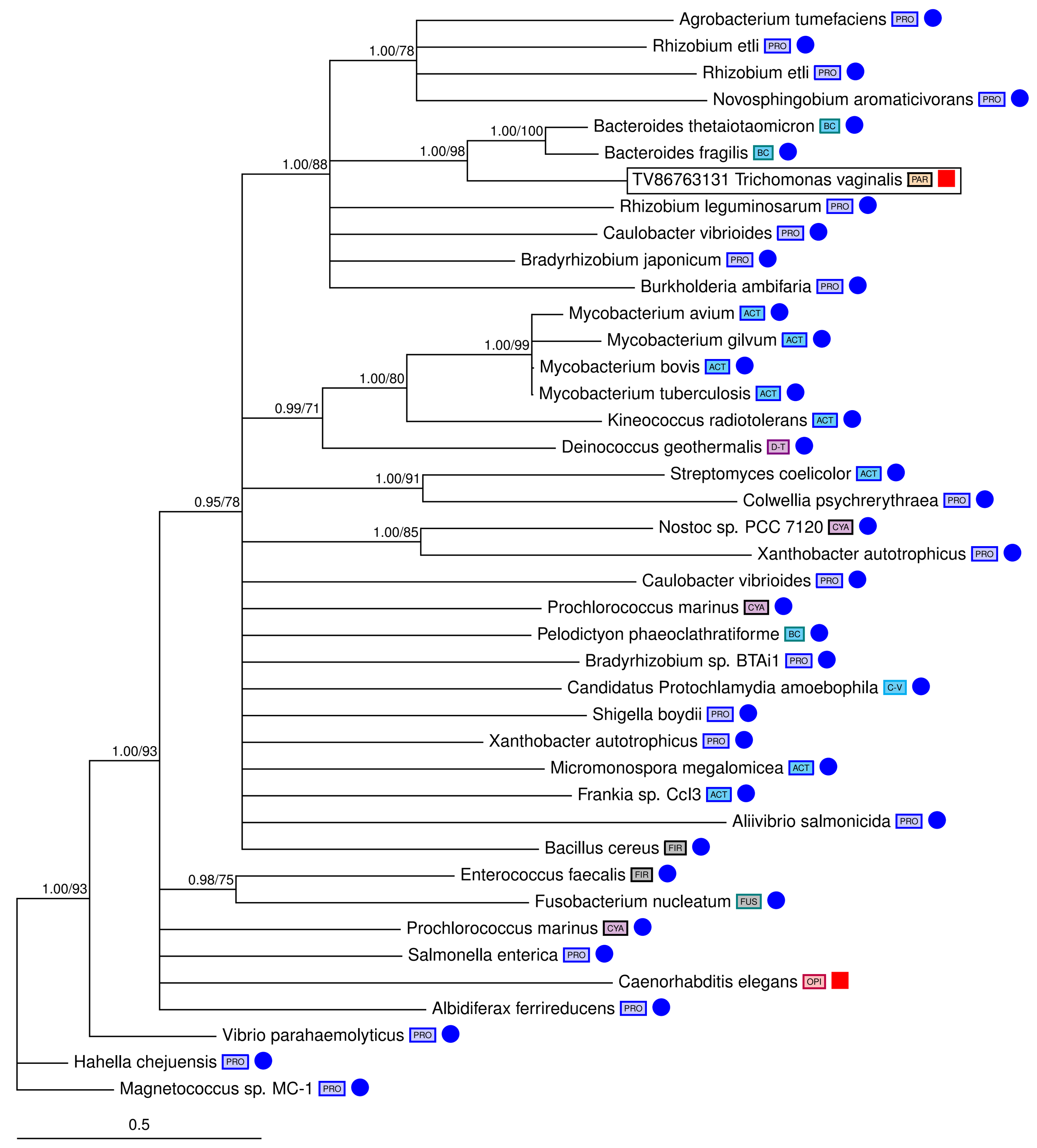

TN152

```
              Candy accession: TV87640196
              RefSeq accession: XP_001327002.1
             Uniprot accession: A2DXR9_TRIVA
                      Comments: LGT - TV TWO NODES + LGT INTO FUNGI
              Species affected: TV,FUNGI
          Adjacent taxa in tree: Bacteria
EC annotation - (Blast/Profile): na
                    PHOBIUS SP: 0
                   PHOBIUS TMD: 0
             RefSeq annotation: opdE downstream ORF 2
       Name of enzyme/protein: Predicted hydrolases of the alpha/beta
                                superfamily
       KEGG PATHWAY - level 1: Other function
       KEGG PATHWAY - level 2: na
```

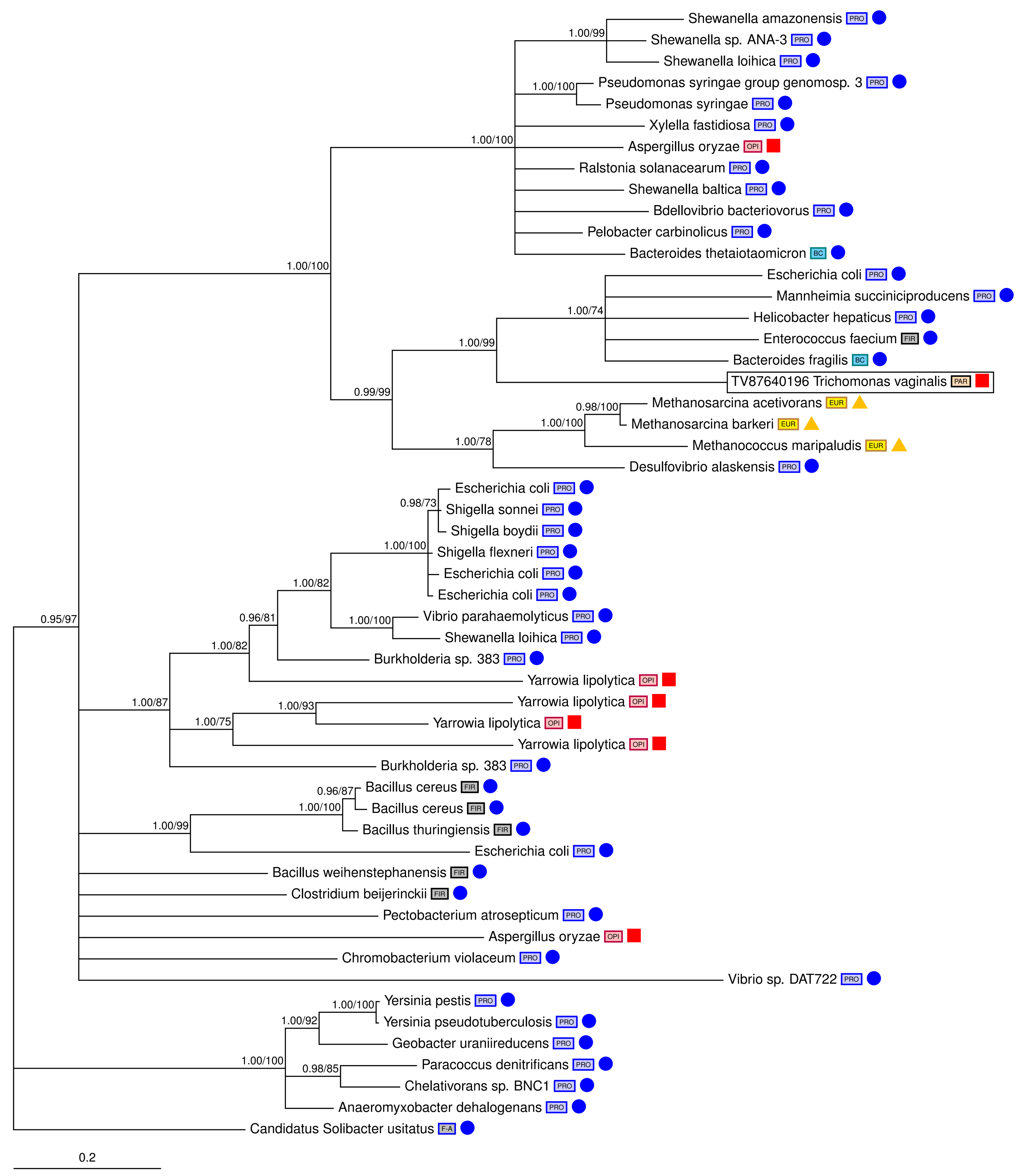

TN153

```
              Candy accession: TV87759087
             RefSeq accession: XP_001312818.1
            Uniprot accession: A2F5C5_TRIVA
                     Comments: LGT - TV TWO NODES + LGT INTO FUNGI
             Species affected: TV
       Adjacent taxa in tree: Bacteroidetes/Chlorobi - Bacteroides
EC annotation - (Blast/Profile): EC:2.1.1.-
                   PHOBIUS SP: 0
                  PHOBIUS TMD: 0
            RefSeq annotation: O-methyltransferase N-terminus family
                               protein
         Name of enzyme/protein: transferase
        KEGG PATHWAY - level 1: Reaction
        KEGG PATHWAY - level 2: Reaction
```

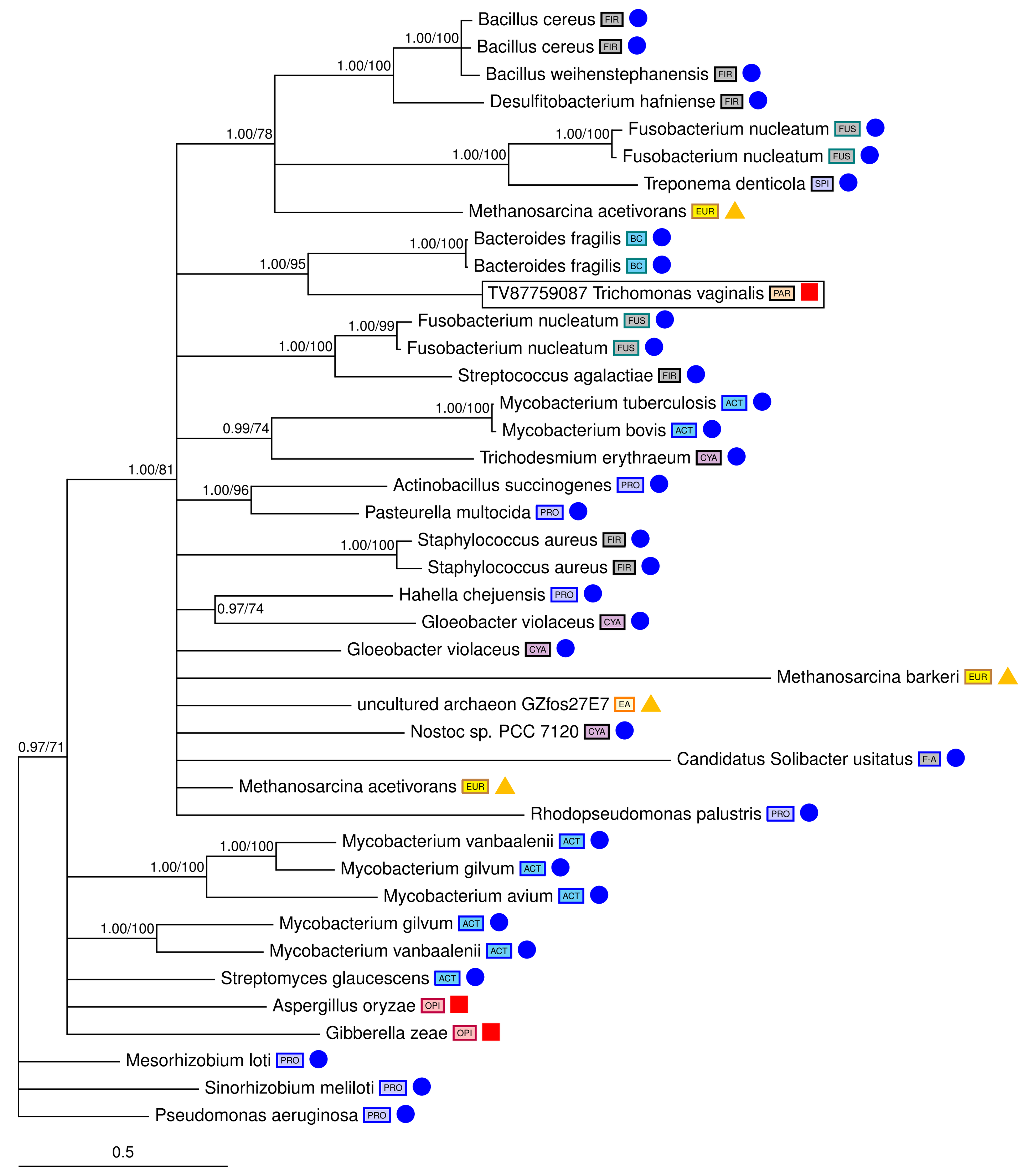

TN154

```
           Candy accession: TV88211046
          RefSeq accession: XP_001310705.1
         Uniprot accession: A2FBE1_TRIVA
                  Comments: LGT - TV TWO NODES + FUNGI
          Species affected: TV
      Adjacent taxa in tree: Proteobacteria - Psychrobacter
EC annotation - (Blast/Profile): na
               PHOBIUS SP: 0
              PHOBIUS TMD: 0
         RefSeq annotation: hypothetical protein
   Name of enzyme/protein: Protein containing DUF336
   KEGG PATHWAY - level 1: Function unknown
   KEGG PATHWAY - level 2: na
```

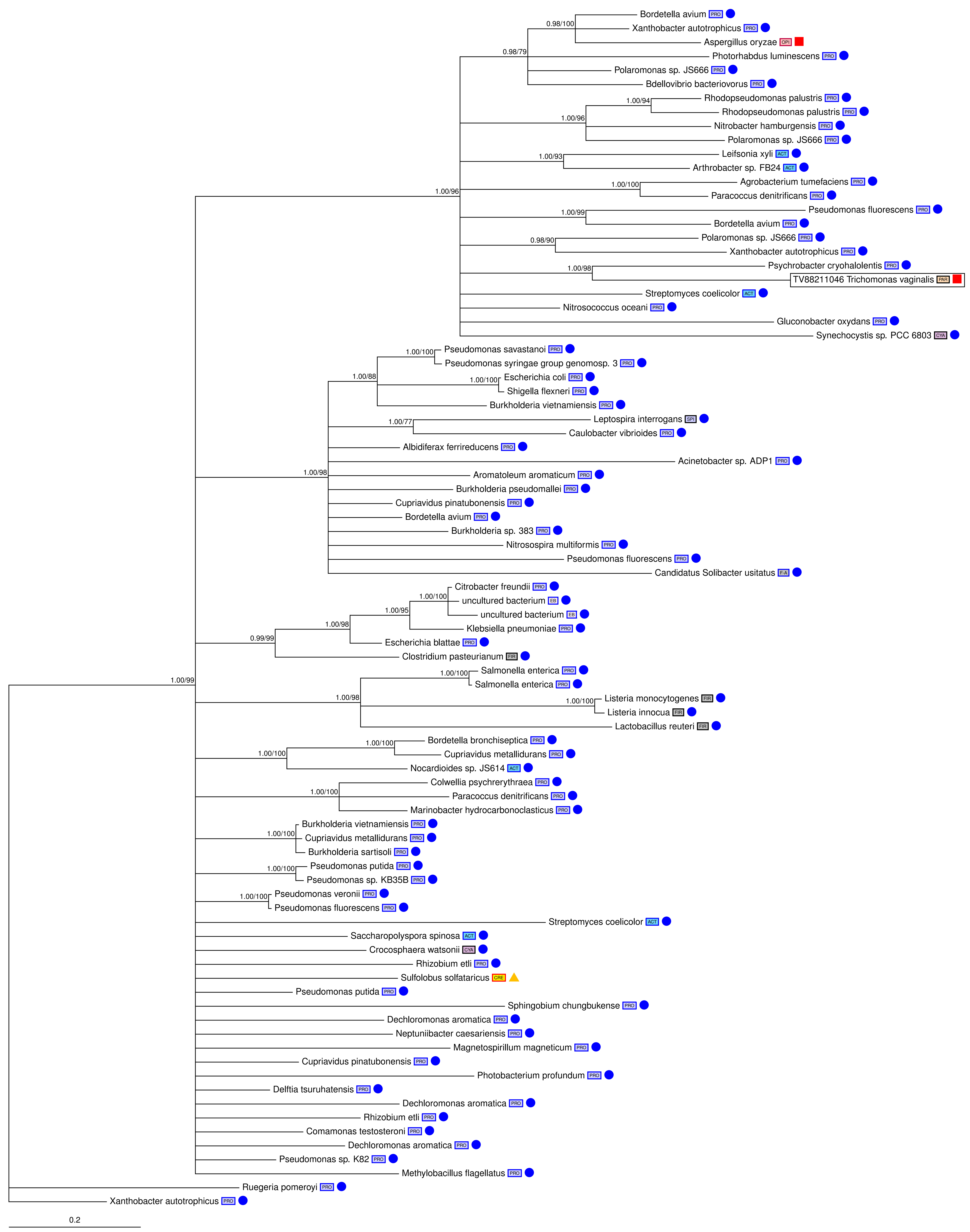

TN155

Candy accession: TV88271378
RefSeq accession: XP_001582671.1
Uniprot accession: A2DCR1_TRIVA
Comments: LGT - TV TWO NODES + FUNGI + MAMMALS
Species affected: TV,FUNGI
Adjacent taxa in tree: Bacteria
EC annotation - (Blast/Profile): EC:3.2.1.20
PHOBIUS SP: 0
PHOBIUS TMD: 0
RefSeq annotation: glycosyl hydrolase
Name of enzyme/protein: alpha-glucosidase
KEGG PATHWAY - level 1: Carbohydrate Metabolism
KEGG PATHWAY - level 2: Galactose metabolism, Starch and sucrose metabolism

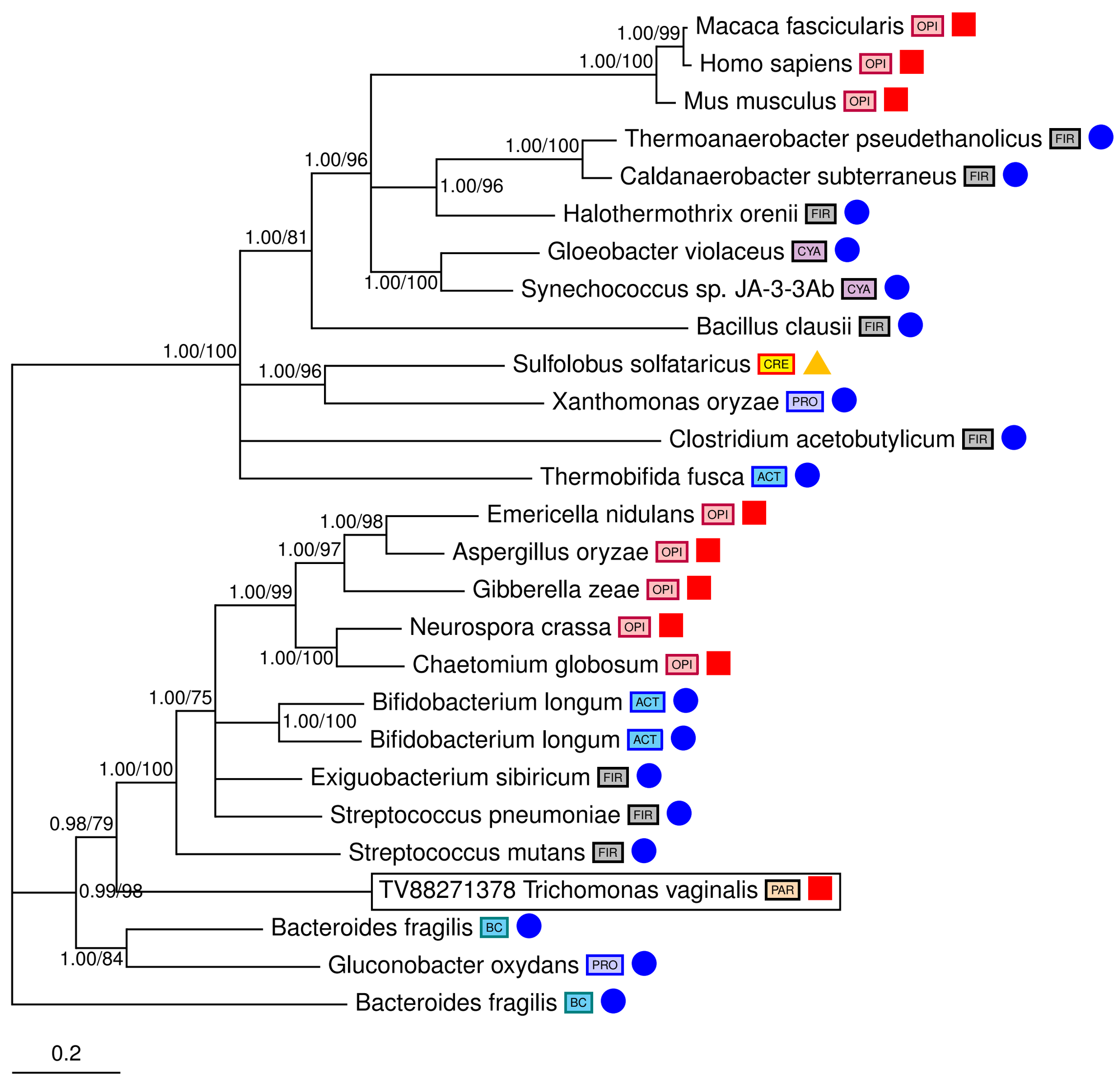

TN156

```
                Candy accession: TV88428064
               RefSeq accession: XP_001307952.1
              Uniprot accession: A2FJA8_TRIVA
                       Comments: LGT - TV ONLY
               Species affected: TV
          Adjacent taxa in tree: Bacteria
EC annotation - (Blast/Profile): EC:2.7.7.8
                     PHOBIUS SP: 0
                    PHOBIUS TMD: 0
              RefSeq annotation: S1 RNA binding domain containing protein
         Name of enzyme/protein: polyribonucleotide
                                 nucleotidyltransferase
         KEGG PATHWAY - level 1: Nucleotide Metabolism
         KEGG PATHWAY - level 2: Purine metabolism, Pyrimidine metabolism
```

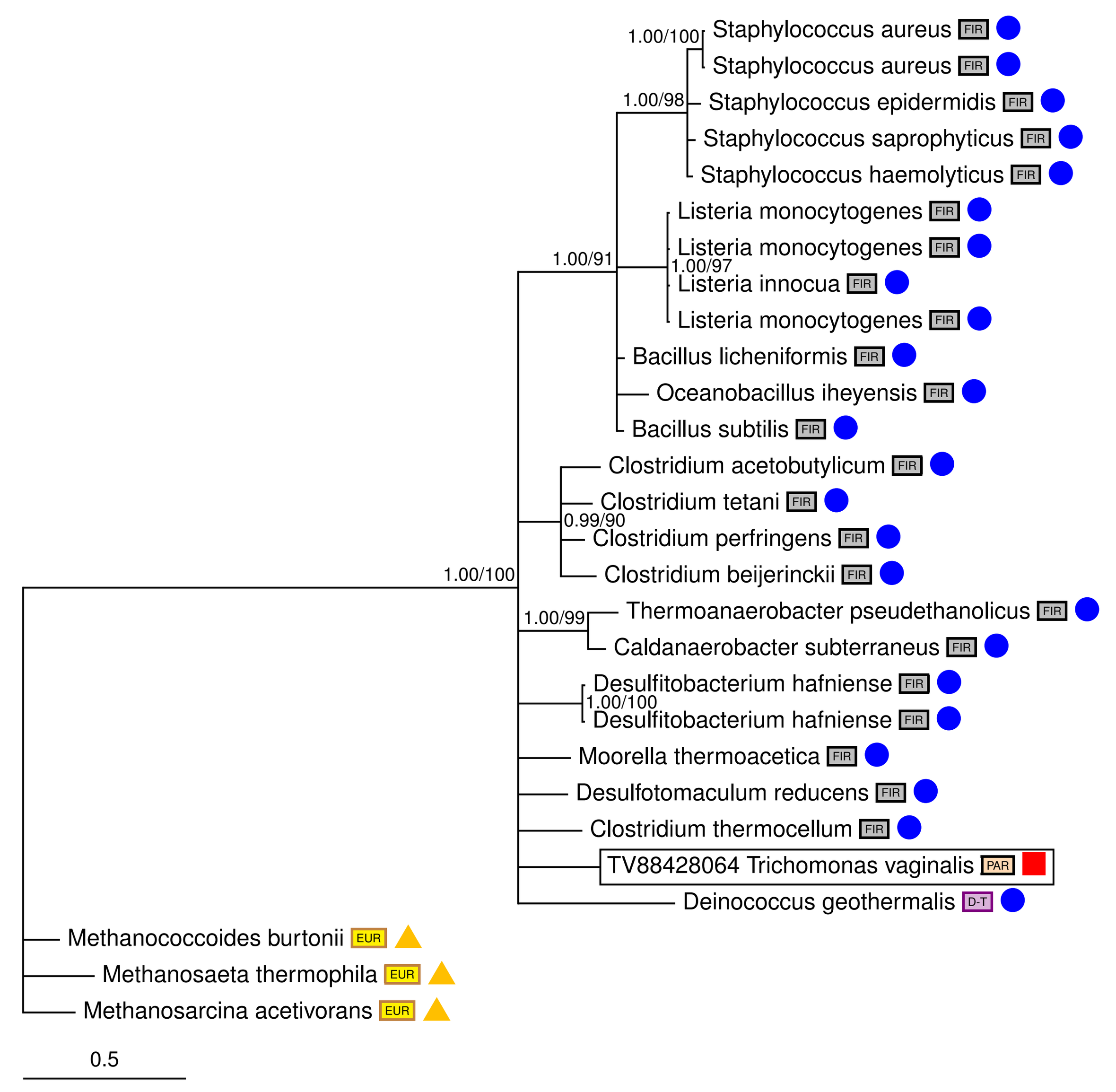

TN157

```
              Candy accession: TV88428069
             RefSeq accession: XP_001307956.1
            Uniprot accession: A2FJB2_TRIVA
                     Comments: LGT - TV ONLY
             Species affected: TV
         Adjacent taxa in tree: Firmicutes
EC annotation - (Blast/Profile): EC:2.4.1.129
                   PHOBIUS SP: 0
                  PHOBIUS TMD: 0
            RefSeq annotation: penicillin binding protein
                               transpeptidase
        Name of enzyme/protein: peptidoglycan glycosyltransferase
      KEGG PATHWAY - level 1: Glycan Biosynthesis and Metabolism
      KEGG PATHWAY - level 2: Peptidoglycan biosynthesis
```

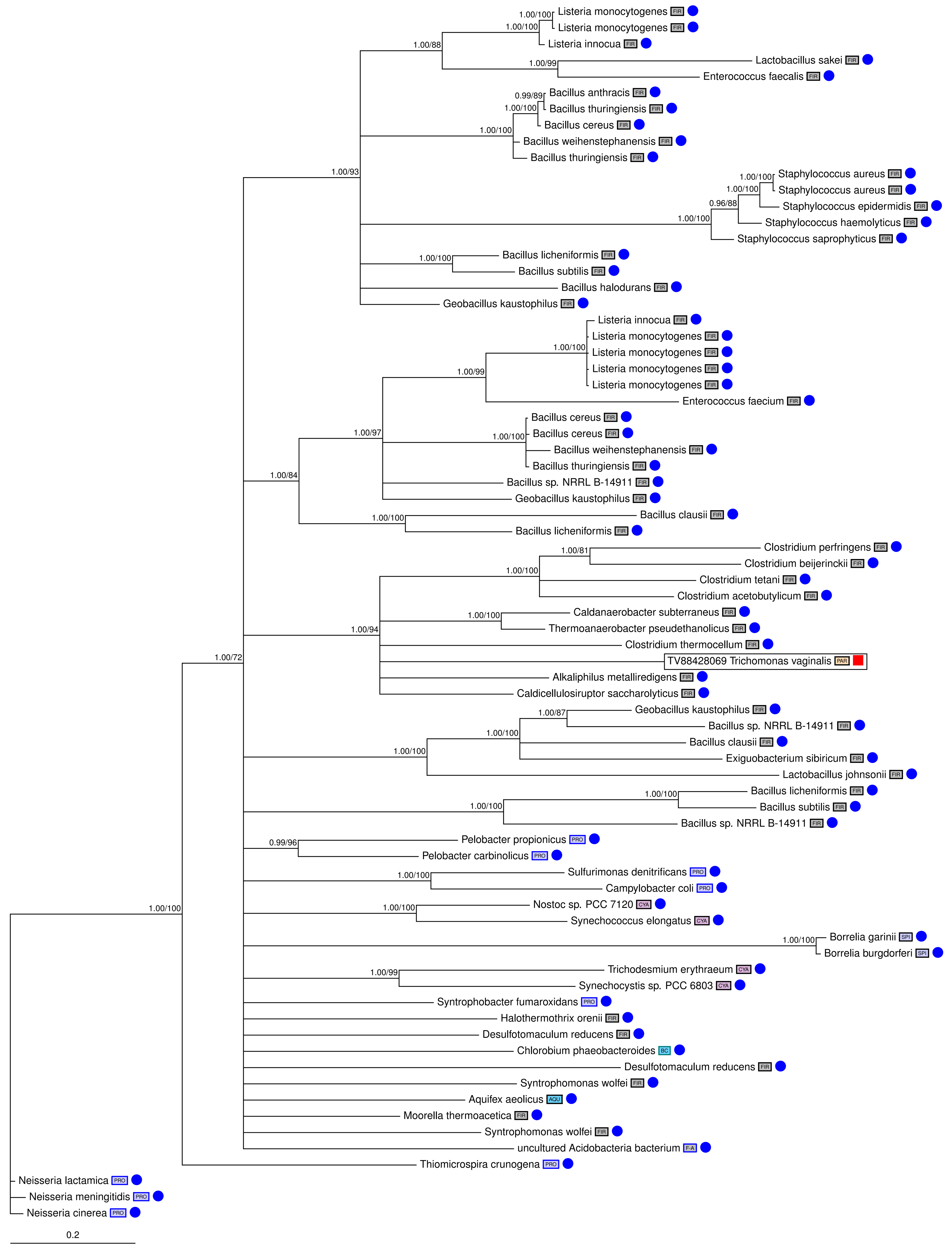

TN158

Candy accession: TV88428074
RefSeq accession: XP_001307946.1
Uniprot accession: A2FJA2_TRIVA
Comments: LGT - TV ONLY
Species affected: TV
Adjacent taxa in tree: Bacteria
EC annotation - (Blast/Profile): na
PHOBIUS SP: Y
PHOBIUS TMD: 8
RefSeq annotation: Auxin Efflux Carrier family protein
Name of enzyme/protein: Auxin Efflux Carrier family protein
KEGG PATHWAY - level 1: Other function - Membrane transport
KEGG PATHWAY - level 2: na

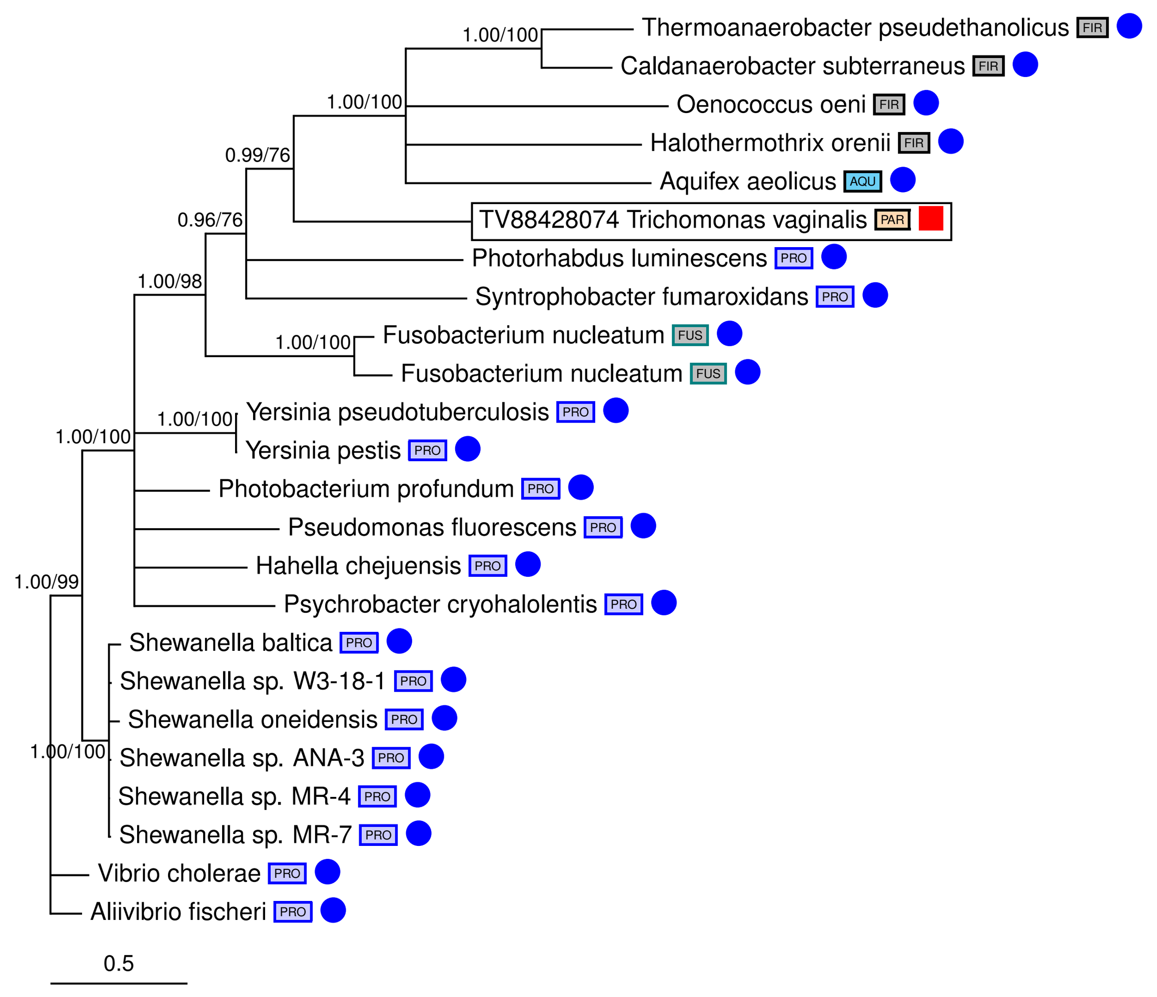

TN159

```
           Candy accession: Q54D94_DICDI
          RefSeq accession: XP_629602.1
         Uniprot accession: Q54D94_DICDI
                  Comments: LGT - DD TWO NODES
          Species affected: DD
      Adjacent taxa in tree: Prokaryotes
EC annotation - (Blast/Profile): na
                PHOBIUS SP: 0
               PHOBIUS TMD: 6
         RefSeq annotation: hypothetical protein DDB_G0292424
  Name of enzyme/protein: Predicted BioY family
  KEGG PATHWAY - level 1: Other function - Membrane transport
  KEGG PATHWAY - level 2: na
```

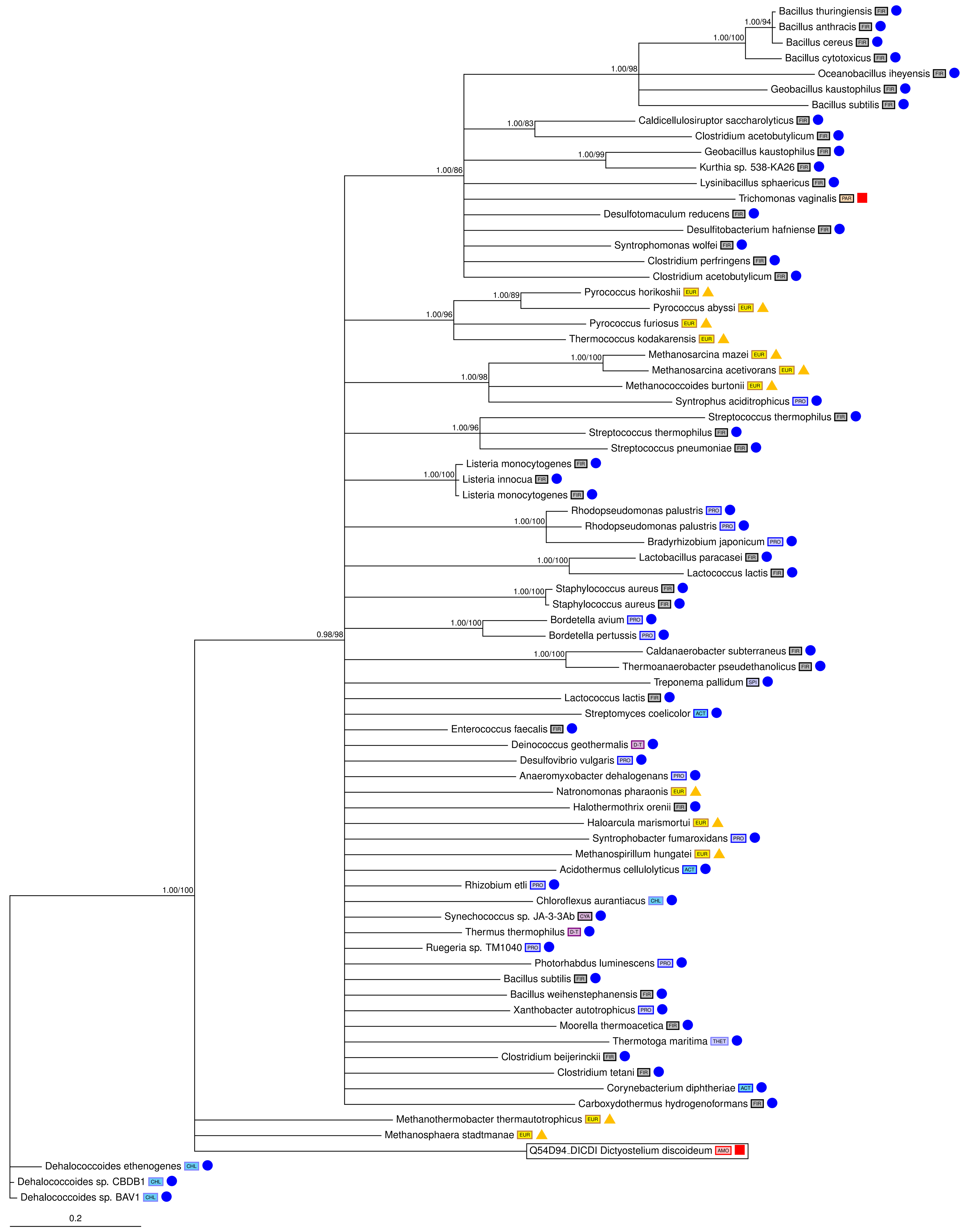

TN160

```
                Candy accession: TV88428079
               RefSeq accession: XP_001307937.1
              Uniprot accession: A2FJ93_TRIVA
                       Comments: LGT - TV ONLY
               Species affected: TV
          Adjacent taxa in tree: Firmicutes
EC annotation - (Blast/Profile): EC:3.6.3.25
                     PHOBIUS SP: 0
                    PHOBIUS TMD: 0
              RefSeq annotation: transporter
       Name of enzyme/protein: sulfate-transporting ATPase
         KEGG PATHWAY - level 1: Reaction
         KEGG PATHWAY - level 2: Reaction
```

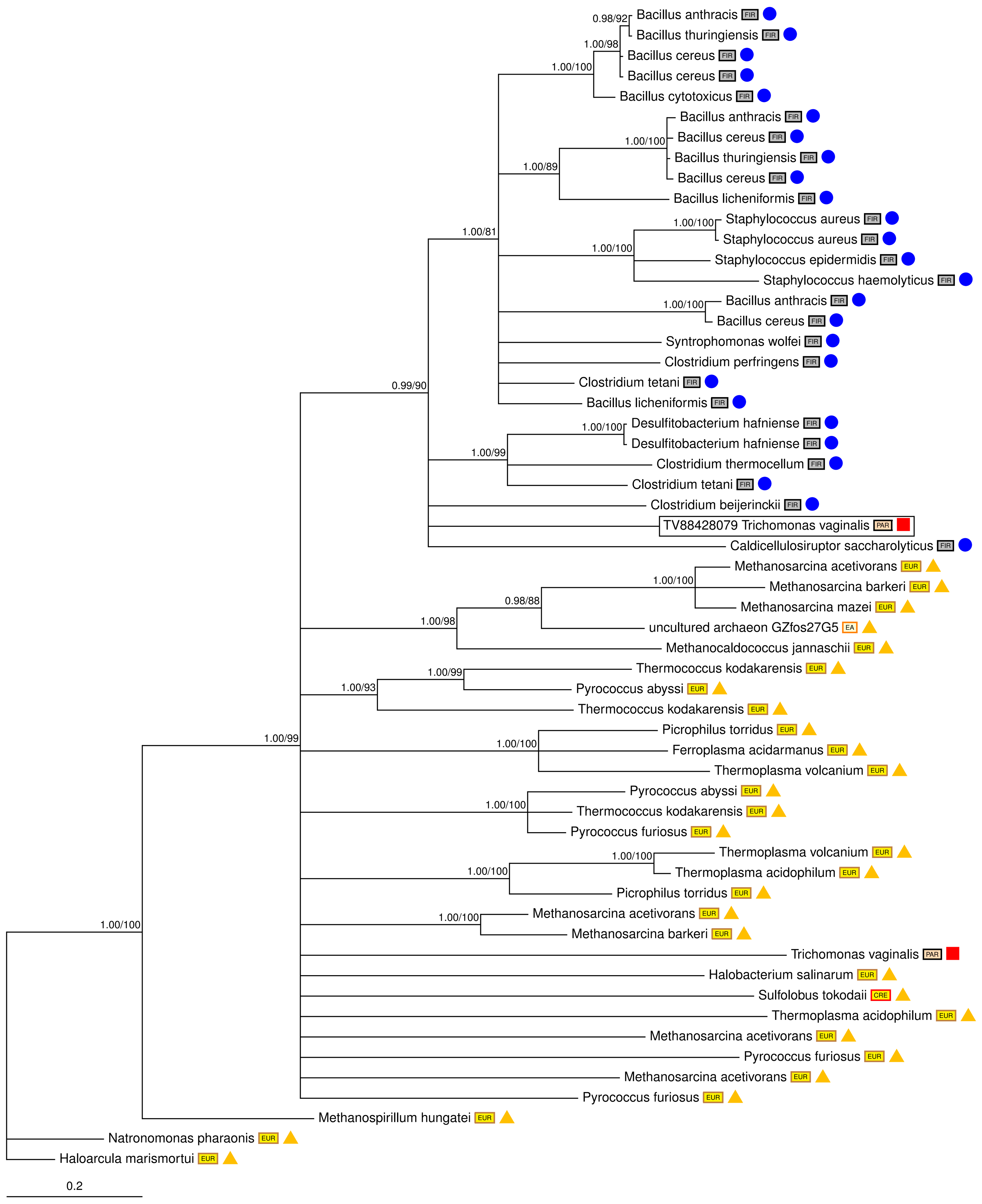

TN161

```
                Candy accession: TV89183132
              RefSeq accession: XP_001320621.1
             Uniprot accession: A2EG37_TRIVA
                      Comments: LGT - TV TWO NODES + FUNGI
              Species affected: TV,FUNGI
          Adjacent taxa in tree: Firmicutes - Thermoanaerobacter
EC annotation - (Blast/Profile): EC:3.2.1.23
                    PHOBIUS SP: 0
                   PHOBIUS TMD: 0
             RefSeq annotation: glycosyl hydrolase
        Name of enzyme/protein: beta-galactosidase
         KEGG PATHWAY - level 1: Carbohydrate Metabolism, Glycan
                                Biosynthesis and Metabolism,  Lipid
                                Metabolism
         KEGG PATHWAY - level 2: Galactose metabolism, Other glycan
                                degradation,  Glycosaminoglycan
                                degradation, Sphingolipid metabolism
```

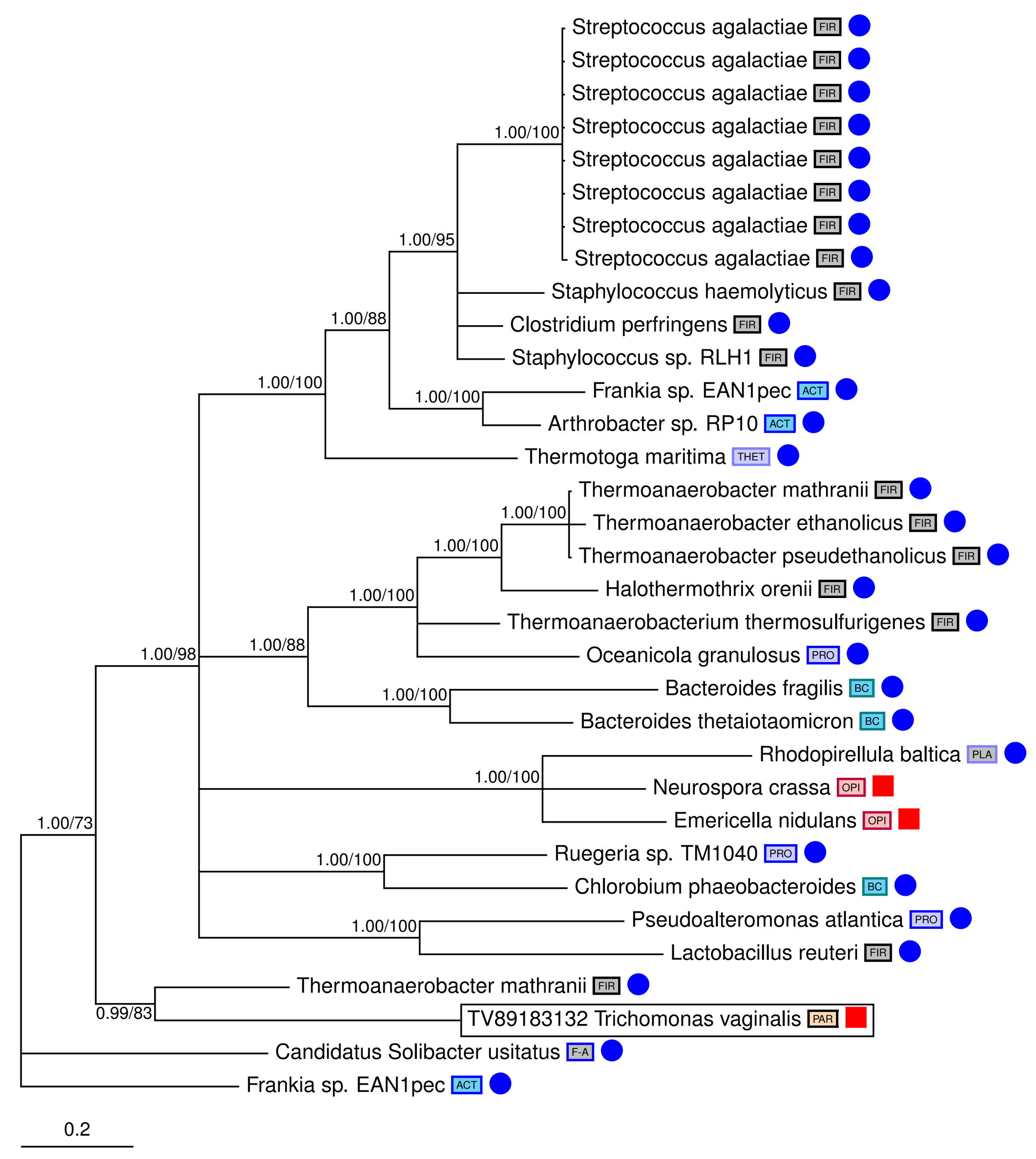

TN162

Candy accession: TV89272063
RefSeq accession: XP_001308218.1
Uniprot accession: A2FIH1_TRIVA
Comments: LGT - TV TWO NODES + ANIMALS
Species affected: TV
Adjacent taxa in tree: Bacteroidetes/Chlorobi - Bacteroides
EC annotation - (Blast/Profile): EC:5.1.3.8
PHOBIUS SP: 0
PHOBIUS TMD: 0
RefSeq annotation: renin binding protein
Name of enzyme/protein: N-acylglucosamine 2-epimerase
KEGG PATHWAY - level 1: Carbohydrate Metabolism
KEGG PATHWAY - level 2: Amino sugar and nucleotide sugar metabolism

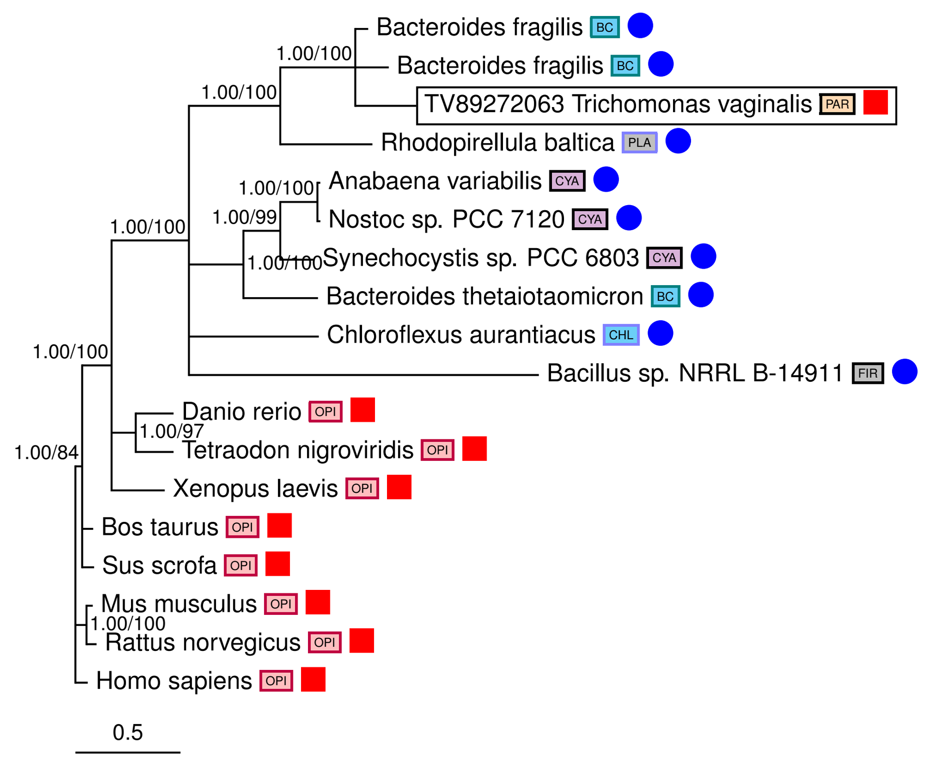

TN163

```
               Candy accession: TV89789068
              RefSeq accession: XP_001306707.1
             Uniprot accession: A2FMW0_TRIVA
                      Comments: LGT - TV TWO NODES + 2 FUNGI
              Species affected: TV,FUNGI
          Adjacent taxa in tree: Bacteria
EC annotation - (Blast/Profile): EC:4.1.1.44
                    PHOBIUS SP: 0
                   PHOBIUS TMD: 0
             RefSeq annotation: hypothetical protein
       Name of enzyme/protein: 4-carboxymuconolactone decarboxylase
        KEGG PATHWAY - level 1: Xenobiotics Biodegradation and
                                Metabolism
        KEGG PATHWAY - level 2: Benzoate degradation
```

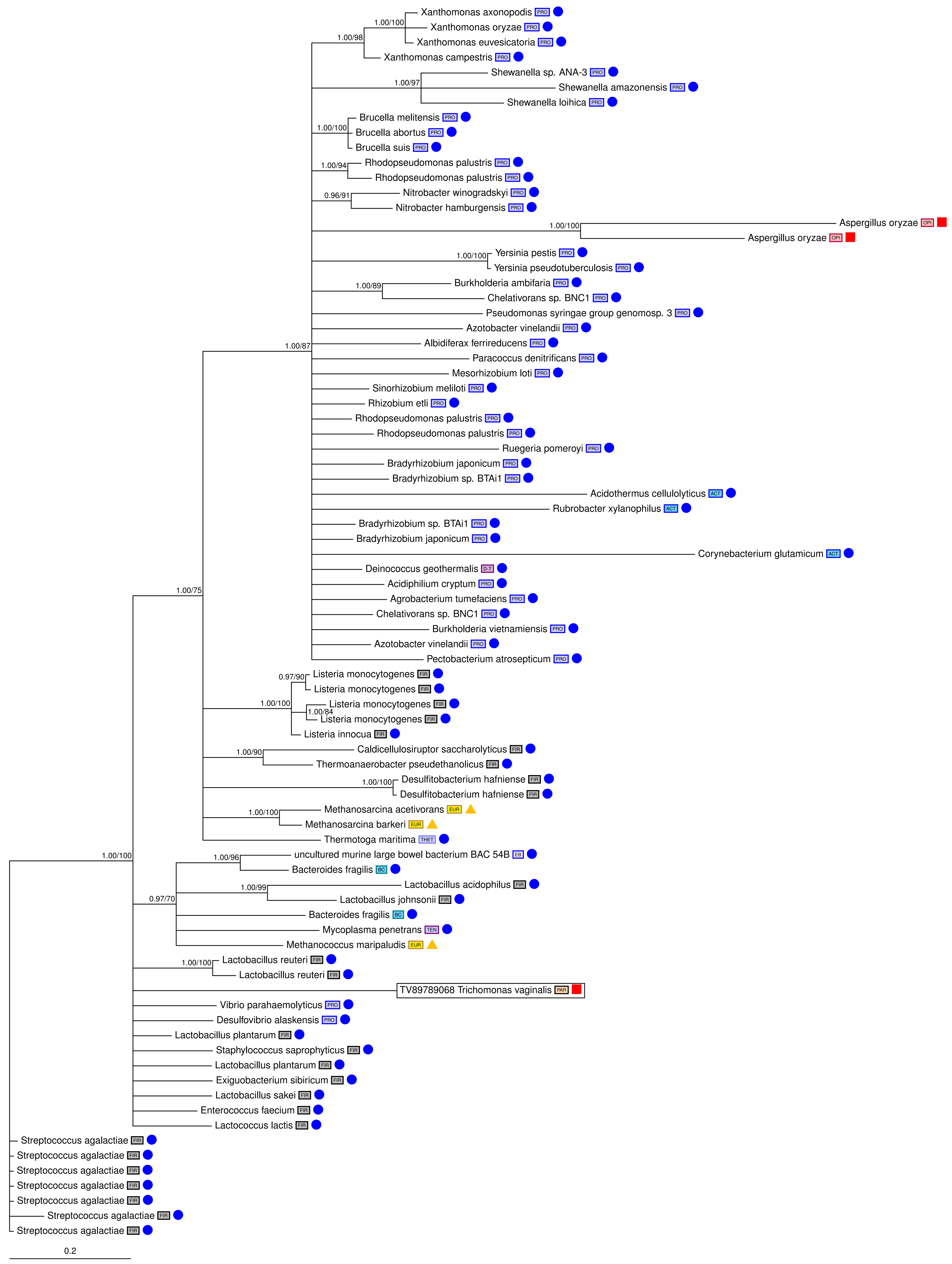

TN164

```
                 Candy accession: TV91346025
               RefSeq accession: XP_001299401.1
              Uniprot accession: A2G8P4_TRIVA
                       Comments: LGT - TV TWO NODES + FUNGI
               Species affected: TV,FUNGI
          Adjacent taxa in tree: Prokaryotes
EC annotation - (Blast/Profile): EC:6.1.1.19
                     PHOBIUS SP: 0
                    PHOBIUS TMD: 0
              RefSeq annotation: DALR anticodon binding domain containing
                                 protein
         Name of enzyme/protein: arginyl-tRNA synthetase
         KEGG PATHWAY - level 1: Translation - Genetic Information
                                 Processing
         KEGG PATHWAY - level 2: Aminoacyl-tRNA biosynthesis
```

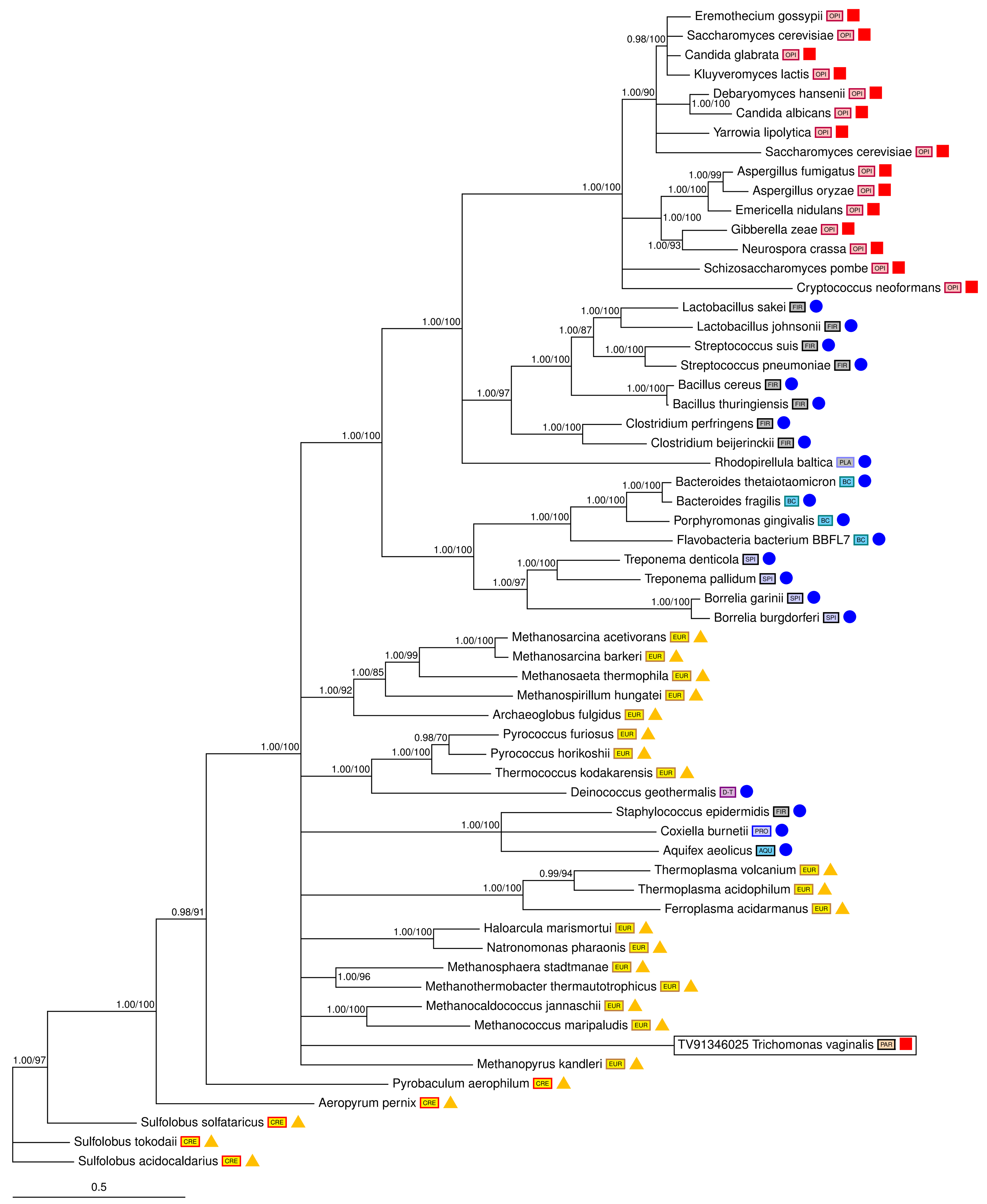

TN165

```
             Candy accession: TV91727152
            RefSeq accession: XP_001320207.1
           Uniprot accession: A2EHA5_TRIVA
                    Comments: LGT - TV TWO NODES
            Species affected: TV
       Adjacent taxa in tree: Archaea - Methanosphaera
EC annotation - (Blast/Profile): EC:1.1.1.157
                 PHOBIUS SP: 0
                PHOBIUS TMD: 0
          RefSeq annotation: 3-hydroxyacyl-CoA dehydrogenase,
                             C-terminal domain containing protein
       Name of enzyme/protein: 3-hydroxyacyl-CoA dehydrogenase
      KEGG PATHWAY - level 1: Xenobiotics Biodegradation and
                             Metabolism, Carbohydrate Metabolism
      KEGG PATHWAY - level 2: Benzoate degradation, Butanoate
                             metabolism
```

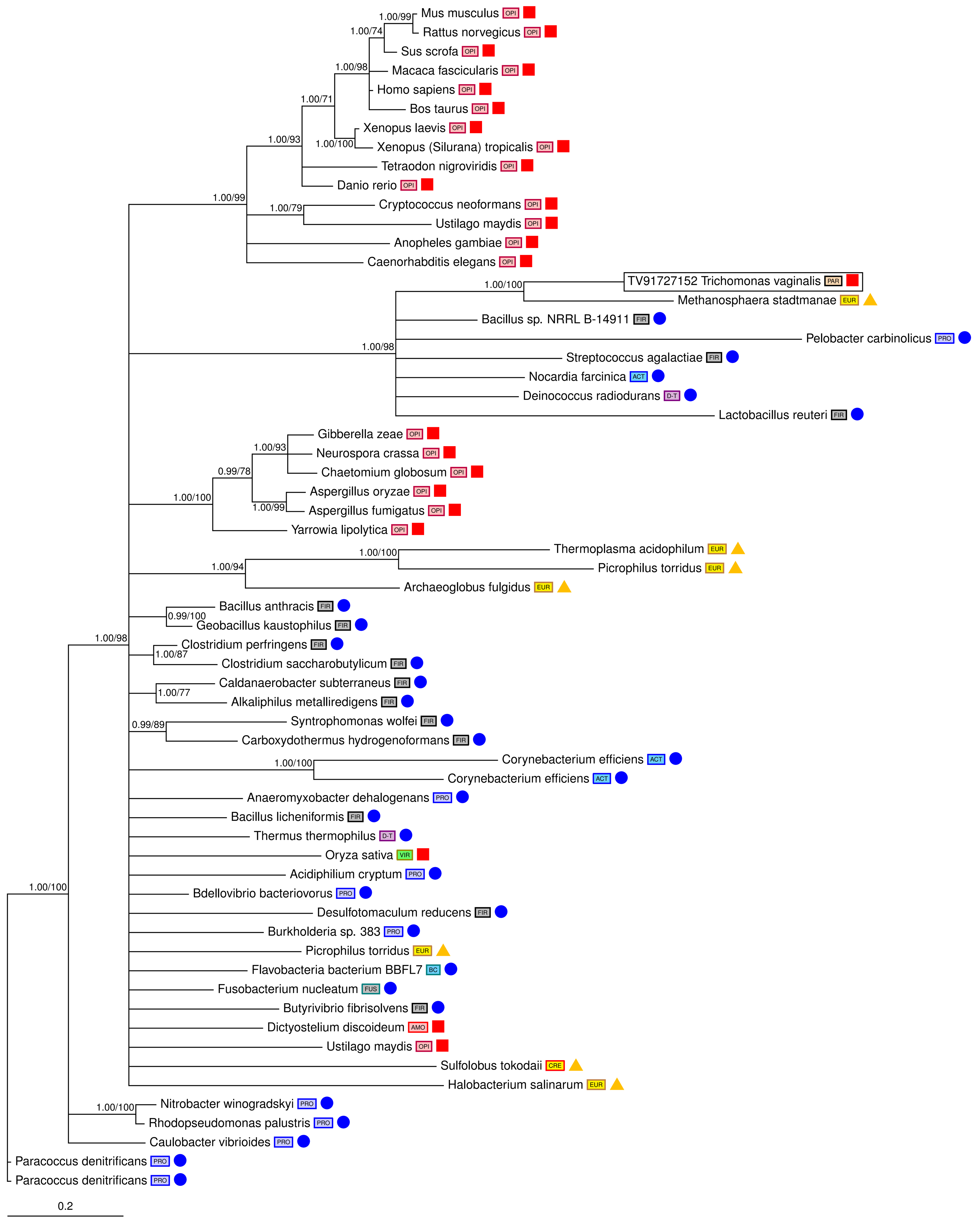

TN166

```
                Candy accession: TV92426055
               RefSeq accession: XP_001313452.1
              Uniprot accession: A2F3K0_TRIVA
                       Comments: LGT - TV ONLY
               Species affected: TV
          Adjacent taxa in tree: Protobacteria
EC annotation - (Blast/Profile): EC:3.4.13.18
                     PHOBIUS SP: 0
                    PHOBIUS TMD: 0
              RefSeq annotation: Clan PB, family C69, PipD-like cysteine
                                 peptidase
         Name of enzyme/protein: Cytosolic nonspecific dipeptidase
         KEGG PATHWAY - level 1: Amino Acid Metabolism, Metabolism of
                                 Other Amino Acids
         KEGG PATHWAY - level 2: Arginine and proline metabolism,
                                 Histidine metabolism, beta-Alanine
                                 metabolism
```

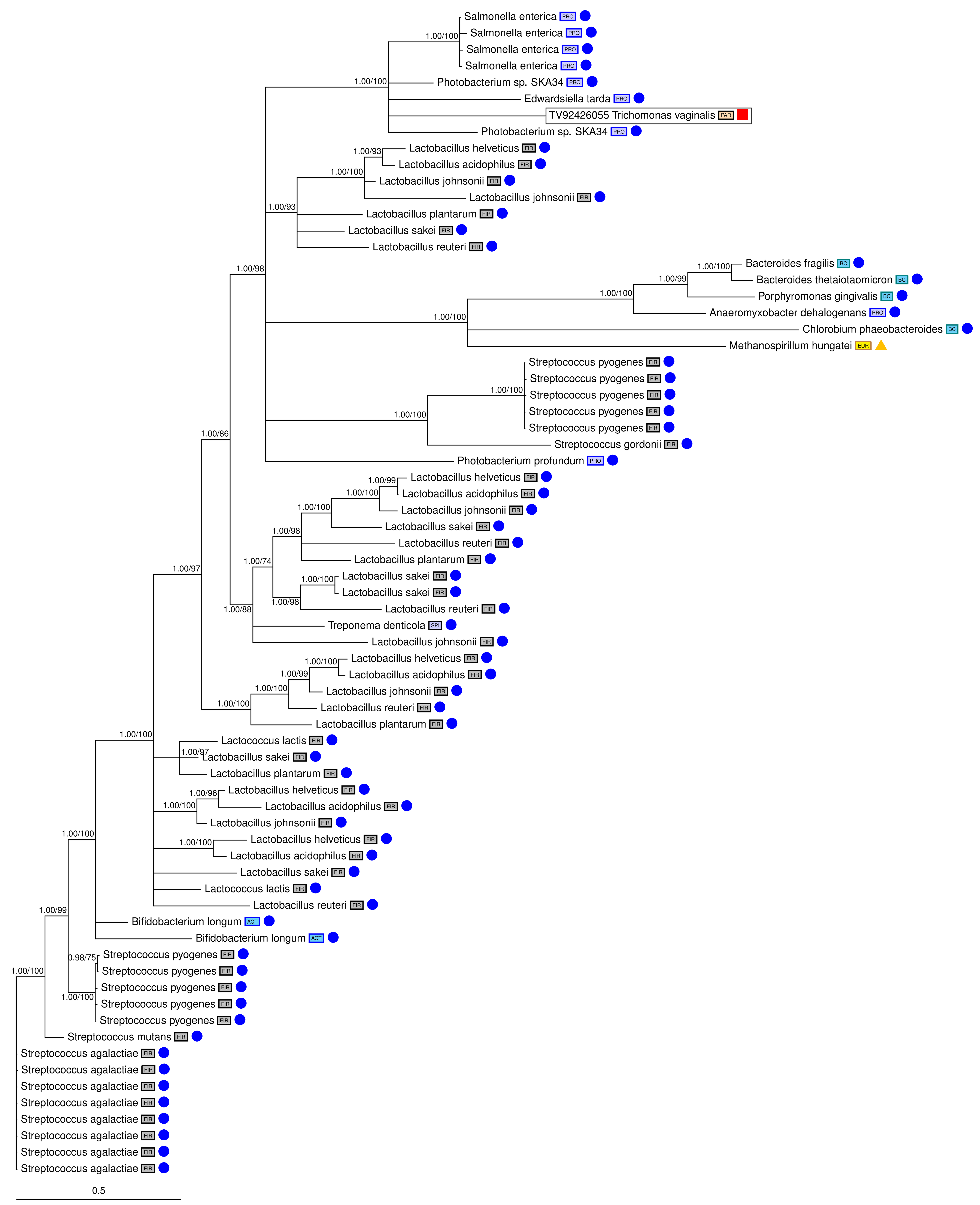

TN167

```
               Candy accession: TV92426060
              RefSeq accession: XP_001313458.1
             Uniprot accession: A2F3K6_TRIVA
                      Comments: LGT - TV ONLY
              Species affected: TV
        Adjacent taxa in tree: Prokaryotes
EC annotation - (Blast/Profile): EC:3.1.-.-
                    PHOBIUS SP: 0
                   PHOBIUS TMD: 0
             RefSeq annotation: metallo-beta-lactamase superfamily
                                protein
          Name of enzyme/protein: Predicted Zn-dependent hydrolase
        KEGG PATHWAY - level 1: Reaction
        KEGG PATHWAY - level 2: Reaction
```

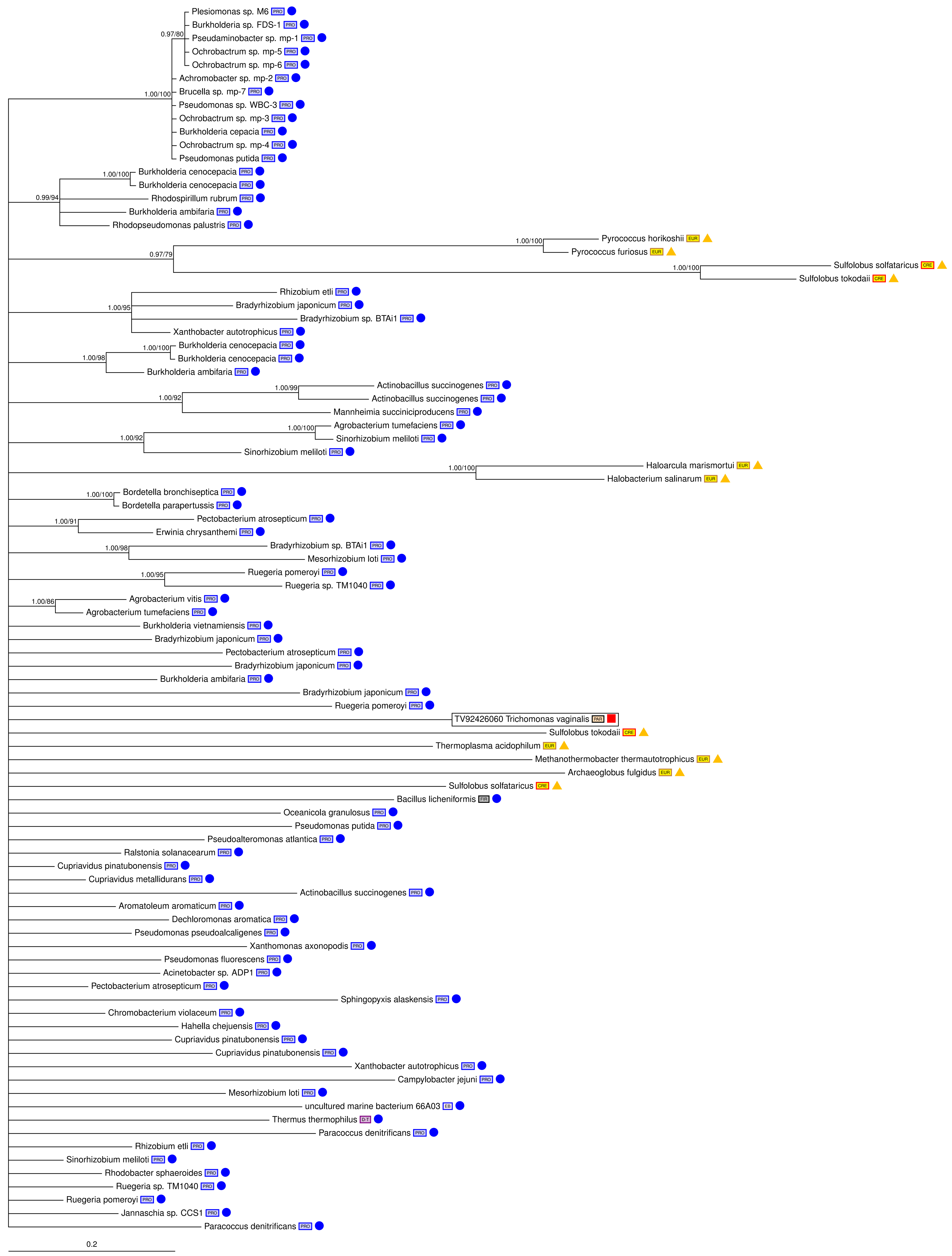

TN168

```
             Candy accession: TV92921294
           RefSeq accession: XP_001581038.1
          Uniprot accession: A2DHK4_TRIVA
                  Comments: LGT - TV TWO NODES + FUNGI
          Species affected: TV, FUNGI
     Adjacent taxa in tree: Bacteroidetes/Chlorobi - Bacteroides
EC annotation - (Blast/Profile): EC:3.2.1.23
               PHOBIUS SP: Y
              PHOBIUS TMD: 0
         RefSeq annotation: beta-galactosidase
   Name of enzyme/protein: Beta-galactosidase
   KEGG PATHWAY - level 1: Carbohydrate Metabolism, Glycan
                           Biosynthesis and Metabolism,  Lipid
                           Metabolism
   KEGG PATHWAY - level 2: Galactose metabolism, Other glycan
                           degradation,  Glycosaminoglycan
                           degradation, Sphingolipid metabolism
```

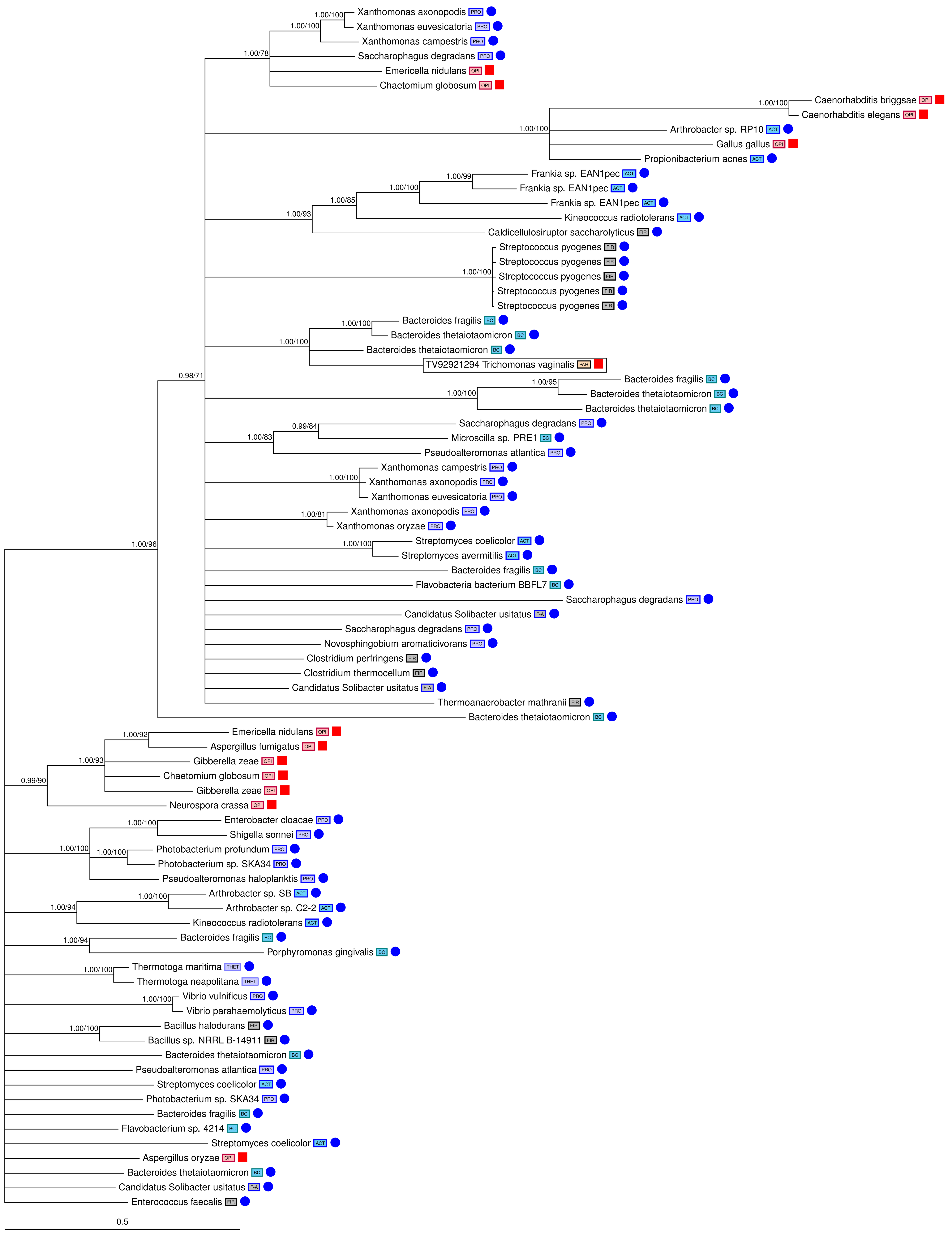

TN169

```
                Candy accession: TV90013073
               RefSeq accession: XP_001313313.1
              Uniprot accession: A2F3W8_TRIVA
                       Comments: LGT - TV TWO NODES + FUNGI
               Species affected: TV, FUNGI
          Adjacent taxa in tree: Proteobacteria - Francisella
EC annotation - (Blast/Profile): na
                     PHOBIUS SP: 0
                    PHOBIUS TMD: 0
              RefSeq annotation: beta lactamase
         Name of enzyme/protein: Predicted Zn-dependent hydrolases of the
                                 beta-lactamase
         KEGG PATHWAY - level 1: Other function
         KEGG PATHWAY - level 2: na
```

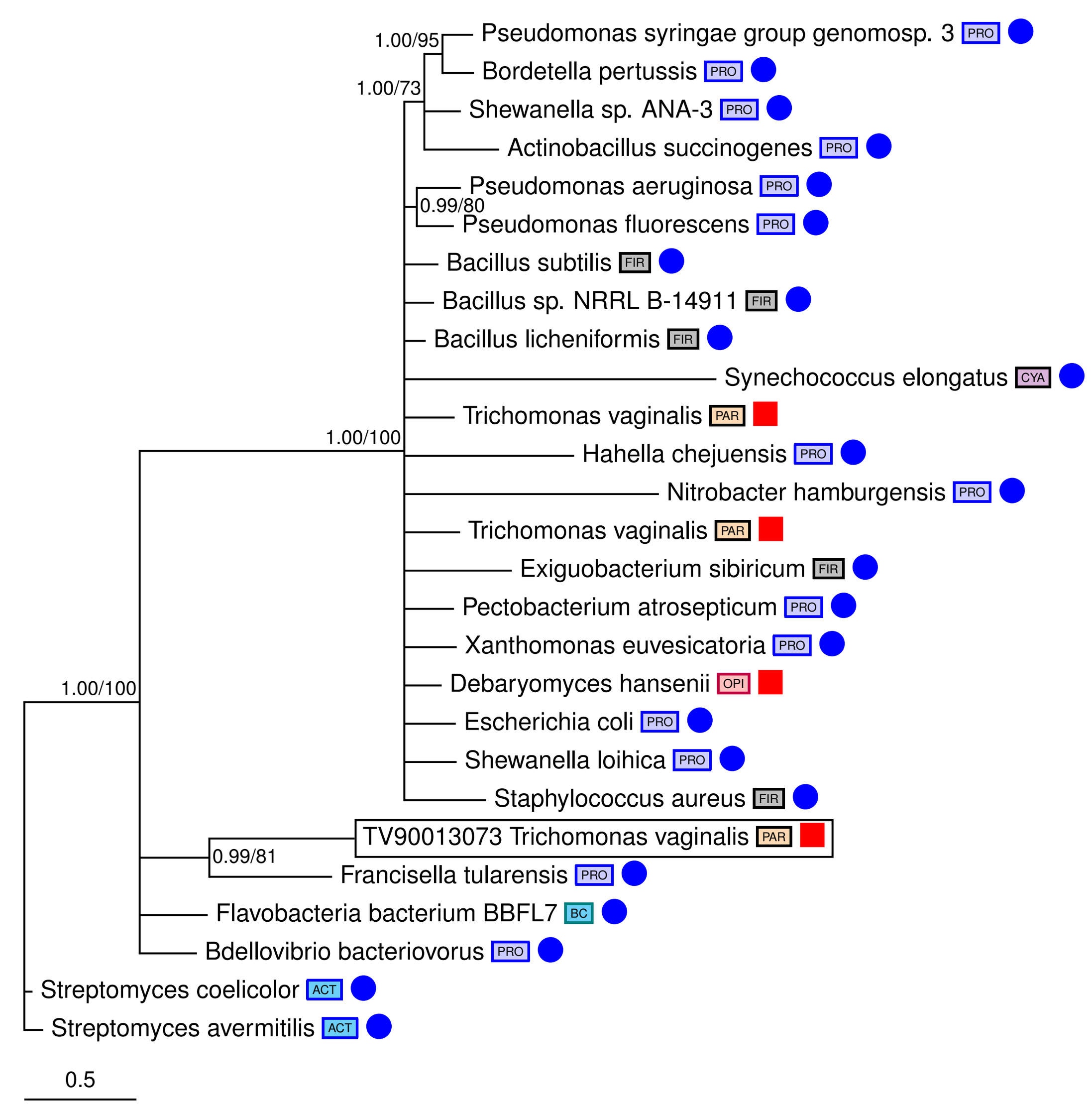

TN170

```
                Candy accession: TV94534133
               RefSeq accession: XP_001321964.1
              Uniprot accession: A2EC64_TRIVA
                       Comments: LGT - TV TWO NODES
               Species affected: TV
           Adjacent taxa in tree: Firmicutes - Clostridia
EC annotation - (Blast/Profile): na
                     PHOBIUS SP: 0
                    PHOBIUS TMD: 0
              RefSeq annotation: hydrolase, NUDIX family protein
        Name of enzyme/protein: Predicted hydrolase, NUDIX family
                                protein
         KEGG PATHWAY - level 1: Other function
         KEGG PATHWAY - level 2: na
```

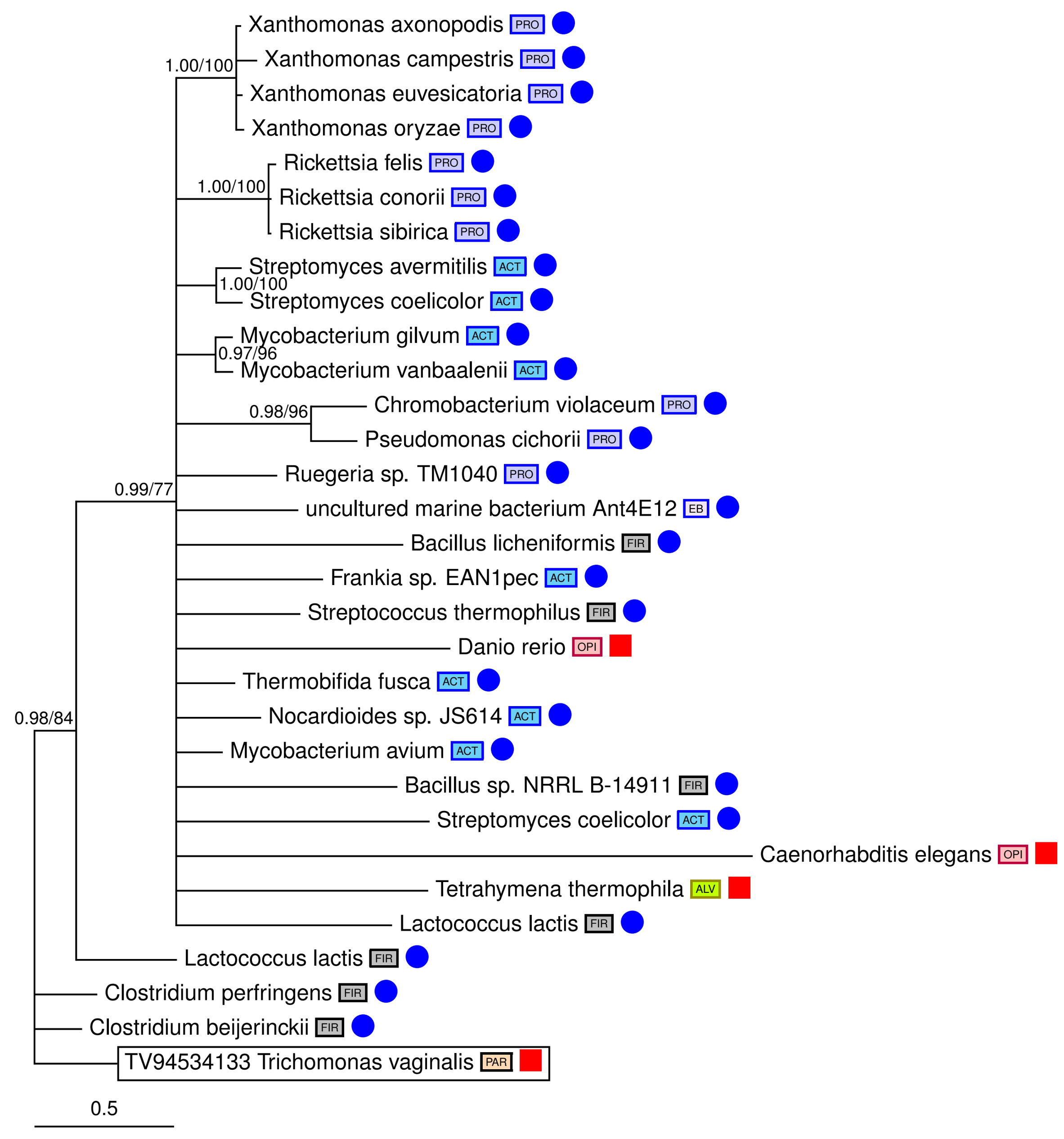

TN171

```
                 Candy accession: TV95301090
                RefSeq accession: XP_001311981.1
               Uniprot accession: A2F7S4_TRIVA
                        Comments: LGT - TV NICE TREE
                Species affected: TV
            Adjacent taxa in tree: Bacteria
EC annotation - (Blast/Profile): EC:3.5.1.18
                      PHOBIUS SP: 0
                     PHOBIUS TMD: 0
               RefSeq annotation: Clan MH, family M20, peptidase T-like
                                  metallopeptidase
         Name of enzyme/protein: succinyl-diaminopimelate desuccinylase
          KEGG PATHWAY - level 1: Amino Acid Metabolism
          KEGG PATHWAY - level 2: Lysine biosynthesis

                 Candy accession: Q50Y52_ENTHI
                RefSeq accession: XP_651923.2
               Uniprot accession: Q50Y52_ENTHI
                        Comments: LGT - EH NICE TREE
                Species affected: TV
            Adjacent taxa in tree: Prokaryotes
EC annotation - (Blast/Profile): EC:3.5.1.18
                      PHOBIUS SP: 0
                     PHOBIUS TMD: 0
               RefSeq annotation: hypothetical protein
         Name of enzyme/protein: succinyl-diaminopimelate desuccinylase
          KEGG PATHWAY - level 1: Amino Acid Metabolism
          KEGG PATHWAY - level 2: Lysine biosynthesis
```

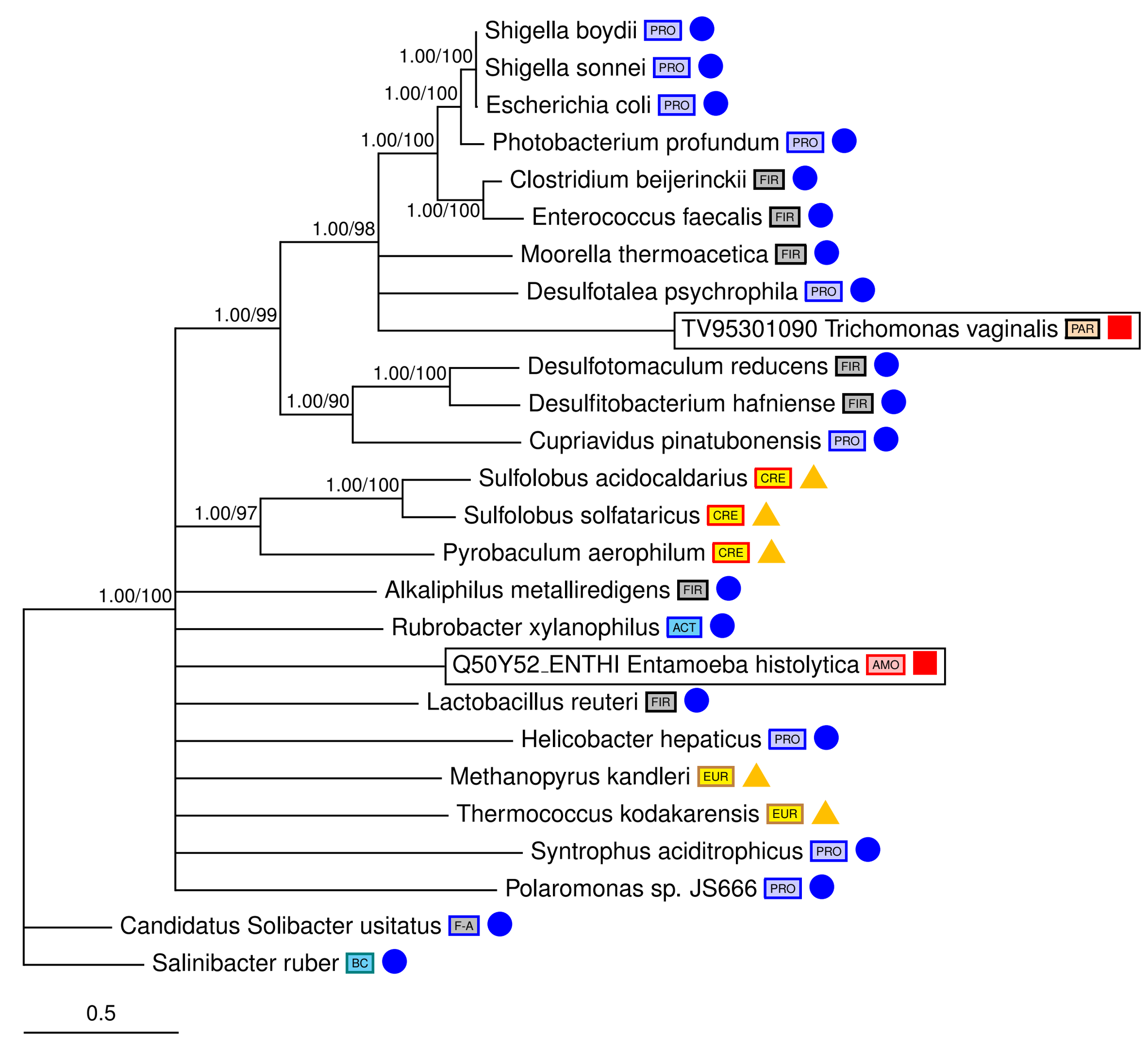

TN172

```
              Candy accession: TV95601040
             RefSeq accession: XP_001302065.1
            Uniprot accession: A2G132_TRIVA
                     Comments: LGT - TV TWO NODES + FUNGI
             Species affected: TV,FUNGI
        Adjacent taxa in tree: Actinobacteria - Corynebacterium
EC annotation - (Blast/Profile): na
                   PHOBIUS SP: 0
                  PHOBIUS TMD: 0
           RefSeq annotation: hypothetical protein
       Name of enzyme/protein: Hypothetical protein
       KEGG PATHWAY - level 1: Function unknown
       KEGG PATHWAY - level 2: na
```

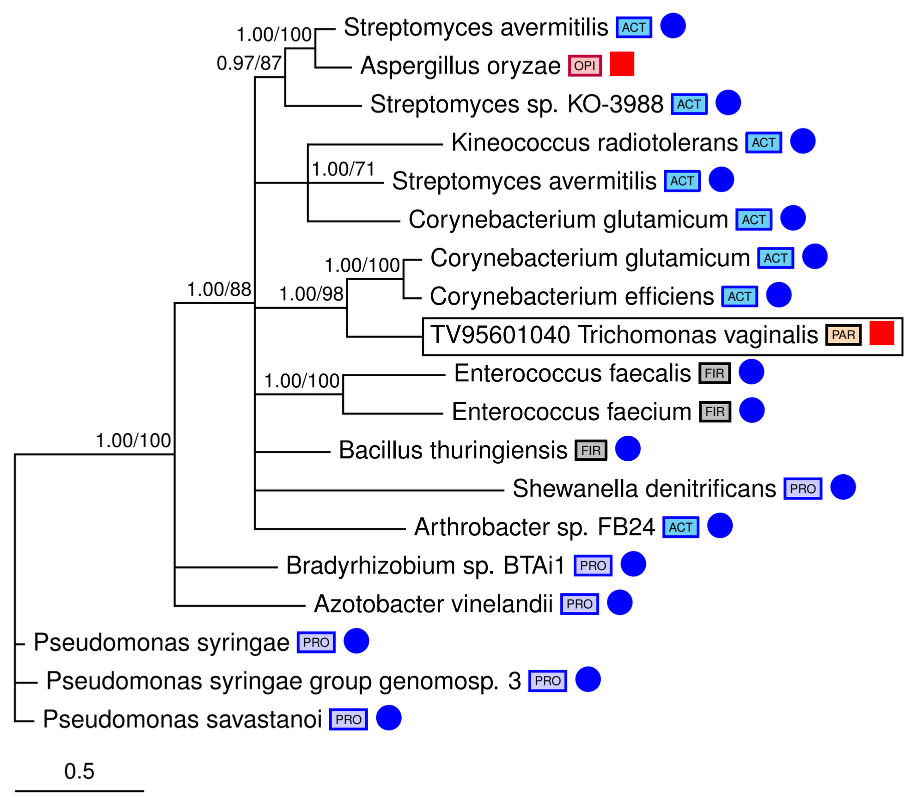

TN173

```
                  Candy accession: TV95894101
                 RefSeq accession: XP_001310032.1
                Uniprot accession: A2FDA0_TRIVA
                         Comments: LGT - TV TWO NODES
                 Species affected: TV
           Adjacent taxa in tree: Bacteria
EC annotation - (Blast/Profile): EC:3.2.1.52
                      PHOBIUS SP: 0
                     PHOBIUS TMD: 1
                RefSeq annotation: glycosyl hydrolase
          Name of enzyme/protein: beta-N-acetylhexosaminidase
          KEGG PATHWAY - level 1: Glycan Biosynthesis and Metabolism,
                                  Carbohydrate Metabolism
          KEGG PATHWAY - level 2: Other glycan degradation,  Various types
                                  of N-glycan biosynthesis,
                                  Glycosaminoglycan degradation, Amino
                                  sugar and nucleotide sugar metabolism
```

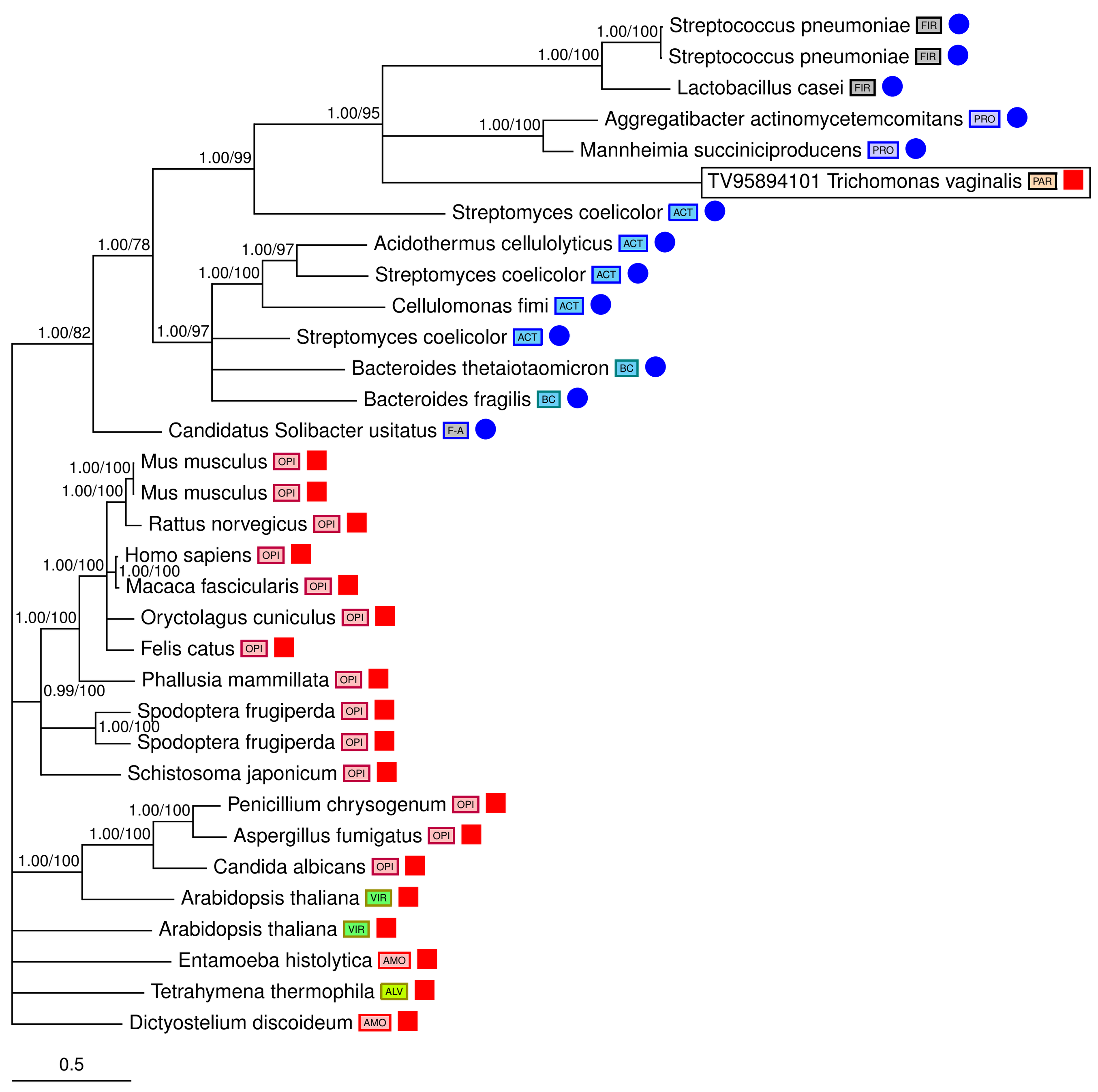

TN174

Candy accession:	TV96353085
RefSeq accession:	XP_001307242.1
Uniprot accession:	A2FL96_TRIVA
Comments:	LGT - TV TWO NODES
Species affected:	TV
Adjacent taxa in tree:	Bacteroidetes/Chlorobi - Chlorobium
EC annotation - (Blast/Profile):	na
PHOBIUS SP:	0
PHOBIUS TMD:	0
RefSeq annotation:	hypothetical protein
Name of enzyme/protein:	Protein containing cysteine synthase domain
KEGG PATHWAY - level 1:	Function unknown
KEGG PATHWAY - level 2:	na

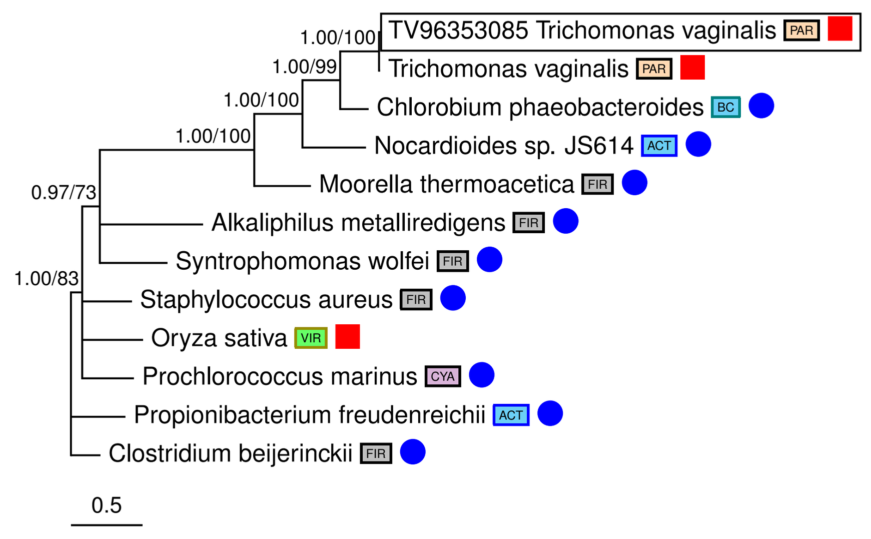

TN175

```
                  Candy accession: O15804_PLAFA
                 RefSeq accession: XP_001350162.1
                Uniprot accession: O15804_PLAFA
                         Comments: LGT - PLASMODIUM
                 Species affected: PF,PV,PY
            Adjacent taxa in tree: Prokaryotes
EC annotation - (Blast/Profile): EC:2.1.3.3
                      PHOBIUS SP: 0
                     PHOBIUS TMD: 1
                RefSeq annotation: aspartate carbamoyltransferase
          Name of enzyme/protein: ornithine carbamoyltransferase
          KEGG PATHWAY - level 1: Amino Acid Metabolism
          KEGG PATHWAY - level 2: Arginine and proline metabolism

                  Candy accession: TV112459013
                 RefSeq accession: XP_001298740.1
                Uniprot accession: A2GAK6_TRIVA
                         Comments: LGT - TV
                 Species affected: TV
            Adjacent taxa in tree: Bacteria
EC annotation - (Blast/Profile): EC:2.1.3.3
                      PHOBIUS SP: 0
                     PHOBIUS TMD: 0
                RefSeq annotation: Aspartate/ornithine
                                   carbamoyltransferase, carbamoyl-P
                                   binding domain containing protein
          Name of enzyme/protein: ornithine carbamoyltransferase
          KEGG PATHWAY - level 1: Amino Acid Metabolism
          KEGG PATHWAY - level 2: Arginine and proline metabolism
```

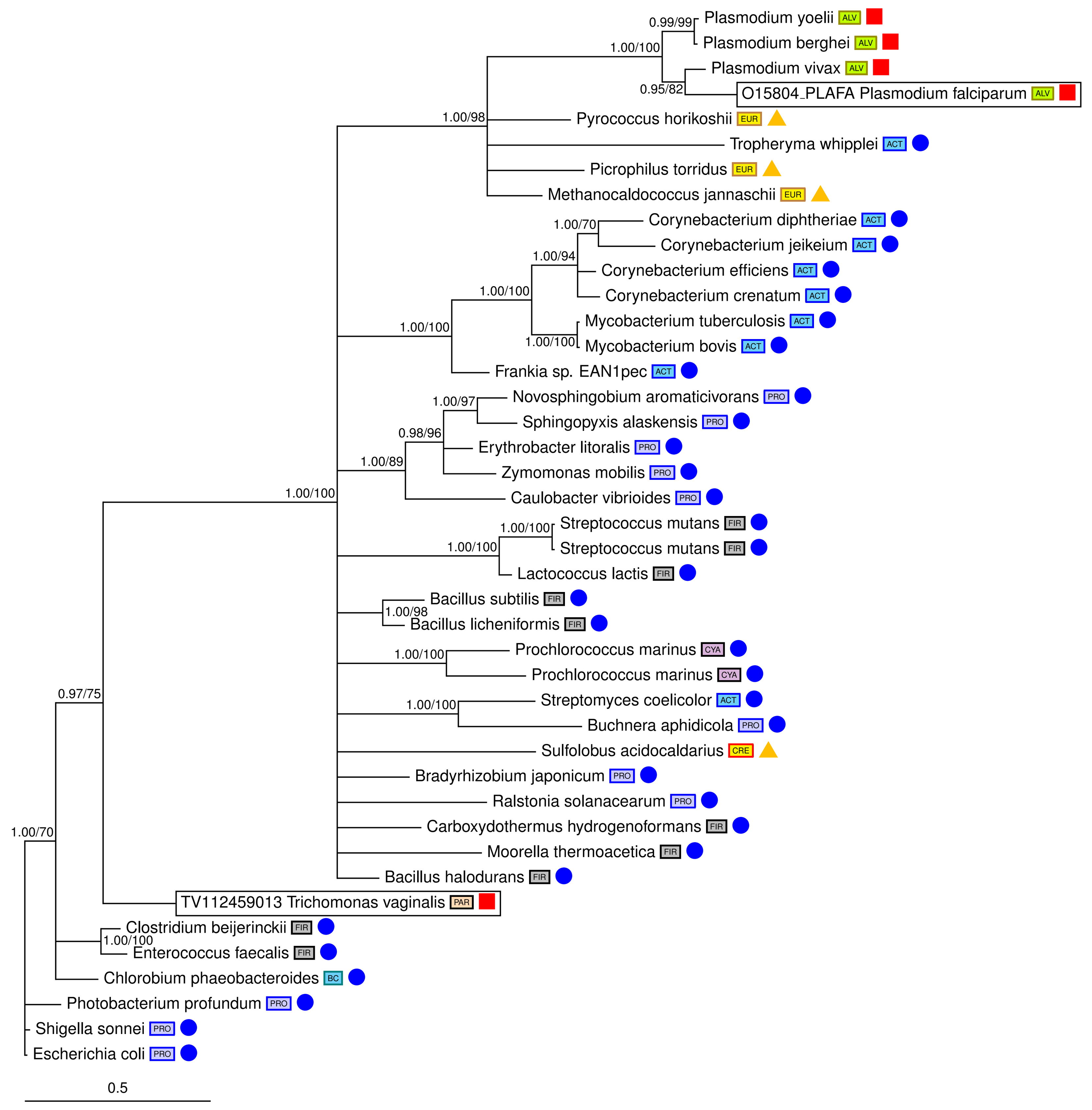

TN176

```
                Candy accession: Q7RJ87_PLAYO
               RefSeq accession: XP_731378.1
              Uniprot accession: Q7RJ87_PLAYO
                       Comments: LGT - DEAP APICOMPLEXA LGT+DUPLICATIONS
                                 + A GREEN ALGAE
               Species affected: PF, PV,PY,TG,CP
           Adjacent taxa in tree: Bacteria
EC annotation - (Blast/Profile): EC:1.1.1.37
                    PHOBIUS SP: 0
                   PHOBIUS TMD: 1
              RefSeq annotation: malate dehydrogenase
         Name of enzyme/protein: malate dehydrogenase
         KEGG PATHWAY - level 1: Carbohydrate Metabolism
         KEGG PATHWAY - level 2: Citrate cycle (TCA cycle), Pyruvate
                                 metabolism, Glyoxylate and dicarboxylate
                                 metabolism
```

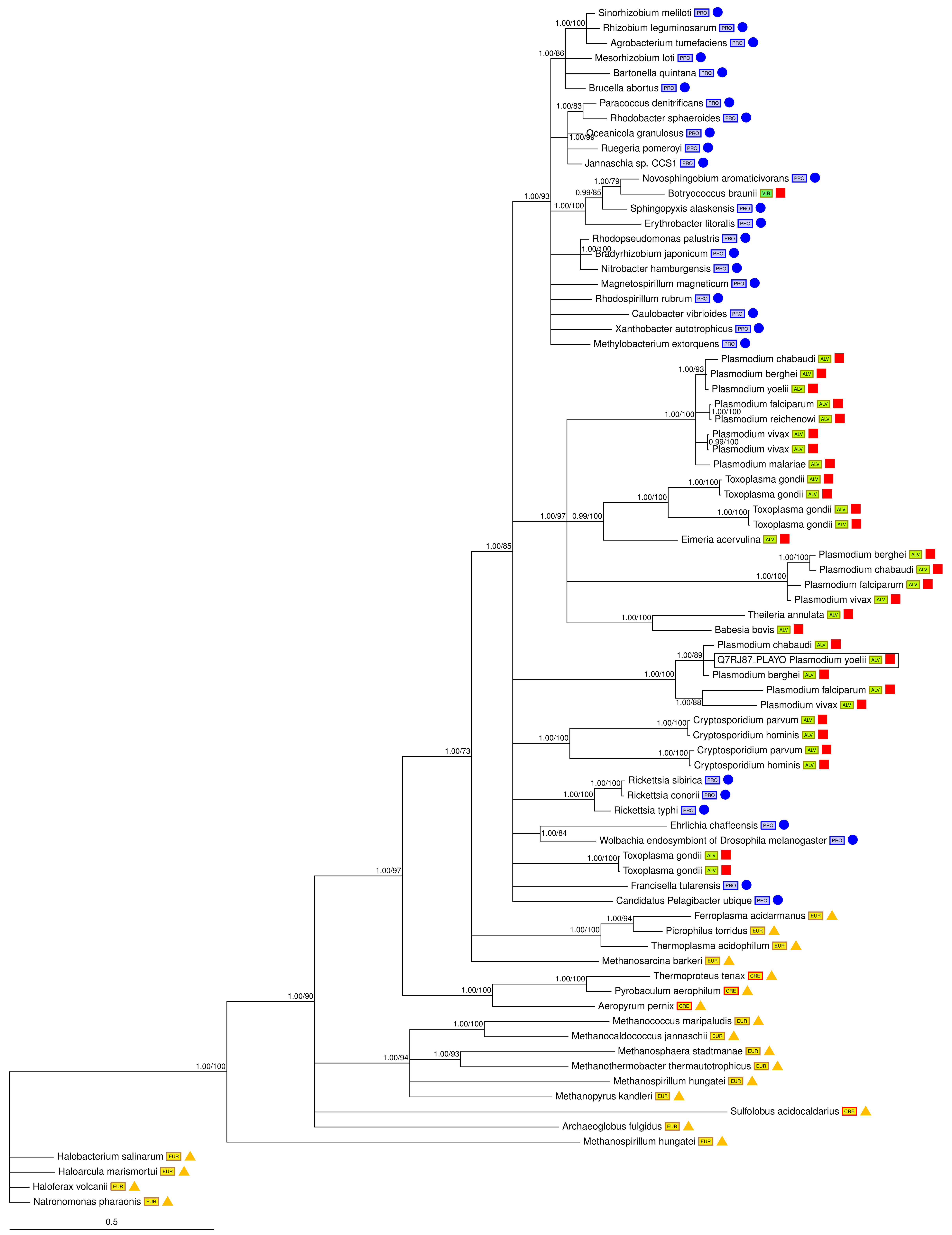

TN177

Candy accession: Q4Q2Q9_LEIMA
RefSeq accession: XP_001686389.1
Uniprot accession: Q4Q2Q9_LEIMA
Comments: LGT - KINETOPLASTIDS TWO NODES
Species affected: LM,TC
Adjacent taxa in tree: Bacteria
EC annotation - (Blast/Profile): EC:2.3.1.30
PHOBIUS SP: 0
PHOBIUS TMD: 0
RefSeq annotation: serine acetyltransferase
Name of enzyme/protein: Serine O-acetyltransferase
KEGG PATHWAY - level 1: Amino Acid Metabolism, Energy Metabolism
KEGG PATHWAY - level 2: Cysteine and methionine metabolism,
Sulfur metabolism

Candy accession: Q51DT0_ENTHI
RefSeq accession: XP_656373.1
Uniprot accession: Q401L4_ENTHI
Comments: LGT - EH,ED TWO NODES
Species affected: EH,ED
Adjacent taxa in tree: Archaea - Haloarcula
EC annotation - (Blast/Profile): EC:2.3.1.30
PHOBIUS SP: 0
PHOBIUS TMD: 0
RefSeq annotation: serine acetyltransferase 1
Name of enzyme/protein: Serine O-acetyltransferase
KEGG PATHWAY - level 1: Amino Acid Metabolism, Energy Metabolism
KEGG PATHWAY - level 2: Cysteine and methionine metabolism,
Sulfur metabolism

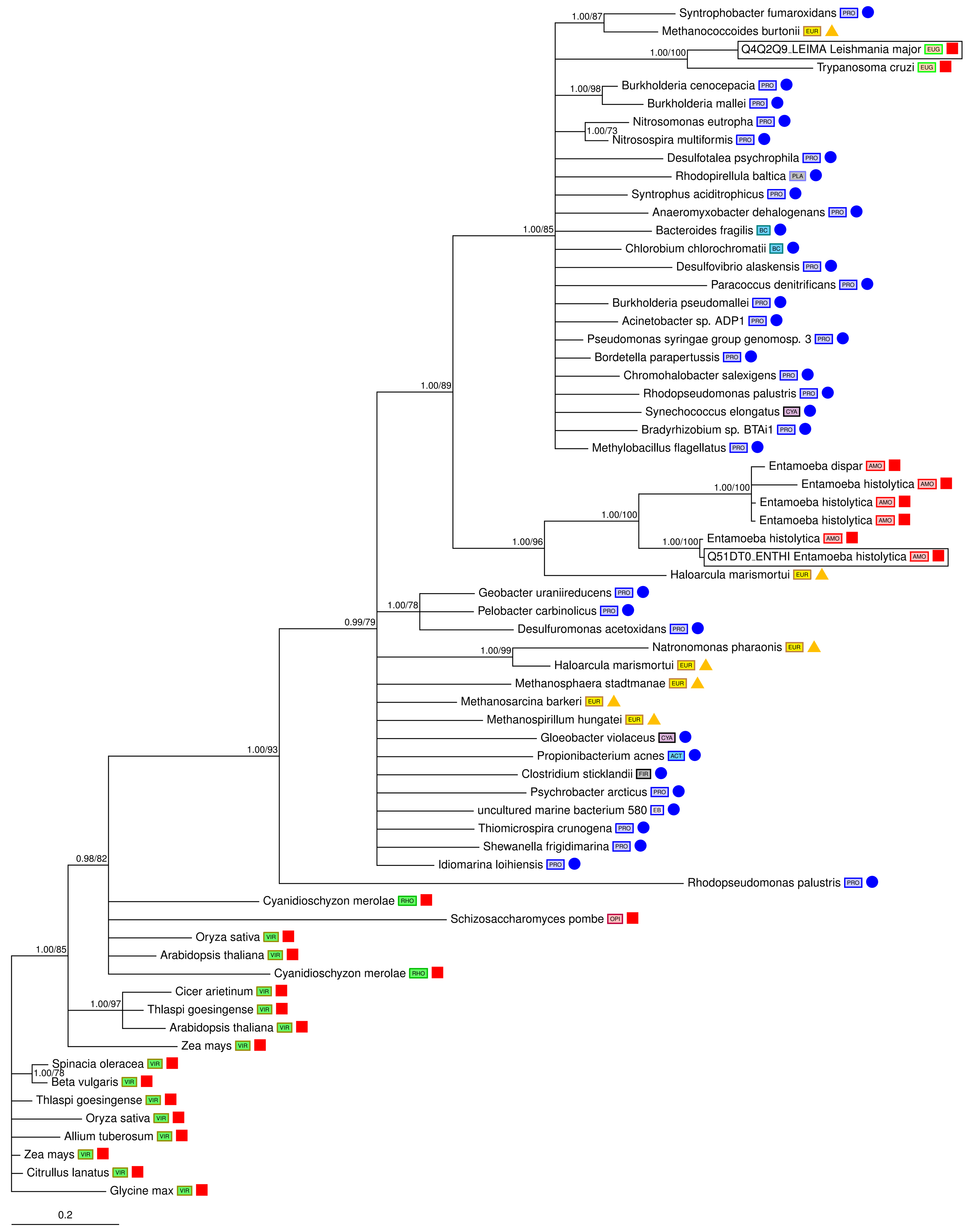

TN178

Candy accession: TV97324255
RefSeq accession: XP_001329597.1
Uniprot accession: A2DQ35_TRIVA
Comments: LGT - TV TWO NODES - 3 DOMAIN ToL
Species affected: TV
Adjacent taxa in tree: Proteobacteria
EC annotation - (Blast/Profile): EC:1.1.1.42
PHOBIUS SP: 0
PHOBIUS TMD: 0
RefSeq annotation: isocitrate dehydrogenase
Name of enzyme/protein: Isocitrate dehydrogenase [NADP]
KEGG PATHWAY - level 1: Carbohydrate Metabolism, Amino Acid metabolism
KEGG PATHWAY - level 2: Citrate cycle (TCA cycle), Glutathione metabolism

Candy accession: Q4Q3T2_LEIMA
RefSeq accession: XP_001686016.1
Uniprot accession: Q4Q3T2_LEIMA
Comments: LGT - TV TWO NODES - 3 DOMAIN ToL
Species affected: LM
Adjacent taxa in tree: Proteobacteria - Zymomonas
EC annotation - (Blast/Profile): EC:1.1.1.42
PHOBIUS SP: 0
PHOBIUS TMD: 0
RefSeq annotation: isocitrate dehydrogenase
Name of enzyme/protein: Isocitrate dehydrogenase [NADP]
KEGG PATHWAY - level 1: Carbohydrate Metabolism, Amino Acid metabolism
KEGG PATHWAY - level 2: Citrate cycle (TCA cycle), Glutathione metabolism

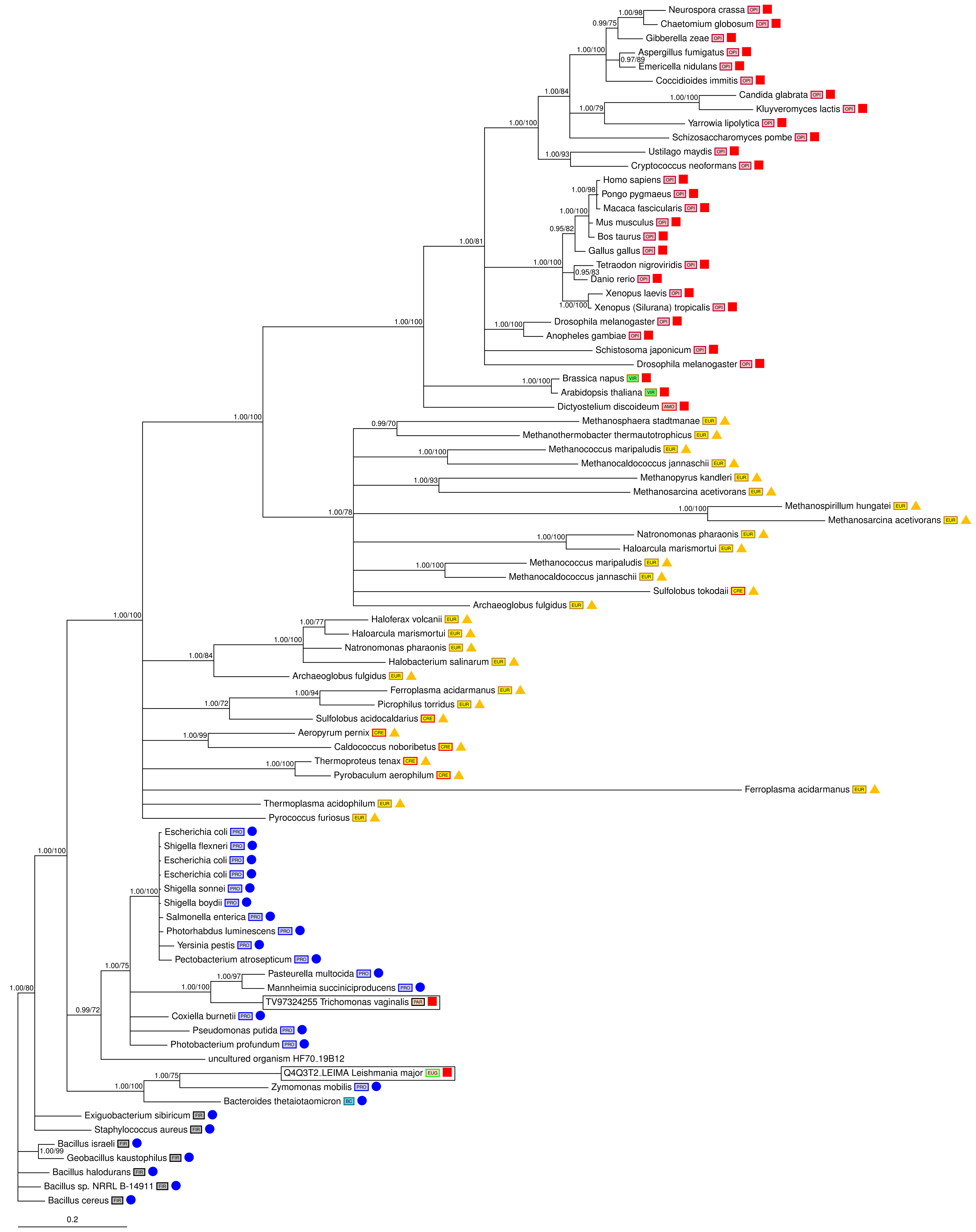

TN179

```
              Candy accession: Q4Q5Z6_LEIMA
             RefSeq accession: XP_001685252.1
            Uniprot accession: Q4Q5Z6_LEIMA
                     Comments: LGT - KINETOPLASTIDS TWO NODES
             Species affected: LM,TB,TC
         Adjacent taxa in tree: Proteobacteria - Desulfuromonas
EC annotation - (Blast/Profile): EC:1.8.1.4
                   PHOBIUS SP: 0
                  PHOBIUS TMD: 0
            RefSeq annotation: acetoin dehydrogenase e3 component-like
                               protein
         Name of enzyme/protein: dihydrolipoyl dehydrogenase
         KEGG PATHWAY - level 1: Carbohydrate Metabolism, Amino Acid
                               metabolism
         KEGG PATHWAY - level 2: Glycolysis / Gluconeogenesis, Citrate
                               cycle (TCA cycle), Pyruvate metabolism,
                               Glycine, serine and threonine
                               metabolism, Valine, leucine and
                               isoleucine degradation
```

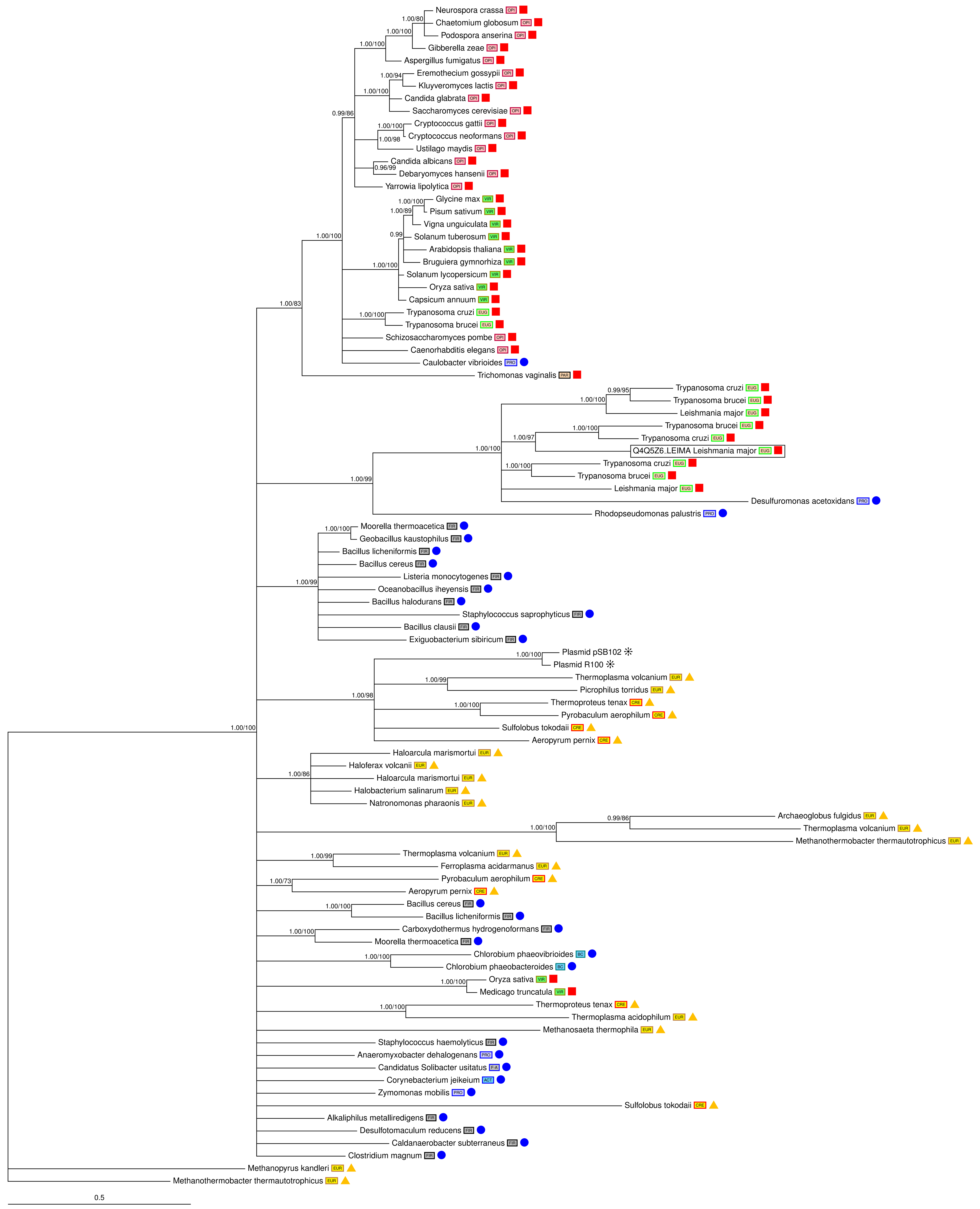

TN180

```
                Candy accession: Q54UX0_DICDI
               RefSeq accession: XP_641017.1
              Uniprot accession: Q54UX0_DICDI
                       Comments: LGT - DD TWO NODES
                Species affected: DD
             Adjacent taxa in tree: Proteobacteria
EC annotation - (Blast/Profile): EC:3.5.1.14
                     PHOBIUS SP: 0
                    PHOBIUS TMD: 0
              RefSeq annotation: hypothetical protein DDB_G0280767
          Name of enzyme/protein: aminoacylase
          KEGG PATHWAY - level 1: Amino Acid Metabolism
          KEGG PATHWAY - level 2: Arginine and proline metabolism
```

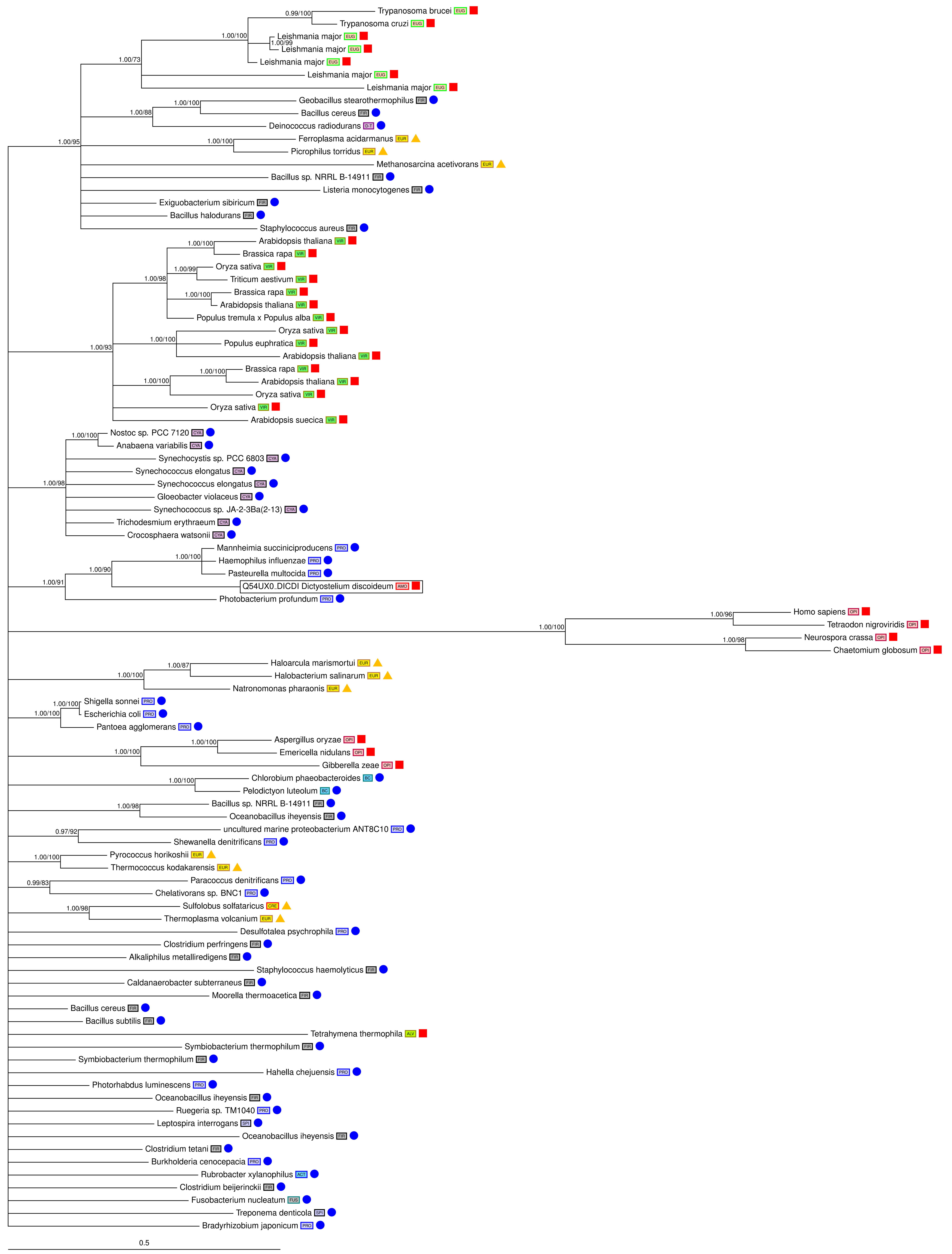

TN181

```
                Candy accession: Q5CRF9 CRYPV
               RefSeq accession: XP_652044.1
              Uniprot accession: Q5CRF9 CRYPV
                       Comments: LGT - CP TWO NODES
               Species affected: CP
         Adjacent taxa in tree: Firmicutes - Clostridia
EC annotation - (Blast/Profile): EC:3.2.1.1
                     PHOBIUS SP: 0
                    PHOBIUS TMD: 0
              RefSeq annotation: alpha-amylase family protein
      Name of enzyme/protein: Alpha-amylase
       KEGG PATHWAY - level 1: Carbohydrate Metabolism
       KEGG PATHWAY - level 2: Starch and sucrose metabolism
```

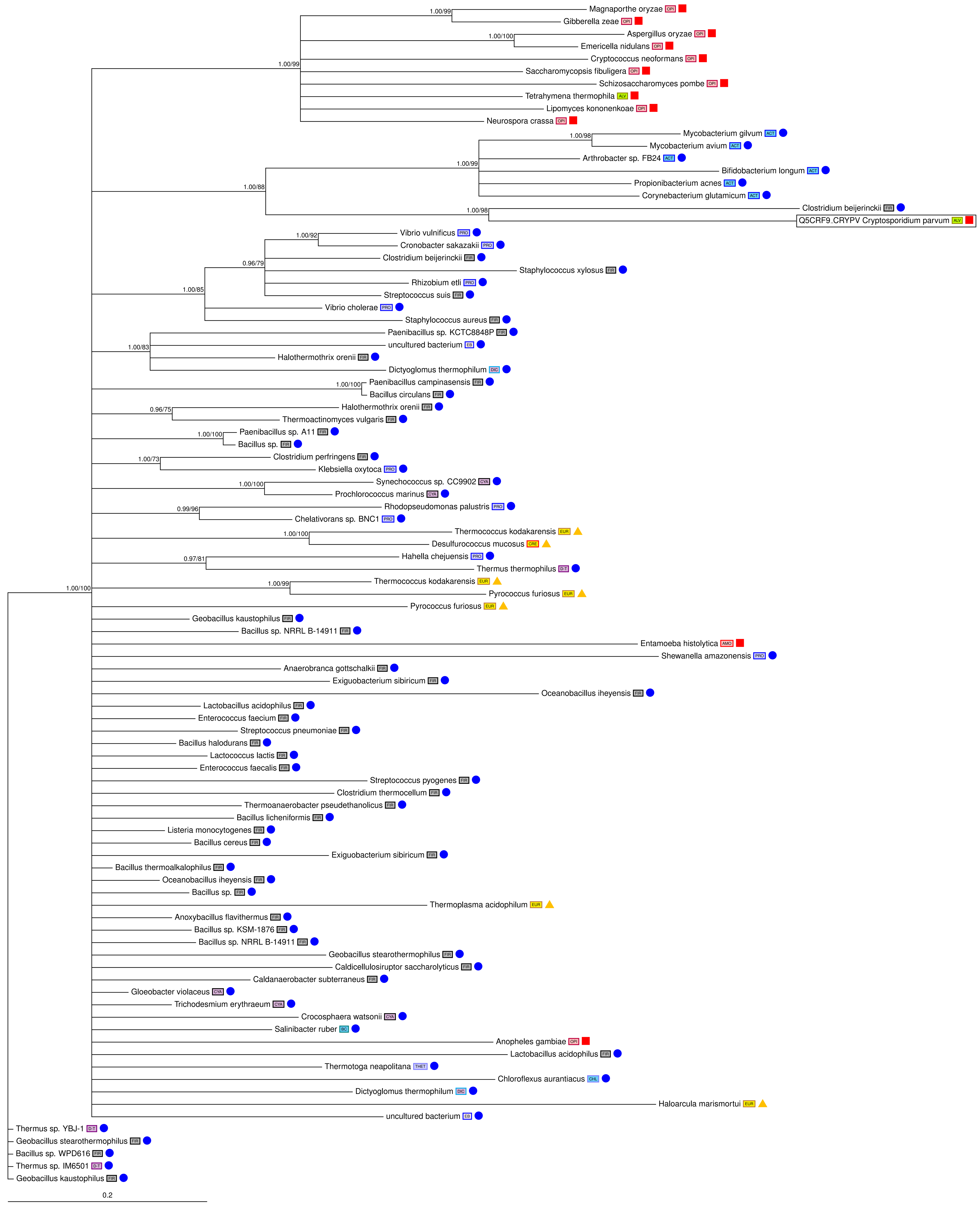

TN182

```
              Candy accession: Q7RD47_PLAYO
             RefSeq accession: XP_726051.1
            Uniprot accession: Q7RD47_PLAYO
                     Comments: LGT - APICOMPLEXA TWO NODES
                               (+CYANOBACTERIA EGT INTO PLANTS)
             Species affected: PF,PV,PY,PB,PC,TA,TG
         Adjacent taxa in tree: Bacteria
EC annotation - (Blast/Profile): EC:1.1.1.267
                  PHOBIUS SP: Y
                 PHOBIUS TMD: 0
            RefSeq annotation: 1-deoxy-D-xylulose 5-phosphate
                               reductoisomerase
      Name of enzyme/protein: 1-deoxy-D-xylulose 5-phosphate
                               reductoisomerase
      KEGG PATHWAY - level 1: Metabolism of Terpenoids and Polyketides
      KEGG PATHWAY - level 2: Terpenoid backbone biosynthesis
```

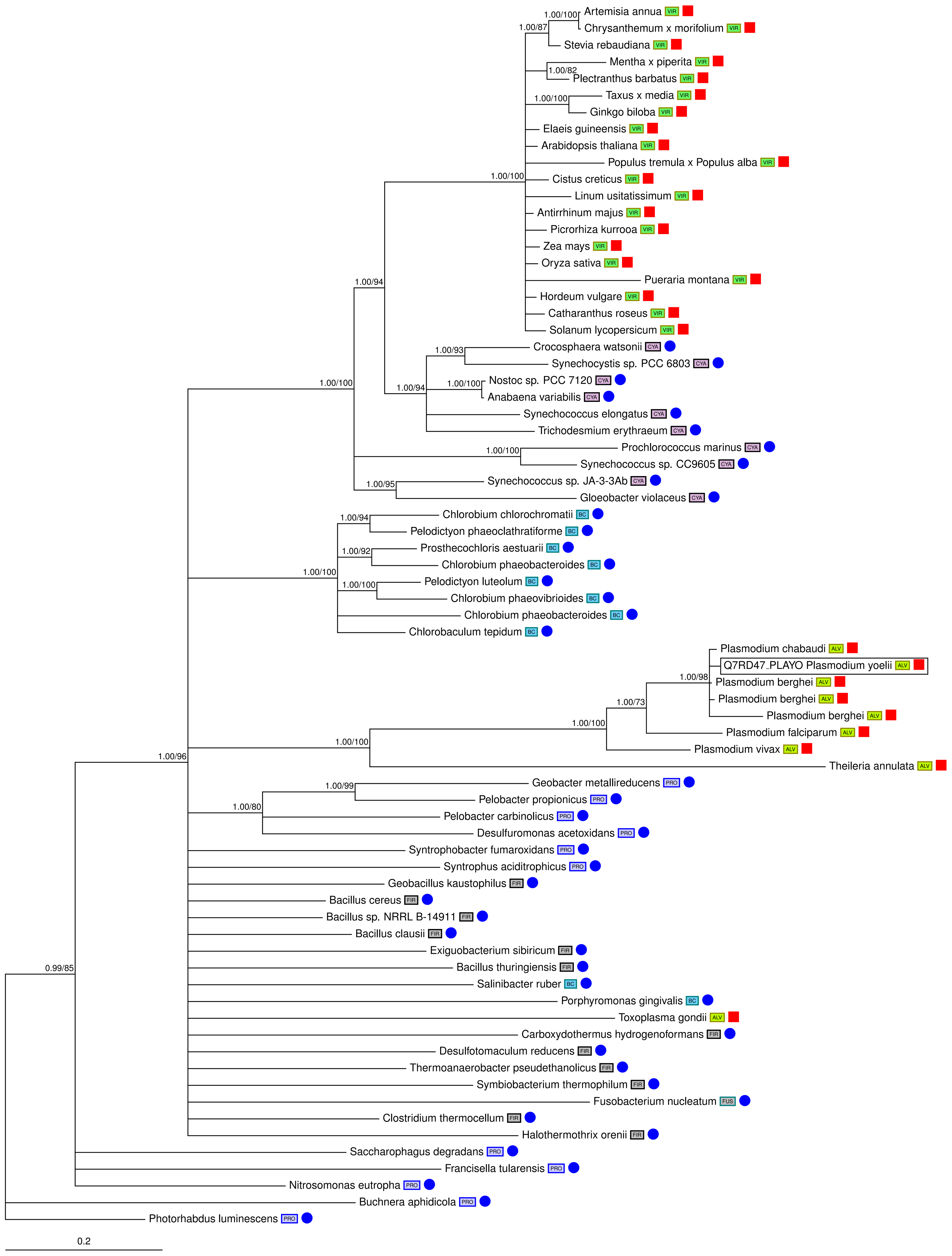

TN183

```
              Candy accession: Q50NN9_ENTHI
             RefSeq accession: XP_648590.1
            Uniprot accession: Q9NH04_ENTHI
                     Comments: LGT - EH TWO NODES
             Species affected: EH
         Adjacent taxa in tree: Spirochaetes - Treponema
EC annotation - (Blast/Profile): EC:1.1.1.38
                  PHOBIUS SP: 0
                 PHOBIUS TMD: 0
            RefSeq annotation: malic enzyme
       Name of enzyme/protein: malate dehydrogenase (oxaloacetate-
                               decarboxylating)
       KEGG PATHWAY - level 1: Carbohydrate Metabolism
       KEGG PATHWAY - level 2: Pyruvate metabolism
```

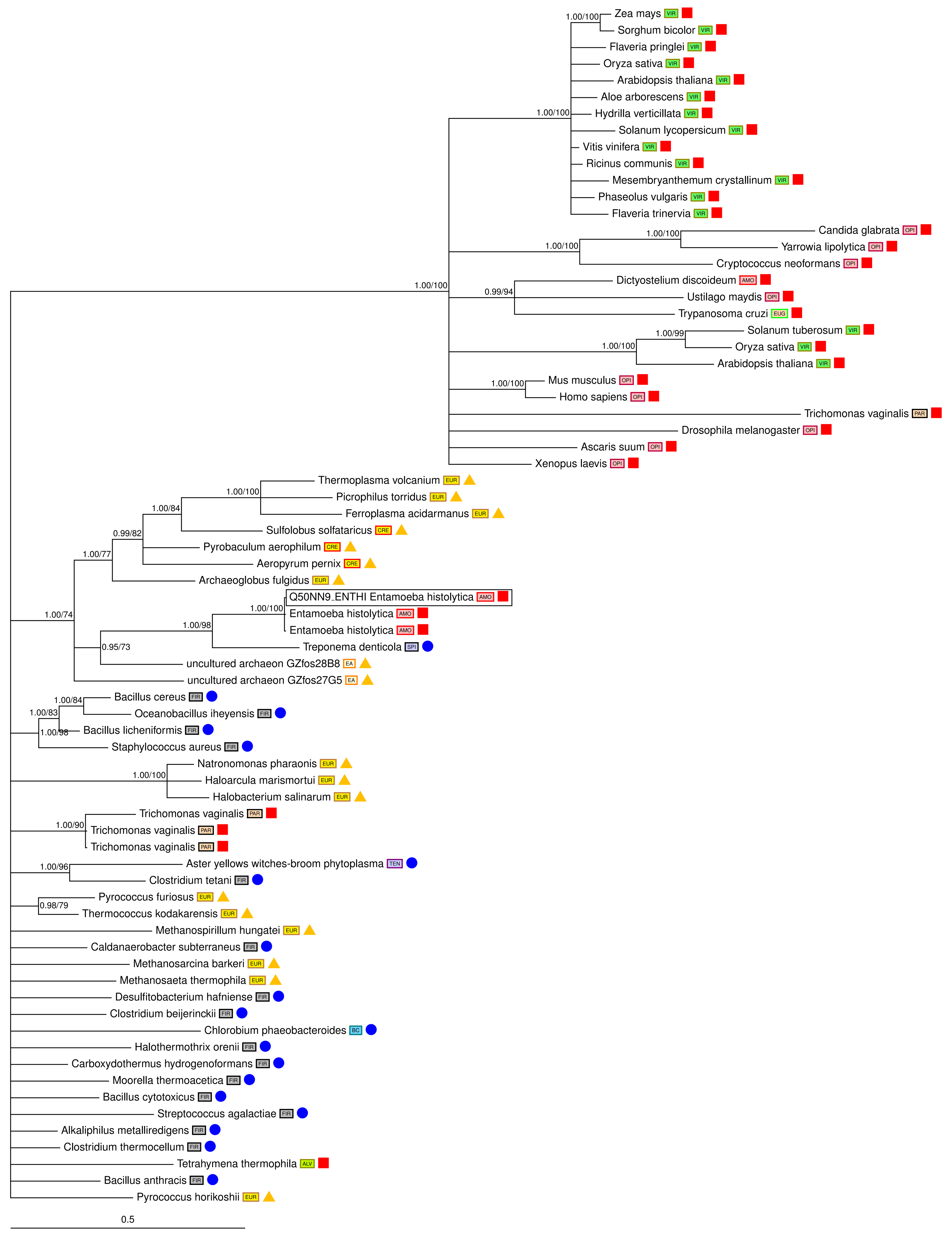

TN184

```
           Candy accession: Q50VG8_ENTHI
          RefSeq accession: XP_650972.1
         Uniprot accession: C4M0I4_ENTHI
                  Comments: LGT - EH TWO NODES
          Species affected: EH
     Adjacent taxa in tree: Firmicutes - Clostridia
EC annotation - (Blast/Profile): EC:3.5.2.3
                PHOBIUS SP: 0
               PHOBIUS TMD: 0
         RefSeq annotation: D-hydantoinase
Name of enzyme/protein: dihydroorotase
   KEGG PATHWAY - level 1: Nucleotide Metabolism
   KEGG PATHWAY - level 2: Pyrimidine metabolism
```

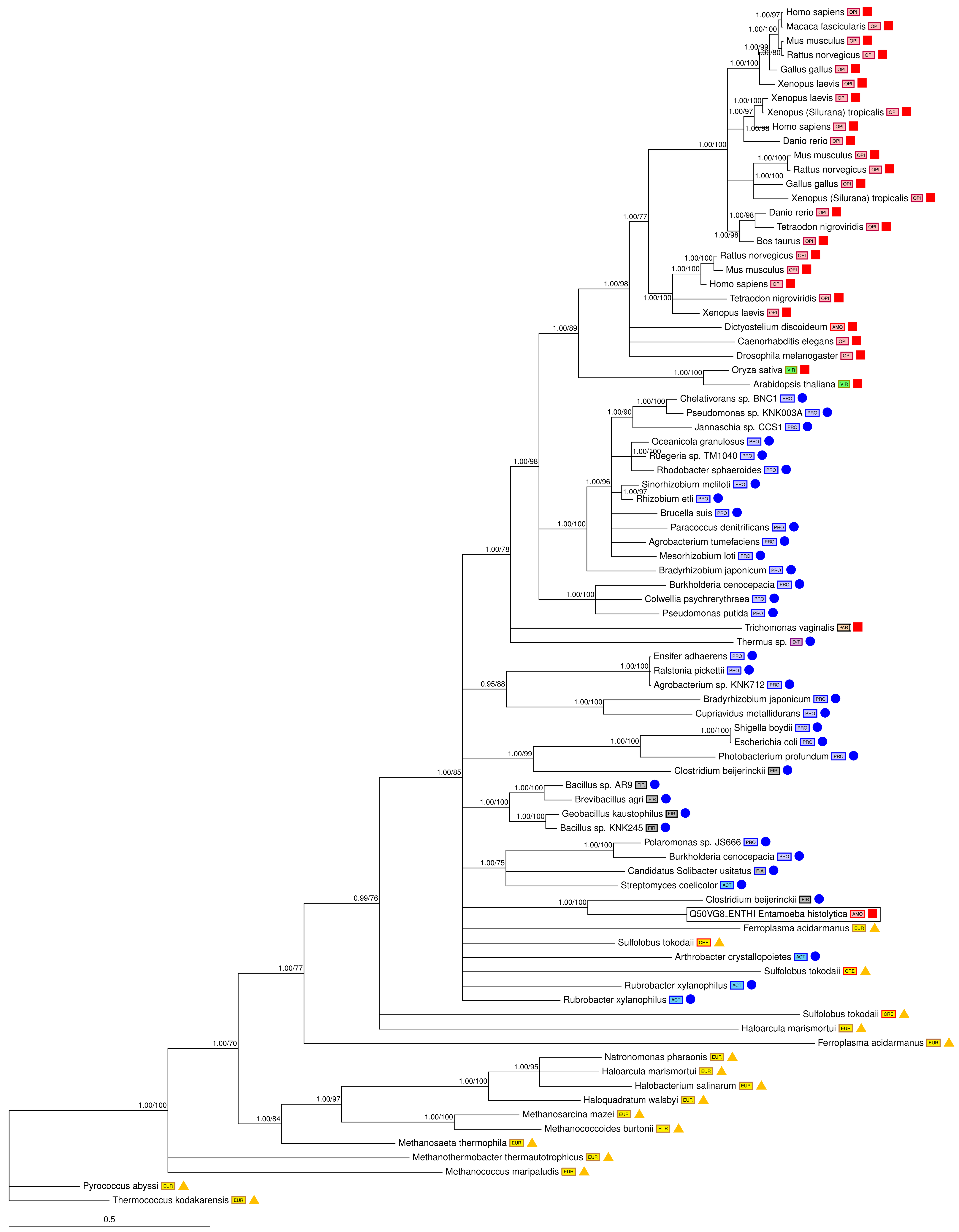

TN185

```
            Candy accession: Q86K10_DICDI
           RefSeq accession: XP_645046.1
          Uniprot accession: TKT_DICDI
                   Comments: LGT - DD TWO NODES
           Species affected: DD
      Adjacent taxa in tree: Chlamydiae/Verrucomicrobia
EC annotation - (Blast/Profile): EC:2.2.1.1
                 PHOBIUS SP: 0
                PHOBIUS TMD: 0
          RefSeq annotation: hypothetical protein DDB_G0272618
     Name of enzyme/protein: Transketolase
     KEGG PATHWAY - level 1: Carbohydrate Metabolism
     KEGG PATHWAY - level 2: Pentose phosphate pathway

            Candy accession: Q50WZ8_ENTHI
           RefSeq accession: XP_650850.1
          Uniprot accession: Q50WZ8_ENTHI
                   Comments: LGT - EH TWO NODES
           Species affected: EH
      Adjacent taxa in tree: Aquificae - Aquifex
EC annotation - (Blast/Profile): EC:2.2.1.1
                 PHOBIUS SP: 0
                PHOBIUS TMD: 0
          RefSeq annotation: transketolase
     Name of enzyme/protein: Transketolase
     KEGG PATHWAY - level 1: Carbohydrate Metabolism
     KEGG PATHWAY - level 2: Pentose phosphate pathway
```

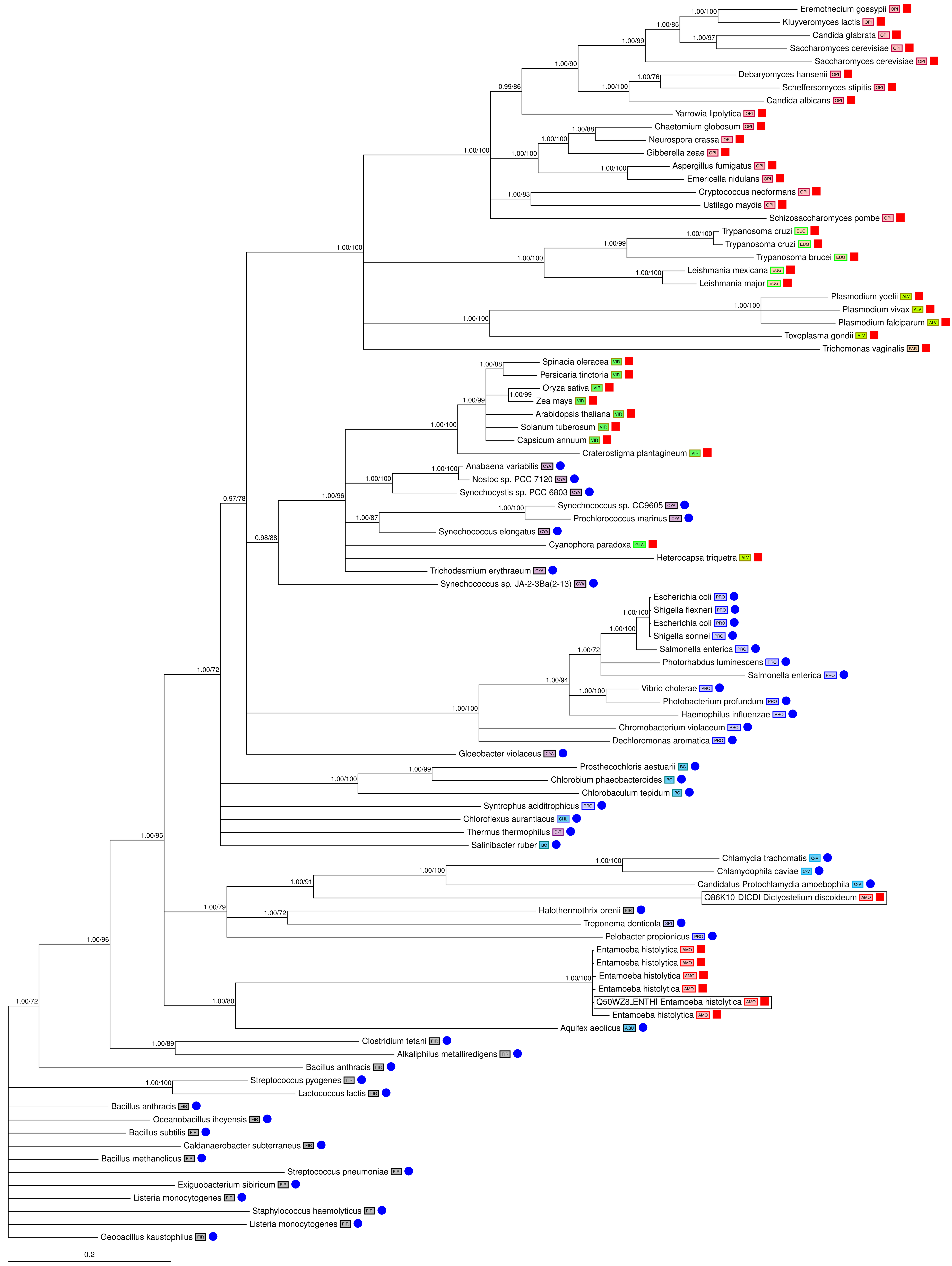

TN186

```
Candy accession: Q51DS3_ENTHI
RefSeq accession: XP_656380.2
Uniprot accession: C4LSC6_ENTHI
Comments: LGT - EH TWO NODES
Species affected: EH
Adjacent taxa in tree: Bacteroidetes/Chlorobi
EC annotation - (Blast/Profile): EC:3.4.19.1
PHOBIUS SP: 0
PHOBIUS TMD: 0
RefSeq annotation: prolyl oligopeptidase family protein
Name of enzyme/protein: acylaminoacyl-peptidase
KEGG PATHWAY - level 1: Reaction
KEGG PATHWAY - level 2: Reaction
```

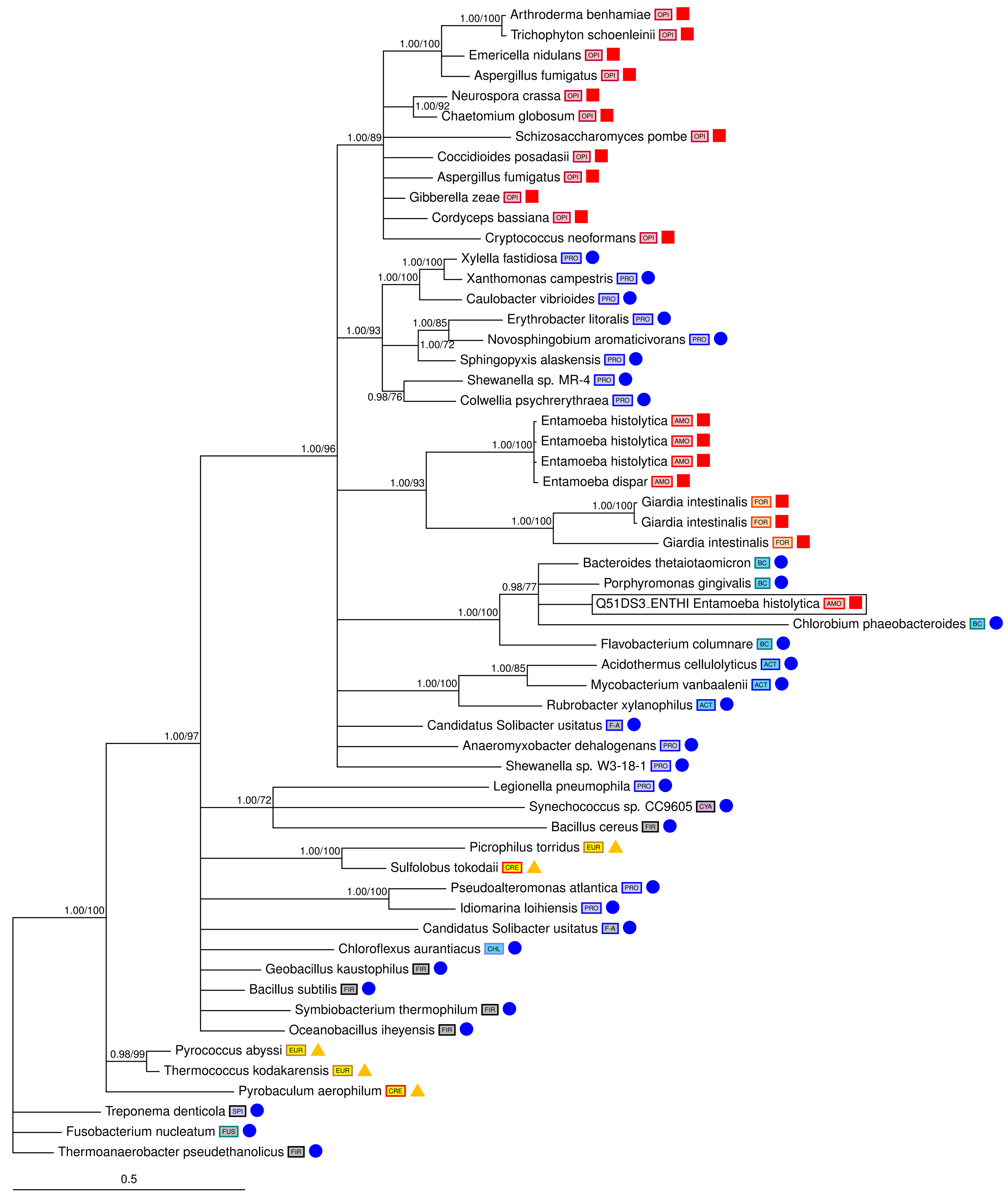

TN187

```
               Candy accession: Q4Q495_LEIMA
              RefSeq accession: XP_001685853.1
             Uniprot accession: Q4Q495_LEIMA
                      Comments: LGT - KINETOPLASTIDS TWO NODES
              Species affected: LM, TB,TC
        Adjacent taxa in tree: Proteobacteria
EC annotation - (Blast/Profile): EC:2.4.2.11
                    PHOBIUS SP: 0
                   PHOBIUS TMD: 0
             RefSeq annotation: nicotinate phosphoribosyltransferase
        Name of enzyme/protein: Nicotinate phosphoribosyltransferase
        KEGG PATHWAY - level 1: Metabolism of Cofactors and Vitamins
        KEGG PATHWAY - level 2: Nicotinate and nicotinamide metabolism

               Candy accession: Q51BM9_ENTHI
              RefSeq accession: XP_655646.1
             Uniprot accession: C4LUN7_ENTHI
                      Comments: LGT - EH TWO NODES
              Species affected: EH
        Adjacent taxa in tree: Bacteroidetes/Chlorobi
EC annotation - (Blast/Profile): EC:2.4.2.11
                    PHOBIUS SP: 0
                   PHOBIUS TMD: 0
             RefSeq annotation: nicotinate phosphoribosyltransferase
        Name of enzyme/protein: Nicotinate phosphoribosyltransferase
        KEGG PATHWAY - level 1: Metabolism of Cofactors and Vitamins
        KEGG PATHWAY - level 2: Nicotinate and nicotinamide metabolism
```

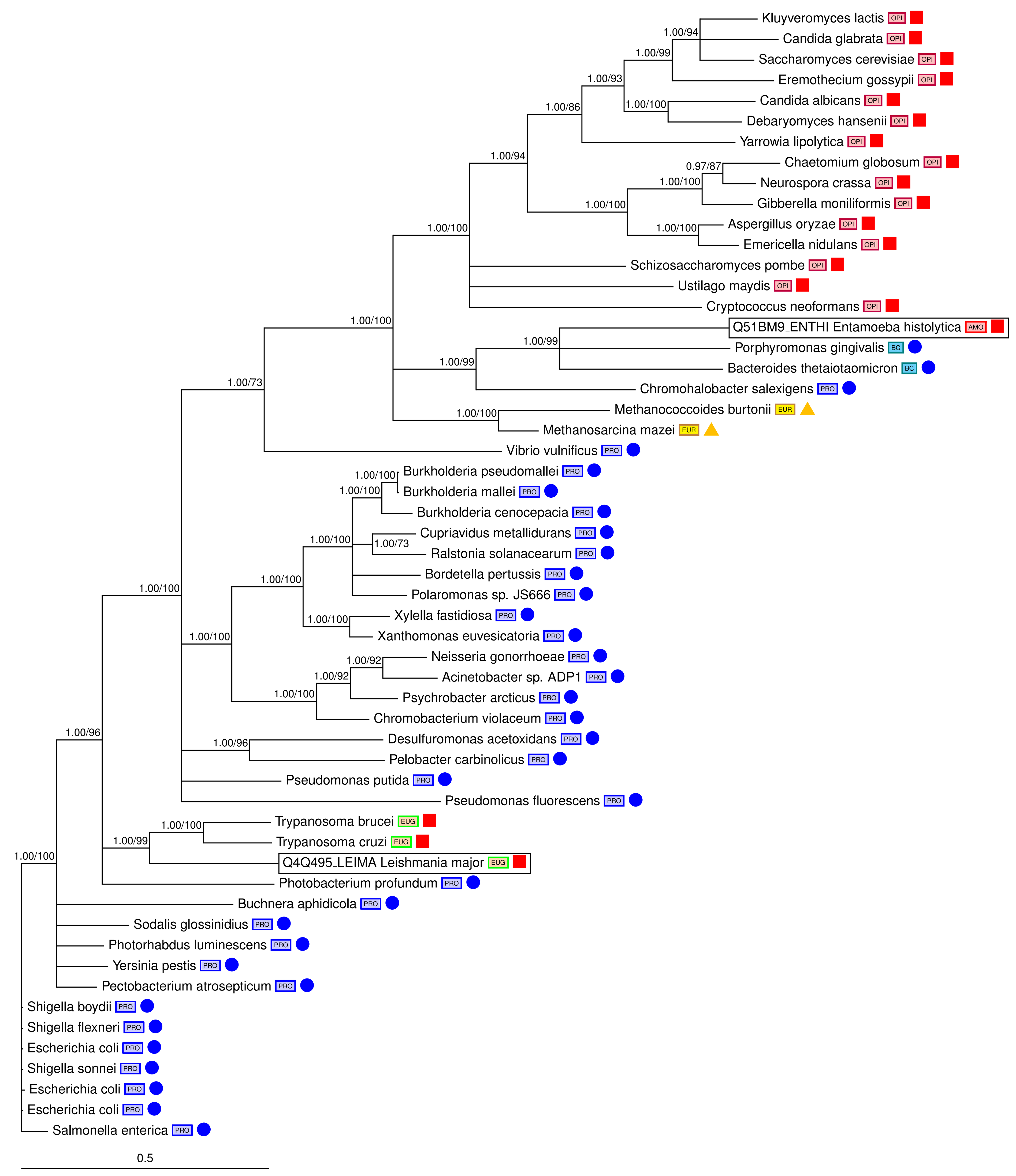

TN188

```
                Candy accession: Q53HX3_LEIMA
               RefSeq accession: XP_001686853.1
              Uniprot accession: Q53HX3_LEIMA
                       Comments: LGT - LM TWO NODES
               Species affected: LM
           Adjacent taxa in tree: Archaea - Methanospirillum
EC annotation - (Blast/Profile): EC:2.3.1.57
                     PHOBIUS SP: 0
                    PHOBIUS TMD: 0
               RefSeq annotation: acetyltransferase-like protein
       Name of enzyme/protein: diamine N-acetyltransferase
       KEGG PATHWAY - level 1: Amino Acid metabolism
       KEGG PATHWAY - level 2: Arginine and proline metabolism
```

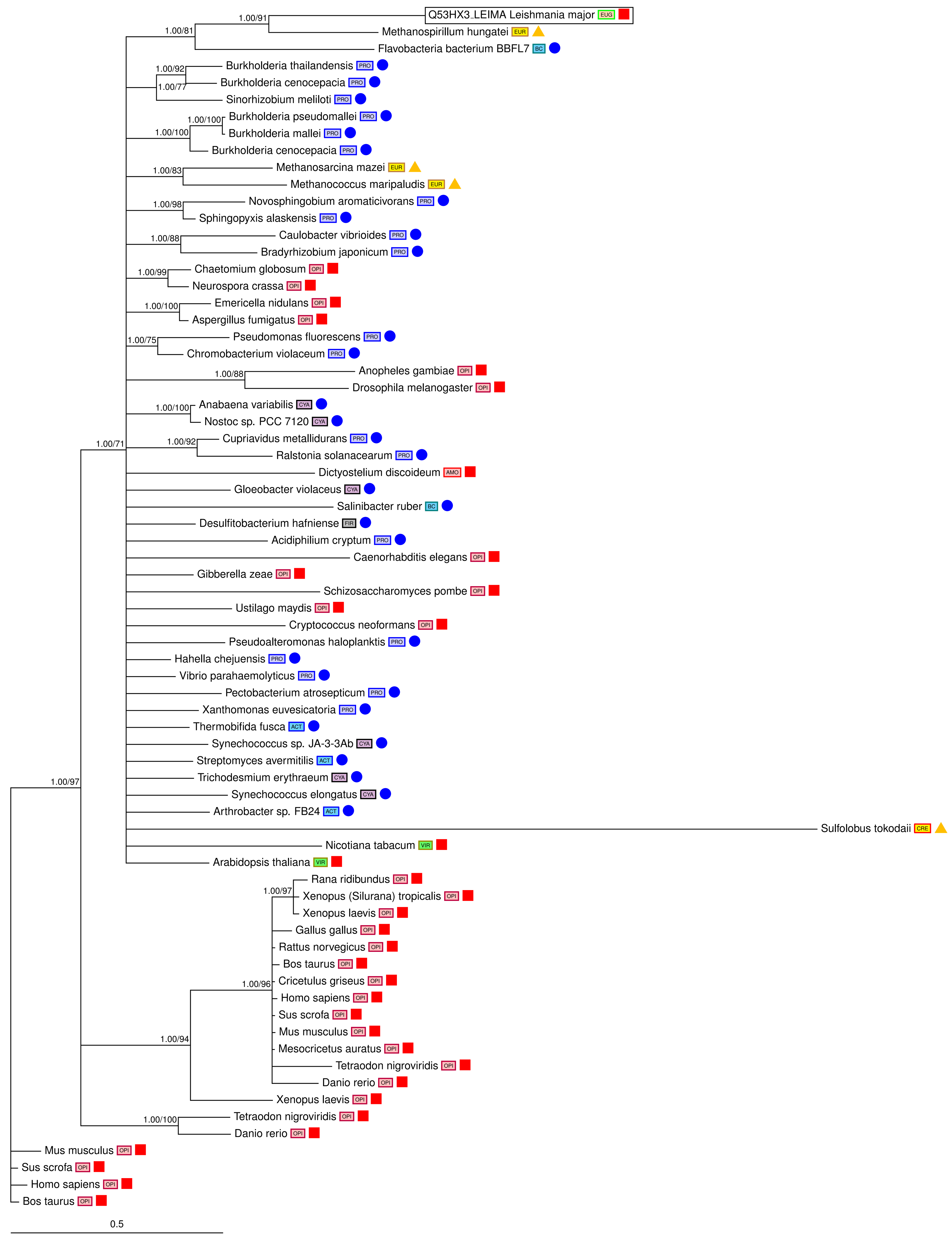

TN189

```
            Candy accession: Q57WJ2_9TRYP
           RefSeq accession: XP_846000.1
          Uniprot accession: Q57WJ2_9TRYP
                   Comments: LGT - KINETOPLASTIDS TWO NODES
           Species affected: LM,TC,TB
     Adjacent taxa in tree: Proteobacteria
EC annotation - (Blast/Profile): na
                 PHOBIUS SP: 0
                PHOBIUS TMD: 0
          RefSeq annotation: hypothetical protein
   Name of enzyme/protein: putative carbohydrate kinase
     KEGG PATHWAY - level 1: Other function
     KEGG PATHWAY - level 2: na
```

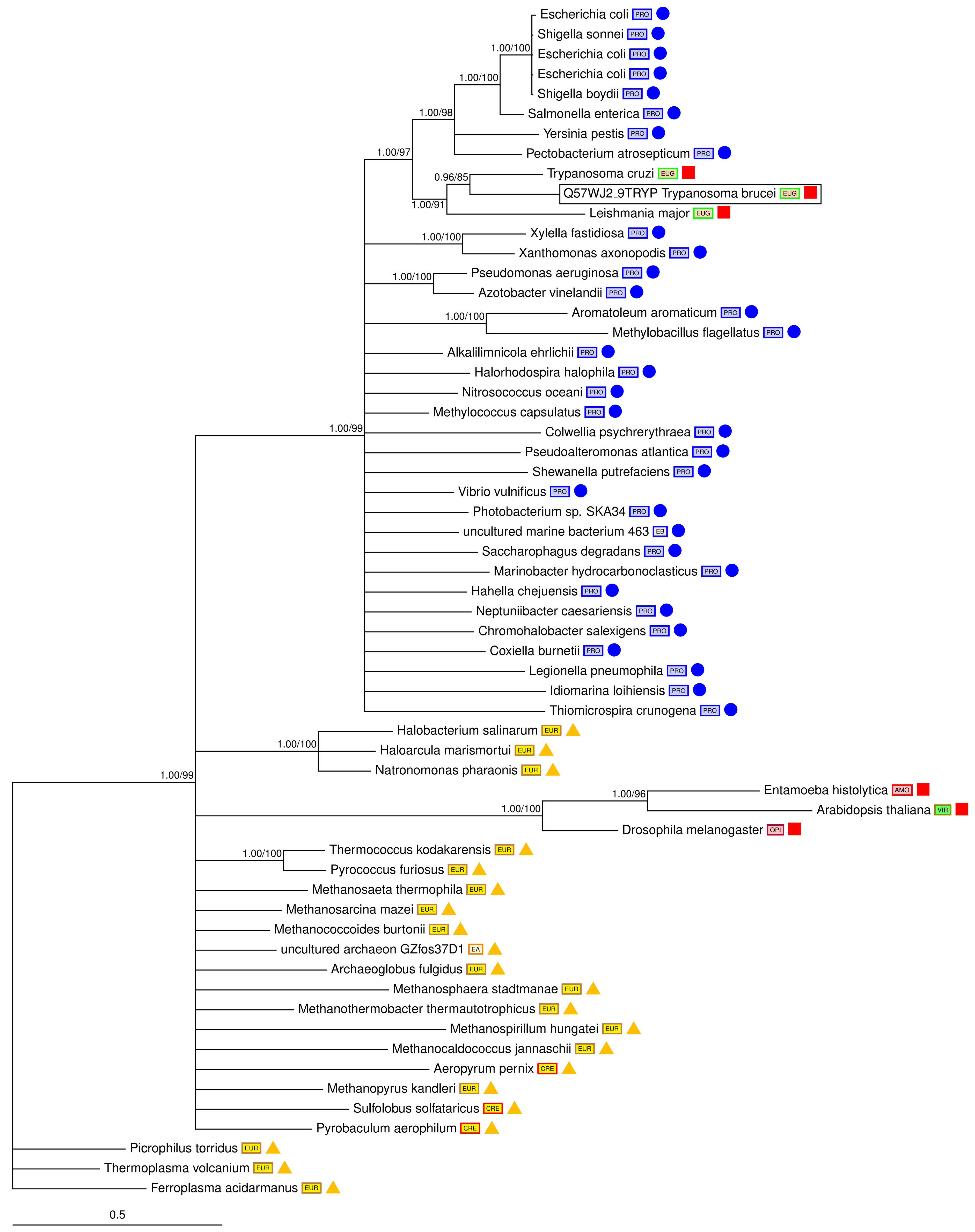

TN190

```
                Candy accession: Q7R205_GIALA
               RefSeq accession: XP_001709869.1
              Uniprot accession: A8B1Z3_GIALA
                       Comments: LGT - GI TWO NODES
               Species affected: GI
          Adjacent taxa in tree: Prokaryotes
EC annotation - (Blast/Profile): EC:4.1.1.32
                     PHOBIUS SP: 0
                    PHOBIUS TMD: 0
              RefSeq annotation: Phosphoenolpyruvate carboxykinase
       Name of enzyme/protein: Phosphoenolpyruvate carboxykinase (GTP)
       KEGG PATHWAY - level 1: Carbohydrate Metabolism
       KEGG PATHWAY - level 2: Glycolysis / Gluconeogenesis, Citrate
                               cycle (TCA cycle), Pyruvate metabolism
```

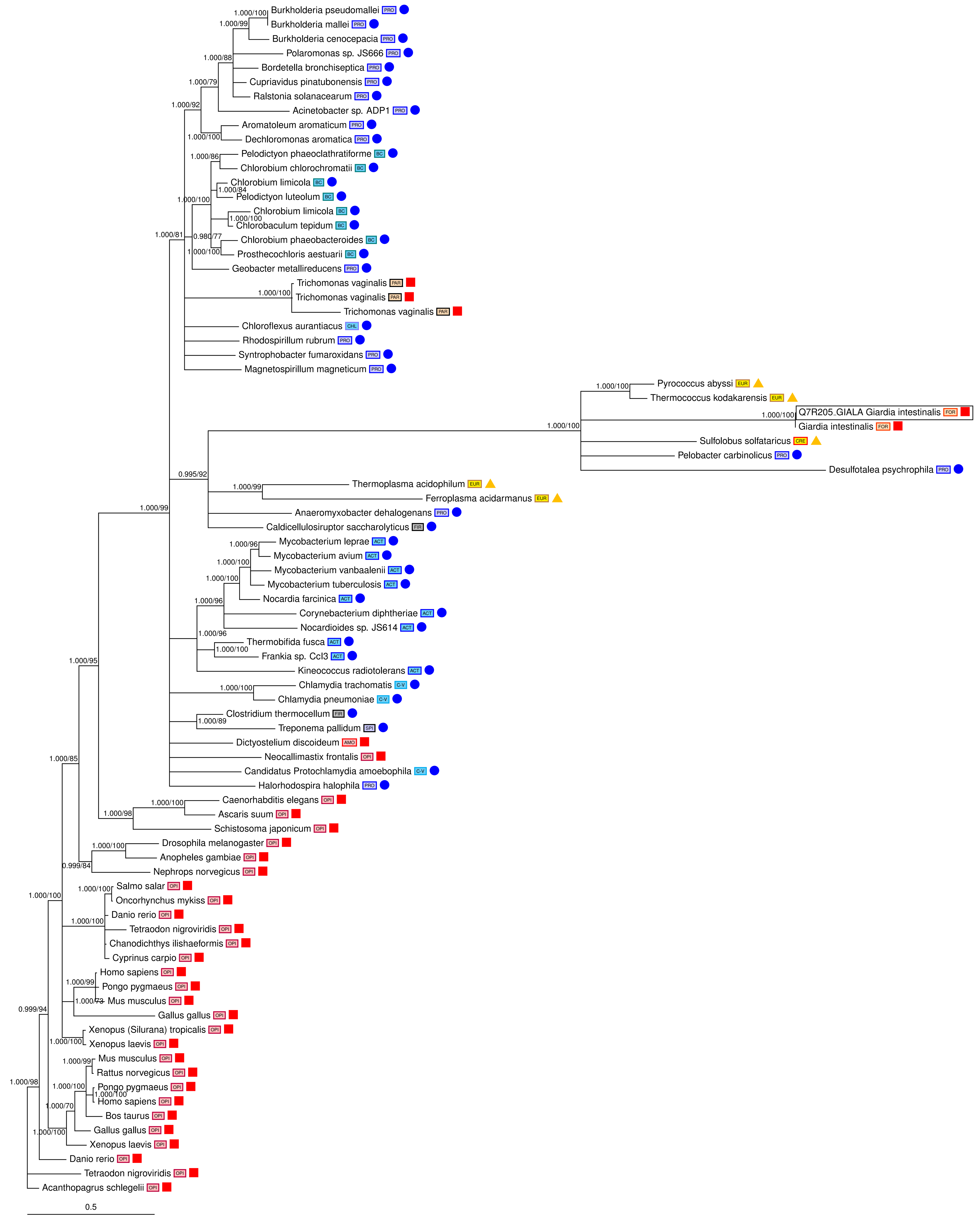

TN191

```
             Candy accession: T20_03789
            RefSeq accession: XP_002367616.1
           Uniprot accession: B6KHX1_TOXGO
                    Comments: LGT - TG TWO NODES - ONE NODE FOR FUNGI
                              LGT
            Species affected: TG,FUNGI
      Adjacent taxa in tree: Archaea - Methanosarcina
EC annotation - (Blast/Profile): EC:3.5.2.6
                 PHOBIUS SP: 0
                PHOBIUS TMD: 0
           RefSeq annotation: alkyl sulfatase, putative
     Name of enzyme/protein: beta-lactam hydrolase
     KEGG PATHWAY - level 1: Biosynthesis of Other Secondary
                              Metabolites
     KEGG PATHWAY - level 2: Penicillin and cephalosporin
                              biosynthesis, beta-Lactam resistance
```

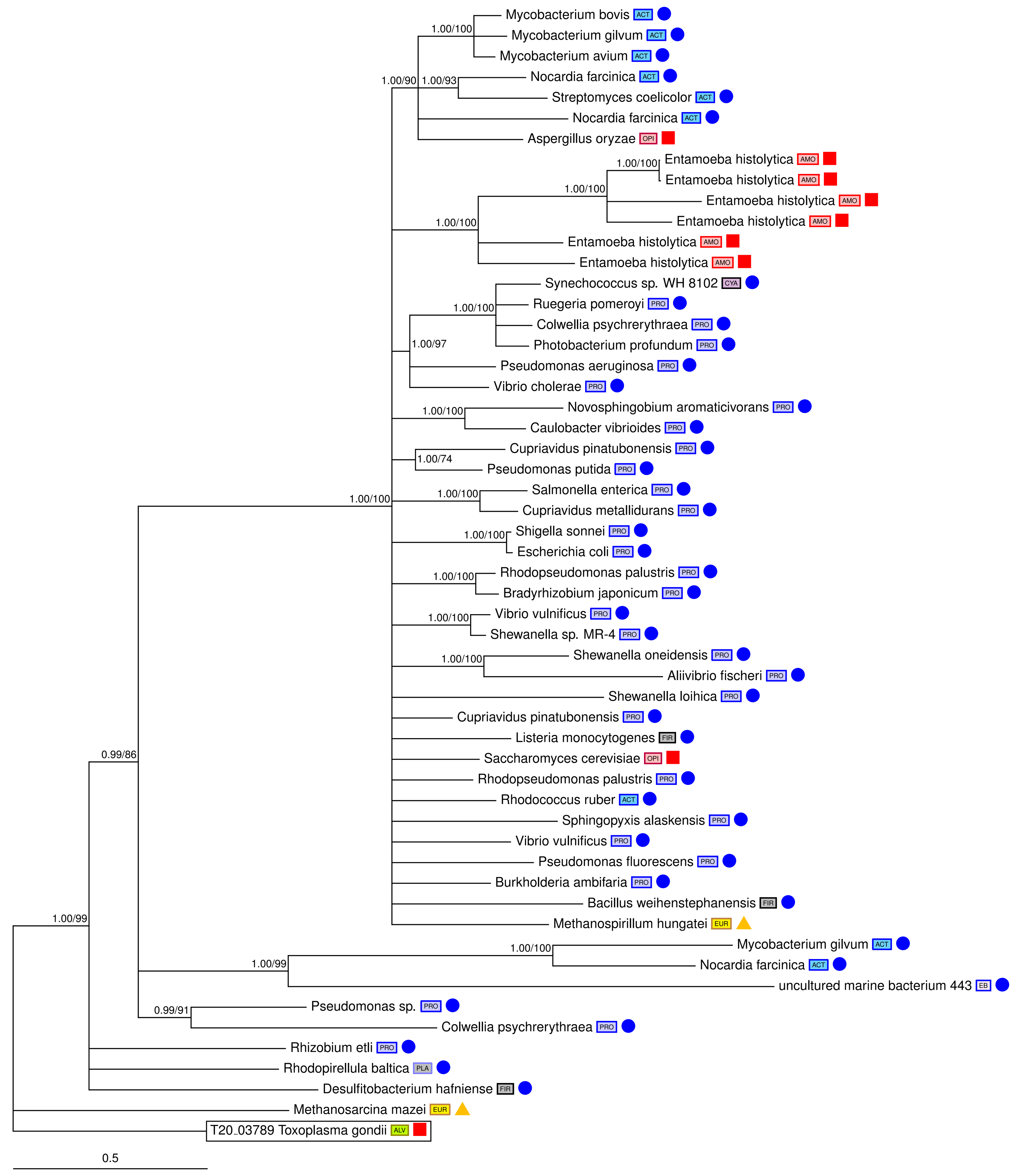

TN192

```
              Candy accession: Q512H5_ENTHI
             RefSeq accession: XP_653570.1
            Uniprot accession: C4LVD3_ENTHI
                     Comments: LGT - EH TWO NODES + LGT FROM
                               CYANOBACTERIA INTO CHLAMYDOMONAS - EGT?
             Species affected: EH,GREEN ALGAE
        Adjacent taxa in tree: Proteobacteria
EC annotation - (Blast/Profile): EC:2.7.7.4
                   PHOBIUS SP: 0
                  PHOBIUS TMD: 0
            RefSeq annotation: sulfate adenylyltransferase
        Name of enzyme/protein: Sulfate adenylyltransferase
        KEGG PATHWAY - level 1: Nucleotide Metabolism, Metabolism of
                               Other Amino Acids, Energy metabolism
        KEGG PATHWAY - level 2: Purine metabolism, Selenocompound
                               metabolism, Sulfur metabolism
```

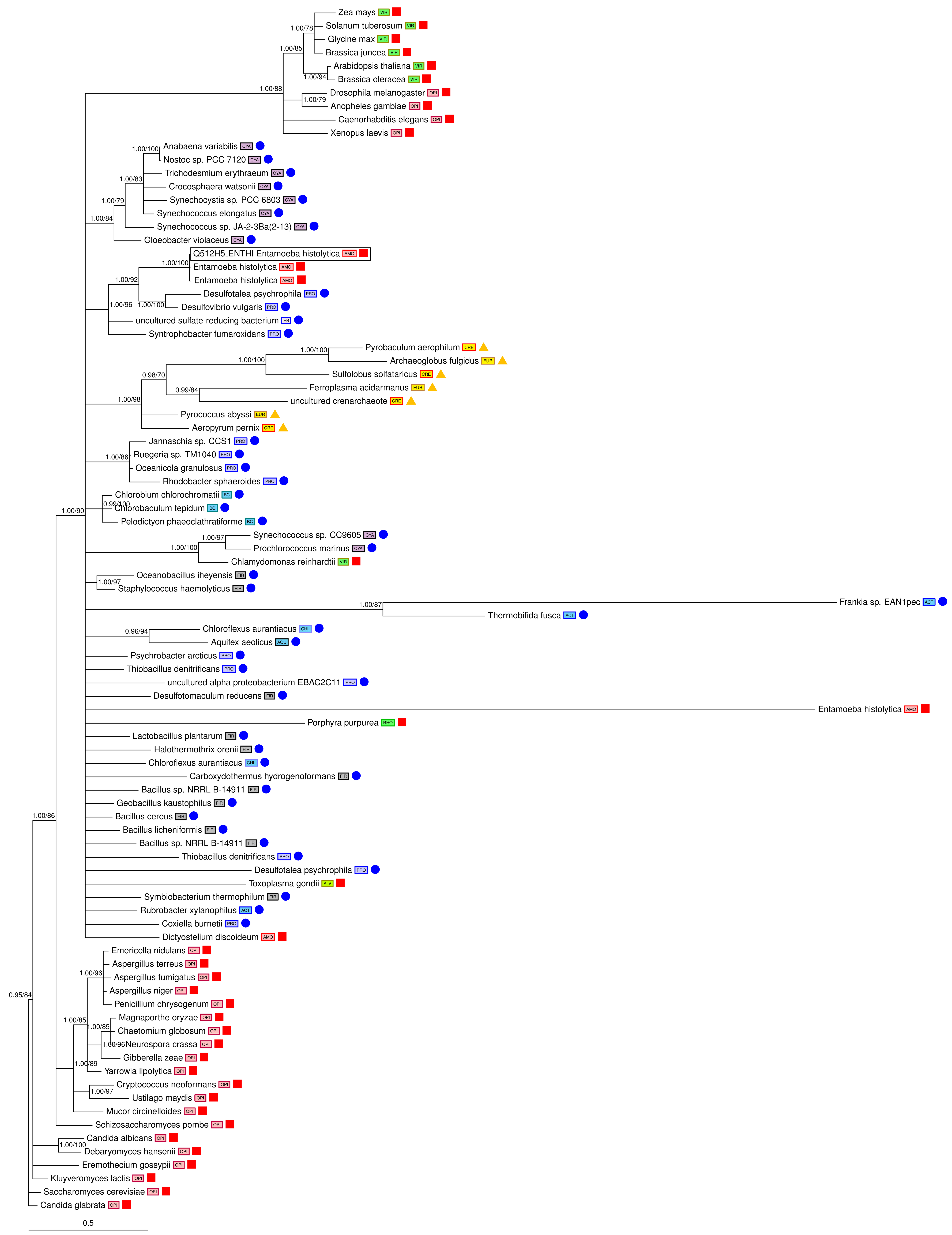

TN193

```
           Candy accession: Q51AV2 ENTHI
           RefSeq accession: XP_655383.1
          Uniprot accession: Q60I38_ENTHI
                   Comments: LGT - EH TWO NODES
           Species affected: EH
       Adjacent taxa in tree: Bacteroidetes/Chlorobi
EC annotation - (Blast/Profile): EC:2.6.1.52
                 PHOBIUS SP: 0
                PHOBIUS TMD: 0
          RefSeq annotation: phosphoserine aminotransferase
     Name of enzyme/protein: phosphoserine transaminase
     KEGG PATHWAY - level 1: Amino Acid Metabolism, Metabolism of
                             Cofactors and Vitamins
     KEGG PATHWAY - level 2: Glycine, serine and threonine
                             metabolism, Vitamin B6 metabolism
```

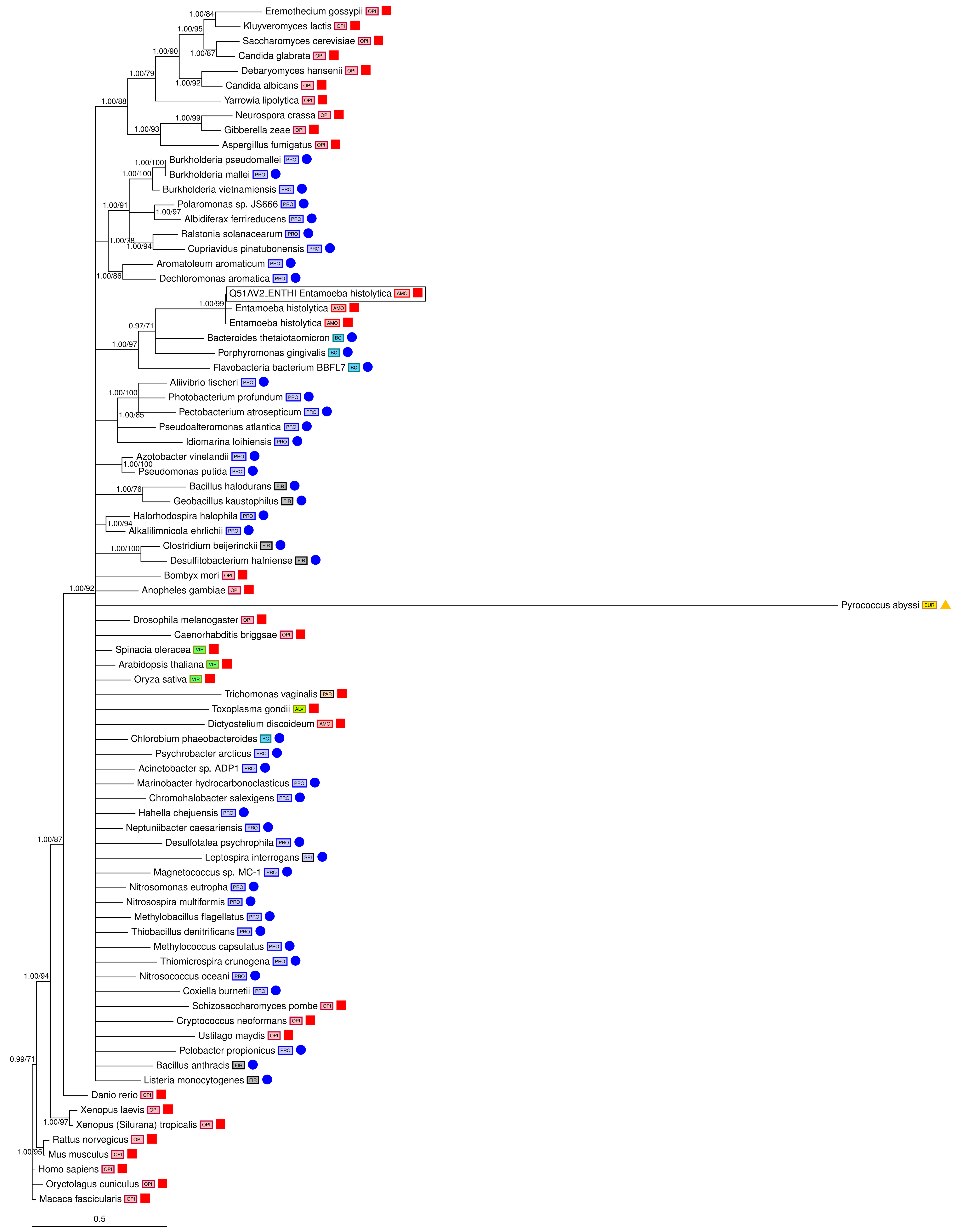

TN194

Candy accession: TV88712302
RefSeq accession: XP_001579287.1
Uniprot accession: A2DMT0_TRIVA
Comments: LGT - TV TWO NODES - LGT INTO TRITRICHOMONAS AND FUNGI?
Species affected: TV
Adjacent taxa in tree: Firmicutes
EC annotation - (Blast/Profile): EC:3.2.1.26
PHOBIUS SP: 0
PHOBIUS TMD: 0
RefSeq annotation: glycosyl hydrolase
Name of enzyme/protein: Beta-fructosidase-like protein
KEGG PATHWAY - level 1: Carbohydrate Metabolism
KEGG PATHWAY - level 2: Galactose metabolism, Starch and sucrose metabolism

Candy accession: Q4QB75_LEIMA
RefSeq accession: XP_001683423.1
Uniprot accession: Q4QB75_LEIMA
Comments: LGT - LM TWO NODES - LGT INTO TRITRICHOMONAS AND FUNGI?
Species affected: LM
Adjacent taxa in tree: Proteobacteria
EC annotation - (Blast/Profile): EC:3.2.1.26
PHOBIUS SP: 0
PHOBIUS TMD: 0
RefSeq annotation: beta-fructosidase-like protein; invertase-like protein; sucrose hydrolase-like protein
Name of enzyme/protein: Beta-fructosidase-like protein
KEGG PATHWAY - level 1: Carbohydrate Metabolism
KEGG PATHWAY - level 2: Galactose metabolism, Starch and sucrose metabolism

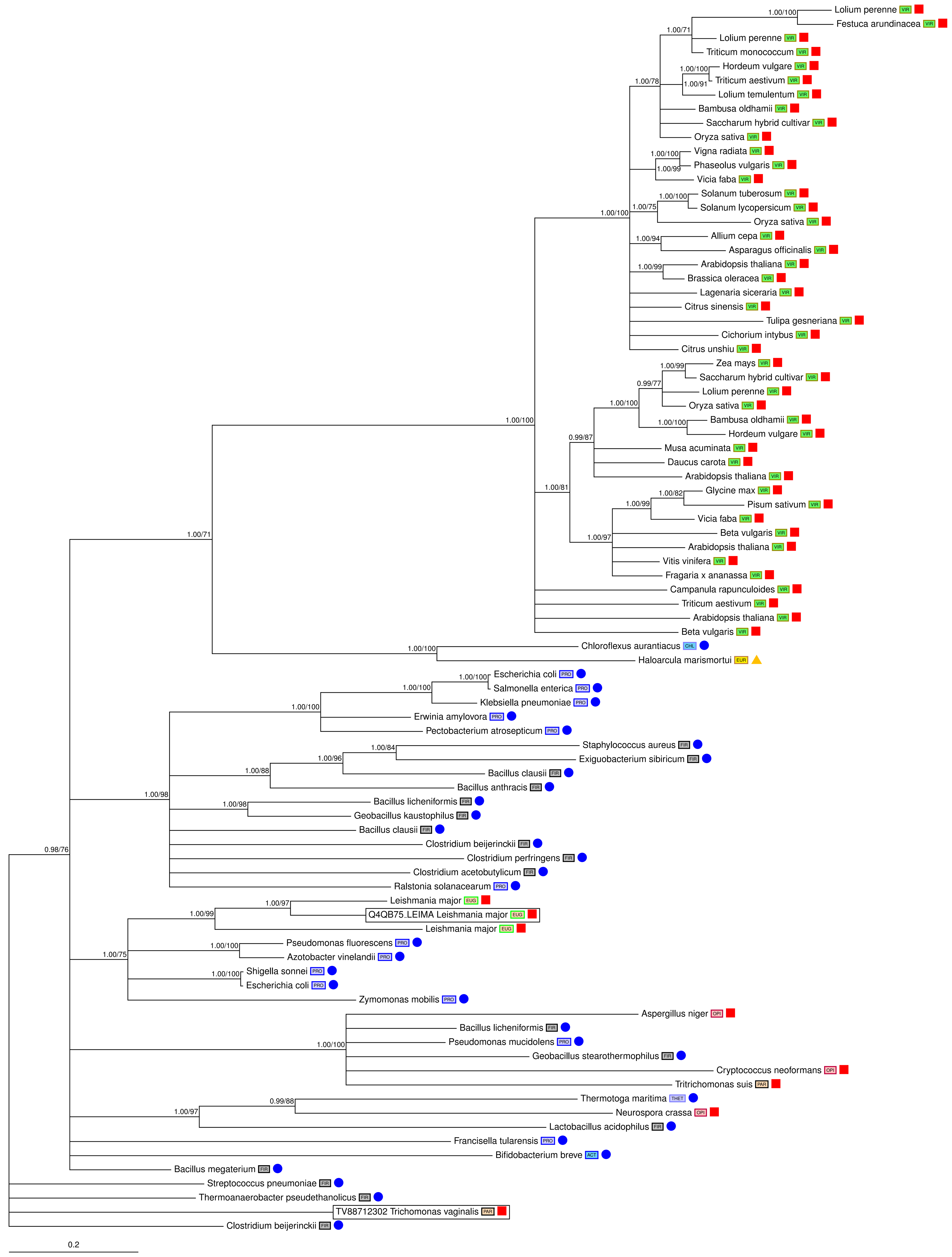

TN195

```
              Candy accession: Q4QEH4_LEIMA
             RefSeq accession: XP_001682274.1
            Uniprot accession: Q4QEH4_LEIMA
                     Comments: LGT - KINETOPLASTIDS TWO NODES
             Species affected: LM,TB,TC,
        Adjacent taxa in tree: Proteobacteria
EC annotation - (Blast/Profile): EC:3.4.11.14
                   PHOBIUS SP: 0
                  PHOBIUS TMD: 0
            RefSeq annotation: peptidase T; peptidase T, metallo-
                               peptidase, ClanMH, family M20B
       Name of enzyme/protein: cytosol alanyl aminopeptidase
       KEGG PATHWAY - level 1: Reaction
       KEGG PATHWAY - level 2: Reaction
```

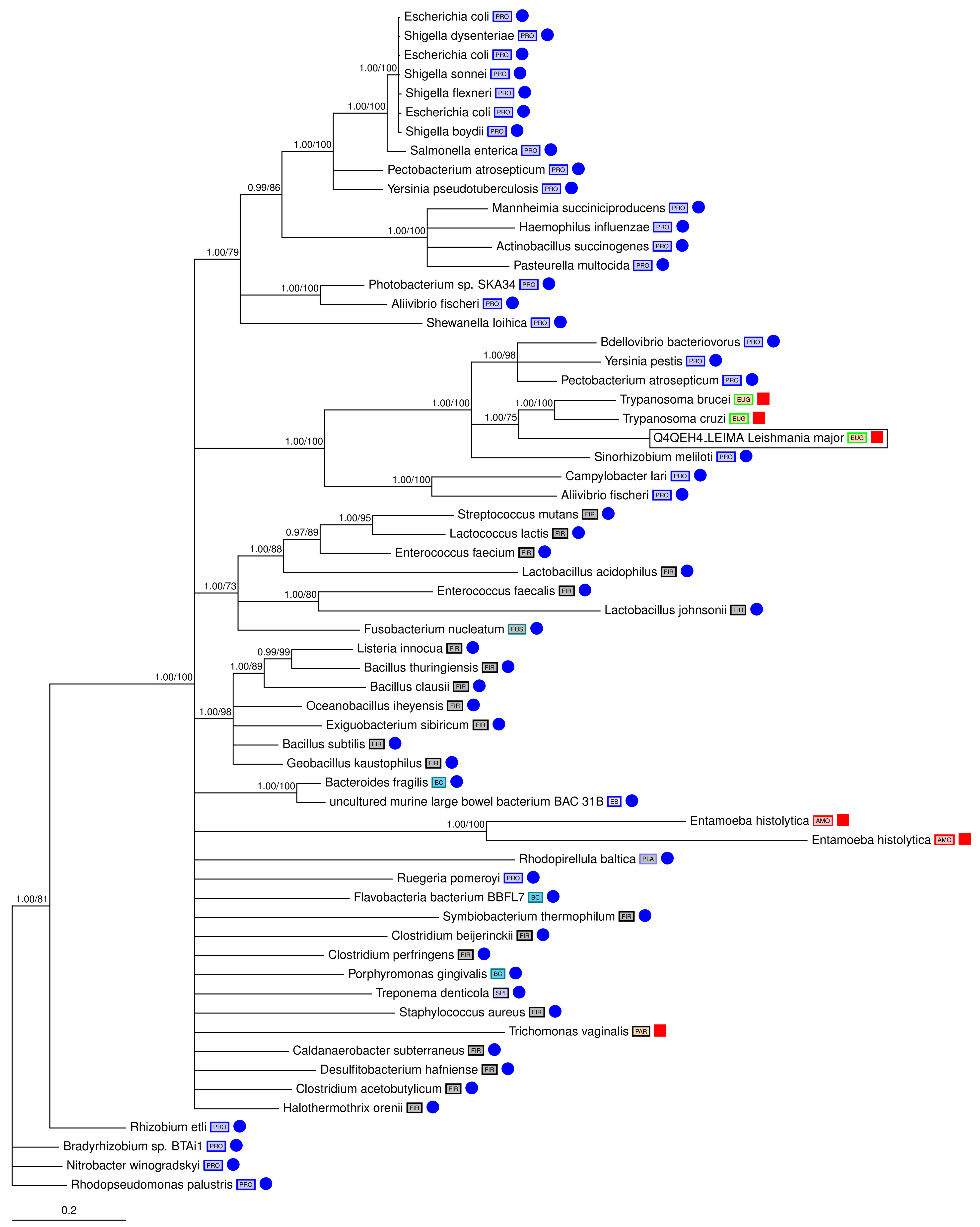

TN196

```
             Candy accession: TV96044382
            RefSeq accession: XP_001583076.1
           Uniprot accession: A2DC49_TRIVA
                    Comments: LGT - TV TWO NODES
            Species affected: TV
      Adjacent taxa in tree: Proteobacteria
EC annotation - (Blast/Profile): EC:4.1.1.15
                  PHOBIUS SP: 0
                 PHOBIUS TMD: 0
           RefSeq annotation: glutamate decarboxylase beta
    Name of enzyme/protein: Glutamate decarboxylase
      KEGG PATHWAY - level 1: Amino Acid Metabolism, Metabolism of
                              Other Amino Acids, Carbohydrate
                              Metabolism
      KEGG PATHWAY - level 2: Alanine, aspartate and glutamate
                              metabolism, Beta-Alanine metabolism,
                              Taurine and hypotaurine metabolism,
                              Butanoate metabolism

             Candy accession: Q54VQ5_DICDI
            RefSeq accession: XP_641300.1
           Uniprot accession: GADA_DICDI
                    Comments: LGT - DD TWO NODES
            Species affected: DD
      Adjacent taxa in tree: Proteobacteria
EC annotation - (Blast/Profile): EC:4.1.1.15
                  PHOBIUS SP: 0
                 PHOBIUS TMD: 0
           RefSeq annotation: glutamate decarboxylase
    Name of enzyme/protein: Glutamate decarboxylase
      KEGG PATHWAY - level 1: Amino Acid Metabolism, Metabolism of
                              Other Amino Acids, Carbohydrate
                              Metabolism
      KEGG PATHWAY - level 2: Alanine, aspartate and glutamate
                              metabolism, Beta-Alanine metabolism,
                              Taurine and hypotaurine metabolism,
                              Butanoate metabolism
```

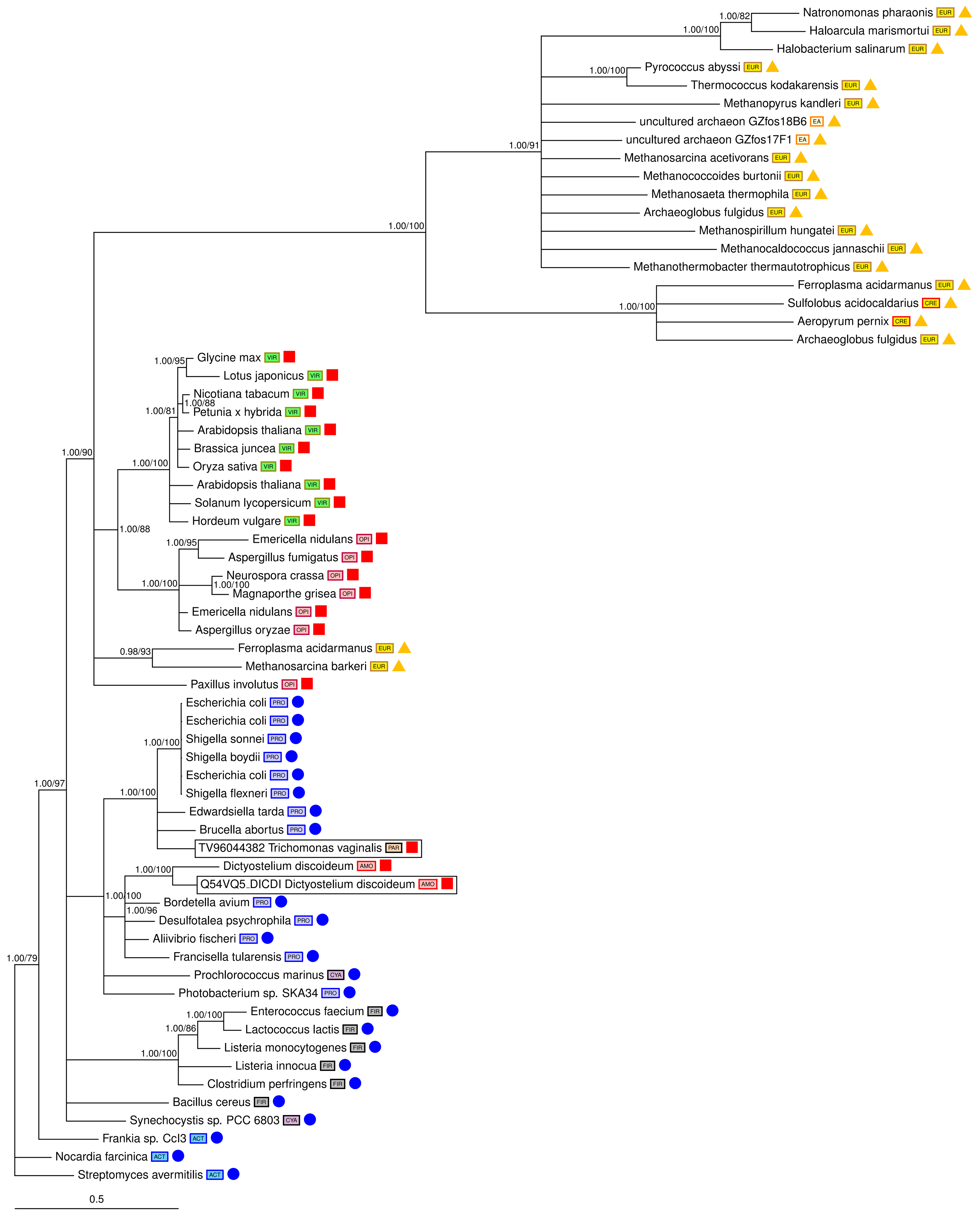

TN197

```
          Candy accession: Q4FYX2 LEIMA
        RefSeq accession: XP_822272.1
       Uniprot accession: Q4FYX2_LEIMA
                Comments: LGT - LM,LD TWO NODES
        Species affected: LM
    Adjacent taxa in tree: Proteobacteria
EC annotation - (Blast/Profile): EC:3.4.24.70
              PHOBIUS SP: 0
             PHOBIUS TMD: 0
        RefSeq annotation: metallo-peptidase, Clan MA(E), Family M3
    Name of enzyme/protein: oligopeptidase A
    KEGG PATHWAY - level 1: Reaction
    KEGG PATHWAY - level 2: Reaction
```

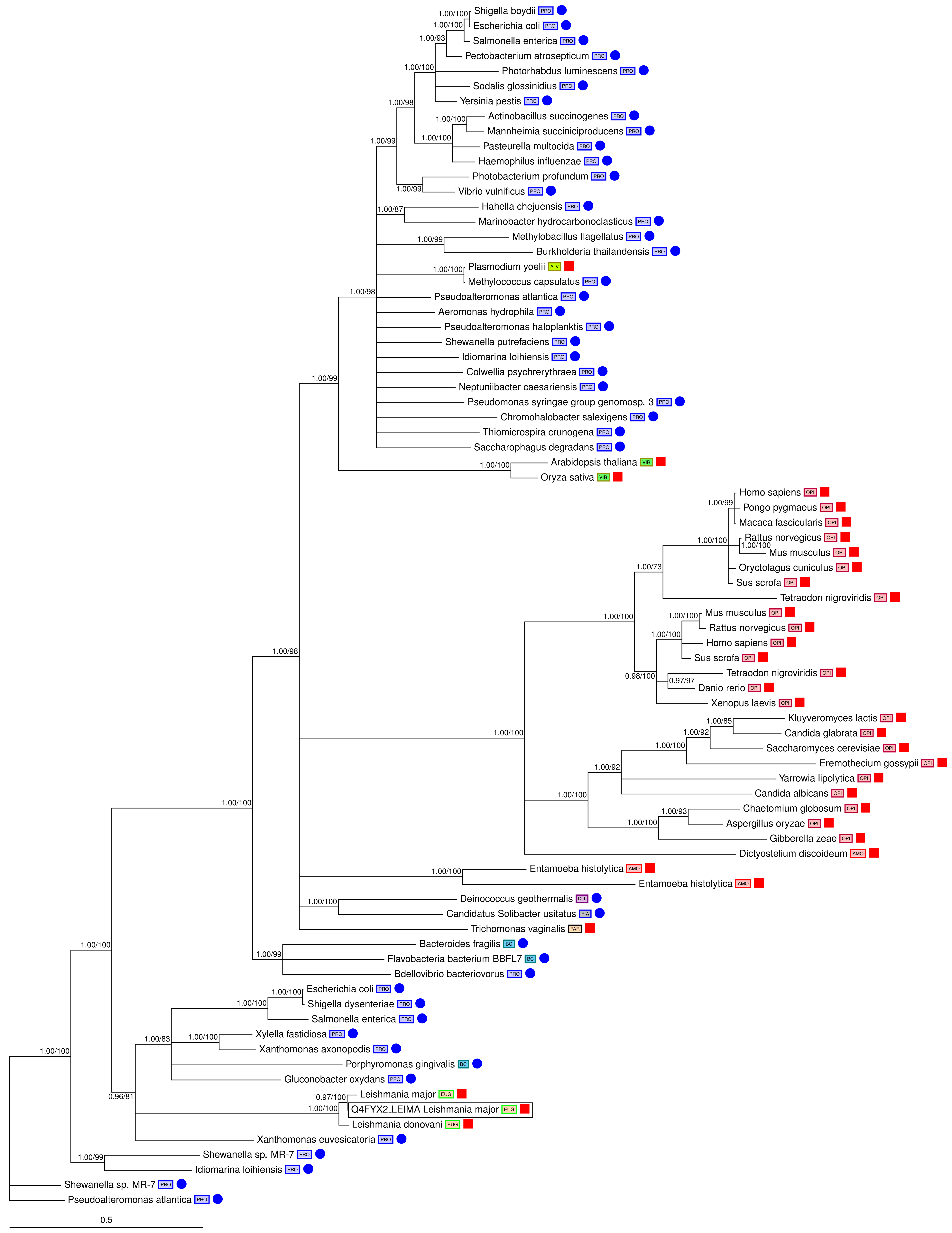

TN198

Candy accession: PV088945
RefSeq accession: XP_001614881.1
Uniprot accession: A5K5G9_PLAVS
Comments: LGT - APICOMPLEXA TWO NODES
Species affected: PF,PV,PY,PB, TV
Adjacent taxa in tree: Bacteria
EC annotation - (Blast/Profile): EC:6.1.1.4
PHOBIUS SP: 0
PHOBIUS TMD: 0
RefSeq annotation: leucyl-tRNA synthetase
Name of enzyme/protein: Leucyl-tRNA synthetase
KEGG PATHWAY - level 1: Translation - Genetic Information Processing
KEGG PATHWAY - level 2: Aminoacyl-tRNA biosynthesis

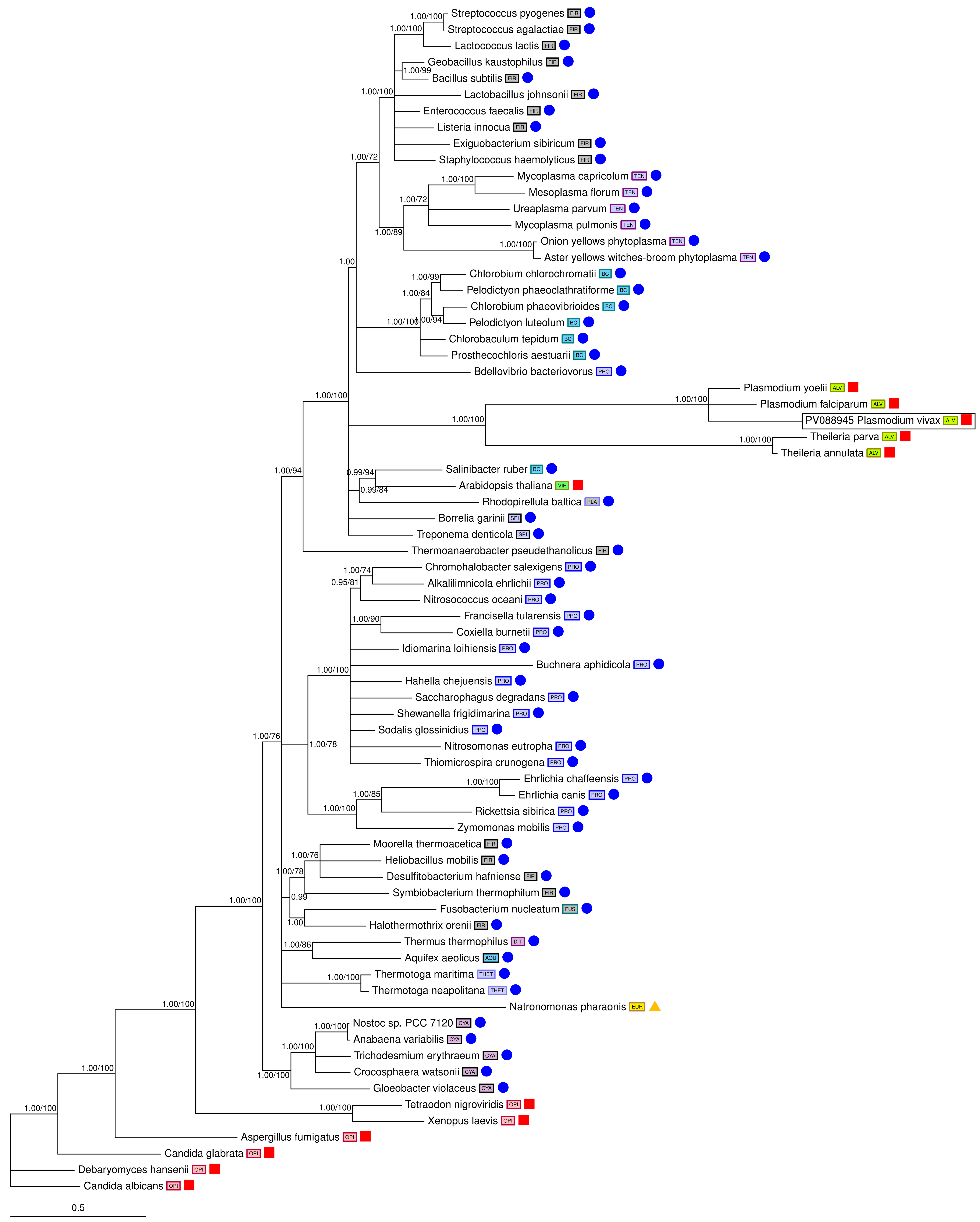

TN199

```
            Candy accession: PV098000
          RefSeq accession: XP_001613260.1
         Uniprot accession: A5KB16_PLAVI
                  Comments: LGT - APICOMPLEXA ONLY + PHAGES
          Species affected: PF,PV,PY,PB,PC
    Adjacent taxa in tree: Bacteria/phage
EC annotation - (Blast/Profile): na
               PHOBIUS SP: Y
              PHOBIUS TMD: 0
         RefSeq annotation: single-strand binding protein
  Name of enzyme/protein: Predicted single stranded DNA binding
                          protein
    KEGG PATHWAY - level 1: Other function
    KEGG PATHWAY - level 2: na
```

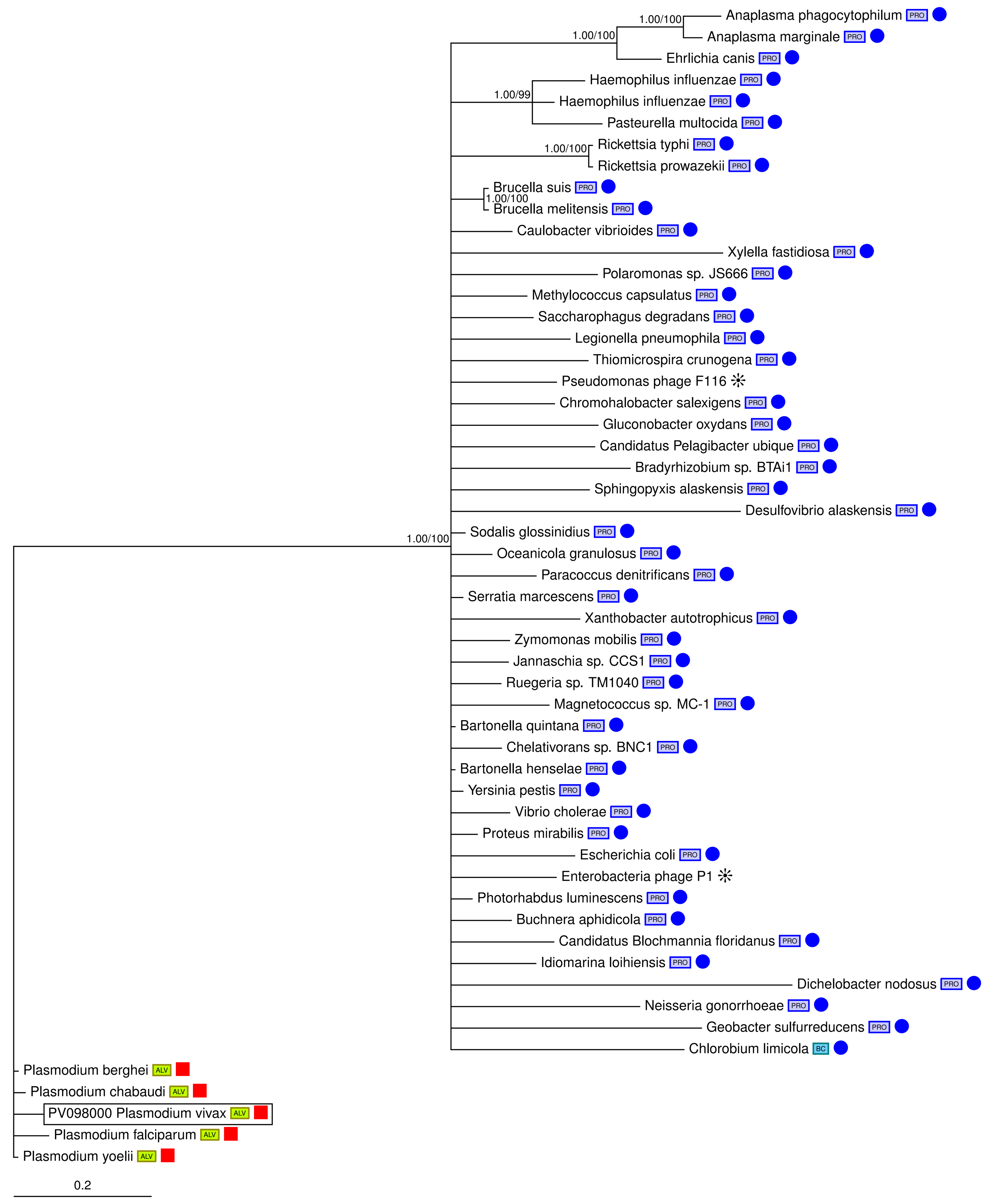

TN200

```
                Candy accession: Q869B9_DICDI
               RefSeq accession: XP_643448.1
              Uniprot accession: Q869B9_DICDI
                       Comments: LGT - DD TWO NODES - INTRACELLULAR
                                 BACTERIA
               Species affected: DD
          Adjacent taxa in tree: Chlamydiae/Verrucomicrobia  -
                                 Protochlamydia
EC annotation - (Blast/Profile): EC:2.4.2.29
                     PHOBIUS SP: 0
                    PHOBIUS TMD: 0
              RefSeq annotation: hypothetical protein DDB_G0275613
         Name of enzyme/protein: tRNA-guanine transglycosylase
         KEGG PATHWAY - level 1: Reaction
         KEGG PATHWAY - level 2: Reaction
```

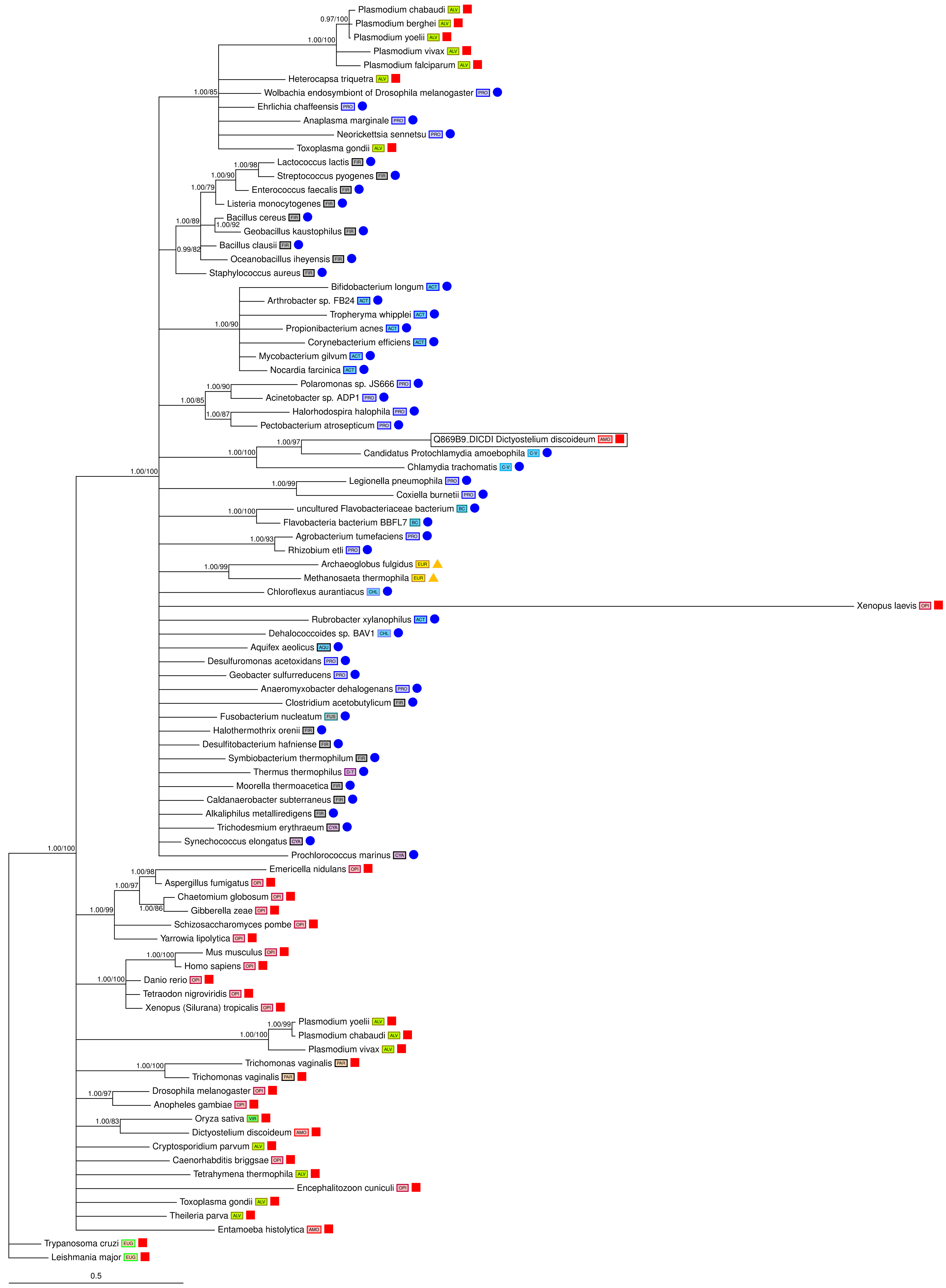

TN201

```
              Candy accession: Q4Q5V2_LEIMA
             RefSeq accession: XP_001685296.1
            Uniprot accession: Q4Q5V2_LEIMA
                     Comments: LGT - LM TWO NODES
             Species affected: LM
       Adjacent taxa in tree: Proteobacteria - Xanthobacter
EC annotation - (Blast/Profile): na
                   PHOBIUS SP: 0
                  PHOBIUS TMD: 0
           RefSeq annotation: hypothetical protein
       Name of enzyme/protein: Protein containing cyclase domain
        KEGG PATHWAY - level 1: Other function
        KEGG PATHWAY - level 2: na
```

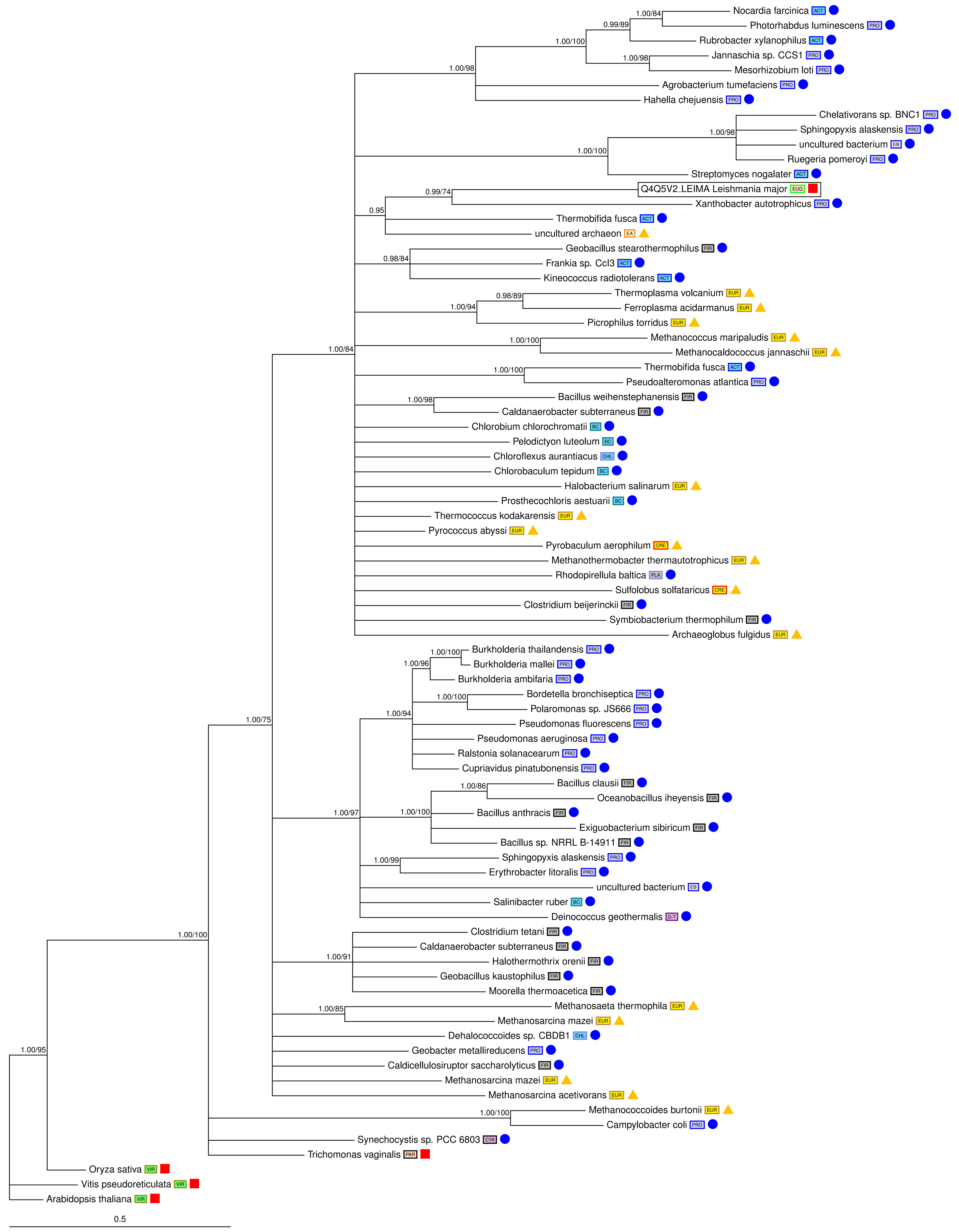

TN202

```
                Candy accession: TV84774084
              RefSeq accession: XP_001310401.1
             Uniprot accession: A2FCA8_TRIVA
                      Comments: LGT - TV TWO NODES
              Species affected: TV
        Adjacent taxa in tree: Firmicutes
EC annotation - (Blast/Profile): EC:2.7.1.12
                   PHOBIUS SP: 0
                  PHOBIUS TMD: 0
            RefSeq annotation: sugar kinase
       Name of enzyme/protein: gluconokinase
       KEGG PATHWAY - level 1: Carbohydrate Metabolism
       KEGG PATHWAY - level 2: Pentose phosphate pathway
```

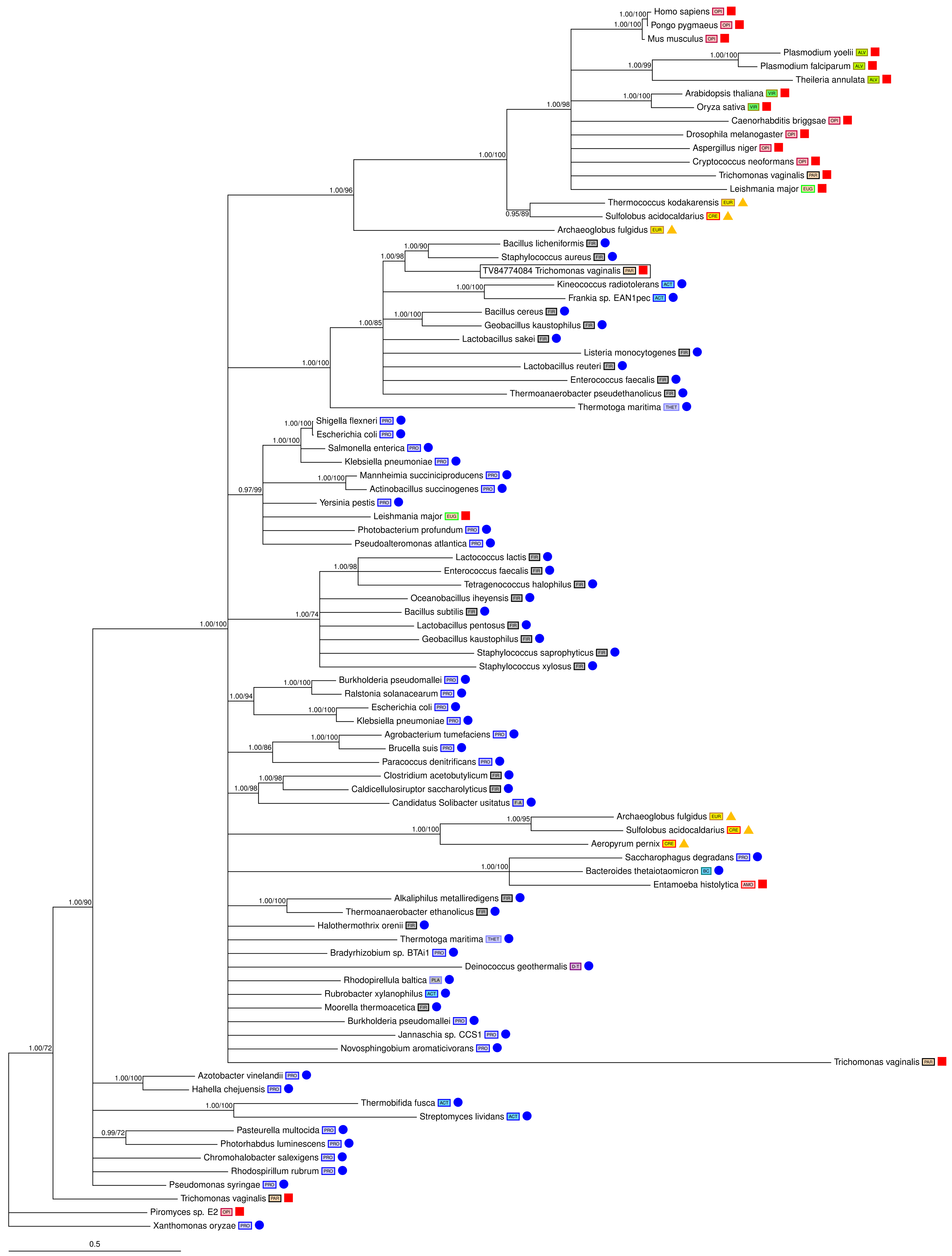

TN203

```
              Candy accession: Q5CYQ7_CRYPV
             RefSeq accession: XP_628323.1
            Uniprot accession: Q5CYQ7_CRYPV
                     Comments: LGT - CRYPTOSPORIDIUM ONLY - 3 PAIRS OF
                               CP+CH PARALOGUES
             Species affected: CP,CH
        Adjacent taxa in tree: Proteobacteria
EC annotation - (Blast/Profile): na
                   PHOBIUS SP: 0
                  PHOBIUS TMD: 6
            RefSeq annotation: hypothetical protein
         Name of enzyme/protein: Putative ABC-type long-chain fatty acid
                               transport system
       KEGG PATHWAY - level 1: Other function - Membrane transport
       KEGG PATHWAY - level 2: na
```

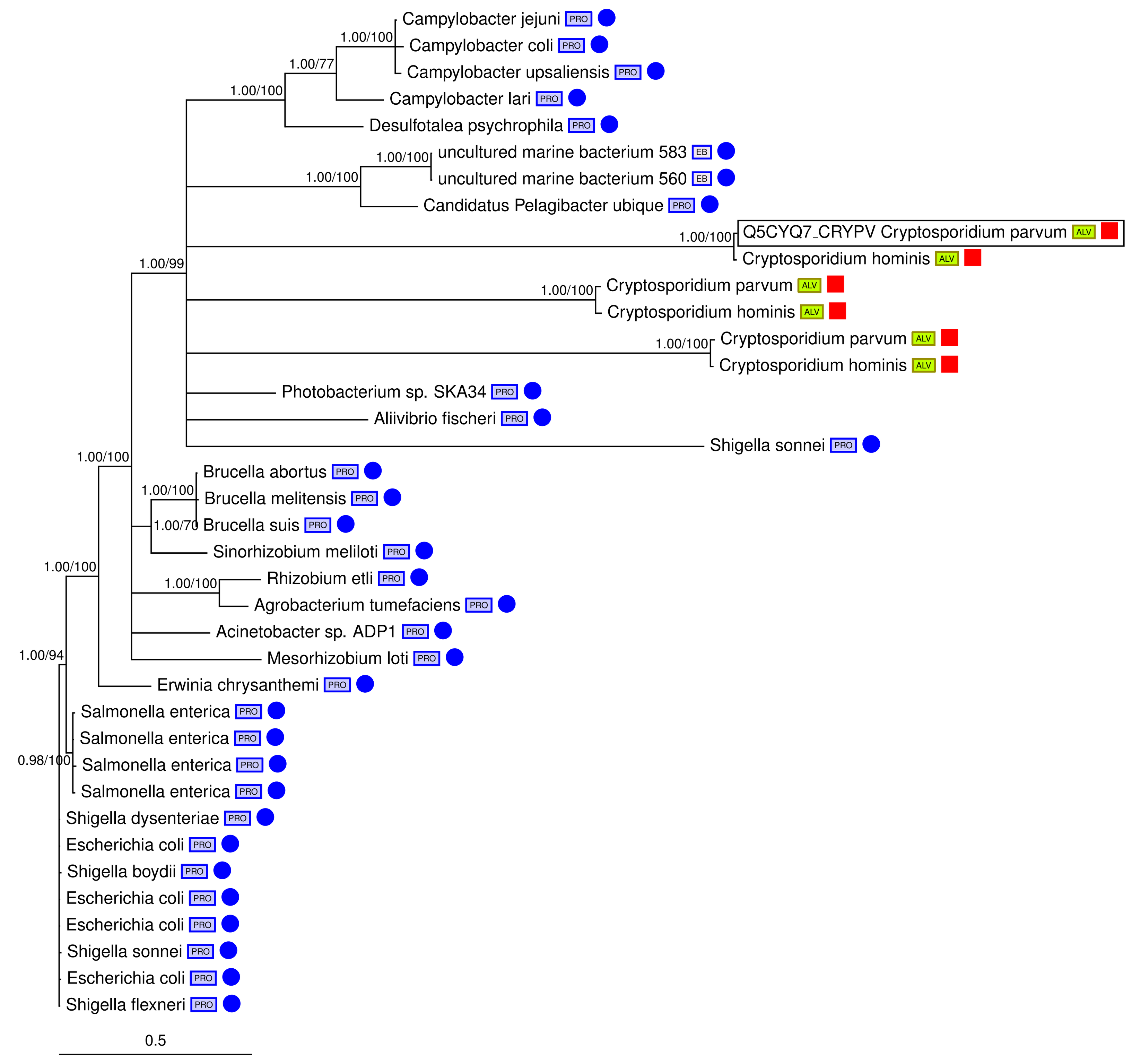

TN204

```
             Candy accession: Q4Q4F2_LEIMA
            RefSeq accession: XP_001685796.1
           Uniprot accession: Q4Q4F2_LEIMA
                    Comments: LGT - KINETOPLASTIDS TWO NODES
            Species affected: LM,TB,TC
       Adjacent taxa in tree: Bacteria
EC annotation - (Blast/Profile): na
                  PHOBIUS SP: 0
                 PHOBIUS TMD: 0
           RefSeq annotation: hypothetical protein
     Name of enzyme/protein: Uncharacterized protein family UPF0027
     KEGG PATHWAY - level 1: Function unknown
     KEGG PATHWAY - level 2: na
```

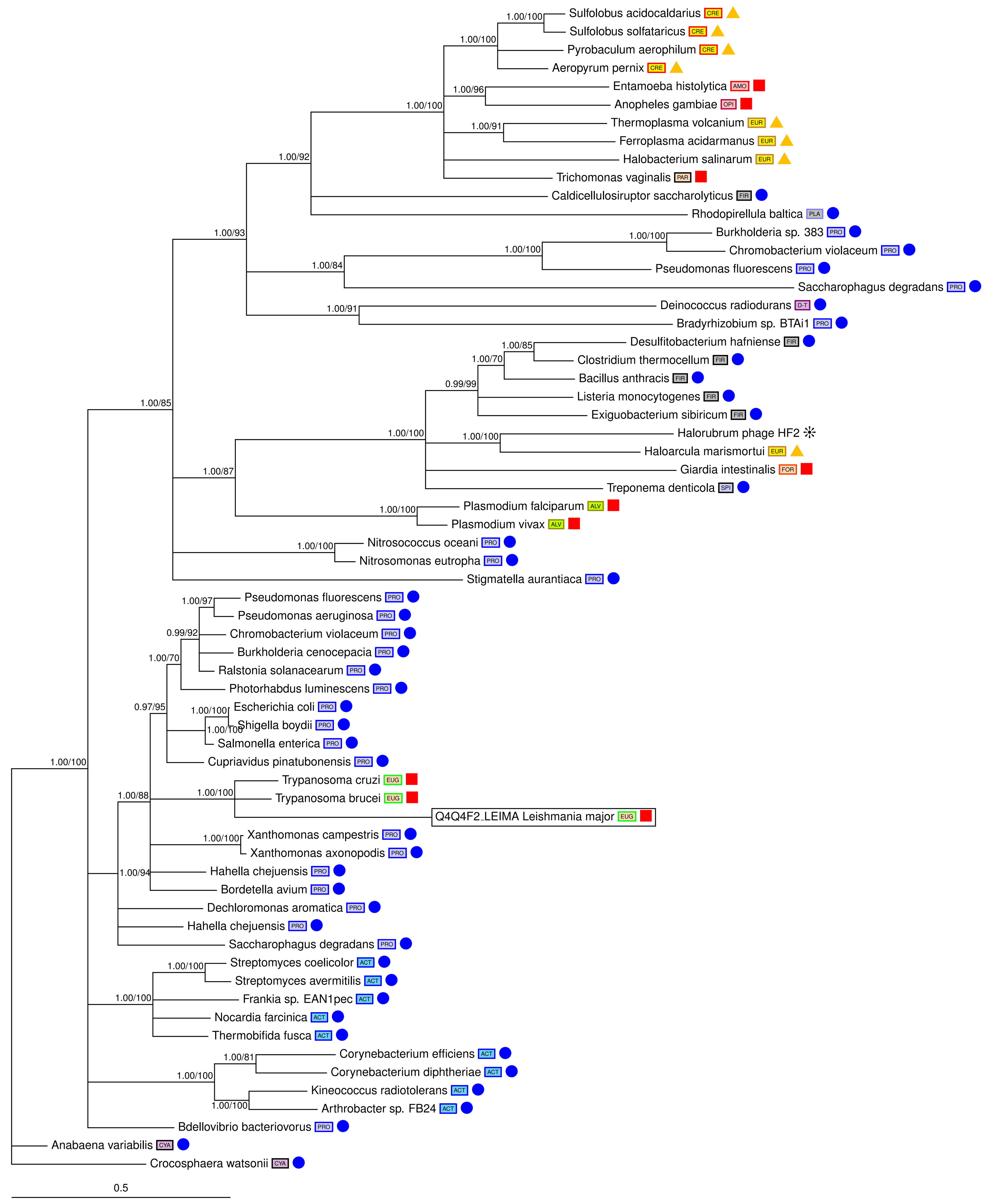

TN205

```
                Candy accession: Q7R1P4_GIALA
               RefSeq accession: XP_001706109.1
              Uniprot accession: A8BMJ3_GIALA
                       Comments: LGT - GI TWO NODES - PHAGES - DEAP LGT
                                 INTO FUNGI
               Species affected: GI,FUNGI
          Adjacent taxa in tree: Proteobacteria
EC annotation - (Blast/Profile): na
                     PHOBIUS SP: 0
                    PHOBIUS TMD: 0
              RefSeq annotation: Phosphatase similar to the C-terminal
                                 domain of histone macroH2A1
         Name of enzyme/protein: Predicted Macro domain, Poa1p-like
                                 family
         KEGG PATHWAY - level 1: Other function
         KEGG PATHWAY - level 2: na
```

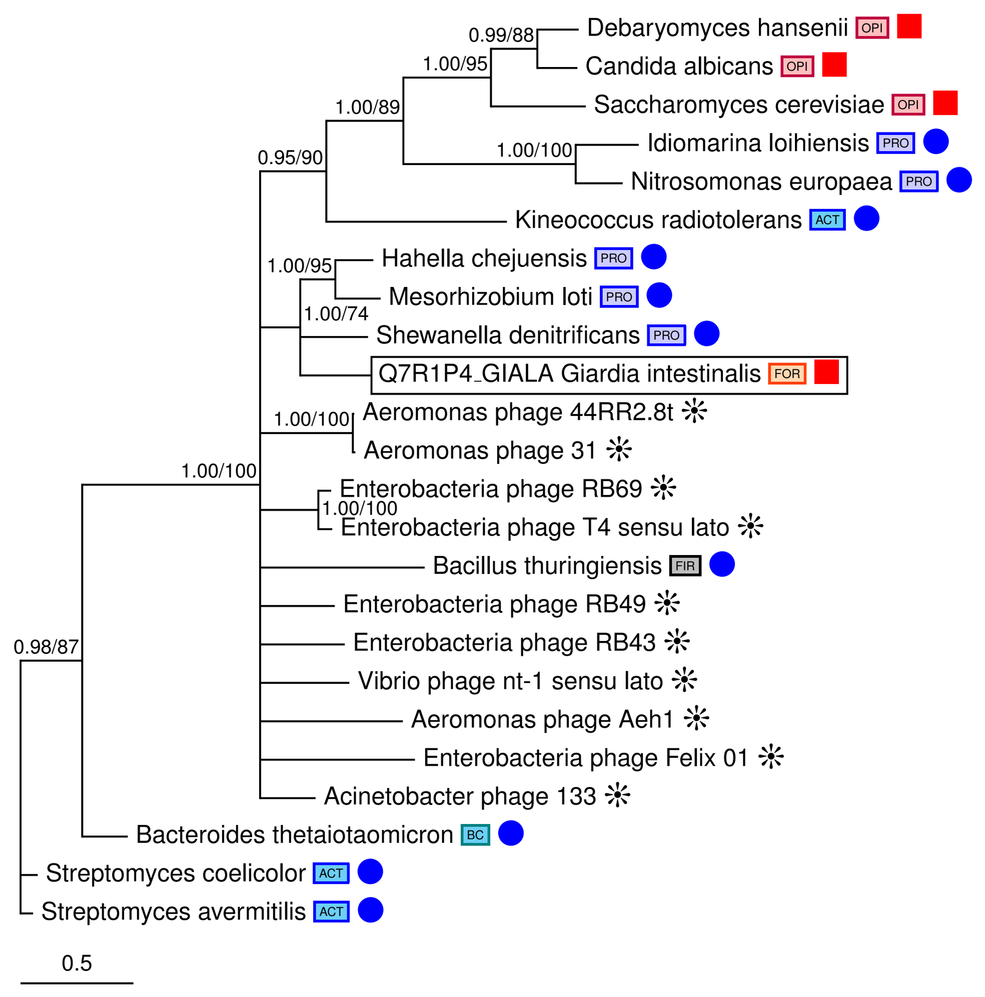

TN206

```
                Candy accession: Q7R6G7_GIALA
               RefSeq accession: XP_001707931.1
              Uniprot accession: A8BCL1_GIALA
                       Comments: LGT - GI ONLY
               Species affected: GI
          Adjacent taxa in tree: Prokaryotes
EC annotation - (Blast/Profile): na
                     PHOBIUS SP: 0
                    PHOBIUS TMD: 0
              RefSeq annotation: Nitroreductase Fd-NR2
       Name of enzyme/protein: Predicted oxidoreductase
       KEGG PATHWAY - level 1: Other function
       KEGG PATHWAY - level 2: na
```

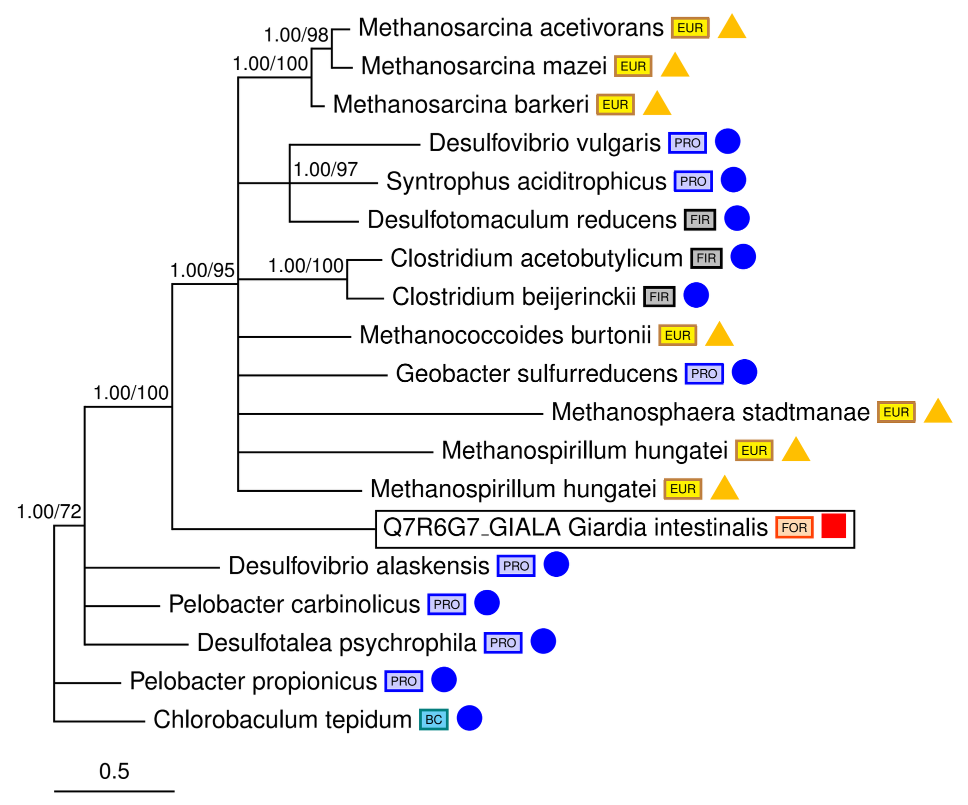

TN207

```
              Candy accession: Q7RSF2_PLAYO
             RefSeq accession: XP_724075.1
            Uniprot accession: Q7RSF2_PLAYO
                     Comments: LGT - APICOMPLEXA ONLY
             Species affected: PF,PV,PY,TG
         Adjacent taxa in tree: Bacteria
EC annotation - (Blast/Profile): na
                   PHOBIUS SP: 0
                  PHOBIUS TMD: 0
            RefSeq annotation: hypothetical protein
        Name of enzyme/protein: Predicted SAM-dependent
                                methyltransferases; 23S rRNA m(5)C1962
                                methyltransferase;
        KEGG PATHWAY - level 1: Other function
        KEGG PATHWAY - level 2: na
```

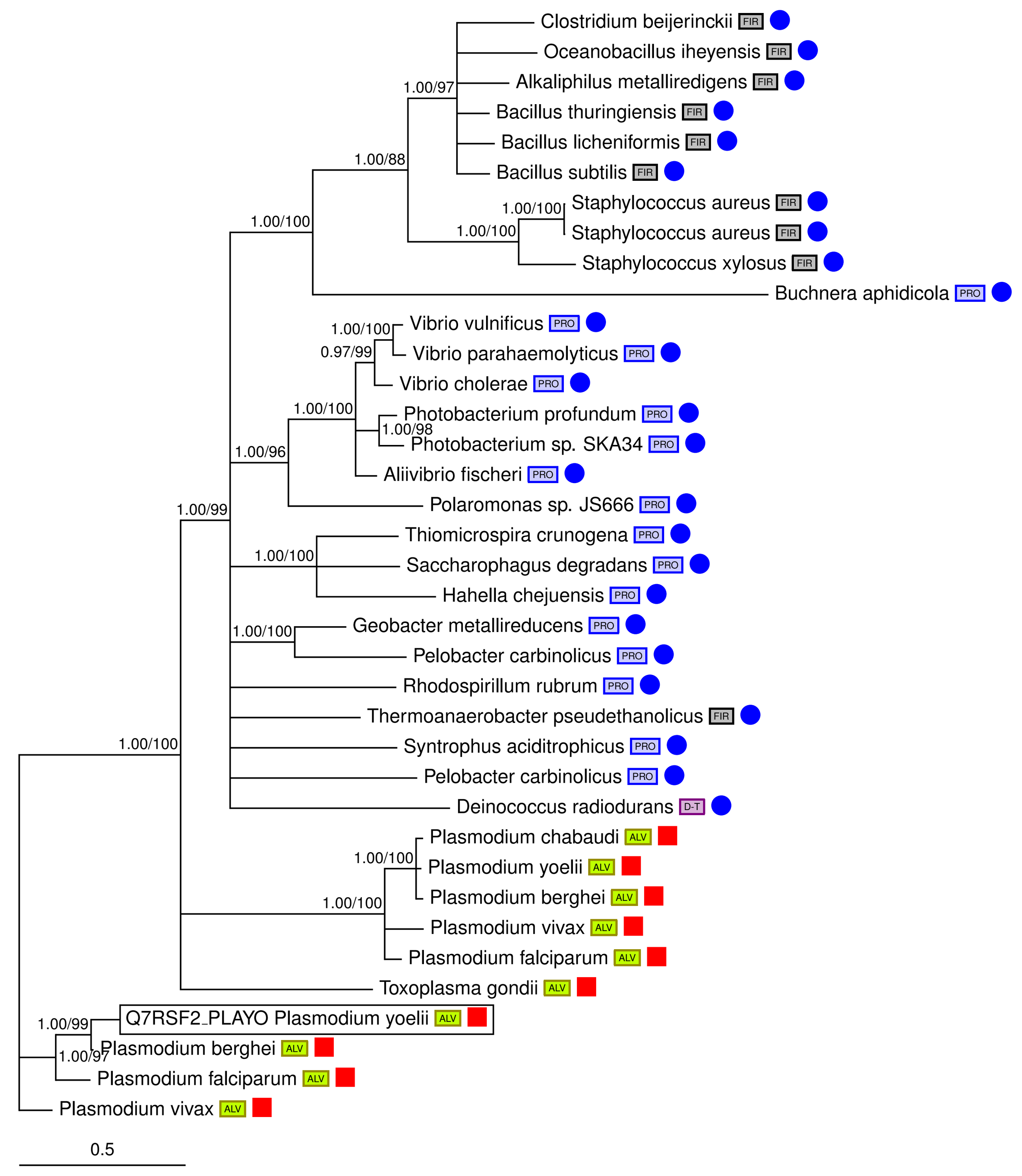

TN208

```
              Candy accession: Q54RW1_DICDI
             RefSeq accession: XP_639376.1
            Uniprot accession: Q54RW1_DICDI
                     Comments: LGT - DD TWO NODES
             Species affected: DD
        Adjacent taxa in tree: Bacteria
EC annotation - (Blast/Profile): EC:3.5.1.16
                   PHOBIUS SP: 0
                  PHOBIUS TMD: 0
            RefSeq annotation: hypothetical protein DDB_G0282881
        Name of enzyme/protein: acetylornithine deacetylase
        KEGG PATHWAY - level 1: Amino Acid Metabolism
        KEGG PATHWAY - level 2: Arginine and proline metabolism

              Candy accession: Q581Z1_9TRYP
             RefSeq accession: XP_847024.1
            Uniprot accession: Q581Z1_9TRYP
                     Comments: LGT - KINETOPLASTIDS TWO NODES
             Species affected: TB,TC,LM
        Adjacent taxa in tree: Proteobacteria
EC annotation - (Blast/Profile): EC:3.5.1.16
                   PHOBIUS SP: 0
                  PHOBIUS TMD: 0
            RefSeq annotation: acetylornithine deacetylase
        Name of enzyme/protein: acetylornithine deacetylase
        KEGG PATHWAY - level 1: Amino Acid Metabolism
        KEGG PATHWAY - level 2: Arginine and proline metabolism
```

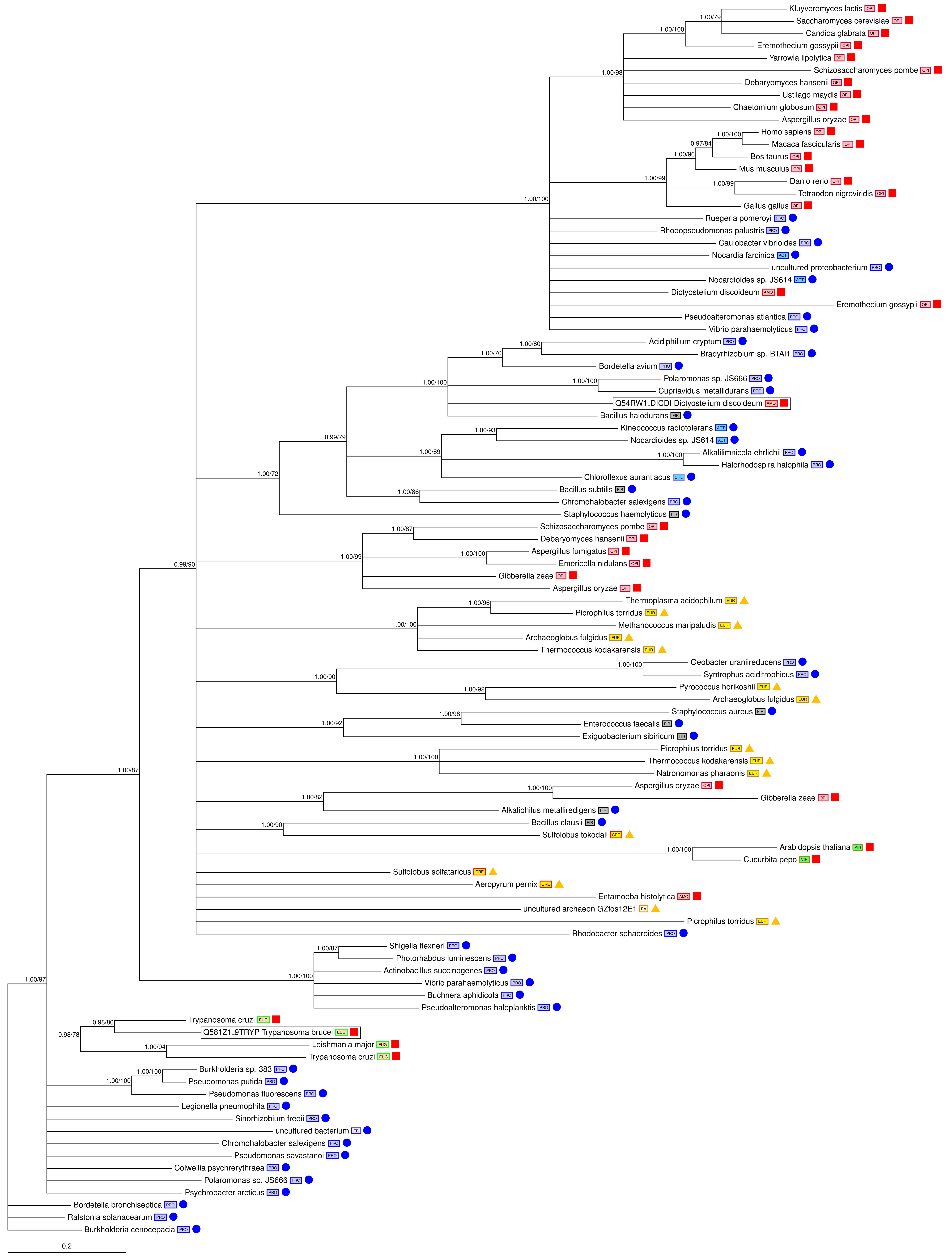

TN209

```
                Candy accession: Q51F02_ENTHI
               RefSeq accession: XP_656821.2
              Uniprot accession: C4M5A2_ENTHI
                       Comments: LGT - EH ONLY
               Species affected: EH
          Adjacent taxa in tree: Bacteria
EC annotation - (Blast/Profile): na
                     PHOBIUS SP: 0
                    PHOBIUS TMD: 0
              RefSeq annotation: ser/thr protein phosphatase family
                                 protein
         Name of enzyme/protein: Predicted phosphohydrolase
         KEGG PATHWAY - level 1: Other function
         KEGG PATHWAY - level 2: na
```

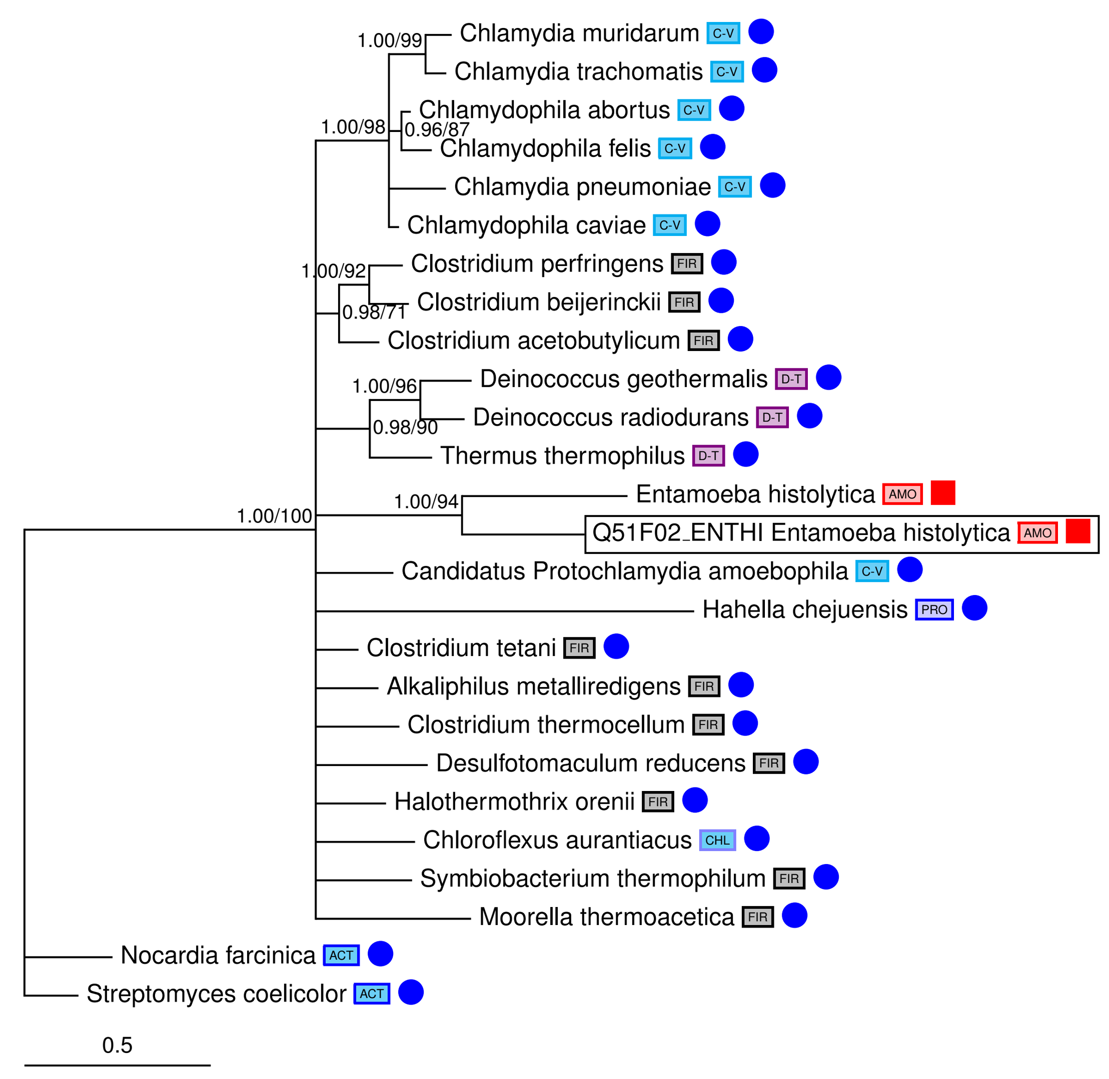

TN210

```
             Candy accession: Q55EN7_DICDI
            RefSeq accession: XP_646983.1
           Uniprot accession: Q55EN7_DICDI
                    Comments: LGT - DD TWO NODES
            Species affected: DD
         Adjacent taxa in tree: Bacteria
EC annotation - (Blast/Profile): EC:3.6.3.2
                  PHOBIUS SP: 0
                 PHOBIUS TMD: 8
           RefSeq annotation: transmembrane protein
    Name of enzyme/protein: Mg2+-importing ATPase
      KEGG PATHWAY - level 1: Reaction
      KEGG PATHWAY - level 2: Reaction
```

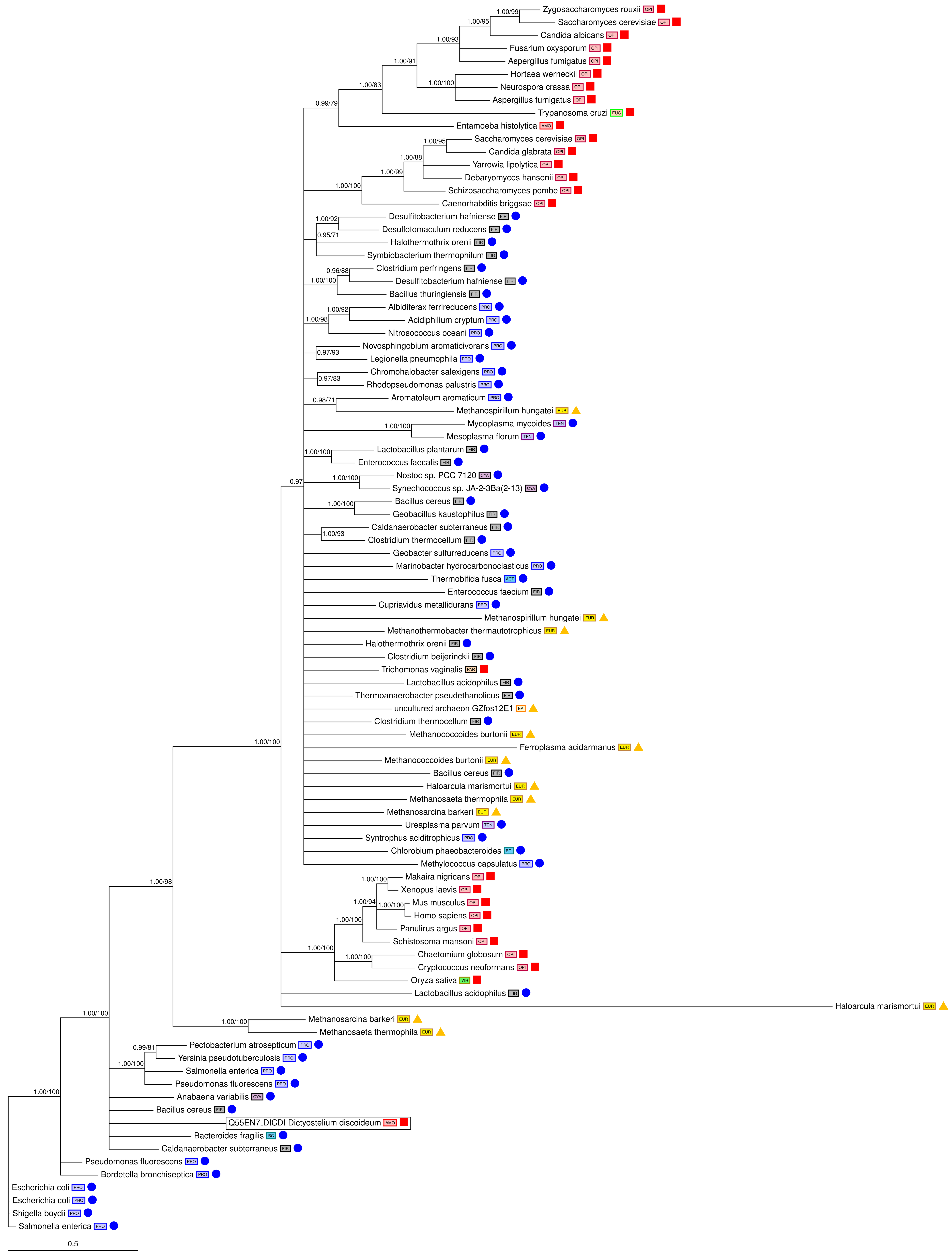

TN211

```
          Candy accession: Q54EK1_DICDI
         RefSeq accession: XP_635239.1
        Uniprot accession: Q54EK1_DICDI
                 Comments: LGT - DD TWO NODES
         Species affected: DD
   Adjacent taxa in tree: Proteobacteria
EC annotation - (Blast/Profile): na
              PHOBIUS SP: 0
             PHOBIUS TMD: 0
        RefSeq annotation: esterase/lipase/thioesterase domain-
                           containing protein
    Name of enzyme/protein: Protein contianing alpha/beta hydrolase
                           domain
    KEGG PATHWAY - level 1: Other function
    KEGG PATHWAY - level 2: na
```

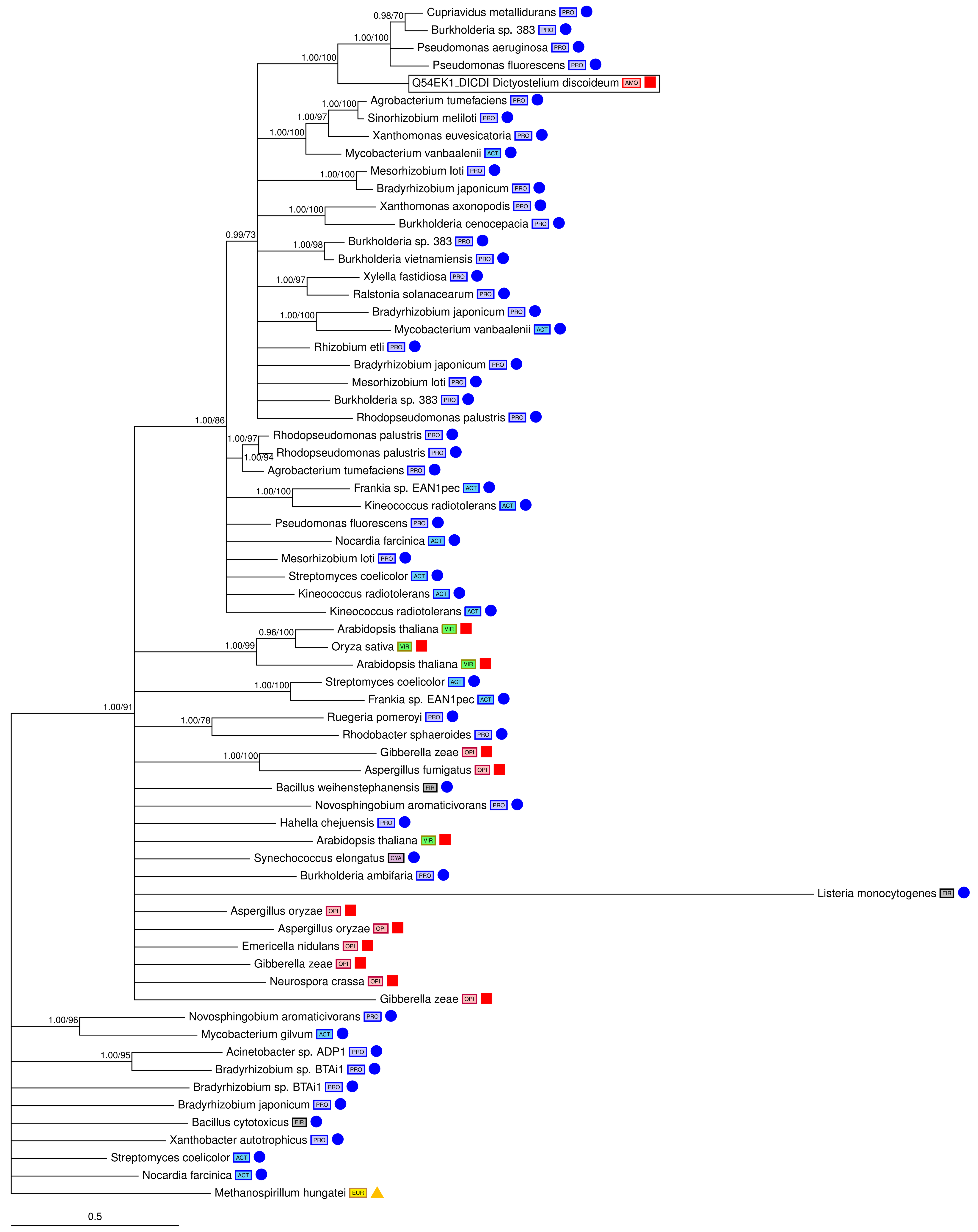

TN212

```
            Candy accession: TV84653053
           RefSeq accession: XP_001306442.1
          Uniprot accession: A2FNL2_TRIVA
                   Comments: LGT - TV TWO NODES
           Species affected: TV
     Adjacent taxa in tree: Bacteria
EC annotation - (Blast/Profile): EC:3.5.3.1
                 PHOBIUS SP: 0
                PHOBIUS TMD: 0
          RefSeq annotation: Arginase family protein
    Name of enzyme/protein: Arginase
    KEGG PATHWAY - level 1: Amino Acid Metabolism
    KEGG PATHWAY - level 2: Arginine and proline metabolism

            Candy accession: Q54HV8_DICDI
           RefSeq accession: XP_636331.1
          Uniprot accession: Q54HV8_DICDI
                   Comments: LGT - DD TWO NODES
           Species affected: DD
     Adjacent taxa in tree: Proteobacteria - Xylella
EC annotation - (Blast/Profile): EC:3.5.3.1
                 PHOBIUS SP: 0
                PHOBIUS TMD: 0
          RefSeq annotation: arginase/agmatinase/formiminoglutamase
                             family protein
    Name of enzyme/protein: Arginase
    KEGG PATHWAY - level 1: Amino Acid Metabolism
    KEGG PATHWAY - level 2: Arginine and proline metabolism
```

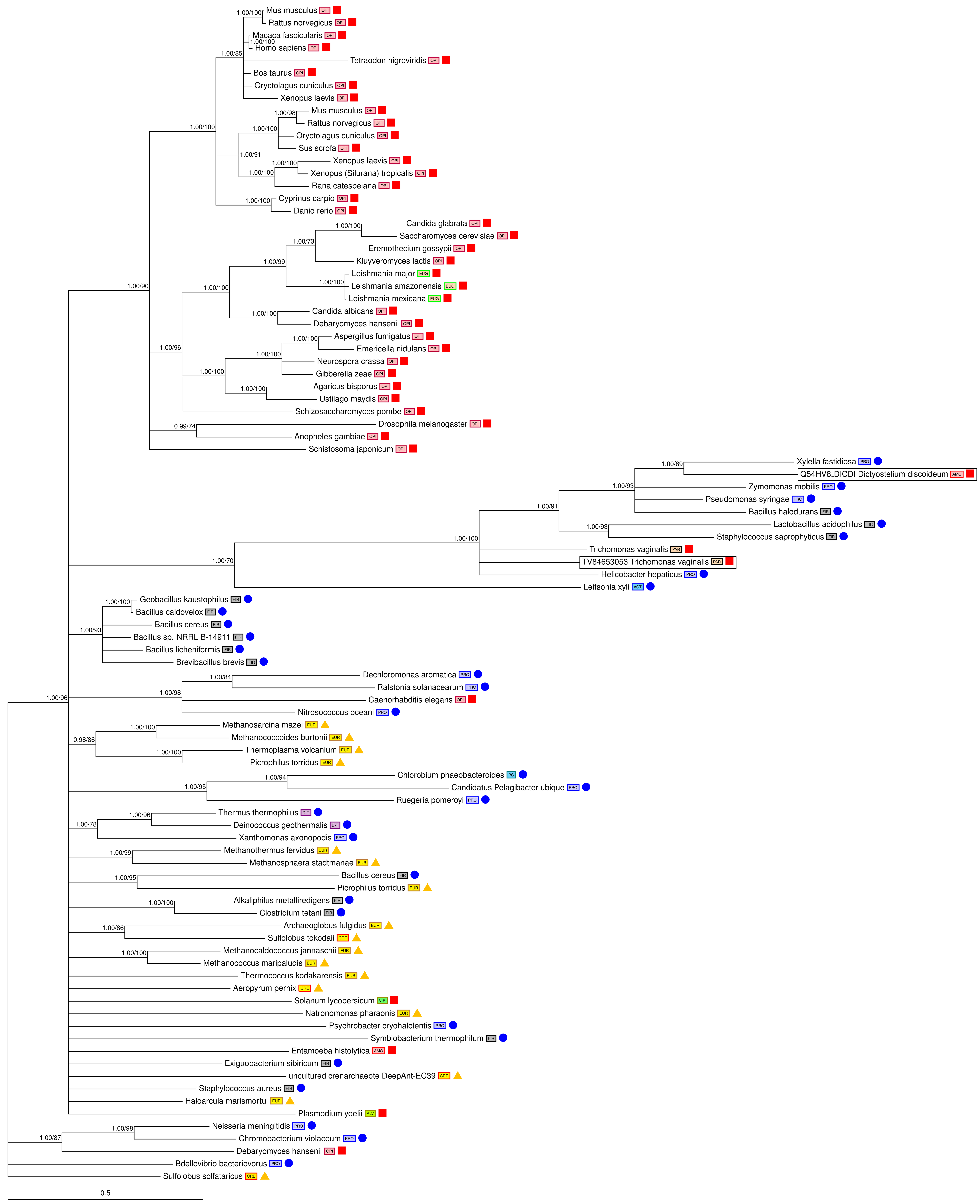

TN213

Candy accession: Q54IU8_DICDI
RefSeq accession: XP_636690.1
Uniprot accession: ARY2_DICDI
Comments: LGT - DD TWO NODES
Species affected: DD
Adjacent taxa in tree: Firmicutes - Bacillus
EC annotation - (Blast/Profile): EC:2.3.1.5
PHOBIUS SP: 0
PHOBIUS TMD: 0
RefSeq annotation: arylamine N-acetyltransferase family protein
Name of enzyme/protein: arylamine N-acetyltransferase
KEGG PATHWAY - level 1: Biosynthesis of Other Secondary Metabolites, Xenobiotics Biodegradation and Metabolism,
KEGG PATHWAY - level 2: Caffeine metabolism, Nitrotoluene degradation, Drug metabolism - other enzymes

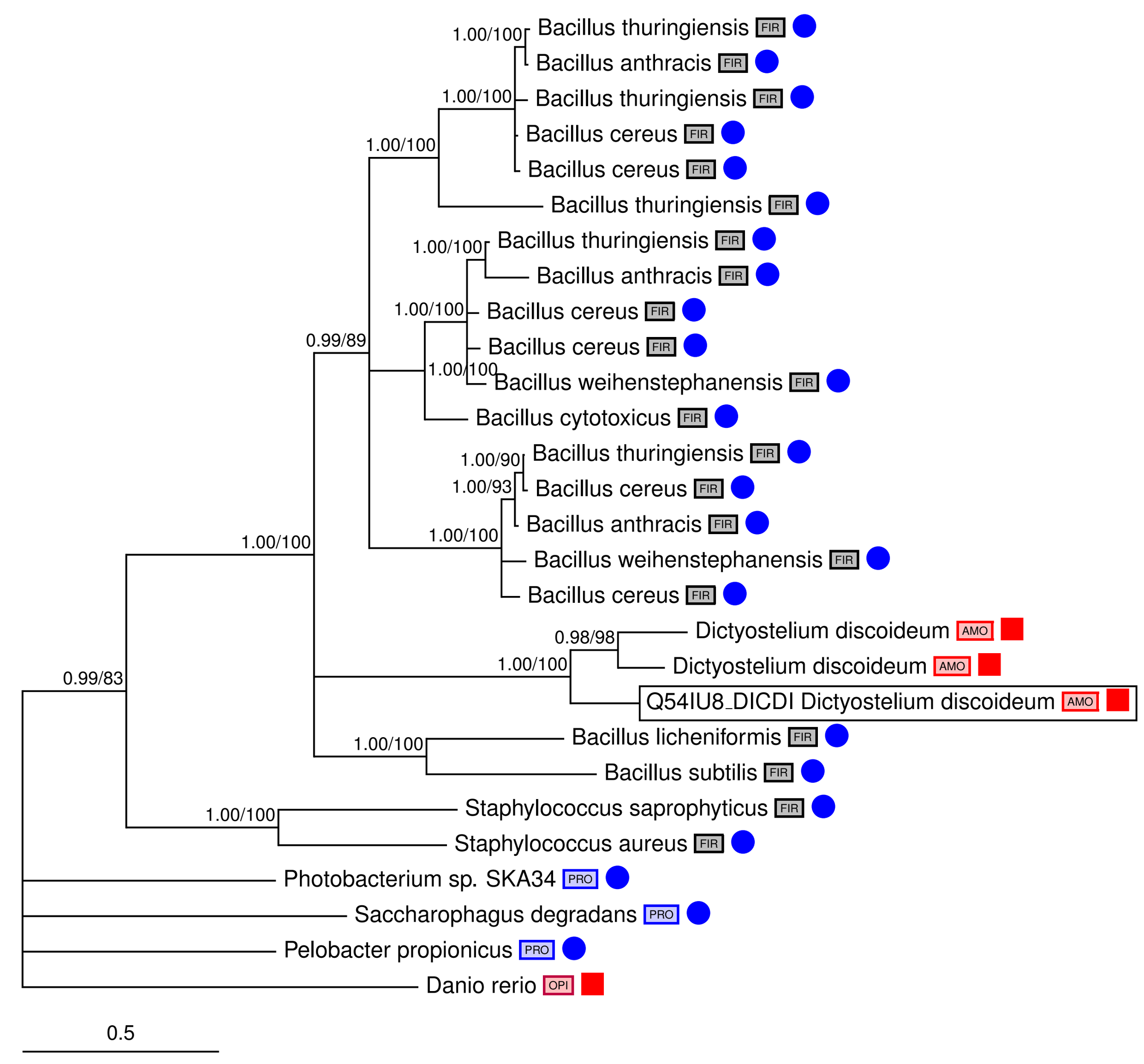

TN214

Candy accession: Q4Q4A9_LEIMA
RefSeq accession: XP_001685839.1
Uniprot accession: Q4Q4A9_LEIMA
Comments: LGT - TV TWO NODES
Species affected: LM, TC
Adjacent taxa in tree: Bacteria
EC annotation - (Blast/Profile): EC:1.3.1.34
PHOBIUS SP: 0
PHOBIUS TMD: 0
RefSeq annotation: oxidoreductase, FAD/FMN-binding family protein
Name of enzyme/protein: NADPH dehydrogenase
KEGG PATHWAY - level 1: Reaction
KEGG PATHWAY - level 2: Reaction

Candy accession: TV85405054
RefSeq accession: XP_001303632.1
Uniprot accession: A2FWL7_TRIVA
Comments: LGT - TV TWO NODES
Species affected: TV
Adjacent taxa in tree: Prokaryotes
EC annotation - (Blast/Profile): EC:1.6.99.1
PHOBIUS SP: 0
PHOBIUS TMD: 0
RefSeq annotation: oxidoreductase, FAD/FMN-binding family protein
Name of enzyme/protein: NADPH dehydrogenase
KEGG PATHWAY - level 1: Reaction
KEGG PATHWAY - level 2: Reaction

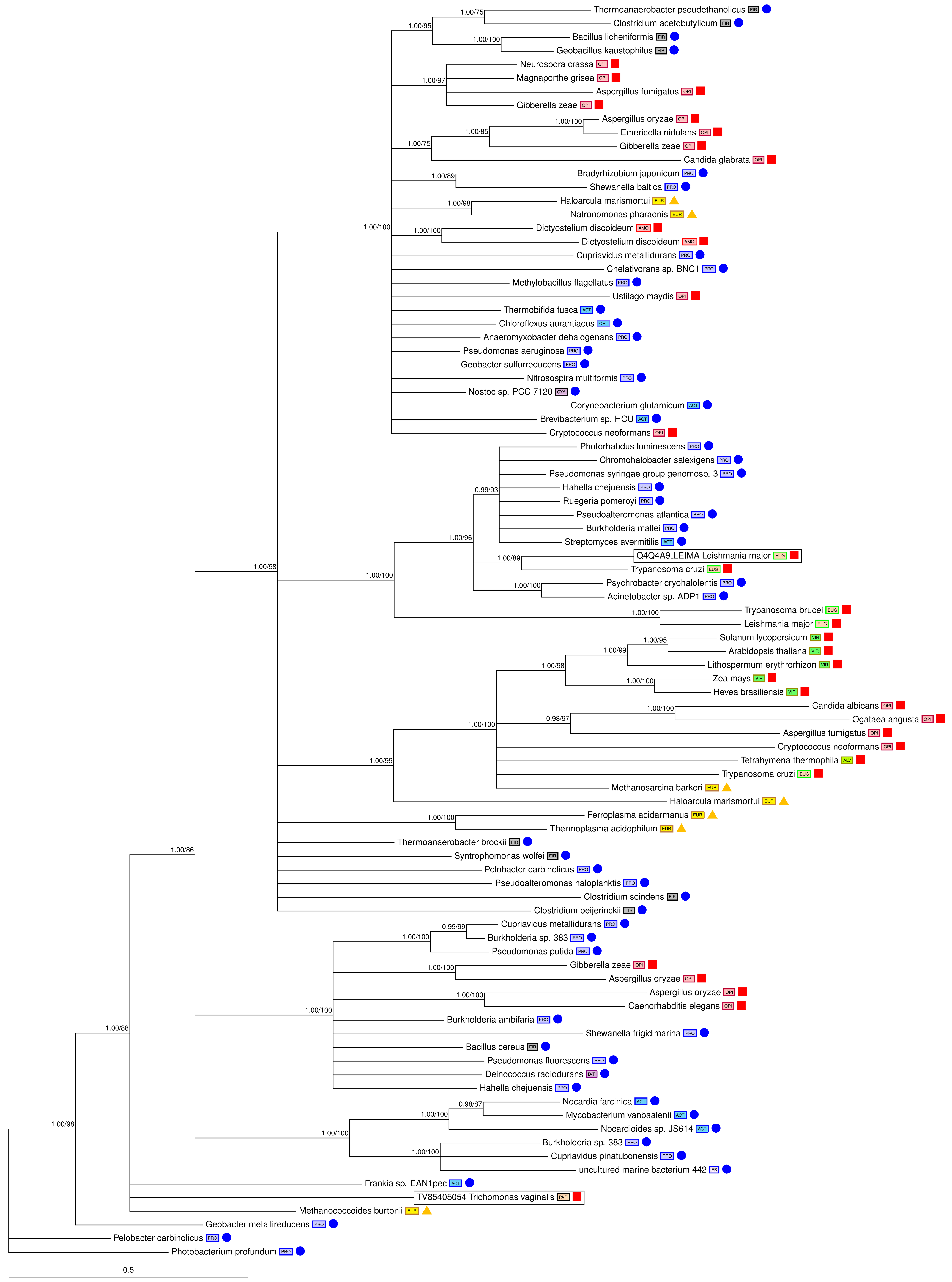

TN215

```
               Candy accession: Q55DP5_DICDI
              RefSeq accession: XP_646086.1
             Uniprot accession: Q55DP5_DICDI
                      Comments: LGT - DD ONLY
              Species affected: DD
         Adjacent taxa in tree: Bacteria
EC annotation - (Blast/Profile): na
                    PHOBIUS SP: 0
                   PHOBIUS TMD: 1
             RefSeq annotation: hypothetical protein DDB_G0270564
        Name of enzyme/protein: Predicted RNA methyltransferase
        KEGG PATHWAY - level 1: Other function - Genetic Information
                                Processing
        KEGG PATHWAY - level 2: na
```

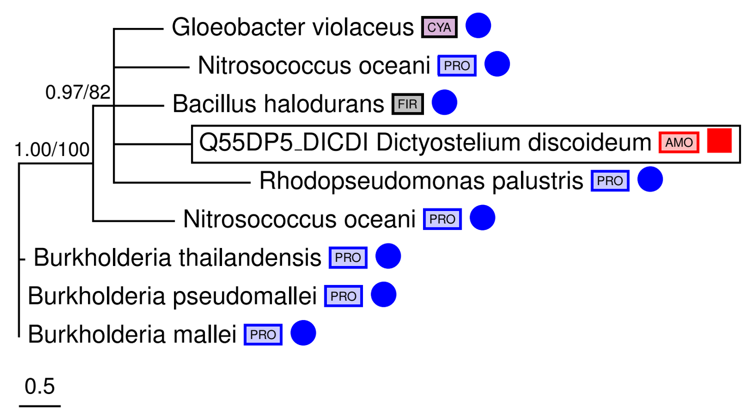

TN216

```
                Candy accession: Q55DT1_DICDI
               RefSeq accession: XP_646050.1
              Uniprot accession: Q55DT1_DICDI
                       Comments: LGT - DD ONLY
               Species affected: DD
          Adjacent taxa in tree: Bacteria
EC annotation - (Blast/Profile): na
                     PHOBIUS SP: 0
                    PHOBIUS TMD: 0
              RefSeq annotation: hypothetical protein DDB_G0269538
         Name of enzyme/protein: Predicted NTF2-like protein
         KEGG PATHWAY - level 1: Function unknown
         KEGG PATHWAY - level 2: na
```

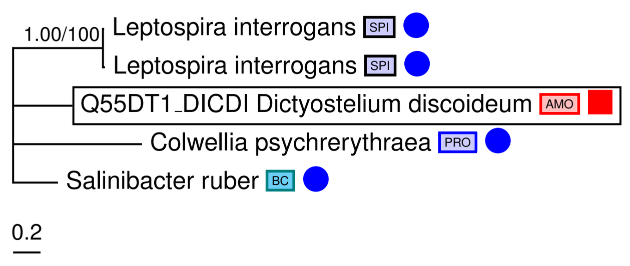

TN217

```
              Candy accession: Q57XC8_9TRYP
            RefSeq accession: XP_847094.1
           Uniprot accession: Q57XC8_9TRYP
                    Comments: LGT - KINETOPLASTIDS TWO NODES
             Species affected: LM,TB
      Adjacent taxa in tree: Protobacteria
EC annotation - (Blast/Profile): EC:2.1.1.14
                  PHOBIUS SP: 0
                 PHOBIUS TMD: 0
           RefSeq annotation: 5-methyltetrahydropteroyltriglutamate--
                              homocysteine S-methyltransferase
        Name of enzyme/protein: 5-methyltetrahydropteroyltriglutamate--
                              homocysteine S-methyltransferase
        KEGG PATHWAY - level 1: Amino Acid Metabolism, Metabolism of
                              Other Amino Acids
        KEGG PATHWAY - level 2: Cysteine and methionine metabolism,
                              Selenocompound metabolism
```

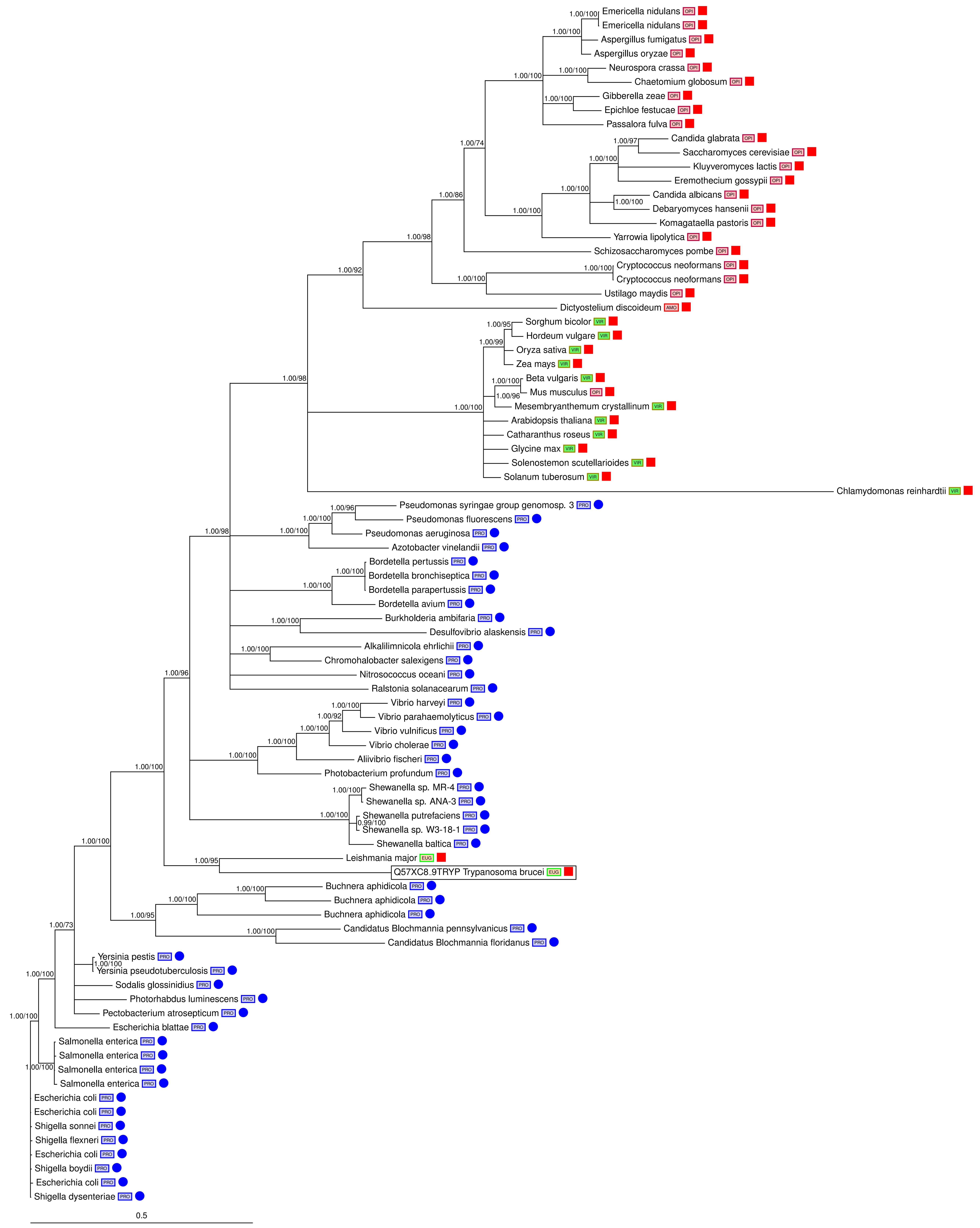

TN218

Candy accession: Q54VL6_DICDI
RefSeq accession: XP_641339.1
Uniprot accession: Q54VL6_DICDI
Comments: LGT - DD TWO NODES
Species affected: DD
Adjacent taxa in tree: Firmicutes - Clostridium
EC annotation - (Blast/Profile): EC:2.3.1.128
PHOBIUS SP: 0
PHOBIUS TMD: 0
RefSeq annotation: hypothetical protein DDB_G0280265
Name of enzyme/protein: ribosomal-protein-alanine N-acetyltransferase
KEGG PATHWAY - level 1: Reaction
KEGG PATHWAY - level 2: Reaction

Candy accession: Q869N8_DICDI
RefSeq accession: XP_645442.1
Uniprot accession: Q869N8_DICDI
Comments: LGT - DD TWO NODES
Species affected: DD
Adjacent taxa in tree: Proteobacteria
EC annotation - (Blast/Profile): EC:2.3.1.128
PHOBIUS SP: 0
PHOBIUS TMD: 0
RefSeq annotation: hypothetical protein DDB_G0271854
Name of enzyme/protein: ribosomal-protein-alanine N-acetyltransferase
KEGG PATHWAY - level 1: Reaction
KEGG PATHWAY - level 2: Reaction

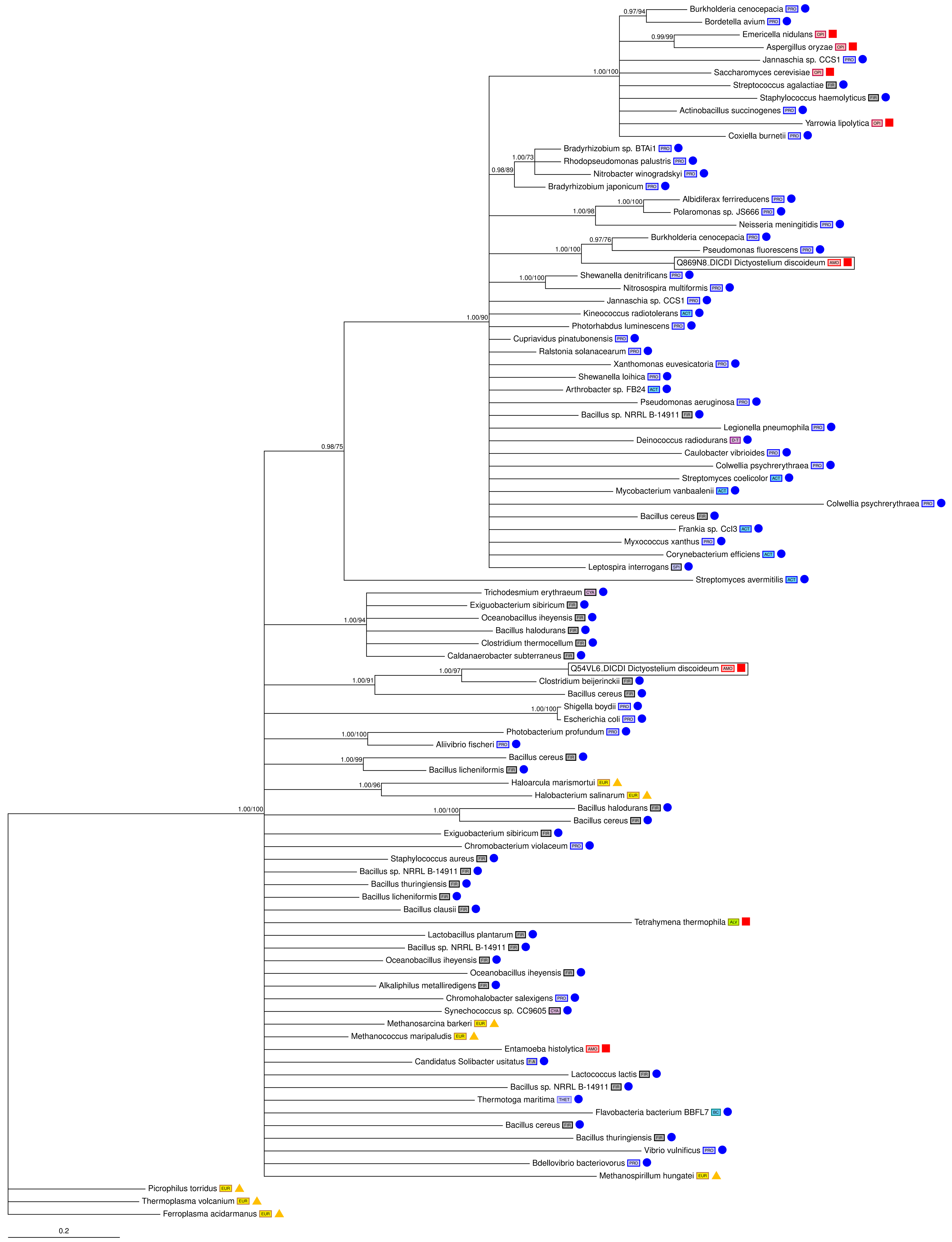

TN219

Candy accession: T72_00687
RefSeq accession: XP_002371464.1
Uniprot accession: B6KUE1_TOXGO
Comments: LGT - TG,PLASMODIUM TWO NODES, DEAP LGT INTO APICOMPLEXA
Species affected: PF,PV,TG
Adjacent taxa in tree: Bacteria
EC annotation - (Blast/Profile): EC:4.1.1.18
PHOBIUS SP: 0
PHOBIUS TMD: 0
RefSeq annotation: lysine decarboxylase, putative
Name of enzyme/protein: Lysine decarboxylase
KEGG PATHWAY - level 1: Amino Acid Metabolism, Biosynthesis of Other Secondary Metabolites
KEGG PATHWAY - level 2: Lysine degradation, Tropane, piperidine and pyridine alkaloid biosynthesis

Candy accession: Q50P27_ENTHI
RefSeq accession: XP_648734.2
Uniprot accession: C4LXC0_ENTHI
Comments: LGT - TG,PLASMODIUM TWO NODES, DEAP LGT INTO APICOMPLEXA
Species affected: EH
Adjacent taxa in tree: Proteobacteria
EC annotation - (Blast/Profile): EC:4.1.1.18
PHOBIUS SP: 0
PHOBIUS TMD: 0
RefSeq annotation: Orn/Arg/Lys decarboxylase
Name of enzyme/protein: Lysine decarboxylase
KEGG PATHWAY - level 1: Amino Acid Metabolism, Biosynthesis of Other Secondary Metabolites
KEGG PATHWAY - level 2: Lysine degradation, Tropane, piperidine and pyridine alkaloid biosynthesis

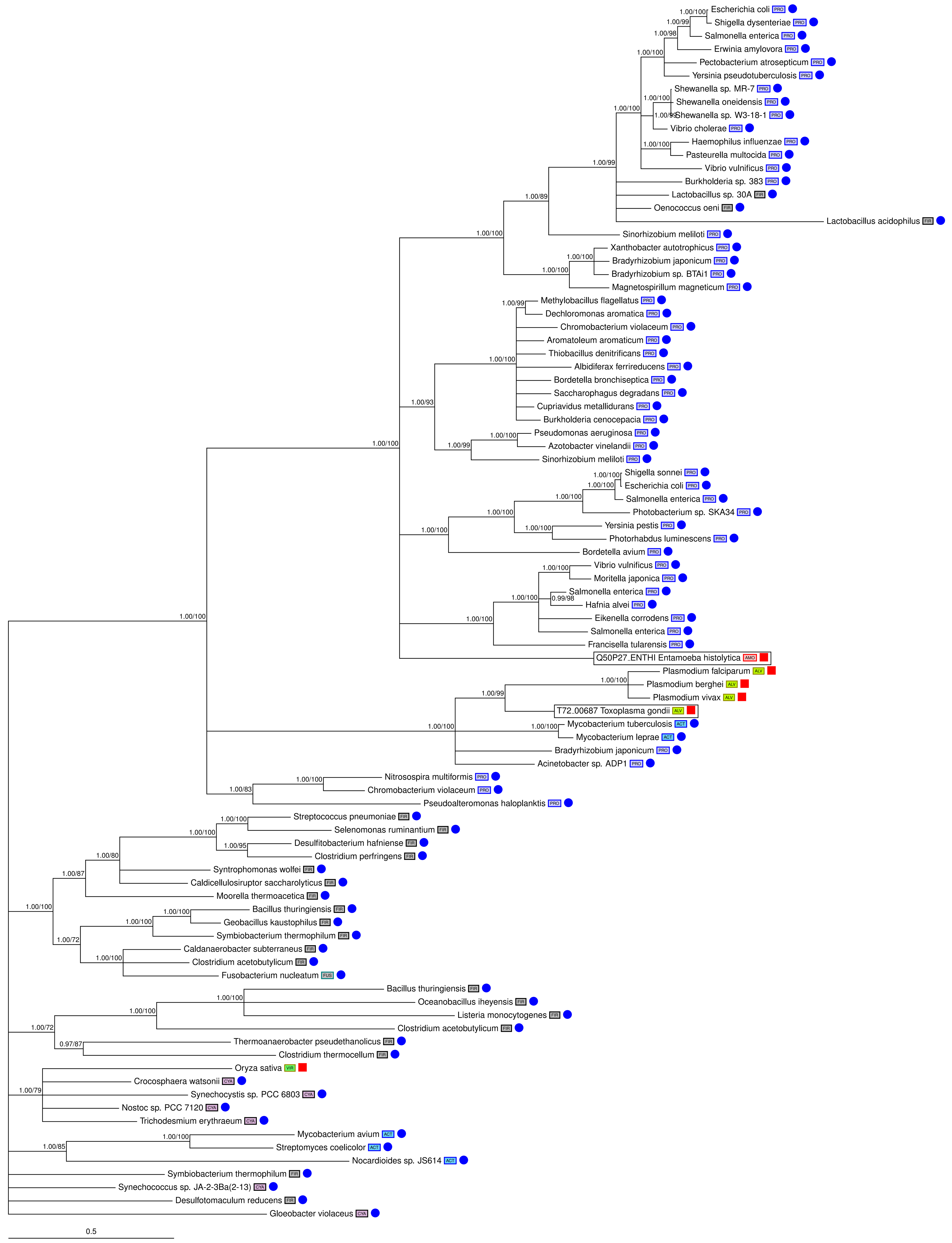

TN220

Candy accession: TV80444129
RefSeq accession: XP_001317907.1
Uniprot accession: A2ENQ8_TRIVA
Comments: LGT - TV TWO NODES + LGT INTO FUNGI
Species affected: TV, FUNGI
Adjacent taxa in tree: Bacteria
EC annotation - (Blast/Profile): EC:4.2.1.1
PHOBIUS SP: 0
PHOBIUS TMD: 0
RefSeq annotation: Carbonic anhydrase family protein
Name of enzyme/protein: Carbonic anhydrase
KEGG PATHWAY - level 1: Energy Metabolism
KEGG PATHWAY - level 2: Nitrogen metabolism

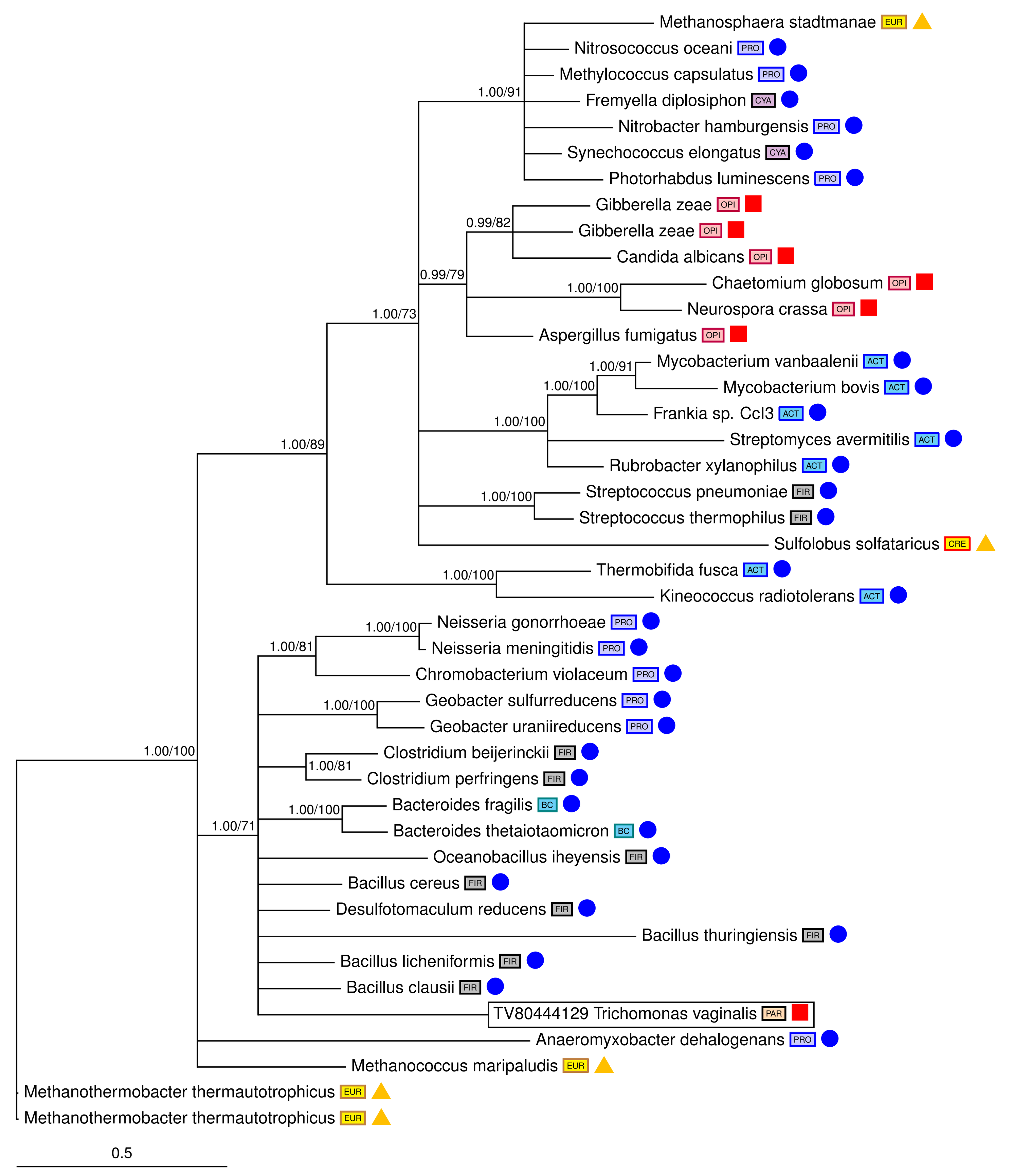

TN221

```
            Candy accession: TV80724024
           RefSeq accession: XP_001300299.1
          Uniprot accession: A2G653_TRIVA
                   Comments: LGT - TV TWO NODES + LGT INTO FUNGI
           Species affected: TV, FUNGI
     Adjacent taxa in tree: Firmicutes - Clostridium
EC annotation - (Blast/Profile): EC:3.2.1.17
                PHOBIUS SP: 0
               PHOBIUS TMD: 0
          RefSeq annotation: glycosyl hydrolase
  Name of enzyme/protein: lysozyme
   KEGG PATHWAY - level 1: Reaction
   KEGG PATHWAY - level 2: Reaction
```

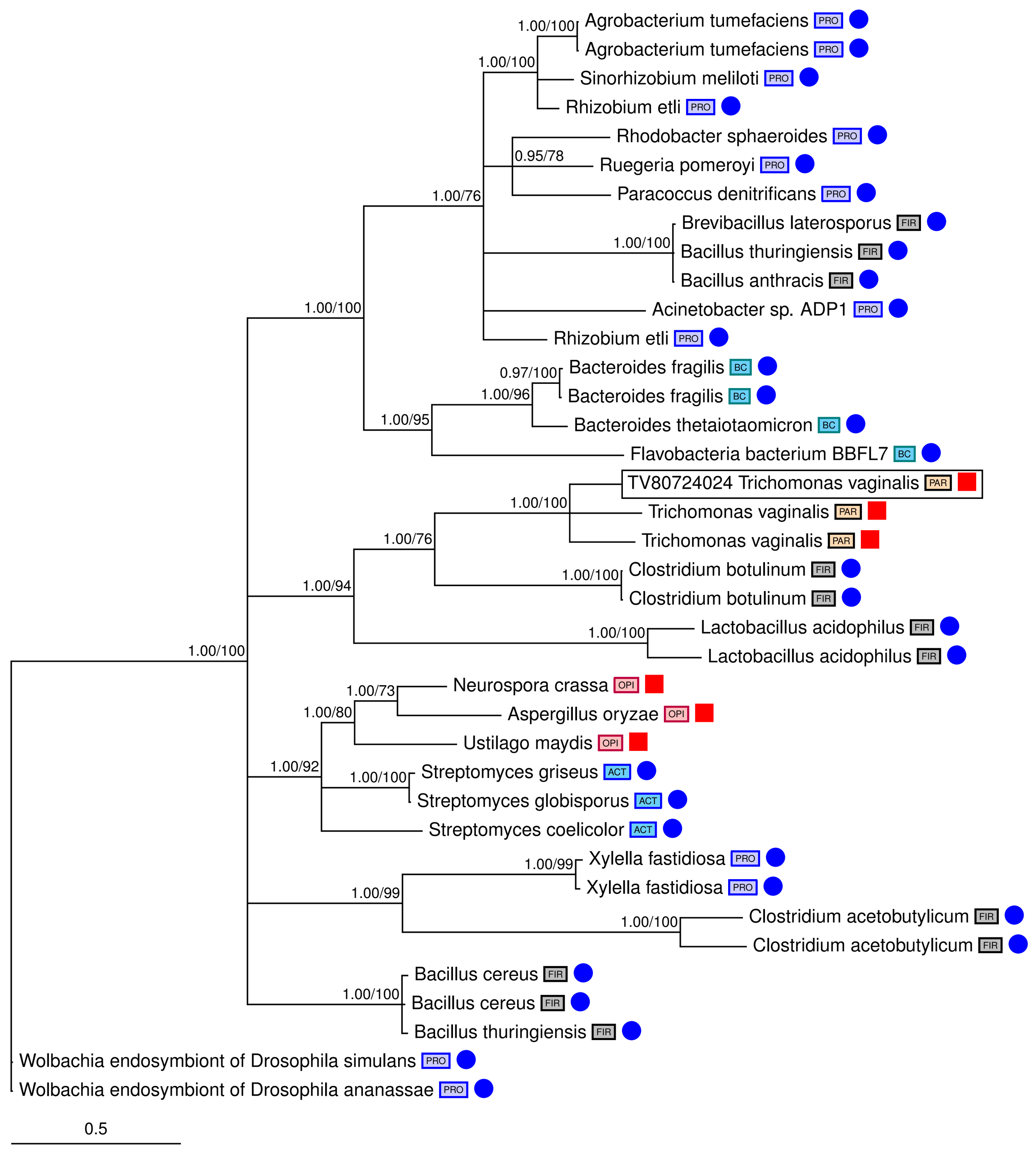

TN222

```
              Candy accession: TV80792013
             RefSeq accession: XP_001579050.1
            Uniprot accession: A2DN76_TRIVA
                     Comments: LGT - TV ONLY
             Species affected: TV
        Adjacent taxa in tree: Bacteria
EC annotation - (Blast/Profile): na
                   PHOBIUS SP: 0
                  PHOBIUS TMD: 0
            RefSeq annotation: hypothetical protein
     Name of enzyme/protein: Predicted DNA polymerase
       KEGG PATHWAY - level 1: Other function - Genetic Information
                               Processing
       KEGG PATHWAY - level 2: na
```

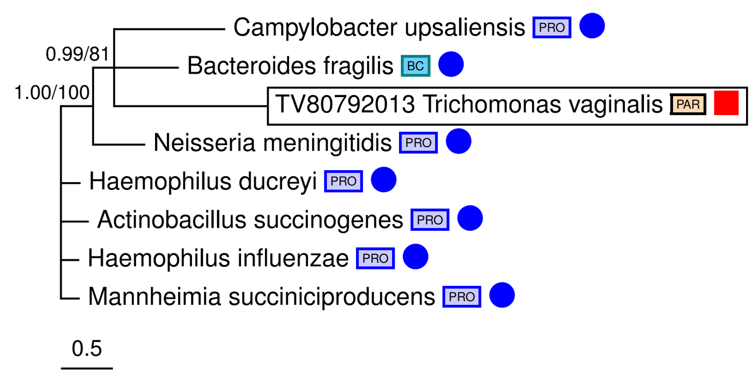

TN224

```
                Candy accession: TV82119086
               RefSeq accession: XP_001330618.1
              Uniprot accession: BELY_TRIVA
                       Comments: LGT - TV TWO NODES
               Species affected: TV
         Adjacent taxa in tree: Bacteroidetes/Chlorobi - Bacteroides
EC annotation - (Blast/Profile): EC:4.1.99.1
                     PHOBIUS SP: 0
                    PHOBIUS TMD: 0
              RefSeq annotation: beta-eliminating lyase
        Name of enzyme/protein: tryptophanase
        KEGG PATHWAY - level 1: Amino Acid Metabolism, Energy metabolism
        KEGG PATHWAY - level 2: Tryptophan metabolism, Nitrogen
                                 metabolism

                Candy accession: Q50Q06_ENTHI
               RefSeq accession: XP_649067.1
              Uniprot accession: C4M571_ENTHI
                       Comments: LGT - EH TWO NODES
               Species affected: EH
         Adjacent taxa in tree: Fusobacteria - Fusobacterium
EC annotation - (Blast/Profile): EC:4.1.99.1
                     PHOBIUS SP: 0
                    PHOBIUS TMD: 0
              RefSeq annotation: tryptophanase
        Name of enzyme/protein: tryptophanase
        KEGG PATHWAY - level 1: Amino Acid Metabolism, Energy metabolism
        KEGG PATHWAY - level 2: Tryptophan metabolism, Nitrogen
                                 metabolism
```

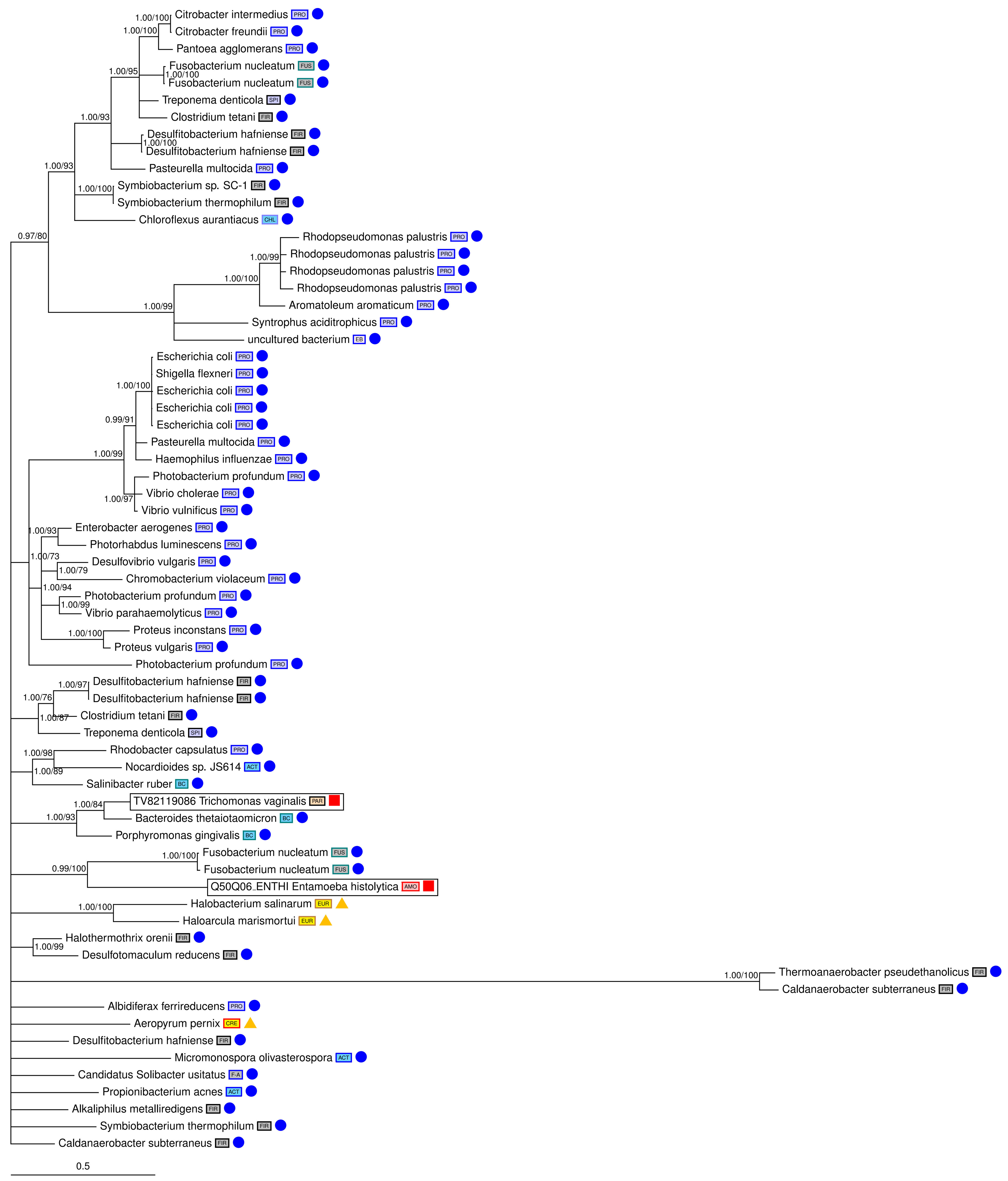

TN225

```
              Candy accession: TV82140099
             RefSeq accession: XP_001316257.1
            Uniprot accession: A2ETI8_TRIVA
                     Comments: LGT - TV TWO NODES - 3 DOMAIN ToL
             Species affected: TV
        Adjacent taxa in tree: Bacteroidetes/Chlorobi - Bacteroides
EC annotation - (Blast/Profile): EC:1.11.1.15
                   PHOBIUS SP: 0
                  PHOBIUS TMD: 0
            RefSeq annotation: thiol peroxidase
      Name of enzyme/protein: Thiol peroxidase
       KEGG PATHWAY - level 1: Metabolism of Other Amino Acids
       KEGG PATHWAY - level 2: Glutathione metabolism
```

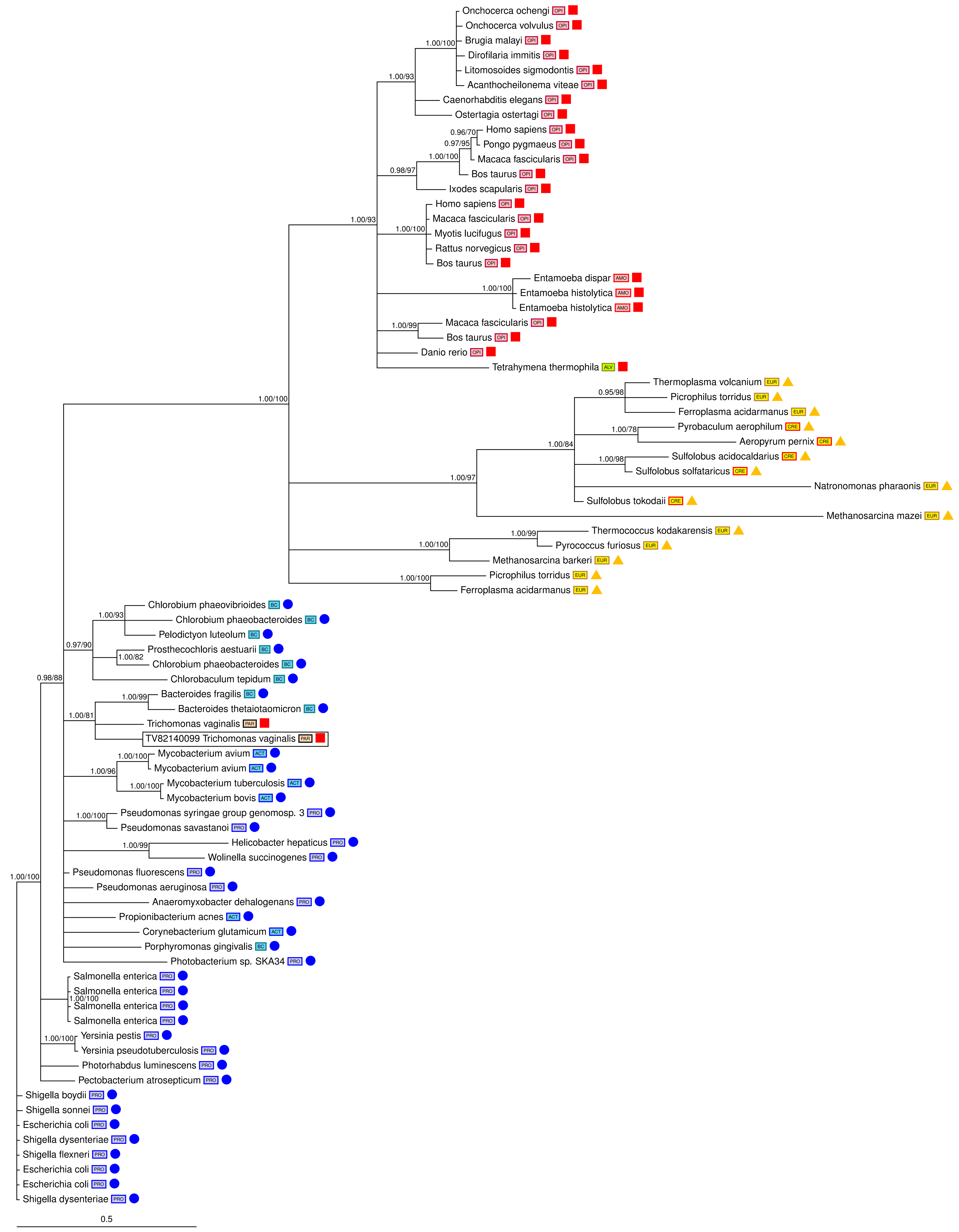

TN226

```
              Candy accession: TV82174116
              RefSeq accession: XP_001318849.1
            Uniprot accession: A2EL73_TRIVA
                    Comments: LGT - TV TWO NODES - DEAP LGT INTO
                              FUNGI?
            Species affected: TV
        Adjacent taxa in tree: Prokaryotes
EC annotation - (Blast/Profile): na
                  PHOBIUS SP: 0
                 PHOBIUS TMD: 12
            RefSeq annotation: Amino acid permease family protein
        Name of enzyme/protein: Amino acid permease family protein
        KEGG PATHWAY - level 1: Other function - Membrane transport
        KEGG PATHWAY - level 2: na
```

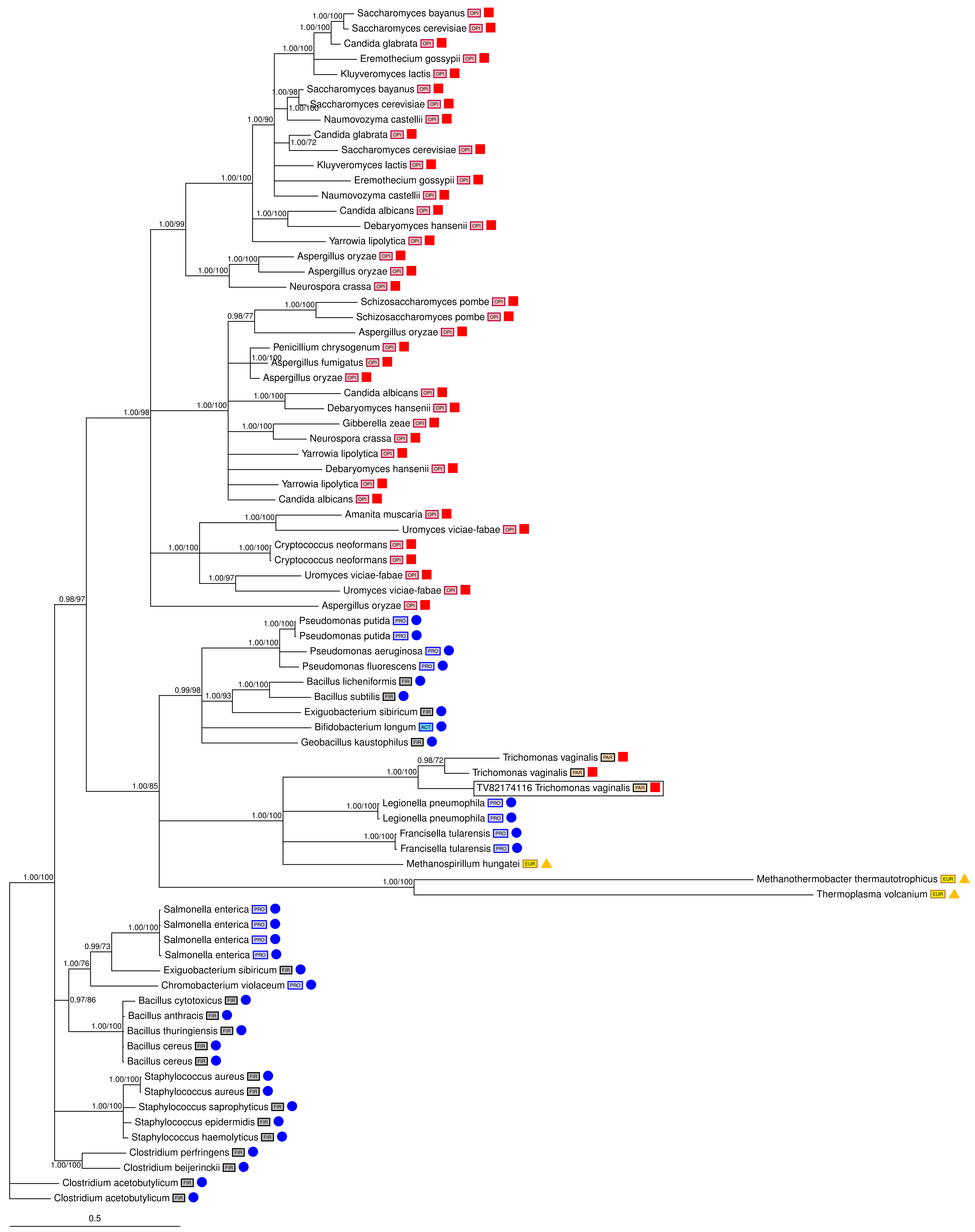

TN227

```
                Candy accession: TV82360235
               RefSeq accession: XP_001579672.1
              Uniprot accession: A2DLQ3_TRIVA
                       Comments: LGT - TV TWO NODES
               Species affected: TV
         Adjacent taxa in tree: Protobacteria
EC annotation - (Blast/Profile): EC:2.7.1.15
                     PHOBIUS SP: 0
                    PHOBIUS TMD: 0
              RefSeq annotation: kinase, pfkB family protein
       Name of enzyme/protein: ribokinase
        KEGG PATHWAY - level 1: Carbohydrate Metabolism
        KEGG PATHWAY - level 2: Pentose phosphate pathway
```

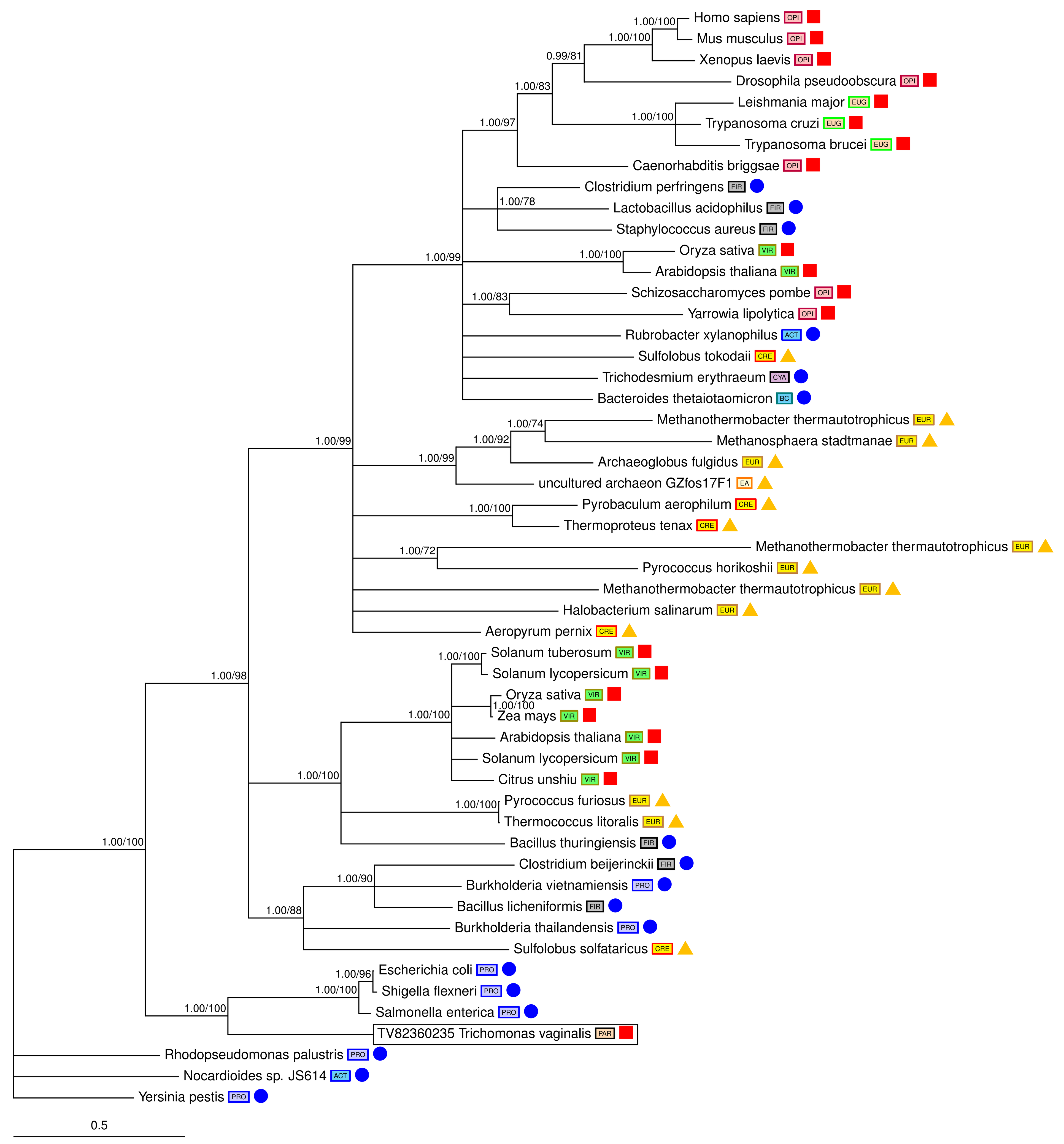

TN228

```
              Candy accession: TV90013073
             RefSeq accession: XP_001313313.1
            Uniprot accession: A2F3W8_TRIVA
                     Comments: LGT - TV TWO NODES + ONE FUNGI
             Species affected: TV
        Adjacent taxa in tree: Proteobacteria - Francisella
EC annotation - (Blast/Profile): na
                  PHOBIUS SP: 0
                 PHOBIUS TMD: 0
           RefSeq annotation: Zn-dependent hydrolase of beta-lactamase
     Name of enzyme/protein: Zn-dependent hydrolase of beta-lactamase
     KEGG PATHWAY - level 1: Other function
     KEGG PATHWAY - level 2: na
```

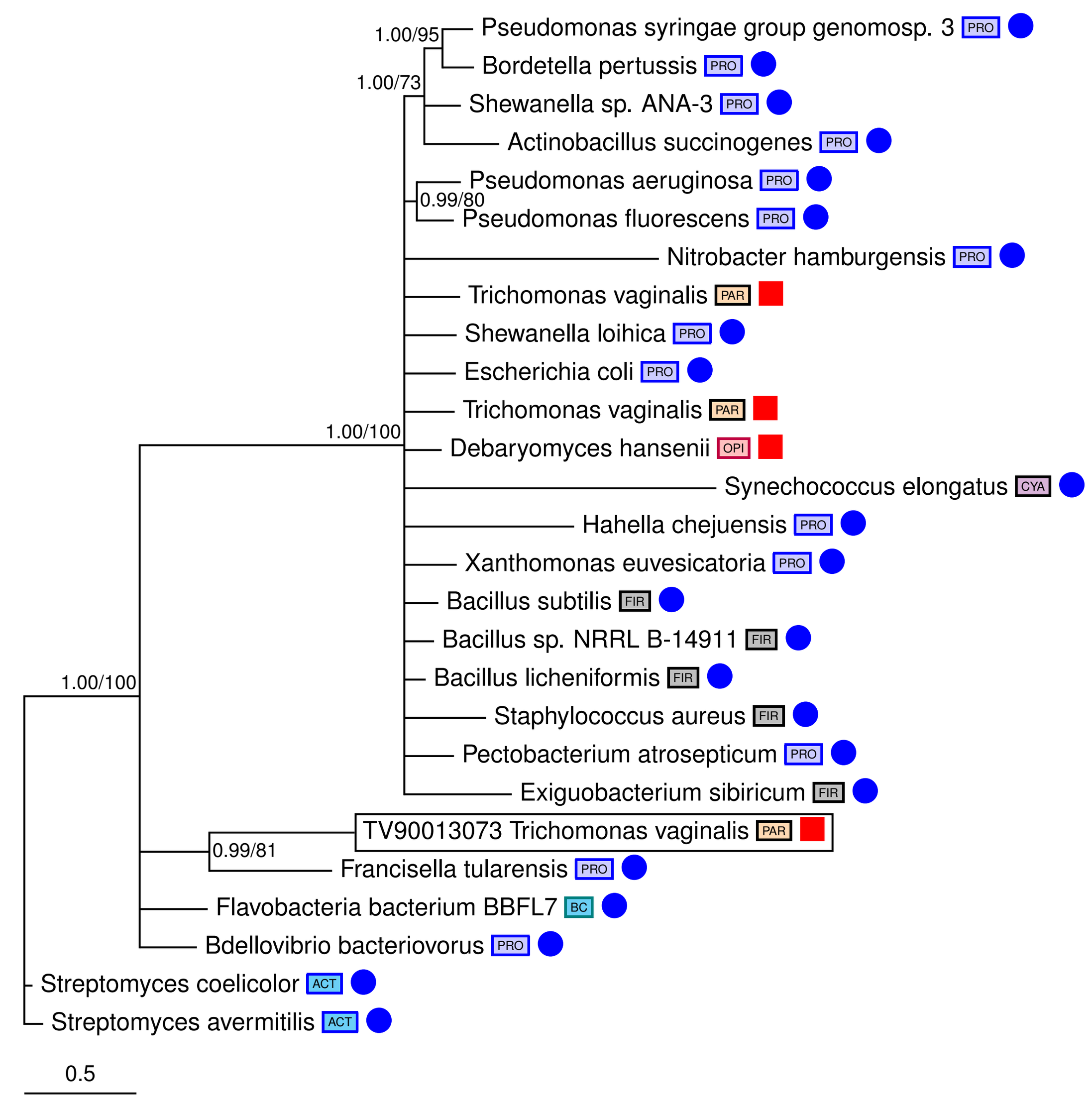

TN229

```
           Candy accession: TV94565011
          RefSeq accession: XP_001301668.1
         Uniprot accession: A2G280_TRIVA
                  Comments: LGT - TV  ONLY
          Species affected: TV
    Adjacent taxa in tree: Firmicutes
EC annotation - (Blast/Profile): na
               PHOBIUS SP: 0
              PHOBIUS TMD: 0
         RefSeq annotation: Integrase core domain containing protein
     Name of enzyme/protein: Putative transposase
    KEGG PATHWAY - level 1: Other function
    KEGG PATHWAY - level 2: na
```

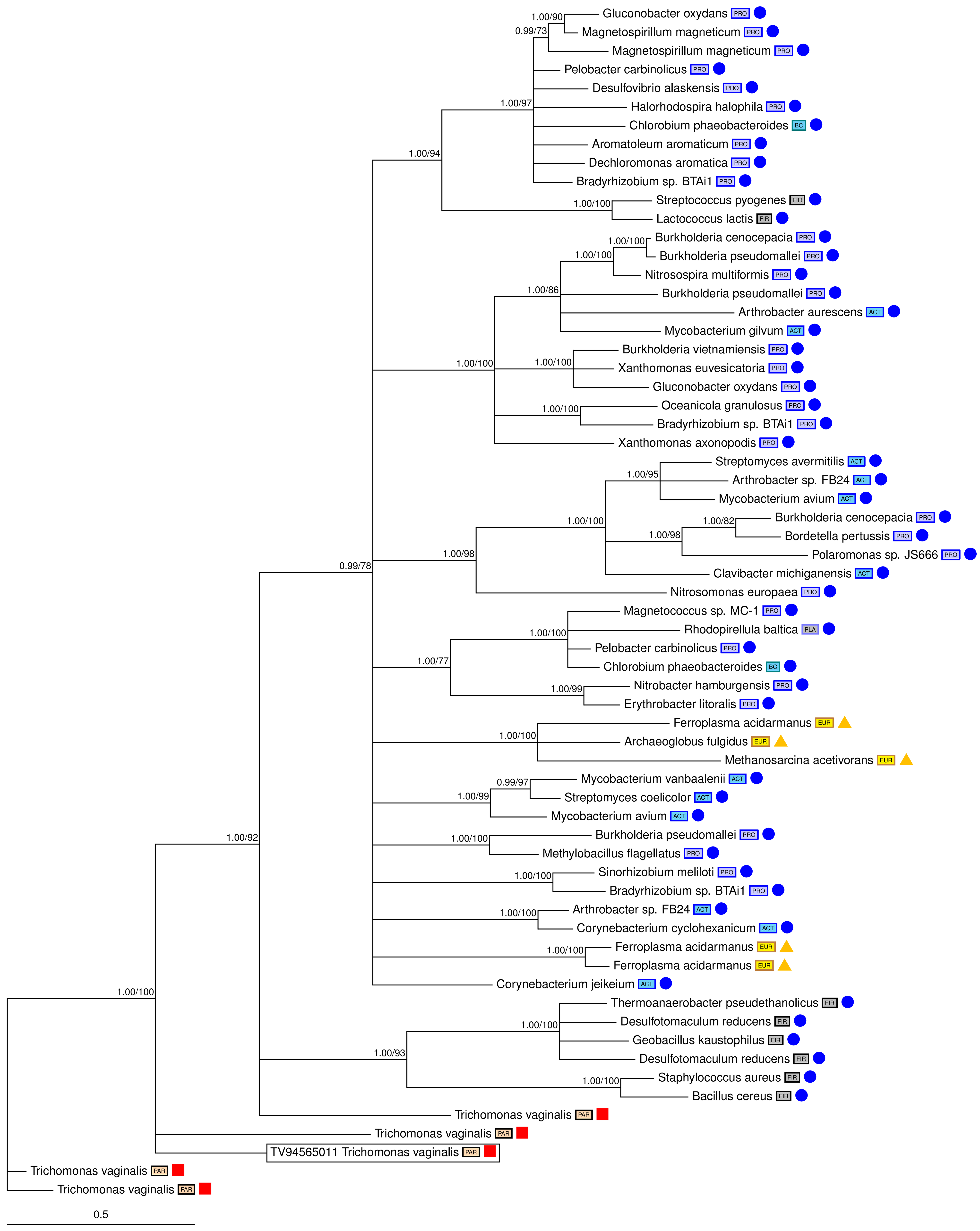

TN230

```
               Candy accession: TV85234074
              RefSeq accession: XP_001311801.1
             Uniprot accession: A2F8B1_TRIVA
                      Comments: LGT - TV ONLY
              Species affected: TV
        Adjacent taxa in tree: Spirochaetes - Treponema
EC annotation - (Blast/Profile): EC:2.7.8.20
                    PHOBIUS SP: 0
                   PHOBIUS TMD: 0
             RefSeq annotation: hypothetical protein
        Name of enzyme/protein: Phosphoglycerol transferase I
        KEGG PATHWAY - level 1: Lipid Metabolism
        KEGG PATHWAY - level 2: Glycerolipid metabolism
```

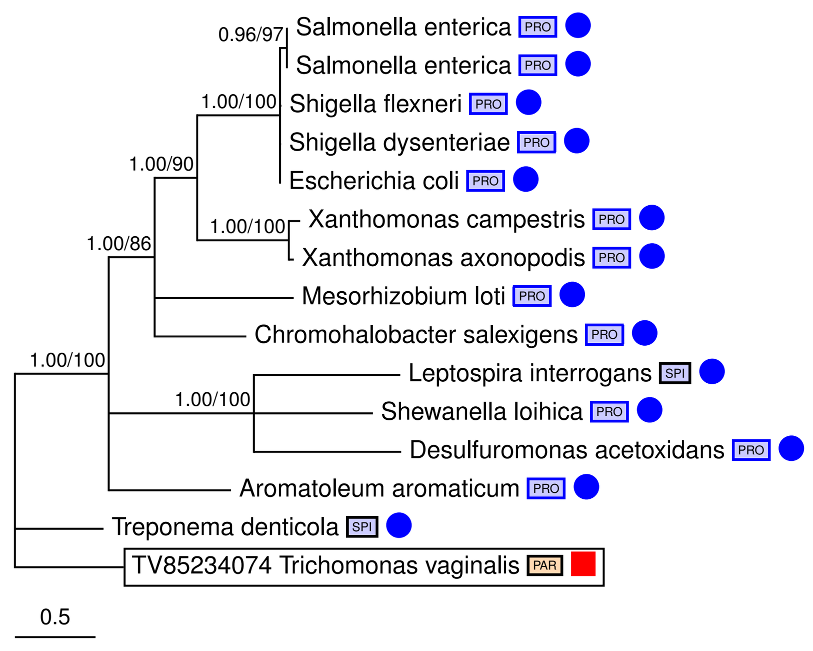

TN231

```
                Candy accession: TV87054088
               RefSeq accession: XP_001316278.1
              Uniprot accession: A2ETD5_TRIVA
                       Comments: LGT - TV TWO NODES
               Species affected: TV
          Adjacent taxa in tree: Proteobacteria - Pseudomonas
EC annotation - (Blast/Profile): EC:4.2.3.1
                     PHOBIUS SP: 0
                    PHOBIUS TMD: 0
              RefSeq annotation: threonine synthase family protein
         Name of enzyme/protein: Threonine synthase
         KEGG PATHWAY - level 1: Amino Acid Metabolism, Metabolism of
                                 Cofactors and Vitamins
         KEGG PATHWAY - level 2: Glycine, serine and threonine
                                 metabolism, Vitamin B6 metabolism
```

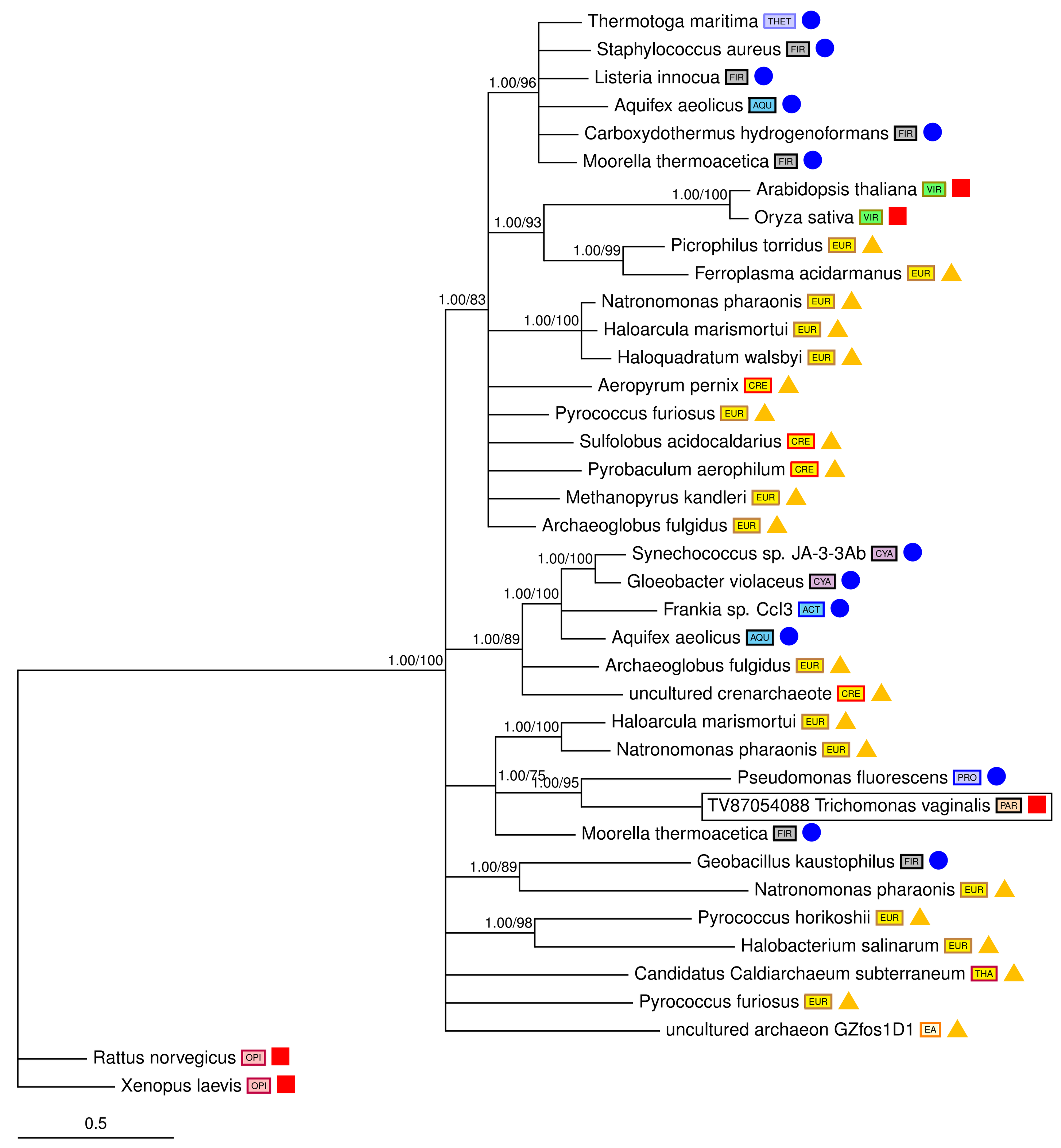

TN232

Candy accession: TV87349170
RefSeq accession: XP_001318930.1
Uniprot accession: A2EKZ9_TRIVA
Comments: LGT - TV TWO NODES
Species affected: TV
Adjacent taxa in tree: Bacteria
EC annotation - (Blast/Profile): EC:3.8.1.-
PHOBIUS SP: 0
PHOBIUS TMD: 0
RefSeq annotation: Amidohydrolase family protein
Name of enzyme/protein: Hydrolases (Putative chlorohydrolase/aminohydrolase)
KEGG PATHWAY - level 1: Reaction
KEGG PATHWAY - level 2: Reaction

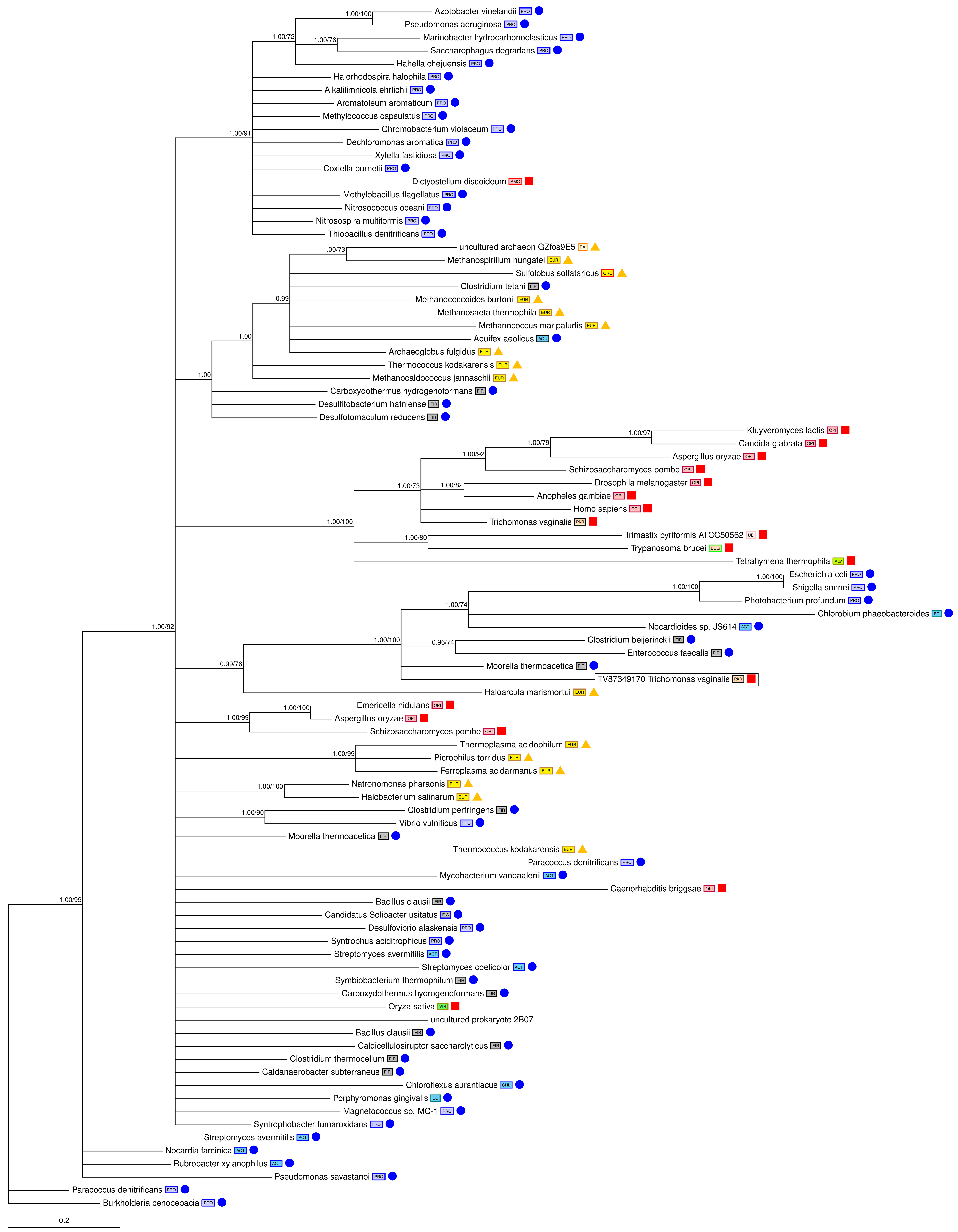

TN233

```
         Candy accession: TV87998038
        RefSeq accession: XP_001302090.1
       Uniprot accession: A2G102_TRIVA
                Comments: LGT - TV TWO NODES
        Species affected: TV
   Adjacent taxa in tree: Prokaryotes
EC annotation - (Blast/Profile): na
             PHOBIUS SP: 0
            PHOBIUS TMD: 11
       RefSeq annotation: major facilitator superfamily
                          transporter
  Name of enzyme/protein: major facilitator superfamily
                          transporter
   KEGG PATHWAY - level 1: Other function - Membrane transport
   KEGG PATHWAY - level 2: na
```

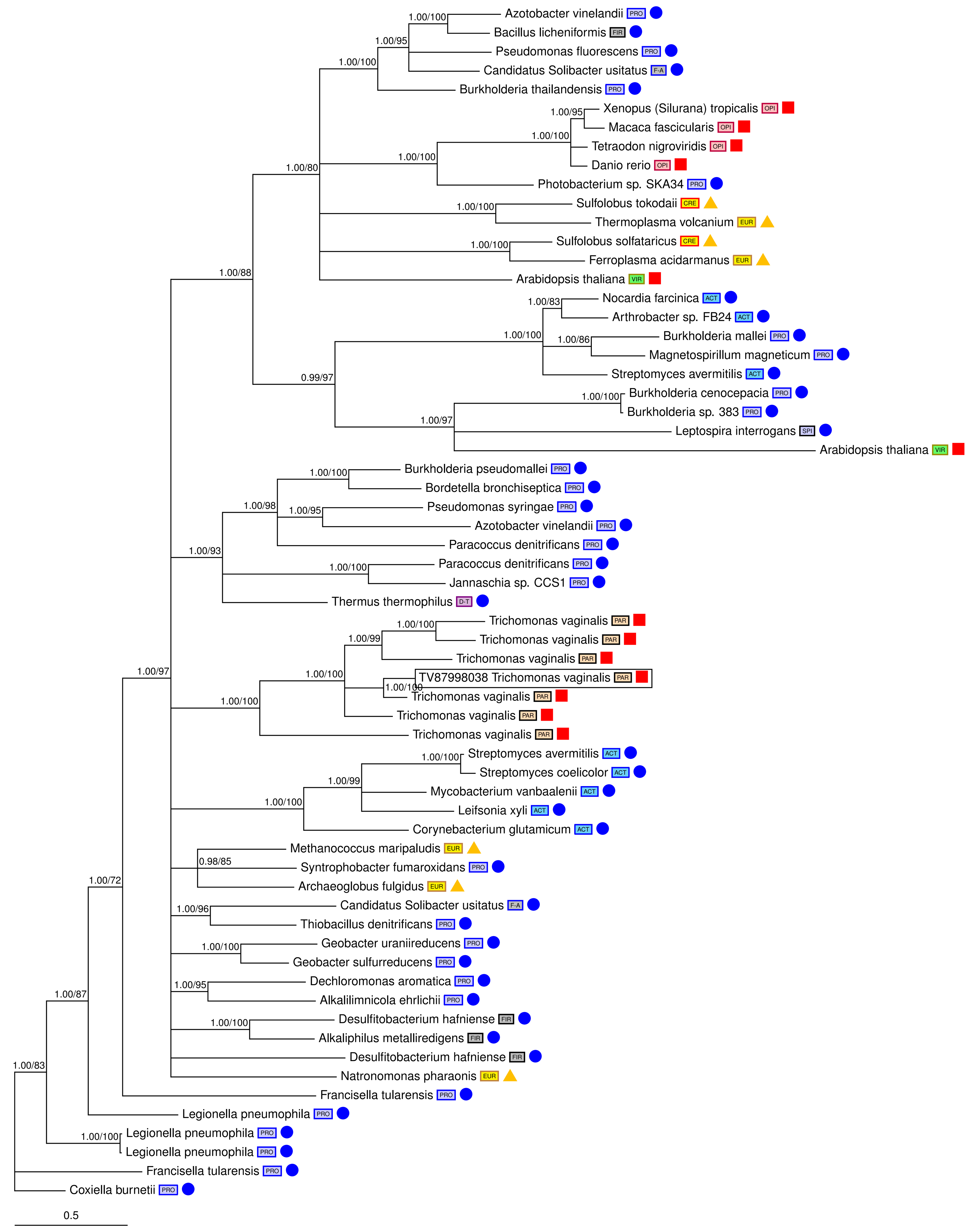

TN234

```
              Candy accession: TV88428065
             RefSeq accession: XP_001307943.1
            Uniprot accession: A2FJ99_TRIVA
                     Comments: LGT - TV TWO NODES
             Species affected: TV, FUNGI
        Adjacent taxa in tree: Bacteria
EC annotation - (Blast/Profile): EC:3.5.1.41
                  PHOBIUS SP: 0
                 PHOBIUS TMD: 1
            RefSeq annotation: Polysaccharide deacetylase family
                               protein
      Name of enzyme/protein: Chitin deacetylase
      KEGG PATHWAY - level 1: Carbohydrate Metabolism
      KEGG PATHWAY - level 2: Amino sugar and nucleotide sugar
                               metabolism
```

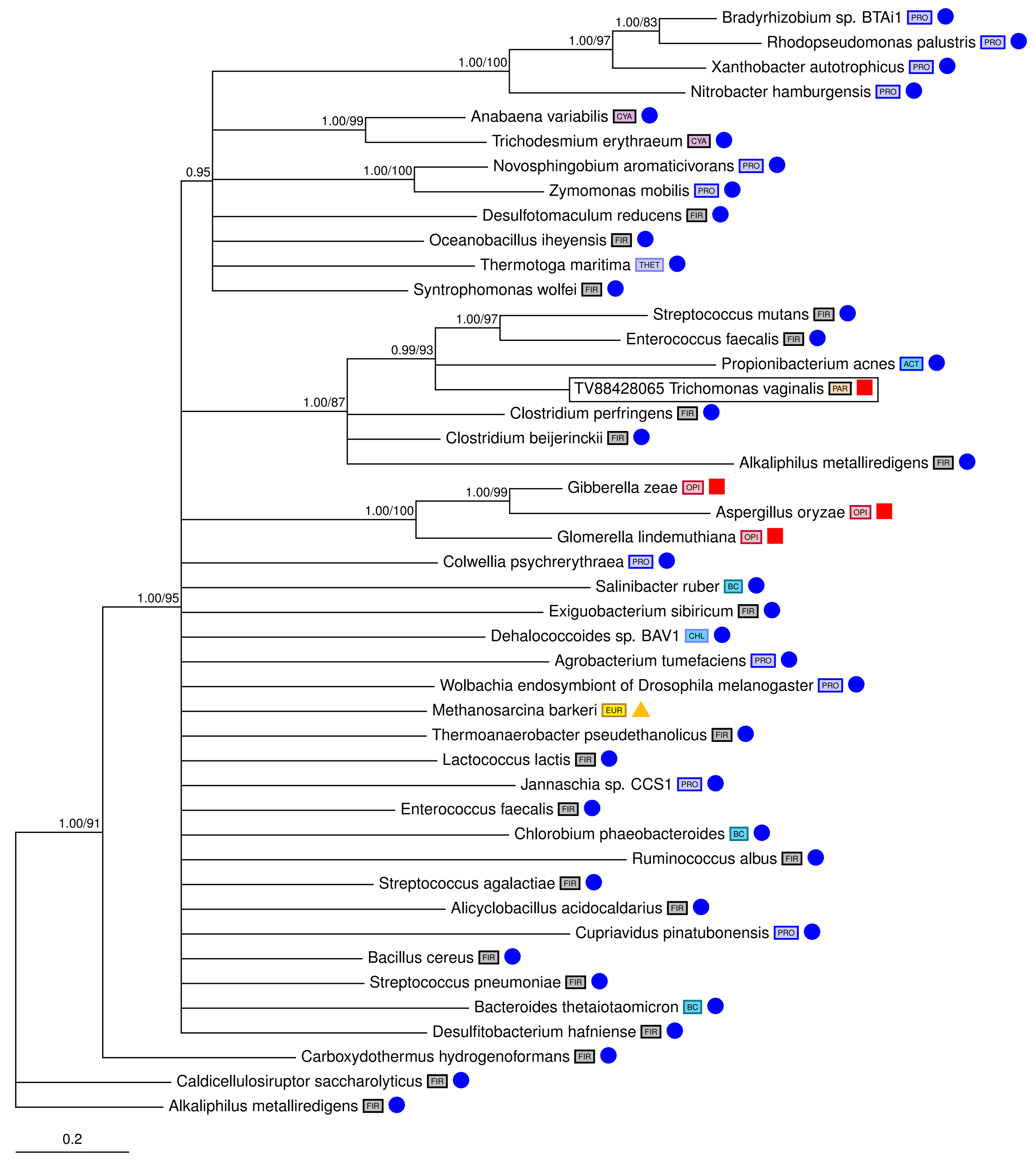

TN235

```
             Candy accession: Q511D6_ENTHI
            RefSeq accession: XP_652839.1
           Uniprot accession: Q869B1_ENTHI
                    Comments: LGT - EH TWO NODES
            Species affected: EH
       Adjacent taxa in tree: Bacteria
EC annotation - (Blast/Profile): EC:1.12.7.2
                  PHOBIUS SP: 0
                 PHOBIUS TMD: 0
           RefSeq annotation: Fe-hydrogenase
    Name of enzyme/protein: ferredoxin hydrogenase
    KEGG PATHWAY - level 1: Energy Metabolism
    KEGG PATHWAY - level 2: Methane metabolism
```

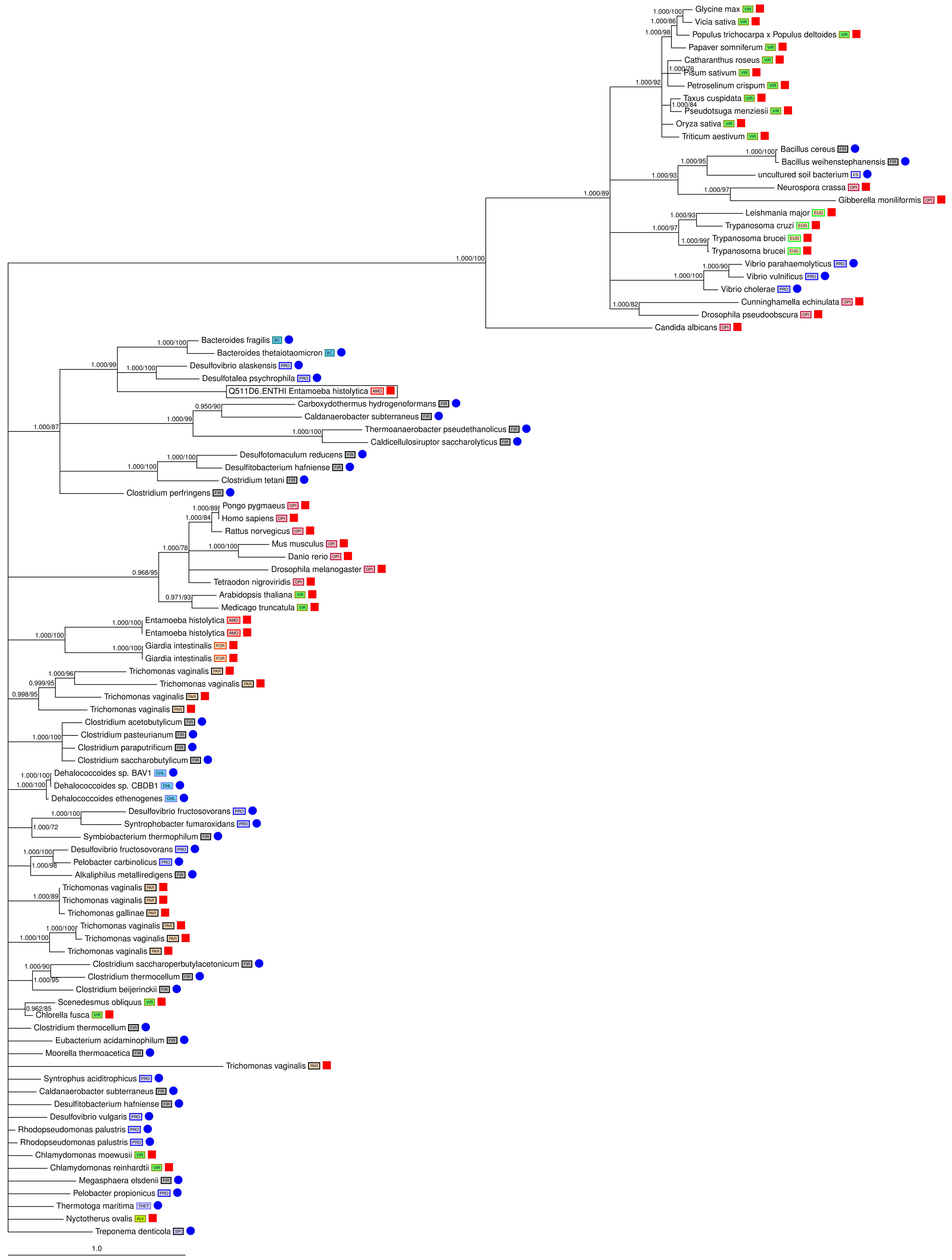

TN236

```
             Candy accession: Q54L36_DICDI
            RefSeq accession: XP_637467.1
           Uniprot accession: Q54L36_DICDI
                    Comments: LGT - DD TWO NODES
            Species affected: DD
       Adjacent taxa in tree: Firmicutes - Staphylococcus
EC annotation - (Blast/Profile): EC:3.1.1.3
                  PHOBIUS SP: 0
                 PHOBIUS TMD: 0
           RefSeq annotation: esterase/lipase/thioesterase domain-
                              containing protein
       Name of enzyme/protein: triacylglycerol lipase
       KEGG PATHWAY - level 1: Lipid Metabolism
       KEGG PATHWAY - level 2: Glycerolipid metabolism
```

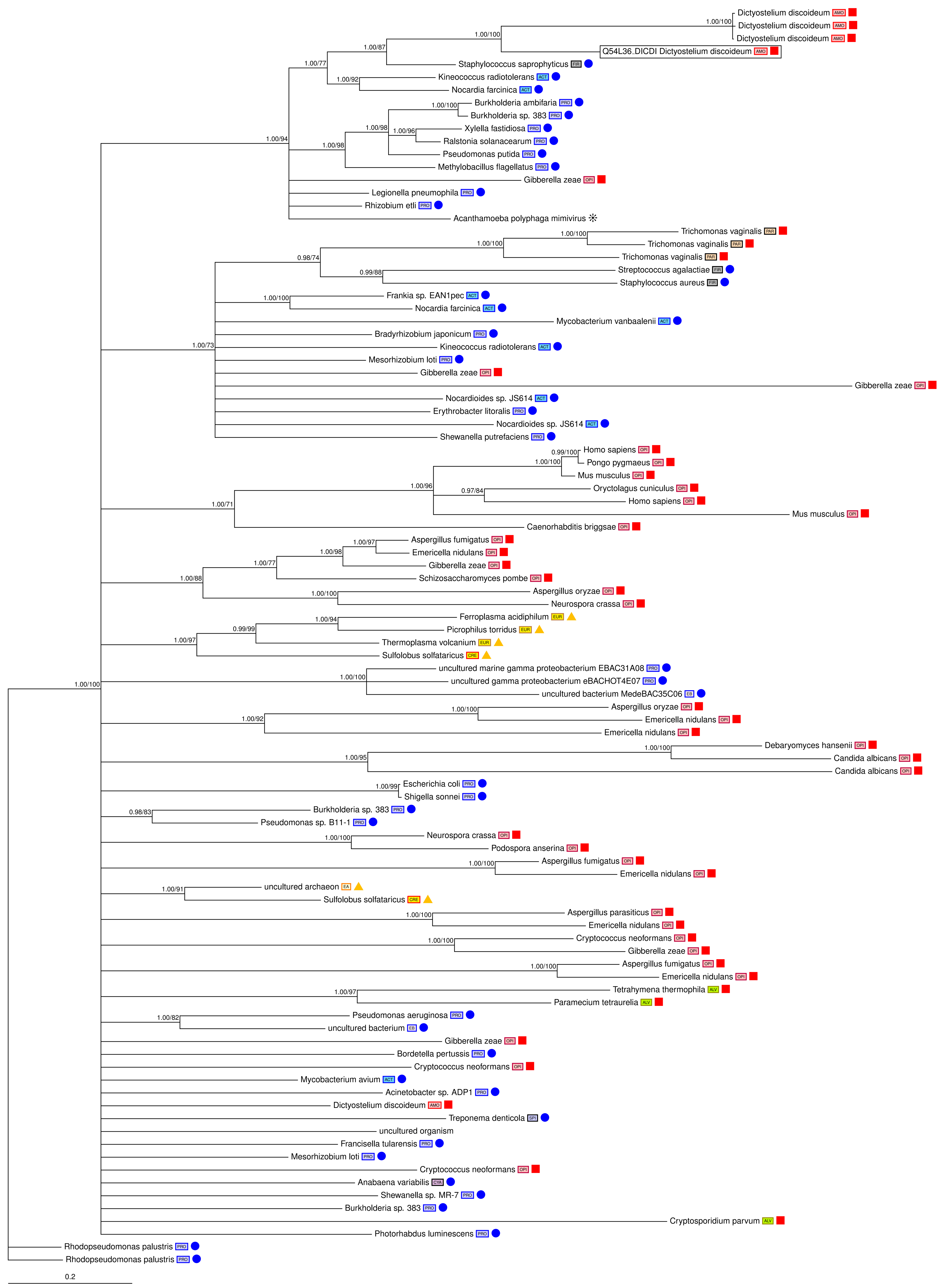

TN237

```
                Candy accession: TV92683026
               RefSeq accession: XP_001302496.1
              Uniprot accession: A2FZU4_TRIVA
                       Comments: LGT - TV TWO NODES
               Species affected: TV
           Adjacent taxa in tree: Prokaryotes
EC annotation - (Blast/Profile): na
                     PHOBIUS SP: 0
                    PHOBIUS TMD: 0
              RefSeq annotation: nitroimidazole resistance protein
          Name of enzyme/protein: Predicted flavin-nucleotide-binding
                                 protein/5-nitroimidazole antibiotic
                                 resistance protein
         KEGG PATHWAY - level 1: Other function
         KEGG PATHWAY - level 2: na

                Candy accession: Q516J9_ENTHI
               RefSeq accession: XP_654156.2
              Uniprot accession: C4LU35_ENTHI
                       Comments: LGT - EH TWO NODES
               Species affected: EH
           Adjacent taxa in tree: Bacteria
EC annotation - (Blast/Profile): na
                     PHOBIUS SP: 0
                    PHOBIUS TMD: 0
              RefSeq annotation: 5-nitroimidazole antibiotic resistance
                                 protein
          Name of enzyme/protein: Predicted flavin-nucleotide-binding
                                 protein/5-nitroimidazole antibiotic
                                 resistance protein
         KEGG PATHWAY - level 1: Other function
         KEGG PATHWAY - level 2: na
```

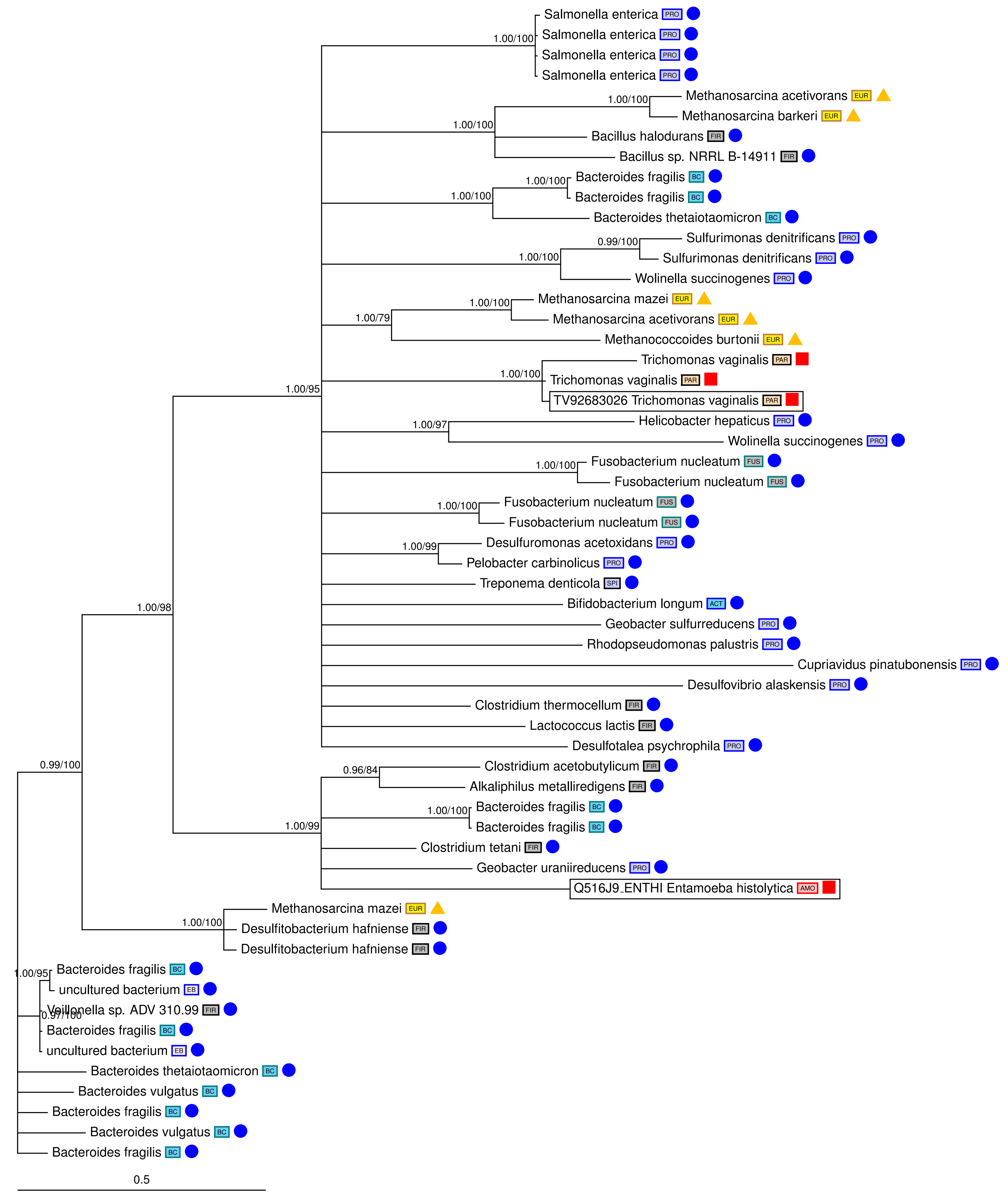

TN238

```
          Candy accession: TV94275097
         RefSeq accession: XP_001313279.1
        Uniprot accession: A2F3Z6_TRIVA
                 Comments: LGT - TV TWO NODES + LGT INTO FUNGI
         Species affected: TV,FUNGI
   Adjacent taxa in tree: Bacteroidetes/Chlorobi - Bacteroides
EC annotation - (Blast/Profile): EC:5.1.3.3
              PHOBIUS SP: 0
             PHOBIUS TMD: 0
       RefSeq annotation: Aldose 1-epimerase family protein
 Name of enzyme/protein: Aldose 1-epimerase
   KEGG PATHWAY - level 1: Carbohydrate Metabolism
   KEGG PATHWAY - level 2: Glycolysis / Gluconeogenesis
```

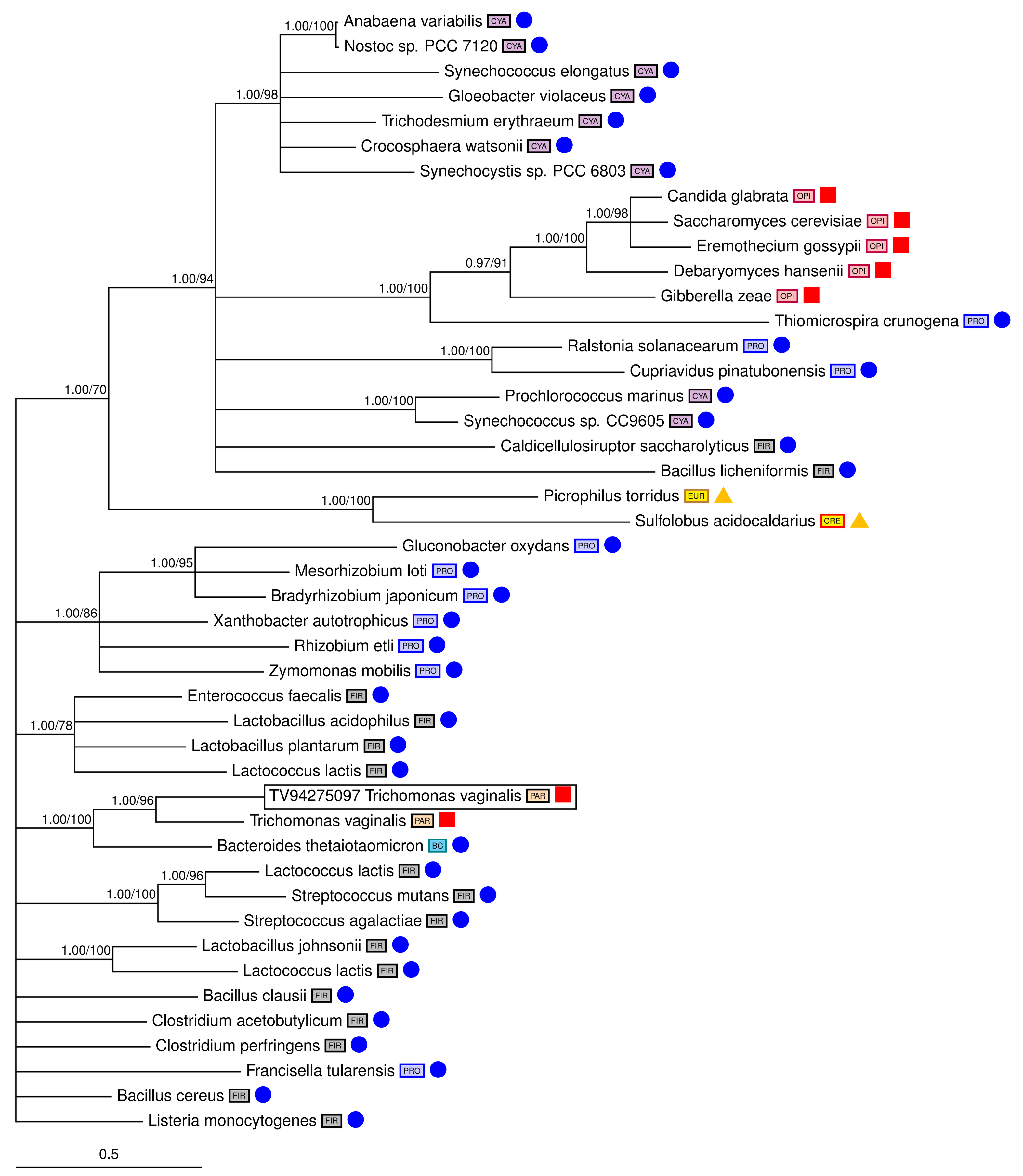

TN239

Candy accession:	TV95580062
RefSeq accession:	XP_001305004.1
Uniprot accession:	A2FSP3_TRIVA
Comments:	LGT - TV ONLY
Species affected:	TV
Adjacent taxa in tree:	Bacteria
EC annotation - (Blast/Profile):	EC:5.1.3.2
PHOBIUS SP:	0
PHOBIUS TMD:	1
RefSeq annotation:	hypothetical protein
Name of enzyme/protein:	UDP-glucose 4-epimerase
KEGG PATHWAY - level 1:	Carbohydrate Metabolism
KEGG PATHWAY - level 2:	Galactose metabolism, Amino sugar and nucleotide sugar metabolism

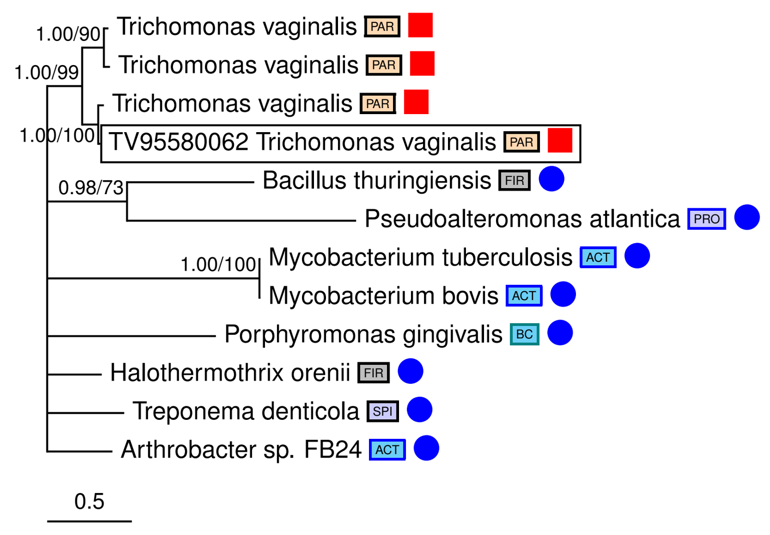

TN240

```
                 Candy accession: TV97007140
                RefSeq accession: XP_001322711.1
               Uniprot accession: A2EA42_TRIVA
                        Comments: LGT - TV ONLY
                Species affected: TV
          Adjacent taxa in tree: Bacteria
EC annotation - (Blast/Profile): na
                      PHOBIUS SP: 0
                     PHOBIUS TMD: 10
               RefSeq annotation: YeeE/YedE family protein
         Name of enzyme/protein: YeeE/YedE family protein
         KEGG PATHWAY - level 1: Other function - Membrane transport
         KEGG PATHWAY - level 2: na
```

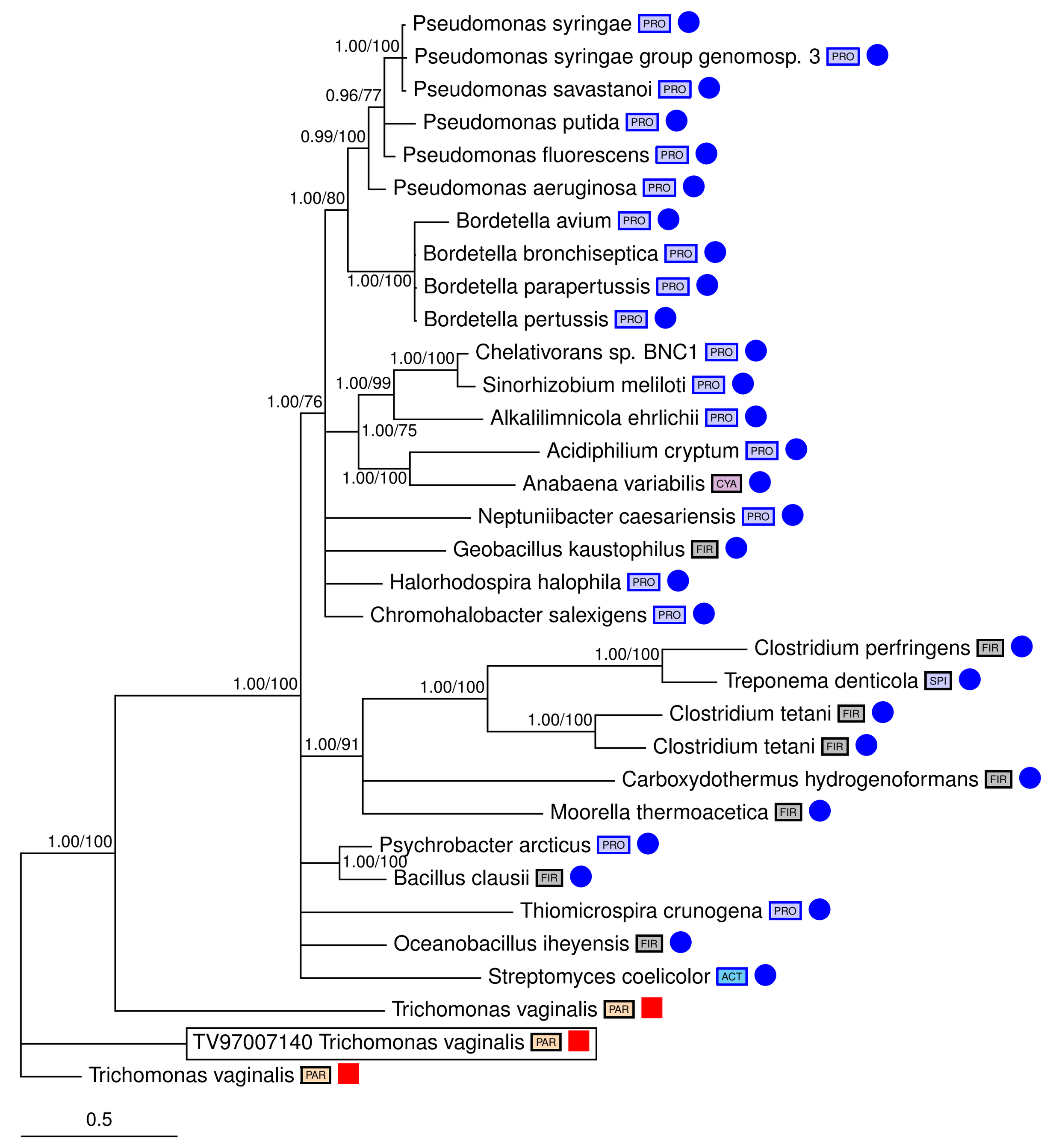

TN241

```
Candy accession: TV112459012
RefSeq accession: XP_001298741.1
Uniprot accession: A2GAK7_TRIVA
Comments: LGT - TV TWO NODES
Species affected: TV
Adjacent taxa in tree: Bacteria
EC annotation - (Blast/Profile): EC:2.1.3.9
PHOBIUS SP: 0
PHOBIUS TMD: 0
RefSeq annotation: hypothetical protein
Name of enzyme/protein: N-acetylornithine carbamoyltransferase
KEGG PATHWAY - level 1: Amino Acid Metabolism
KEGG PATHWAY - level 2: Arginine and proline metabolism
```

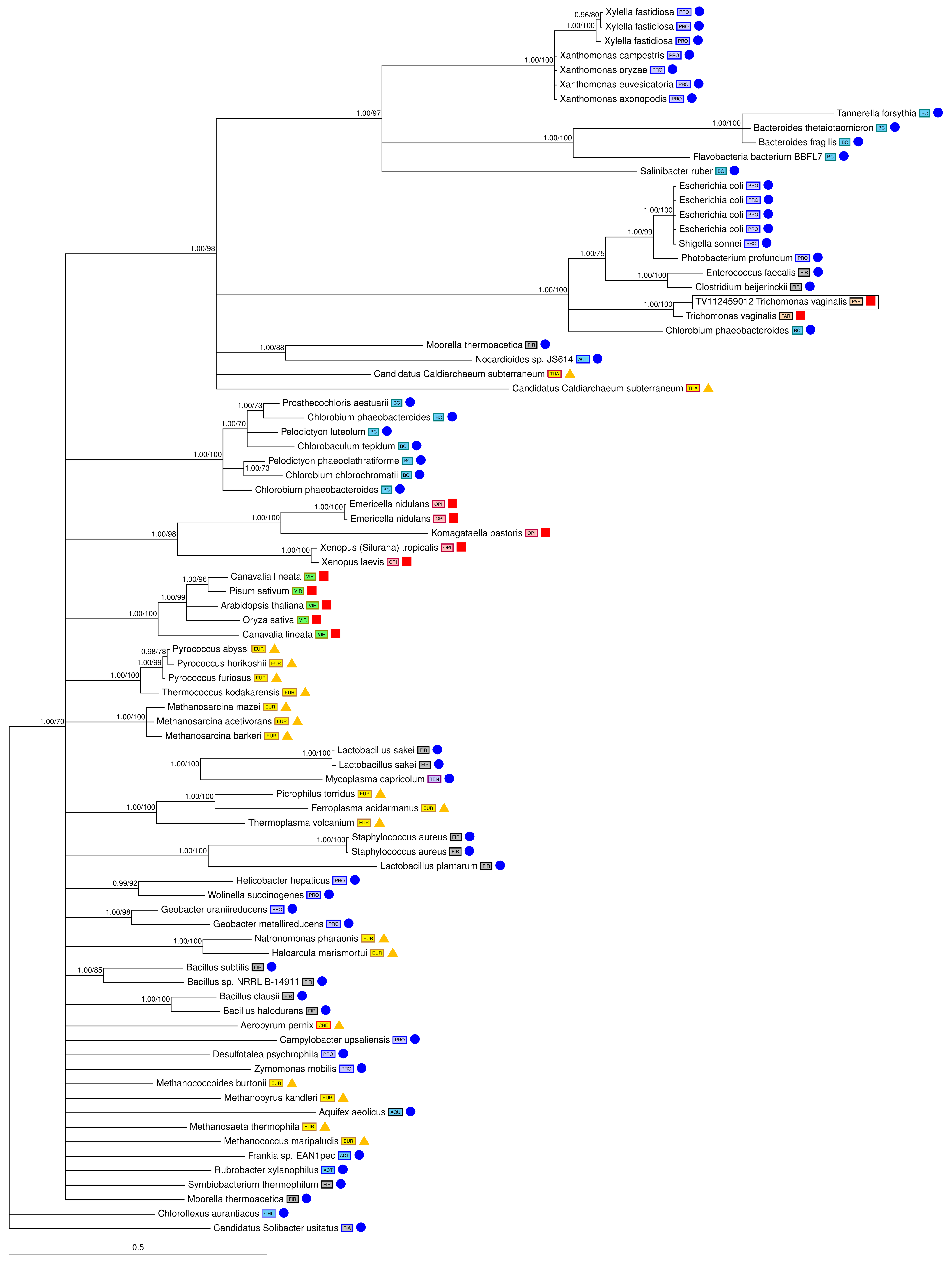

TN242

Candy accession:	Q5CPU3_CRYPV
RefSeq accession:	XP_625421.1
Uniprot accession:	Q5CPU3_CRYPV
Comments:	LGT - DEEP LGT INTO APICOMPLEXA
Species affected:	CP,TA,PF,PC,PV
Adjacent taxa in tree:	Bacteria
EC annotation - (Blast/Profile):	EC:2.3.1.88
PHOBIUS SP:	0
PHOBIUS TMD:	0
RefSeq annotation:	possible ribosomal-protein-alanine acetyltransferase
Name of enzyme/protein:	Predicted acetyltransferase
KEGG PATHWAY - level 1:	Reaction
KEGG PATHWAY - level 2:	Reaction

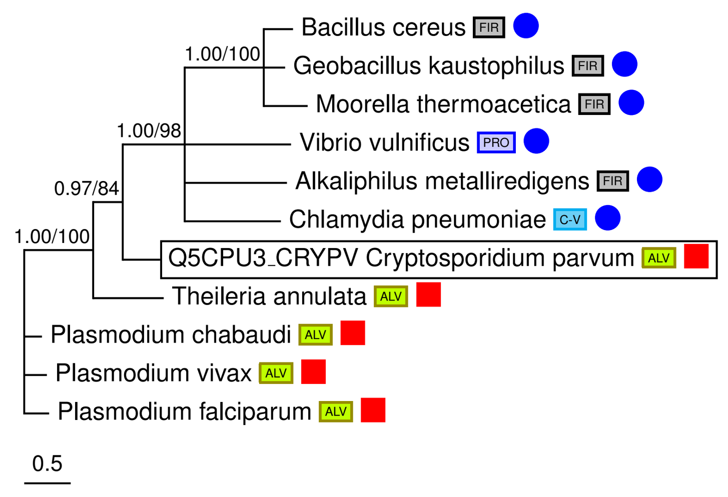

TN243

Candy accession:	EAL44226
RefSeq accession:	XP_649612.1
Uniprot accession:	C4M2R8_ENTHI
Comments:	LGT - EH ONLY + MIMIVIRUS + PHAGES
Species affected:	EH
Adjacent taxa in tree:	Prokaryotes
EC annotation - (Blast/Profile):	na
PHOBIUS SP:	0
PHOBIUS TMD:	0
RefSeq annotation:	ser/thr protein phosphatase family protein
Name of enzyme/protein:	Protein containing metallophosphatase domain
KEGG PATHWAY - level 1:	Other function
KEGG PATHWAY - level 2:	na

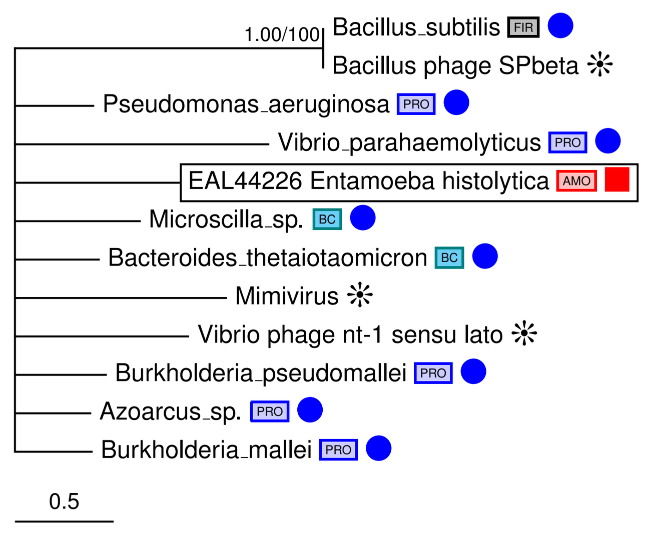

TN244

```
              Candy accession: EAL47525
             RefSeq accession: XP_652912.1
            Uniprot accession: C4M4C4_ENTHI
                     Comments: LGT - EH ONLY
             Species affected: EH
       Adjacent taxa in tree: Prokaryotes
EC annotation - (Blast/Profile): na
                   PHOBIUS SP: Y
                  PHOBIUS TMD: 0
            RefSeq annotation: competence protein ComEC
     Name of enzyme/protein: Predicted lactamase B superafamily
        KEGG PATHWAY - level 1: Other function
        KEGG PATHWAY - level 2: na
```

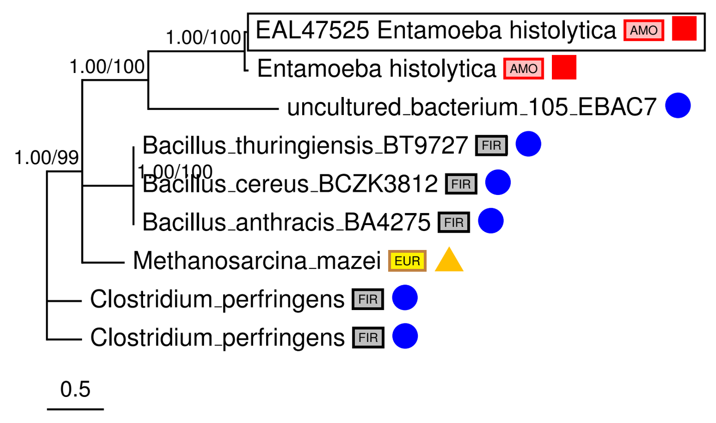

TN245

```
                Candy accession: LM0065
               RefSeq accession: XP_001687110.1
              Uniprot accession: Q4Q0N8_LEIMA
                       Comments: LGT - KINETOPLASTIDS ONLY - WITH DEAP
                                 DUPLICATION
               Species affected: LM,TB,TC
          Adjacent taxa in tree: Bacteria
EC annotation - (Blast/Profile): EC:3.2.1.28
                     PHOBIUS SP: 0
                    PHOBIUS TMD: 0
              RefSeq annotation: glycosyl hydrolase
         Name of enzyme/protein: alpha,alpha-trehalase
        KEGG PATHWAY - level 1: Carbohydrate Metabolism
        KEGG PATHWAY - level 2: Starch and sucrose metabolism
```

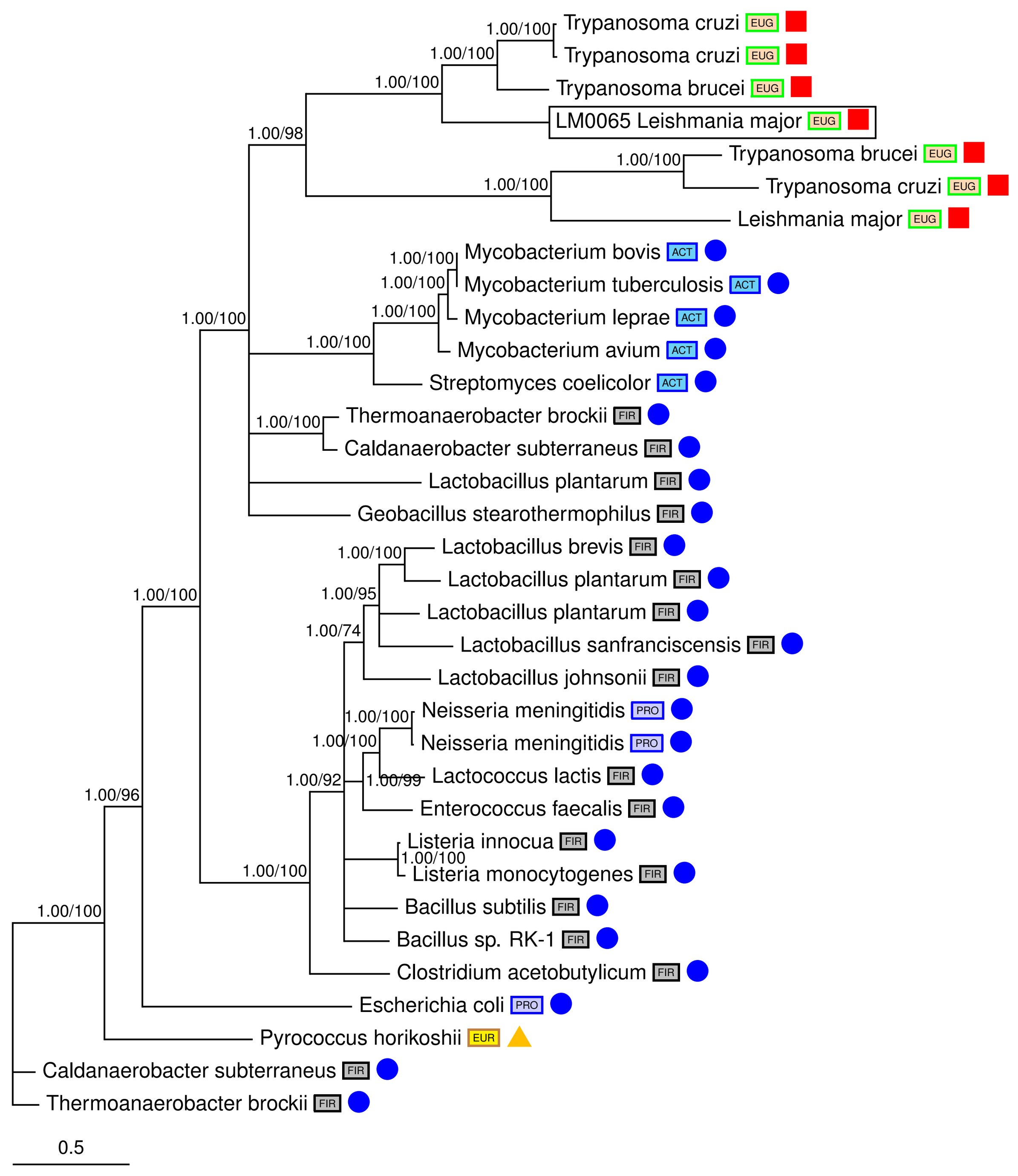

TN246

```
              Candy accession: TV143798005
             RefSeq accession: XP_001294330.1
            Uniprot accession: A2GN67_TRIVA
                     Comments: LGT - TV ONLY
             Species affected: TV
         Adjacent taxa in tree: Bacteria
EC annotation - (Blast/Profile): EC:2.4.1.58
                   PHOBIUS SP: 0
                  PHOBIUS TMD: 0
            RefSeq annotation: glycosyl transferase
    Name of enzyme/protein: lipopolysaccharide glucosyltransferase I
    KEGG PATHWAY - level 1: Glycan Biosynthesis and Metabolism
    KEGG PATHWAY - level 2: Lipopolysaccharide biosynthesis
```

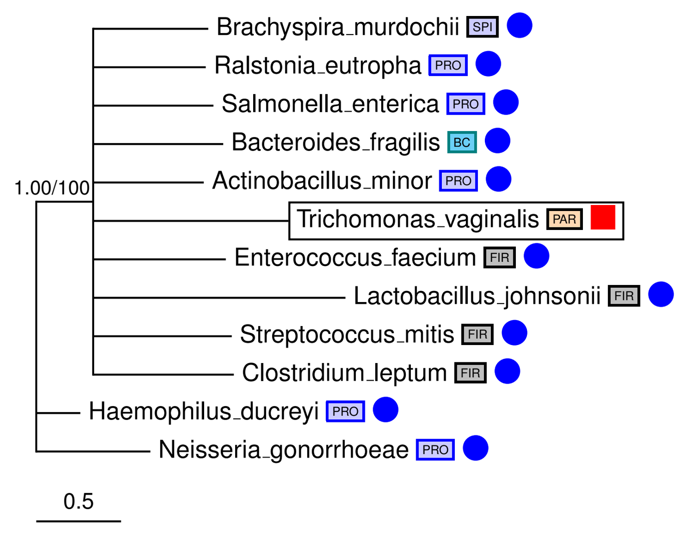

TN247

```
                Candy accession: TV84270673
               RefSeq accession: XP_001276902.1
              Uniprot accession: A2D7D7_TRIVA
                       Comments: LGT - TV ONLY
               Species affected: TV
          Adjacent taxa in tree: Bacteria
EC annotation - (Blast/Profile): EC:3.4.-.-
                     PHOBIUS SP: 0
                    PHOBIUS TMD: 0
              RefSeq annotation: Clan CA, family C40, NlpC/P60
                                 superfamily cysteine peptidase
         Name of enzyme/protein: Peptidase family C40
         KEGG PATHWAY - level 1: Reaction
         KEGG PATHWAY - level 2: Reaction
```

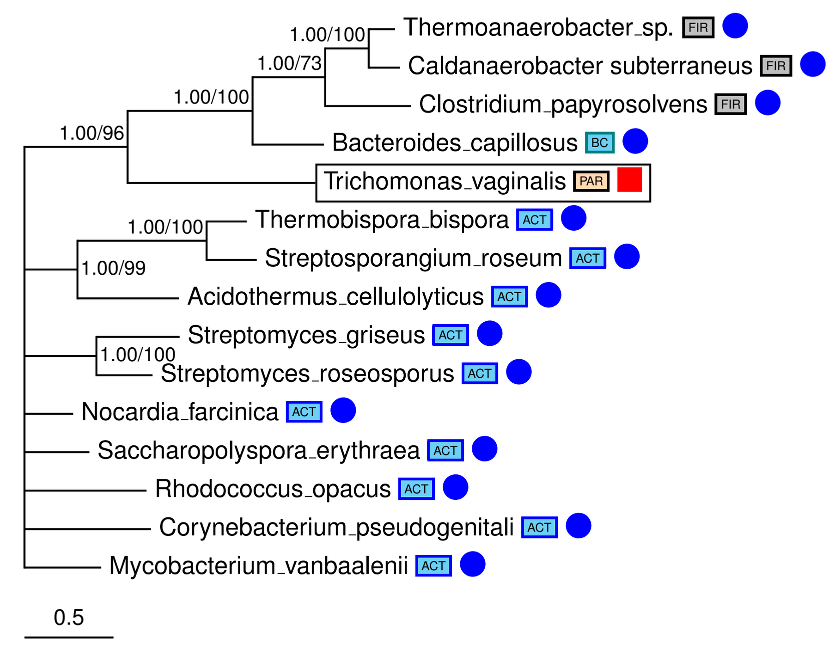

TN248

```
                 Candy accession: TV90380186
                RefSeq accession: XP_001324349.1
               Uniprot accession: A2E5G5_TRIVA
                        Comments: LGT - TV ONLY
                Species affected: TV
            Adjacent taxa in tree: Bacteria
EC annotation - (Blast/Profile): EC:6.1.1.15
                     PHOBIUS SP: 0
                    PHOBIUS TMD: 0
               RefSeq annotation: YbaK / prolyl-tRNA synthetases
                                  associated domain containing protein
          Name of enzyme/protein:  prolyl-tRNA synthetase  (fragment)
         KEGG PATHWAY - level 1: Translation - Genetic Information
                                  Processing
         KEGG PATHWAY - level 2: Aminoacyl-tRNA biosynthesis
```

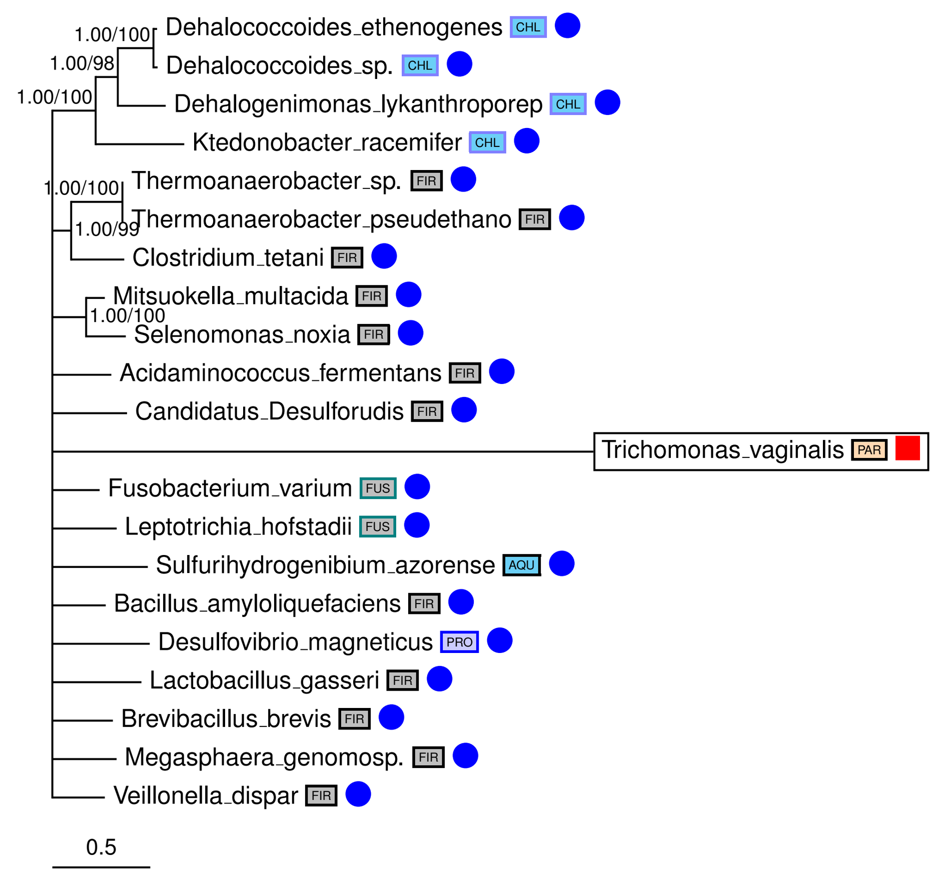

TN249

```
                Candy accession: Q4QDN1_LEIMA
               RefSeq accession: XP_001682567.1
              Uniprot accession: Q4QDN1_LEIMA
                       Comments: LGT - KINETOPLASTIDS TWO NODES
               Species affected: LM,LD,LI,LC,LT,CF
           Adjacent taxa in tree: Bacteria
EC annotation - (Blast/Profile): EC:3.2.2.1, EC:3.2.2.3
                     PHOBIUS SP: 0
                    PHOBIUS TMD: 0
              RefSeq annotation: nonspecific nucleoside hydrolase
         Name of enzyme/protein: purine nucleosidase, uridine
                                 nucleosidase
         KEGG PATHWAY - level 1: Nucleotide Metabolism, Metabolism of
                                 Cofactors and Vitamins
         KEGG PATHWAY - level 2: Purine metabolism, Nicotinate and
                                 nicotinamide metabolism
```

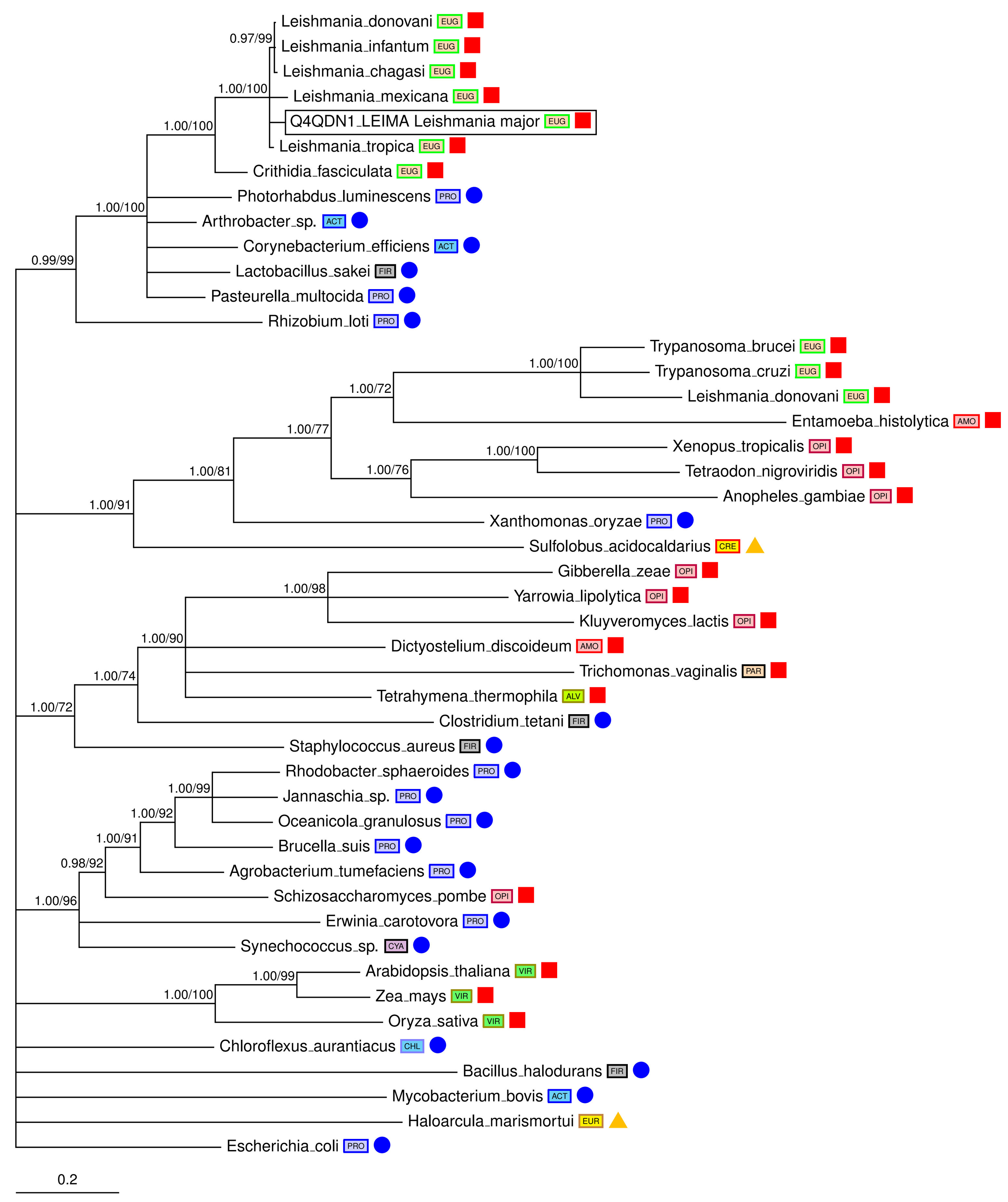

TN250

```
              Candy accession: EAL49869
             RefSeq accession: XP_655257.1
            Uniprot accession: Q769I7_ENTHI
                     Comments: LGT - EH TWO NODES
             Species affected: EH
       Adjacent taxa in tree: Proteobacteria
EC annotation - (Blast/Profile): EC:2.8.1.7
                   PHOBIUS SP: 0
                  PHOBIUS TMD: 0
            RefSeq annotation: cysteine desulfurase
     Name of enzyme/protein: Cysteine desulfurase
     KEGG PATHWAY - level 1: Metabolism of Cofactors and Vitamins
     KEGG PATHWAY - level 2: Thiamine metabolism
```

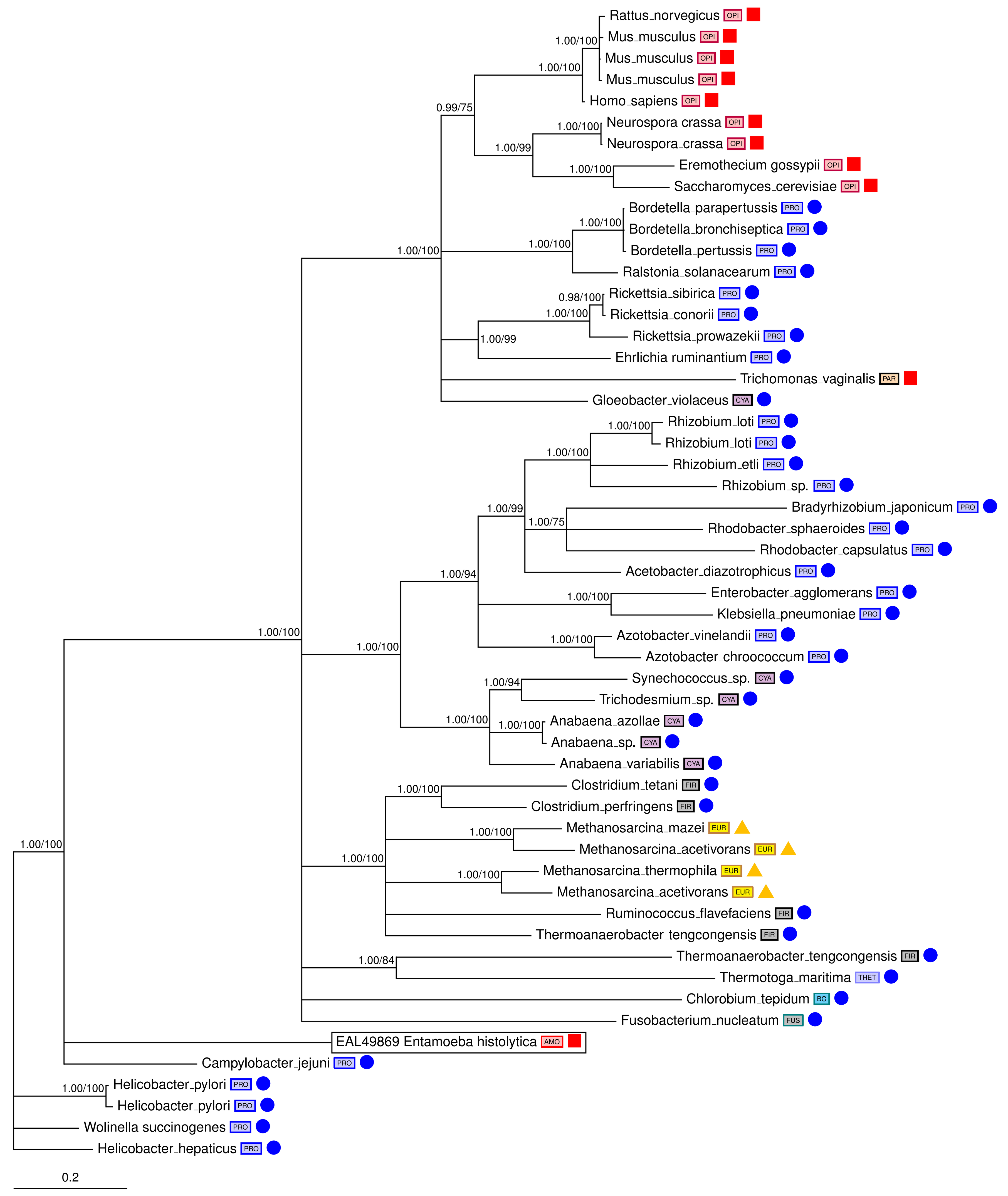

TN251

```
                Candy accession: Q581W1_9TRYPlmtc
               RefSeq accession: XP_847054.1
              Uniprot accession: Q581W1_9TRYP
                       Comments: LGT - KINETOPLASTIDS TWO NODES
               Species affected: LM,LT,LA,LD,LT2,TB,TC
          Adjacent taxa in tree: Proteobacteria
EC annotation - (Blast/Profile): EC:1.5.1.33
                    PHOBIUS SP: 0
                   PHOBIUS TMD: 0
             RefSeq annotation: pteridine reductase
       Name of enzyme/protein: Pteridine reductase
        KEGG PATHWAY - level 1: Reaction
        KEGG PATHWAY - level 2: Reaction
```

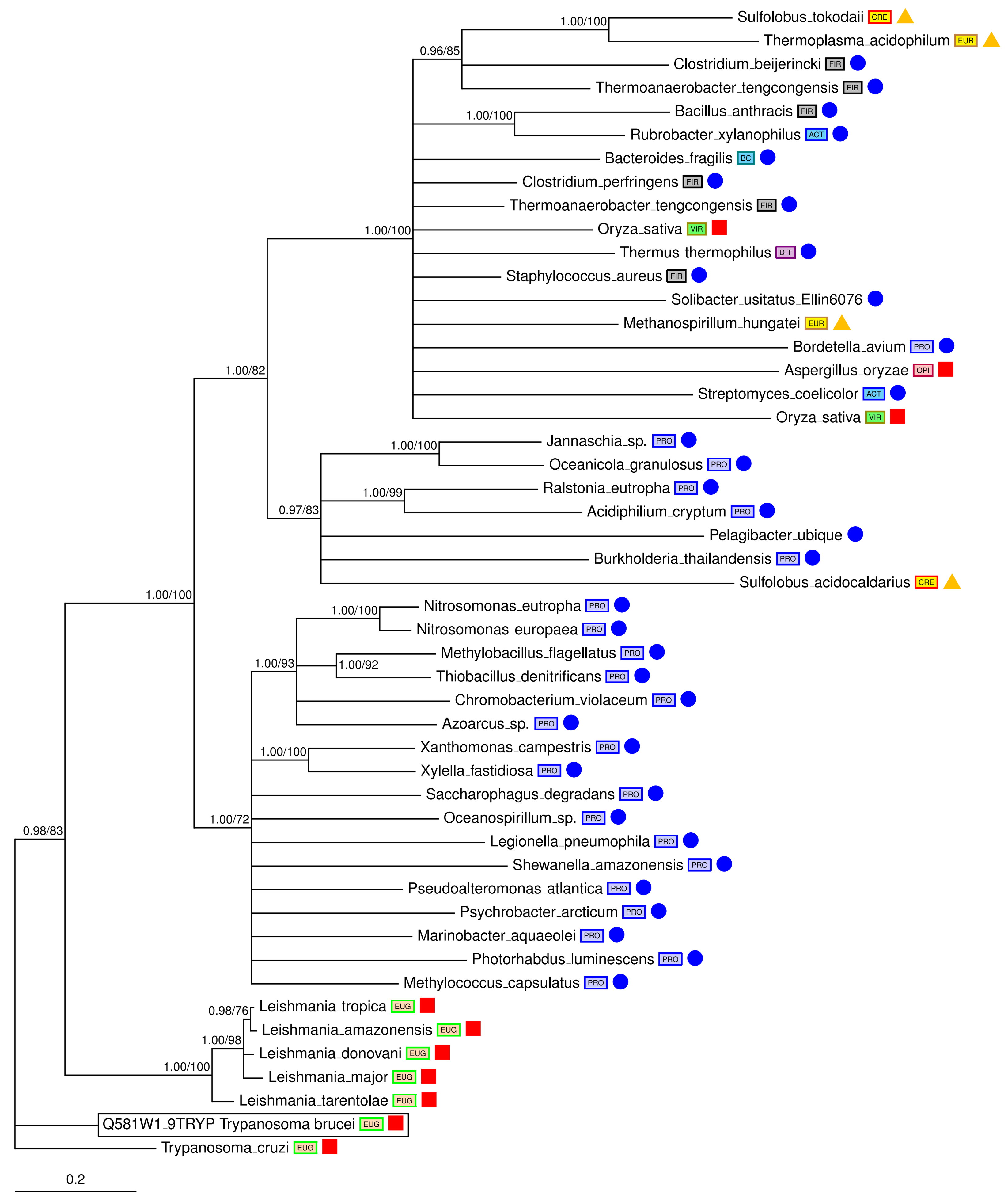

TN252

```
              Candy accession: TC0085
            RefSeq accession: XP_803162.1
           Uniprot accession: PRCMB_TRYCR
                    Comments: LGT - TC TWO NODES
            Species affected: TC
       Adjacent taxa in tree: Firmicutes - Clostridium
EC annotation - (Blast/Profile): EC:5.1.1.4
                  PHOBIUS SP: 0
                 PHOBIUS TMD: 0
           RefSeq annotation: proline racemase
    Name of enzyme/protein: Proline racemase
    KEGG PATHWAY - level 1: Amino Acid Metabolism
    KEGG PATHWAY - level 2: Arginine and proline metabolism
```

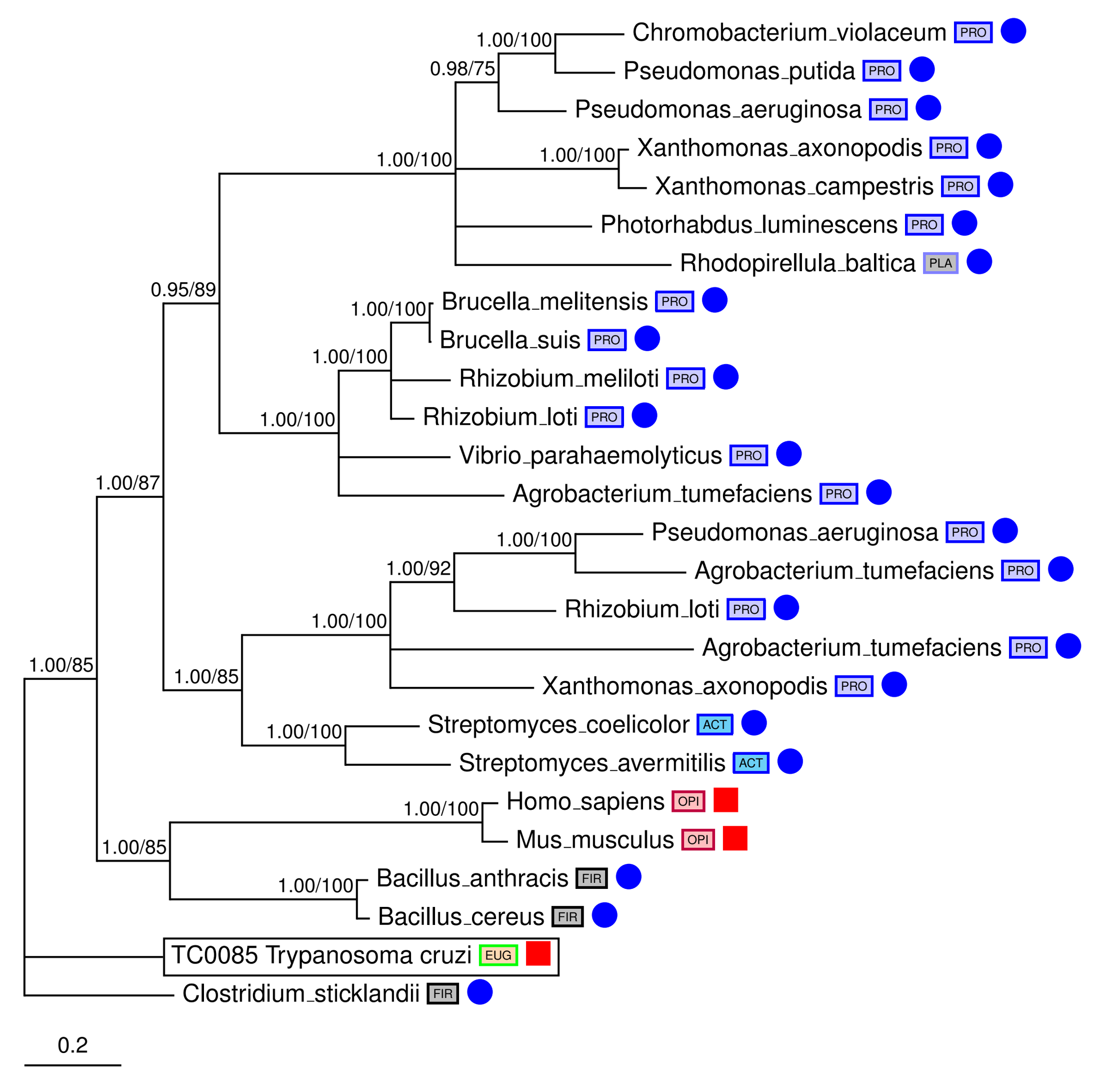

TN253

Candy accession: TV94939050
RefSeq accession: XP_001307535.1
Uniprot accession: Q86S09_TRIVA
Comments: LGT - TV TWO NODES
Species affected: TV
Adjacent taxa in tree: Firmicutes - Clostridium
EC annotation - (Blast/Profile): EC:1.1.1.-
PHOBIUS SP: 0
PHOBIUS TMD: 0
RefSeq annotation: alcohol dehydrogensae
Name of enzyme/protein: Oxidoreductases
KEGG PATHWAY - level 1: Reaction
KEGG PATHWAY - level 2: Reaction

Candy accession: Q7QXR9_GIALA
RefSeq accession: XP_001706932.1
Uniprot accession: Q7QXR9_GIALA
Comments: LGT - GI,SB TWO NODES
Species affected: GI,SB
Adjacent taxa in tree: Prokaryotes
EC annotation - (Blast/Profile): EC:1.1.1.1
PHOBIUS SP: 0
PHOBIUS TMD: 0
RefSeq annotation: Alcohol dehydrogenase 3 lateral transfer candidate
Name of enzyme/protein: alcohol dehydrogenase
KEGG PATHWAY - level 1: Carbohydrate Metabolism, Lipid Metabolism, Amino Acid Metabolism
KEGG PATHWAY - level 2: Glycolysis / Gluconeogenesis, Fatty acid metabolism, Glycine, serine and threonine metabolism

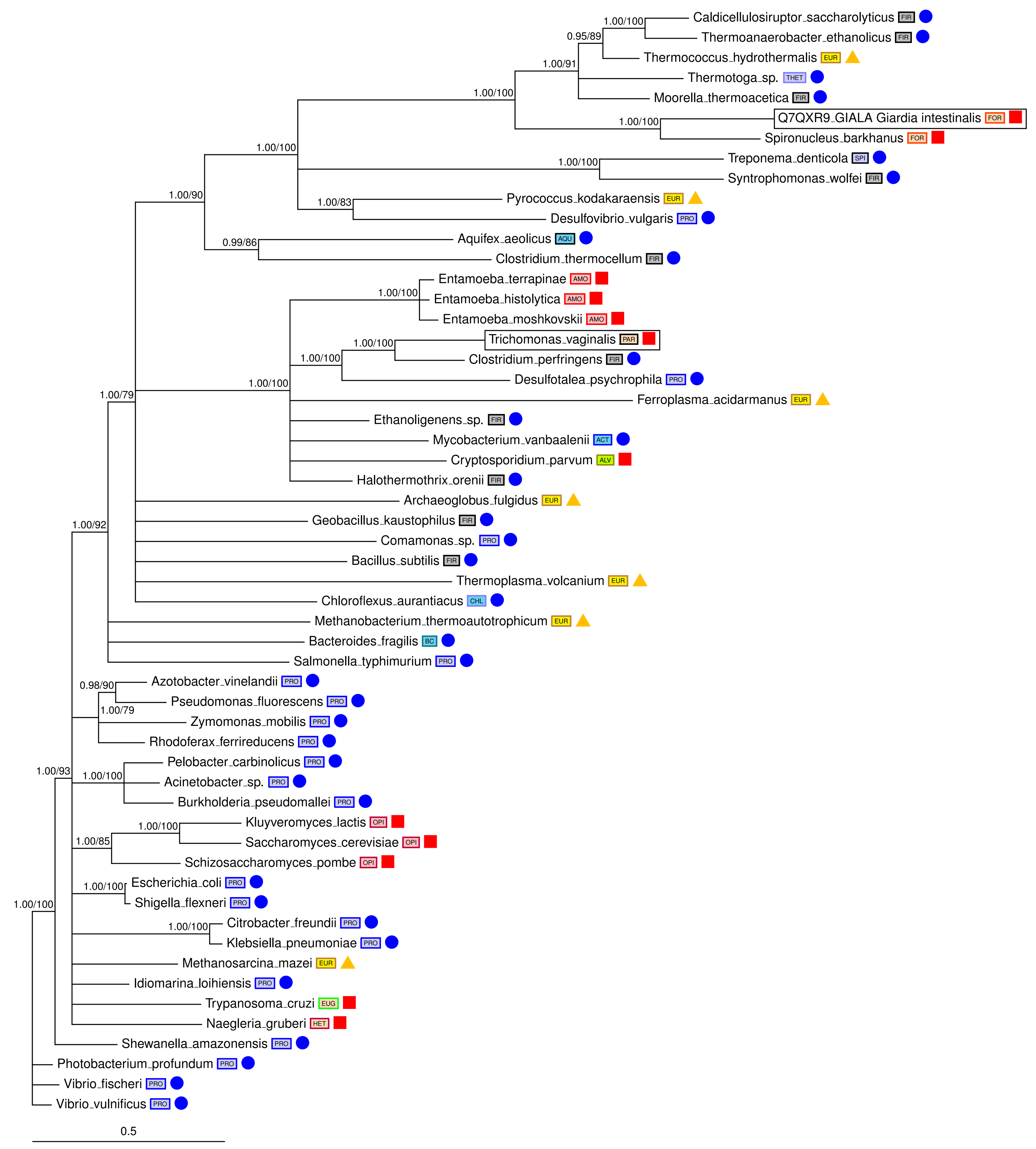

TN254

```
            Candy accession: TC0236
           RefSeq accession: XP_821790.1
          Uniprot accession: Q4E5B9_TRYCR
                   Comments: LGT - KINETOPLASTIDS TWO NODES
           Species affected: LM,TB,TC
       Adjacent taxa in tree: Bacteria
EC annotation - (Blast/Profile): EC:3.5.1.-
                 PHOBIUS SP: Y
                PHOBIUS TMD: 0
          RefSeq annotation: NAD-dependent deacetylase
   Name of enzyme/protein: Hydrolases (NAD-dependent deacetylase)
    KEGG PATHWAY - level 1: Reaction
    KEGG PATHWAY - level 2: Reaction
```

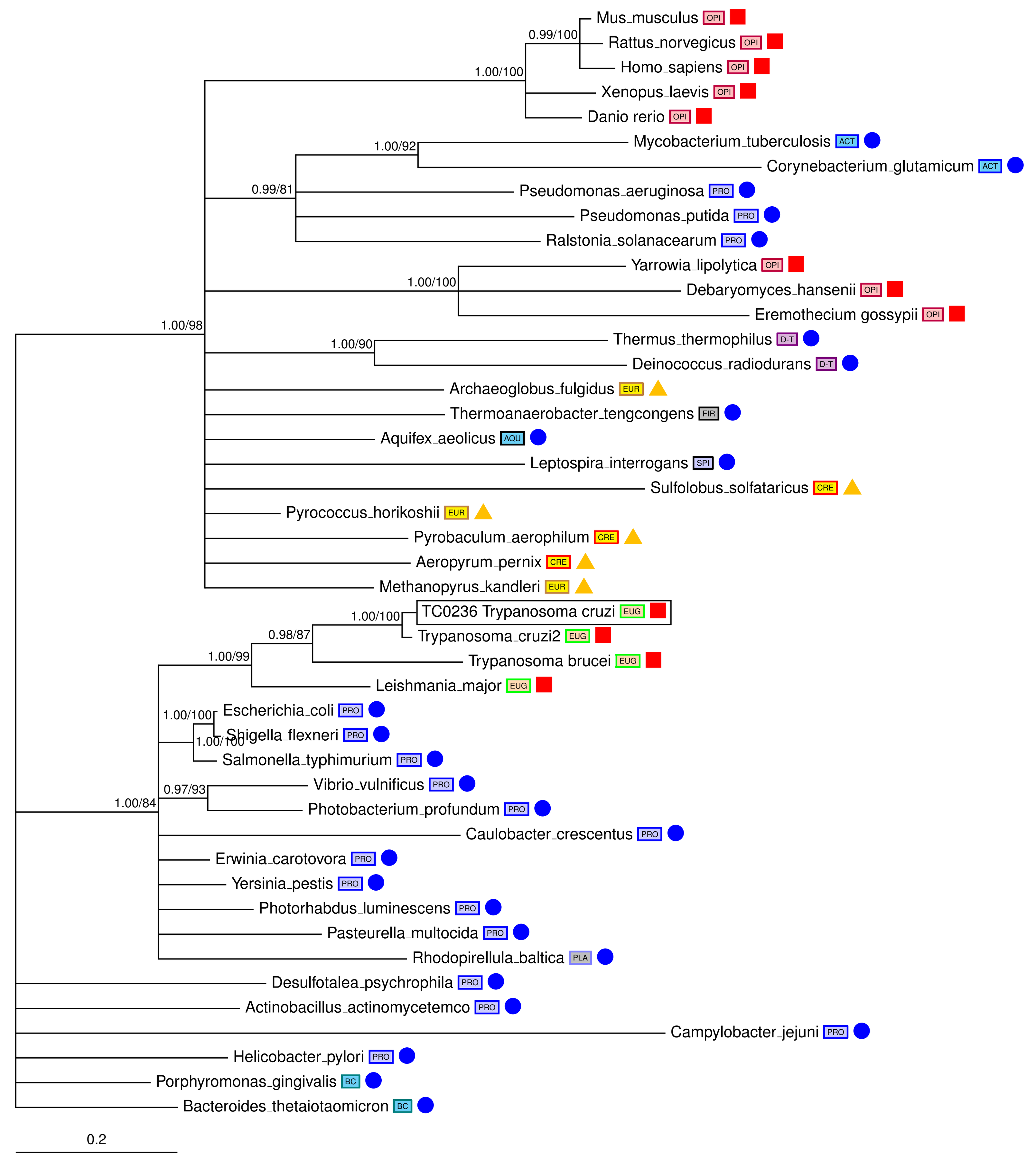

TN255

```
                Candy accession: Q9U0V9_LEIMA
               RefSeq accession: XP_001683404.1
              Uniprot accession: Q9U0V9_LEIMA
                       Comments: LGT - KINETOPLASTIDS TWO NODES
               Species affected: LM, TB,TC
          Adjacent taxa in tree: Bacteria
EC annotation - (Blast/Profile): EC:2.3.1.16
                     PHOBIUS SP: 0
                    PHOBIUS TMD: 0
              RefSeq annotation: 3-ketoacyl-coa thiolase-like protein
        Name of enzyme/protein: acetyl-CoA C-acyltransferase
         KEGG PATHWAY - level 1: Lipid Metabolism, Amino Acid Metabolism
         KEGG PATHWAY - level 2: Fatty acid elongation, Fatty acid
                                 metabolism,  Valine, leucine and
                                 isoleucine degradation
```

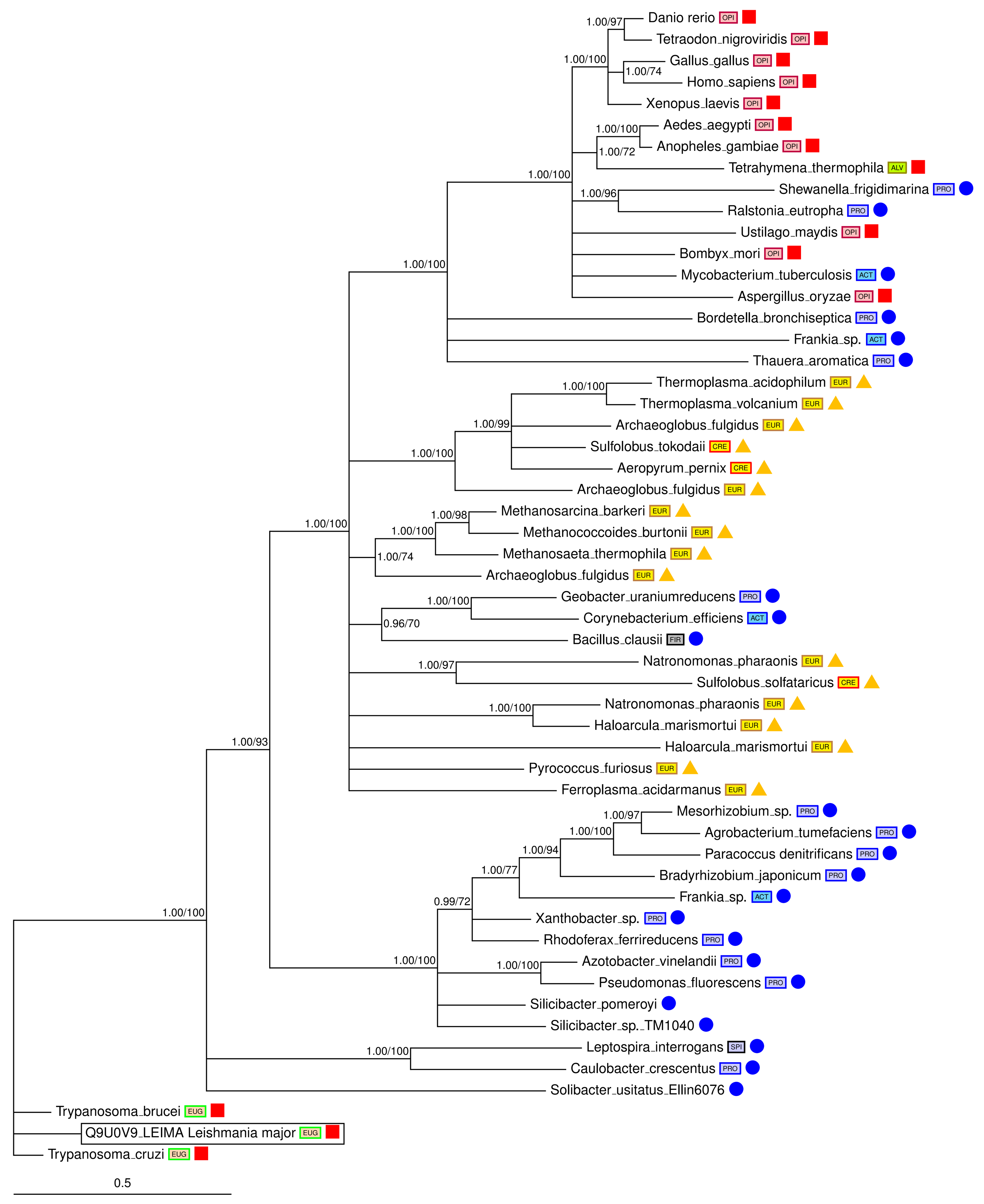

TN256

```
                Candy accession: LM0111
               RefSeq accession: XP_001685791.1
              Uniprot accession: Q4Q4F7_LEIMA
                       Comments: LGT - LM  TWO NODES
               Species affected: LM
           Adjacent taxa in tree: Protobacteria
EC annotation - (Blast/Profile): EC:1.1.1.9
                     PHOBIUS SP: 0
                    PHOBIUS TMD: 0
              RefSeq annotation: d-xylulose reductase
         Name of enzyme/protein: D-xylulose reductase
        KEGG PATHWAY - level 1: Carbohydrate Metabolism
        KEGG PATHWAY - level 2: Pentose and glucuronate interconversions
```

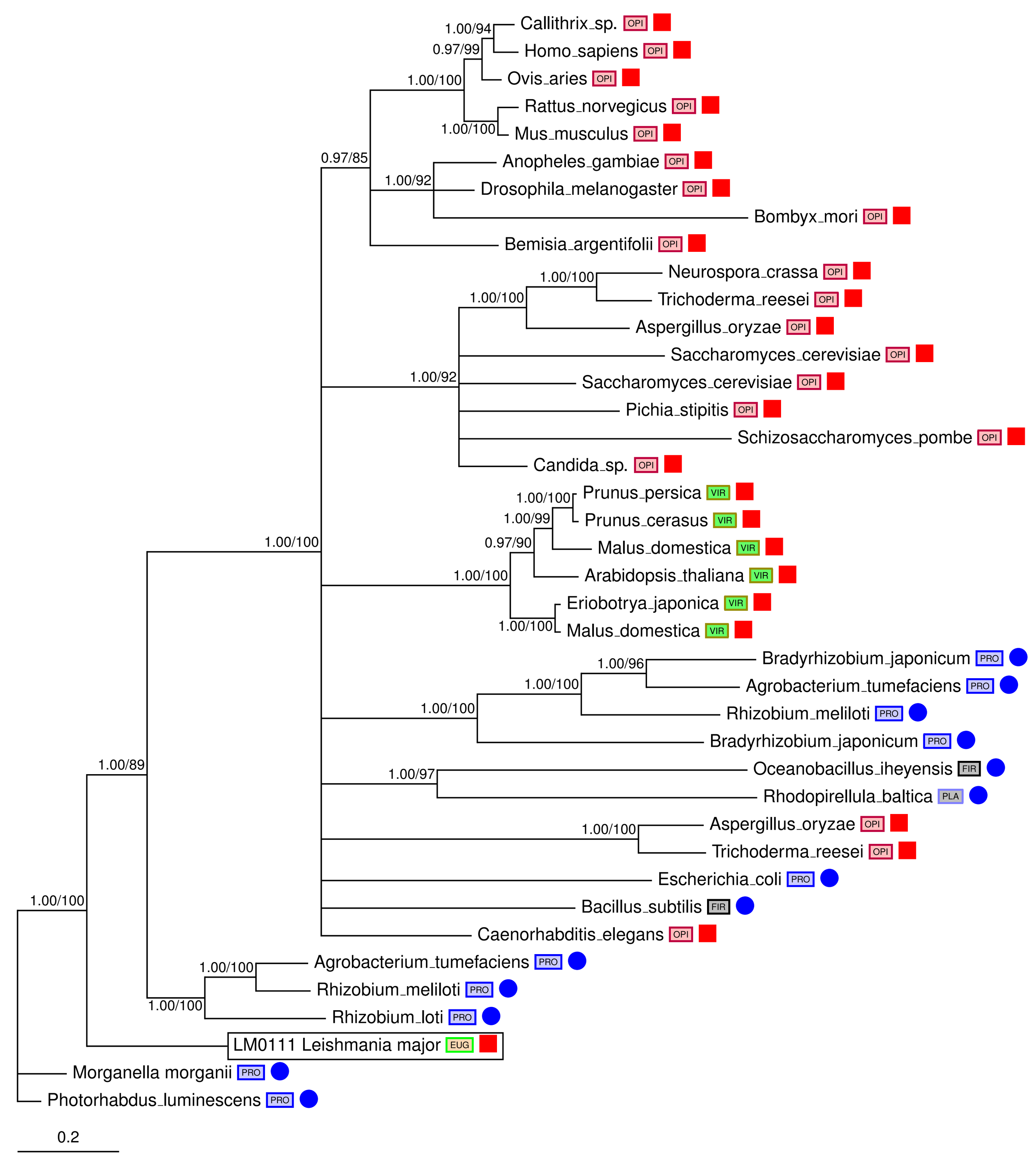

TN257

```
                 Candy accession: Q4Q784_LEIMAtc
                RefSeq accession: XP_001684814.1
               Uniprot accession: Q4Q784_LEIMA
                        Comments: LGT - LM TWO NODES
                Species affected: LM
           Adjacent taxa in tree: Firmicutes
EC annotation - (Blast/Profile): EC:1.1.1.1
                      PHOBIUS SP: 0
                     PHOBIUS TMD: 0
               RefSeq annotation: alcohol dehydrogenase
        Name of enzyme/protein: alcohol dehydrogenase
           KEGG PATHWAY - level 1: Carbohydrate Metabolism, Lipid
                                   Metabolism, Amino Acid Metabolism
           KEGG PATHWAY - level 2: Glycolysis / Gluconeogenesis, Fatty acid
                                   metabolism, Glycine, serine and
                                   threonine metabolism
```

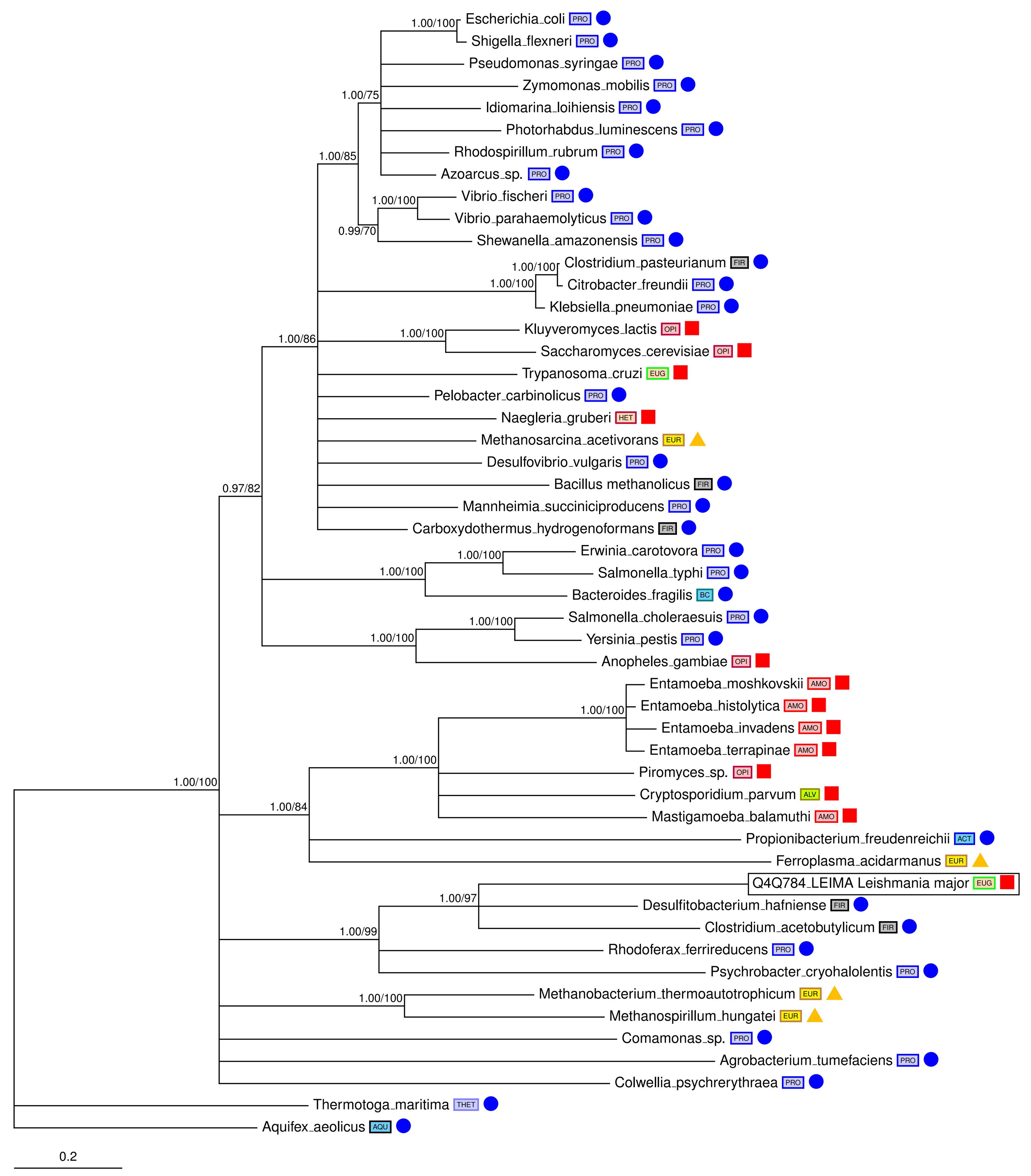

TN258

```
                 Candy accession: LM0063tc
                RefSeq accession: XP_001684577.1
               Uniprot accession: Q4Q7X1_LEIMA
                        Comments: LGT - KINETOPLASTIDS TWO NODES
                Species affected: LM,TC
           Adjacent taxa in tree: Bacteria/phage
EC annotation - (Blast/Profile): EC:1.4.1.4
                      PHOBIUS SP: 0
                     PHOBIUS TMD: 0
               RefSeq annotation: glutamate dehydrogenase
          Name of enzyme/protein: Glutamate dehydrogenase (NADP+)
          KEGG PATHWAY - level 1: Amino Acid Metabolism, Energy Metabolism
          KEGG PATHWAY - level 2: Alanine, aspartate and glutamate
                                  metabolism, Arginine and proline
                                  metabolism, Nitrogen metabolism
```

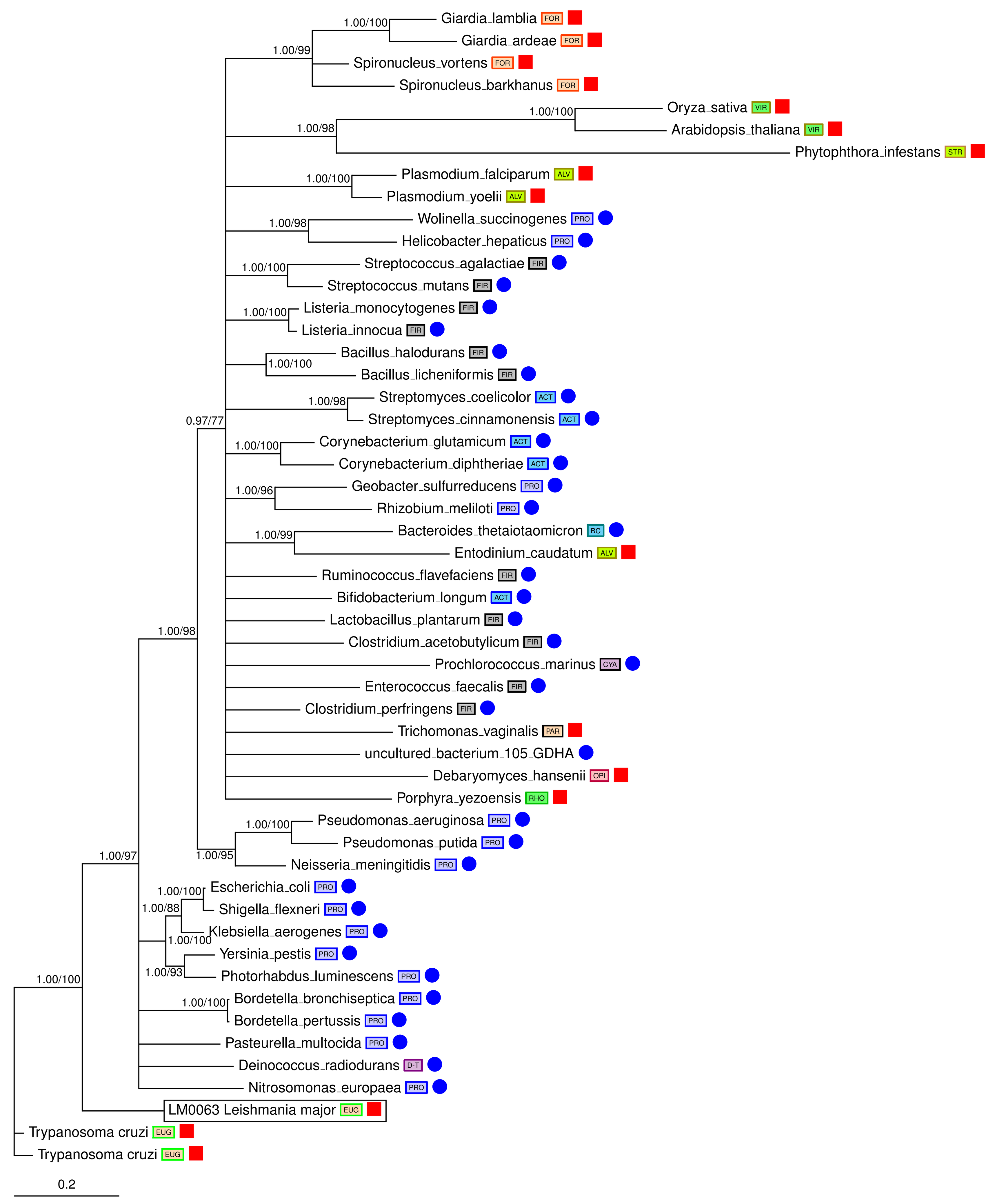

TN259

```
                Candy accession: Q4Q812_LEIMAtbtc
               RefSeq accession: XP_001684536.1
              Uniprot accession: Q4Q812_LEIMA
                       Comments: LGT - KINETOPLASTIDS TWO NODES
               Species affected: LM, TB,TC
         Adjacent taxa in tree: Protobacteria
EC annotation - (Blast/Profile): EC:1.3.8.7
                     PHOBIUS SP: Y
                    PHOBIUS TMD: 0
              RefSeq annotation: acyl-coa dehydrogenase
      Name of enzyme/protein: Acyl-coa dehydrogenase
       KEGG PATHWAY - level 1: Lipid Metabolism, Amino Acid Metabolism,
                               Metabolism of Other Amino Acids
       KEGG PATHWAY - level 2: Fatty acid metabolism, Valine, leucine
                               and isoleucine degradation, beta-Alanine
                               metabolism
```

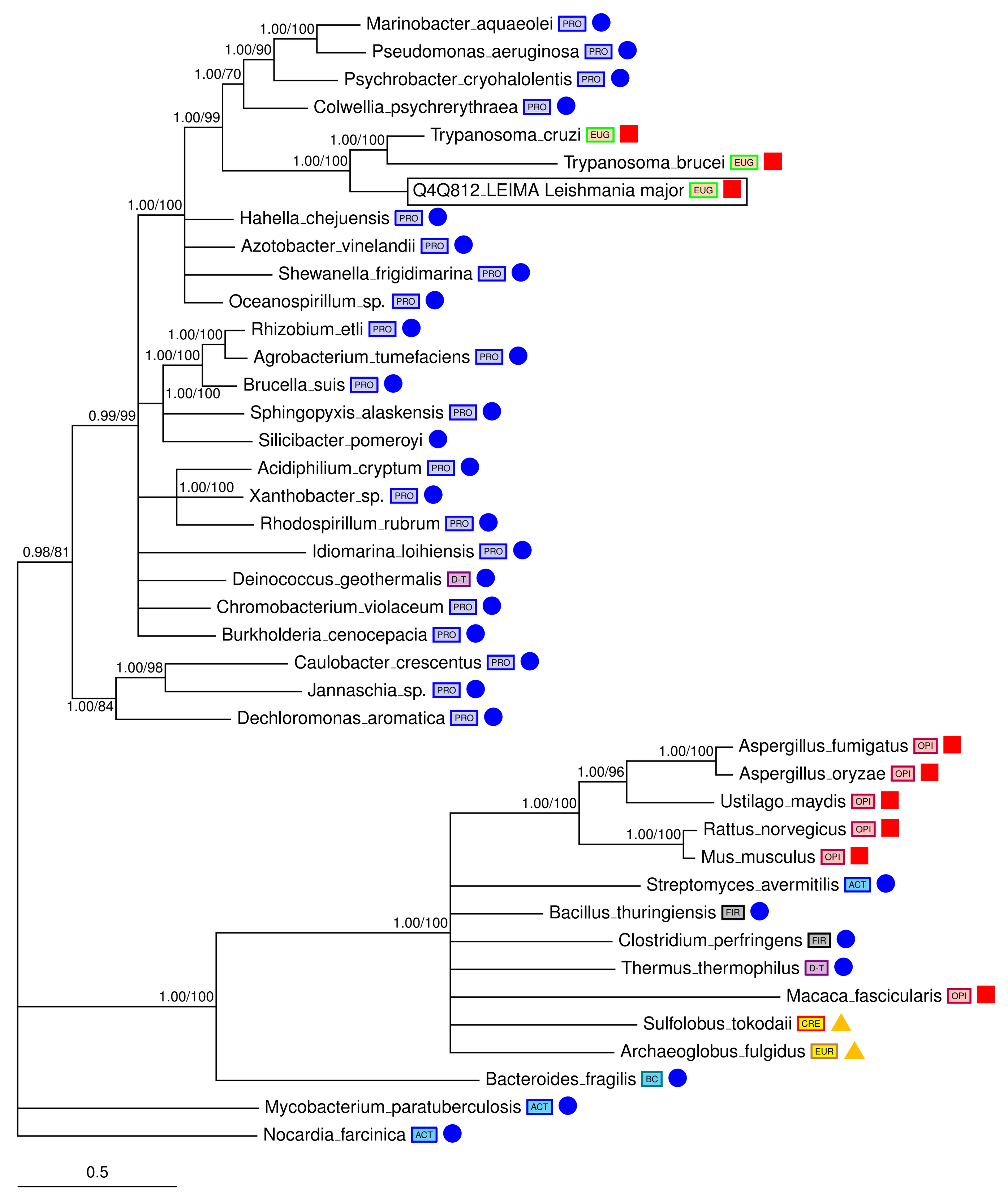

TN260

```
                Candy accession: XP_001323296
               RefSeq accession: XP_001323296
              Uniprot accession: A2E8E5_TRIVA
                       Comments: LGT - TV TWO NODES
               Species affected: TV
          Adjacent taxa in tree: Protobacteria
EC annotation - (Blast/Profile): EC:4.1.3.3
                     PHOBIUS SP: Y
                    PHOBIUS TMD: 0
              RefSeq annotation: N-acetylneuraminate lyase
         Name of enzyme/protein: N-acetylneuraminate lyase
         KEGG PATHWAY - level 1: Carbohydrate Metabolism
         KEGG PATHWAY - level 2: Amino sugar and nucleotide sugar
                                 metabolism
```

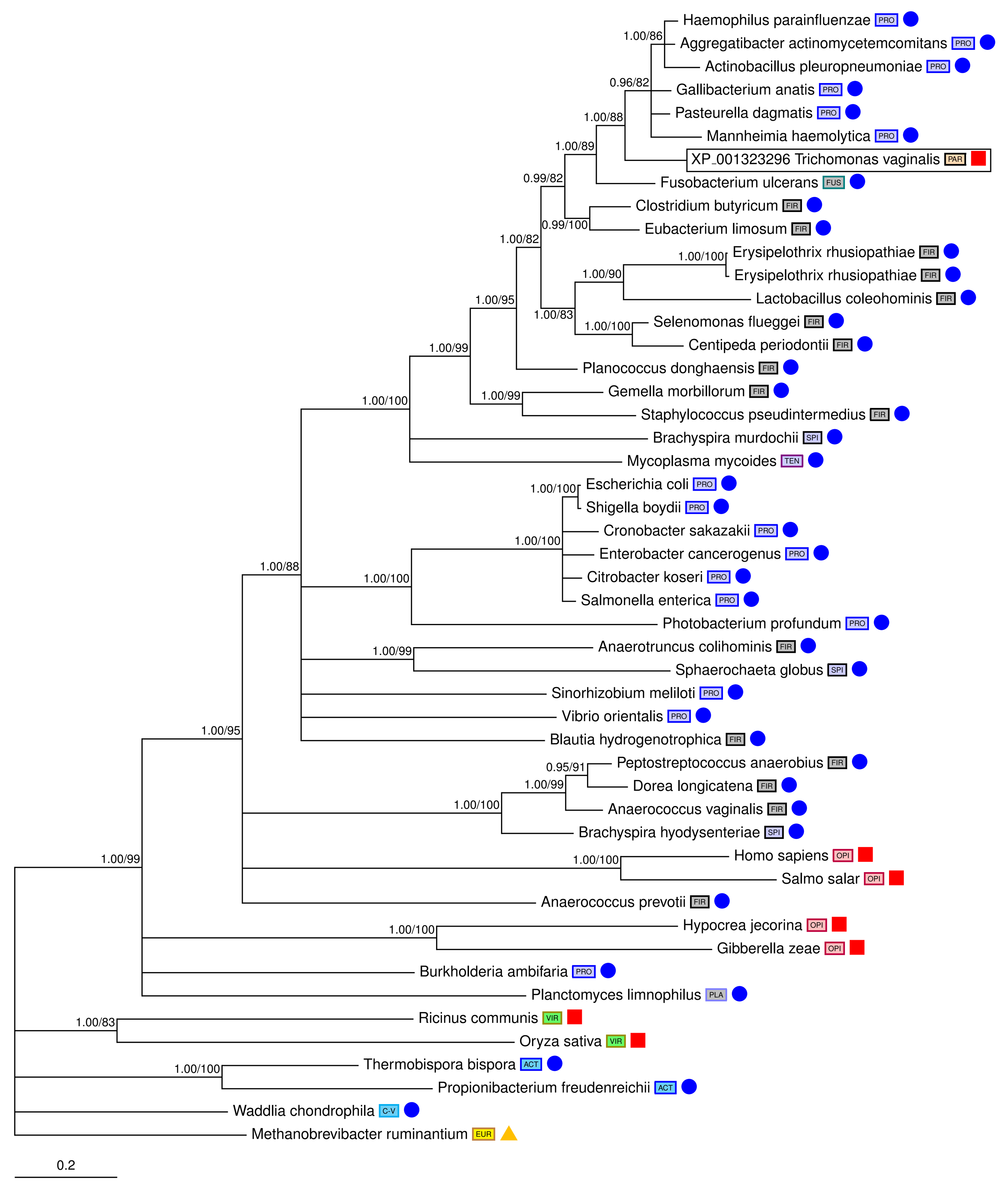

TN261

```
                 Candy accession: XP 001313252
                RefSeq accession: XP_001313252
               Uniprot accession: A2F476_TRIVA
                        Comments: LGT - TV ONLY
                Species affected: TV
           Adjacent taxa in tree: Protobacteria
EC annotation - (Blast/Profile): EC:4.1.1.-
                      PHOBIUS SP: 0
                     PHOBIUS TMD: 0
               RefSeq annotation: ferulate decarboxylase
          Name of enzyme/protein: Phenolic acid decarboxylase
          KEGG PATHWAY - level 1: Reaction
          KEGG PATHWAY - level 2: Reaction
```

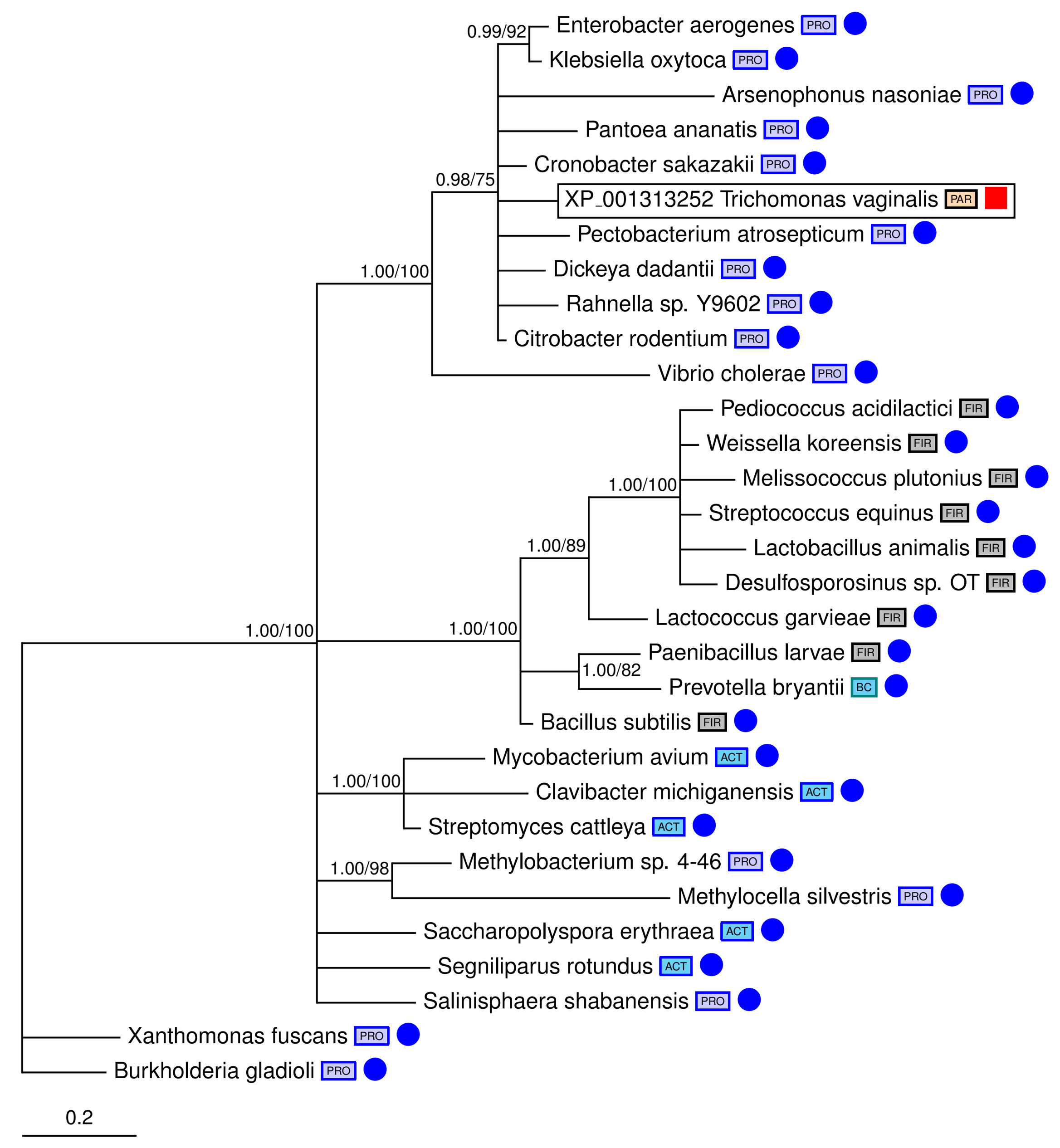

TN262

```
                 Candy accession: XP 001330346
                RefSeq accession: XP_001330346
               Uniprot accession: A2F0H0_TRIVA
                        Comments: LGT - TV TWO NODES
                Species affected: TV
           Adjacent taxa in tree: Protobacteria
EC annotation - (Blast/Profile): EC:2.2.1.2
                     PHOBIUS SP: 0
                    PHOBIUS TMD: 0
               RefSeq annotation: Transaldolase A
         Name of enzyme/protein: Transaldolase
         KEGG PATHWAY - level 1: Carbohydrate Metabolism
         KEGG PATHWAY - level 2: Pentose phosphate pathway
```

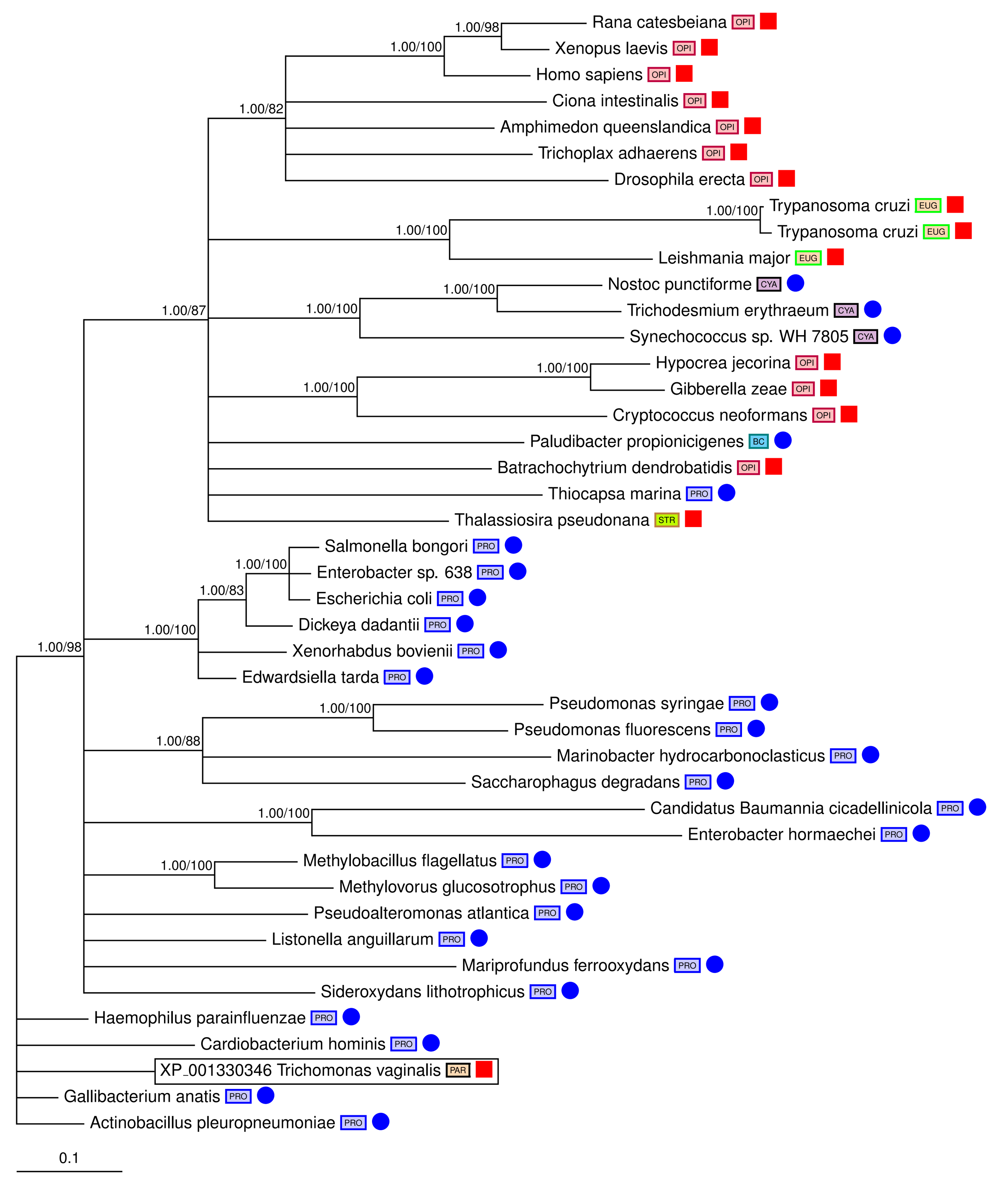

TN263

```
                Candy accession: XP_001316088
               RefSeq accession: XP_001316088
              Uniprot accession: A2ETX7_TRIVA
                       Comments: LGT - TV TWO NODES
               Species affected: TV,FUNGI
           Adjacent taxa in tree: Bacteria
EC annotation - (Blast/Profile): EC:3.2.1.51
                     PHOBIUS SP: 0
                    PHOBIUS TMD: 1
              RefSeq annotation: Calx-beta domain containing protein
         Name of enzyme/protein: alpha-L-fucosidase
         KEGG PATHWAY - level 1: Glycan Biosynthesis and Metabolism
         KEGG PATHWAY - level 2: Other glycan degradation
```

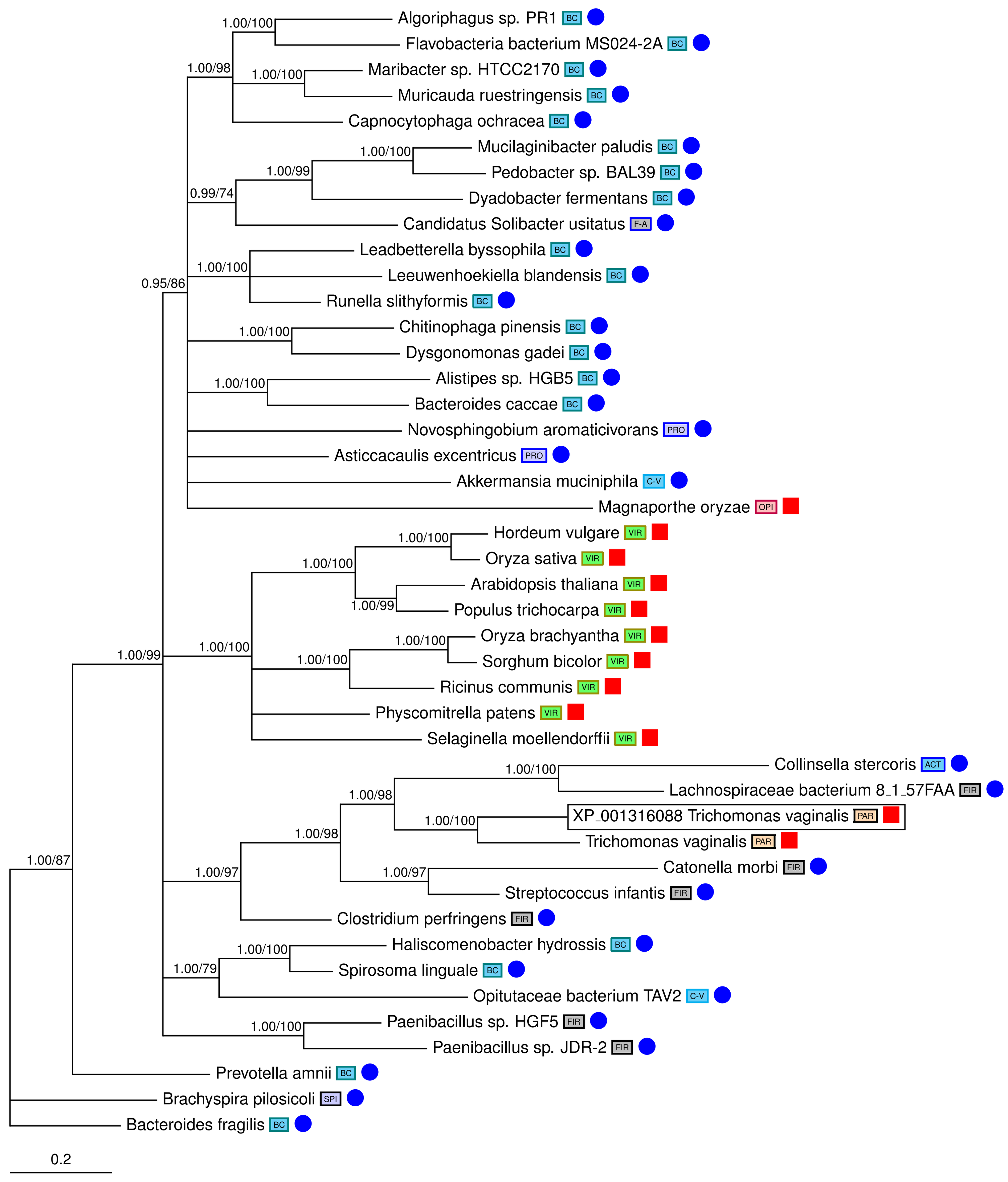

TN264

```
                 Candy accession: XP_001324859
                RefSeq accession: XP_001324859
               Uniprot accession: A2E3Y1_TRIVA
                        Comments: LGT - TV TWO NODES
                Species affected: TV,AMOEBOZOA
           Adjacent taxa in tree: Bacteroidetes/Chlorobi
EC annotation - (Blast/Profile): EC:3.2.1.45
                      PHOBIUS SP: 0
                     PHOBIUS TMD: 0
               RefSeq annotation: O-Glycosyl hydrolase family 30 protein
         Name of enzyme/protein: glucosylceramidase
         KEGG PATHWAY - level 1: Glycan Biosynthesis and Metabolism,
                                  Lipid Metabolism
         KEGG PATHWAY - level 2: Other glycan degradation,  Sphingolipid
                                  metabolism
```

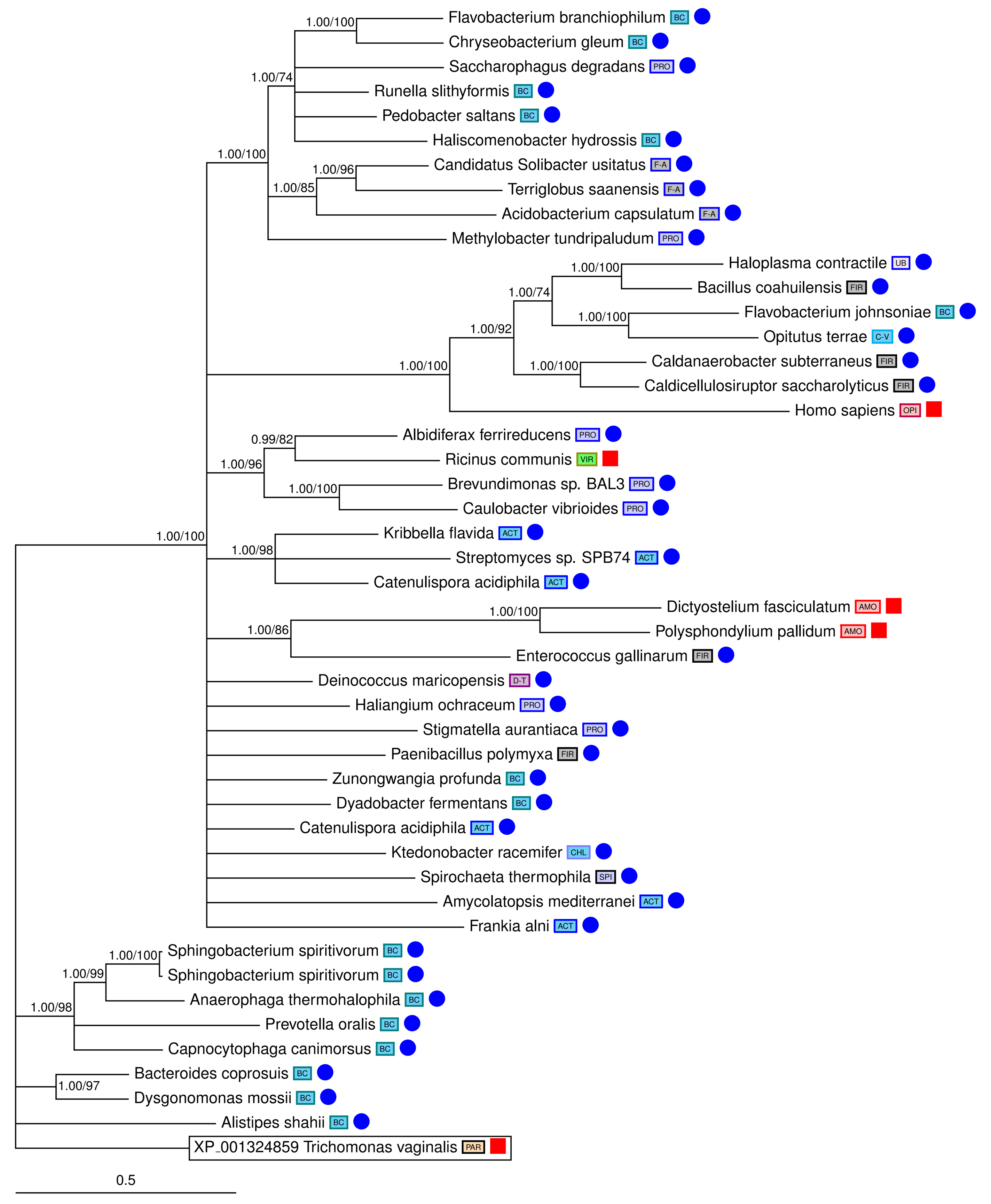

TN265

```
           Candy accession: XP_001319692
          RefSeq accession: XP_001319692
         Uniprot accession: A2EIP2_TRIVA
                  Comments: LGT - TV TWO NODES
          Species affected: TV,FUNGI
     Adjacent taxa in tree: Bacteroidetes/Chlorobi - Maribacter
EC annotation - (Blast/Profile): EC:3.2.1.18
                PHOBIUS SP: 0
               PHOBIUS TMD: 0
         RefSeq annotation: BNR/Asp-box repeat family protein
    Name of enzyme/protein: exo-alpha-sialidase
     KEGG PATHWAY - level 1: Glycan Biosynthesis and Metabolism,
                             Lipid Metabolism
     KEGG PATHWAY - level 2: Other glycan degradation,  Sphingolipid
                             metabolism
```

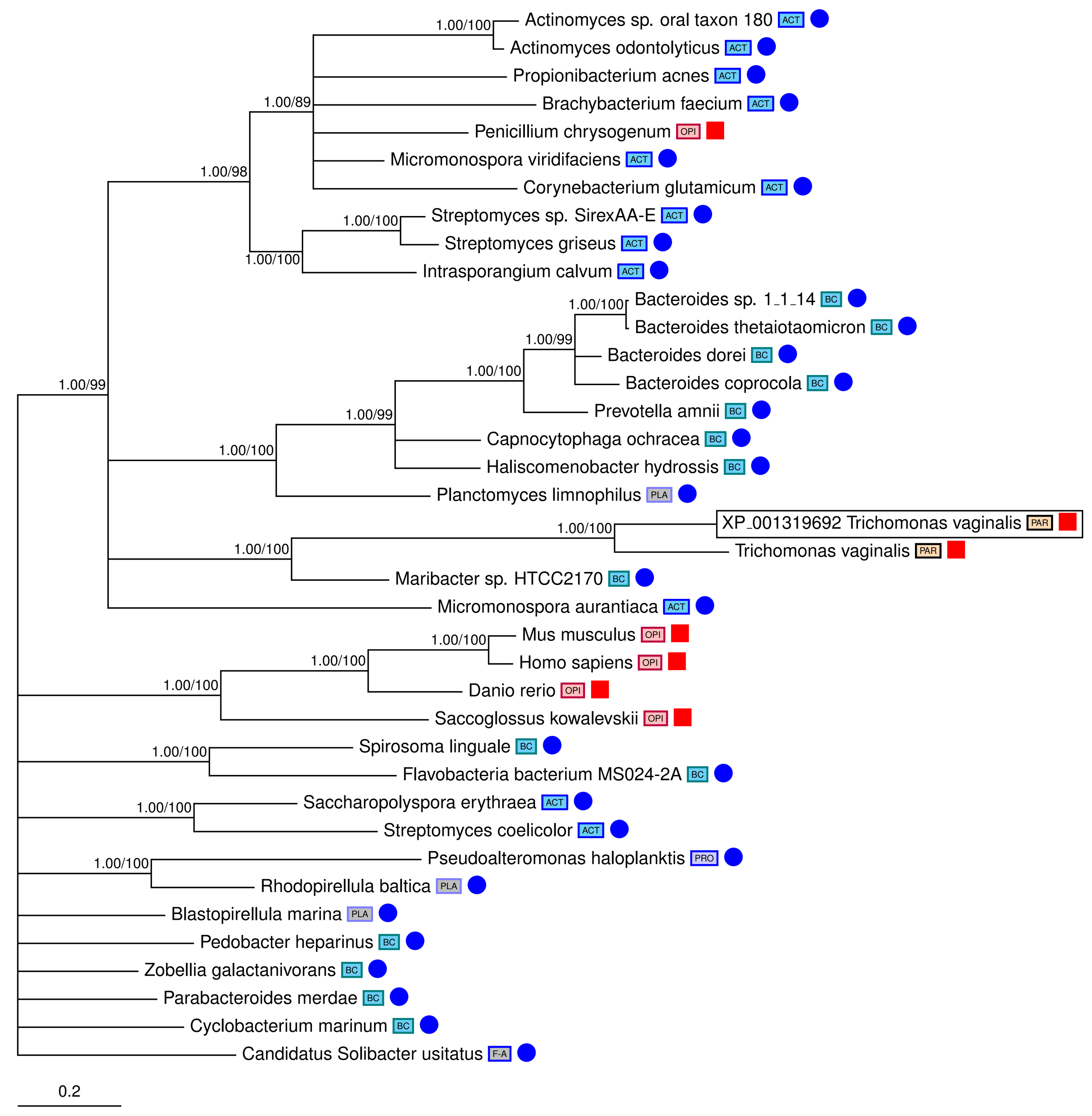

Supplement: Additional file 5 — Phylogenetic trees supporting prokaryote-to-eukaryote lateral gene transfers (LGTs). Figure illustrating the phylogenies for the candidate LGTs from prokaryotes to eukaryotes supported by at least one well-supported node in the phylogenetic tree. [file gb-2013-14-2-r19-S5.PDF]
