# Supplementary material for: Patterns of prokaryotic lateral gene transfers affecting parasitic microbial eukaryotes
Source: Genome Biol. 2013 Feb 25;14(2):R19. doi: 10.1186/gb-2013-14-2-r19 (PMC4053834; doi:10.1186/gb-2013-14-2-r19)

Additional File 6. Potential eukaryote-to-eukaryote LGTs

See legend of Additional File 5 for details, including the taxa color key. These trees recovered one or more eukaryote taxa in a polytomy or as part of the same well-supported clan supporting, or which cannot reject, potential LGT between eukaryotes. (trees numbered EEXXX). Taxon sampling suggests, for several cases, at least one initial LGT from a prokaryote donor to a eukaryote followed by potential eukaryote to eukaryote LGT.

Entries in the table of contents below are clickable hyperlinks to the tree figures.

21 September, 2012

Contents

EE001 2
EE002 3
EE003 4
EE004 5
EE005 6
EE006 7
EE007 8
EE008 9
EE009 10
EE010 11
EE011 12
EE012 13
EE013 14
EE014 15
EE015 16
EE016 17
EE017 18
EE018 19
EE019 20
EE020 21
EE021 22
EE022 23
EE023 24
EE024 25
EE025 26
EE026 27

EE001

```
           Candy accession: TV81529518
          RefSeq accession: XP_001583562.1
         Uniprot accession: Q6XKE2_TRIVA
                  Comments: LGT - E-E LGT OR DEAP LGT INTO
                            METAMONADA (DIMPLONADS+PARABASALA)?
          Species affected: TV
     Adjacent taxa in tree: Bacteria
EC annotation - (Blast/Profile): na
                PHOBIUS SP: 0
               PHOBIUS TMD: 0
         RefSeq annotation: Hydrogenosomal oxygen reductase
    Name of enzyme/protein: Protein containing metallo-beta-
                            lactamase/flavodoxin domain
     KEGG PATHWAY - level 1: Other function
     KEGG PATHWAY - level 2: na

           Candy accession: Q86QZ1_GIALA
          RefSeq accession: XP_001707670.1
         Uniprot accession: Q86QZ1_GIAIN
                  Comments: LGT - E-E LGT OR DEAP LGT INTO
                            METAMONADA (DIMPLONADS+PARABASALA)?
          Species affected: GI,SB,TV
     Adjacent taxa in tree: Bacteria
EC annotation - (Blast/Profile): na
                PHOBIUS SP: 0
               PHOBIUS TMD: 0
         RefSeq annotation: A-type flavoprotein lateral transfer
                            candidate
    Name of enzyme/protein: Protein containing metallo-beta-
                            lactamase/flavodoxin domain
     KEGG PATHWAY - level 1: Other function
     KEGG PATHWAY - level 2: na
```

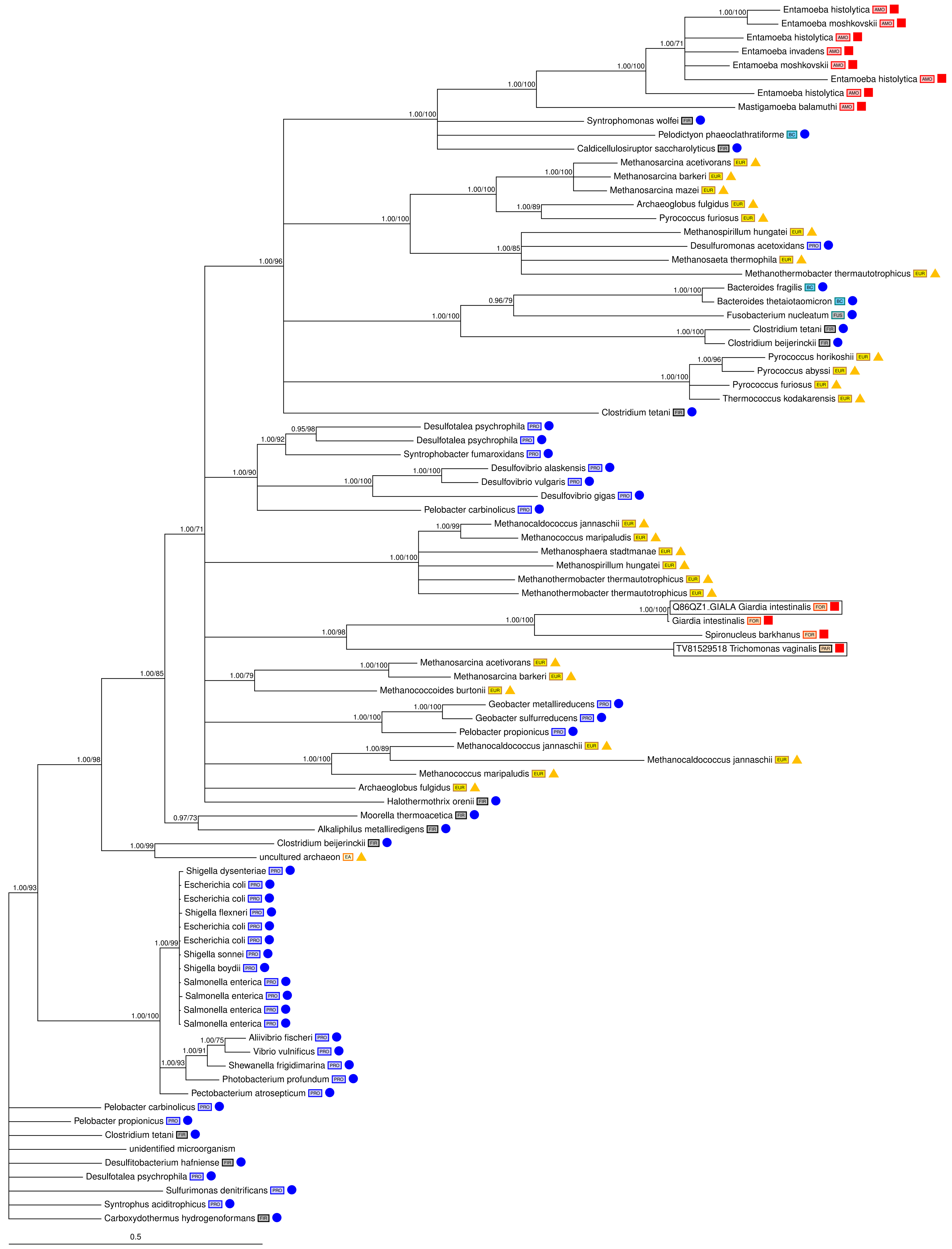

EE002

Candy accession: TV92066132
RefSeq accession: XP_001319096.1
Uniprot accession: A2EKG5_TRIVA
Comments: LGT - POSSIBLE LGT BETWEEN APICOMPLEXA AND PARABASALA
Species affected: TV
Adjacent taxa in tree: Apicomplexa
EC annotation - (Blast/Profile): EC:6.3.1.1
PHOBIUS SP: 0
PHOBIUS TMD: 0
RefSeq annotation: hypothetical protein
Name of enzyme/protein: Aspartate-ammonia ligase
KEGG PATHWAY - level 1: Amino Acid Metabolism, Metabolism of Other Amino Acids, Energy Metabolism
KEGG PATHWAY - level 2: Alanine, aspartate and glutamate metabolism, Cyanoamino acid metabolism, Nitrogen metabolism

Candy accession: Q5CPD9_CRYPV
RefSeq accession: XP_625302.1
Uniprot accession: Q5CPD9_CRYPV
Comments: LGT - POSSIBLE LGT BETWEEN APICOMPLEXA AND PARABASALA
Species affected: CP,CH
Adjacent taxa in tree: Parabasala
EC annotation - (Blast/Profile): EC:6.3.1.1
PHOBIUS SP: 0
PHOBIUS TMD: 0
RefSeq annotation: asparagine synthetase A (AsnA) like protein
Name of enzyme/protein: Aspartate-ammonia ligase
KEGG PATHWAY - level 1: Amino Acid Metabolism, Metabolism of Other Amino Acids, Energy Metabolism
KEGG PATHWAY - level 2: Alanine, aspartate and glutamate metabolism, Cyanoamino acid metabolism, Nitrogen metabolism

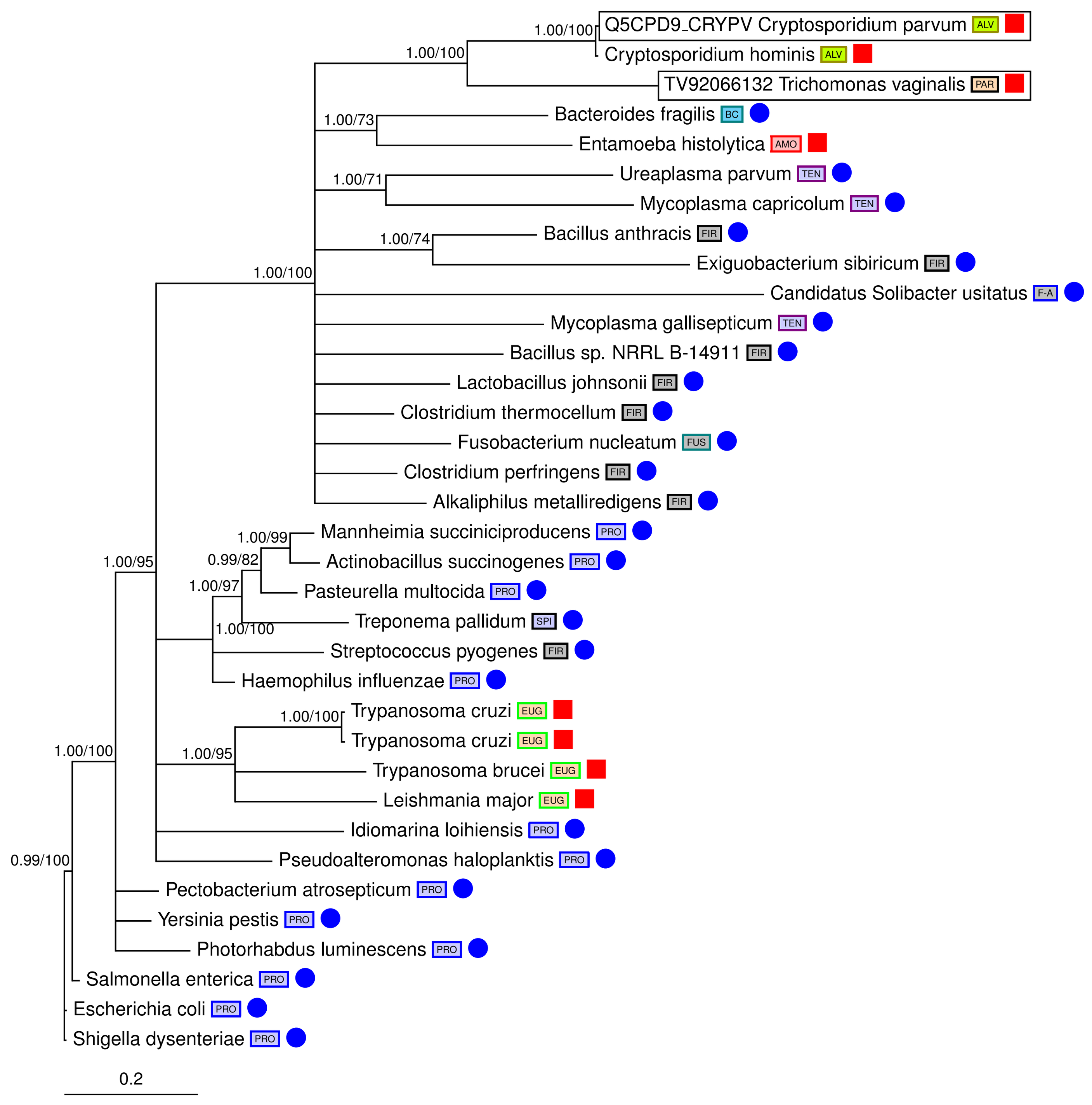

EE003

Candy accession: Q95WU5_GIALA
RefSeq accession: XP_001707929.1
Uniprot accession: Q95WU5_GIAIN
Comments: LGT - POSSIBLE LGT BETWEEN DIPLOMONAD AND AMOEBOZOA
Species affected: EH
Adjacent taxa in tree: Diplomonads
EC annotation - (Blast/Profile): EC:3.4.19.1
PHOBIUS SP: Y
PHOBIUS TMD: 0
RefSeq annotation: Alanyl dipeptidyl peptidase
Name of enzyme/protein: acylaminoacyl-peptidase
KEGG PATHWAY - level 1: Reaction
KEGG PATHWAY - level 2: Reaction

Candy accession: Q9U593_ENTHI
RefSeq accession: XP_655222.1
Uniprot accession: Q9U593_ENTHI
Comments: LGT - POSSIBLE LGT BETWEEN DIPLOMONAD AND AMOEBOZOA
Species affected: GI
Adjacent taxa in tree: Amoebozoa
EC annotation - (Blast/Profile): EC:3.4.19.1
PHOBIUS SP: Y
PHOBIUS TMD: 0
RefSeq annotation: dipeptidyl-peptidase
Name of enzyme/protein: acylaminoacyl-peptidase
KEGG PATHWAY - level 1: Reaction
KEGG PATHWAY - level 2: Reaction

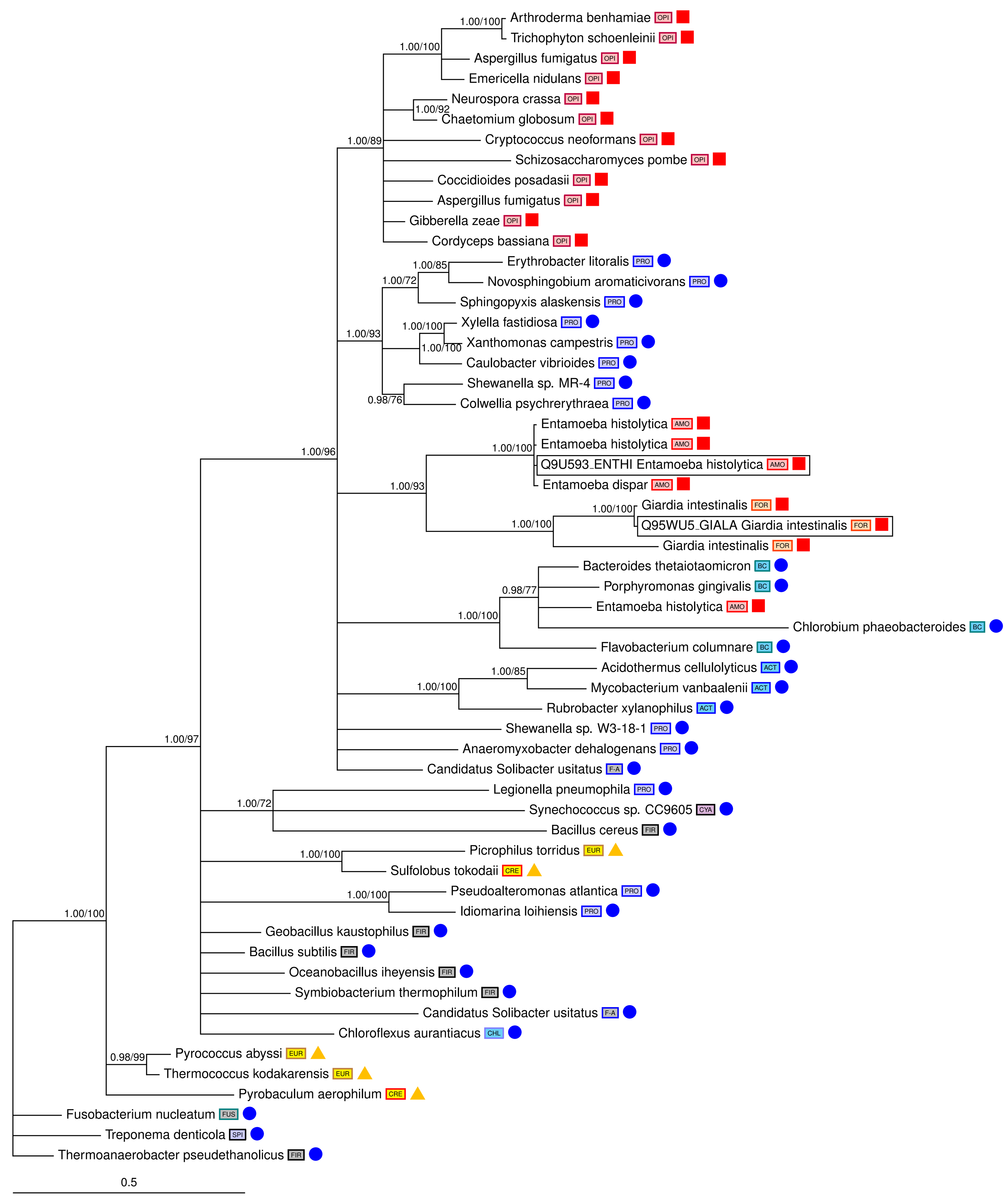

EE004

```
              Candy accession: Q50UK9_ENTHI
             RefSeq accession: XP_650664.1
            Uniprot accession: C4M148_ENTHI
                     Comments: LGT? - EH POLYTOMY WITH SC
             Species affected: EH
         Adjacent taxa in tree: Prokaryotes
EC annotation - (Blast/Profile): na
                   PHOBIUS SP: 0
                  PHOBIUS TMD: 0
            RefSeq annotation: metallo-beta-lactamase superfamily
                               protein
        Name of enzyme/protein: Predicted metallo-beta-lactamase
        KEGG PATHWAY - level 1: Other function
        KEGG PATHWAY - level 2: na
```

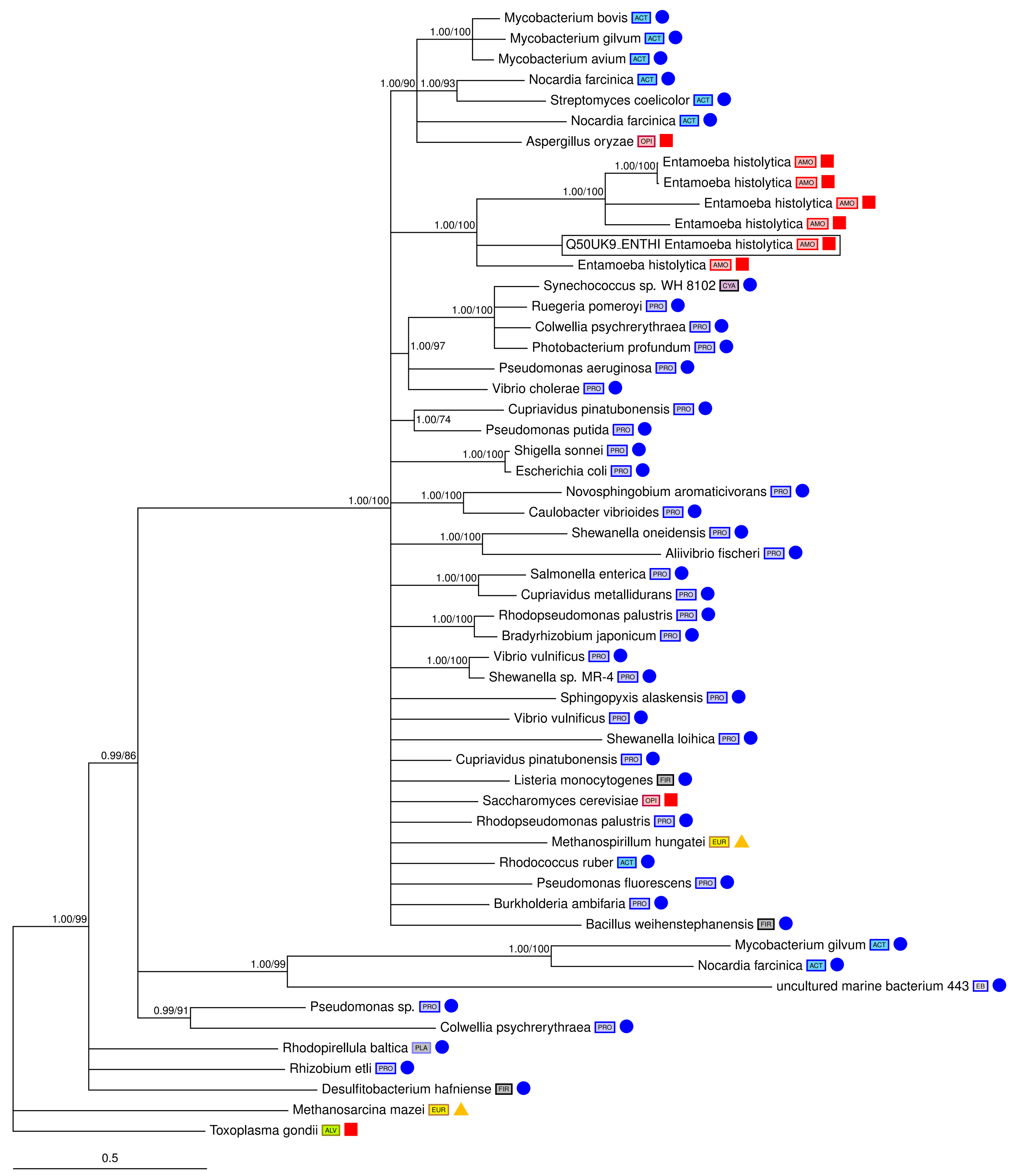

EE005

```
           Candy accession: TV82397081
          RefSeq accession: XP_001310516.1
         Uniprot accession: A2FBY7_TRIVA
                  Comments: LGT? - TV-EH POLYTOMY
          Species affected: TV
    Adjacent taxa in tree: Polytomy
EC annotation - (Blast/Profile): na
               PHOBIUS SP: 0
              PHOBIUS TMD: 0
        RefSeq annotation: hypothetical protein
   Name of enzyme/protein: Predicted tRNA(Ile)-lysidine synthase
   KEGG PATHWAY - level 1: Other function
   KEGG PATHWAY - level 2: na

           Candy accession: Q51D31_ENTHI
          RefSeq accession: XP_656114.1
         Uniprot accession: C4LXX3_ENTHI
                  Comments: LGT? - EH-TV POLYTOMY
          Species affected: EH
    Adjacent taxa in tree: Polytomy
EC annotation - (Blast/Profile): na
               PHOBIUS SP: 0
              PHOBIUS TMD: 0
        RefSeq annotation: hypothetical protein
   Name of enzyme/protein: Predicted tRNA(Ile)-lysidine synthase
   KEGG PATHWAY - level 1: Other function
   KEGG PATHWAY - level 2: na
```

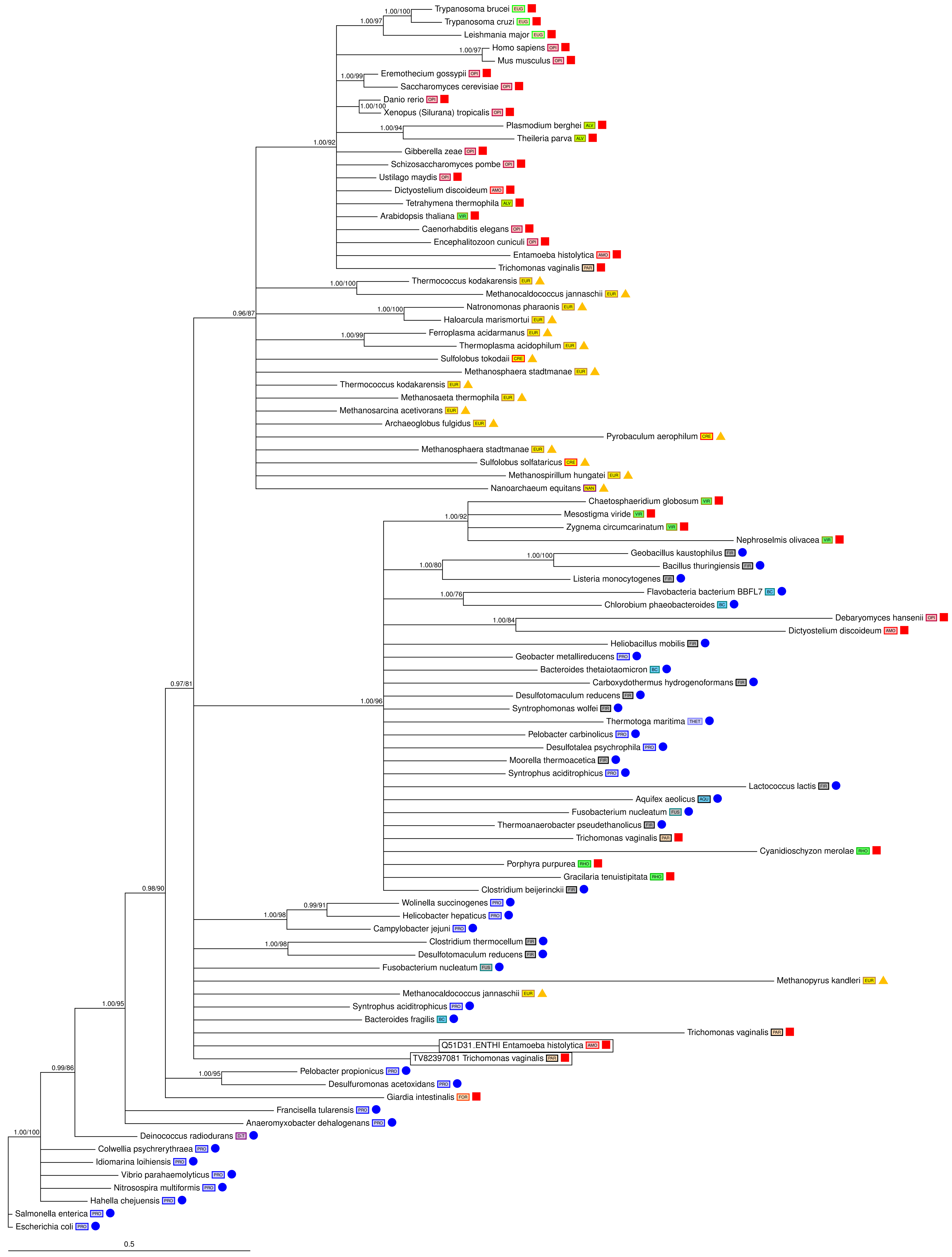

EE006

```
                Candy accession: A2FLL8_TRIVA
               RefSeq accession: XP_001307121.1
              Uniprot accession: A2FLL8_TRIVA
                       Comments: LGT? - GI-TV POLYTOMY
               Species affected: GI, TV
           Adjacent taxa in tree: Polytomy
EC annotation - (Blast/Profile): EC:6.1.1.3
              RefSeq annotation: Phosphoribulokinase family protein-
                                 related protein
         Name of enzyme/protein: Phosphoribulokinase family protein-
                                 related protein
         KEGG PATHWAY - level 1: Translation - Genetic Information
                                 Processing
         KEGG PATHWAY - level 2: Translation - Genetic Information
                                 Processing
```

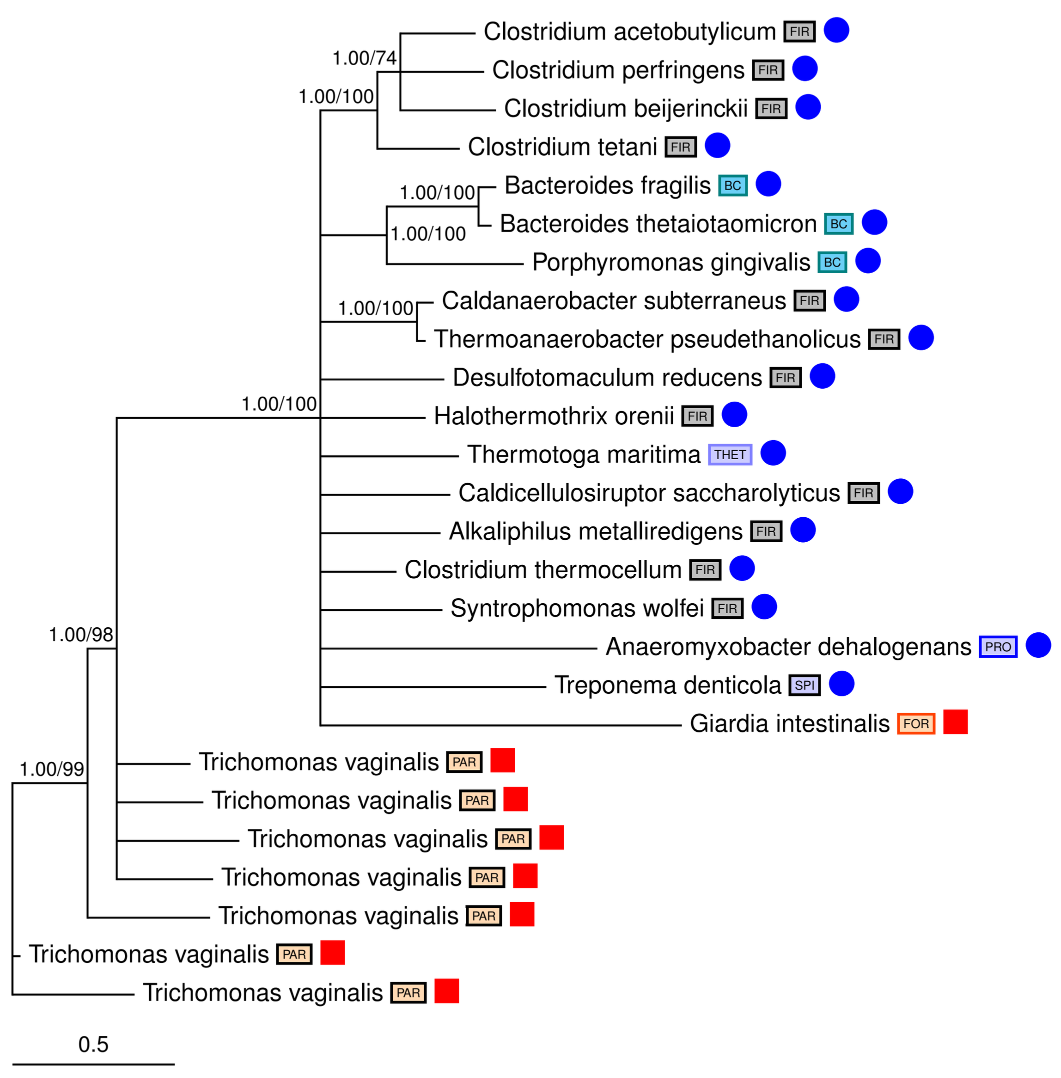

EE007

```
              Candy accession: TC0218
             RefSeq accession: XP_813761.1
            Uniprot accession: Q4DHC1_TRYCR
                     Comments: LGT? - TC - POLYTOMY WITH A BROWN ALGEA?
             Species affected: TC,PZ
         Adjacent taxa in tree: Bacteria
EC annotation - (Blast/Profile): EC:3.1.3.18
                   PHOBIUS SP: 0
                  PHOBIUS TMD: 0
            RefSeq annotation: haloacid dehalogenase hydrolase
      Name of enzyme/protein: phosphoglycolate phosphatase
      KEGG PATHWAY - level 1: Carbohydrate Metabolism
      KEGG PATHWAY - level 2: Glyoxylate and dicarboxylate metabolism
```

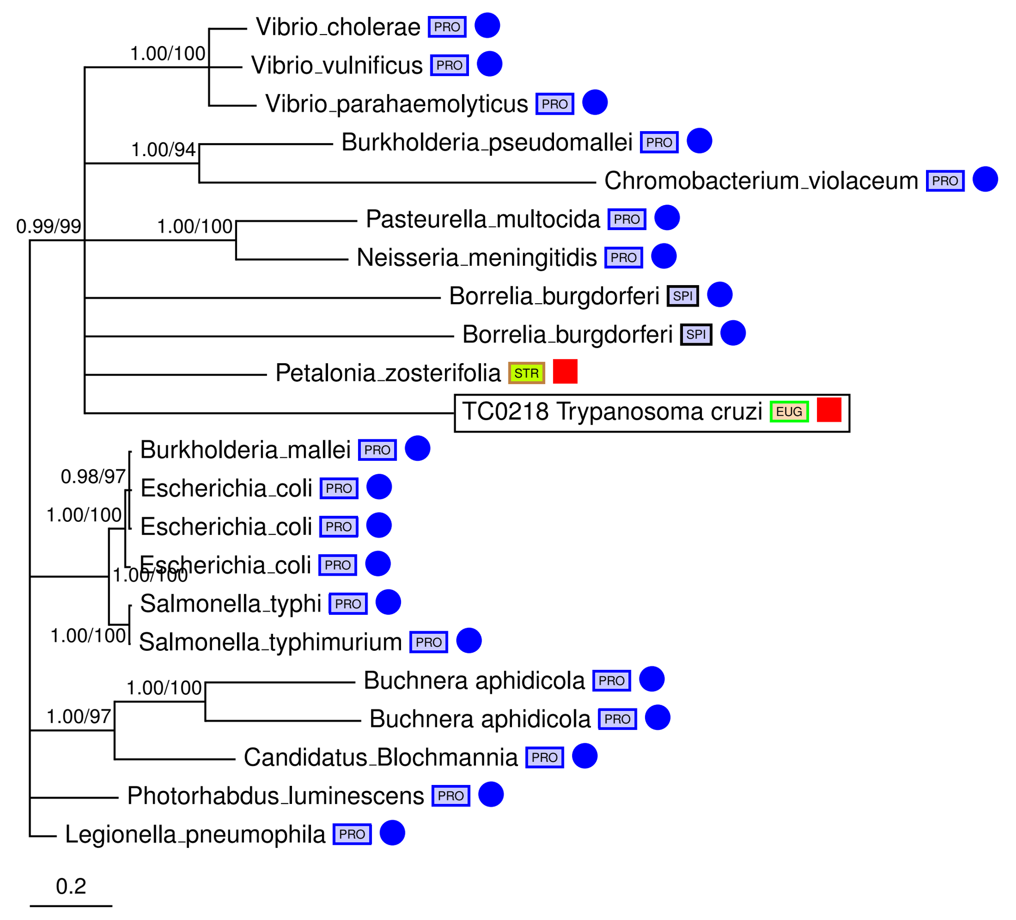

EE008

Candy accession:	Q50LZ9_ENTHI
RefSeq accession:	XP_648004.1
Uniprot accession:	C4M8F0_ENTHI
Comments:	LGT? - POLYTOMY OF ANIMAL HOST ASSOCIATED ANAEROBIC PROTISTS - TV AND EH
Species affected:	EH,TV
Adjacent taxa in tree:	Firmicutes
EC annotation - (Blast/Profile):	na
PHOBIUS SP:	0
PHOBIUS TMD:	0
RefSeq annotation:	hypothetical protein
Name of enzyme/protein:	Protein containing nitroreductase domain
KEGG PATHWAY - level 1:	Other function
KEGG PATHWAY - level 2:	na

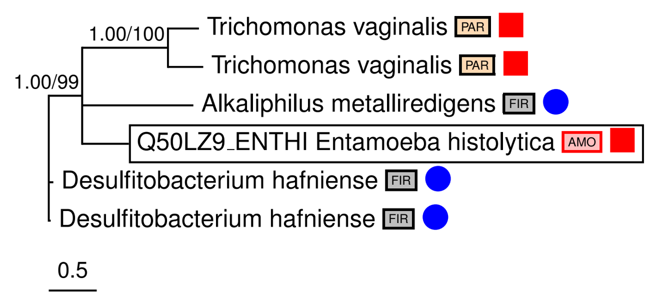

EE009

```
                Candy accession: Q9GQI5_GIAIN
               RefSeq accession: XP_001704452.1
              Uniprot accession: Q9GQI5_GIAIN
                       Comments: LGT - LIKELY LGT BETWEEN DIPLOMONAD AND
                                 AMOEBOZOA
               Species affected: GI
          Adjacent taxa in tree: Entamoeba
EC annotation - (Blast/Profile): EC:6.1.1.7
                     PHOBIUS SP: 0
                    PHOBIUS TMD: 0
              RefSeq annotation: Alanyl-tRNA synthetase
         Name of enzyme/protein: alanine-tRNA ligase
         KEGG PATHWAY - level 1: Translation - Genetic Information
                                 Processing
         KEGG PATHWAY - level 2: Aminoacyl-tRNA biosynthesis

                Candy accession: Q519U1_ENTHI
               RefSeq accession: XP_654998.1
              Uniprot accession: Q519U1_ENTHI
                       Comments: LGT - LIKELY LGT BETWEEN DIPLOMONAD AND
                                 AMOEBOZOA
               Species affected: EH
          Adjacent taxa in tree: Giardia
EC annotation - (Blast/Profile): EC:6.1.1.7
                     PHOBIUS SP: 0
                    PHOBIUS TMD: 0
              RefSeq annotation: alanyl-tRNA synthetase
         Name of enzyme/protein: alanine-tRNA ligase
         KEGG PATHWAY - level 1: Translation - Genetic Information
                                 Processing
         KEGG PATHWAY - level 2: Translation - Genetic Information
                                 Processing
```

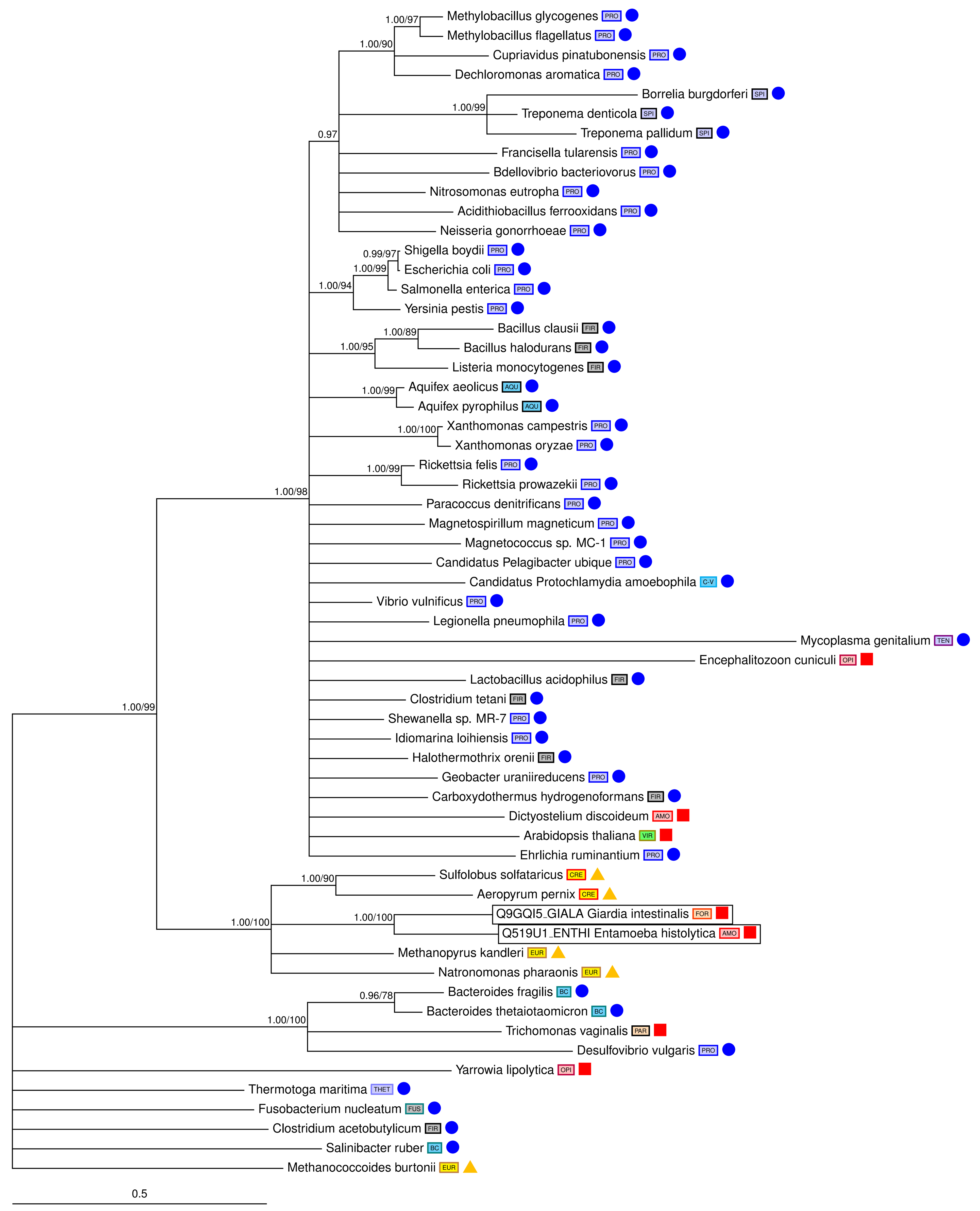

EE010

```
                Candy accession: TV93446258
              RefSeq accession: XP_001327107.1
             Uniprot accession: A2DXD2_TRIVA
                      Comments: LGT?- POLYTOMY OF  ANIMAL HOST
                                ASSOCIATED ANAEROBIC PROTISTS - TV AND
                                EH
              Species affected: EH,TV
         Adjacent taxa in tree: Polytomy
EC annotation - (Blast/Profile): EC:3.4.11.4
                    PHOBIUS SP: 0
                   PHOBIUS TMD: 0
             RefSeq annotation: Clan MH, family M20, peptidase T-like
                                metallopeptidase
        Name of enzyme/protein: tripeptide aminopeptidase
        KEGG PATHWAY - level 1: Reaction
        KEGG PATHWAY - level 2: Reaction

               Candy accession: C4M2F1_ENTHI
              RefSeq accession: XP_650152
             Uniprot accession: C4M2F1_ENTHI
                      Comments: LGT?- POLYTOMY OF  ANIMAL HOST
                                ASSOCIATED ANAEROBIC PROTISTS - TV AND
                                EH
              Species affected: EH,TV
         Adjacent taxa in tree: Polytomy
EC annotation - (Blast/Profile): EC:3.4.11.14
             RefSeq annotation: peptidase T
        Name of enzyme/protein: peptidase T
        KEGG PATHWAY - level 1: Reaction
        KEGG PATHWAY - level 2: Reaction
```

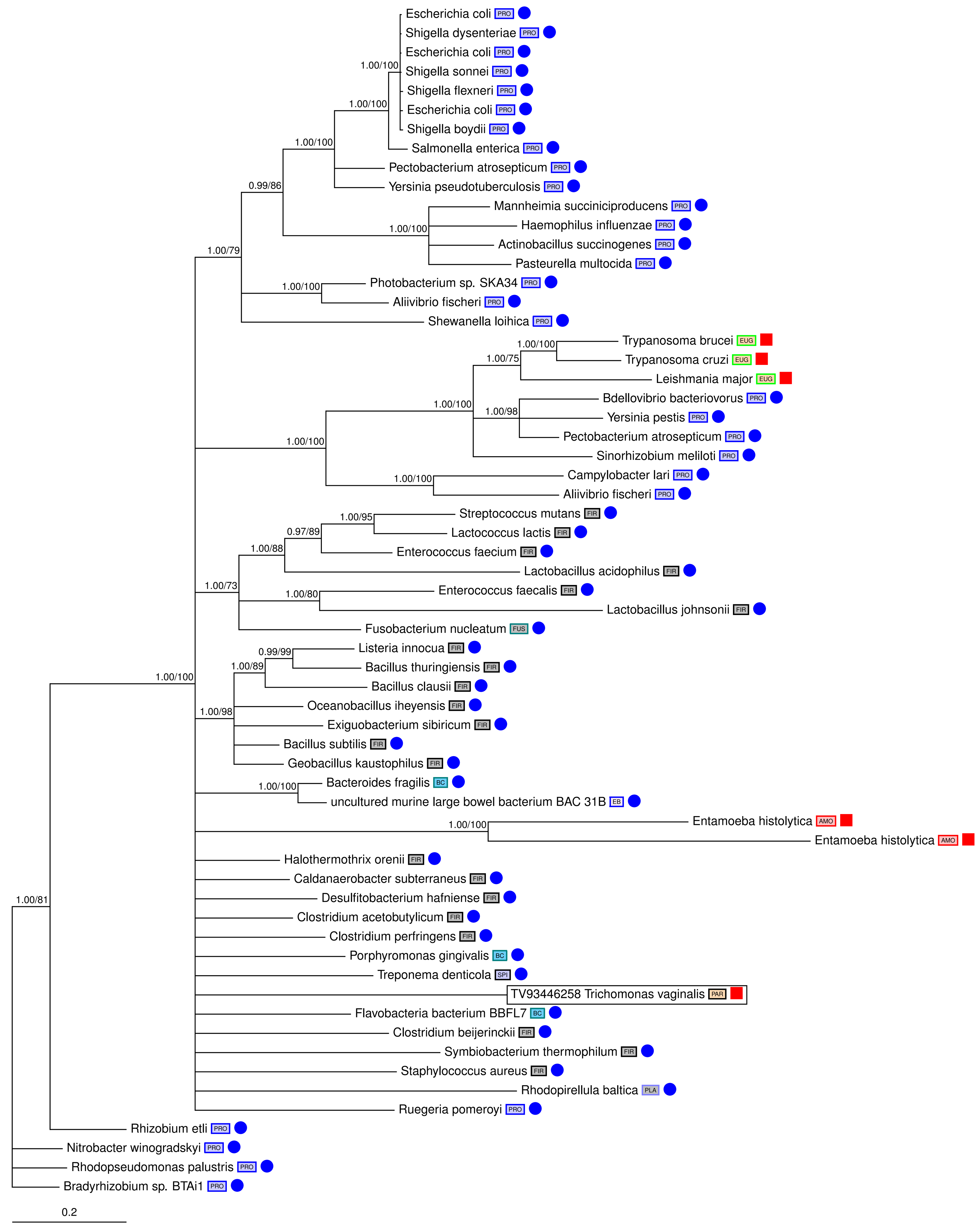

EE011

```
            Candy accession: Q7QXP8_GIALA
           RefSeq accession: XP_001709915.1
          Uniprot accession: Q7QXP8_GIALA
                   Comments: LGT - POSSIBLE LGT BETWEEN DIPLOMONAD
                             AND AMOEBOZOA
           Species affected: GL,EH
      Adjacent taxa in tree: Entamoeba
EC annotation - (Blast/Profile): EC:1.12.7.2
                PHOBIUS SP: 0
               PHOBIUS TMD: 0
          RefSeq annotation: Fe-hydrogenase-1
   Name of enzyme/protein: Iron hydrogenase
     KEGG PATHWAY - level 1: Energy Metabolism
     KEGG PATHWAY - level 2: Methane metabolism

            Candy accession: Q51EJ9_ENTHI
           RefSeq accession: XP_656685.1
          Uniprot accession: Q51EJ9_ENTHI
                   Comments: LGT - POSSIBLE LGT BETWEEN DIPLOMONAD
                             AND AMOEBOZOA
           Species affected: GL,EH
      Adjacent taxa in tree: Giardia
EC annotation - (Blast/Profile): EC:1.12.7.2
                PHOBIUS SP: 0
               PHOBIUS TMD: 0
          RefSeq annotation: Fe-hydrogenase
   Name of enzyme/protein: Iron hydrogenase
     KEGG PATHWAY - level 1: Energy Metabolism
     KEGG PATHWAY - level 2: Methane metabolism
```

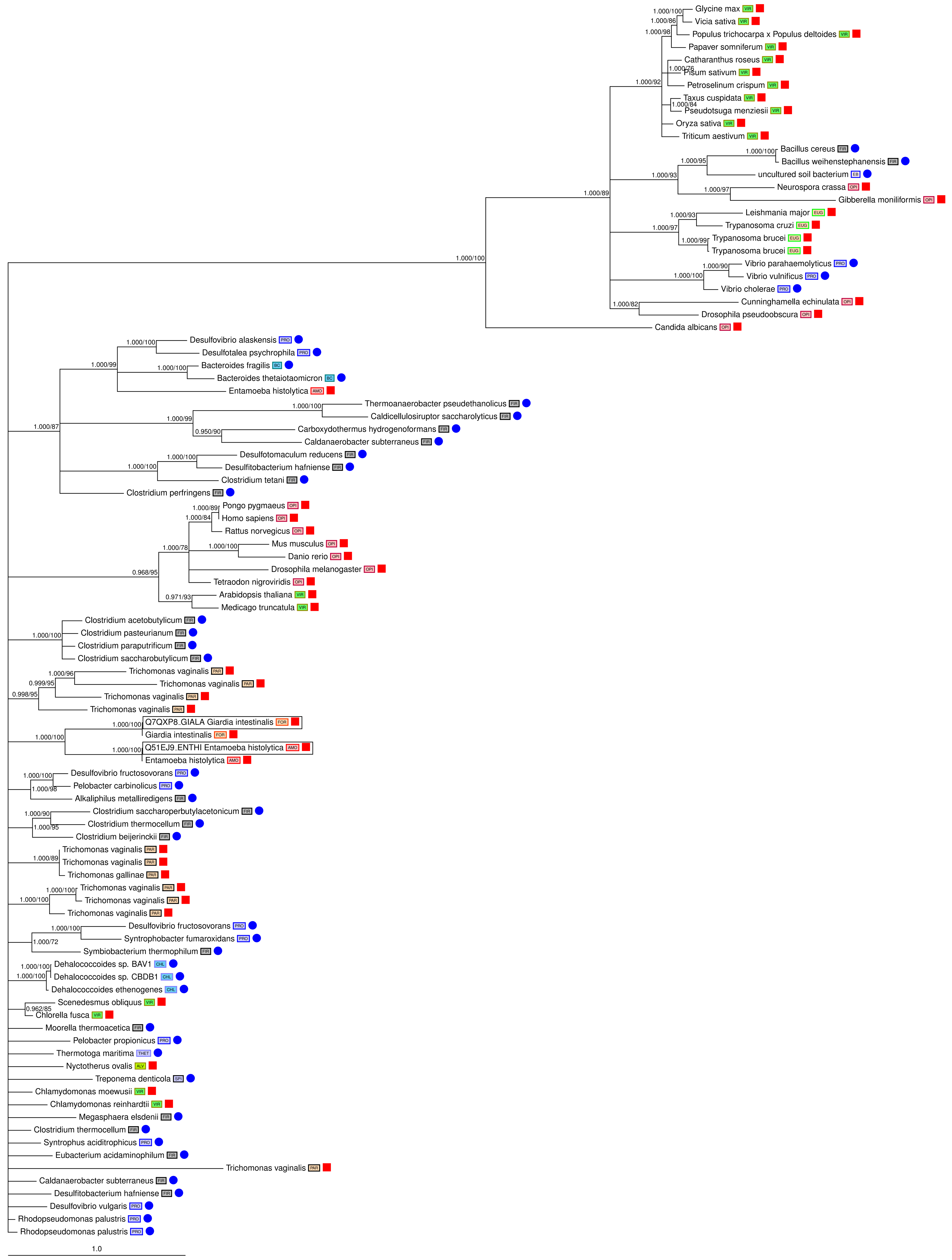

EE012

```
              Candy accession: TV86485458
             RefSeq accession: XP_001584037.1
            Uniprot accession: A2D925_TRIVA
                     Comments: LGT? - POLYTOMY OF  ANIMAL HOST
                               ASSOCIATED PROTISTS - TV AND
                               KINETOPLASTIDS
             Species affected: TV,LM,TC
         Adjacent taxa in tree: Bacterial
EC annotation - (Blast/Profile): na
                   PHOBIUS SP: 0
                  PHOBIUS TMD: 0
            RefSeq annotation: YbaK / prolyl-tRNA synthetases
                               associated domain containing protein
       Name of enzyme/protein: Predicted cysteinyl-tRNA(Pro) deacylases
        KEGG PATHWAY - level 1: Other function
        KEGG PATHWAY - level 2: na
```

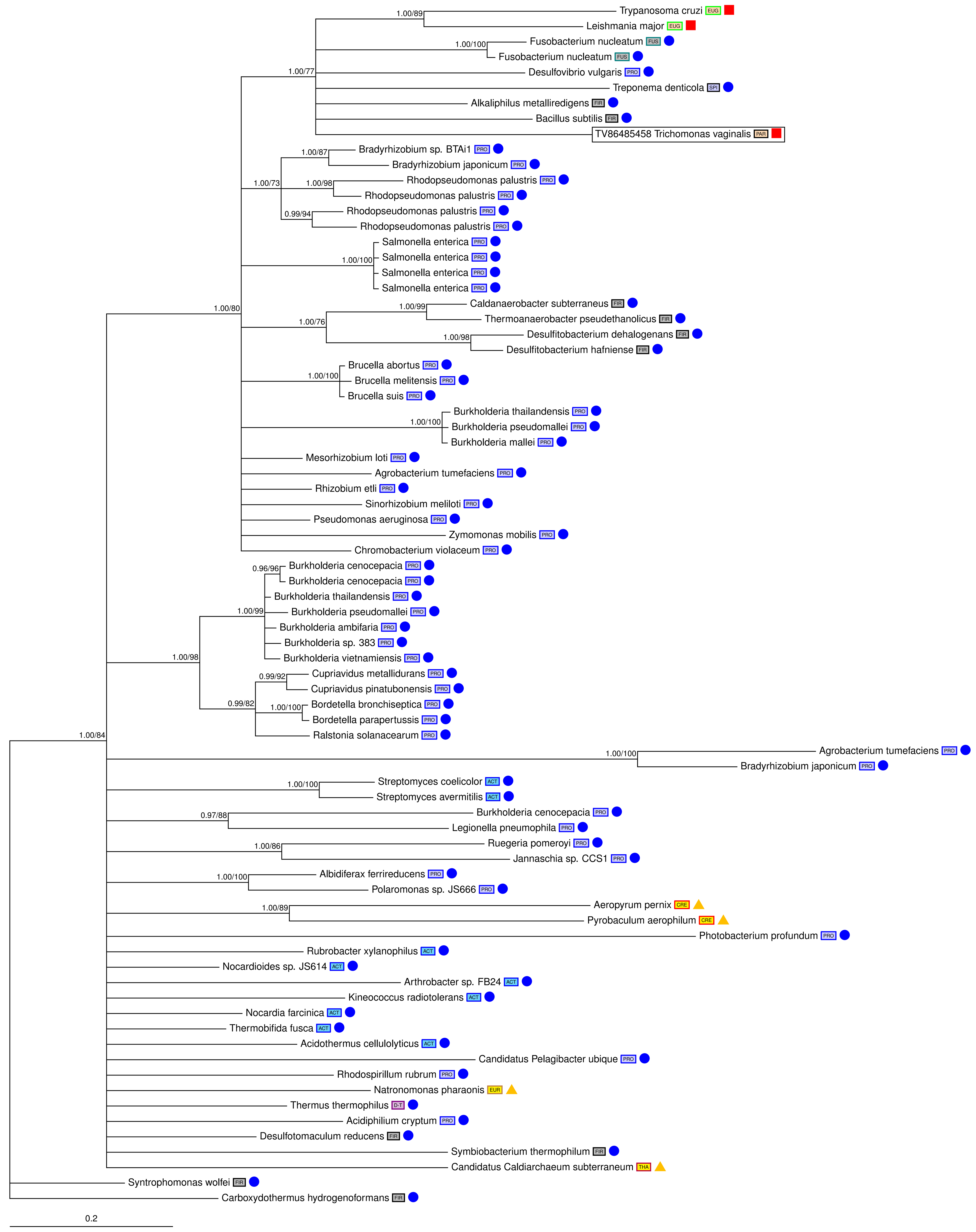

EE013

```
                Candy accession: TV86693111
               RefSeq accession: XP_001316689.1
              Uniprot accession: A2ESA2_TRIVA
                       Comments: LGT? - POLYTOMY OF  ANIMAL HOST
                                 ASSOCIATED ANAEROBIC MICROBIAL
                                 EUKARYOTES - RUMEN CILIATES, RUMEN FUNGI
                                 AND TV
               Species affected: TV
          Adjacent taxa in tree: Firmicutes
EC annotation - (Blast/Profile): EC:3.2.1.4
                     PHOBIUS SP: 0
                    PHOBIUS TMD: 0
              RefSeq annotation: hypothetical protein
         Name of enzyme/protein: cellulase
         KEGG PATHWAY - level 1: Carbohydrate Metabolism
         KEGG PATHWAY - level 2: Starch and sucrose metabolism
```

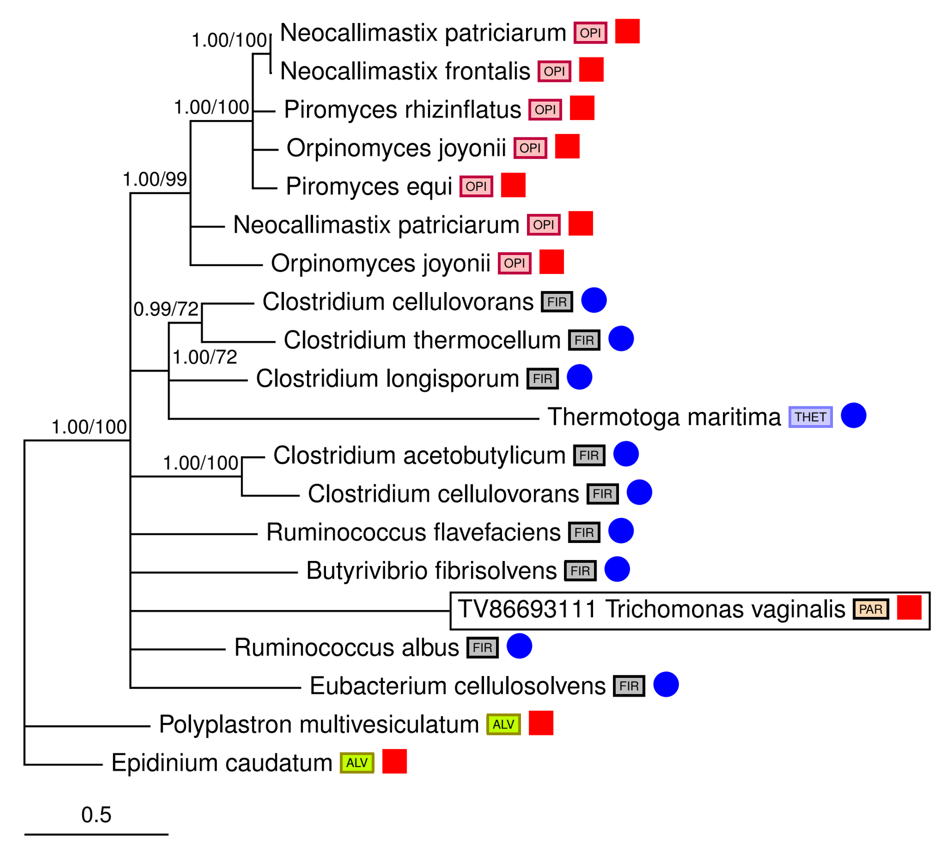

EE014

```
           Candy accession: TV96829043
          RefSeq accession: XP_001307133.1
         Uniprot accession: A2FLN0_TRIVA
                  Comments: LGT? - POLYTOMY  OF ANIMAL HOST
                            ASSOCIATED PROTISTS TV AND LM WITH
                            BACTERIA IN SAME CLAN
          Species affected: n
      Adjacent taxa in tree: Bacteroidetes
EC annotation - (Blast/Profile): EC:1.3.1.9
                PHOBIUS SP: 0
               PHOBIUS TMD: 0
         RefSeq annotation: oxidoreductase, 2-nitropropane
                            dioxygenase family protein
    Name of enzyme/protein: enoyl-[acyl-carrier-protein] reductase
                            (NADH)
    KEGG PATHWAY - level 1: Lipid Metabolism
    KEGG PATHWAY - level 2: Fatty acid biosynthesis
```

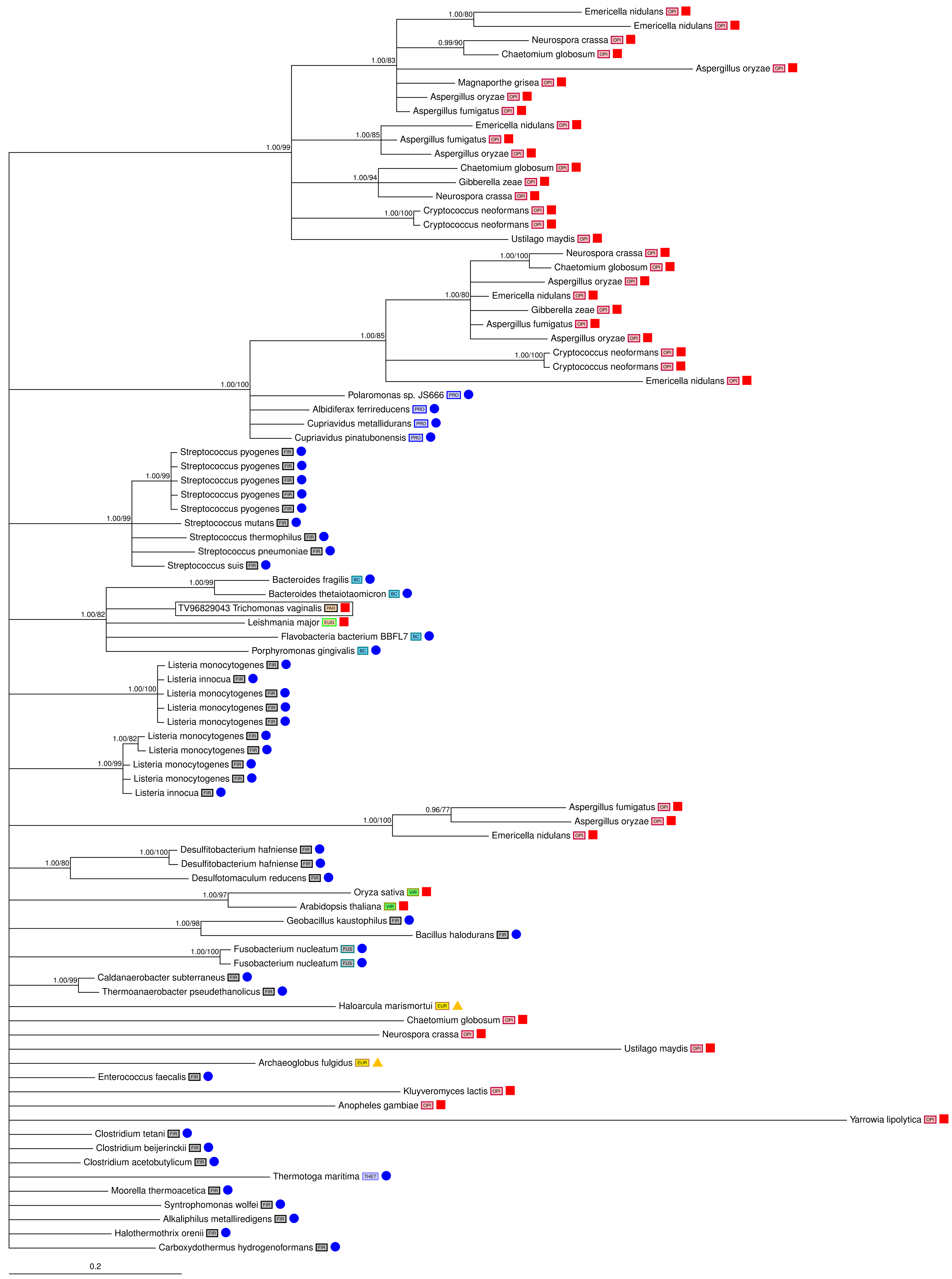

EE015

Candy accession: Q518Q4_ENTHI
RefSeq accession: XP_654698.1
Uniprot accession: C4LZV4_ENTHI
Comments: LGT? - POLYTOMY OF ANIMAL HOST
ASSOCIATED PROTISTS - LM,TC,TB AND EH
Species affected: EH, LM,TB,TC
Adjacent taxa in tree: Polytomy
EC annotation - (Blast/Profile): EC:5.3.3.2
PHOBIUS SP: 0
PHOBIUS TMD: 0
RefSeq annotation: isopentenyl-diphosphate delta-isomerase
Name of enzyme/protein: isopentenyl-diphosphate Delta-isomerase
KEGG PATHWAY - level 1: Metabolism of Terpenoids and Polyketides
KEGG PATHWAY - level 2: Terpenoid backbone biosynthesis

Candy accession: Q5QQ43_LEIMA
RefSeq accession: XP_843634.1
Uniprot accession: Q5QQ43_LEIMA
Comments: LGT? - POLYTOMY OF ANIMAL HOST
ASSOCIATED PROTISTS - LM,TC,TB AND EH
Species affected: EH, LM,TB,TC
Adjacent taxa in tree: Polytomy
EC annotation - (Blast/Profile): EC:5.3.3.2
PHOBIUS SP: 0
PHOBIUS TMD: 0
RefSeq annotation: isomerase
Name of enzyme/protein: isopentenyl-diphosphate Delta-isomerase
KEGG PATHWAY - level 1: Metabolism of Terpenoids and Polyketides
KEGG PATHWAY - level 2: Terpenoid backbone biosynthesis

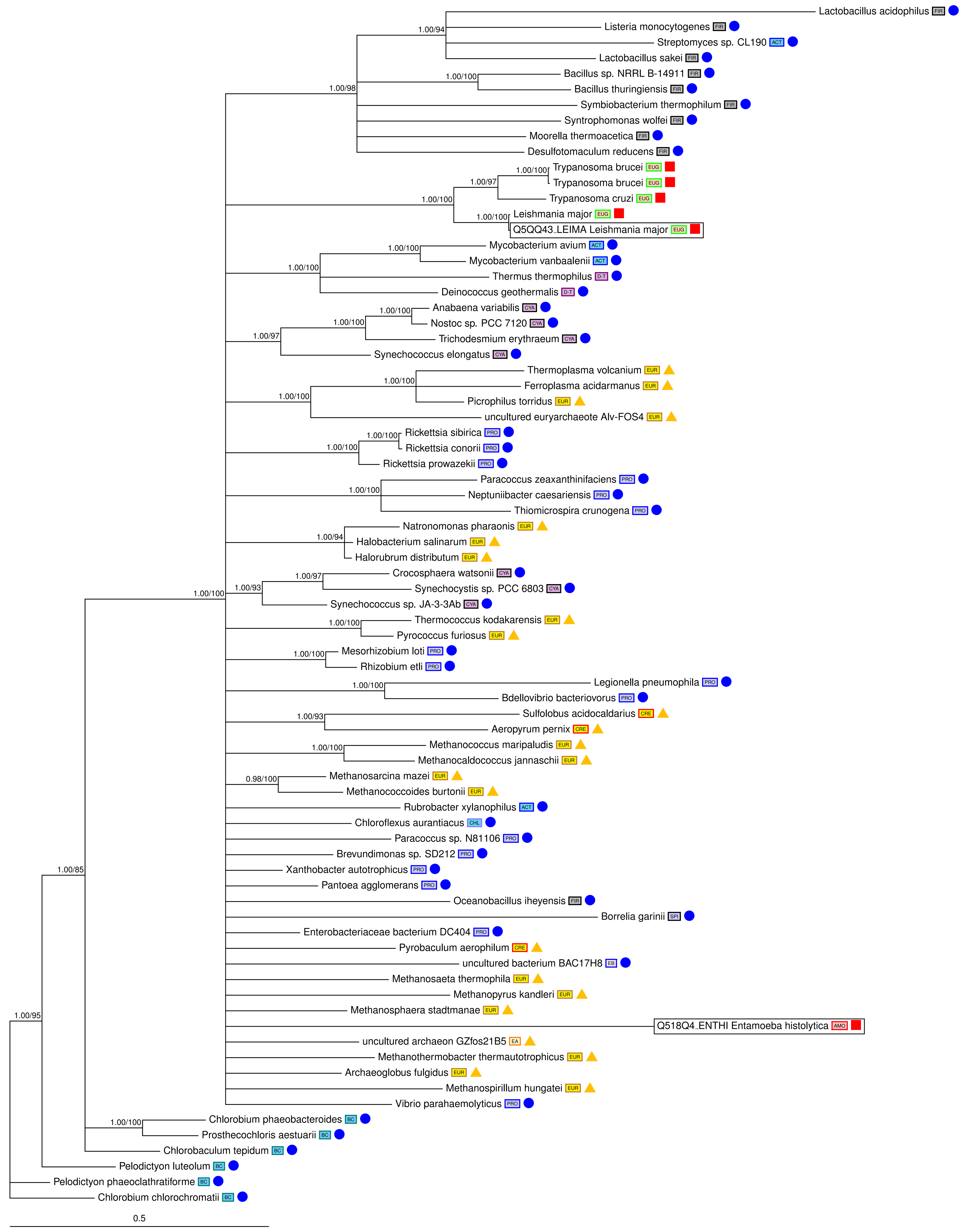

EE016

```
                Candy accession: TV81616199
               RefSeq accession: XP_001326960.1
              Uniprot accession: A2DY01_TRIVA
                       Comments: LGT? - POLYTOMY OF  ANIMAL HOST
                                 ASSOCIATED ANAEROBIC PROTISTS - TV AND
                                 EH ONE NODE
               Species affected: EH, TV
           Adjacent taxa in tree: Bacterial
EC annotation - (Blast/Profile): EC:4.1.2.13
                     PHOBIUS SP: 0
                    PHOBIUS TMD: 0
              RefSeq annotation: fructose-1,6-bisphosphate aldolase
         Name of enzyme/protein: fructose-bisphosphate aldolase
         KEGG PATHWAY - level 1: Carbohydrate Metabolism
         KEGG PATHWAY - level 2: Glycolysis / Gluconeogenesis, Pentose
                                 phosphate pathway,  Fructose and mannose
                                 metabolism

                Candy accession: Q50TT0_ENTHI
               RefSeq accession: XP_650373.1
              Uniprot accession: C4LXD7_ENTHI
                       Comments: LGT? - POLYTOMY OF  ANIMAL HOST
                                 ASSOCIATED ANAEROBIC PROTISTS - TV AND
                                 EH ONE NODE
               Species affected: EH, TV
           Adjacent taxa in tree: Bacterial
EC annotation - (Blast/Profile): EC:4.1.2.13
                     PHOBIUS SP: 0
                    PHOBIUS TMD: 0
              RefSeq annotation: fructose-1,6-bisphosphate aldolase
         Name of enzyme/protein: fructose-bisphosphate aldolase
         KEGG PATHWAY - level 1: Carbohydrate Metabolism
         KEGG PATHWAY - level 2: Glycolysis / Gluconeogenesis / Fructose
                                 and mannose metabolism
```

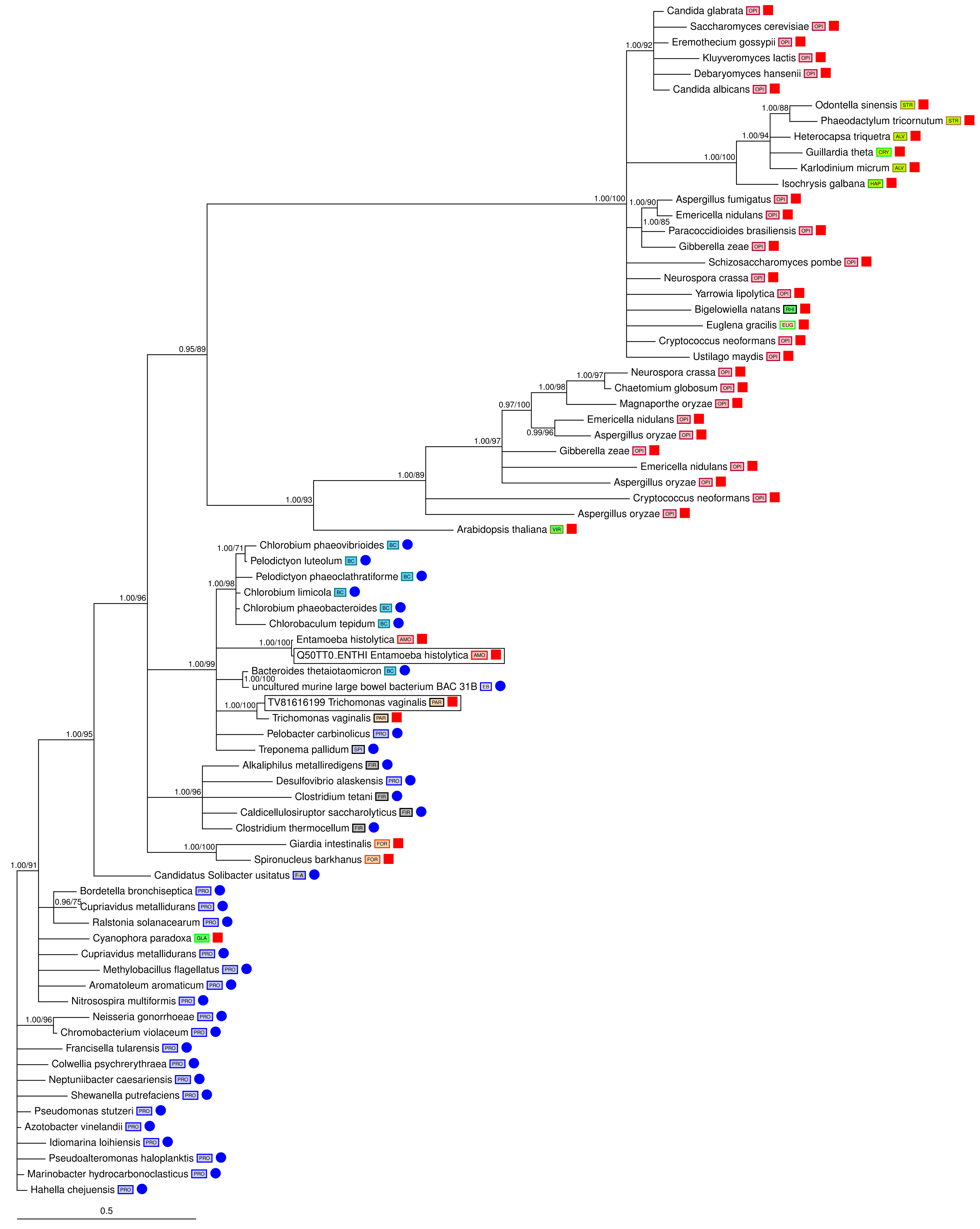

EE017

```
               Candy accession: Q4QES5_LEIMA
              RefSeq accession: XP_001682173.1
             Uniprot accession: Q4QES5_LEIMA
                      Comments: LGT? - POLYTOMY OF  ANIMAL HOST
                                ASSOCIATED PARASITC PROTISTS -  LM+CP+CH
              Species affected: LM, CP
         Adjacent taxa in tree: Polytomy
EC annotation - (Blast/Profile): EC:2.4.1.14
                    PHOBIUS SP: 0
                   PHOBIUS TMD: 0
             RefSeq annotation: sucrose-phosphate synthase-like protein
      Name of enzyme/protein: sucrose-phosphate synthase
        KEGG PATHWAY - level 1: Carbohydrate Metabolism
        KEGG PATHWAY - level 2: Starch and sucrose metabolism
```

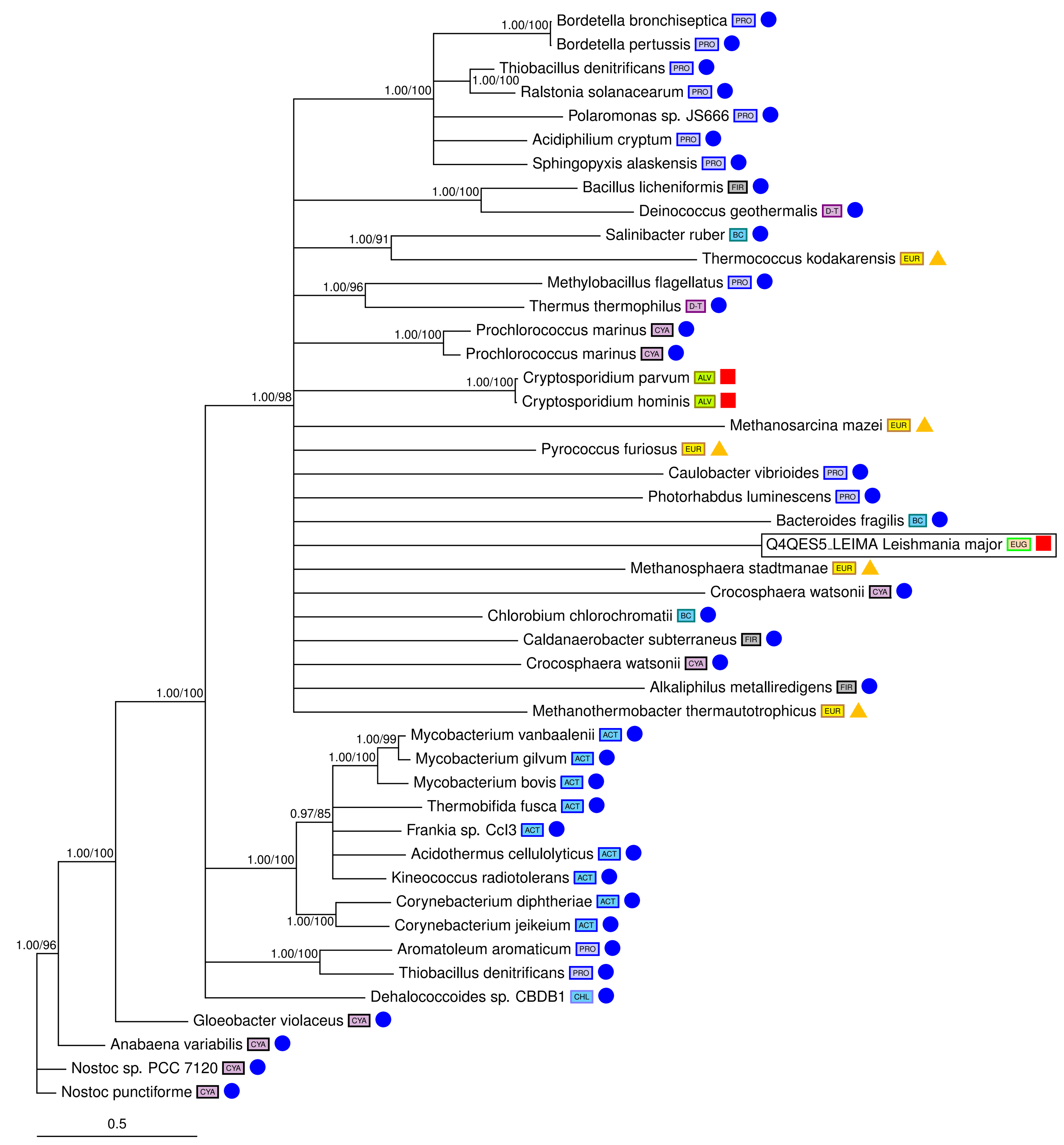

EE018

Candy accession: TV82184103
RefSeq accession: XP_001311676.1
Uniprot accession: A2F8L4_TRIVA
Comments: LGT? - POLYTOMY OF ANIMAL HOST ASSOCIATED ANAEROBIC PROTISTS - TV, EH, GI
Species affected: EH,GI,TV
Adjacent taxa in tree: Polytomy
EC annotation - (Blast/Profile): EC:2.7.1.48
PHOBIUS SP: 0
PHOBIUS TMD: 0
RefSeq annotation: Phosphoribulokinase / Uridine kinase family protein
Name of enzyme/protein: uridine kinase
KEGG PATHWAY - level 1: Nucleotide Metabolism
KEGG PATHWAY - level 2: Pyrimidine metabolism

Candy accession: Q7QP06_GIALA
RefSeq accession: XP_001704181.1
Uniprot accession: A8BYJ2_GIALA
Comments: LGT? - POLYTOMY OF ANIMAL HOST ASSOCIATED ANAEROBIC PROTISTS - TV, EH, GI
Species affected: EH,GI,TV
Adjacent taxa in tree: Polytomy
EC annotation - (Blast/Profile): EC:2.7.1.48
PHOBIUS SP: 0
PHOBIUS TMD: 0
RefSeq annotation: Uridine kinase
Name of enzyme/protein: uridine kinase
KEGG PATHWAY - level 1: Nucleotide Metabolism
KEGG PATHWAY - level 2: Pyrimidine metabolism

Candy accession: Q50SP1_ENTHI
RefSeq accession: XP_656795.1
Uniprot accession: C4LYS1_ENTHI
Comments: LGT? - POLYTOMY OF ANIMAL HOST ASSOCIATED ANAEROBIC PROTISTS - TV, EH, GI
Species affected: EH,GI,TV
Adjacent taxa in tree: Polytomy
EC annotation - (Blast/Profile): EC:2.7.1.48
PHOBIUS SP: 0
PHOBIUS TMD: 0
RefSeq annotation: phosphoribulokinase /uridine kinase family protein
Name of enzyme/protein: uridine kinase
KEGG PATHWAY - level 1: Nucleotide Metabolism
KEGG PATHWAY - level 2: Pyrimidine metabolism

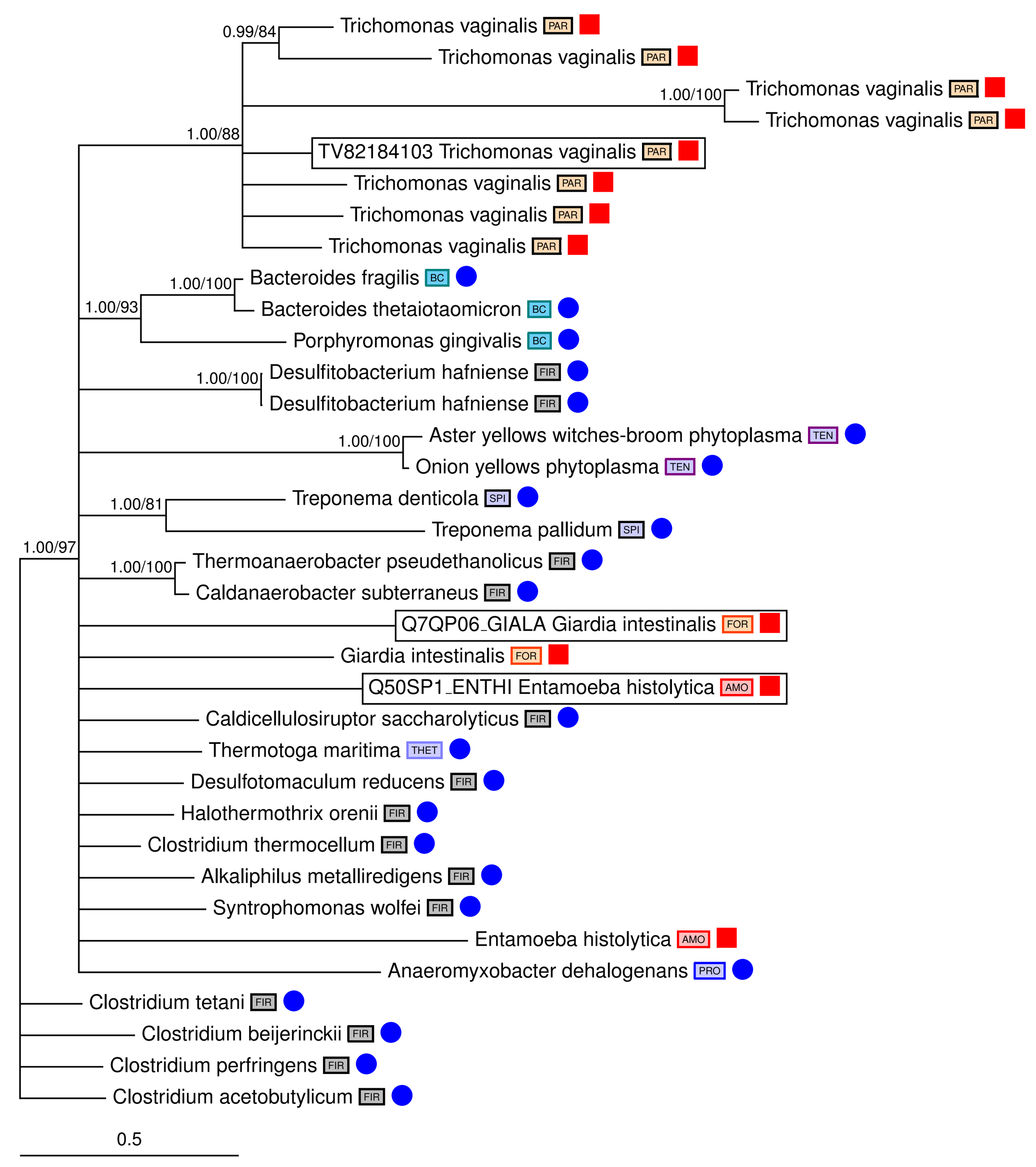

EE019

```
               Candy accession: TV82365005
              RefSeq accession: XP_001295233.1
             Uniprot accession: A2GKL5_TRIVA
                      Comments: LGT? - POLYTOMY OF  ANIMAL HOST
                                ASSOCIATED ANAEROBIC PROTISTS - TV, EH
              Species affected: PF,TV,EH
         Adjacent taxa in tree: Prokaryote
EC annotation - (Blast/Profile): na
                    PHOBIUS SP: 0
                   PHOBIUS TMD: 0
             RefSeq annotation: choline binding protein
        Name of enzyme/protein: Protein containing LRR domains
        KEGG PATHWAY - level 1: Function unknown
        KEGG PATHWAY - level 2: na
```

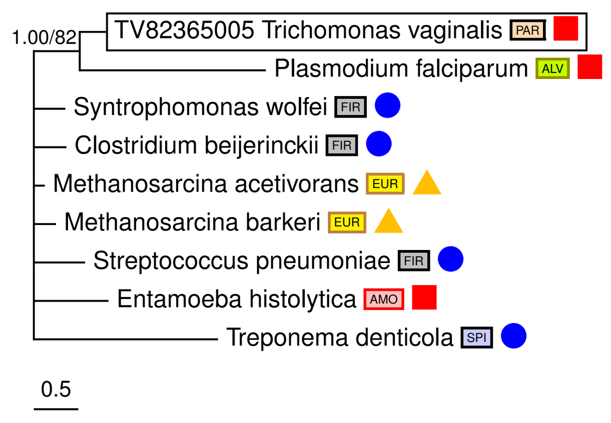

EE020

Candy accession: TV87414345
RefSeq accession: XP_001580194.1
Uniprot accession: A2DK37_TRIVA
Comments: LGT? - POLYTOMY OF ANIMAL HOST ASSOCIATED PROTISTS - TV, LM
Species affected: TV,LM
Adjacent taxa in tree: Prokaryote
EC annotation - (Blast/Profile): na
PHOBIUS SP: 0
PHOBIUS TMD: 0
RefSeq annotation: thiamine biosynthesis protein ThiI
Name of enzyme/protein: Predicted thiamine biosynthesis protein
KEGG PATHWAY - level 1: Other function
KEGG PATHWAY - level 2: na

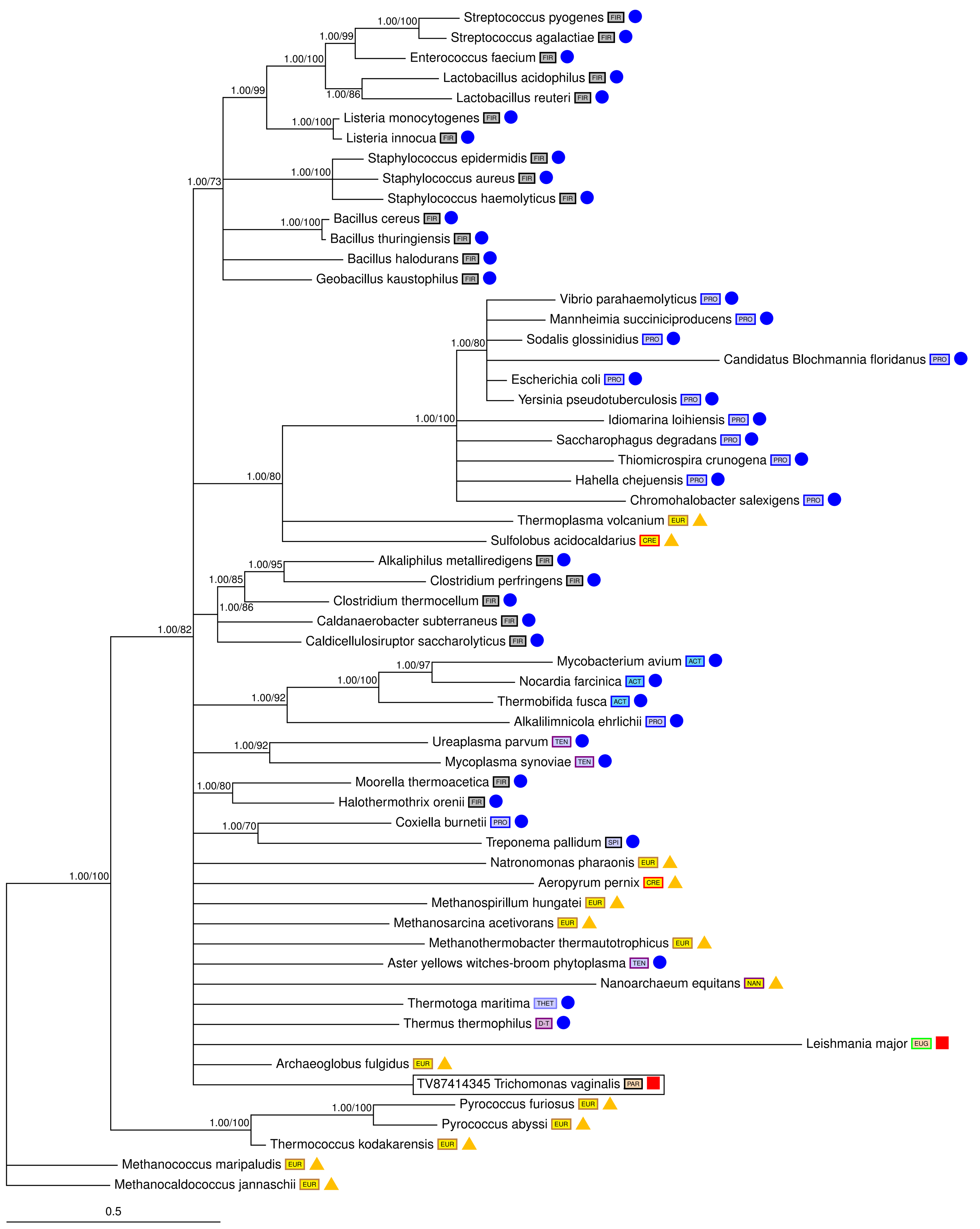

EE021

Candy accession: TV91362078
RefSeq accession: XP_001330242.1
Uniprot accession: A2F143_TRIVA
Comments: LGT? - POLYTOMY OF ANIMAL HOST ASSOCIATED ANAEROBIC PROTISTS - TV, GI
Species affected: TV
Adjacent taxa in tree: GI?
EC annotation - (Blast/Profile): na
PHOBIUS SP: 0
PHOBIUS TMD: 0
RefSeq annotation: metallo-beta-lactamase superfamily protein
Name of enzyme/protein: Predicted Metallo-beta-lactamase
KEGG PATHWAY - level 1: Other function
KEGG PATHWAY - level 2: na

Candy accession: Q7QZA5_GIALA
RefSeq accession: XP_001708073.1
Uniprot accession: A8BBV2_GIAIC
Comments: LGT? - POLYTOMY OF ANIMAL HOST ASSOCIATED ANAEROBIC PROTISTS - TV, GI
Species affected: GI
Adjacent taxa in tree: TV?
EC annotation - (Blast/Profile): EC:3.1.2.6
PHOBIUS SP: 0
PHOBIUS TMD: 0
RefSeq annotation: Metallo-beta-lactamase superfamily protein
Name of enzyme/protein: Predicted Metallo-beta-lactamase
KEGG PATHWAY - level 1: Reaction
KEGG PATHWAY - level 2: Reaction

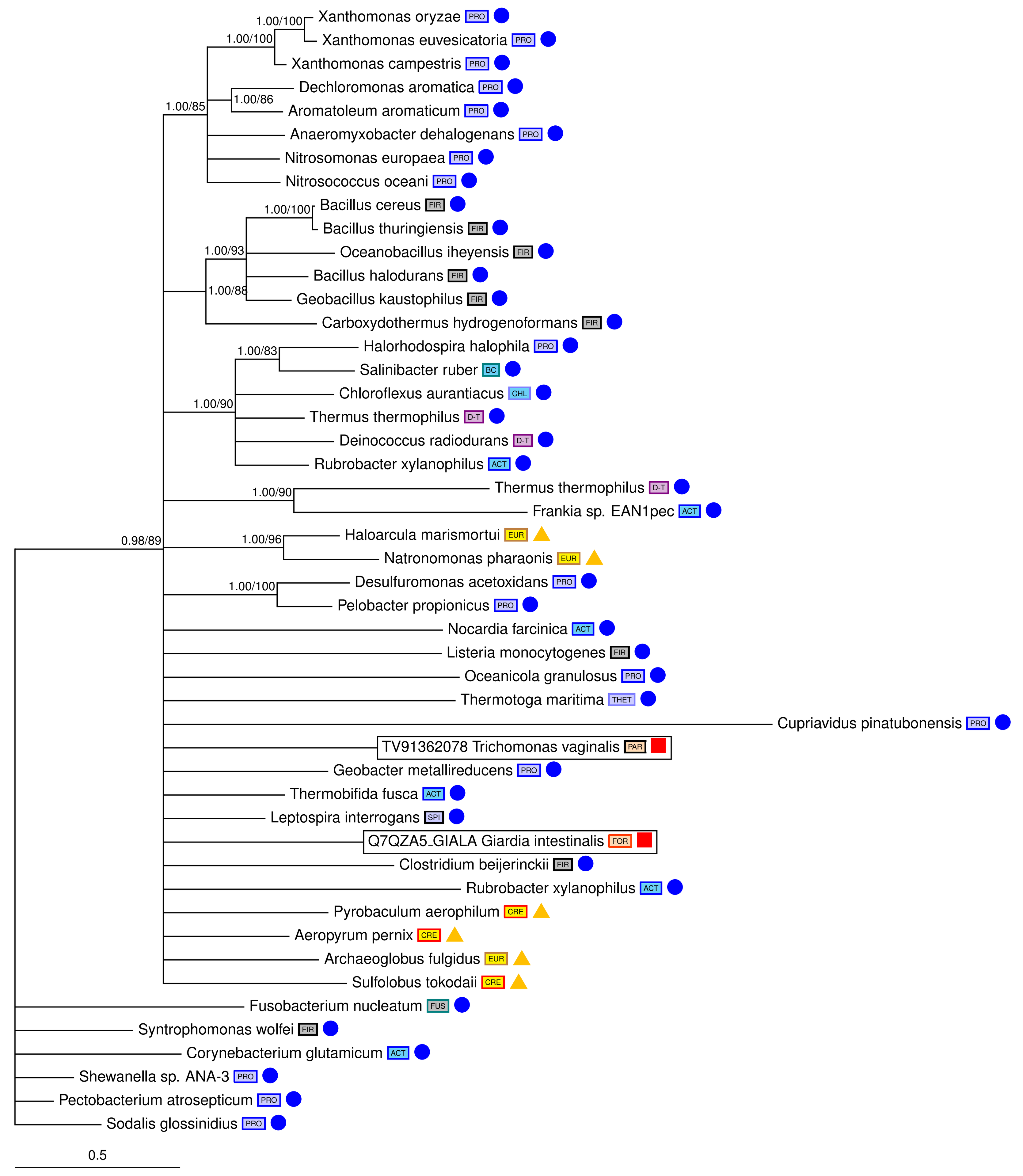

EE022

```
                Candy accession: Q6S4W6_CRYPV
               RefSeq accession: XP_625605.1
              Uniprot accession: Q6S4W6_CRYPV
                       Comments: LGT? - POLYTOMY OF  ANIMAL HOST
                                 ASSOCIATED  INTRACELLULAR PARASITIC
                                 PROTISTS - CP, CH, EC
               Species affected: CP,CH,EC
          Adjacent taxa in tree: Polytomy
EC annotation - (Blast/Profile): EC:2.7.1.21
                     PHOBIUS SP: 0
                    PHOBIUS TMD: 0
              RefSeq annotation: thymidine kinase of likely bacterial
                                 origin
     Name of enzyme/protein: Thymidine kinase
     KEGG PATHWAY - level 1: Nucleotide Metabolism
     KEGG PATHWAY - level 2: Pyrimidine metabolism
```

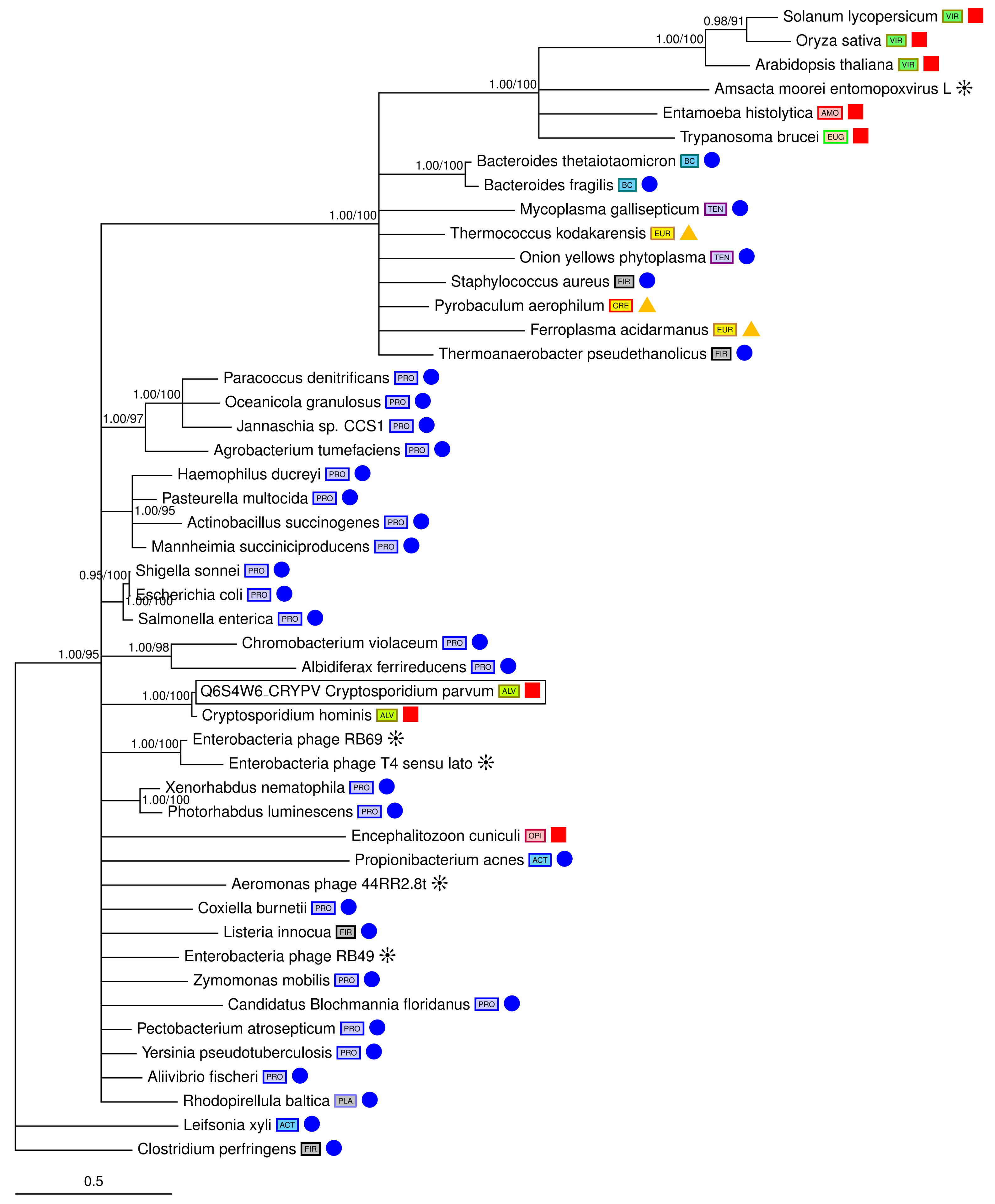

EE023

```
              Candy accession: Q50TU7_ENTHI
             RefSeq accession: XP_650419.1
            Uniprot accession: C4LVY0_ENTHI
                     Comments: LGT? - POLYTOMY OF  ANIMAL HOST
                               ASSOCIATED ANAEROBIC PROTISTS - TV, EH
             Species affected: EH
        Adjacent taxa in tree: Polytomy
EC annotation - (Blast/Profile): EC 1.1.1.2
                  PHOBIUS SP: 0
                 PHOBIUS TMD: 0
            RefSeq annotation: Bacterial
       Name of enzyme/protein: Alcohol dehydrogenase
       KEGG PATHWAY - level 1: Carbohydrate Metabolism, Lipid
                               Metabolism
       KEGG PATHWAY - level 2: Glycolysis / Gluconeogenesis /
                               Glycerolipid metabolism

              Candy accession: TV84258179
             RefSeq accession: XP_001319933.1
            Uniprot accession: A2EHX8_TRIVA
                     Comments: LGT? - POLYTOMY OF  ANIMAL HOST
                               ASSOCIATED ANAEROBIC PROTISTS - TV, EH
             Species affected: TV
        Adjacent taxa in tree: Polytomy
EC annotation - (Blast/Profile): EC 1.1.1.2
                  PHOBIUS SP: 0
                 PHOBIUS TMD: 0
            RefSeq annotation: Bacterial
       Name of enzyme/protein: Alcohol dehydrogenase
       KEGG PATHWAY - level 1: Carbohydrate Metabolism, Lipid
                               Metabolism
       KEGG PATHWAY - level 2: Glycolysis / Gluconeogenesis /
                               Glycerolipid metabolism
```

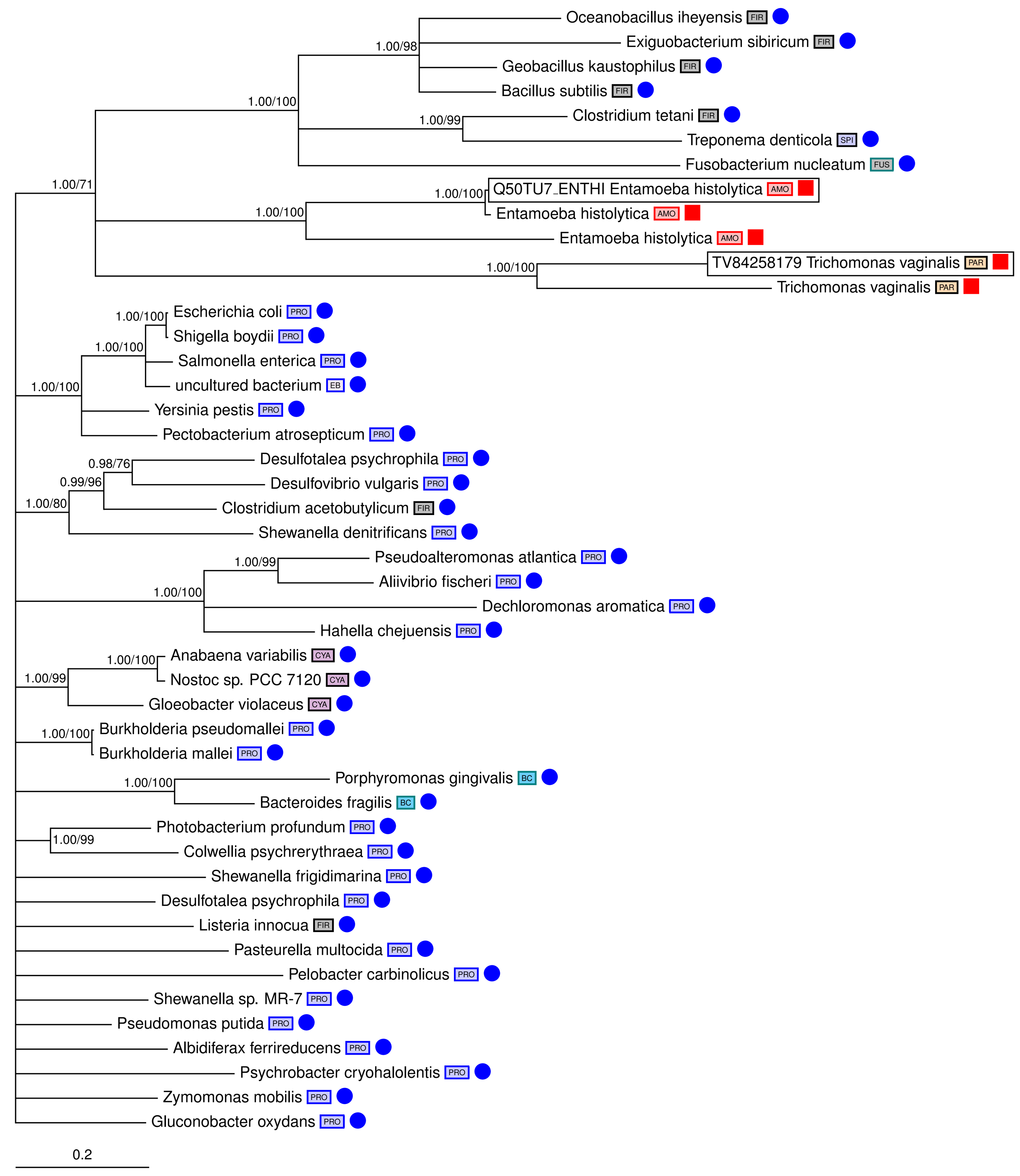

EE024

```
                Candy accession: Q4FWX1_LEIMA
               RefSeq accession: XP_843254
              Uniprot accession: A2FNL2_TRIVA
                       Comments: LGT - FUNGI TO LM
               Species affected: LM, FUNGI
           Adjacent taxa in tree: Fungi
EC annotation - (Blast/Profile): EC:3.5.3.1
                     PHOBIUS SP: 0
                    PHOBIUS TMD: 0
              RefSeq annotation: Arginase family protein
         Name of enzyme/protein: Arginase
         KEGG PATHWAY - level 1: Amino Acid Metabolism
         KEGG PATHWAY - level 2: Arginine and proline metabolism
```

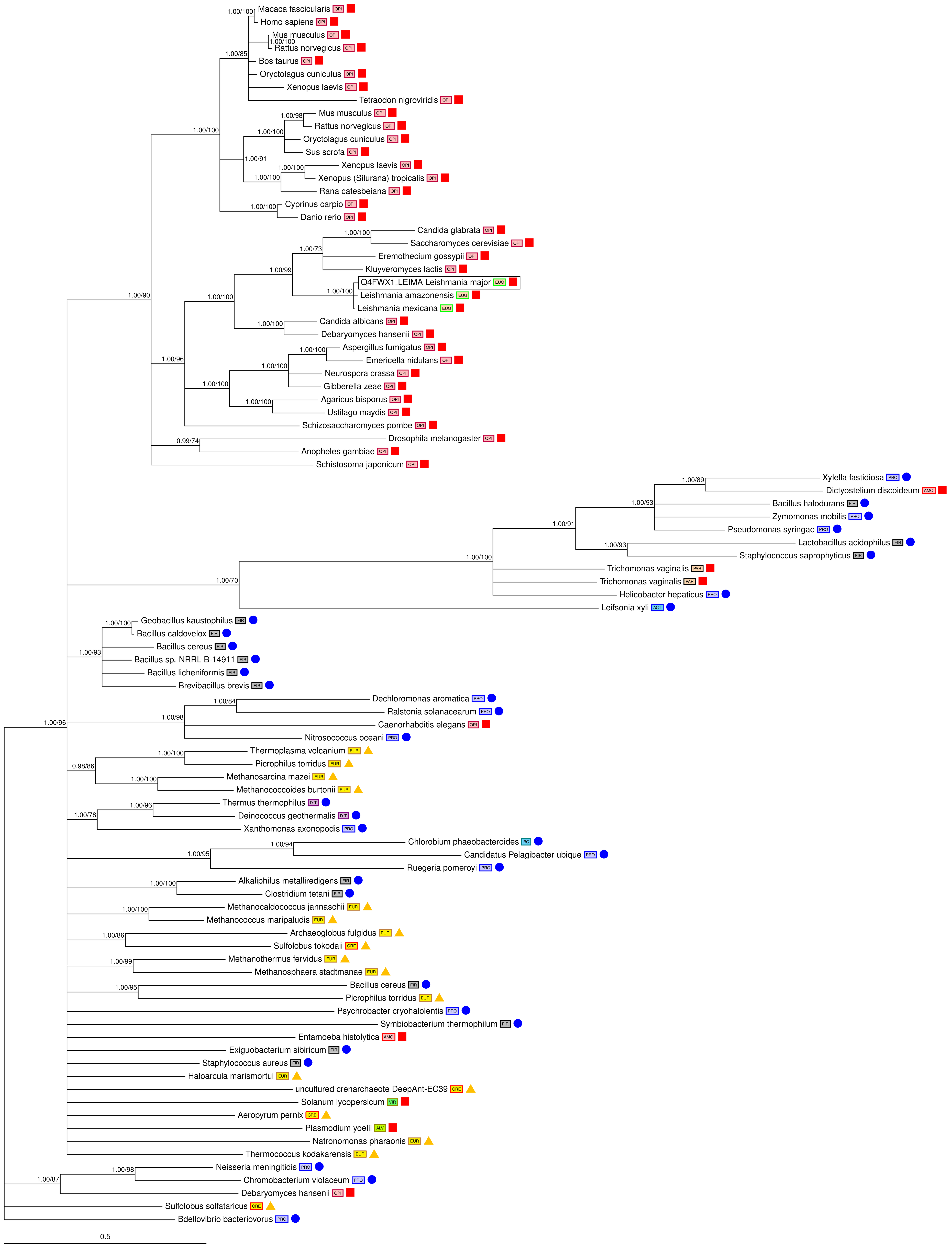

EE025

```
                    Candy accession: Q51C70_ENTHI
                   RefSeq accession: XP_652300.1
                  Uniprot accession: C4M230_ENTHI
                           Comments: LGT? - POLYTOMY EH AND CP
                   Species affected: EH,HT,HM
                Adjacent taxa in tree: Prokaryotes
EC annotation - (Blast/Profile): EC:1.1.1.1
                         PHOBIUS SP: 0
                        PHOBIUS TMD: 0
                  RefSeq annotation: alcohol dehydrogenase
            Name of enzyme/protein: alcohol dehydrogenase
             KEGG PATHWAY - level 1: Carbohydrate Metabolism, Lipid
                                     Metabolism, Amino Acid Metabolism
             KEGG PATHWAY - level 2: Glycolysis / Gluconeogenesis, Fatty acid
                                     metabolism, Glycine, serine and
                                     threonine metabolism
```

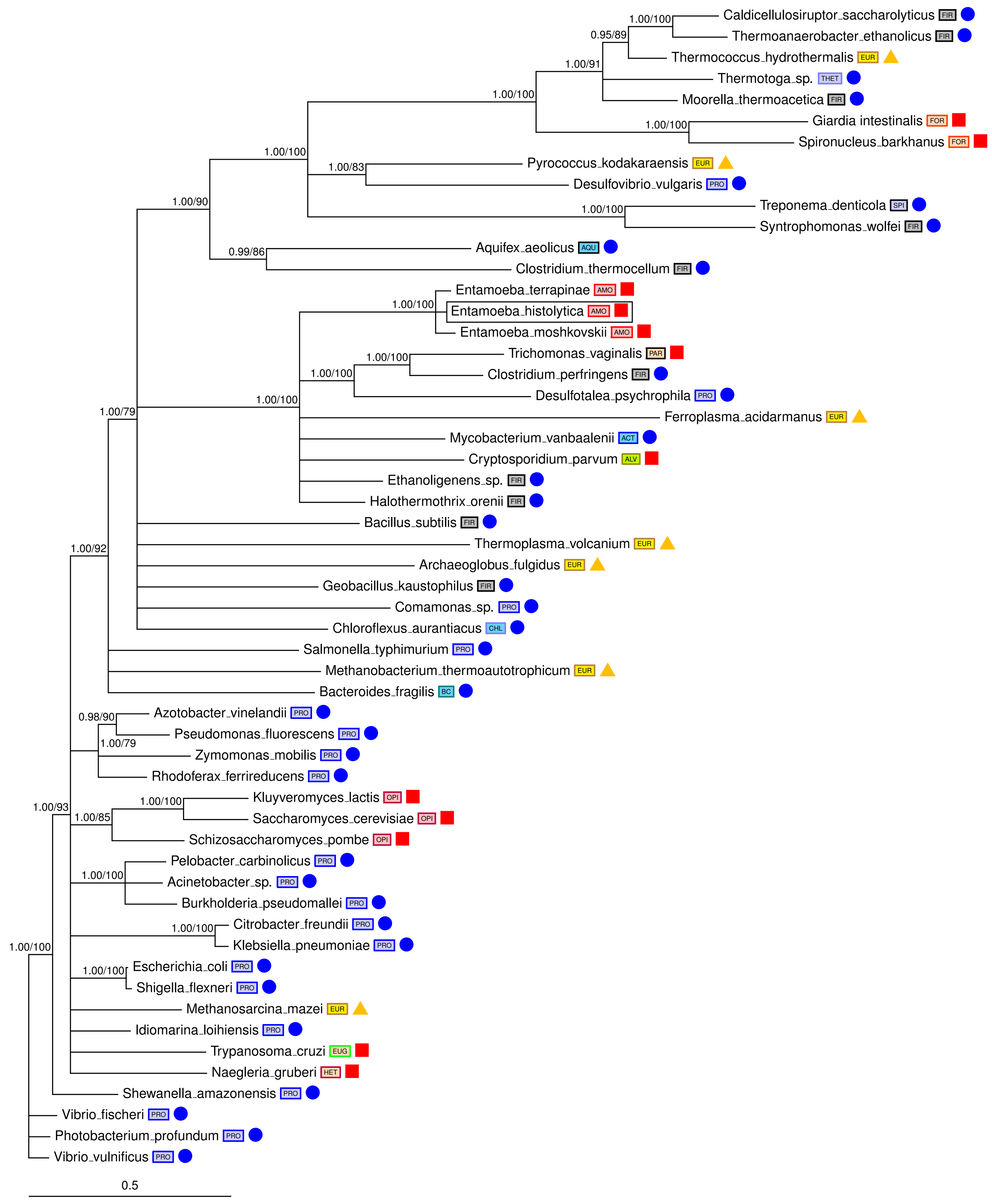

EE026

```
                Candy accession: B1N387_ENTHI
               RefSeq accession: XP_001913653
              Uniprot accession: B1N387_ENTHI
                       Comments: LGT -  DEEP LGT INTO AMOEBOZOA -> CP AND
                                 FUNGI, ALL HOST ASSOCIATED
               Species affected: Amoebozoa, CP, PIROMYCES
          Adjacent taxa in tree: Prokaryotes
EC annotation - (Blast/Profile): EC:1.1.1.1
                     PHOBIUS SP: 0
                    PHOBIUS TMD: 0
              RefSeq annotation: alcohol dehydrogenase
         Name of enzyme/protein: alcohol dehydrogenase
         KEGG PATHWAY - level 1: Carbohydrate Metabolism, Lipid
                                 Metabolism, Amino Acid Metabolism
         KEGG PATHWAY - level 2: Glycolysis / Gluconeogenesis, Fatty acid
                                 metabolism, Glycine, serine and
                                 threonine metabolism
```

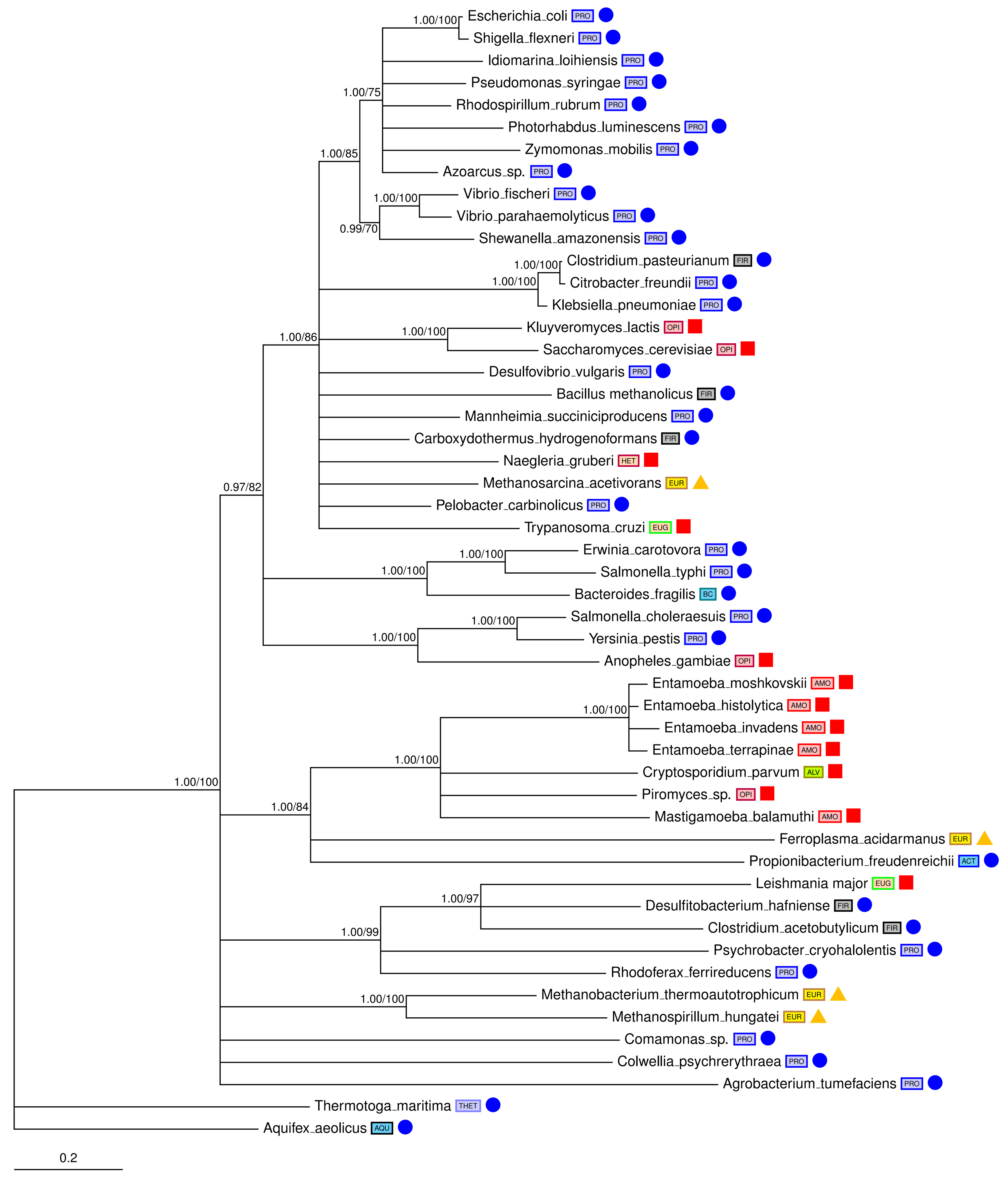

Supplement: Additional file 6 — Phylogenetic trees supporting eukaryote-to-eukaryote lateral gene transfers (LGTs). Figure illustrating the phylogenetic trees for the candidate LGTs from eukaryotes to prokaryotes supported by at least one well-supported node in the phylogenetic tree. [file gb-2013-14-2-r19-S6.PDF]
