# Supplementary material for: Patterns of prokaryotic lateral gene transfers affecting parasitic microbial eukaryotes
Source: Genome Biol. 2013 Feb 25;14(2):R19. doi: 10.1186/gb-2013-14-2-r19 (PMC4053834; doi:10.1186/gb-2013-14-2-r19)

Additional File 7. Potential Eukaryote-to-prokaryote LGTs

See legend of Additional File 5 for details, including the taxa color key. For these trees an LGT from a eukaryote to a prokaryote lineage is the most parsimonious explanation for the resulting tree topologies, or else the direction of the LGT is difficult to establish. In some trees arrows point to prokaryotic taxa that are the putative recipients of the LGT. The trees are numbered EBXXX.

Entries in the table of contents below are clickable hyperlinks to the tree figures.

21 September, 2012

Contents

EB001 2
EB002 3
EB003 4
EB004 5
EB005 6
EB006 7
EB007 8
EB008 9
EB009 10
EB010 11
EB011 12

EB001

Candy accession:	Q4CLY9_TRYCR
RefSeq accession:	XP_802735.1
Uniprot accession:	Q4CLY9_TRYCR
Comments:	LIKELY LGT INTO BACTEROIDES FROM A KINETOPLASTID
Species affected:	LM,TB,TC
Adjacent taxa in tree:	Bacteroides
EC annotation - (Blast/Profile):	EC:2.7.1.52
PHOBIUS SP:	0
PHOBIUS TMD:	0
RefSeq annotation:	fucose kinase
Name of enzyme/protein:	Fucose kinase
KEGG PATHWAY - level 1:	Carbohydrate Metabolism
KEGG PATHWAY - level 2:	Fructose and mannose metabolism, Amino sugar and nucleotide sugar metabolism

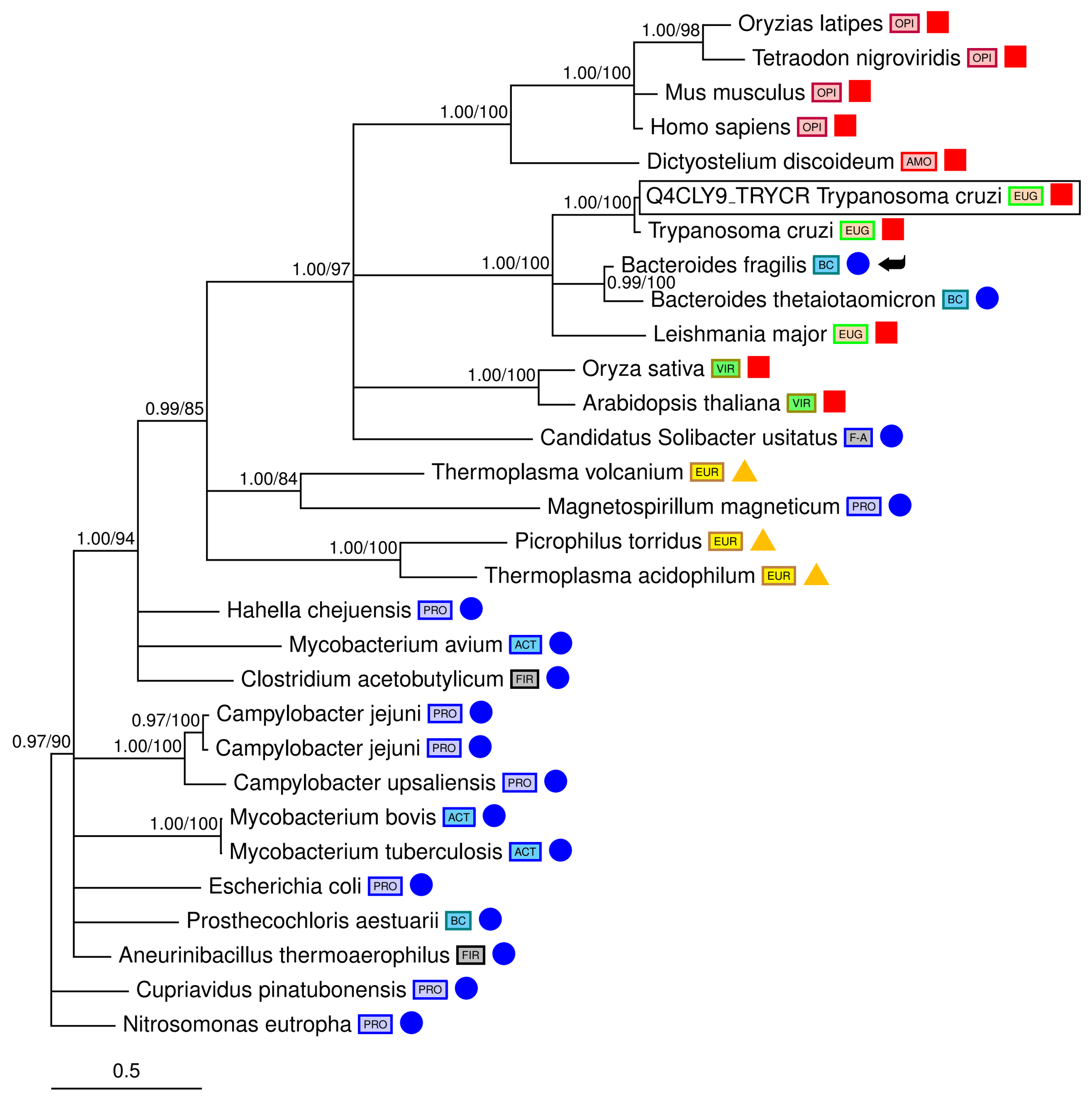

EB002

```
                 Candy accession: TV83584099
                RefSeq accession: XP_001313668.1
               Uniprot accession: A2F2U6_TRIVA
                        Comments: LIKELY LGT INTO  BACTEROIDES FROM A
                                  PARABASALA - MUCOSAL CLAN - TV, EH, TP,
                                  BF
                Species affected: TV
           Adjacent taxa in tree: Bacteroides
EC annotation - (Blast/Profile): EC:1.3.99.5
                      PHOBIUS SP: 0
                     PHOBIUS TMD: 6
               RefSeq annotation: 3-oxo-5-alpha-steroid 4-dehydrogenase
                                  family protein
        Name of enzyme/protein: 3-oxo-5alpha-steroid 4-dehydrogenase
        KEGG PATHWAY - level 1: Lipid Metabolism
        KEGG PATHWAY - level 2: Steroid hormone biosynthesis
```

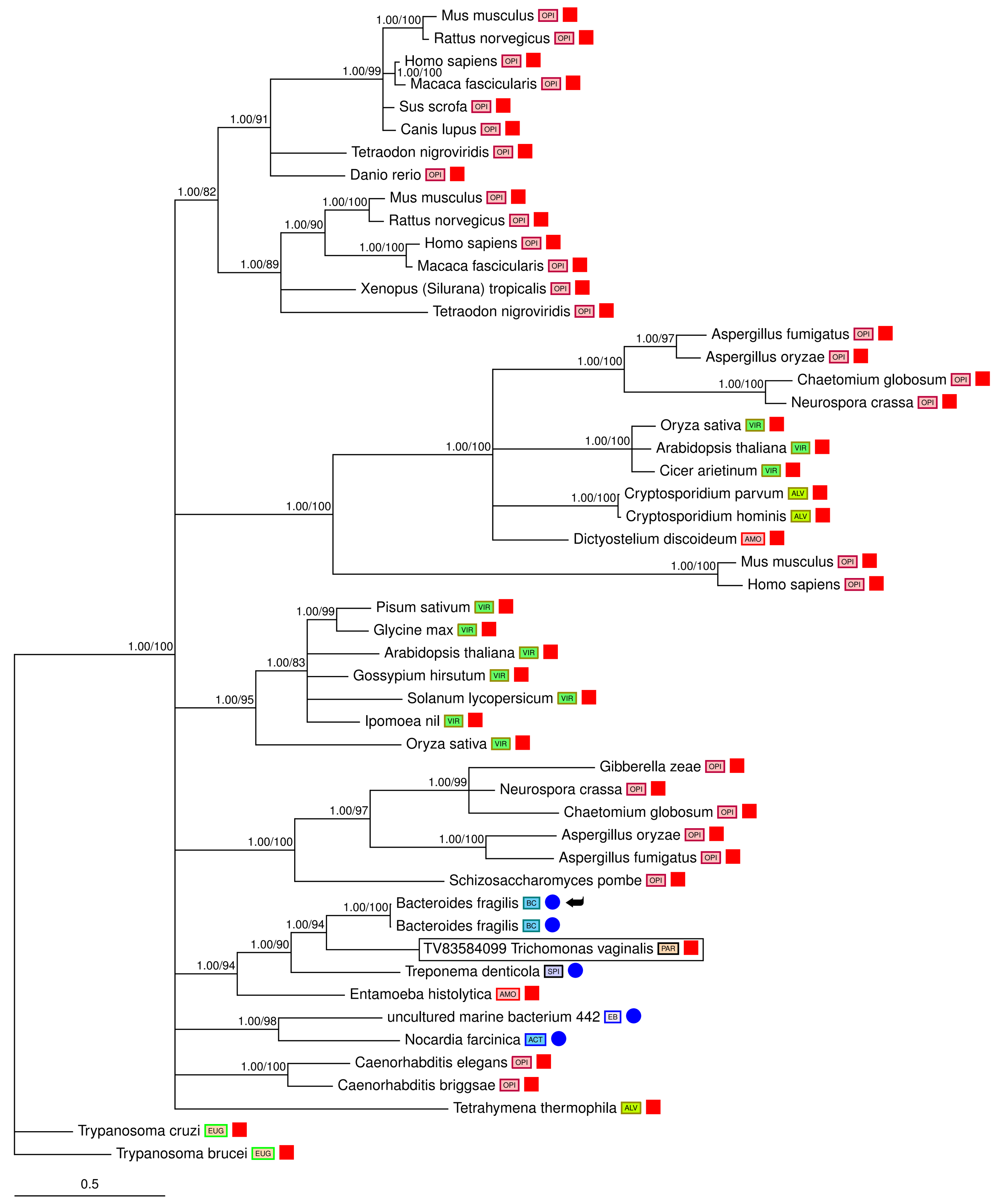

EB003

```
            Candy accession: Q4Q5Z6_LEIMA
           RefSeq accession: XP_001685252.1
          Uniprot accession: Q4Q5Z6_LEIMA
                   Comments: LIKELY LGT INTO Caulobacter vibrioides
           Species affected: LM,TB,TC
     Adjacent taxa in tree: Proteobacteria
EC annotation - (Blast/Profile): EC:1.8.1.4
                PHOBIUS SP: 0
               PHOBIUS TMD: 0
         RefSeq annotation: acetoin dehydrogenase e3 component-like
                            protein
     Name of enzyme/protein: dihydrolipoyl dehydrogenase -
                            phosphatidylinositol phosphatase
     KEGG PATHWAY - level 1: Carbohydrate Metabolism, Amino Acid
                            metabolism
     KEGG PATHWAY - level 2: Glycolysis / Gluconeogenesis, Citrate
                            cycle (TCA cycle), Glycine, serine and
                            threonine metabolism
```

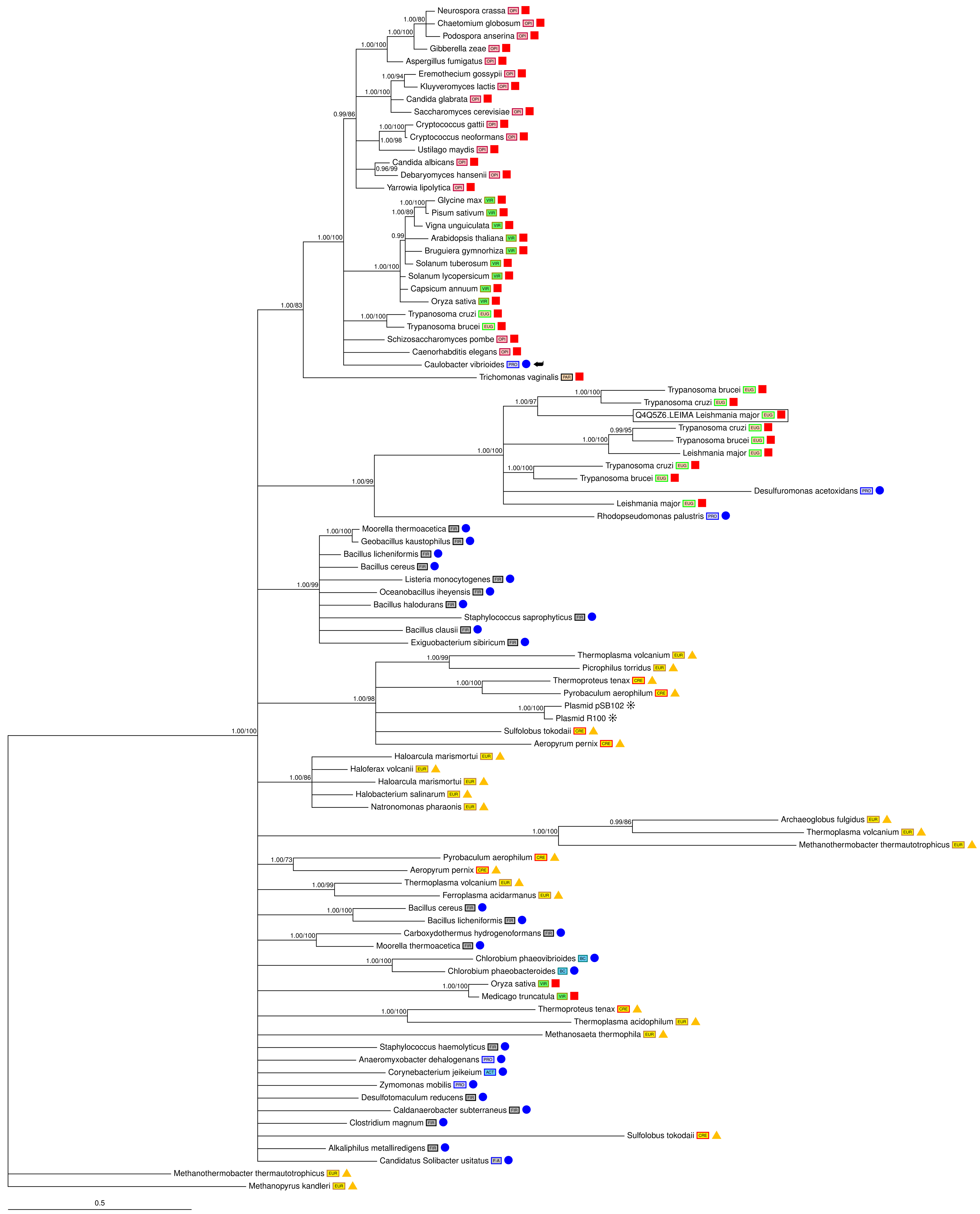

EB004

```
              Candy accession: Q54JR3_DICDI
             RefSeq accession: XP_637089.1
            Uniprot accession: Q54JR3_DICDI
                     Comments: POSSIBLE LGT INTO  RHODOPIRELLULA FROM
                               AN AMOEBOZOA
             Species affected: DD
        Adjacent taxa in tree: Planctomycetes
EC annotation - (Blast/Profile): EC:2.7.4.3
                   PHOBIUS SP: 0
                  PHOBIUS TMD: 0
            RefSeq annotation: adenylate kinase
      Name of enzyme/protein: Adenylate kinase
      KEGG PATHWAY - level 1: Nucleotide Metabolism
      KEGG PATHWAY - level 2: Purine metabolism
```

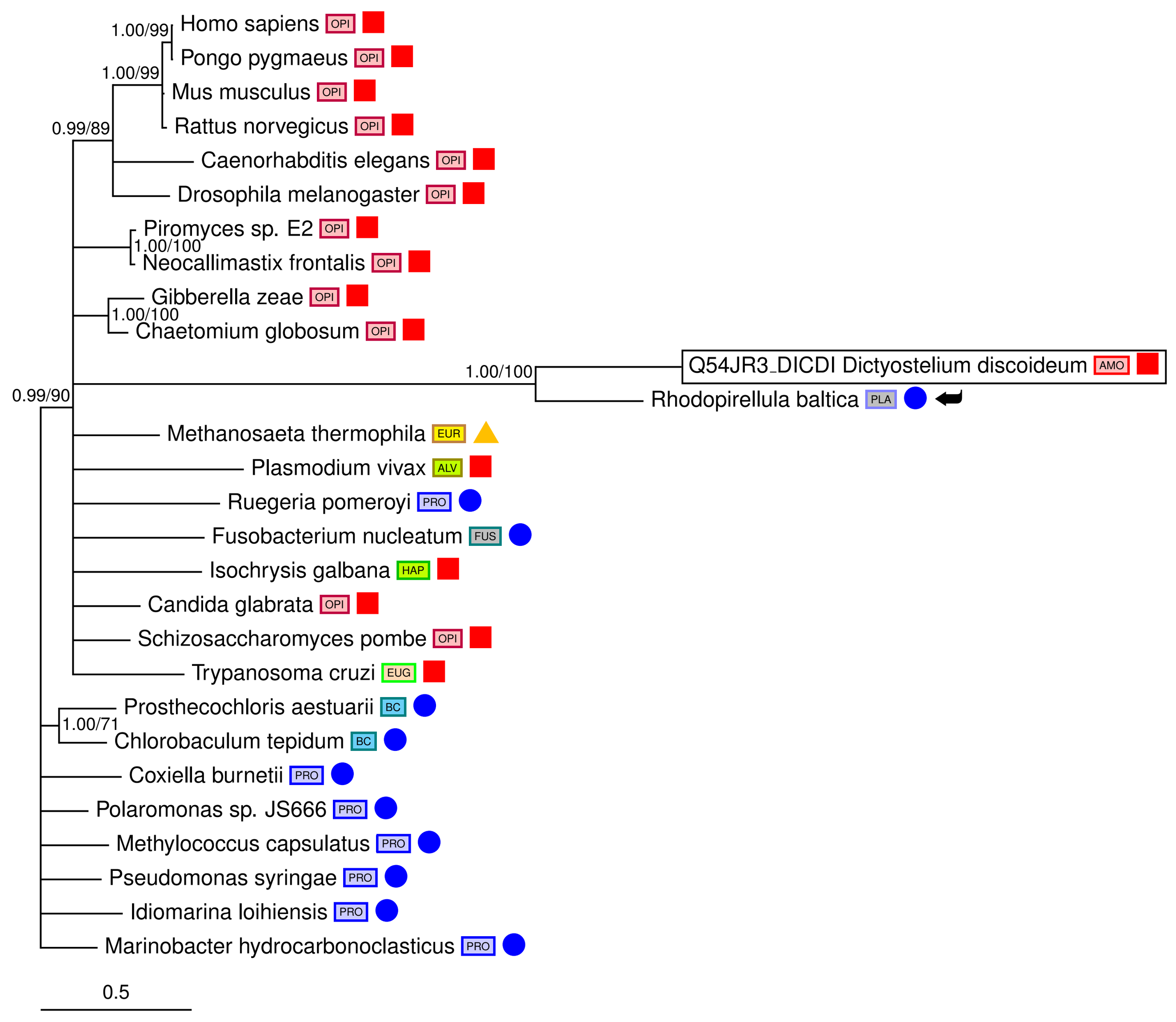

EB005

Candy accession: Q55BE9_DICDI
RefSeq accession: XP_645663.1
Uniprot accession: Q55BE9_DICDI
Comments: POSSIBLE LGT INTO BACTERIA FROM AN AMOEBOZOA
Species affected: EH
Adjacent taxa in tree: BacteriaL
EC annotation - (Blast/Profile): EC:3.1.4.46
PHOBIUS SP: 0
PHOBIUS TMD: 2
RefSeq annotation: hypothetical protein DDB_G0271374
Name of enzyme/protein: glycerophosphodiester phosphodiesterase
KEGG PATHWAY - level 1: Lipid Metabolism
KEGG PATHWAY - level 2: Glycerophospholipid metabolism

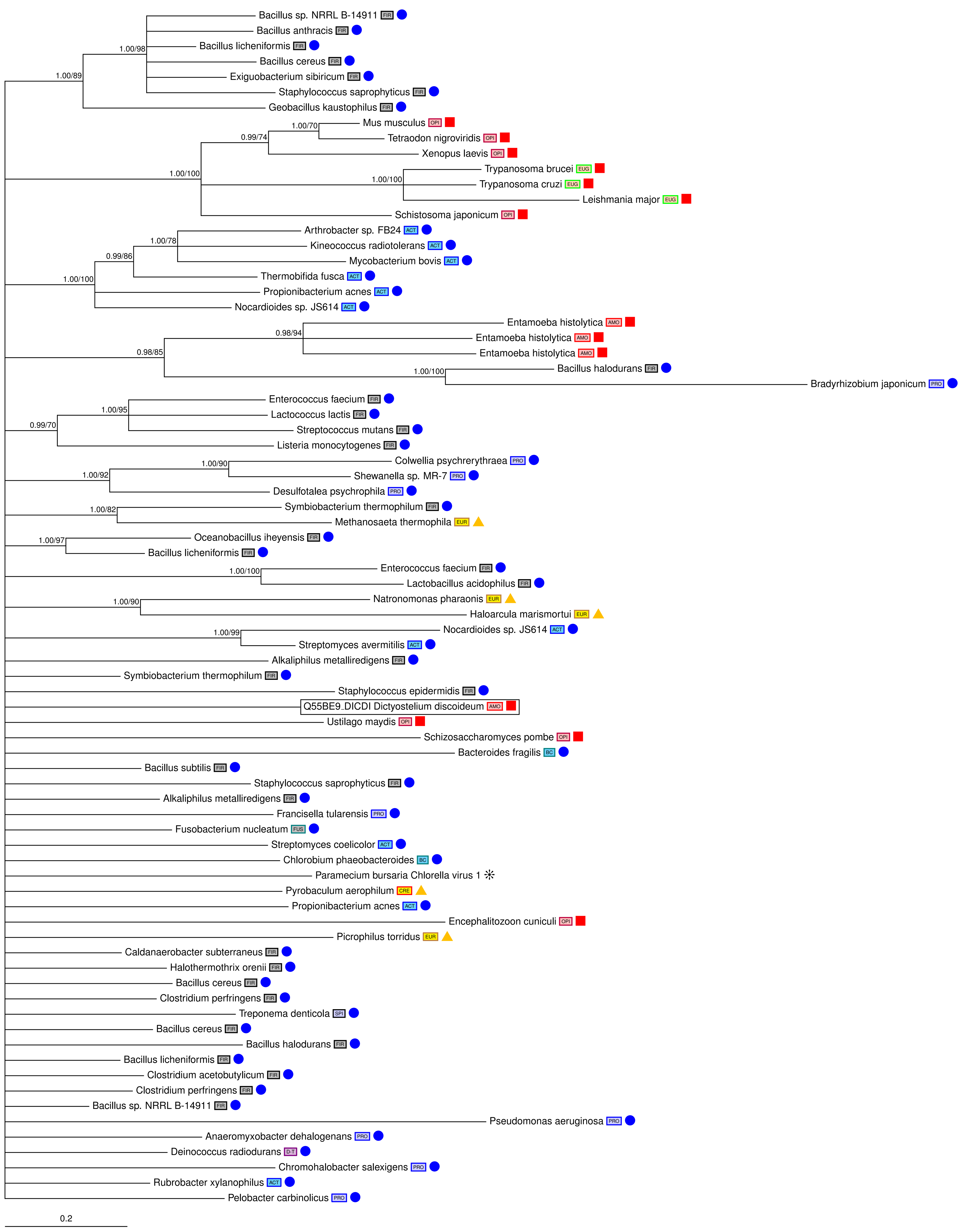

EB006

```
               Candy accession: Q511Z1_ENTHI
              RefSeq accession: XP_653045.1
             Uniprot accession: C4MAP6_ENTHI
                      Comments: LIKELY LGT INTO BACTEROIDES FROM A
                                EUKARYOTE
              Species affected: EH
         Adjacent taxa in tree: Bacteroides
EC annotation - (Blast/Profile): na
                    PHOBIUS SP: 0
                   PHOBIUS TMD: 0
             RefSeq annotation: kinase, PfkB family
         Name of enzyme/protein: kinase, PfkB family
        KEGG PATHWAY - level 1: Hypothetical proteins
        KEGG PATHWAY - level 2: Hypothetical proteins
```

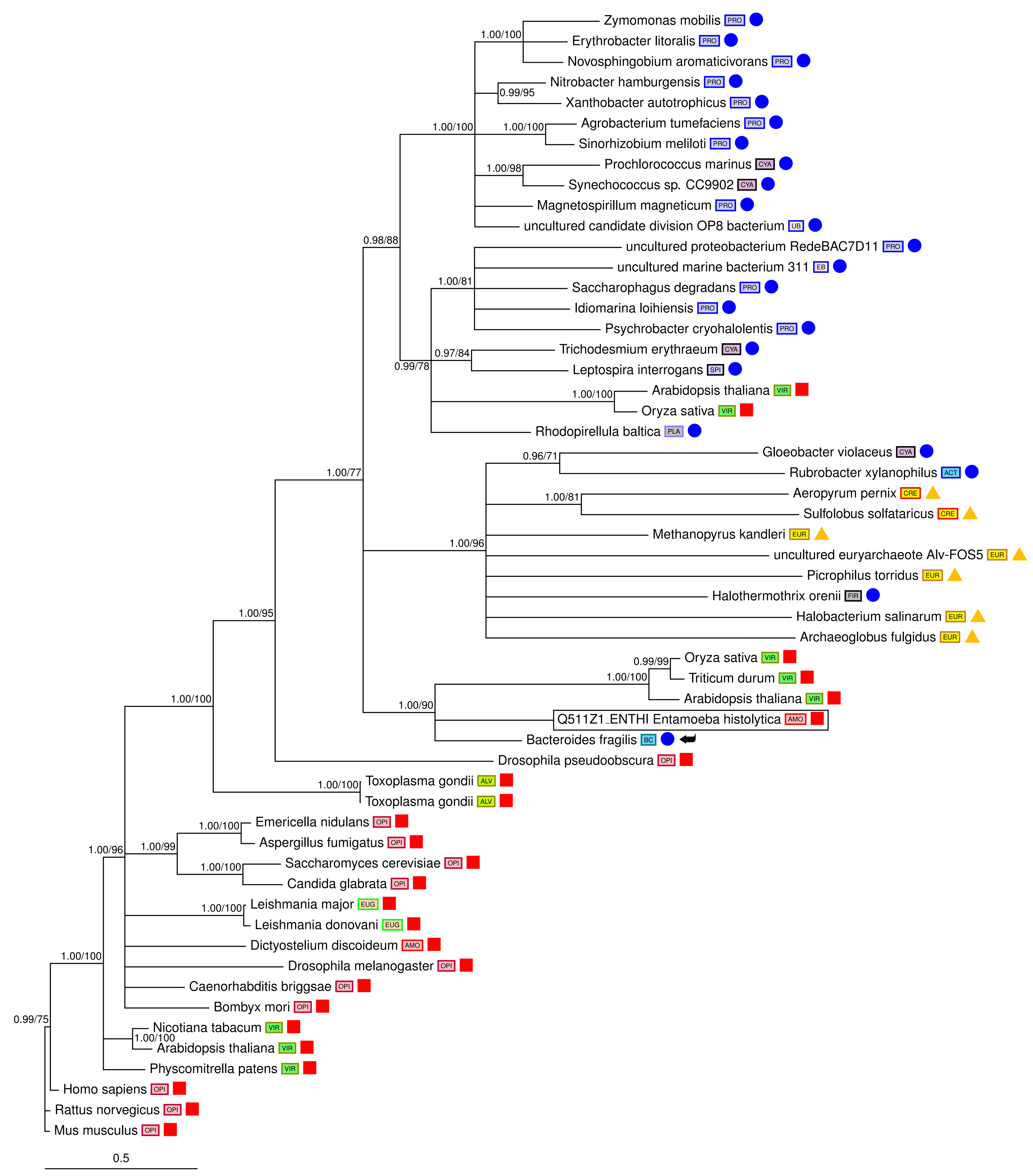

EB007

```
                 Candy accession: Q50MN5_ENTHI
                RefSeq accession: XP_649089.1
               Uniprot accession: C4LXC4_ENTHI
                        Comments: POSSIBLE LGT INTO CAMPYLOBACTER FROM A
                                  PARABASALA
                Species affected: TV
            Adjacent taxa in tree: Campylobacter
EC annotation - (Blast/Profile): EC:2.7.1.40
                     PHOBIUS SP: 0
                    PHOBIUS TMD: 0
              RefSeq annotation: hypothetical protein
         Name of enzyme/protein: Pyruvate kinase
         KEGG PATHWAY - level 1: Carbohydrate Metabolism, Nucleotide
                                 Metabolism
         KEGG PATHWAY - level 2: Glycolysis / Gluconeogenesis,  Purine
                                 metabolism, Pyruvate metabolism
```

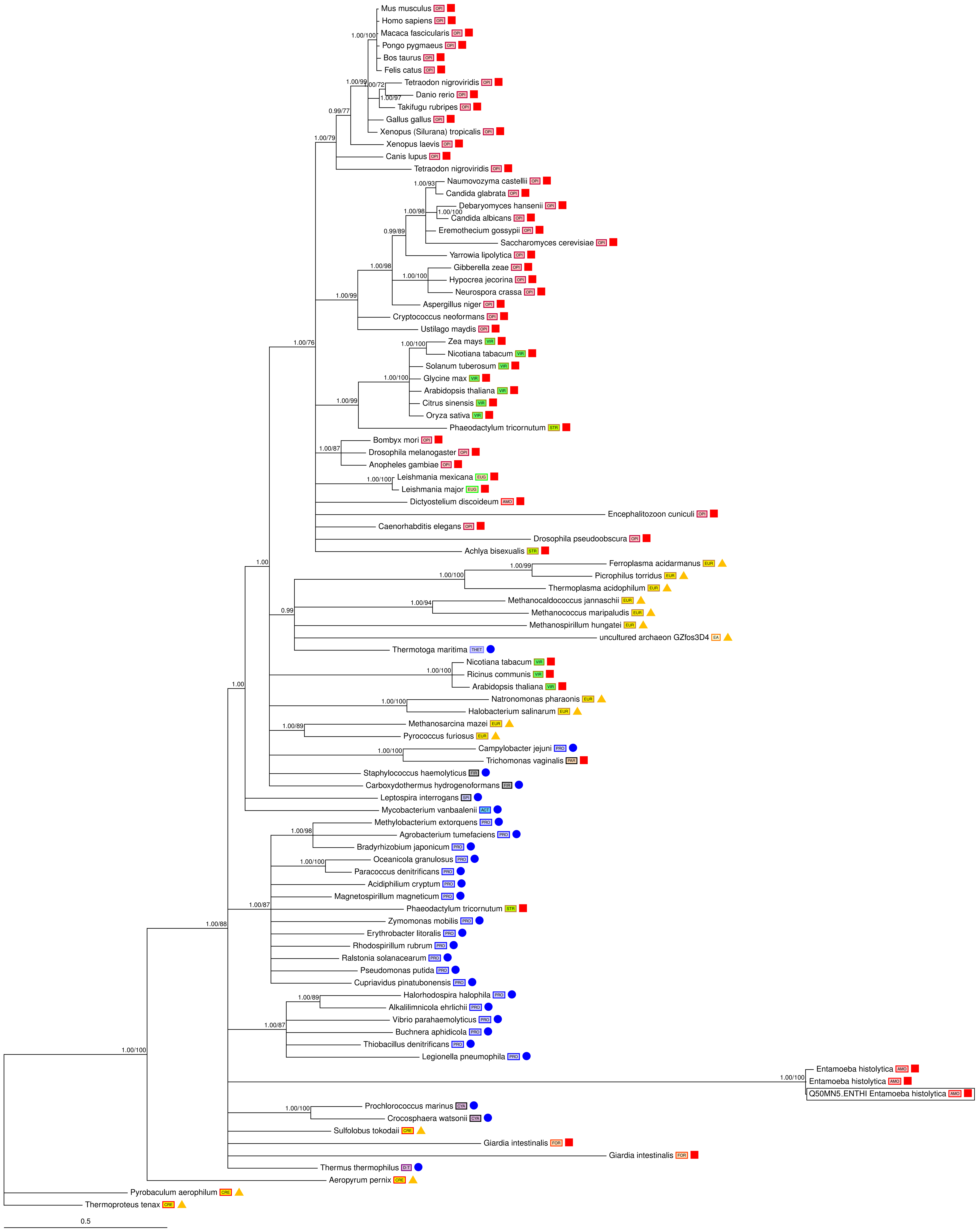

EB008

```
           Candy accession: Q4DTA6_TRYCR
          RefSeq accession: XP_817608.1
         Uniprot accession: Q4DTA6_TRYCR
                  Comments: POSSIBLE LGT INTO BACTERIA FROM A
                            KINETOPLASTID
          Species affected: LM,TC
     Adjacent taxa in tree: Protobacteria
EC annotation - (Blast/Profile): EC:1.6.99.1
               PHOBIUS SP: 0
              PHOBIUS TMD: 0
         RefSeq annotation: prostaglandin F2alpha synthase
      Name of enzyme/protein: NADPH dehydrogenase
     KEGG PATHWAY - level 1: Carbohydrate Metabolism
     KEGG PATHWAY - level 2: Fructose and mannose metabolism,  Amino
                            sugar and nucleotide sugar metabolism
```

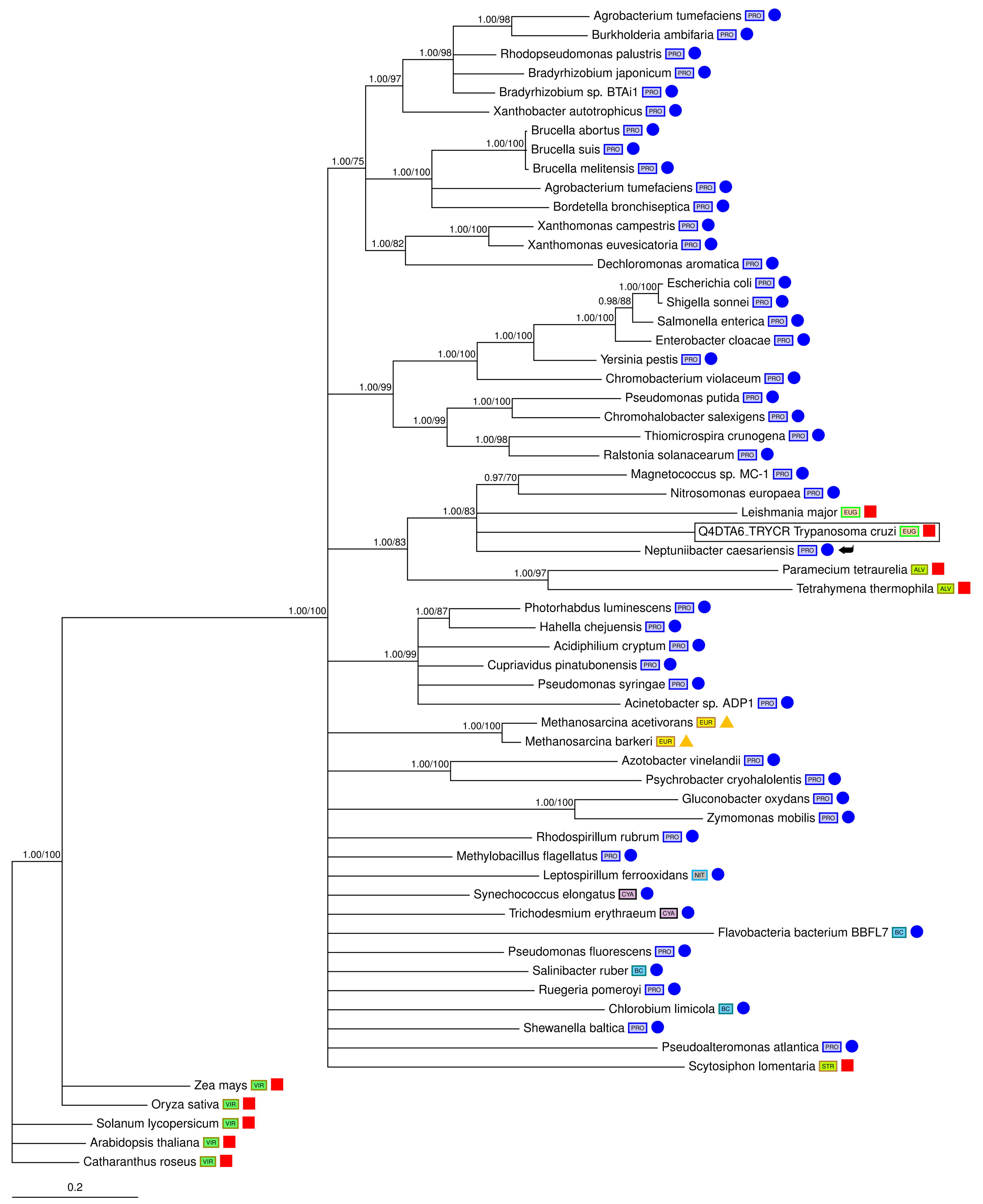

EB009

Candy accession:	Q86IG3_DICDI
RefSeq accession:	XP_644904.1
Uniprot accession:	Q86IG3_DICDI
Comments:	POSSIBLE LGT INTO BACTERIA FROM A EUKARYOTE
Species affected:	NA
Adjacent taxa in tree:	Planctomycetes
EC annotation - (Blast/Profile):	EC:3.1.3.-
PHOBIUS SP:	0
PHOBIUS TMD:	1
RefSeq annotation:	phosphatidylinositol phosphatase
Name of enzyme/protein:	phosphatidylinositol phosphatase
KEGG PATHWAY - level 1:	Reaction
KEGG PATHWAY - level 2:	Reaction

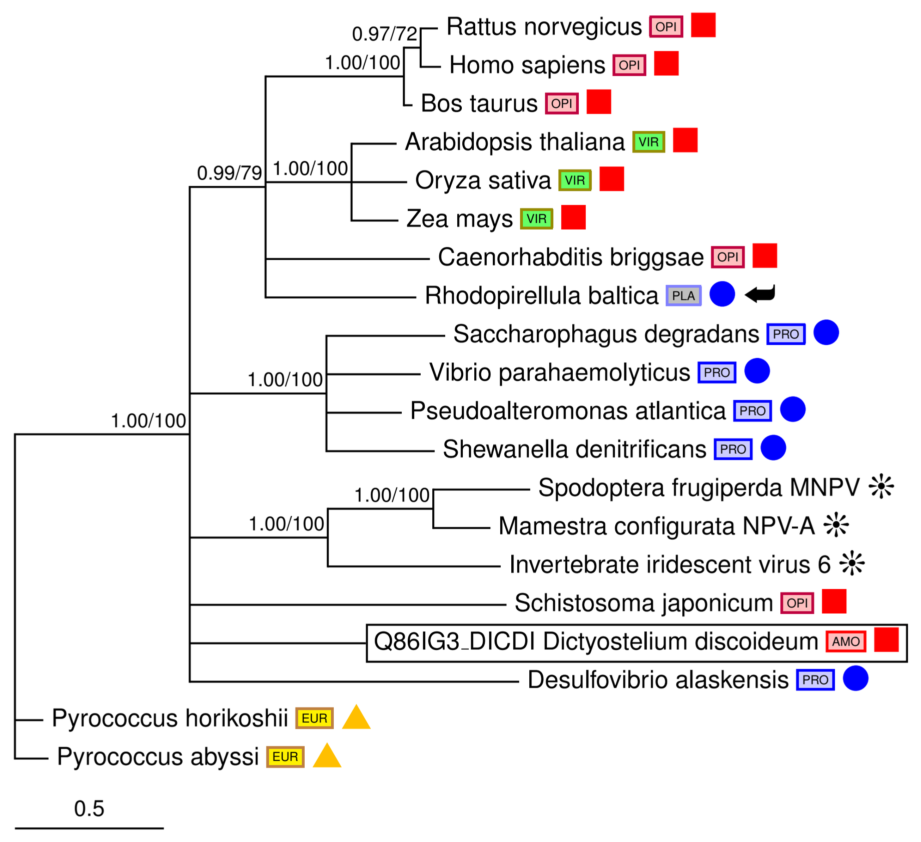

EB010

```
                Candy accession: TV81444064
               RefSeq accession: XP_001308053.1
              Uniprot accession: A2FJ08_TRIVA
                       Comments: POSSIBLE LGT INTO TREPONEMA FROM A
                                 PARABASALA
               Species affected: TV
           Adjacent taxa in tree: Treponema
EC annotation - (Blast/Profile): na
                     PHOBIUS SP: Y
                    PHOBIUS TMD: 0
              RefSeq annotation: hypothetical protein
        Name of enzyme/protein: Hypothetical proteins
        KEGG PATHWAY - level 1: Hypothetical proteins
        KEGG PATHWAY - level 2: Hypothetical proteins
```

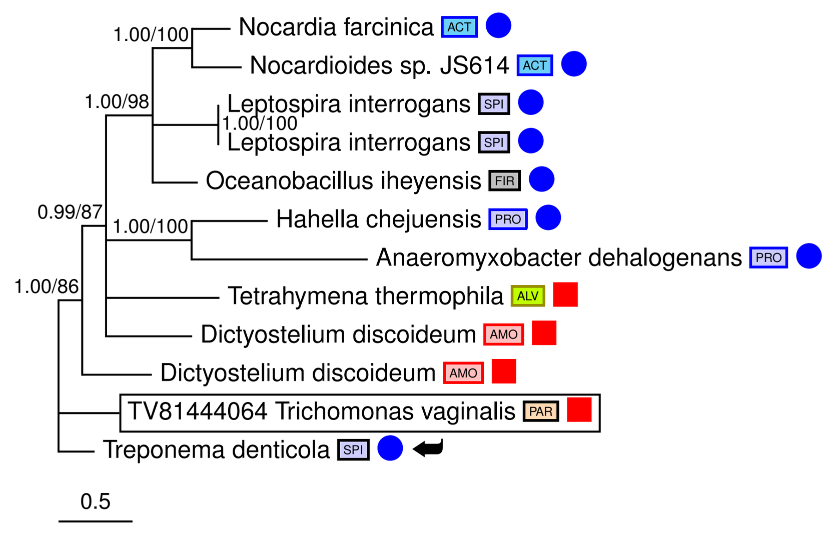

EB011

```
                Candy accession: TV93234199
               RefSeq accession: XP_001326381.1
              Uniprot accession: A2DZF4_TRIVA
                       Comments: POSSIBLE LGT INTO BACILLUS FROM A
                                 PARABASALA
               Species affected: TV
          Adjacent taxa in tree: Bacillus
EC annotation - (Blast/Profile): EC:4.6.1.13
                     PHOBIUS SP: 0
                    PHOBIUS TMD: 0
              RefSeq annotation: Phosphatidylinositol-specific
                                 phospholipase C, X domain containing
                                 protein
        Name of enzyme/protein: phosphatidylinositol diacylglycerol-
                                 lyase
        KEGG PATHWAY - level 1: Carbohydrate Metabolism
        KEGG PATHWAY - level 2: Inositol phosphate metabolism
```

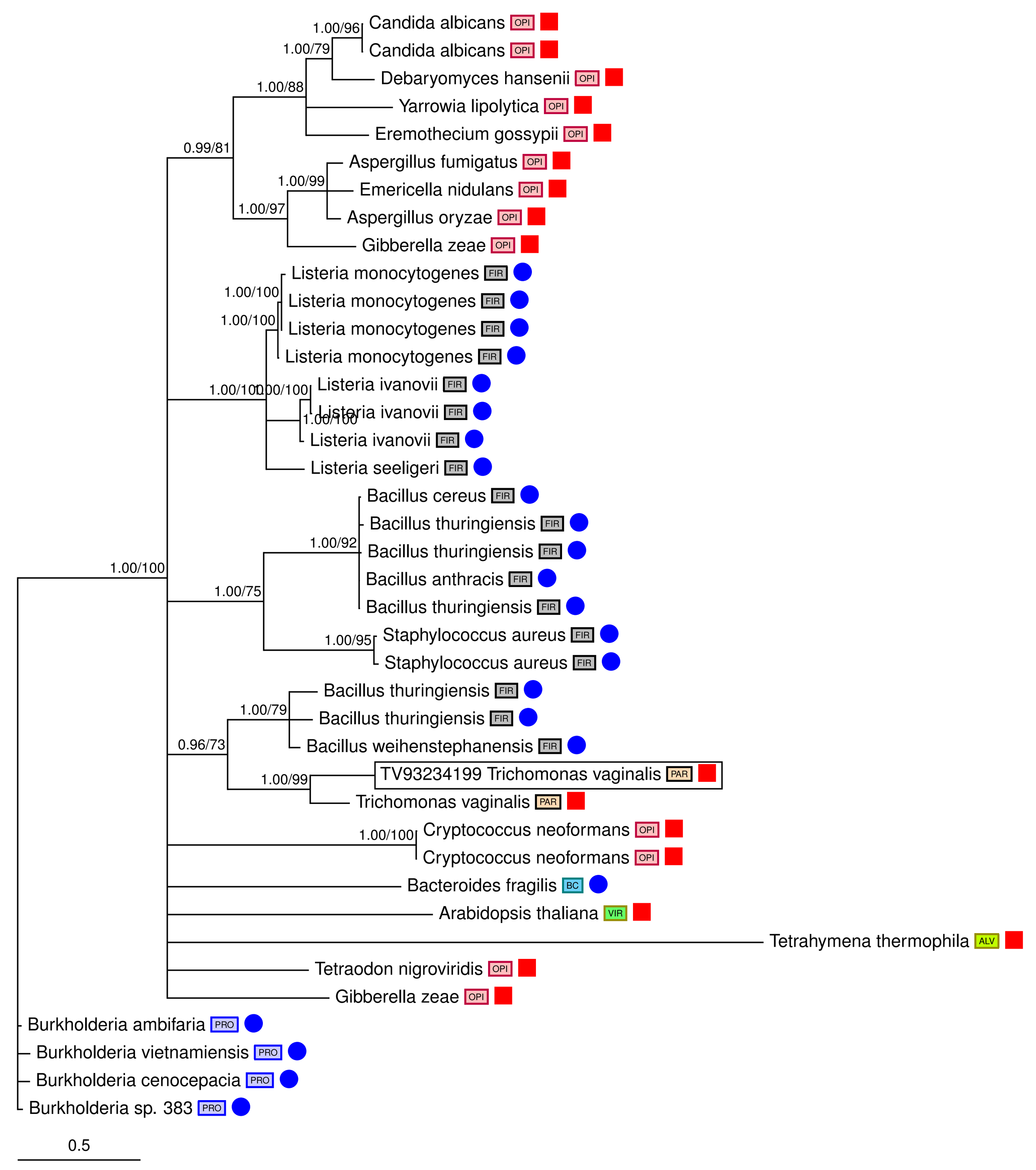

Supplement: Additional file 7 — Phylogenetic trees supporting eukaryote-to-prokaryote lateral gene transfers (LGTs). Figure illustrating the phylogenetic trees for the candidate LGTs from eukaryotes to prokaryotes supported by at least one well-supported node in the phylogenetic tree. [file gb-2013-14-2-r19-S7.PDF]
