# Supplementary material for: Patterns of prokaryotic lateral gene transfers affecting parasitic microbial eukaryotes
Source: Genome Biol. 2013 Feb 25;14(2):R19. doi: 10.1186/gb-2013-14-2-r19 (PMC4053834; doi:10.1186/gb-2013-14-2-r19)
Supplement: Additional file 9 — Relative numbers of lateral gene transfers (LGTs) to proteome size. Figure and table of the relationship between the number of identified LGTs and the number of annotated genes in each respective genome. [file gb-2013-14-2-r19-S9.PDF]

Additional File 9.

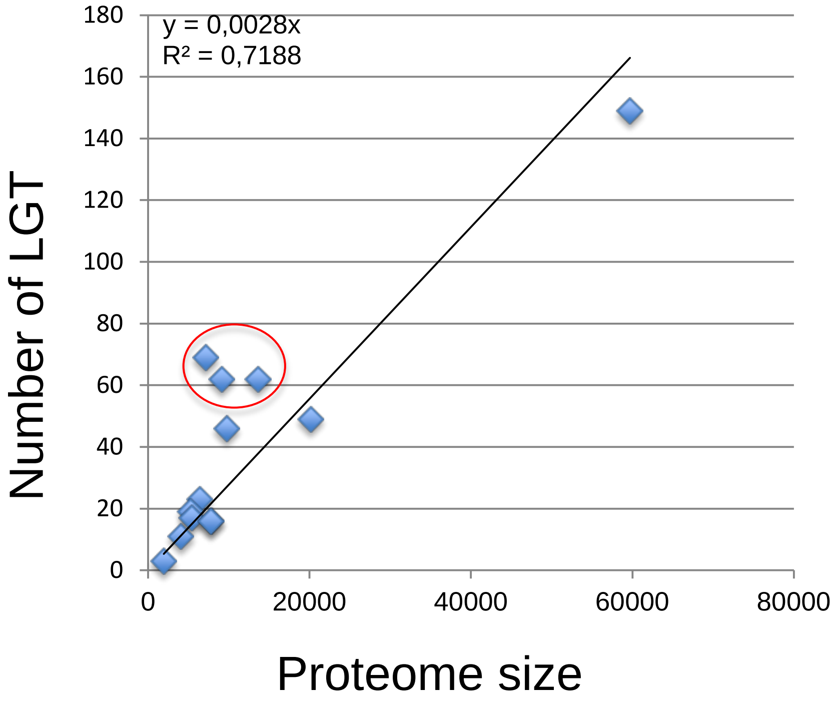

Taxa	No of annotated genes	LGT
Trichomonas vaginalis	59681	149
Leishmania major	7111	69
Dictyostelium discoideum	13605	62
Entamoeba histolytica	9090	62
Trypanosoma cruzi	20184	49
Trypanosoma bruceii	9750	46
Giardia lamblia	6394	23
Plasmodium falciparum	5258	19
Plasmodium vivax	5393	17
Plasmodium yoelii yoelii	7813	16
Toxoplasma gondii	7793	16
Cryptosporidium parvum	4074	11
Encephalitozoon cuniculi	1918	3

Additional File 9. Numbers of LGTs detected compared to proteome size. The number of LGTs per individual annotated proteome was plotted against the proteome size. A linear regression forced over the point 0:0 and calculated for the 13 available points is shown. The three most obvious outliers are circled and highlighted in red in the table below which also contains all relevant numbers.
