# Supplementary material for: Patterns of prokaryotic lateral gene transfers affecting parasitic microbial eukaryotes
Source: Genome Biol. 2013 Feb 25;14(2):R19. doi: 10.1186/gb-2013-14-2-r19 (PMC4053834; doi:10.1186/gb-2013-14-2-r19)
Supplement: Additional file 12 — Taxonomy of donor lineages for candidate lateral gene transfer (LGT) between specific subsets of protists, with extended versions and additional comparison. Diagrams presenting comparisons of donor lineages for candidate LGTs between different groups of protists. [file gb-2013-14-2-r19-S12.PDF]

Additional file 12

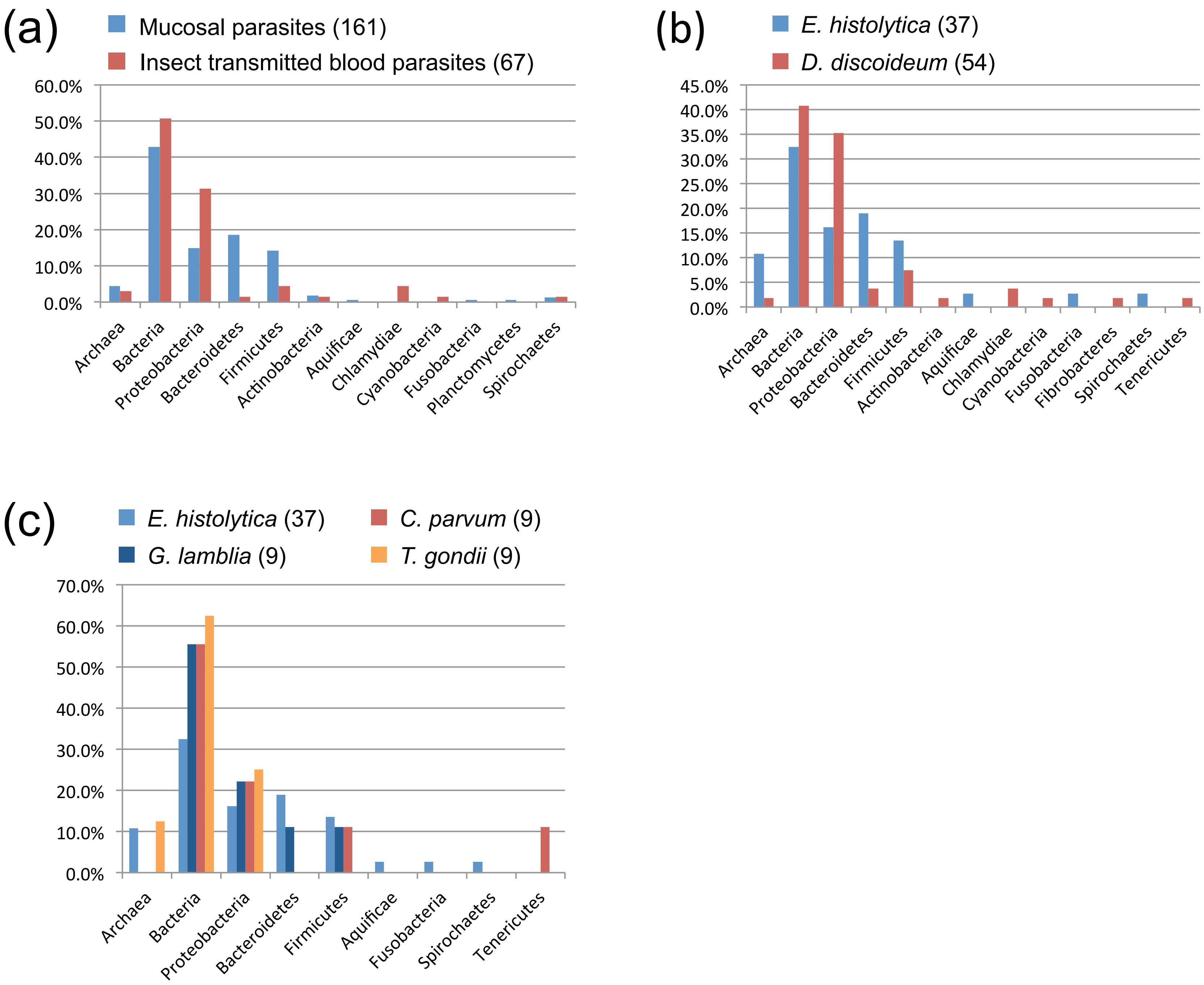

Additional file 12. Comparisons of the taxonomy of donor lineages for candidate LGTs between different group of protists.

a) Comparison of the prokaryotic lineages inferred to be donating genes to the extracellular mucosal parasites *Entamoeba histolytica*, *Trichomonas vaginalis* and *Giardia lamblia* compared to the inferred donor lineages for the insect-transmitted blood parasites *Trypanosoma brucei*, *T. cruzi*, *Plasmodium falciparum*, *P. vivax* and *P. yoelii*. A Fischer's exact test (b) Comparison of the prokaryotic lineageignoring the large unresolved "Bacteria" category) shows that the differences in donor lineages are significant: p-value = 5.4e-04 (extended version of Figure 5c).

s inferred to be donating genes to the parasite *Entamoeba* histolytica and its free-living amoebozoan relative *Dictyostelium discoideum*. A Fischer's exact test (ignoring the large unresolved "Bacteria" category) shows that the differences in donor lineages are significant: p-value = 0.011 (extended version of Figure 5d).

c) Comparison of the prokaryotic lineages inferred to be donating genes to the extracellular mucosal parasites *Entamoeba histolytica* and *Giardia lamblia* with the gut dependent apicomplexa *Cryptosporidium parvum* and *Toxoplasma gondii*. A Fisher's exact test (ignoring the large unresolved "Bacteria" category) show that there are no significant differences in donor lineages when contrasting *E. histolytica* versus *G. lamblia* (p-value = 0.935), *E. histolytica* versus *C. parvum* (p-value = 0. 354), and *E. histolytica* versus *T. gondii* (p-value = 0.329).
