# Supplementary material for: Patterns of prokaryotic lateral gene transfers affecting parasitic microbial eukaryotes
Source: Genome Biol. 2013 Feb 25;14(2):R19. doi: 10.1186/gb-2013-14-2-r19 (PMC4053834; doi:10.1186/gb-2013-14-2-r19)
Supplement: Additional file 15 — Lateral gene transfer (LGTs) affecting KEGG secondary metabolites and regulatory pathways. Figure illustrating the LGTs mapped onto the KEGG secondary metabolite and regulatory pathways. [file gb-2013-14-2-r19-S15.PDF]

Additional File 15

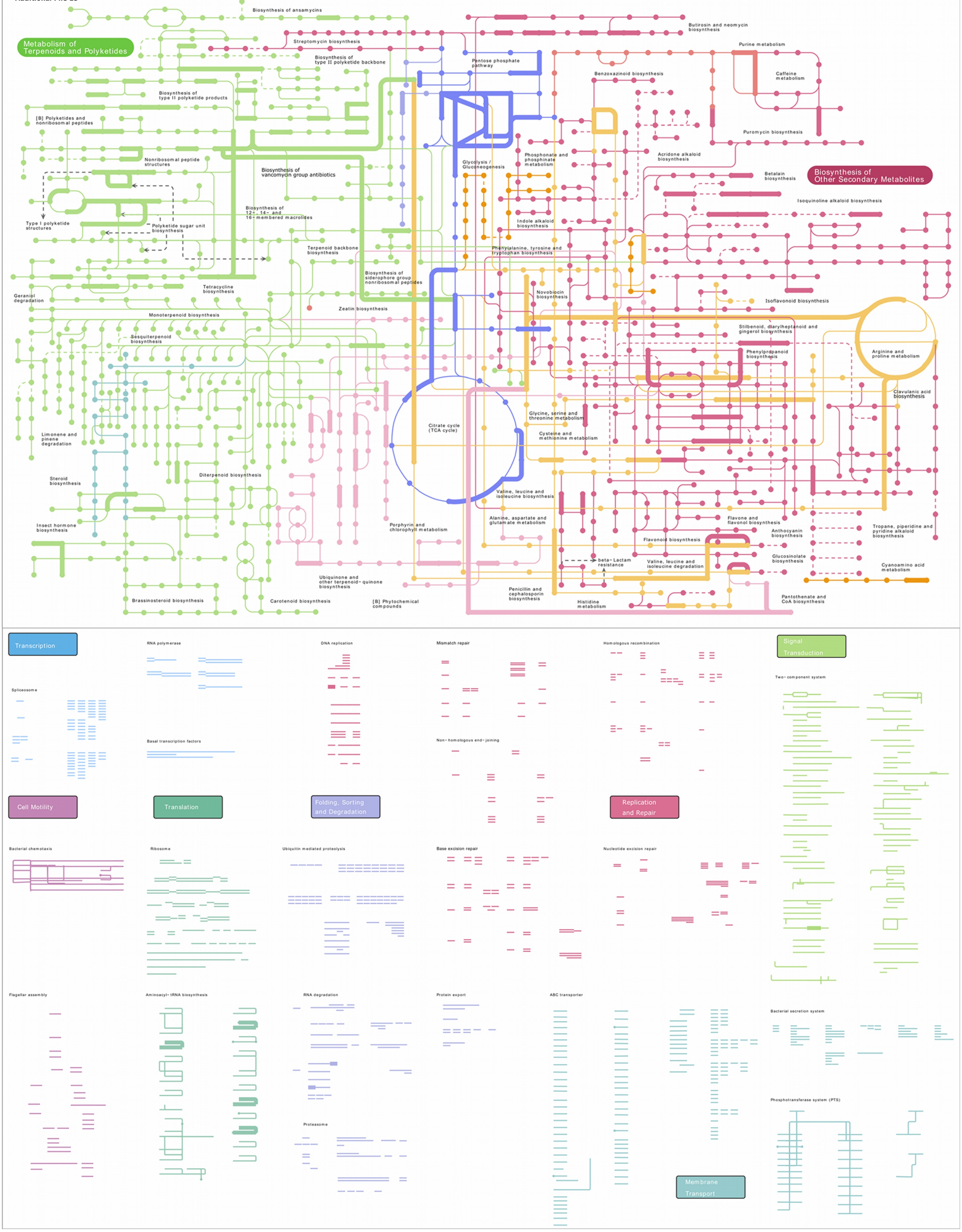

Additional file 15. Mapping of candidate LGTs onto the KEGG overview maps “Biosynthesis of secondary metabolites” and “Regulatory pathways or Functional modules”. Candidate LGTs (thick edges) were mapped onto the KEGG pathways using iPath2.0, which generated three maps: (i) “Metabolic pathways” (illustrated in Figure 2, main text), (ii) “Biosynthesis of secondary metabolites” and (iii) the “Regulatory pathways or Functional modules” overview pathways. The latter two maps are illustrated here (top and bottom panels, respectively). Genes in the first two pathways represent the more frequently transferred “operational genes” that we discuss in the main text. Relatively few candidate LGTs were identified among the entries in “Regulatory pathways or Functional modules”; these pathways contain mainly “informational genes” which are more resistant to LGT, see main text.
