# Supplementary material for: Patterns of prokaryotic lateral gene transfers affecting parasitic microbial eukaryotes
Source: Genome Biol. 2013 Feb 25;14(2):R19. doi: 10.1186/gb-2013-14-2-r19 (PMC4053834; doi:10.1186/gb-2013-14-2-r19)
Supplement: Additional file 16 — Kyoto Encyclopedia of Genes and Genomes (KEGG) pathway for the degradation of gangliosides. Figure illustrating a schematic overview of the KEGG pathway for the degradation of gangliosides. [file gb-2013-14-2-r19-S16.PDF]

Additional File 16.

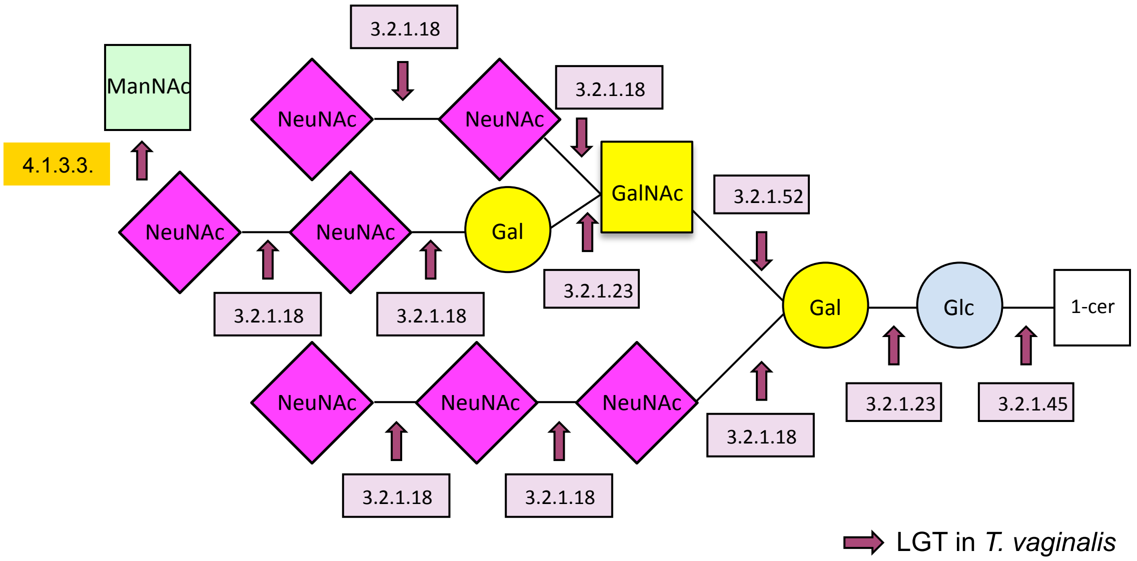

Additional File 16. Schematic overview of degradation of gangliosides. Overview of a ganglioside structure and the enzymes that can degrade it according to the KEGG pathway ec00511. A schematic ganglioside is shown with its different component parts: NeuNAc, N-acetylneuraminic acid; Gal, galactose; GlcNAc, N-acetylglucosamine; Glc, Glucose. The sites of activity of the four glycosidases required to fully degrade the ganglioside are indicated by the arrows and their respective EC numbers. These are from left to right: exo-α-sialidase (EC:3.2.1.18), β-galactosidase (EC:3.2.1.23), β-N-acetylhexosaminidase (EC: 3.2.1.52), and glucosylceramidase (EC:3.2.1.45). The table in Fig. 3b lists selected features of these enzymes. One additional enzyme (EC number in orange box), N-Acetylneuraminate lyase, which also correspond to an LGT, could contribute to the further metabolism of the sugars liberated during glycan degradation.

.
