# Supplementary material for: Patterns of prokaryotic lateral gene transfers affecting parasitic microbial eukaryotes
Source: Genome Biol. 2013 Feb 25;14(2):R19. doi: 10.1186/gb-2013-14-2-r19 (PMC4053834; doi:10.1186/gb-2013-14-2-r19)
Supplement: Additional file 17 — Table legends. Legends for tables in additional files 2, 3, 4, 8, 10, 11, 13, and 14. [file gb-2013-14-2-r19-S17.PDF]

Additional files 2-4, 8, 10, 11, 13 and 14 - table legends

Patterns of prokaryotic lateral gene transfers affecting parasitic microbial eukaryotes

Cecilia Alsmark[1,2], Peter G. Foster[3], Thomas Sicheritz-Ponten[4], Sirintra Nakjang[1], T. Martin Embley[1], Robert P. Hirt[1]

Additional files 2-4. Definitions of column headings and cell content abbreviations.

Tree number: Identification tag for LGT used in text

RefSeq accession: GenBank RefSeq accession numbers

UniProt ID: UniProt identification

LGT class: **Additional file 2**: One-node or two-nodes LGTs, referring to minimum number of supported nodes separating the query gene from other eukaryote sequences in the Bayesian consensus tree.

No of LGT in tree: The number of LGT inferred from our analyses in a given tree.

Species affected: The species affected by LGT inferred from our analyses. These are abbreviated as follows:

Cryptosporidium parvum, CP

Dictyostelium discoideum, DD

Encephalitozoon cuniculi, EC

Entamoeba histolytica, EH

Giardia lamblia, GL

Leishmania major, LM

Plasmodium falciparum, PF

Plasmodium vivax, PV

Plasmodium yoelii yoelii, PY

Toxoplasma gondii, TG

Trichomonas vaginalis, TV

Trypanosoma bruceii, TB

Trypanosoma cruzi. TC

NN in tree: Nearest neighbour to query sequence in consensus MrBayes tree.
EC annotation (BLASTP/Profile): EC numbers from the KEGG database, http://www.genome.jp/kegg/pathway.html. In some cases the annotation corresponds to a partial EC number (entries with dashes).
PHOBIUS SP: Signal peptide identified by PHOBIUS (Y: yes, or 0: nil).
PHOBIUS TMD: The number of transmembrane domain identified by PHOBIUS.
RefSeq annotation: Functional annotation from GenBank Protein database coupled to the RefSeq accession number.
Name of enzyme/protein: Annotation based on KEGG enzyme name for the entries annotated with EC numbers. For entries without EC numbers profile bases searches were used and the annotation (see Methods) of the top hit is indicated
KEGG PATHWAY - level 1:
Primary Functional annotations using the KEGG pathway database categories (first level). When the entry corresponds to a hypothetical protein, with no known function, it is labelled as such. When the entry does not correspond to one of the KEGG pathway database categories it is labelled as unclassified. "Reaction" indicated enzymes not mapped on a KEGG metabolic pathways. "Other Functions" entries without EC numbers but with an inferred function based on profile based searches.
KEGG PATHWAY - level 2:
Secondary Functional annotations using the KEGG pathway database categories (second level). When the entry corresponds to a hypothetical protein, with no known function, it is labelled as such. When the entry does not correspond to one of the KEGG pathway database categories it is labelled as unclassified.

Additional file 2. Accessions numbers and annotations of proteins for prokaryote-to-eukaryote LGTs cases supported in the Bayesian consensus trees by at least one node. The trees are illustrated in **Additional file 5**.

Additional file 3. Accessions numbers and annotations of proteins for candidate eukaryote-to-eukaryote LGTs cases supported in the Bayesian consensus trees. A set of 50 individual genes across 26 trees supported, or could not exclude, 27 eukaryote-to-eukaryote transfers with 23 topologies strongly suggesting at least one initial LGT from a prokaryotic donor for the implicated genes. Trees are illustrated in **Additional file 6**.

Additional file 4. Accessions numbers and annotations of proteins for candidate eukaryote-to-prokaryote LGT cases supported in Bayesian consensus tree. The trees are illustrated in **Additional file 7**.

Additional file 8. Comparison of LGT counts from this study contrasted with previously published LGT cases for the target taxa.

Additional file 10. The estimated number of paralogues for each LGT case listed in the **Additional files 2-4**, respectively for Prok->Euk 2 nodes LGTs (P2E_2nodes) and 1 node supported LGTs (P2E_1node) and Euk->Euk LGTs (E2E). The shown numbers correspond to the number of BLASTP consecutive best hits sequences from the query taxa to itself (cutoff e-value: 0.01). The RefSeq accession number of the query sequence is provided here and corresponds to the same entries listed in **Additional files 2** and **3**. In a number of cases there were additional hits interspersed by sequences from other taxa, these are indicated with the ≥ signs as BLASTP might not rank all the paralogues of a given taxa consecutively. Hence these numbers are likely conservative estimates of the number of paralogues.

Additional file 11. Counts for specific comparisons between selected target taxa of: (i) The functional annotations from all LGTs listed in **Additional file 2** (see Figure 1b,c). (ii) The taxonomy of the potential prokaryotic donor lineages for our target taxa from LGTs with well-supported lineages donors (see Figure 5c,d).

Additional file 13. Counts of the taxonomy of the potential prokaryotic donor lineages for LGT cases supported by at least one node (defined as the Nearest Neighbors lineage – NN – to a given target taxa in trees listed in **Additional files 2 and 5**).

Additional file 14. The number of proteins annotated in KEGG for all the 13 target genomes analysed in this study and the corresponding diversity of KEGG entries annotated enzymes. The third column indicates the number of distinct enzymes (EC numbers) for a given genome. The fourth column indicates the number of LGT cases

supported by at least one node (**Additional file 2**). From these numbers we infer an average microbial eukaryote genome encoding 12,006 proteins corresponding to 328 distinct enzymes (with EC numbers) and 38 LGTs.
